# Benchmarking Ontologies: Bigger or Better?

**DOI:** 10.1371/journal.pcbi.1001055

**Published:** 2011-01-13

**Authors:** Lixia Yao, Anna Divoli, Ilya Mayzus, James A. Evans, Andrey Rzhetsky

**Affiliations:** 1 Department of Biomedical Informatics, Columbia University, New York, New York, United States of America; 2 Institute for Genomics and Systems Biology, University of Chicago, Chicago, Illinois, United States of America; 3 Department of Medicine, University of Chicago, Chicago, Illinois, United States of America; 4 Computation Institute, University of Chicago, Chicago, Illinois, United States of America; 5 Sociology Department, University of Chicago, Chicago, Illinois, United States of America; 6 Department of Human Genetics, University of Chicago, Chicago, Illinois, United States of America; University of Colorado School of Medicine, United States of America

## Abstract

A scientific ontology is a formal representation of knowledge within a domain, typically including central concepts, their properties, and relations. With the rise of computers and high-throughput data collection, ontologies have become essential to data mining and sharing across communities in the biomedical sciences. Powerful approaches exist for testing the internal consistency of an ontology, but not for assessing the fidelity of its domain representation. We introduce a family of metrics that describe the *breadth* and *depth* with which an ontology represents its knowledge domain. We then test these metrics using (1) four of the most common medical ontologies with respect to a corpus of medical documents and (2) seven of the most popular English thesauri with respect to three corpora that sample language from medicine, news, and novels. Here we show that our approach captures the quality of ontological representation and guides efforts to narrow the breach between ontology and collective discourse within a domain. Our results also demonstrate key features of medical ontologies, English thesauri, and discourse from different domains. Medical ontologies have a small intersection, as do English thesauri. Moreover, dialects characteristic of distinct domains vary strikingly as many of the same words are used quite differently in medicine, news, and novels. As ontologies are intended to mirror the state of knowledge, our methods to tighten the fit between ontology and domain will increase their relevance for new areas of biomedical science and improve the accuracy and power of inferences computed across them.

## Author Summary

An ontology represents the concepts and their interrelation within a knowledge domain. Several ontologies have been developed in biomedicine, which provide standardized vocabularies to describe diseases, genes and gene products, physiological phenotypes, anatomical structures, and many other phenomena. Scientists use them to encode the results of complex experiments and observations and to perform integrative analysis to discover new knowledge. A remaining challenge in ontology development is how to evaluate an ontology's representation of knowledge within its scientific domain. Building on classic measures from information retrieval, we introduce a family of metrics including *breadth* and *depth* that capture the conceptual coverage and parsimony of an ontology. We test these measures using (1) four commonly used medical ontologies in relation to a corpus of medical documents and (2) seven popular English thesauri (ontologies of synonyms) with respect to text from medicine, news, and novels. Results demonstrate that both medical ontologies and English thesauri have a small overlap in concepts and relations. Our methods suggest efforts to tighten the fit between ontologies and biomedical knowledge.

## Introduction

Controlled terminologies and ontologies are indispensable for modern biomedicine [1]. Ontology was historically restricted to philosophical inquiry into the nature of existence, but logicians at the turn of the 20^th^ Century translated the term into a precise representation of knowledge using statements that highlight essential qualities, parts and relationships [2]. In the early 1970's, explicit approaches to knowledge representation emerged in artificial intelligence [3], and in the 1990's were christened ontologies in computer science [4]. These representations were promoted as stable schemas for data—a kind of object-oriented content—to facilitate data sharing and reuse. Ontologies have since been used intensively for research in biomedicine, astronomy, information science and many other areas. Biomedical scientists use ontologies to encode the results of complex experiments and observations consistently, and analysts use the resulting data to integrate and model system properties. In this way, ontologies facilitate data storage, sharing between scientists and subfields, integrative analysis, and computational reasoning across many more facts than scientists can consider with traditional means.

In addition to their computational utility, key biomedical ontologies serve as lingua franca: they allow numerous researchers to negotiate and agree on central, domain-specific concepts and their hierarchical interrelations. Concepts commonly modeled with ontologies include organismal phenotypes [5]–[7] and gene functions in genetics and genomics [1], [8]; signs, symptoms and disease classifications in medicine [9]; species, niche names and inter-species relations in ecology and evolution [10]. Building an ontology in any of these areas faces similar challenges: lack of an external standard that defines the most critical concepts and concept linkages for the ontology's proposed function; vast numbers of aliases referring to the same concept; and no yardstick with which to compare competing terminologies. This paper considers scientific ontologies generally and then develops a framework and validates a family of measures that helps to overcome these challenges.

### Proper ontologies, group ontologies and free text

The word *ontology* historically represented the product of one person's philosophical inquiry into the structure of the real world: What entities exist? What are their properties? How are they grouped and hierarchically related?

While this original definition still holds in philosophy, the computational interpretation of an ontology is a data structure typically produced by a community of researchers through a procedure that resembles the work of a standards-setting committee or a business negotiation (L. Hunter, 2010, personal communication). To agree on the meaning of shared symbols, the process involves careful utility-oriented design. The collective ontologies that result are intended to be used as practical tools, such as to support the systematic annotation of biomedical data by a large number of researchers. A standard domain-specific ontology used in the sciences today includes a set of *concepts* representing external entities, a set of *relations*, typically defined as the predicates of statements linking two concepts (such as cat *is-an* animal, cat *has-a* tail), and *taxonomy* or hierarchy defined over concepts, comprised by the union of relations. An ontology may also explicitly represent a set of properties associated with each concept and rules for these properties to be inherited from parent to child concept. Furthermore, formal ontologies sometimes incorporate explicit axioms or logical constraints that must hold in logical reasoning over ontology objects.

In practice, what different research groups mean by the term *ontology* can range from unstructured terminologies, to sets of concepts and relations without complete connection into a hierarchy, to taxonomies, to consistent, formal ontologies with defined properties and logical constraints.

An ontology developed by group represents a glimpse into the specific worldviews held within that group and its broader domain. By the same logic, we can consider the union of all published articles produced by a scientific community as a *much more complete* sample of scientific worldviews. While a research team that writes a joint paper agrees on its topic-specific worldview to some extent, its collective domain ontology is neither explicitly defined, nor free from redundancy and contradiction. Insofar as scientists communicate with each other and respond to prior published research, however, these worldviews spread and achieve substantial continuity and homogeneity [11]. A large collection of scientific documents therefore represents a mixture of partially consistent scientific worldviews. This picture is necessarily complicated by the flexibility and imprecision of natural language. Even when scientists agree on specific concepts and relations, their corresponding expressions often differ, as the same meaning can be expressed in many ways.

Nevertheless, if we accept that the published scientific record constitutes the best available trace of collective scientific worldviews, we arrive at the following conclusion: Insofar as an ontology is intended to represent knowledge within a scientific domain, it should correspond with the scientific record. Moreover, an ontology would practically benefit from evaluation and improvement based on its match with a corpus of scientific prose that represents the distribution of its (potential) users' worldviews.

### Previous work on ontology evaluation

Previously proposed metrics for ontology evaluation can be divided into four broad categories: Measures of an ontology's (1) internal *consistency* (2) *usability* (or *task-based performance*), (3) *comparison* with other ontologies and (4) *match to reality*. While this review is necessarily abbreviated, we highlight the most significant approaches to ontology evaluation.

Metrics of an ontology's *internal consistency* are nicely reviewed by Yu and colleagues [12]. They especially highlight: *clarity*, *coherence*, *extendibility*, *minimal ontological commitment*, and *minimal encoding bias* [4]; *competency* [13]; *consistency*, *completeness*, *conciseness*, *expandability*, and *sensitiveness* [14]. The names of these metrics suggest their purposes. For example, *conciseness* measures how many unique concepts and relations in an ontology have multiple names. *Consistency* quantifies the frequency with which an ontology includes concepts that share subconcepts and the number of circularity errors.

Measurements of an ontology's *usability* [15]–[17] build on empirical tools from cognitive science that assess the ease with which ontologies can be understood and deployed in specific tasks [18]. Results from such studies provide concrete suggestions for improving individual ontologies, but they are also sometimes used to compare competing ontologies. For example, Gangemi and colleagues [19] described a number of *usability-profiling measures*, such as *presence*, *amount*, *completeness*, and *reliability*, that assess the degree to which parts of an ontology are updated by ontologists [19]. The authors also discuss an ontology's “cognitive ergonomics”: an ideal ontology should be easily understood, manipulated, and exploited by its intended users.

Approaches to ontology *comparison* typically involve the 1) direct matching of ontology concepts and 2) the hierarchical arrangement of those concepts, often between an ontology computationally extracted and constructed from text and a reference or “gold standard” ontology built by experts. Concept comparison draws on the information retrieval measures of precision and recall [12], [20], [21] (sometimes called *term* [22] or *lexical* precision and recall [22]; see Materials and Methods section below for precise definitions of precision and recall). Matching ontology terms, however, raises challenging questions about the ambiguity of natural language and the imperfect relationship between terms and the concepts that underlie them. Some ignore these challenges by simply assessing precision and recall on the perfect match between terms. Others deploy string similarity techniques like stemming or edit distance to establish a fuzzy match between similar ontology terms [23], [24].

The second aspect of ontology matching involves a wide variety of structural comparisons. One approach is to measure the *Taxonomic Overlap*, or intersection between sets of super- and subconcepts associated with a concept shared in both ontologies, then averaged across all concepts to create a global measure [23]–[25]. Another uses these super and subconcept sets to construct asymmetric *taxonomic precision and recall* measures [26], closely related to *hierarchical precision* and *recall* [27], [28]. A similar approach creates an *augmented precision and recall* based on the shortest path between concepts [29] or other types of paths and a branching factor [30]. An alternate approach is the *OntoRand* index that uses a clustering logic to compare concept hierarchies containing shared concepts [31]. The relative closeness of concepts is assessed based on common ancestors or path distance, and then hierarchies are partitioned and concept partitions are compared.

Approaches for *matching an ontology to reality* are more diverse and currently depend heavily on expert participation [12]. For example, Missikoff and colleagues [32] suggested that an ontology's match to reality be evaluated by measuring each ontology concept's “frequency of use” by experts in the community. Missikoff and colleagues' ultimate goal was to converge to a consensus ontology negotiated among virtual users via a web-interface. Smith [33] recommended an approach to ontology evolution which rests on explicitly aligning ontology terms to unique entities in the world studied by scientists. Ontology developers would then be required to employ a process of manual tracking, whereby new discoveries about tracked entities would guide corresponding changes to the ontology. In a related effort, Ceusters and Smith suggested studying the evolution of ontologies over time [34]: they defined an *ontology benchmarking calculus* that follows temporal changes in the ontology as concepts are added, dropped and re-defined.

A converse approach to matching ontologies with domain knowledge appears in work that attempts to learn ontologies automatically (or with moderate input from experts) from a collection of documents [35]–[38] using machine learning and natural language processing. The best results (*F*-measure around 0.3) indicate that the problem is extremely difficult. Brewster and colleagues [36], [39] proposed (but did not implement) matching concepts of a deterministic ontology to a corpus by maximizing the posterior probability of the ontology given the corpus [36], [39]. In this framework, alternative ontologies can be compared in terms of the posterior probability conditioned on the same corpus. Their central idea, which shares our purpose but diverges in detail, is that “the ontology can be penalized for terms present in the corpus and absent in ontology, and for terms present in the ontology but absent in the corpus” (see also [19]). Each of these approaches to mapping ontologies to text face formidable challenges associated with the ambiguity of natural language. These include synonymy or multiple phrases with the same meaning; polysemy or identical expressions with different meanings; and other disjunctions between the structure of linguistic symbols and their conceptual referents.

In summary, among the several approaches developed to evaluate an ontology's consistency, usability, comparison and match to reality, metrics that evaluate consistency are the most mature among the four and have inspired a number of practical applications [40]–[42]. The approach that we propose and implement here belongs to the less developed areas of matching ontologies to each other and to discourse in the world. When considering approaches that compare ontologies to each other and to discourse, metrics comparing ontologies to one another jump from the comparison of individual concepts to the comparison of entire concept hierarchies without considering intermediate concept-to-concept relationships. This is notable because discourse typically only expresses concepts and concept relationships, and so the measures we develop will focus on these two levels in mapping ontologies to text.

Our purpose here is to formally define measures of an ontology's fit with respect to published knowledge. By doing this we attempt to move beyond the tradition of comparing ontologies by size and relying on expert intuitions. Our goal is to make the evaluation of an ontology computable and to capture both the breadth and depth of its domain representation—its conceptual coverage and the parsimony or efficiency of that coverage. This will allow us to compare and improve ontologies as knowledge representations. To test our approach, we initially analyzed four of the most commonly used medical ontologies against a large corpus of medical abstracts. To facilitate testing multiple ontologies in reference to multiple domains we also analyzed seven synonym dictionaries or thesauri—legitimate if unusual ontologies [43]—and compared their fit to three distinctive corpora: medical abstracts, news articles, and 19-century novels in English.

### Medical ontologies

Medical ontologies have become prominent in recent years, not only for medical researchers but also physicians, hospitals and insurance companies. Medical ontologies link disease concepts and properties together in a coherent system and are used to index the biomedical literature, classify patient disease, and facilitate the standardization of hospital records and the analysis of health risks and benefits. Terminologies and taxonomies characterized by hierarchical inclusion of one or a few relationship types (e.g., disease_concept_x_ *is-a* disease_concept_y_) are often considered *lightweight* ontologies and are the most commonly used in medicine [44], [45]. *Heavyweight* ontologies capture a broader range of biomedical connections and contain formal axioms and constraints to characterize entities and relationships distinctive to the domain. These are becoming more popular in biomedical research, including the Foundational Model of Anatomy [46] with its diverse physical relations between anatomical components.

The first, widely used medical ontology was Jacques Bertillon's taxonomic Classification of Causes of Death, adopted in 1893 by the International Statistical Institute to track disease for public health purposes [47]. Five years later, at a meeting of the American Public Health Association in Ottawa, the Bertillon Classification was recommended for use by registrars throughout North America. It was simultaneously adopted by several Western European and South American countries and updated every ten years. In the wake of Bertillon's death in 1922, the Statistics Institute and the health section of the League of Nations drafted proposals for new versions and the ontology was renamed the International List of Causes of Death (ICD). In 1938 the ICD widened from mortality to morbidity [48] and was eventually taken up by hospitals and insurance companies for billing purposes. At roughly the same time, other ontologies emerged, including the Quarterly Cumulative Index Medicus Subject Headings, which eventually gave rise to the Medical Subject Headings (MeSH) that the NIH's National Library of Medicine uses to annotate biomedical research literature [49], [50]. By 1986 several medical ontologies were in wide use and the National Library of Medicine began the Unified Medical Language System (UMLS) project in order to link many of them to facilitate information retrieval and integrative analysis [51]. By far the most frequently cited ontology today in biomedicine is the Gene Ontology (GO), a structurally lightweight taxonomy begun in 1998 that now comprises over 22,000 entities biologists use to characterize gene products [52].

### Thesaurus as ontology

We propose to further test and evaluate our ontology metrics using the fit between a synonym dictionary or thesaurus and a corpus. A thesaurus is a set of words (concepts) connected by synonymy and occasionally antonymy. Because synonymy constitutes an *is-equivalent-to* relationship (i.e., *word*_x_ *is-equivalent-to word*_y_), thesauri can be viewed as ontologies, albeit rudimentary ones. Moreover, because a given thesaurus is intended to describe the substitution of words in a domain of language, the relationship between a thesaurus and a corpus provides a powerful model for developing and testing general measures of the fit between ontology and knowledge domain. Most useful for our purposes, the balance between theoretical coverage and parsimony is captured with the thesauri model: A bloated 100,000 word thesaurus is clearly not superior to one with 20,000 entries efficiently tuned to its domain. A writer using the larger thesaurus would not only be inconvenienced by needing to leaf through more irrelevant headwords (the word headings followed by lists of synonyms), but be challenged by needing to avoid inappropriate synonyms.

Synonymy is transitive but not necessarily symmetric – the headword is sometimes more general than its substitute. Occasionally thesauri also include antonyms, i.e., *is-the-opposite-of*, but fewer words have antonyms and for those that do, antonyms listed are far fewer than synonyms.

A typical thesaurus differs from a typical scientific ontology. While ontologies often include many types of relations, thesauri contain only one or two. Thesauri capture the natural diversity of concepts but are not optimized for non-redundancy and frequently contain cycles. Any two exchangeable words, each the other's synonym, constitute a cycle. As such, thesauri are not consistent, rational structures across which strict, logical inference is possible. They instead represent a wide sample of conflicting linguistic choices that represent a combination of historical association and neural predisposition. Despite these differences, we believe thesauri are insightful models of modern, domain-specific ontologies. Working with thesauri also contributes practically to evaluating the match between ontologies and discourse. Because all of our measures depend on mapping concepts from ontology to text, assessment of the match between thesaurus and text can directly improve our identification of ontology concepts via synonymy.

## Results

### Overview of analysis

**Figure 1:**
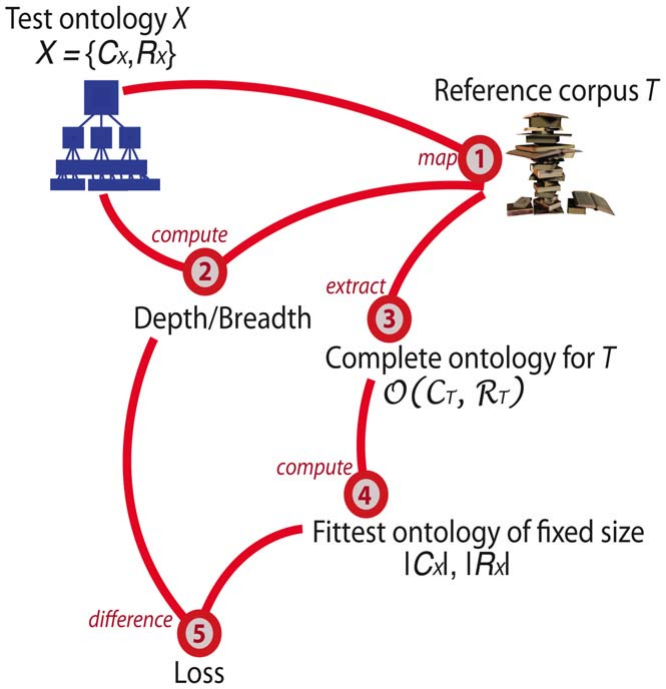
An overview of our proposed approach to benchmarking ontologies. The test ontology, *X*, is represented as a set of concepts and set of relations, *C_X_* and *R_X_* respectively, and is compared to domain-specific reference corpus, *T*. Our analysis begins by mapping concepts and relations of *X* to *T* using natural language processing tools (step 1). This mapping allows us to estimate from the text a set of concept- and relation-specific frequency parameters required for computing Breadth and Depth metrics for *X* with respect to *T* (step 2). The next step involves estimating the complete ontology for corpus *T* – an ideal ontology that includes every concept and every relation mentioned in *T* (step 3). Given the complete ontology, we can estimate the fittest ontology (a subset of the complete ontology) of the same size as the test ontology *X* (step 4) and compute the loss measures for *X* (step 5). See Materials and Methods section for precise definitions of the concepts and metrics involved.

Our proposed approach to benchmarking an ontology *X* with respect to a reference corpus *T* is outlined in Figure 1. The essence of the approach requires mapping concepts and relations of the test ontology to their mentions in the corpus – a task as important as it is difficult [53]. Given this mapping, we show how to compute ontology-specific metrics, Breadth and Depth, defined at three levels of granularity (see *Materials and Methods* *)*. We also define another important concept – the *perfect ontology* with respect to corpus *T*. This ideal ontology represents all concepts and relations mentioned in *T* and can be directly compared to *X*. If corpus *T* is sufficiently large, the perfect ontology is much larger than the test ontology *X*. This allows us to identify a subset of the perfect ontology that constitutes the *fittest ontology* of the same size as test ontology *X* –the one with maximum Breadth and Depth. Finally, given knowledge about the fittest ontology of fixed size and metrics for the test ontology *X*, we can compute loss metrics, indicating how much ontology *X* can be improved in terms of its fit to the corpus. All definitions are provided in the *Materials and Methods* section.

### Analysis of biomedical ontologies

**Figure 2:**
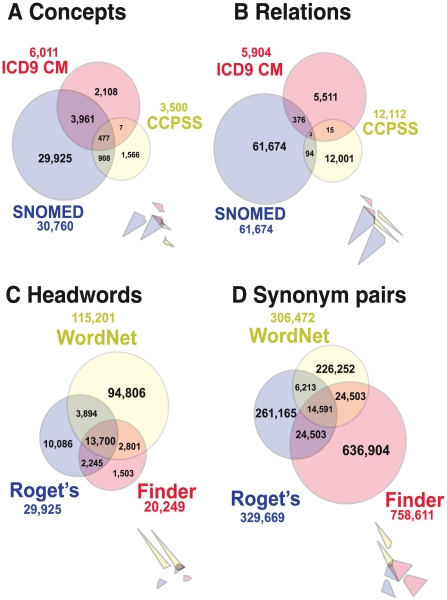
Overlap of the three largest thesauri and three medical ontologies in our study. (Inset diagrams represent modified Venn diagrams where each set is depicted in such a way that the number of elements in the set is *exactly* proportional to size of the corresponding area.) (A–B) Venn diagrams showing intersections between three of the compared medical ontologies: ICD9 CM, SNOMED and CCPSS at the level of concepts (disease and syndrome only) and at the level of relations between these concepts. (C–D) Venn diagrams showing intersections between the three largest thesauri: *WordNet*, *The Synonym Finder* (Finder), and *Webster*'*s New World Roget*'*s A–Z Thesaurus* (Roget's) at the level of headwords and synonym pairs.

To demonstrate our approach to the comparison of biomedical ontologies, we identified concepts associated with disease phenotypes and relations in four medical ontologies: ICD9-CM [48], [54], CCPSS [55], SNOMED CT [56] and MeSH (see Table 1 and Figure 2). Comparing each medical ontology concept-by-concept (as assessed with UMLS MetaMap—see Materials and Methods), we found that despite a reasonable overlap in biomedical terms and concepts, different ontologies intersect little in their relations (see Figure 2 A and B). This suggests that each ontology covers only a small subset of the full range of possible human disease concepts and circumstances. This likely results from the different ways in which each ontology is used in biomedicine.

**Table 1:** Size of biomedical ontologies and seven thesauri.

Biomedical Ontologies	Disease concepts	Disease relations
International Classification of Diseases, Clinical Modification (ICD9-CM)	6,011	5,904
Canonical Clinical Problem Statement System (CCPSS)	3,500	12,112
Systematized Nomenclature of Medicine, Clinical Terms (SNOMED-CT)	30,760	62,146
Medical Subject Headings 2009 (MeSH)	3,776	2,605

Thesauri	Headwords	Synonym pairs
The Synonym Finder [72]	20,249	758,611
Webster's New World Roget's A–Z Thesaurus [73]	29,925	329,669
21st Century Synonym and Antonym Finder [74]	7,507	146,806
The Oxford Dictionary of Synonyms and Antonyms [75]	8,487	105,902
A Dictionary of Synonyms and Antonyms [76]	3,771	57,366
Scholastic Dictionary of Synonym, Antonyms and Homonyms [77]	2,147	19,759
WordNet [78]	115,201*	306,472

*Note: WordNet is subdivided into synonymous sets (*synsets*) rather than being organized by headwords. We extracted all possible synonym pairs, which explains why WordNet contains so many headwords.

To evaluate the fit between an ontology and a corpus, we first estimated the frequency of ontology-specific concepts and relations in the corpus. We mapped ontology concepts to the biomedical literature and then estimated their frequency using MetaMap, which draws on a variety of natural language processing techniques, including tokenization, part-of-speech tagging, shallow parsing and word-sense disambiguation [57]. We then estimated the frequency of concept relations in the literature (see Materials and Methods). We parameterized these relation frequencies as the probability that two concepts co-occur within a statement in our medical corpus (see Table 2, Materials and Methods).

**Table 2:** Three corpora.

Corpus	Description	Size in words
*Medicine*	Clinical journal article abstracts from PubMed database	113,007,884
*Novels*	19th century literature—written in or translated to English	10,099,229
*News*	The Reuters corpus containing news stories published between August 20, 1996 and August 19, 1997	207,833,336

Our measures of ontology representation build on established metrics from information retrieval (IR), which have been previously used in ontology comparison. IR tallies the correspondence between a user's query and relevant documents in a collection: When the subset of relevant documents in a collection is known, one can compute IR metrics such as *recall*, *precision* and their harmonic mean, the *F*-measure, that capture the quality of a query in context (see Materials and Methods). We compute these measures as first-order comparisons between ontologies in terms of whether concept-concept pairs “retrieve” contents from the corpus.

The major rift between IR metrics and the nature of ontologies lies in the binary character of IR definitions: IR measures weight all relations in an ontology equally, but concepts and relations from an ontology vary widely in their frequency of usage within the underlying domain. Further, unlike IR documents retrieved from a query, concepts and relations present in an ontology but not a corpora should not be considered “false positives” or nonexistent in scientific discourse. Unless the ontology contains explicit errors, it is reasonable to assume that by expanding the corpus, one could eventually account for every ontology relation. Formulated differently, we cannot justifiably classify any ontology relation as false, but only improbable. This logic recommends we avoid IR measures that rely on false-positives (e.g., precision) and augment the remaining metrics to model theoretical coverage and parsimony as functions of concept and relation importance rather than mere existence in the domain of interest.

To do this, we first define the *complete ontology* that incorporates *every* concept and relation encountered in a corpus. In our implementation, we approximate this with all of the concepts and relations that appear in the corpus and are identified by UMLS MetaMap with the semantic type “disease or syndrome.” We then define two measures, *breadth* and *depth*, to describe the fit between an ontology and a corpus. *Breadth^2^* (see Materials and Methods for definition of several versions of *Breadth* and *Depth*) is a generalization of recall that substitutes *true-positives* and *false-negatives* with real-valued weights corresponding to the frequency of concepts and the probability of relations in text. *Depth^2^* normalizes breadth by the number of relations in the ontology (see Materials and Methods) and so captures the average probability mass for each ontology relation in the corpus. Large ontologies tend to have better *breadth* of coverage relative to a corpus, but not necessarily more *depth*: They may be padded with rare concepts lowering their corpus fit compared with small, efficient ontologies containing only the most frequent ones.

*Breadth* and *depth* allow us to compare ontologies of different size, but do not account for the fact that as ontologies grow, each incremental concept and relation necessarily accounts for less of the usage probability in a corpus. To address this challenge, we define the *fittest* ontology of fixed *size* (with a predetermined number of relations) such that *depth* is maximized over all possible concepts and relations. Furthermore, for an arbitrary ontology we can compute its depth *loss* relative to the *fittest* ontology of same *size* (see Materials and Methods). This approach allows us to more powerfully control for size in comparing ontologies.

Our analysis of the disease-relevant subsets of four medical ontologies indicates that CCPSS, despite having the smallest number of concepts and a moderate number of relations, performs comparably or better with respect to our clinical corpus than its larger competitors. When we consider concepts and relations jointly (see Table 3), CCPSS outperforms the three other terminologies in terms of *Breadth^2^* and *Relative Depth^2^*, while being second only to MeSH in *Depth^2^*. ICD9-CM and SNOMED rank last in *Breadth^2^* and *Depth^2^*, respectively. When only concepts (but not relations) are considered (Table 3), SNOMED CT has the greatest *Breadth^1^* and *Relative Depth^1^* but the worst *Depth^1^*, whereas MeSH and CCPSS lead in terms of *Depth^1^*. It is striking that the relatively small CCPSS matches clinical text equally or better than the three other ontologies. Table 3 also indicates that *Depth^2^ Loss* is smallest for the largest ontology, SNOMED CT and that CCPSS is next. Given its small size, CCPSS is still less likely to miss an important disease relation than MeSH or ICD9-CM. ICD9-CM, with the highest *Relative Depth^1^* ^,*2*^ *Loss*, would benefit most by substituting its lowest probability concepts with the highest probability ones missed.

**Table 3:** Comparison of three medical ontologies in terms of Breadth, Depth and (Depth) Loss, Relative Depth and Relative Depth Loss.

*Metric*	*ICD9 CM (I)*	*CCPSS* *(C)*	*SNOMED (S)*	MESH (M)
Breadth^1^	1.416×10^−2^	2.259×10^−2^	3.227×10^−2^	2.655×10^−2^
Depth^1^	2.347×10^−6^	6.454×10^−6^	1.049×10^−6^	1.019×10^−5^
Relative Depth^1^	0.365	0.605	0.818	0.687
Depth^1^ Loss	4.083×10^−6^	4.218×10^−6^	0.234×10^−6^	4.646×10^−6^
Relative Depth^1^ Loss	0.635	0.395	0.182	0.313
Breadth^2^	1.648×10^−3^	1.405×10^−2^	9.829×10^−3^	7.843×10^−3^
Depth^2^	0.279×10^−6^	1.160×10^−6^	0.158×10^−6^	2.077×10^−6^
Relative Depth^2^	0.055	0.455	0.319	0.272
Depth^2^ Loss	4.831×10^−6^	1.387×10^−6^	3.383×10^−7^	5.551×10^−6^
Relative Depth^2^ Loss	0.945	0.545	0.681	0.728

### Analysis of thesauri

In order to demonstrate the power of our metrics to capture different dimensions of the fit between ontology and knowledge domain, we compared 7 of the most common English thesauri (see Table 1 and Figure 2) against three corpora that sampled published text from the domains of medicine, news and novels (see Table 2). Our thesauri included (1) The Synonym Finder, (2) Webster's New World Roget's A–Z Thesaurus, (3) 21st Century Synonym and Antonym Finder, (4) The Oxford Dictionary of Synonyms and Antonyms, (5) A Dictionary of Synonyms and Antonyms, (6) Scholastic Dictionary of Synonym, Antonyms and Homonyms, and (7) WordNet (see Materials and Methods).

While comparing multiple thesauri word-by-word, we found a pattern similar to our medical ontologies. Despite a larger overlap in headwords than medical ontology concepts, different dictionaries intersect little in their relations. (A headword in a thesaurus is a word or phrase appearing as the heading of a list of synonyms and antonyms. Not every word or phrase that is listed as a synonym in a thesaurus also occurs as a separate headword.) On average, only one relation per headword is found in all three of the largest dictionaries (see Figures 2 C and D). This trend persists as we consider a longer list of thesauri (see Table 2 in Text S1) and indicates that any single dictionary covers only a small portion of synonyms used in the body of English. But some dictionaries are better than others.

To evaluate the fit between thesaurus and corpus, we estimated the frequencies of thesauri headwords and synonyms in the corpus. We assessed headword frequency as we did with medical ontology concepts. In the case of synonymy relations, we parameterize the synonym frequencies as the probability that a headword is substituted with each of its synonyms within a specific four-word context (see Materials and Methods).

**Figure 3:**
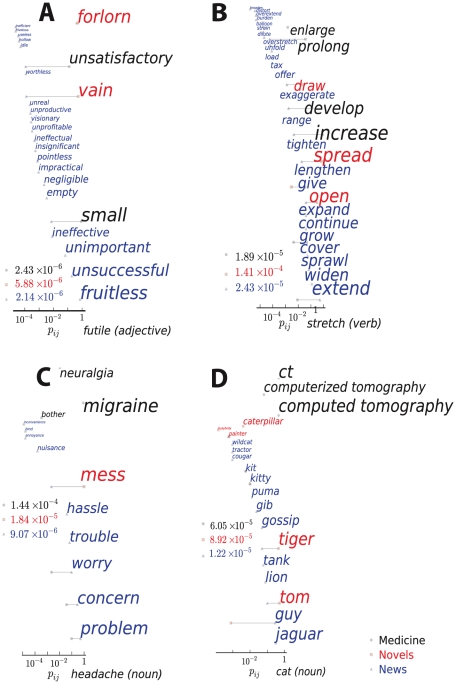
Four examples of synonym substitution probabilities in three corpora in our study. Plots A–D correspond to the headwords *futile* (adjective), *stretch* (verb), *headache* (noun) and *cat* (noun) respectively. The horizontal position of each synonym represents the substitution probability on a logarithmic scale as does the font size. The color of each synonym indicates the corpus in which the substitution is most probable: black – medicine, red – novels, and blue – news. The frequency of each headword in the three corpora is also listed using the same color codes.

**Figure 4:**
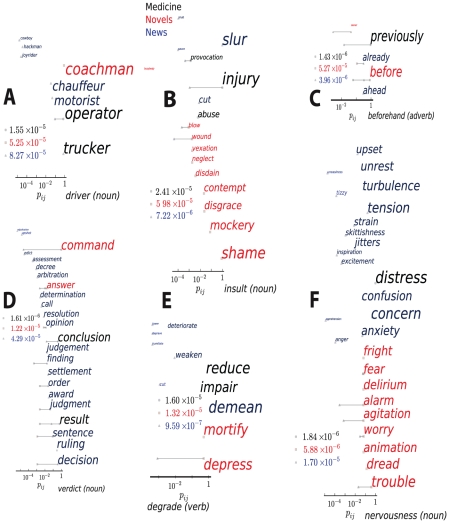
Six additional examples of synonym replacement (see **Figure 3** legend). Plots A–F correspond to the headwords *driver* (noun), *insult* (noun), *beforehand* (adverb), *verdict* (noun), *degrade* (verb) and *nervousness* (noun).

While thesauri typically aim to capture universal properties of language, corpora can be surprisingly dissimilar and sometimes disjoint in their use of words and synonym substitutions. Figures 3 and 4 visualize ten words whose synonym substitution probabilities are most unlike one another across the medicine, news and novels corpora. Some words carry a different semantic sense in each corpus (e.g., *cat* as *feline* versus *CT scan* versus *Caterpillar construction equipment*), while other words have very different distributions of common senses.

It is illuminating to consider the dominant substitutions for the three corpora: The noun *insult* translates most frequently to *injury* in Medicine, *slur* in News, and *shame* in Novels; the verb *degrade* to *impair*, *demean*, and *depress* in the same respective corpora (see Figures 3 and 4); the adjective *futile* to *small*, *fruitless* and *vain*. In some contexts words are used literally and consistently, while in others, metaphorically and widely varying. The meaning of the noun *headache* in our medical corpus is always literal: the closest synonyms here are *migraine* and *neuralgia* – with no other synonyms used. In *novels* and *news* the predominant meaning of *headache* is metaphorical. Novels are replete with *headache*'s synonym *mess*, a disordered and problematic situation (i.e., headache-inducing). The news corpus also predominantly uses *headache* to mean problem, but the most frequent synonyms are more precise and literal (*problem*, *concern*, *worry*, *trouble*). The metaphorical *mess* and *hassle* are also present, but at far lower frequencies than in novels. The verb *stretch* is treated as equivalent to *develop*, *increase*, *prolong*, and *enlarge* in the medical corpus. In *novels* it means *open*, *spread*, and *draw.* The news corpus hosts dozens of distinct synonyms for *stretch*, the most frequent three being *extend*, *widen*, and *sprawl.*

**Figure 5:**
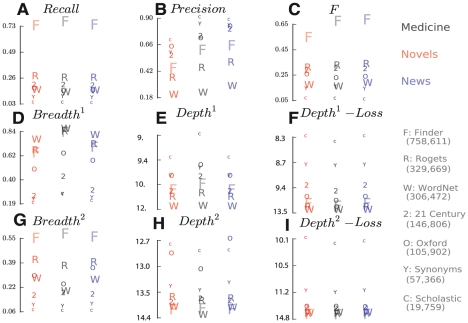
Nine metrics computed for all seven English thesauri across three corpora. The size of each dictionary symbol is proportional to the total number of synonymous relations it contains. (A, B, C) Information retrieval metrics Recall, Precision, and F-measure; (D, E, F) concept-frequency metrics Breadth^1^, Depth^1^, and Depth^1^ Loss; and (G, H, I) metrics based on frequency of both concepts and relations—Breadth^2^, Depth^2^, and Depth^2^ Loss.

Figure 5, a–i and table 2 in the *Supplement* compare all metrics discussed for all seven thesauri and three corpora. From Figure 5 d and g, we observe that our importance-based *breadth* corresponds to counts-based *recall* (a). The correspondence is not perfect, however: *Oxford* and *WordNet* have greater *breadth* than *21^st^ Century*, but this is reversed in *recall.* On the other hand, larger thesauri tend to lead in both *recall* and *breadth*, but small thesauri excel in *precision* and *depth*, as shown in Figure 5 e and h. The rankings of *depth* across all seven thesauri on three corpora, however, are very different from those of *precision*, which suggests that *depth* captures a different internal characteristic of ontology. For fixed *precision* and *recall*, we can define multiple equal-sized corpus-matched ontologies with widely varying *depth* and *breadth* by sampling from the complete ontology. The converse, however, is not true: Our *breadth* and *depth* metrics uniquely define an ontology's *precision* and *recall*. Figure 5 f and i indicate that *depth loss* is negatively correlated with the size of our seven thesauri (see Discussion). This is likely because a large thesaurus nearly exhausts the common relations in all domains by including synonyms that are rare in one context but common in another. Small dictionaries must focus. Unless explicitly tuned to a domain, they are more likely to miss important words in it.

Finally, we can compare corpora to each other with respect to all thesauri. As clearly shown in Figure 5, our three corpora map onto the seven thesauri non-uniformly. *Precision*, for example, is significantly lower across all thesauri for the *medical* corpus than for *news* or *novels*. This is likely due to the specialized and precise medical sublanguage, which renders a large portion of common synonyms irrelevant.

## Discussion

We introduced novel measures that assess the match between an ontology and discourse. These differ from former approaches to ontology comparison by focusing on concept and concept-to-concept relations, as these are the ontology elements present in textual statements. Moreover, our measures account for conceptual distinctions between comparing ontologies to one another versus to the discourse associated with a knowledge domain. In the latter comparison, the notion of a false positive, or a concept that appears in ontology but not in text is misleading, as it does not necessarily indicate the concept was not in discourse, but that the discourse was insufficiently sampled. Building on these insights, we introduce novel measures that capture the *Breadth* and *Depth* of an ontology's match to its domain with three versions of increasing complexity. *Breadth* is the total probability mass behind an ontology's concepts and relations with respect to the reference corpus. *Depth*, in contrast, is its average probability mass per concept and relation. Metaphorically, if *breadth* is “national income,” then *depth* is “income-per-capita.” An ontology with greater breadth captures more concepts and relations; an ontology with greater depth better captures its most important ones.

By measuring the match between a medical ontology and a corpus of medical documents, we are also assessing the utility of each ontology's terms and relations for annotating that corpus. In this sense, *breadth* measures the overall utility of a given ontology in annotation, whereas *depth* measures the average annotation utility per ontology constituent.

We also defined the *fittest* ontology of fixed *size* such that *depth* is maximized over all concepts and relations in order to more carefully compare ontologies of different sizes. For an arbitrary ontology we also computed its depth *loss* relative to the *fittest* ontology of same *size* (see Materials and Methods). This approach not only allows us to control for size in comparing ontologies, but also has direct application for pruning an ontology of its most improbable parts.

To illustrate the meaning and relation of depth loss to depth and breadth, imagine a casino with an enormous roulette wheel on which numbers may appear more than once, and some much more frequently than others. A gambler has limited time to observe the wheel before picking a set of numbers on which to bet. In this analogy, the numbers correspond to concepts and relations in science, the gambler to an ontologist, and a win to an efficient representation of science. The probability of winning or achieving a good scientific representation given a set of bets maps to *breadth* and the probability of winning normalized by number of bets to *depth*. The *fittest* ontology of given size is an optimal bundle of bets: the gambling ontologist can still lose by missing any particular concept or relation, but her risk is minimized. *Depth loss*, then, is the *unnecessary* risk of losing a gamble beyond that required by the constrained number of bets. As an ontology grows in size, the overall probability of missing an important scientific concept or relation shrinks. Therefore, *depth loss* will usually decrease as ontologies grow, even if the smaller ontology has greater *depth*.

By capturing the *breadth* and *depth* of an ontology's coverage, our measures suggest precisely what the analyst gains by assessing the direct match between ontology and discourse, rather than attempting to extract or “learn” an ontology from discourse and subsequently compare it with a reference ontology. When an ontology is developed from discourse, all information about the relative frequency with which concepts and relations occur in the domain is lost. Consequently, a match with such an ontology can only grossly capture the representativeness of relations in the reference ontology. The larger difference between these approaches, however, is in the position of authority. Our measures suggest that discourse is the authoritative source of a community's scientific knowledge and should be the reference against which most scientific ontologies are judged. Measures that assess “learned ontologies” with a gold standard, by contrast, assume that ontologists and their constructions are the ultimate reference.

Our approach to ontology evaluation has several limitations. It may be viewed as restrictive due to its reliance on the availability of a large corpus related to the domain of interest. This is usually not a problem for biomedical ontologies as the amount of biomedical text is typically overwhelming. For esoteric ontologies, however, it may be difficult to locate and sufficiently sample the textual domain they are intended to map. At the extreme, consider a hypothetical ontology configuring entities corresponding to a novel theory.

Further, one can imagine ontologies for which any degree of match to an external domain is meaningless. For example, a hypothetical mathematical ontology should be, first and foremost, clear and internally consistent. As is common in mathematics, relevance to external research may not be required. This level of abstraction and invariance to reality, however, is atypical for biomedicine and other areas of science where the corpus of published research indicates much of what is known.

Our approach addresses only one dimension of ontology quality: its match to collective discourse. Other quality dimensions such as consistency and usability are also clearly important. We do not advocate retiring other views of ontology quality: our measures of external validity can be used synergistically with assessments of internal validity to expand the overall utility of an ontology.

Another limitation of our method is that we assume that formal relations among ontology concepts are represented explicitly in text, like the concepts themselves. As Brewster and colleagues have pointed out [36], this is often not the case. More advanced methods are needed to improve on our use of concept co-occurrence. Our approach depends heavily on the advancement of parsing and mapping technologies to enable linkages between ontology concepts and their textual instances. It is particularly dependent on quality in the part-of-speech tagging, recognition of verb nominalization [58] and the association of inflectional and morphological variations in vocabulary.

In this way, proper application of our proposed method demands that users surmount significant technical hurdles. It is not trivial to map concepts and relations from an ontology to a real corpus considering the ambiguities and complexities of unstructured discourse. Although we believe that these technical problems can be resolved with a reasonable degree of accuracy, there remains a lingering concern that ontology evaluation is confounded by imperfections in the analysis of text. To address this concern, our analysis of synonym substitution probabilities suggests a practical approach for generating probabilistic domain-specific thesauri that can be immediately used in more closely mapping arbitrary ontologies to text. These substitution probabilities can also be deployed to improve the cross-mapping of ontologies, expanding database queries, and text mining.

Several previous approaches to ontology comparison involve explicit comparison of the entire taxonomy of relations. Our approach instead emphasizes comparison of ontology relationships individually. This is because metrics of taxonomic distance between two ontologies [23]–[28] are not easily transplanted to the comparison of ontology with text. Ontology comparisons often weight the match between concepts by the centrality of those concepts in each ontology's hierarchy [26]. The upper-level – the most central and abstract – relations in an ontology, however, are rarely mentioned explicitly in prose. This is partly because of the indexical power of context: an article published in the journal *Metabolism* does not need to mention or describe metabolism to its audience. The publication alone signals it. In contrast, specific concepts that are taxonomically close to the bottom of the hierarchy – the “leaves” of the tree – are often mentioned in text with disproportionate frequency. In short, while centrality denotes importance within an ontology, and ontology importance should correlate with frequency in discourse, we expect that this relationship is confounded in scientific domains where the most central “branching” concepts are likely so conditioned by context (e.g., a biology journal) that they remain unspoken.

In summary, our measures provide a reliable assessment of ontologies as representations of knowledge. We demonstrated their utility using biomedical ontologies, English thesauri and corpora, and we showed that different corpora call for different representations. We believe our straightforward approach can be extended to arbitrary ontologies and knowledge embedded in the literature of their communities. For example, our approach can directly assess the degree to which other popular ontologies represent published knowledge in their respective domains. Our approach would also recommend how these ontologies could be made more efficient or parsimonious. Finally, our measures facilitate comparison between competing ontologies. In conjunction with efforts to make ontologies logically consistent, greater external validity will insure that ontological inferences anchor to the most salient concepts and relations used by the community of science.

## Materials and Methods

### Data

We used four medical ontologies, seven English thesauri (Table 1), and three corpora (Table 2) from the areas of medicine, news, and novels. The four biomedical ontologies we used were ICD9-CM, CCPSS, SNOMED-CT, and MeSH each described in the following paragraphs.

*ICD9-CM* [48], [54], the *I*nternational Statistical *C*lassification of *D*iseases and Related Health Problems, is a taxonomy of signs, symptoms, abnormal findings, complaints, social circumstances, and external causes of injury or disease. It uses predominantly one type of relation (*is-a*), whereas CCPSS and SNOMED CT employ richer repertoires of relation types. The International Classification of Diseases is published by the World Health Organization (WHO) and is used worldwide for morbidity and mortality statistics, reimbursement systems, and automated decision support in medicine. The ICD9-CM version was created by the U.S. National Center for Health Statistics as an extension of the ICD9 system to include diagnostic and operative procedures – the CM referring to *clinically modified*. Here we use the 2009 version of ICD9-CM. A typical relation between two concepts in ICD9-CM looks as follows:
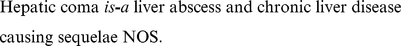



*CCPSS*, the *C*anonical *C*linical *P*roblem *S*tatement *S*ystem [55], is a knowledge base that encodes clinical problems encountered by ailing humans. It is specifically designed to encode *clinical knowledge* regarding relations between medical conditions. Typical relations encoded in CCPSS look as follows:
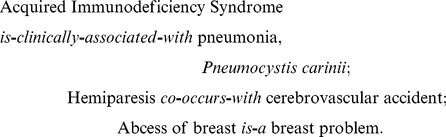



*SNOMED CT*, *S*ystematized *No*menclature of *Med*icine – *C*linical *T*erms [56], is a synthesis of terminologies produced by the College of American Pathologists and by the National Health Service of the United Kingdom. The American component is called *SNOMED Reference Terminology*, and the British one is referred to both as *Clinical Terms* and *Read Codes*. SNOMED CT is the most comprehensive clinical terminology in existence and includes ∼350,000 concepts. A typical relation in SNOMED CT looks as follows:
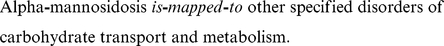



*Medical Subject Headings (MeSH)* [49] is a comprehensive controlled vocabulary designed by the United States National Library of Medicine (NLM). Its intended use is information retrieval; MeSH was not designed as a formal ontology. The 2009 version contains a total of 25,186 subject headings spanning anatomy; organism classification; diseases; chemicals and drugs; food and beverages; analytical, diagnostic and therapeutic techniques and equipment; health care, psychiatry and psychology; biological and physical sciences; anthropology, education, sociology and social phenomena; persons; technology and information science; humanities; publication characteristics and geographic locations. It is mainly used by the MEDLINE/PubMed article database for indexing journal articles and books. A typical relation present in the MeSH is-a hierarchy looks like 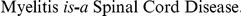



We tested the medical ontologies against a corpora of modern medicine comprised of clinical journal article abstracts from the PubMed database. We limited ourselves only to English abstracts in the core clinical journals for the entire period covered by PubMed, 1945 through February of 2009. The resulting corpus included 786,180 clinical medicine-related abstracts (see Table 2).

Our broader analysis of synonym dictionaries included seven of the most common, sampling from very different kinds of thesauri. These include the large thesauri (1) The Synonym Finder and (2) Webster's New World Roget's A–Z Thesaurus; moderately-sized thesauri (3) 21st Century Synonym and Antonym Finder and (4) The Oxford Dictionary of Synonyms and Antonyms; and portable, compact thesauri (5) A Dictionary of Synonyms and Antonyms and (6) Scholastic Dictionary of Synonym, Antonyms and Homonyms. Each thesaurus shared a common layout involving alphabetically arranged headwords followed by synonyms (and antonyms). Finally, we included the electronic dictionary (7) WordNet, which arranges its words asymmetrically into sets of synonyms or “synsets.”

To evaluate the match between these thesauri and a variety of text corpora, we added English news and novels to our sample of clinical medicine (see Table 2). The news corpus covered all Reuters news stories between 08/20/1996 and 08/19/1997. The novels corpus contained 50 of the most influential novels of the 19^th^ Century, written or translated into English. Complete information regarding each of these data sources can be found in the supplement.

### Parsing and mapping

To map biomedical concepts to our clinical corpus we used MetaMap. MetaMap [59] is a knowledge-intensive natural language processing program developed at the National Library of Medicine for mapping snippets of biomedical text to the UMLS Metathesaurus [60], [61].

MetaMap uses the SPECIALIST minimal commitment parser [62] to conduct shallow syntactic parsing of text – using the Xerox part-of-speech tagger. For each identified phrase its variants are generated using the SPECIALIST lexicon and a supplementary database of synonyms. A phrase variant comprises the original phrase tokens, all its acronyms, abbreviations, synonyms, derivational variants, meaningful combinations of these, and inflectional and spelling variants. Given a collection of phrase variants, the system retrieves from the Metathesaurus a set of candidate strings each matching one of the variant constituents. Each Metathesaurus-derived string is evaluated against the input text by a linear combination of four metrics, called centrality, variation, coverage and cohesiveness. The first two metrics quantify matches of dictionary entries to the head of the phrase, and the mean inverse distance between dictionary and text phrases. The latter two metrics measure the extent and sparsity of matches between the textual and dictionary strings. The candidate matches are then ordered according to mapping strength, and the highest-rank candidate is assigned as the final match. We used MetaMap's Strict Model to filter matches in order to achieve the highest level of accuracy [57].

The UMLS (Unified Medical Language System) Metathesaurus is a rich terminological resource for the biomedical domain [63], [64]. All concepts in the UMLS Metathesaurus are categorized into 135 semantic types (or categories). In this work we focused on the semantic type of “Disease or Syndrome”. This is why the counts of concepts and relations in Table 3 are much less than the total number of concepts and relations from each of the four ontologies in Table 1.

We used the Stanford POS tagger [65], [66] to parse the news and novels corpora comparable to MetaMap's parsing of medical texts. After parsing, we processed the inflectional and morphological variations of each word. For the medical corpus, we retrieved the base form of a word by querying the UMLS Specialist Lexicon based on its appearance in the text (e.g., singular or plural for a noun, different tenses for a verb). For the news and novels corpora, we converted all words to their base word form (e.g., translating nouns from plural to singular and verbs from past and future to present tense) with a rich set of morphological rules. Then we used these base word forms, in addition to their part of speech, to indicate word context for the calculations below. We also used these base forms to match against thesaurus entries.

### The probability of ontology relationships in text

In this section, we define several metrics for mapping an ontology to a corpus, arranging the metrics by increasing complexity. The simpler metrics do not distinguish between multiple predicate types in an ontology, summarizing all relations between the same pair of concepts, *i* and *j*, with a single association probability, *p_ij_*. More general versions of our metrics account for multiple relation types that occur in more complex ontologies, but these involve numerous additional parameters that require estimation from real data and therefore are more challenging to implement. For this reason, we count relations represented in a test ontology *X* in two separate ways. |ℜ*_X_* | is the number of ordered pairs of concepts *with at least one relation* defined between them in ontology *X*, while |*R_X_*| is the total number of all relations in the ontology. For predicate-poor ontologies such as thesauri, these two ways of counting relations are equivalent. In predicate-rich ontologies with more than one relation between the same pair of concepts, |*R_X_*|>|ℜ*_X_* |.

Suppose an ontology has *N* concepts and each concept *i* has relations with other *M_i_* concepts (each denoted as concept *j* where *j* = 1, 2, …, M_i_). We practically infer the probability *p_ij_* that concept *i* is associated with concept *j* through simple concept co-occurrence in text. Namely, we estimate:
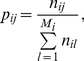
(1)


where *n_ij_* is the number of times concept *i* co-occurs with concept *j* in the same unit of text, such as a sentence or a paragraph (the medical abstract in our implementation). Note that when concept *i* is unobserved in the corpus, we encounter a singularity (zero divided by zero) when applying equation 1 directly and *p_ij_* violates the basic property of probability by not summing to 1. For this study we pragmatically postulate that if concept *i* is not observed in the corpus, then the value of *p_ij_* is set to 0. Datasets S1, S2, and S3 contain complete sets of non-zero estimates of synonym substitution probabilities for our three reference corpora.

The advantage of setting *p_ij_* to 0 when *i* is unobserved is that the ontology will be punished for concepts and relations unobserved in the corpus. One could alternately make *p_ij_* behave as a probability under all conditions (for all values of *n_ij_*) and still punish the ontology by making *p_ij_* very small for all unobserved *i* in the following manner: 
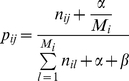
(2)


where parameter α and 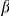 are small positive constants (0≤*α* 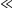 *β* 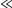 1). This would require us to further add a pseudo-concept 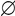, that relates to every concept *i* with the following probability:
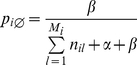
(3)


such that 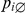 is close to 1 when *i* is not observed and every *p_ij_* is approximately 0.

One can imagine the use of more advanced natural language processing techniques than co-occurrence to assess the precise semantic relation in text, but we use the probability estimate from equation 1 in our preliminary evaluation of four medical ontologies against our corpus of clinical abstracts.

Consider further an arbitrary ontology that has multiple distinct relations defined for the same pair of concepts. In such a case, we could supplement *p_ij_* with an additional set of parameters, π*_k_*_|*ij*_. These new parameters reflect the relative frequency (importance) of textual mentions of the *k* ^th^ relation between concepts *i* and *j*, where
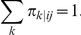



In the case of thesauri, in which the primary relation is synonymy, we are able to assess *p_ij_* more precisely than with medical ontologies. An English thesaurus has *N* headwords and each headword (denoted as *w_i_* where *i* = 1, 2, …, N) has a list of *M* synonyms (denoted as *w_i_*_,*j*_ where *j* = 1, 2, …, M*_i_*). We compute the probability of substituting word *w_i_* with its synonym *w_i_*_,*j*_ through probabilistic conditioning on all contexts observed in a corpus in the following way.
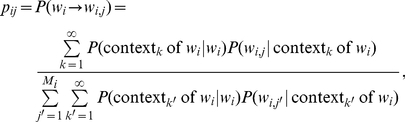
(4)


where 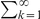
is a shorthand for “sum over all possible contexts of headword 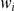
”.

Equation (4) is closely related to distributional similarity metrics explored by computational linguists, e.g. [67]. This notion, that words occurring in the same contexts tend to have similar meanings is called the Distributional Hypothesis and was introduced by Zellig Harris [68], then popularized by Firth—“a word is characterized by the company it keeps” [69]. Some researchers prefer to induce word relationships like *synonymy* and *antonymy* from co-occurrence rather than substitution in order to capture lexical as well as semantic similarity [70], [71]. In our analysis, however, we do not induce synonymy, but rather begin with established synonyms from a published Thesaurus. We then simply calculate their substitution frequencies based on shared context.

In our practical implementation, we defined the context of word *w_i_* within a sentence as a list of *k* words immediately preceding and following it, enriched with positional and part-of-speech (POS) information: 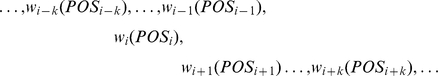



To increase the number of comparable four-token contexts for synonyms in our relatively small corpus, we only considered nouns, verbs, adjectives and adverbs in our analysis of context, disregarding tokens with other part-of-speech tags. That is, given a word *w_i_* in the text, we select the nouns, verbs, adjectives and adverbs around it within window size 2*k* = 4 (two before and two after *w_i_*), providing a four-word context for all words except those at a sentence boundary. Because many contexts constructed in this way are unique or very rare, we generalize them by ignoring word order and binning words that appear uniquely in the corpus into part-of-speech pseudo-words (e.g., *rare-noun*, *rare-verb*, *rare-adjective*, and *rare-adverb*). Equation 4 suffers the same limitation as equation 1 for headwords *i* that do not occur in corpus. One could extend it in the same manner as equation 1 by adding the pseudo-concept 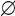 such that
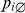
collects the vast majority of the probability mass for unobserved headwords.

### Information retrieval metrics

In information retrieval (IR), the goal is to identify documents from a large collection most relevant to a user's query. If the subset of relevant documents is known, we can calculate the quality of an information retrieval method with the metrics *precision*, *recall*, and the *F*-measure (harmonic mean of *precision* and *recall*). 
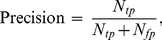
(5)

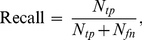
(6)

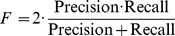
(7)


*True positives (tp)*, *false positives (fp)*, *false negatives (fn) and true negatives (tn)* are defined by the cross-tabulation between relevance and retrieval: *True positives* comprise documents that are both relevant to the query and retrieved by the method; *false positives* are documents retrieved but irrelevant; *false negatives* are relevant but not retrieved; and *true negatives* are irrelevant documents not retrieved.

More measures, such as accuracy and fallout, are introduced and computed in Text S1.

### Ontology evaluation metrics

For a given reference corpus *T*, we define the *complete ontology* 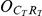, which incorporates all concepts and all relations encountered in corpus *T*. We also use the corpus to derive a frequency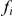 for each concept *i* in C*_T_*, the set of all concepts in *T*, and concept association probability
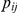
for each relation in R*_T_*, the set of all relations in *T*. In the special case of a thesaurus, we understand this probability to be the probability of appropriate substitutability, or “substitution probability” for short. It should be noted that our ability to estimate *f_i_* depends on mapping concepts from ontology to text. This is why we spent so much time and energy working with thesauri to facilitate the detection of concept synonyms in text.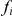 should be normalized in such a way that 
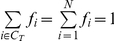
 (*N* is the total number of concepts in corpus *T*) and, by definition,
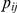
is normalized so that 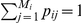 for any concept, *c_i_*, involved in at least one relationship. In our implementation, we approximate the complete ontology for our medical corpus with all “Disease or Syndrome” concepts in MetaMap, which includes the union of our four medical ontologies in addition to more than a hundred additional terminologies, such as the UK Clinical Terms, Logical Observation Identifiers Names and Codes (LOINC) that identifies medical laboratory observations, RxNorm that provides normalized names for clinical drugs, and the Online Mendelian Inheritance in Man (OMIM) database that catalogues diseases with a known genetic component. The complete ontology only retains those concepts and relations that appear in the corpus. For our thesauri, we approximated the complete ontology with the union of compared thesauri, excluding concepts and relations not found in the corpus.

Consider that we are trying to evaluate arbitrary ontology *X* with respect to reference corpus *T*. We define *C_X_* and *R_X_* as sets of concepts and relations within *X*, and | *C_X_* | and | *R_X_* | the cardinalities of those sets. To evaluate *X* with respect to *T*, we identify sets *C_X_*(*true-positives—tp*), *C*_X_(*false negatives—fn*), *R*_X_(*tp*), and *R_X_*(*fn*) such that *C*_X_(*tp*) = *C*_X_ ∩ C*_T_*, *R_X_*(*tp*)  =  *R_X_* ∩ R*_T_*, *C*_X_(*fn*)  =  *C_T_* — *C_X_*(*tp*), and *R_X_*(*fn*)  =  *R_T_* — *R_X_*(*tp*), where “—” represents set difference.

Then we derive the first ontology evaluation measure—*Breadth*—to capture the theoretical coverage of an ontology's concepts:
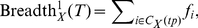
(8)


We derive a corollary version of breadth to capture the theoretical coverage of an ontology's concept and relations:
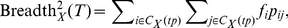
(9)


where *p_ij_* equals 0 if there is no relation between them in *X*. Both Breadth metrics are defined on the interval [0,1].

By modifying these measures to account for the number of concepts and relations, we develop measures of *Depth* to capture theoretical parsimony:
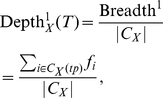
(10)

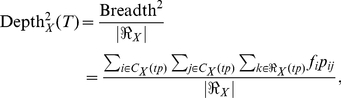
(11)


where |ℜ*_X_*| is the number of ordered pairs of concepts *with at least one relation* defined between them in ontology *X.* This normalization thus ignores the number of different relations that *X* may catalog between concepts *i* and *j*.

We can also compare an arbitrary ontology *X* with the fittest ontology of the same size *O*(*X*) by including the most representative *C_X_* concepts and *R_X_* relations from corpus *T* that maximize depth. In practice, to compute the fittest ontology of fixed size, we have to perform a numerical optimization over a set of concepts and relations where the size of the ontology being optimized is kept fixed, but the concepts and relations taken from the fittest ontology are added or removed to improve the breadth and depth of the optimized ontology. An estimate of the depth of the fittest ontology of fixed size, Depth*_O_*_(*X*)_(*T*), allows us to define and compute a Loss measure.
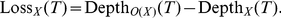
(12)


The above measure can be called the Loss of Depth or Depth Loss. In a similar way we can compute an ontology's Loss of Breadth. (In practice, our estimates of the fittest ontology of fixed size were constrained only by the total number of relations in the corresponding test ontology, so that the Depth Loss in Table 2 was computed using equation (19) in Text S1.)

Note that unlike Breadth, Depth is not naturally defined on the interval [0,1], but will rather tend to result in small positive numbers. Therefore, we define normalized versions of Depth and Depth Loss in the following way.
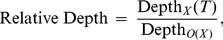
(13)

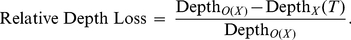
(14)


If we consider an arbitrary ontology with multiple types of relations between concepts *i* and *j*, we can further extend Breadth^2^ and Depth^2^ measures in the following way:
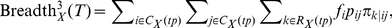
(15)

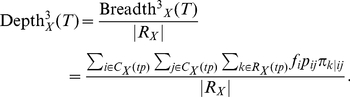
(16)


Note that this definition of Depth^3^ and Breadth^3^ involves three levels of ontology evaluation: parameter *f_i_* captures usage of the *i^th^* concept in the corpus; parameter *p_ij_* reflects the relative importance of all relations between concepts *i* and *j* with respect to all relations involving concept *i* in the corpus; and parameter π*_k_*_|*ij*_ measures the relative prevalence of the *k^th^* relation between concepts *i* and *j* in the corpus.

Precise implementation of this task would require capturing mentions of every concept *i –* relation *k* – concept *j* triplet in the text using natural language processing tools. The parameter estimates would then be computed by normalizing counts of captured relations and concepts in an appropriate way.

If, on average, there is only one type of relation per pair of concepts, use of metric Depth^3^ and Breadth^3^ would be overkill. For computational simplicity, we use only the first- and the second-level *Breadth* and *Depth* in our practical implementation.

## Supporting Information

Dataset S1 Probabilities – novels.
(8.37 MB DOC)

Dataset S2 Probabilities – medicine.
(11.39 MB DOC)

Dataset S3 Probabilities – news.
(25.12 MB DOC)

Text S1 Additional details on methods and data.
(0.17 MB PDF)
