# Supplementary material for: Benchmarking Ontologies: Bigger or Better?
Source: PLoS Comput Biol. 2011 Jan 13;7(1):e1001055. doi: 10.1371/journal.pcbi.1001055 (PMC3020923; doi:10.1371/journal.pcbi.1001055)
Supplement: Dataset S1 — Probabilities – novels. (8.37 MB DOC) [file pcbi.1001055.s001.doc]

word partofspeech synonym Prob

be v come 0.11960023406395100000000

be v stand 0.02070294528538030000000

be v pass 0.01540539040302570000000

be v transpire 0.00001784577381565380000

be v breathe 0.00172788702885037000000

be v endure 0.00248254923503888000000

be v last 0.00581026951402701000000

be v hold 0.00547462202874760000000

be v live 0.02906783922268530000000

be v represent 0.00069670339950583500000

be v appear 0.01079759185452010000000

be v follow 0.01191564198818270000000

be v remain 0.01573031736157330000000

be v hap 0.00001194968712676660000

be v look 0.08074770658392920000000

be v persist 0.00134649877690236000000

be v cost 0.00103582979484485000000

be v happen 0.02362317561769320000000

be v move 0.00643721595892605000000

be v signify 0.00117409648793364000000

be v prevail 0.00099662048818609900000

be v continue 0.00518327023896030000000

be v do 0.55751508807534800000000

be v dwell 0.00044775357350090500000

be v develop 0.00016410486542414800000

be v survive 0.00086284078759033400000

be v imply 0.00001659856039494650000

be v stay 0.00833772983357552000000

be v inhabit 0.00023344388193488700000

be v constitute 0.00002834210500638280000

be v abide 0.00025900940305866500000

be v seem 0.04727802268631830000000

be v equal 0.00036416634352174800000

be v befall 0.00038158964294294400000

be v reside 0.00075371545509840500000

be v escort 0.00025876372032698000000

be v rest 0.00289560048702800000000

be v occur 0.00392551092194433000000

be v attend 0.00187537674981686000000

be v act 0.00407251074404913000000

be v occupy 0.00076505414601811800000

be v obtain 0.00090810155316695600000

be v arise 0.00102601495018554000000

be v exist 0.00563188340971744000000

be v accompany 0.00194569785338050000000

be v embody 0.00001916768298718370000

be v subsist 0.00004771177385849330000

have v stand 0.01176324167050780000000

have v seduce 0.00006051225499333990000

have v bribe 0.00012667165139302800000

have v persuade 0.00246789890358578000000

have v direct 0.00120256370056857000000

have v know 0.18747557348473400000000

have v keep 0.01387950453422420000000

have v push 0.00085740447492593500000

have v demonstrate 0.00000917549953431611000

have v trick 0.00070345496429750500000

have v permit 0.00275260064135332000000

have v display 0.00037649302510538000000

have v own 0.00224617105210207000000

have v harbor 0.00070345496429750500000

have v support 0.00066645320274358200000

have v deliver 0.00145150431639472000000

have v become 0.00522143320246019000000

have v endure 0.00141784335891335000000

have v preserve 0.00040513061990043900000

have v deceive 0.00379797918781877000000

have v embrace 0.00105879975234573000000

have v make 0.03311621968399140000000

have v count 0.00283617314286667000000

have v hold 0.00375773960359748000000

have v dupe 0.00012139891691556700000

have v brook 0.00002677144581202620000

have v take 0.04082250900233420000000

have v maintain 0.00040131440538763700000

have v secure 0.00078894437951707400000

have v bid 0.00066356867441840400000

have v show 0.00673938838609819000000

have v command 0.00088667028208068500000

have v experience 0.00134807460984532000000

have v use 0.01668644183122250000000

have v encounter 0.00053814057671046100000

have v comprehend 0.00106514114633934000000

have v induce 0.00056667205457299300000

have v entertain 0.00148059543808615000000

have v receive 0.00865213404183412000000

have v retain 0.00133692752080827000000

have v bear 0.01404900577281740000000

have v acquire 0.00053673077202736700000

have v request 0.00035124953124941000000

have v undergo 0.00089506343233195600000

have v carry 0.00228218534913146000000

have v procure 0.00089183896312812300000

have v throw 0.00408679336722083000000

have v involve 0.00038122556871462200000

have v get 0.03707924906772980000000

have v cherish 0.00005210777513314850000

have v give 0.03337584574872820000000

have v outwit 0.00016551881512883600000

have v sustain 0.00010942632777961300000

have v tell 0.08734212559906040000000

have v exhibit 0.00014933226711816500000

have v allow 0.01034255997697550000000

have v ought 0.01531595279673490000000

have v must 0.10920336134405000000000

have v should 0.08628795541455230000000

have v drive 0.00281807359734583000000

have v boast 0.00165153372019409000000

have v express 0.00337798569026673000000

have v abide 0.00058754712341997300000

have v cultivate 0.00001545651428886460000

have v nurse 0.00007456123287788370000

have v wear 0.00237850643516651000000

have v find 0.02809205387180660000000

have v cheat 0.00021021497776065100000

have v suffer 0.00791831004263590000000

have v eat 0.00388412665536180000000

have v consume 0.00167928446574154000000

have v evince 0.00009704635972785380000

have v accept 0.00226810636294530000000

have v organize 0.00005411192033057730000

have v fool 0.00107478525908575000000

have v feel 0.03324595890966720000000

have v ask 0.04869339096227890000000

have v oblige 0.00527390676238890000000

have v manifest 0.00002599371692554580000

have v require 0.00479653610040534000000

have v stimulate 0.00006061286949848140000

have v cause 0.00095365891906121600000

have v include 0.00019063820170664300000

have v possess 0.00320967140850789000000

have v press 0.00165320100861702000000

have v drink 0.00429043347656824000000

have v number 0.00012304459538295100000

have v tolerate 0.00025112621850013200000

have v occupy 0.00126007216562334000000

have v force 0.00156504536065734000000

have v enjoin 0.00019405654187519400000

have v contain 0.00106276079440846000000

have v obtain 0.00093997397057284200000

have v order 0.00276401482493087000000

have v let 0.06597863000677130000000

have v enjoy 0.00382168108165642000000

have v embody 0.00002605388756657430000

have v encompass 0.00006161649322314470000

come v gain 0.00097848891291533300000

come v upbraid 0.00012632768600242200000

come v fetch 0.00129914210473906000000

come v confess 0.00189631571532774000000

come v suggest 0.00021549937661228200000

come v admonish 0.00000577581268140268000

come v comply 0.00011438516338520200000

come v stretch 0.00018129863448874400000

come v alienate 0.00000649151120931562000

come v manage 0.00127432532110572000000

come v spread 0.00181014115440277000000

come v own 0.00277519148302558000000

come v return 0.01540087027688200000000

come v score 0.00005004628753550640000

come v win 0.00159129188889228000000

come v slip 0.00093540500824145400000

come v transpire 0.00018367407298678700000

come v sound 0.00028174119496648700000

come v become 0.00526307836930857000000

come v breathe 0.00038678304973710400000

come v yield 0.00113794010462650000000

come v conclude 0.00120622343732744000000

come v affect 0.00023099237148601000000

come v fall 0.00888398087070877000000

come v acknowledge 0.00180962403639831000000

come v reprimand 0.00003047035873760390000

come v separate 0.00050998068729087100000

come v bend 0.00075401198596536000000

come v run 0.01713651341143540000000

come v reveal 0.00074221603932423100000

come v appear 0.00967563569038405000000

come v secure 0.00024107399231410700000

come v follow 0.00572017357119476000000

come v show 0.01059205921003700000000

come v divide 0.00057796787251607100000

come v unmask 0.00007195410015145110000

come v meet 0.01212553934988130000000

come v extend 0.00013210967720648700000

come v grant 0.00078405559248529600000

come v enter 0.01580908588515670000000

come v furnish 0.00046453174143797100000

come v attack 0.00082590675545463500000

come v encounter 0.00204750106083507000000

come v open 0.00495591574154494000000

come v derive 0.00001078012691799710000

come v submit 0.00039513542924080800000

come v look 0.09232550083417010000000

come v awaken 0.00173472409159155000000

come v penetrate 0.00020119863777017300000

come v plunge 0.00000640792951992535000

come v supply 0.00018907692539812200000

come v happen 0.02508949974517370000000

come v soften 0.00020831275910415800000

come v revive 0.00026162847510533500000

come v acquire 0.00006034612017888820000

come v expose 0.00024977032074108100000

come v move 0.00820394842961697000000

come v discharge 0.00044268016781943700000

come v betide 0.00015716290296237800000

come v attain 0.00177598265350599000000

come v retort 0.00018221410632729700000

come v prevail 0.00011210974856797500000

come v procure 0.00257831677449345000000

come v tumble 0.00030855471100150800000

come v rejoin 0.00077992514674709100000

come v oppose 0.00006879380898146010000

come v chance 0.00014481823584750400000

come v earn 0.00035749221890179900000

come v issue 0.00098541615797540700000

come v do 0.24465836234686500000000

come v get 0.09816528146755130000000

come v bite 0.00131798327265754000000

come v reach 0.00675078683327077000000

come v protest 0.00084678753133495000000

come v develop 0.00094641395074177300000

come v allow 0.00434281322594453000000

come v reject 0.00008548811488782560000

come v ensue 0.00060823783257174300000

come v catch 0.00466396766669361000000

come v mend 0.00035543605132241900000

come v stumble 0.00030475022499450500000

come v annoy 0.00030514766193044800000

come v bother 0.00101595155298154000000

come v contract 0.00006843087211714580000

come v scold 0.00026005967116692400000

come v rally 0.00008770751846634260000

come v arrive 0.01299312625254120000000

come v disclose 0.00007058681236149790000

come v result 0.00059721903125703700000

come v amount 0.00078541613886666000000

come v close 0.00300920826148882000000

come v agree 0.00196915873610056000000

come v offer 0.00133682325272085000000

come v find 0.03161964415214040000000

come v seem 0.01536800049361340000000

come v approach 0.00455205134709959000000

come v settle 0.00486339690528264000000

come v expand 0.00002638317005553200000

come v befall 0.00255017617407917000000

come v fix 0.00159971799245854000000

come v provide 0.00235101884799419000000

come v ejaculate 0.00003851114784891640000

come v originate 0.00007901559636870190000

come v produce 0.00038821251860980900000

come v finish 0.00524798392502579000000

come v go 0.23786360882471500000000

come v present 0.00359580135147615000000

come v discover 0.00139618872400090000000

come v relent 0.00002611745034651790000

come v disapprove 0.00037326189453564800000

come v irritate 0.00011514836712143800000

come v sing 0.00227931210251087000000

come v admit 0.00261352680105749000000

come v sicken 0.00006491511209315620000

come v cover 0.00006964006503611910000

come v proceed 0.00244201496814778000000

come v occur 0.00421473006701692000000

come v uncover 0.00004593992548131050000

come v near 0.00006847839087860610000

come v end 0.00350396108279066000000

come v number 0.00007530575337597860000

come v rush 0.00096704330662252300000

come v advance 0.00144683618311999000000

come v acquiesce 0.00003467306061841330000

come v succeed 0.00418627898897960000000

come v degenerate 0.00013011873579561700000

come v beset 0.00149304757814259000000

come v obtain 0.00120404220575427000000

come v arise 0.00126797187288502000000

come v visit 0.00188687487644922000000

come v propose 0.00267844457042806000000

come v descend 0.00161362617530219000000

come v concede 0.00014511744818598600000

come v recover 0.00189718453971371000000

always r ever 0.77091343694768200000000

always r continually 0.08103996552872690000000

always r constantly 0.00150843037864604000000

always r forever 0.14491826249135700000000

always r regularly 0.00161990465358808000000

suspicion n fancy 0.00646168770943948000000

suspicion n watchfulness 0.00086682869496679400000

suspicion n conclusion 0.00086682869496679400000

suspicion n unbelief 0.00028168910171166200000

suspicion n notion 0.01538331090173120000000

suspicion n thought 0.08166991101027380000000

suspicion n question 0.00964271362769198000000

suspicion n hint 0.39850448877186900000000

suspicion n feeling 0.01417378997068670000000

suspicion n idea 0.19713855551750800000000

suspicion n theory 0.00288530122753233000000

suspicion n suggestion 0.00114851779667846000000

suspicion n doubt 0.16454743027495700000000

suspicion n conjecture 0.00114851779667846000000

suspicion n speculation 0.00086682869496679400000

suspicion n trace 0.00252463857409079000000

suspicion n misgiving 0.01074584920227860000000

suspicion n supposition 0.00288530122753233000000

suspicion n guess 0.01044239871073510000000

suspicion n bit 0.00252463857409079000000

suspicion n reservation 0.00028168910171166200000

suspicion n impression 0.05785915926825440000000

suspicion n taste 0.01714992554964790000000

reliable a honest 0.01568921707396100000000

reliable a safe 0.00784460853698046000000

reliable a regular 0.02353382561094150000000

reliable a respectable 0.02353382561094150000000

reliable a sure 0.10197991098074700000000

reliable a certain 0.05491225975886360000000

reliable a true 0.53716809631815000000000

reliable a decent 0.00784460853698046000000

reliable a sound 0.00784460853698046000000

reliable a secure 0.00784460853698046000000

reliable a responsible 0.03137843414792200000000

reliable a strong 0.03922304268490250000000

reliable a good 0.07844608536980510000000

reliable a real 0.02353382561094150000000

reliable a clear 0.01568921707396100000000

reliable a candid 0.00784460853698046000000

reliable a competent 0.00784460853698046000000

reliable a trustworthy 0.00784460853698046000000

steal v keep 0.02896186719579330000000

steal v withdraw 0.00582625806310446000000

steal v pass 0.05586149724442260000000

steal v swindle 0.02121924759074260000000

steal v take 0.25662793942073200000000

steal v purloin 0.28645984247502500000000

steal v snatch 0.00270450476417334000000

steal v rob 0.29399171157121100000000

steal v impress 0.00017870233466938500000

steal v borrow 0.00096991316885996000000

steal v drift 0.00597577752360372000000

steal v divert 0.00017207379034390900000

steal v cheat 0.00210606749254223000000

steal v fly 0.00044743017789493100000

steal v seize 0.00028689017774163700000

steal v remove 0.01641071091192660000000

steal v lift 0.01909732283166830000000

steal v press 0.00184051793975777000000

steal v rustle 0.00017870233466938500000

steal v liberate 0.00068302299111832300000

provoking a annoying 1.00000000000000000000000

prize v like 0.01570937653411880000000

prize v love 0.98429062346588100000000

honest a reliable 0.00011428602169841200000

honest a explicit 0.00301996277178465000000

honest a plain 0.01612900419166690000000

honest a conceivable 0.00005896719498603180000

honest a worthy 0.00659806310184826000000

honest a impartial 0.00133984263818615000000

honest a proper 0.00320616471532436000000

honest a credible 0.00011793438997206400000

honest a respectable 0.00360798997540563000000

honest a moral 0.00093492704974168400000

honest a legal 0.00219951045806281000000

honest a humble 0.00054002321173048900000

honest a tactless 0.00021437482210978300000

honest a honorable 0.03271626336100040000000

honest a full 0.00224006987222839000000

honest a faithful 0.00021437482210978300000

honest a bold 0.00270223199914484000000

honest a reasonable 0.00218656173120754000000

honest a straight 0.00011428602169841200000

honest a direct 0.00100665425726155000000

honest a white 0.01196357992273000000000

honest a modest 0.00256528493833761000000

honest a true 0.08898596904003790000000

honest a exact 0.00112094027895996000000

honest a right 0.07028007640252940000000

honest a sincere 0.00296707226989216000000

honest a decent 0.00011428602169841200000

honest a correct 0.00123522630065837000000

honest a sound 0.00231379647976122000000

honest a open 0.19419776867028200000000

honest a naked 0.00005896719498603180000

honest a responsible 0.00057507847676571500000

honest a free 0.01215711788784230000000

honest a good 0.47600343374082000000000

honest a virtuous 0.00150031306333780000000

honest a natural 0.01865569305672680000000

honest a real 0.00122835426689666000000

honest a candid 0.00112094027895996000000

honest a conscientious 0.00005896719498603180000

honest a fair 0.01533209646624720000000

honest a loyal 0.00005896719498603180000

honest a simple 0.01813029222369220000000

honest a trustworthy 0.00011428602169841200000

pique v interest 1.00000000000000000000000

perturb v disturb 0.11111111111111100000000

perturb v excite 0.05555555555555550000000

perturb v agitate 0.05555555555555550000000

perturb v worry 0.05555555555555550000000

perturb v rattle 0.05555555555555550000000

perturb v trouble 0.05555555555555550000000

perturb v alarm 0.05555555555555550000000

perturb v vex 0.22222222222222300000000

perturb v shake 0.05555555555555550000000

perturb v provoke 0.11111111111111100000000

perturb v annoy 0.05555555555555550000000

perturb v perplex 0.05555555555555550000000

perturb v madden 0.05555555555555550000000

pawn n fool 1.00000000000000000000000

participate v use 1.00000000000000000000000

over a complete 0.01378937019662930000000

over a past 0.02651019141123190000000

over a better 0.78255105715757100000000

over a outside 0.00323663997857508000000

over a dead 0.05856805540566730000000

over a high 0.07598539846380510000000

over a external 0.00323663997857508000000

over a superfluous 0.00323663997857508000000

over a heavy 0.02641272747222000000000

over a extinct 0.00323663997857508000000

over a warm 0.00323663997857508000000

arrival n meeting 0.06075488587189590000000

arrival n baby 0.13907262481598800000000

arrival n entrance 0.50684245488493300000000

arrival n dawn 0.09674617378503500000000

arrival n return 0.11711378931872700000000

arrival n comer 0.07947007132342160000000

nuisance n trial 0.07142857142857120000000

nuisance n burden 0.07142857142857120000000

nuisance n trouble 0.21428571428571400000000

nuisance n pain 0.35714285714285800000000

nuisance n difficulty 0.21428571428571400000000

nuisance n worry 0.07142857142857120000000

motto n thought 0.02203060945272080000000

motto n legend 0.01101530472636040000000

motto n word 0.96695408582091900000000

maxim n rule 0.20000000000000000000000

maxim n word 0.80000000000000000000000

liveliness n life 1.00000000000000000000000

irrational a strange 1.00000000000000000000000

influential a prominent 0.25000000000000000000000

influential a important 0.75000000000000000000000

implicitly r certainly 1.00000000000000000000000

gain v gather 0.00157995610445320000000

gain v win 0.02271369825380070000000

gain v realize 0.00051671365424803100000

gain v clear 0.00315991220890641000000

gain v make 0.36199663012132000000000

gain v collect 0.00157995610445320000000

gain v secure 0.00157995610445320000000

gain v improve 0.03844714884692820000000

gain v complete 0.00157995610445320000000

gain v accomplish 0.00473986831335962000000

gain v acquire 0.00157995610445320000000

gain v attain 0.00315991220890641000000

gain v hit 0.00315991220890641000000

gain v carry 0.01769135059065370000000

gain v procure 0.00157995610445320000000

gain v get 0.25682121547438200000000

gain v reach 0.01714482179469650000000

gain v delay 0.00077507048137204500000

gain v capture 0.00499822514048363000000

gain v catch 0.00183831293157722000000

gain v effect 0.00315991220890641000000

gain v achieve 0.09744611620701100000000

gain v approach 0.00613276808783035000000

gain v produce 0.00157995610445320000000

gain v merit 0.00025835682712401400000

gain v enlarge 0.00025835682712401400000

gain v advance 0.14014533438530000000000

gain v succeed 0.00437667049599100000000

guise n dress 1.00000000000000000000000

teach v initiate 0.00011829731273926500000

teach v spread 0.00148071767956912000000

teach v announce 0.00056347768635963600000

teach v reprimand 0.00803818168908951000000

teach v indicate 0.00012285430529176100000

teach v question 0.00566996698735207000000

teach v warn 0.00073712583175056600000

teach v show 0.01277794302257250000000

teach v accustom 0.00068177499909890100000

teach v advise 0.00581616500826340000000

teach v fit 0.00116484082054410000000

teach v guide 0.00011829731273926500000

teach v impart 0.00246169314228366000000

teach v tell 0.28535730372137100000000

teach v develop 0.15754836110615400000000

teach v repeat 0.01934781225279180000000

teach v learn 0.04044125307712940000000

teach v practice 0.00080918629694315800000

teach v preach 0.00012285430529176100000

teach v enlighten 0.00109059608112809000000

teach v explain 0.41337914141400300000000

teach v publish 0.00056347768635963600000

teach v form 0.00056347768635963600000

teach v prepare 0.00334450682656664000000

teach v sharpen 0.00056347768635963600000

teach v school 0.00056347768635963600000

teach v describe 0.02431930854808860000000

teach v inform 0.01075371214787070000000

teach v rehearse 0.00148071767956912000000

funereal a melancholy 1.00000000000000000000000

fork v turn 1.00000000000000000000000

otherwise r then 0.91814647145012000000000

otherwise r differently 0.08185352854987950000000

disagree v hurt 1.00000000000000000000000

future n fate 0.01019276021394530000000

future n tomorrow 0.63304711466728700000000

future n destiny 0.02360024458578740000000

future n eternity 0.08734911243106160000000

future n hereafter 0.05877916144824790000000

future n fortune 0.18703160665367000000000

din n noise 1.00000000000000000000000

decree v find 1.00000000000000000000000

crop v cut 1.00000000000000000000000

stand v be 0.65224173811155200000000

stand v digest 0.00011988667794377400000

stand v depend 0.00048976491392211200000

stand v permit 0.00062559254247576000000

stand v support 0.00010047967311592100000

stand v endure 0.01556967643019380000000

stand v apply 0.00014695769255583400000

stand v insist 0.00028545784817907200000

stand v await 0.00299175201282454000000

stand v last 0.00276108150659794000000

stand v hold 0.01085197949321600000000

stand v brook 0.00002449362273463190000

stand v take 0.04026178876082320000000

stand v question 0.00027797841141833800000

stand v set 0.01352128002204080000000

stand v meet 0.02189504167662690000000

stand v extend 0.00001017129644438760000

stand v wait 0.03705257571760370000000

stand v remain 0.03249000400909880000000

stand v encounter 0.00001045836318175320000

stand v resist 0.00186295358547443000000

stand v submit 0.00064165464263014000000

stand v handle 0.00002449362273463190000

stand v swallow 0.00109171694766481000000

stand v defend 0.00030124775578035000000

stand v rise 0.00152126098960081000000

stand v brave 0.00004371203270872190000

stand v stop 0.01574368237355660000000

stand v bear 0.04367498516480070000000

stand v persist 0.00021112001081044300000

stand v erect 0.00232922688576475000000

stand v attain 0.00002449362273463190000

stand v prevail 0.00558595832755715000000

stand v halt 0.00136380133158497000000

stand v continue 0.00196501485211041000000

stand v promote 0.00009316907543059020000

stand v allow 0.00156148974443865000000

stand v repeat 0.00119749221219691000000

stand v survive 0.00014341592570825800000

stand v favor 0.00002449362273463190000

stand v stay 0.00912638837026079000000

stand v dispute 0.00111801767727175000000

stand v confront 0.00004006041326868130000

stand v place 0.00062485486447822700000

stand v abide 0.00034753641579705700000

stand v project 0.00002374078346287260000

stand v wear 0.00058464530265179600000

stand v suffer 0.00303427120108041000000

stand v affirm 0.00007268018514273380000

stand v defy 0.00004274058632924520000

stand v fill 0.00815737974839884000000

stand v accept 0.00081353198889899300000

stand v forgive 0.00480865428595132000000

stand v rest 0.00248416187277156000000

stand v uphold 0.00009522936330015580000

stand v belong 0.00009821658129111750000

stand v occupy 0.00171533853775354000000

stand v arrange 0.00039311365964833700000

stand v withstand 0.00006662434159859380000

stand v countenance 0.00008132444049942660000

stand v pause 0.01030355255397620000000

stand v put 0.02754250295557160000000

stand v obtain 0.00033047352669641400000

stand v exist 0.01252082641803970000000

stand v face 0.00327743020611051000000

stand v illustrate 0.00116316221117785000000

ashamed a chaste 0.00045878553706044800000

ashamed a humiliated 0.00045180682748262700000

ashamed a innocent 0.00988913967985667000000

ashamed a proper 0.00028682300385610300000

ashamed a uneasy 0.07102231856453710000000

ashamed a shamefaced 0.00045180682748262700000

ashamed a self-conscious 0.00090324048518733200000

ashamed a guilty 0.04243847341486170000000

ashamed a disgraced 0.00090324048518733200000

ashamed a uncomfortable 0.05726806372247820000000

ashamed a modest 0.00095873294554941800000

ashamed a distressed 0.00415767065202163000000

ashamed a decent 0.00194339239022191000000

ashamed a confused 0.00135542048244788000000

ashamed a afraid 0.52421545140473700000000

ashamed a naked 0.00043496821877201400000

ashamed a shy 0.00135504731266996000000

ashamed a embarrassed 0.03174774505371290000000

ashamed a red 0.00647380164316326000000

ashamed a bewildered 0.00497131425200352000000

ashamed a reserved 0.00565932058580512000000

ashamed a sorry 0.22790794908798600000000

ashamed a abashed 0.00208030381006917000000

ashamed a degraded 0.00028682300385610300000

ashamed a penitent 0.00133820870395935000000

ashamed a disconcerted 0.00104015190503458000000

crib n hut 1.00000000000000000000000

cot n bed 1.00000000000000000000000

convincing a powerful 0.07291666666666670000000

convincing a reasonable 0.14583333333333300000000

convincing a probable 0.07291666666666670000000

convincing a possible 0.07291666666666670000000

convincing a decisive 0.56250000000000000000000

convincing a likely 0.07291666666666670000000

charge n promise 0.00348738723546027000000

charge n expense 0.00147623050633039000000

charge n rule 0.00029602047110726200000

charge n command 0.17735855083198000000000

charge n price 0.01700698432635420000000

charge n credit 0.00029602047110726200000

charge n request 0.00147623050633039000000

charge n rate 0.00847028259410014000000

charge n attack 0.00029602047110726200000

charge n business 0.02584763337053870000000

charge n bearing 0.03879718299449550000000

charge n complaint 0.00348738723546027000000

charge n entreaty 0.00147623050633039000000

charge n obligation 0.00147623050633039000000

charge n worth 0.00147623050633039000000

charge n law 0.04431500457593490000000

charge n rent 0.00059204094221452300000

charge n duty 0.00147623050633039000000

charge n control 0.02035262058727630000000

charge n will 0.00697477447092054000000

charge n care 0.61059376093717900000000

charge n word 0.03297094544678150000000

commentary n detail 0.00161808447352765000000

commentary n article 0.00161808447352765000000

commentary n account 0.00161808447352765000000

commentary n opinion 0.00970850684116597000000

commentary n paper 0.00323616894705532000000

commentary n history 0.00647233789411064000000

commentary n relation 0.87336109458655600000000

commentary n remark 0.10236763831052900000000

clutch n hold 1.00000000000000000000000

climax n height 0.16352440322335400000000

climax n point 0.33019106989002100000000

climax n tip 0.00314226344331254000000

climax n limit 0.16352440322335400000000

climax n extremity 0.16352440322335400000000

climax n perfection 0.00314226344331254000000

climax n pitch 0.16352440322335400000000

climax n head 0.00628452688662509000000

climax n top 0.00314226344331254000000

horror n anxiety 0.00406747975943227000000

horror n nervousness 0.00020044298210126400000

horror n alarm 0.01902144946322740000000

horror n abomination 0.00499404850771993000000

horror n dread 0.00020044298210126400000

horror n hatred 0.01637289320885400000000

horror n anguish 0.02035276571192920000000

horror n fright 0.73295985264969500000000

horror n fear 0.04354213335737940000000

horror n aversion 0.01902144946322740000000

horror n consternation 0.01902144946322740000000

horror n panic 0.00437588666536920000000

horror n terror 0.11586970578573600000000

attainment n blow 0.14285714285714200000000

attainment n success 0.42857142857142900000000

attainment n deed 0.28571428571428600000000

attainment n accomplishment 0.14285714285714200000000

gather v gain 0.00079278822285424900000

gather v reckon 0.00039639411142712400000

gather v associate 0.00039639411142712400000

gather v invite 0.00019819705571356200000

gather v believe 0.00458179889230005000000

gather v conclude 0.00019819705571356200000

gather v fold 0.27707948388756000000000

gather v make 0.01309363827873990000000

gather v build 0.00079278822285424900000

gather v take 0.45624924616712600000000

gather v collect 0.02617439867017230000000

gather v meet 0.00930462760929407000000

gather v think 0.04893533809584410000000

gather v extend 0.00019819705571356200000

gather v suppose 0.00277475877998987000000

gather v summon 0.00039639411142712400000

gather v draw 0.00893804786734065000000

gather v expect 0.01476463682153220000000

gather v select 0.00893804786734065000000

gather v capture 0.00059459116714068600000

gather v learn 0.00019819705571356200000

gather v close 0.00180704011231017000000

gather v save 0.00503380073133731000000

gather v find 0.02219352107373480000000

gather v hear 0.01893441933798560000000

gather v imagine 0.00039639411142712400000

gather v choose 0.00019819705571356200000

gather v discover 0.00684084084364749000000

gather v pull 0.00019819705571356200000

gather v order 0.00039639411142712400000

gather v increase 0.06394141935866770000000

gather v judge 0.00059459116714068600000

gather v surmise 0.00446902393367032000000

appealing a charming 1.00000000000000000000000

affront v disturb 0.01753392433185950000000

affront v hurt 0.08666713859423670000000

affront v trespass 0.00350678486637187000000

affront v meet 0.05076452266341400000000

affront v wound 0.01052035459911570000000

affront v resist 0.00350678486637187000000

affront v offend 0.03239583106445080000000

affront v oppose 0.00701356973274377000000

affront v confuse 0.00350678486637187000000

affront v vex 0.00701356973274377000000

affront v scorn 0.00350678486637187000000

affront v provoke 0.00701356973274377000000

affront v contradict 0.00350678486637187000000

affront v annoy 0.66719086929058400000000

affront v upset 0.02538226133170700000000

affront v insult 0.00350678486637187000000

affront v dispute 0.00701356973274377000000

affront v tease 0.00350678486637187000000

affront v embarrass 0.00350678486637187000000

affront v disconcert 0.00350678486637187000000

affront v challenge 0.02538226133170700000000

affront v cross 0.01052035459911570000000

affront v slight 0.01052035459911570000000

affront v discompose 0.00350678486637187000000

aberration n lie 1.00000000000000000000000

consideration n fancy 0.00170068587233504000000

consideration n aspect 0.02269461939307100000000

consideration n importance 0.00395266740141167000000

consideration n evidence 0.00427340429479437000000

consideration n observation 0.00205730260787046000000

consideration n motive 0.00016881167377458700000

consideration n judgment 0.00011254111584972400000

consideration n notion 0.00684344645117975000000

consideration n thought 0.07900713696863320000000

consideration n weight 0.00411767753156783000000

consideration n value 0.00005627055792486190000

consideration n understanding 0.00016881167377458700000

consideration n matter 0.11717380978012100000000

consideration n patience 0.00020922502729686500000

consideration n concern 0.00307723652787403000000

consideration n incident 0.00115370173678461000000

consideration n idea 0.01304420803099440000000

consideration n particulars 0.00024752141124003800000

consideration n proposal 0.00024752141124003800000

consideration n point 0.00322285910514216000000

consideration n state 0.00862570550989634000000

consideration n problem 0.02941975541098580000000

consideration n tip 0.00005627055792486190000

consideration n perplexity 0.00011326073309080900000

consideration n generosity 0.00067935249820231300000

consideration n attention 0.00206314721755191000000

consideration n speculation 0.00133990372800419000000

consideration n caution 0.04538923878614200000000

consideration n circumstance 0.01841958742427750000000

consideration n honor 0.01613060136824700000000

consideration n occasion 0.00655206511971428000000

consideration n prudence 0.00005627055792486190000

consideration n consultation 0.00432278464629924000000

consideration n care 0.00867614168767439000000

consideration n cause 0.03839859587748040000000

consideration n benevolence 0.00067935249820231300000

consideration n emergency 0.18155695514456800000000

consideration n condition 0.00437905520422410000000

consideration n difficulty 0.00016881167377458700000

consideration n plan 0.00277406173934474000000

consideration n magnitude 0.00521216139171009000000

consideration n notice 0.00580074498198527000000

consideration n consequence 0.19596270655392700000000

consideration n moment 0.01727380837878620000000

consideration n puzzle 0.00005627055792486190000

consideration n respect 0.01590082205000980000000

consideration n situation 0.12542613820784100000000

consideration n kindness 0.00067618977707474100000

consideration n study 0.00036078214433084700000

wintry a dark 1.00000000000000000000000

wag v shake 1.00000000000000000000000

unsuccessful a unhappy 0.02577733199598790000000

unsuccessful a vain 0.12076228686058200000000

unsuccessful a unfortunate 0.13365095285857600000000

unsuccessful a broken 0.02577733199598790000000

unsuccessful a poor 0.03866599799398190000000

unsuccessful a useless 0.54749247743229700000000

unsuccessful a disconcerted 0.10787362086258800000000

unique a peculiar 0.01742809213392210000000

unique a odd 0.00871404606696101000000

unique a new 0.01742809213392210000000

unique a original 0.00871404606696101000000

unique a strange 0.06099832246872740000000

unique a best 0.39482231542318200000000

unique a different 0.26773265802056500000000

unique a particular 0.21544838161879800000000

unique a single 0.00871404606696101000000

unhealthy a offensive 1.00000000000000000000000

twisted a distorted 1.00000000000000000000000

thrashing n beating 1.00000000000000000000000

excuse v spare 0.05550599937490190000000

excuse v permit 0.17774870763880200000000

excuse v approve 0.00412154250913019000000

excuse v dismiss 0.00018621237852797400000

excuse v defend 0.00357766375463143000000

excuse v discharge 0.00421440144393629000000

excuse v forget 0.10674079775757400000000

excuse v justify 0.20981100193818900000000

excuse v indulge 0.00032230927131882400000

excuse v overlook 0.00140547317362904000000

excuse v allow 0.01012522647049050000000

excuse v repent 0.02045062558526900000000

excuse v pardon 0.12056502977136800000000

excuse v explain 0.05783347887921600000000

excuse v apologize 0.00111564786928286000000

excuse v forgive 0.22451484717164600000000

excuse v apologise 0.00157482263355901000000

excuse v appease 0.00018621237852797400000

stale a weak 0.00260901975400669000000

stale a close 0.00521803950801341000000

stale a dry 0.00260901975400669000000

stale a weary 0.00521803950801341000000

stale a useless 0.02348117778606040000000

stale a flat 0.00260901975400669000000

stale a tired 0.00782705926202012000000

stale a dead 0.11479686917629500000000

stale a old 0.00521803950801341000000

stale a cold 0.01565411852404030000000

stale a commonplace 0.00260901975400669000000

stale a hard 0.00260901975400669000000

stale a simple 0.80954155795751000000000

soot n crock 1.00000000000000000000000

miserable a funereal 0.00150454897877569000000

miserable a unhappy 0.08162221248423450000000

miserable a low-spirited 0.00091021490946196400000

miserable a ill 0.00818300950015227000000

miserable a sick 0.01247833769732330000000

miserable a discontented 0.00107598048179110000000

miserable a despondent 0.00015996706366919700000

miserable a cheerless 0.00080016576674042100000

miserable a melancholy 0.03232451241813260000000

miserable a gloomy 0.02047892262463400000000

miserable a stingy 0.00152628756426815000000

miserable a inconsolable 0.00015996706366919700000

miserable a heart-breaking 0.00011004914272154400000

miserable a narrow-minded 0.00046967401982944700000

miserable a unfortunate 0.03087029253927470000000

miserable a broken 0.00038006534911228400000

miserable a poor 0.04013505152067190000000

miserable a execrable 0.00161459812149723000000

miserable a fatal 0.00333338973100484000000

miserable a wretched 0.00230996138010081000000

miserable a uncomfortable 0.00523094052111952000000

miserable a dismal 0.00138123906503892000000

miserable a small 0.01294993093396870000000

miserable a dreary 0.00638352133572159000000

miserable a venal 0.00015996706366919700000

miserable a distressed 0.00342481463959492000000

miserable a black 0.00388088301807526000000

miserable a wounded 0.00130862491028647000000

miserable a mean 0.02966111037432530000000

miserable a grim 0.00072612179752773400000

miserable a mournful 0.00083617094024927700000

miserable a low 0.00248699718787609000000

miserable a desolate 0.00022009828544308800000

miserable a sorrowful 0.00015996706366919700000

miserable a inferior 0.00187375307667199000000

miserable a shameful 0.00094622008297082100000

miserable a unlucky 0.01141793518449640000000

miserable a troublesome 0.00450575194064255000000

miserable a tragic 0.00022775725400324700000

miserable a pitiful 0.00789052353313472000000

miserable a vile 0.00080016576674042100000

miserable a distressing 0.00329459897055595000000

miserable a forlorn 0.00150454897877569000000

miserable a depressed 0.00176029859715004000000

miserable a disgraceful 0.00385661781236107000000

miserable a touching 0.00223067077630342000000

miserable a sorry 0.09540797735615240000000

miserable a contemptible 0.00107598048179110000000

miserable a pained 0.00015996706366919700000

miserable a mercenary 0.00080016576674042100000

miserable a hard 0.01796600195332140000000

miserable a crushed 0.00011004914272154400000

miserable a friendless 0.00373761641043223000000

miserable a downcast 0.11835785299702100000000

miserable a anxious 0.30786034189722900000000

miserable a strained 0.01543798082569840000000

miserable a blue 0.00653509617774961000000

miserable a worried 0.01155997058465140000000

miserable a deplorable 0.04115625342850950000000

miserable a pitiable 0.00887683897477656000000

miserable a destitute 0.00355073558991062000000

miserable a sad 0.00739558396388305000000

miserable a grieved 0.00160033153348084000000

miserable a ruinous 0.00072612179752773400000

miserable a base 0.00187614624853152000000

miserable a difficult 0.00566331125395202000000

miserable a despicable 0.00057923908481413100000

shamefully r badly 1.00000000000000000000000

fancy n hallucination 0.00042636668751682300000

fancy n fashion 0.00013426804981570400000

fancy n sentiment 0.00042636668751682300000

fancy n discrimination 0.29803031457426000000000

fancy n imagination 0.45909394580250300000000

fancy n ghost 0.00056063473733252800000

fancy n disposition 0.00196267488769460000000

fancy n style 0.00013426804981570400000

fancy n notion 0.00270310688305046000000

fancy n set 0.00013426804981570400000

fancy n humor 0.00334101356946914000000

fancy n mood 0.00013426804981570400000

fancy n thought 0.00155280837183635000000

fancy n belief 0.04416950422261760000000

fancy n feeling 0.01169070841691260000000

fancy n understanding 0.00057350285036740200000

fancy n idea 0.04510192125950640000000

fancy n vision 0.00042636668751682300000

fancy n look 0.01133122322563540000000

fancy n rage 0.00281160674126660000000

fancy n attraction 0.00042636668751682300000

fancy n longing 0.00170546675006730000000

fancy n peculiarity 0.00042636668751682300000

fancy n opinion 0.00590160771321767000000

fancy n sense 0.00013426804981570400000

fancy n eye 0.00712849600579829000000

fancy n view 0.00470017574977670000000

fancy n supposition 0.00602352447057451000000

fancy n liking 0.00013426804981570400000

fancy n delusion 0.00204989099392056000000

fancy n whim 0.02449462513210060000000

fancy n partiality 0.00013944025947017700000

fancy n phantom 0.00013944025947017700000

fancy n wish 0.00642172910836380000000

fancy n fiction 0.00013426804981570400000

fancy n picture 0.00198687096273358000000

fancy n desire 0.04257575922489430000000

fancy n passion 0.00338510959163400000000

fancy n impression 0.00668184820015368000000

fancy n taste 0.00067134024907852400000

scope n room 1.00000000000000000000000

sarcastically r sneeringly 0.50000000000000000000000

sarcastically r scornfully 0.50000000000000000000000

rid v detach 0.50000000000000000000000

rid v kill 0.25000000000000000000000

rid v unmarry 0.25000000000000000000000

revel v like 1.00000000000000000000000

noble a reliable 0.00005710621432878020000

noble a honest 0.00080919545360269000000

noble a gentle 0.38027028121534900000000

noble a excellent 0.01092607568562410000000

noble a extraordinary 0.00179869628321345000000

noble a wonderful 0.01155256273978610000000

noble a worthy 0.01210156688760930000000

noble a grand 0.00390160820694461000000

noble a splendid 0.00255291473246364000000

noble a useful 0.01092607568562410000000

noble a superior 0.00021423677814949200000

noble a lovely 0.00080919545360269000000

noble a generous 0.00048074624679563700000

noble a remarkable 0.00202936938538266000000

noble a respectable 0.00017131864298634100000

noble a moral 0.00040933291842341200000

noble a faithful 0.00021423677814949200000

noble a lofty 0.00096149249359127500000

noble a straight 0.00005710621432878020000

noble a true 0.03317412307620180000000

noble a exquisite 0.00005710621432878020000

noble a best 0.00442515355402559000000

noble a striking 0.00187223882156195000000

noble a decent 0.00005710621432878020000

noble a valuable 0.00048074624679563700000

noble a responsible 0.00063775777573853300000

noble a high 0.00225626771782173000000

noble a fine 0.08643824773654640000000

noble a good 0.39338029997157700000000

noble a upright 0.00040933291842341200000

noble a prime 0.00005710621432878020000

noble a candid 0.00005710621432878020000

noble a rich 0.00449975484647607000000

noble a great 0.02773723855056090000000

noble a fair 0.00109472652524659000000

noble a big 0.00096149249359127500000

noble a trustworthy 0.00005710621432878020000

noble a proud 0.00210397067783315000000

purify v free 0.16666666666666700000000

purify v forgive 0.83333333333333300000000

prosecute v charge 0.02399710691498970000000

prosecute v try 0.56402242140883000000000

prosecute v execute 0.04799421382997950000000

prosecute v continue 0.19600650944127300000000

prosecute v engage 0.09598842765995920000000

prosecute v pursue 0.02399710691498970000000

prosecute v perform 0.04799421382997950000000

friend n confidante 0.00056742238208036500000

friend n peer 0.00020148991916333100000

friend n confidant 0.00154561679536001000000

friend n quaker 0.00038053863810162900000

friend n lover 0.00573007242491981000000

friend n playfellow 0.00169765094521997000000

friend n girl 0.34150172715466700000000

friend n partner 0.03428429037897580000000

friend n well-wisher 0.00419593919380481000000

friend n comrade 0.00207212238611771000000

friend n bedfellow 0.00056142848367810900000

friend n mate 0.02287216533941100000000

friend n financier 0.00004973352756225270000

friend n benefactor 0.13657302789798700000000

friend n fellow 0.24241720626451700000000

friend n patron 0.00038114703394032600000

friend n assistant 0.00039736914442694600000

friend n admirer 0.00004973352756225270000

friend n shadow 0.00935723683115667000000

friend n acquaintance 0.02123316278798050000000

friend n suitor 0.00117324886907344000000

friend n companion 0.01960846912245060000000

friend n ally 0.00051489458352394900000

friend n accomplice 0.00081085334707047000000

friend n schoolfellow 0.00323747592618443000000

friend n cousin 0.02772412333248030000000

friend n advocate 0.00464404920479368000000

friend n angel 0.01978028380229760000000

friend n beau 0.00143644765193318000000

friend n associate 0.00056142848367810800000

friend n mistress 0.09443964461988240000000

unhappy a unsuccessful 0.00013843536046690400000

unhappy a miserable 0.10267595119711200000000

unhappy a morose 0.00010650016465104100000

unhappy a ill 0.02603326845180950000000

unhappy a discontented 0.00135352027438324000000

unhappy a despondent 0.00026830556572846700000

unhappy a cheerless 0.00075492116712079000000

unhappy a melancholy 0.15173398611703100000000

unhappy a unsuitable 0.00135352027438324000000

unhappy a gloomy 0.00663075203631095000000

unhappy a inconsolable 0.00026830556572846700000

unhappy a antagonistic 0.00013843536046690400000

unhappy a unbecoming 0.00079941340228788200000

unhappy a injudicious 0.00079941340228788200000

unhappy a unfortunate 0.01421086522223500000000

unhappy a broken 0.00054517628666227600000

unhappy a poor 0.27231098216661400000000

unhappy a incorrect 0.00118164468398536000000

unhappy a unwise 0.00021300032930208300000

unhappy a foolish 0.01066736718135670000000

unhappy a inauspicious 0.00013843536046690400000

unhappy a wretched 0.00350024726826839000000

unhappy a unprosperous 0.00026830556572846700000

unhappy a dismal 0.00284675593487477000000

unhappy a hopeless 0.00040674092619537100000

unhappy a dreary 0.04217428200505800000000

unhappy a distressed 0.00204645346569852000000

unhappy a awkward 0.00572544706556540000000

unhappy a troubled 0.00088033511611099300000

unhappy a bad 0.07643863787953540000000

unhappy a mournful 0.00322791841024221000000

unhappy a low 0.08391121850060690000000

unhappy a desolate 0.00027687072093380900000

unhappy a sorrowful 0.00429833585637329000000

unhappy a unlucky 0.01298744400935170000000

unhappy a displeased 0.00027687072093380900000

unhappy a disappointed 0.00414143726794078000000

unhappy a forlorn 0.00106601835452618000000

unhappy a threatening 0.00075492116712079000000

unhappy a depressed 0.00414143726794078000000

unhappy a impertinent 0.00618993501652920000000

unhappy a heavy 0.00699035387468904000000

unhappy a crushed 0.00013843536046690400000

unhappy a ominous 0.00013843536046690400000

unhappy a worried 0.00075492116712079000000

unhappy a sad 0.13872160075194700000000

unhappy a cursed 0.00338380068595809000000

unhappy a doleful 0.00048079837518457300000

unhappy a grieved 0.00150984233424158000000

philanthropist n friend 1.00000000000000000000000

favor n consideration 0.00415124980490752000000

favor n help 0.00658216185282633000000

favor n leave 0.00638096039880981000000

favor n regard 0.00205230327433630000000

favor n letter 0.01048556694748240000000

favor n message 0.00415124980490752000000

favor n communication 0.00982747834238322000000

favor n blessing 0.13802376090406100000000

favor n gift 0.00757020419741157000000

favor n harmony 0.03320999843926010000000

favor n allowance 0.00572287179371062000000

favor n answer 0.19070587689887500000000

favor n service 0.05234247211638000000000

favor n guidance 0.00638096039880981000000

favor n present 0.39813972541431800000000

favor n reply 0.11604486660900900000000

favor n advantage 0.00638096039880981000000

favor n kindness 0.00184733240370094000000

quick a alive 0.00845218194721590000000

quick a sharp 0.00082790986317176100000

quick a clever 0.01491070522091470000000

quick a irascible 0.00061828413465051800000

quick a precipitate 0.00168132638891690000000

quick a active 0.00045328192693703800000

quick a lively 0.00015855140362819100000

quick a wise 0.00267179859447363000000

quick a rapid 0.28210618834645100000000

quick a brief 0.00090656385387407600000

quick a intelligent 0.00168132638891690000000

quick a keen 0.01009044985181580000000

quick a ready 0.05388054298288820000000

quick a smart 0.01548162953160490000000

quick a momentary 0.37274040817516300000000

quick a temporary 0.00228510167290940000000

quick a live 0.00310731115334876000000

quick a hasty 0.00168132638891690000000

quick a impatient 0.04433097245444230000000

quick a light-footed 0.00168132638891690000000

quick a able 0.00015855140362819100000

quick a animated 0.00037462793623472300000

quick a fast 0.04433097245444230000000

quick a swift 0.00061828413465051800000

quick a acute 0.04156028667603970000000

quick a energetic 0.00045328192693703800000

quick a handy 0.00045328192693703800000

quick a brisk 0.00276609732989021000000

quick a fiery 0.00037462793623472300000

quick a bright 0.00168132638891690000000

quick a sudden 0.03911556393039030000000

quick a prompt 0.00082790986317176100000

quick a superficial 0.00114255083645470000000

quick a whirlwind 0.00061828413465051800000

quick a warm 0.04577716645226460000000

lonesome a cheerless 0.09090909090909080000000

lonesome a gloomy 0.18181818181818200000000

lonesome a lonely 0.27272727272727300000000

lonesome a dreary 0.09090909090909080000000

lonesome a depressed 0.18181818181818200000000

lonesome a sad 0.18181818181818200000000

sell v push 0.00014945186357092100000

sell v negotiate 0.00703697639683475000000

sell v deceive 0.00325786455529754000000

sell v betray 0.00021647631177120100000

sell v furnish 0.00010823815588560000000

sell v hoodwink 0.00004121370768532120000

sell v dispose 0.00006543683344696110000

sell v exchange 0.00006543683344696110000

sell v move 0.00261921521230182000000

sell v carry 0.00085991213517211400000

sell v do 0.78724820671995200000000

sell v get 0.00519307587642257000000

sell v cheat 0.00006543683344696110000

sell v fool 0.00006543683344696110000

sell v surrender 0.00013087366689392200000

sell v go 0.19234843475791900000000

sell v cross 0.00023187927894156400000

sell v fail 0.00021241899743987800000

sell v delude 0.00004280132243863920000

sell v beguile 0.00004121370768532120000

journey v go 1.00000000000000000000000

reflect v discredit 0.00110847230332164000000

reflect v suggest 0.00022227591966940800000

reflect v display 0.00007269379344147660000

reflect v return 0.01512461975853830000000

reflect v announce 0.00091064499850837400000

reflect v reply 0.09311884487208090000000

reflect v indicate 0.00007269379344147660000

reflect v reveal 0.00177530006232986000000

reflect v betray 0.00060106991794282300000

reflect v study 0.00598308779223930000000

reflect v follow 0.00791044585877535000000

reflect v show 0.00733138030235723000000

reflect v think 0.71711006698738100000000

reflect v flash 0.00159082799845307000000

reflect v contemplate 0.01135014456688510000000

reflect v echo 0.00057261990607708800000

reflect v consider 0.08304362385222540000000

reflect v exhibit 0.00062481582244872600000

reflect v repeat 0.02975604897957920000000

reflect v meditate 0.00023340641139046100000

reflect v heed 0.00023340641139046100000

reflect v catch 0.00260049545512594000000

reflect v express 0.00138334702315865000000

reflect v wonder 0.01162731147880860000000

reflect v concentrate 0.00057261990607708800000

reflect v speculate 0.00007269379344147660000

reflect v present 0.00029496971311088500000

reflect v match 0.00208179984321195000000

reflect v credit 0.00014538758688295400000

reflect v shine 0.00075667630445900900000

reflect v examine 0.00171820858724647000000

hypothesis n reason 0.50000000000000000000000

hypothesis n position 0.50000000000000000000000

hazard v attempt 0.33333333333333400000000

hazard v pretend 0.11111111111111100000000

hazard v try 0.22222222222222200000000

hazard v dare 0.11111111111111100000000

hazard v offer 0.11111111111111100000000

hazard v advance 0.11111111111111100000000

frightening a terrible 1.00000000000000000000000

destroy v discredit 0.00080859190516668800000

destroy v eradicate 0.00020424261668273900000

destroy v deny 0.00040848523336548000000

destroy v conclude 0.00205761893174291000000

destroy v burst 0.00043619358065759100000

destroy v disable 0.00162142535108532000000

destroy v despoil 0.01387807739275890000000

destroy v shoot 0.00840021192466233000000

destroy v reveal 0.01420909253922980000000

destroy v overcome 0.00020424261668273900000

destroy v crush 0.00492541025851683000000

destroy v waste 0.00713999418134514000000

destroy v exhaust 0.01531592688879800000000

destroy v stop 0.01181749669888300000000

destroy v murder 0.00862325279028717000000

destroy v expose 0.01511168427211520000000

destroy v discharge 0.01879072855494350000000

destroy v suppress 0.00020424261668273900000

destroy v break 0.03010286888228410000000

destroy v rob 0.00698302919025974000000

destroy v sacrifice 0.24656524392196900000000

destroy v oppose 0.01428656262612440000000

destroy v drown 0.00104892143070581000000

destroy v overwhelm 0.00043619358065759100000

destroy v conquer 0.00168255955634618000000

destroy v suspend 0.00162142535108532000000

destroy v extinguish 0.00182566796776806000000

destroy v contradict 0.00020424261668273900000

destroy v upset 0.01535639433242230000000

destroy v dispute 0.00202991058445080000000

destroy v kill 0.20462899816153100000000

destroy v trample 0.00020424261668273900000

destroy v smash 0.02680756580461070000000

destroy v hang 0.01230139207299900000000

destroy v consume 0.00162142535108532000000

destroy v undo 0.00061272785004822100000

destroy v dissolve 0.02882245704089270000000

destroy v slay 0.00162142535108532000000

destroy v weaken 0.02615483816327640000000

destroy v overthrow 0.00080859190516668800000

destroy v finish 0.06094958907441040000000

destroy v dissipate 0.00456393149157842000000

destroy v defeat 0.00618535684266375000000

destroy v discontinue 0.00162142535108532000000

destroy v efface 0.00182566796776806000000

destroy v strangle 0.00378023738995893000000

destroy v overturn 0.00324285070217064000000

destroy v scorch 0.00162142535108532000000

destroy v remove 0.03297169907023740000000

destroy v annihilate 0.00182566796776806000000

destroy v reverse 0.00147831693966345000000

destroy v end 0.01577805609348040000000

destroy v vanquish 0.00162142535108532000000

destroy v dispel 0.00215881203887360000000

destroy v assassinate 0.00324285070217064000000

destroy v ruin 0.06826625007729970000000

destroy v avoid 0.03362123103778550000000

destroy v cancel 0.00182566796776806000000

destroy v demolish 0.00309974229074877000000

destroy v exterminate 0.00043619358065759100000

expostulate v warn 0.01204403073561950000000

expostulate v object 0.01605870764749270000000

expostulate v reproach 0.00802935382374634000000

expostulate v argue 0.00401467691187317000000

expostulate v protest 0.85807027863102600000000

expostulate v remonstrate 0.10178295225024200000000

enquiry n question 1.00000000000000000000000

delight n satisfaction 0.04604850277672050000000

delight n excitement 0.20739247465231900000000

delight n charm 0.00012413854275587300000

delight n pleasure 0.52574922450500000000000

delight n paradise 0.01797040339589920000000

delight n happiness 0.14287474009849500000000

delight n delirium 0.00006206927137793650000

delight n joy 0.05977844675743270000000

diet n dinner 0.50000000000000000000000

diet n lunch 0.50000000000000000000000

detail v enumerate 0.05523964256701870000000

detail v indicate 0.05523964256701870000000

detail v show 0.05523964256701870000000

detail v tell 0.38667749796913100000000

detail v mention 0.16571892770105600000000

detail v describe 0.28188464662875700000000

aside r yet 0.00992929508441347000000

aside r apart 0.21968565374264800000000

aside r however 0.00262929099208160000000

aside r alone 0.00078319306147111600000

aside r out 0.25107877208031100000000

aside r down 0.04635877820958470000000

aside r away 0.38536325277486100000000

aside r still 0.08417176405462900000000

bust v burst 0.12500000000000000000000

bust v break 0.12500000000000000000000

bust v catch 0.12500000000000000000000

bust v fail 0.50000000000000100000000

bust v tear 0.12500000000000000000000

biography n portrait 0.50000000000000000000000

biography n life 0.50000000000000000000000

mad a sick 0.03976633346172070000000

mad a preposterous 0.00347630970302083000000

mad a stupid 0.02612371704557730000000

mad a nonsensical 0.00065021680079037500000

mad a injudicious 0.00077993793306999500000

mad a light-headed 0.00053981984162905300000

mad a violent 0.00198078058593178000000

mad a groundless 0.01254494371090130000000

mad a odd 0.01658443569862440000000

mad a eager 0.01051034517766050000000

mad a dangerous 0.02414390836246080000000

mad a foolish 0.01440704015173270000000

mad a laughable 0.00299386464695958000000

mad a absurd 0.02988231306203190000000

mad a ridiculous 0.02245938227288000000000

mad a excited 0.00487181530940039000000

mad a silly 0.00768487219336367000000

mad a fierce 0.00202997520803013000000

mad a childish 0.00187795066244081000000

mad a passionate 0.00187795066244081000000

mad a furious 0.00187795066244081000000

mad a confused 0.00195065040237113000000

mad a irritated 0.00464739544029180000000

mad a devoted 0.01881144953172930000000

mad a crazy 0.33809949259667900000000

mad a unreasonable 0.00585417411468032000000

mad a frantic 0.00710957063319291000000

mad a indignant 0.00299386464695958000000

mad a displeased 0.00868810141634709000000

mad a sore 0.00202997520803013000000

mad a reckless 0.00412652650381121000000

mad a ardent 0.00249812731905393000000

mad a giddy 0.00541824152563994000000

mad a disturbed 0.01043102037200150000000

mad a delirious 0.01950680496920870000000

mad a queer 0.00302175393383746000000

mad a improper 0.19813985651927600000000

mad a fiery 0.00065021680079037500000

mad a extravagant 0.00072954160644937800000

mad a insane 0.00141785604594953000000

mad a angry 0.08762682927183480000000

mad a incoherent 0.01602965029726270000000

mad a imprudent 0.00065021680079037500000

mad a wild 0.01676347294625570000000

mad a perilous 0.00187795066244081000000

mad a idiotic 0.00346954986241791000000

mad a inconsistent 0.00065021680079037500000

mad a upset 0.00786567995635997000000

mad a absent-minded 0.00187795066244081000000

average a normal 1.00000000000000000000000

atrocious a hideous 1.00000000000000000000000

fool n mark 0.01754796401884960000000

fool n balloon 0.00827686226022235000000

fool n imbecile 0.00471961173067483000000

fool n dupe 0.00839839732624834000000

fool n droll 0.00943922346134969000000

fool n lubber 0.00217059756952782000000

fool n ninny 0.01343730619812260000000

fool n numskull 0.01343730619812260000000

fool n dummy 0.25066382302917600000000

fool n victim 0.10067125726546400000000

fool n cretin 0.00269835203161357000000

fool n idiot 0.01699455672767010000000

fool n goose 0.14338511118751200000000

fool n ass 0.01657940859037500000000

fool n turkey 0.00471961173067483000000

fool n featherbrain 0.04229952013617340000000

fool n pigeon 0.00242057339835041000000

fool n simpleton 0.21149760068086700000000

fool n noddy 0.00052775446208575400000

fool n blockhead 0.13011516199692000000000

curiosity n wonder 0.00117826466841558000000

curiosity n regard 0.02765091724163990000000

curiosity n search 0.00226064114296425000000

curiosity n concern 0.00637466207061600000000

curiosity n business 0.00643630993243281000000

curiosity n miracle 0.00013621345771548400000

curiosity n sight 0.00304476443053663000000

curiosity n peculiarity 0.00109163241710464000000

curiosity n interest 0.45357608384918500000000

curiosity n attention 0.49825051078939000000000

us n u.s. 1.00000000000000000000000

upbraid v expostulate 0.01394181334301090000000

upbraid v blame 0.32002661343978700000000

upbraid v condemn 0.15272485332365600000000

upbraid v accuse 0.01394181334301090000000

upbraid v reproach 0.24968245327526800000000

upbraid v scold 0.11089941329462300000000

upbraid v remonstrate 0.01394181334301090000000

upbraid v denounce 0.02788362668602190000000

upbraid v damn 0.09695759995161190000000

queen n star 0.00281002134553559000000

queen n nance 0.00283526782547041000000

queen n queer 0.00281002134553559000000

queen n pouf 0.00269283848403263000000

queen n king 0.97171350863908400000000

queen n princess 0.01713834236034150000000

unaware a dull 0.49492565494453600000000

unaware a innocent 0.00507434505546376000000

unaware a stupid 0.49492565494453600000000

unaware a blind 0.00507434505546376000000

turmoil n excitement 0.10348837209302300000000

turmoil n distress 0.10348837209302300000000

turmoil n agitation 0.20697674418604700000000

turmoil n struggle 0.20697674418604700000000

turmoil n disorder 0.10348837209302300000000

turmoil n scrap 0.08604651162790700000000

turmoil n trouble 0.18953488372093000000000

dine v treat 0.00707686476274229000000

dine v lunch 0.01622920488508720000000

dine v feed 0.00707686476274229000000

dine v eat 0.69886742659102000000000

dine v breakfast 0.22001319419201200000000

dine v sup 0.05073644480639560000000

suppression n repression 0.12352130469737000000000

suppression n screening 0.12352130469737000000000

suppression n destruction 0.12746449217105000000000

suppression n restraint 0.12746449217105000000000

suppression n confinement 0.12746449217105000000000

suppression n arrest 0.12352130469737000000000

suppression n defeat 0.12352130469737000000000

suppression n impediment 0.12352130469737000000000

stupefaction n surprise 1.00000000000000000000000

stoppage n conclusion 0.03134719266655750000000

stoppage n end 0.71787526600098300000000

stoppage n rest 0.03134719266655750000000

stoppage n barrier 0.06269438533311500000000

stoppage n interruption 0.06269438533311500000000

stoppage n arrest 0.03134719266655750000000

stoppage n hindrance 0.03134719266655750000000

stoppage n hitch 0.03134719266655750000000

stipulate v make 0.30952380952381000000000

stipulate v impose 0.04761904761904760000000

stipulate v give 0.26190476190476200000000

stipulate v limit 0.02380952380952380000000

stipulate v agree 0.07142857142857140000000

stipulate v settle 0.02380952380952380000000

stipulate v provide 0.11904761904761900000000

stipulate v assure 0.04761904761904760000000

stipulate v require 0.02380952380952380000000

stipulate v designate 0.02380952380952380000000

stipulate v arrange 0.04761904761904760000000

suffering n anxiety 0.02904802857654460000000

suffering n blow 0.00024684952956121200000

suffering n sorrow 0.00024684952956121200000

suffering n grief 0.18215620840096200000000

suffering n misery 0.01126054024211150000000

suffering n cramp 0.00024684952956121200000

suffering n misfortune 0.01313941741616980000000

suffering n agony 0.00049369905912242300000

suffering n concern 0.02749124234481710000000

suffering n woe 0.00063592563082292500000

suffering n load 0.17279467069284800000000

suffering n trouble 0.26871328426118500000000

suffering n care 0.00356995492061973000000

suffering n pain 0.00743634207803151000000

suffering n difficulty 0.10997231662479400000000

suffering n discomfort 0.17254782116328700000000

sojourn n stay 1.00000000000000000000000

seduce v make 0.80000000000000000000000

seduce v ruin 0.20000000000000000000000

saddle v embarrass 1.00000000000000000000000

charming a appealing 0.02862770903938590000000

charming a nice 0.05527253533702790000000

charming a delightful 0.14381031152801100000000

charming a delicate 0.00193244359759124000000

charming a lovely 0.04360175889133240000000

charming a amiable 0.08974958250871050000000

charming a smooth 0.00364051791252306000000

charming a elegant 0.07100794497024140000000

charming a exquisite 0.00924727960026580000000

charming a graceful 0.00215688218789893000000

charming a desirable 0.00080375896631585400000

charming a attractive 0.01004950024711020000000

charming a beautiful 0.19608238614238200000000

charming a fascinating 0.03305166295389820000000

charming a good-looking 0.00896513793758203000000

charming a sweet 0.06892748784097760000000

charming a handsome 0.05804297980503120000000

charming a magic 0.00095425696797952900000

charming a fair 0.01659958871495550000000

charming a pretty 0.10352029411829400000000

charming a agreeable 0.05395598073248630000000

retract v withdraw 0.84612332153372300000000

retract v deny 0.11595132696860000000000

retract v contradict 0.02034001587995620000000

retract v dissolve 0.00201266251554168000000

retract v recall 0.01356001058663740000000

retract v cancel 0.00201266251554168000000

restrict v tie 0.16666666666666700000000

restrict v confine 0.33333333333333300000000

restrict v limit 0.16666666666666700000000

restrict v assign 0.16666666666666700000000

restrict v temper 0.16666666666666700000000

rendezvous n meeting 1.00000000000000000000000

act n work 0.14860565809918900000000

act n blow 0.00841492866676782000000

act n rule 0.00017848257933256700000

act n bill 0.04883860654376310000000

act n command 0.01378831685157140000000

act n motion 0.00017848257933256700000

act n proposal 0.01378831685157140000000

act n order 0.00017848257933256700000

act n document 0.00017848257933256700000

act n exertion 0.00017848257933256700000

act n operation 0.13463885866828500000000

act n execution 0.01387188240824760000000

act n introduction 0.00547574305588719000000

act n law 0.57915109054433500000000

act n appearance 0.00017848257933256700000

act n enterprise 0.00017848257933256700000

act n gig 0.00017848257933256700000

act n action 0.02669356836185290000000

act n front 0.00185784139396173000000

act n turn 0.00185784139396173000000

act n bit 0.00158948652594503000000

justice n consideration 0.01233068219278730000000

justice n faith 0.00872983041727145000000

justice n judge 0.00069379161812344400000

justice n sentence 0.00069379161812344400000

justice n rule 0.00244379765925251000000

justice n virtue 0.00069379161812344400000

justice n goodness 0.75151803119680000000000

justice n judgment 0.00138758323624689000000

justice n feeling 0.03492549955202430000000

justice n decision 0.00069379161812344400000

justice n right 0.08849578376994150000000

justice n referee 0.00069379161812344400000

justice n determination 0.00256123416004039000000

justice n nobility 0.00349821059183626000000

justice n law 0.01201292242601920000000

justice n opinion 0.01044093093172680000000

justice n truth 0.02948477348267800000000

justice n honor 0.02362932655383390000000

justice n chancellor 0.00256123416004039000000

justice n honesty 0.00069379161812344400000

justice n justification 0.00854396433540193000000

justice n verdict 0.00138758323624689000000

justice n sanction 0.00188586238911146000000

masterpiece n treasure 0.71985624438454600000000

masterpiece n prize 0.28014375561545400000000

despair n nuisance 0.00215685494967261000000

despair n scourge 0.00107842747483630000000

despair n sorrow 0.00107842747483630000000

despair n misery 0.00107842747483630000000

despair n distress 0.31352299285800600000000

despair n trial 0.00521581897015061000000

despair n gloom 0.00433439713880323000000

despair n burden 0.00107842747483630000000

despair n curse 0.00107842747483630000000

despair n extremity 0.17112522528980400000000

despair n desperation 0.17112522528980400000000

despair n nightmare 0.00215685494967261000000

despair n pain 0.32497049317990700000000

low-spirited a gloomy 0.50000000000000000000000

low-spirited a depressed 0.50000000000000000000000

literal a plain 0.25000000000000000000000

literal a real 0.25000000000000000000000

literal a clear 0.25000000000000000000000

literal a simple 0.25000000000000000000000

lethargy n stupefaction 1.00000000000000000000000

lass n girl 1.00000000000000000000000

fetch v bring 0.08539725427480070000000

fetch v entrap 0.00007177550604197920000

fetch v deliver 0.00280837980004253000000

fetch v yield 0.02150189088143290000000

fetch v make 0.02459380738096620000000

fetch v take 0.09762331067881620000000

fetch v collect 0.00071491731728004900000

fetch v conduct 0.00007177550604197920000

fetch v summon 0.00014355101208395800000

fetch v grasp 0.00007177550604197920000

fetch v snatch 0.00075291634070756300000

fetch v bear 0.00014355101208395800000

fetch v delight 0.00007177550604197920000

fetch v accomplish 0.00204342250399675000000

fetch v discharge 0.00014355101208395800000

fetch v carry 0.03707108992667450000000

fetch v execute 0.00146791080474113000000

fetch v procure 0.00007177550604197920000

fetch v do 0.47311334828801200000000

fetch v get 0.23708487005946800000000

fetch v lead 0.00014355101208395800000

fetch v raise 0.00295193081212649000000

fetch v capture 0.00021532651812593700000

fetch v catch 0.00378998786202099000000

fetch v effect 0.00014355101208395800000

fetch v fascinate 0.00012680137840777600000

fetch v transport 0.00684151073500139000000

fetch v bewitch 0.00007177550604197920000

fetch v perform 0.00075291634070756300000

incompatible a unbecoming 0.50000000000000000000000

incompatible a unsympathetic 0.50000000000000000000000

immoral a vile 0.50000000000000000000000

immoral a shameless 0.50000000000000000000000

work n charge 0.00234837285788934000000

work n act 0.00983716583207805000000

work n masterpiece 0.00004859346627770350000

work n place 0.03242655596906010000000

work n child 0.08014513010938970000000

work n post 0.00014653232867880000000

work n outcome 0.00001806323651095370000

work n heart 0.02098115952817760000000

work n station 0.00090783131349480400000

work n engagement 0.00132422209732027000000

work n handiwork 0.00045636875859602300000

work n attempt 0.03791473343649180000000

work n trial 0.00076272569123721700000

work n task 0.05334532249272130000000

work n occupation 0.03810340581785250000000

work n struggle 0.00008632322803545260000

work n trade 0.00201075265631783000000

work n deed 0.00328252422647985000000

work n profession 0.00270561478310500000000

work n pursuit 0.00316774488358150000000

work n call 0.00006825999152449900000

work n activity 0.00011256643971313500000

work n business 0.05527874205798340000000

work n employment 0.01558188150088190000000

work n practice 0.00022795725912309400000

work n exertion 0.00001181491171661570000

work n operation 0.00462090226677445000000

work n creation 0.00125803239803454000000

work n job 0.00104014052502189000000

work n strain 0.00090187159436833100000

work n capacity 0.00043043871549397600000

work n opera 0.00025028976608407900000

work n errand 0.00006825999152449900000

work n endeavor 0.00036954738500946300000

work n position 0.00109231058418403000000

work n move 0.00001806323651095370000

work n line 0.00112166154716216000000

work n duty 0.00769542652527404000000

work n trouble 0.01008634341144820000000

work n transaction 0.00001806323651095370000

work n accomplishment 0.00045587061304491500000

work n service 0.02661665596798830000000

work n piece 0.01922115680865520000000

work n invention 0.00045636875859602300000

work n enterprise 0.00068006059443631000000

work n discipline 0.00037722785624768100000

work n effort 0.37938090395891700000000

work n issue 0.00014912837387192900000

work n effect 0.00148930060848182000000

work n gig 0.00033146560339146700000

work n projects 0.00025507479436680100000

work n factory 0.00001806323651095370000

work n harvest 0.00020238735798769300000

work n action 0.00292866337889704000000

work n undertaking 0.00011781837313676500000

work n slavery 0.01893930348173500000000

work n movement 0.04053401446194000000000

work n office 0.00443697650802267000000

work n turn 0.00069533675072499700000

work n feat 0.00204749226829567000000

work n field 0.00074296169599485200000

work n workshop 0.00075757213926939800000

work n art 0.02444225840880290000000

work n situation 0.01752771533256860000000

work n toil 0.00095009510562312900000

work n result 0.06384158403673760000000

work n study 0.00213282746811511000000

behold v consider 0.00289931572101775000000

behold v observe 0.01001207783117070000000

behold v witness 0.00765367727538383000000

behold v catch 0.00205498494864124000000

behold v watch 0.01503137953646530000000

behold v notice 0.01375572432456500000000

behold v discover 0.00093591162334856600000

behold v see 0.94662943626508700000000

behold v examine 0.00102749247432062000000

headstrong a contrary 0.04632063074901440000000

headstrong a determined 0.04632063074901440000000

headstrong a reckless 0.04632063074901440000000

headstrong a obstinate 0.09264126149802890000000

headstrong a bull-headed 0.04632063074901440000000

headstrong a imprudent 0.62943495400788500000000

headstrong a difficult 0.09264126149802890000000

glimmer v flicker 0.50000000000000000000000

glimmer v twinkle 0.50000000000000000000000

fruitless a unavailing 0.49354838709677400000000

fruitless a vain 0.00645161290322581000000

fruitless a useless 0.00645161290322581000000

fruitless a idle 0.49354838709677400000000

papa n parent 0.01297747914466250000000

papa n governor 0.01837125234472560000000

papa n pa 0.05312318774681630000000

papa n pap 0.00610137865269317000000

papa n father 0.90942670211110300000000

flinch v withdraw 0.04644600600745610000000

flinch v shirk 0.05383438006504130000000

flinch v blanch 0.02322300300372800000000

flinch v retreat 0.02322300300372800000000

flinch v shrink 0.53659782075641200000000

flinch v flee 0.04644600600745610000000

flinch v start 0.02322300300372800000000

flinch v tremble 0.06966900901118410000000

flinch v escape 0.15411476613753900000000

flinch v avoid 0.02322300300372800000000

favored a prosperous 1.00000000000000000000000

fabulous a great 1.00000000000000000000000

explicit a plain 0.16486424298205200000000

explicit a direct 0.10687988955361200000000

explicit a certain 0.02899217671422000000000

explicit a exact 0.10687988955361200000000

explicit a positive 0.21375977910722500000000

explicit a open 0.10687988955361200000000

explicit a free 0.13587206626783200000000

explicit a frank 0.01449608835711000000000

explicit a precise 0.01449608835711000000000

explicit a candid 0.10687988955361200000000

enchanted a delighted 1.00000000000000000000000

burn v squander 0.00023391717584083400000

burn v hurt 0.01334757395448320000000

burn v wish 0.12427083327763700000000

burn v excite 0.00159974134479411000000

burn v wound 0.00433138968270069000000

burn v exhaust 0.00136582416895328000000

burn v smoke 0.02482749547834830000000

burn v torment 0.00023391717584083400000

burn v warm 0.00023391717584083400000

burn v grieve 0.00160271379302269000000

burn v fire 0.00990047804549906000000

burn v rob 0.00136582416895328000000

burn v vex 0.00663229102045550000000

burn v bite 0.00160271379302269000000

burn v injure 0.00222864328601996000000

burn v ache 0.12498974429095300000000

burn v boil 0.01362167709197450000000

burn v annoy 0.00650101092258769000000

burn v blaze 0.08332649619396860000000

burn v illuminate 0.00090096226550018100000

burn v rage 0.00580223184329375000000

burn v consume 0.14374100463016900000000

burn v cut 0.00222864328601996000000

burn v sting 0.00222864328601996000000

burn v light 0.32526951749118900000000

burn v flicker 0.04225005440820940000000

burn v thrill 0.00023391717584083400000

burn v twinkle 0.04225005440820940000000

burn v irritate 0.00023391717584083400000

burn v stir 0.01081416591640450000000

burn v desire 0.00183068607240638000000

droll a odd 0.00152940258393846000000

droll a absurd 0.02641429048631980000000

droll a ridiculous 0.03641959910868230000000

droll a strange 0.64134187898612700000000

droll a merry 0.01112026464693510000000

droll a amusing 0.00152940258393846000000

droll a funny 0.24828436766325400000000

droll a queer 0.02224052929387020000000

droll a comic 0.01112026464693510000000

dishonorable a outrageous 0.01608598185399010000000

dishonorable a ignominious 0.00804299092699501000000

dishonorable a unbecoming 0.06950811269867170000000

dishonorable a evil 0.00779417637061102000000

dishonorable a venal 0.00779417637061102000000

dishonorable a mean 0.06742676718774910000000

dishonorable a bad 0.38928819758557600000000

dishonorable a low 0.08466789633262490000000

dishonorable a unworthy 0.08466789633262490000000

dishonorable a unjust 0.00804299092699501000000

dishonorable a shameful 0.01608598185399010000000

dishonorable a odious 0.04239204483295070000000

dishonorable a infamous 0.02412897278098510000000

dishonorable a shocking 0.01583716729760600000000

dishonorable a disgraceful 0.05018622120356170000000

dishonorable a offensive 0.00779417637061102000000

dishonorable a improper 0.00779417637061102000000

dishonorable a unfair 0.00779417637061102000000

dishonorable a despicable 0.08466789633262490000000

discredit v reject 0.25000000000000000000000

discredit v ruin 0.75000000000000000000000

derision n cheek 1.00000000000000000000000

various a many 0.89041139270570400000000

various a individual 0.00629339109488326000000

various a uncertain 0.00629339109488326000000

various a other 0.03406791415569650000000

various a different 0.06293391094883270000000

dealings n behavior 0.00315867322794941000000

dealings n trade 0.94624825128713000000000

dealings n business 0.02845521097040960000000

dealings n practice 0.01897919128656140000000

dealings n transaction 0.00157933661397470000000

dealings n action 0.00157933661397470000000

damp v break 1.00000000000000000000000

satisfaction n delight 0.04863258779566020000000

satisfaction n expiation 0.00060097920649807700000

satisfaction n ease 0.00186470212029915000000

satisfaction n relief 0.07787433651747540000000

satisfaction n conciliation 0.00013110477464757800000

satisfaction n amusement 0.11514921055161100000000

satisfaction n reward 0.00954646727372543000000

satisfaction n pleasure 0.51464455241782200000000

satisfaction n entertainment 0.00013110477464757800000

satisfaction n indulgence 0.02910088981260760000000

satisfaction n prize 0.00046987443185049900000

satisfaction n happiness 0.03319102268481510000000

satisfaction n joy 0.15154970291283900000000

satisfaction n comfort 0.01510437794742530000000

satisfaction n compensation 0.00140810757157779000000

satisfaction n recompense 0.00046987443185049900000

satisfaction n pride 0.00013110477464757800000

permission n promise 0.01116684928088700000000

permission n grace 0.00279171232022174000000

permission n blessing 0.90353769243977100000000

permission n consent 0.07971203363889820000000

permission n admission 0.00279171232022174000000

bowl v move 1.00000000000000000000000

blameless a innocent 0.20000000000000000000000

blameless a good 0.80000000000000000000000

difference n strife 0.00040526051799039800000

difference n discord 0.00068156010492365100000

difference n contention 0.00040526051799039800000

difference n feud 0.00040526051799039800000

difference n interval 0.00020784820557876700000

difference n departure 0.02253646455781500000000

difference n digression 0.00081052103598079800000

difference n judgment 0.00081052103598079800000

difference n row 0.07362029320134580000000

difference n question 0.39095522832024400000000

difference n argument 0.01563653684658810000000

difference n point 0.21004799292242200000000

difference n separation 0.00108682062291405000000

difference n division 0.04883389241784320000000

difference n distinction 0.00241193259040140000000

difference n contrast 0.04137001856309820000000

difference n exception 0.07514728220866130000000

difference n destruction 0.00040526051799039800000

difference n rest 0.01799435469307830000000

difference n opposition 0.04151025591415950000000

difference n peculiarity 0.00061310872356916500000

difference n conflict 0.00020784820557876700000

difference n contradiction 0.00020784820557876700000

difference n cause 0.01448231468340430000000

difference n misunderstanding 0.02096297616265850000000

difference n disagreement 0.01824333870621360000000

bankruptcy n collapse 1.00000000000000000000000

aback r back 1.00000000000000000000000

beginning n heart 0.45532447465591100000000

beginning n prelude 0.00434356235341458000000

beginning n establishment 0.00156699413586690000000

beginning n head 0.03239081384941650000000

beginning n top 0.50637415500539100000000

zest n appetite 0.16666666666666700000000

zest n eagerness 0.16666666666666700000000

zest n interest 0.16666666666666700000000

zest n gaiety 0.16666666666666700000000

zest n life 0.16666666666666700000000

zest n passion 0.16666666666666700000000

whimper v fuss 0.00280287375896897000000

whimper v weep 0.10892137298490400000000

whimper v cry 0.74189289999110300000000

whimper v snivel 0.10433974688048900000000

whimper v complain 0.01401436879484490000000

whimper v object 0.02242299007175180000000

whimper v moan 0.00280287375896897000000

whimper v tear 0.00280287375896897000000

vogue n fashion 0.33333333333333300000000

vogue n thing 0.66666666666666700000000

private a discreet 0.00519235624549892000000

private a secret 0.68452563169827400000000

private a individual 0.00279969583516676000000

private a singular 0.00279969583516676000000

private a intimate 0.00279969583516676000000

private a confidential 0.18668880864498400000000

private a quiet 0.02641157946983600000000

private a own 0.07799078852007430000000

private a particular 0.01079174791583240000000

unavailing a fruitless 0.96382428940568500000000

unavailing a vain 0.01808785529715760000000

unavailing a useless 0.01808785529715760000000

thump v knock 1.00000000000000000000000

taunt v upbraid 0.07692307692307680000000

taunt v torment 0.07692307692307680000000

taunt v reproach 0.30769230769230800000000

taunt v bother 0.23076923076923100000000

taunt v tease 0.15384615384615400000000

taunt v ride 0.07692307692307680000000

taunt v jeer 0.07692307692307680000000

create v make 0.09602185833763860000000

create v build 0.00511698674295504000000

create v conceive 0.02450305651769330000000

create v occasion 0.06916185307413420000000

create v form 0.03228907447658640000000

create v imagine 0.00574803604637576000000

create v devise 0.00287401802318788000000

create v cause 0.71467248176605300000000

create v perform 0.04961263501537530000000

strife n contention 0.00030840400925211800000

strife n game 0.00061680801850423900000

strife n match 0.00061680801850423900000

strife n trial 0.00030840400925211800000

strife n struggle 0.00092521202775635900000

strife n quarrelling 0.00030840400925211800000

strife n argument 0.00030840400925211800000

strife n round 0.00030840400925211800000

strife n fight 0.00061680801850423900000

strife n bout 0.00030840400925211800000

strife n combat 0.99506553585196600000000

strife n fray 0.00030840400925211800000

squander v waste 0.08333333333333310000000

squander v lose 0.25000000000000000000000

squander v kill 0.66666666666666700000000

confess v swear 0.00708711252329854000000

confess v own 0.06422555205941330000000

confess v relate 0.00276691061267976000000

confess v acknowledge 0.11883332764434100000000

confess v reveal 0.00528749304433089000000

confess v grant 0.00050780762526047900000

confess v state 0.00281480953503770000000

confess v confide 0.00073040444456222600000

confess v assert 0.03539652308263090000000

confess v allow 0.02197329440357670000000

confess v disclose 0.00013820537620489500000

confess v affirm 0.00025924214088687400000

confess v vent 0.00718991875115941000000

confess v accept 0.00179957094424607000000

confess v recognize 0.17602730810015000000000

confess v sing 0.00186346356101640000000

confess v admit 0.55106906217237000000000

confess v declare 0.00202999397883506000000

scruple v hesitate 0.00821355236139630000000

scruple v question 0.00410677618069815000000

scruple v object 0.01642710472279260000000

scruple v falter 0.00205338809034907000000

scruple v stumble 0.00205338809034907000000

scruple v waver 0.00205338809034907000000

scruple v doubt 0.96509240246406600000000

repine v fuss 0.00060725383413432200000

repine v regret 0.57134287095391200000000

repine v complain 0.42440635220714800000000

repine v moan 0.00060725383413432200000

repine v fret 0.00303626917067162000000

gentle a noble 0.76586855058701400000000

gentle a kind 0.07994079656912700000000

gentle a faint 0.00011868410825771100000

gentle a broken 0.00023736821651542300000

gentle a slow 0.00023736821651542300000

gentle a respectable 0.00258492009278220000000

gentle a thoughtful 0.00023736821651542300000

gentle a low 0.00743561699599042000000

gentle a suitable 0.00922733193478330000000

gentle a fine 0.00779166932076356000000

gentle a soothing 0.07658685505870140000000

gentle a quiet 0.00209479042014650000000

gentle a moderate 0.00743561699599042000000

gentle a merciful 0.00102663344582710000000

gentle a acceptable 0.00011868410825771100000

gentle a undisturbed 0.00102663344582710000000

gentle a easy 0.01792958063353710000000

gentle a compassionate 0.00102663344582710000000

gentle a affable 0.01781089652527940000000

gentle a calm 0.00126400166234252000000

practicable a feasible 0.12500000000000000000000

practicable a sensible 0.12500000000000000000000

practicable a possible 0.50000000000000000000000

practicable a serviceable 0.25000000000000000000000

popularity n fashion 1.00000000000000000000000

satisfy v conciliate 0.00096268536419857800000

satisfy v recompense 0.00059761844471166800000

satisfy v quit 0.00210970369628091000000

satisfy v charge 0.00967524547041898000000

satisfy v enrapture 0.00007039187212388260000

satisfy v convince 0.00346357128846421000000

satisfy v amuse 0.00817569144806569000000

satisfy v satiate 0.00096268536419857800000

satisfy v answer 0.03447124179003560000000

satisfy v relieve 0.00007039187212388260000

satisfy v bestow 0.00770212413654336000000

satisfy v suit 0.00541860839546634000000

satisfy v meet 0.01070339091941050000000

satisfy v furnish 0.00025502752838662400000

satisfy v entertain 0.00229282075515620000000

satisfy v complete 0.00381364824876156000000

satisfy v lend 0.00070459072001805200000

satisfy v delight 0.00351740809673851000000

satisfy v accomplish 0.01126409065041700000000

satisfy v pay 0.03509282593342120000000

satisfy v cheer 0.00022733374385261300000

satisfy v rejoice 0.00221648638078418000000

satisfy v please 0.09699132003608700000000

satisfy v indulge 0.00146934215290881000000

satisfy v gratify 0.00168692707101564000000

satisfy v do 0.60505499048532300000000

satisfy v give 0.09354326238494840000000

satisfy v flatter 0.00089001040828388100000

satisfy v observe 0.00244839866735159000000

satisfy v cure 0.00270738144331636000000

satisfy v fulfil 0.00093541732318870700000

satisfy v suffice 0.00023021172152804400000

satisfy v capture 0.00054613531776100600000

satisfy v repay 0.00011510586076402200000

satisfy v reassure 0.00011472638356517400000

satisfy v comfort 0.00503406287637432000000

satisfy v settle 0.00752727558366153000000

satisfy v fascinate 0.00009149961620566010000

satisfy v reward 0.00427115196965429000000

satisfy v provide 0.00207921169103553000000

satisfy v fill 0.00022983224432919600000

satisfy v assure 0.00725288752495465000000

satisfy v serve 0.00213715314745308000000

satisfy v appease 0.01765834385913330000000

satisfy v atone 0.00018895553143615700000

satisfy v quench 0.00011856365931227500000

satisfy v pacify 0.00015906089500646600000

satisfy v equip 0.00084349574767875400000

satisfy v perform 0.00190769427814532000000

treat v stand 0.02544417112852150000000

treat v dine 0.00017706572828004000000

treat v satisfy 0.00902812209287782000000

treat v remedy 0.00167455081081359000000

treat v manage 0.02105707042877550000000

treat v amuse 0.00412473979619394000000

treat v employ 0.00229827014115712000000

treat v think 0.09331937141865510000000

treat v use 0.36096880794253100000000

treat v advise 0.00229827014115712000000

treat v entertain 0.00063963123928541700000

treat v discuss 0.00755791082290066000000

treat v handle 0.00167455081081359000000

treat v consult 0.00202740258880646000000

treat v deal 0.01191846285591210000000

treat v indulge 0.00008853286414001970000

treat v consider 0.00153189323392636000000

treat v give 0.03781573297311540000000

treat v cure 0.00370422658966270000000

treat v refresh 0.00370422658966270000000

treat v contract 0.00229827014115712000000

treat v agree 0.00621361735362328000000

treat v explain 0.01865525094332790000000

treat v approach 0.00144336036978634000000

treat v dress 0.02175485126528190000000

treat v settle 0.01104243033010420000000

treat v address 0.00081669696756545700000

treat v prepare 0.00654027401203129000000

treat v engage 0.00357753261972796000000

treat v regard 0.00926459605629474000000

treat v cover 0.00420030482326942000000

treat v reason 0.01720220378381240000000

treat v operate 0.28383636243290400000000

treat v attend 0.00135482750564632000000

treat v buy 0.00176308367495361000000

treat v arrange 0.00370422658966270000000

treat v contain 0.00176308367495361000000

treat v face 0.01351601725870970000000

morose a unhappy 0.11111111111111100000000

morose a melancholy 0.11111111111111100000000

morose a severe 0.11111111111111100000000

morose a dismal 0.11111111111111100000000

morose a low 0.22222222222222200000000

morose a sober 0.11111111111111100000000

morose a silent 0.11111111111111100000000

morose a sad 0.11111111111111100000000

ill a harmful 0.00054501341938712900000

ill a sick 0.01700454352079060000000

ill a weak 0.01199938771221930000000

ill a destructive 0.00122656165595030000000

ill a antagonistic 0.00262458913351849000000

ill a unfortunate 0.01661792179515240000000

ill a irascible 0.00262458913351849000000

ill a poor 0.14076240439188700000000

ill a infirm 0.00247392253558208000000

ill a dangerous 0.01380555155704710000000

ill a wicked 0.15610340237836700000000

ill a rough 0.00247392253558208000000

ill a evil 0.00054501341938712900000

ill a inauspicious 0.00262458913351849000000

ill a feeble 0.04782442187717800000000

ill a mean 0.00330613737008167000000

ill a bad 0.24183030324613700000000

ill a low 0.05304102239436340000000

ill a unlucky 0.00968370290169396000000

ill a sore 0.00383701900870843000000

ill a wrong 0.16286078287194800000000

ill a vile 0.00572216540281167000000

ill a irritable 0.00068154823656317500000

ill a threatening 0.00068154823656317500000

ill a cross 0.01703226167852980000000

ill a unkind 0.04480548592220880000000

ill a unwell 0.01149281036777910000000

ill a sinful 0.01475302585243460000000

ill a ominous 0.00262458913351849000000

ill a deficient 0.00344391810640840000000

ill a objectionable 0.00247392253558208000000

ill a ruinous 0.00247392253558208000000

imbecile a stupid 0.00612745098039216000000

imbecile a absurd 0.00612745098039216000000

imbecile a silly 0.01225490196078430000000

imbecile a defective 0.00612745098039216000000

imbecile a vacant 0.96936274509803900000000

harmful a ill 0.09677419354838710000000

harmful a destructive 0.03225806451612890000000

harmful a painful 0.03225806451612890000000

harmful a cruel 0.06451612903225800000000

harmful a dangerous 0.09677419354838710000000

harmful a evil 0.03225806451612890000000

harmful a bad 0.32258064516129100000000

harmful a serious 0.29032258064516200000000

harmful a unwholesome 0.03225806451612890000000

hand v pass 0.03897625684491890000000

hand v return 0.07177121560520440000000

hand v will 0.01636297134821280000000

hand v help 0.02283969413269790000000

hand v get 0.23472679422133300000000

hand v give 0.61446523896002000000000

hand v present 0.00085782888761306500000

expel v remove 1.00000000000000000000000

excitedly r eagerly 0.60000000000000000000000

excitedly r wildly 0.20000000000000000000000

excitedly r passionately 0.20000000000000000000000

dubiously r doubtfully 1.00000000000000000000000

quite r completely 0.03980758071319370000000

quite r absolutely 0.02131224167518900000000

quite r fully 0.01717831874654990000000

quite r truly 0.01908612577561150000000

quite r actually 0.00110597486764002000000

quite r indeed 0.02634811033450250000000

quite r utterly 0.04594837644955610000000

quite r rather 0.01709927027208550000000

quite r thoroughly 0.02136222492719840000000

quite r positively 0.00005034996279246730000

quite r exceedingly 0.00132445597948564000000

quite r really 0.06920026237921860000000

quite r very 0.49820245968157300000000

quite r fairly 0.00117189694068352000000

quite r certainly 0.01047286456854480000000

quite r totally 0.02498976546167560000000

quite r assuredly 0.00005034996279246730000

quite r perfectly 0.09148217373503510000000

quite r pretty 0.05124799797945290000000

quite r right 0.00687759567132166000000

quite r entirely 0.01075433027764010000000

quite r verily 0.00005750439126618310000

quite r altogether 0.02486976924699190000000

path n fashion 0.00551783284588815000000

path n lane 0.00551783284588815000000

path n course 0.02419134551744990000000

path n idea 0.28140947514029600000000

path n round 0.00171940616175333000000

path n manner 0.00551783284588815000000

path n street 0.04531645629977590000000

path n track 0.01345383944893840000000

path n boulevard 0.00672691972446922000000

path n avenue 0.00171940616175333000000

path n road 0.04966049561299330000000

path n line 0.00401058634404222000000

path n way 0.54464552348597000000000

path n plan 0.00200529317202111000000

path n pathway 0.00551783284588815000000

path n walk 0.00306992154698504000000

doctor v rebuild 0.07447000811706770000000

doctor v improve 0.01743486148888840000000

doctor v alter 0.10933973109484500000000

doctor v disguise 0.06973944595555400000000

doctor v color 0.01743486148888840000000

doctor v load 0.01743486148888840000000

doctor v correct 0.07683569996989310000000

doctor v change 0.17907917705039900000000

doctor v forge 0.32889162225073200000000

doctor v heal 0.01743486148888840000000

doctor v attend 0.09190486960595610000000

digest v stand 0.25000000000000000000000

digest v bear 0.75000000000000000000000

completely r quite 0.53277040301766800000000

completely r finally 0.01220953582929370000000

completely r fully 0.00783002841226446000000

completely r conclusively 0.00571716360260580000000

completely r truly 0.00080830784633201100000

completely r utterly 0.18009065348208300000000

completely r really 0.00083724153176235500000

completely r perfectly 0.06072689346945790000000

completely r simply 0.00005581610211749000000

completely r entirely 0.19279337268965200000000

completely r altogether 0.00616058401676435000000

debate v discuss 0.99024390243902400000000

debate v ponder 0.00975609756097561000000

daze v amaze 1.00000000000000000000000

culminate v close 1.00000000000000000000000

admire v respect 0.02670557873080220000000

admire v love 0.86676734493524600000000

admire v approve 0.00996022230817592000000

admire v adore 0.02470589529631710000000

admire v value 0.00042261752509278500000

admire v appreciate 0.00084523505018557100000

admire v wonder 0.03895719965880890000000

admire v worship 0.00402050974910229000000

admire v credit 0.00169902994661654000000

admire v enjoy 0.02591636679965230000000

censure v reproach 1.00000000000000000000000

bribe v tempt 0.32234448484798000000000

bribe v approach 0.04386478124775740000000

bribe v reward 0.16456088919933400000000

bribe v buy 0.46922984470492900000000

round a honest 0.02766002769294290000000

round a plain 0.05387568931559320000000

round a close 0.00144436607029252000000

round a complete 0.00216654910543877000000

round a whole 0.58253089072485100000000

round a generous 0.00072218303514625500000

round a near 0.00072218303514625500000

round a large 0.00072218303514625500000

round a solid 0.00072218303514625500000

round a open 0.04492110989258380000000

round a free 0.13676083305569700000000

round a fair 0.06332735285289400000000

round a perfect 0.00144436607029252000000

round a simple 0.08298008307882860000000

artisan n master 1.00000000000000000000000

anger v get 1.00000000000000000000000

yield n surrender 0.33333333333333300000000

yield n profit 0.33333333333333300000000

yield n return 0.33333333333333300000000

further r yet 0.01268187960218630000000

further r farther 0.49447477030637500000000

further r moreover 0.00220390209511201000000

further r abroad 0.00499757891142261000000

further r likewise 0.00021428399334351300000

further r then 0.12085106373885400000000

further r also 0.08455192839641350000000

further r again 0.12241991490052600000000

further r longer 0.00034798827669014300000

further r too 0.04404025774613110000000

further r more 0.05567812427042290000000

further r away 0.05753830776252340000000

uninhabited a empty 1.00000000000000000000000

pardon n grace 0.00035374210750880600000

pardon n acquittal 0.03050737930249540000000

pardon n freedom 0.00017964983937623100000

pardon n mercy 0.38642680449827500000000

pardon n conciliation 0.00017409226813257500000

pardon n forgiveness 0.58235833198421200000000

unacquainted a innocent 0.25000000000000000000000

unacquainted a unknown 0.75000000000000000000000

trash n nonsense 0.95597484276729600000000

trash n light 0.04402515723270440000000

mark n fool 0.00430145510992849000000

mark n rank 0.00025708836236224800000

mark n period 0.00012456759290611100000

mark n feature 0.00166067055802055000000

mark n cipher 0.00025708836236224800000

mark n star 0.01282520353148040000000

mark n name 0.03213563680936560000000

mark n proof 0.03944955609097050000000

mark n importance 0.00136797652739891000000

mark n rule 0.00025393371910102500000

mark n evidence 0.00582393135345596000000

mark n dignity 0.00025708836236224800000

mark n letter 0.03675906820377780000000

mark n quality 0.00024913518581222200000

mark n start 0.04608308895343310000000

mark n value 0.00025708836236224800000

mark n glory 0.00064354285091941100000

mark n end 0.00679787004549170000000

mark n stop 0.00012936612619491500000

mark n point 0.00482244827244167000000

mark n track 0.00025708836236224800000

mark n cut 0.00025708836236224800000

mark n smell 0.16589912023235900000000

mark n hand 0.28011811375051300000000

mark n distinction 0.00025393371910102500000

mark n type 0.00024913518581222200000

mark n peculiarity 0.00025708836236224800000

mark n bullet 0.00012936612619491500000

mark n account 0.00903179945140699000000

mark n character 0.00050622354817447000000

mark n signature 0.00024913518581222200000

mark n leader 0.00025708836236224800000

mark n line 0.00012456759290611100000

mark n honor 0.04639092329271450000000

mark n object 0.00411604267102782000000

mark n figure 0.00038165595526835800000

mark n purpose 0.00187899860886219000000

mark n clue 0.00025708836236224800000

mark n note 0.06735220693194070000000

mark n destination 0.00038165595526835800000

mark n relic 0.00038165595526835800000

mark n ticket 0.00025708836236224800000

mark n dash 0.00370310536232945000000

mark n spot 0.20801264460808000000000

mark n paragraph 0.00025708836236224800000

mark n consequence 0.01357184585392840000000

mark n impression 0.00012456759290611100000

mark n intention 0.00088787950344283000000

fast r soon 0.35869374557696500000000

fast r rapidly 0.04316413092103690000000

fast r deeply 0.05035815274120980000000

fast r wildly 0.02158206546051850000000

fast r speedily 0.07553722911181460000000

fast r quick 0.00103831242765381000000

fast r quickly 0.44962636376080100000000

sovereignty n liberty 0.50000000000000000000000

sovereignty n power 0.50000000000000000000000

dear r dearly 1.00000000000000000000000

somebody n name 0.22449743891899800000000

somebody n nabob 0.00319269472141631000000

somebody n someone 0.74930947989967600000000

somebody n person 0.00553105291571864000000

somebody n personage 0.00307719672115836000000

somebody n soul 0.01439213682303260000000

sentry n sentinel 1.00000000000000000000000

rank n consideration 0.00010198285543021800000

rank n bank 0.06083644774121710000000

rank n place 0.02821310158021190000000

rank n authority 0.05577079373654220000000

rank n importance 0.00010198285543021800000

rank n dignity 0.00010198285543021800000

rank n train 0.00053751916654285600000

rank n family 0.08279943992794250000000

rank n glory 0.00020396571086043600000

rank n reputation 0.32914966590102900000000

rank n state 0.10988652672606000000000

rank n order 0.00063950202197307400000

rank n procession 0.00051453898834339100000

rank n division 0.00010198285543021800000

rank n power 0.00010198285543021800000

rank n position 0.32968718506757200000000

rank n circumstance 0.00020396571086043600000

rank n line 0.00043553631111263900000

rank n blood 0.00030594856629065400000

rank n condition 0.00010198285543021800000

rank n situation 0.00020396571086043600000

resident n inhabitant 1.00000000000000000000000

register v reveal 0.07142857142857120000000

register v betray 0.28571428571428600000000

register v show 0.07142857142857120000000

register v read 0.35714285714285800000000

register v hit 0.14285714285714300000000

register v point 0.07142857142857120000000

bank n rank 0.16584118675665400000000

bank n cashier 0.03986620249458560000000

bank n savings 0.02705381463651390000000

bank n group 0.00027800699089669000000

bank n bar 0.00027800699089669000000

bank n side 0.00282578039490105000000

bank n train 0.00055779163098657600000

bank n course 0.33647788343450100000000

bank n shore 0.01338790382280860000000

bank n treasure 0.01736352234571910000000

bank n edge 0.00027800699089669000000

bank n club 0.01107394513738490000000

bank n rise 0.00027978464008988600000

bank n cellar 0.00452087001185431000000

bank n pile 0.00027800699089669000000

bank n pot 0.00027800699089669000000

bank n line 0.02408014438788510000000

bank n rick 0.00424108537176443000000

bank n house 0.32577581968288900000000

bank n kitty 0.00335386038269225000000

bank n granary 0.02191036991428770000000

purpose v will 1.00000000000000000000000

prohibit v check 1.00000000000000000000000

practise v do 1.00000000000000000000000

further a new 0.05608730745947400000000

further a other 0.04291383601264550000000

further a more 0.90099885652788100000000

pawnbroker n uncle 1.00000000000000000000000

bottle n glass 1.00000000000000000000000

awful a noble 0.00465719073216608000000

awful a ill 0.03084900662952430000000

awful a sick 0.02284429850016910000000

awful a dreadful 0.06487113290933150000000

awful a disgusting 0.02386384775575790000000

awful a grand 0.00363766544806556000000

awful a painful 0.00500547181184913000000

awful a dreaded 0.00149699413966009000000

awful a unfortunate 0.00842196308213598000000

awful a horrible 0.07471453243316690000000

awful a solemn 0.00172263560728467000000

awful a poor 0.01428206414539240000000

awful a sickening 0.00052618081951735800000

awful a fearful 0.04662553501706970000000

awful a wretched 0.04156531123846870000000

awful a disagreeable 0.03491373033718980000000

awful a hideous 0.00208998053973875000000

awful a lofty 0.00205150650744821000000

awful a unpleasant 0.05414680879403900000000

awful a frightful 0.00353607530731798000000

awful a dismal 0.00274175791933661000000

awful a mean 0.00166863331373135000000

awful a tremendous 0.00159092318371719000000

awful a bad 0.05174341318039710000000

awful a inferior 0.00049838502684015200000

awful a alarming 0.00103095350701903000000

awful a prodigious 0.00048238455629950500000

awful a abominable 0.03216057675324970000000

awful a vile 0.01202888946416800000000

awful a distressing 0.00130749434672447000000

awful a shocking 0.00474255162381277000000

awful a nasty 0.00159092318371719000000

awful a pathetic 0.00052618081951735800000

awful a petty 0.00075441266731358700000

awful a terrible 0.22550086697188300000000

awful a contemptible 0.00678668414174749000000

awful a offensive 0.00030461436540612800000

awful a grotesque 0.00159092318371719000000

awful a unwell 0.00075441266731358700000

awful a great 0.07769975208745340000000

awful a appalling 0.00991067698558553000000

awful a serious 0.01789544086106560000000

awful a objectionable 0.10169789380167600000000

awful a base 0.00316932363301424000000

rain n thunderstorm 0.01894136299838850000000

rain n deluge 0.67052425014295400000000

rain n flood 0.30478375006497900000000

rain n squall 0.00575063679367885000000

infidel n heathen 1.00000000000000000000000

anxiety n suffering 0.03105567491634370000000

anxiety n disquiet 0.01085137737529300000000

anxiety n distress 0.00036626091736594400000

anxiety n agitation 0.02912738137578640000000

anxiety n concern 0.02912738137578640000000

anxiety n uneasiness 0.08413728512743550000000

anxiety n apprehension 0.62259777690743500000000

anxiety n perplexity 0.00064879278562713000000

anxiety n trouble 0.00192829354055729000000

anxiety n fear 0.00174031523943378000000

anxiety n misgiving 0.00174031523943378000000

anxiety n pain 0.07599509597151320000000

anxiety n diffidence 0.11068404922798800000000

ill-natured a unfriendly 1.00000000000000000000000

habituate v use 1.00000000000000000000000

pray v crave 0.00679682080024274000000

pray v adjure 0.00046428688560610000000

pray v urge 0.00092857377121220300000

pray v entreat 0.02096484162328400000000

pray v importune 0.00322885847126499000000

pray v contemplate 0.00393784062236286000000

pray v implore 0.00046428688560610000000

pray v plead 0.01302847925823340000000

pray v meditate 0.00542858048741360000000

pray v beseech 0.00369314535687109000000

pray v beg 0.90591273615520900000000

pray v press 0.03515154968269360000000

evolution n development 1.00000000000000000000000

enumerate v detail 0.03355283788021320000000

enumerate v count 0.30197554092191900000000

enumerate v add 0.52006898714330500000000

enumerate v mention 0.14440263405456300000000

enthusiastically r effusively 1.00000000000000000000000

enlist v enter 0.38520408163265300000000

enlist v admit 0.61479591836734700000000

place n charge 0.00543541841947133000000

place n work 0.01533611022467920000000

place n rank 0.00153603245105332000000

place n living 0.00044376664598183300000

place n post 0.00895732639789670000000

place n chambers 0.00027774655497354200000

place n park 0.00193755897203707000000

place n niche 0.00061774664813080700000

place n space 0.01355708036596850000000

place n station 0.00545099544980293000000

place n apartment 0.00109962901716062000000

place n neighborhood 0.00143719440315252000000

place n lane 0.00030108660160997300000

place n section 0.00004976292443275940000

place n home 0.02719533997530750000000

place n progress 0.00013989517125413100000

place n standing 0.00030108660160997300000

place n estate 0.00166786650160441000000

place n task 0.01904781248587760000000

place n pad 0.00004205317557697980000

place n occupation 0.00507581829214146000000

place n bag 0.00678422137481257000000

place n matter 0.02132224742493280000000

place n profession 0.00022371774453547600000

place n concern 0.00045643939774482200000

place n pursuit 0.01224414748839800000000

place n street 0.00853432477532516000000

place n pale 0.00012386741666887400000

place n point 0.00857836841051389000000

place n region 0.00049930682452439300000

place n state 0.00403309479671687000000

place n residence 0.00047143823146824800000

place n employment 0.00095016247705121300000

place n retreat 0.00775827069180366000000

place n division 0.00005552348611744410000

place n lodgings 0.00186905872776467000000

place n appointment 0.00763230844433087000000

place n operation 0.00052187944436898500000

place n step 0.07517554548638120000000

place n alley 0.00006834393055142940000

place n town 0.01512401128463040000000

place n country 0.03451042421727590000000

place n job 0.00044782049924348000000

place n drive 0.00025020164846969100000

place n province 0.00006994758562706520000

place n avenue 0.00005572209267742890000

place n chair 0.03195328793820620000000

place n road 0.01034845364328040000000

place n responsibility 0.00736293375238930000000

place n interest 0.00169005059363781000000

place n position 0.01230028856533670000000

place n use 0.01693690207784120000000

place n abode 0.00019795196459440200000

place n circumstance 0.00566680794465733000000

place n line 0.00176525711768535000000

place n lieu 0.01791465279579340000000

place n exchange 0.00049762924432759400000

place n duty 0.02213566898523910000000

place n calling 0.00063920826877009300000

place n purpose 0.01274198772175450000000

place n means 0.00621951134471854000000

place n footing 0.00114984119008461000000

place n way 0.12837023352258300000000

place n chance 0.01832995113010860000000

place n accommodation 0.00447866319894835000000

place n flat 0.00052927965243776900000

place n gain 0.00136691746734186000000

place n affair 0.01295608916191050000000

place n thing 0.07510538716500490000000

place n distance 0.00678901575549525000000

place n court 0.00363185389106463000000

place n vocation 0.00002801353056418040000

place n condition 0.01336024421602320000000

place n function 0.00066970664657171800000

place n quarter 0.00067550503840527600000

place n scene 0.00055168353979162200000

place n seat 0.04527306378634670000000

place n spot 0.00106296510391866000000

place n house 0.03686524107901980000000

place n office 0.00234559757484902000000

place n headquarters 0.00005572209267742890000

place n room 0.10381544665284000000000

place n berth 0.00004742462685848690000

place n stead 0.00004205317557697980000

place n habitation 0.00006834393055142940000

place n field 0.01313057169973780000000

place n property 0.00359214989833290000000

place n city 0.00894325782424729000000

place n role 0.00454868003602087000000

place n void 0.00016742666164292900000

place n reservation 0.00086737783717529200000

place n situation 0.04514397977397870000000

plain a honest 0.01875043409554070000000

plain a explicit 0.00541546604979301000000

plain a complete 0.00117026470347935000000

plain a dull 0.01444281746378140000000

plain a typical 0.00117026470347935000000

plain a proper 0.00628425381232551000000

plain a visible 0.00204796323108886000000

plain a familiar 0.00567323064856195000000

plain a unqualified 0.00567128279378452000000

plain a regular 0.00284587098346114000000

plain a evident 0.02779451739610330000000

plain a ordinary 0.00632015150261628000000

plain a profound 0.00687744518755464000000

plain a rough 0.00120616239377012000000

plain a severe 0.00417772872102616000000

plain a humble 0.00055924153971579400000

plain a direct 0.22235029366107600000000

plain a ugly 0.00232464547320171000000

plain a distinct 0.07372667631919880000000

plain a modest 0.00417772872102616000000

plain a apparent 0.07645454686040770000000

plain a smooth 0.00511398910884616000000

plain a genuine 0.00190467193935497000000

plain a striking 0.00120616239377012000000

plain a dead 0.02152537874322540000000

plain a positive 0.00234052940695869000000

plain a open 0.00200176857174099000000

plain a general 0.00288176867375191000000

plain a obvious 0.00552140266669542000000

plain a bald 0.00120616239377012000000

plain a frank 0.00190467193935497000000

plain a total 0.00313730537528505000000

plain a conspicuous 0.00117026470347935000000

plain a transparent 0.02949067052767950000000

plain a natural 0.02698997103863300000000

plain a monotonous 0.00120616239377012000000

plain a clear 0.11242556242469100000000

plain a intelligible 0.00511398910884616000000

plain a candid 0.01755397055219020000000

plain a easy 0.09785349067446460000000

plain a common 0.00522768171057044000000

plain a perfect 0.00190467193935497000000

plain a homely 0.00567128279378452000000

plain a simple 0.15603721995531000000000

plain a pronounced 0.00117026470347935000000

edify v school 1.00000000000000000000000

disobey v resist 0.06959751279636810000000

disobey v disregard 0.50891142756440300000000

disobey v revolt 0.42149105963922900000000

weather n blow 0.01446016308942650000000

weather n storm 0.83748863636085700000000

weather n snowstorm 0.03772236457820050000000

weather n stand 0.09428680012009650000000

weather n climate 0.01604203585141920000000

deduce v believe 0.67816650384178700000000

deduce v understand 0.29835502864837700000000

deduce v suppose 0.01956538959152960000000

deduce v presume 0.00391307791830591000000

sick a mad 0.06650151122119840000000

sick a ill 0.01356477315539690000000

sick a weak 0.01352811235065870000000

sick a ailing 0.00932399782357903000000

sick a cruel 0.00796987468463626000000

sick a bored 0.00314856043960988000000

sick a weary 0.00149548082247083000000

sick a rabid 0.01663851335759360000000

sick a tired 0.82859057970790300000000

sick a feeble 0.00014492172619618900000

sick a black 0.00224322123370625000000

sick a invalid 0.00622602435316406000000

sick a crazy 0.00455757491562418000000

sick a disturbed 0.00108736199970752000000

sick a lame 0.00074774041123541300000

sick a unwell 0.00074774041123541300000

sick a pale 0.02191739811540220000000

sick a annoyed 0.00156661327068251000000

connoisseur n judge 1.00000000000000000000000

communicative a demonstrative 0.25000000000000000000000

communicative a open 0.25000000000000000000000

communicative a free 0.25000000000000000000000

communicative a candid 0.25000000000000000000000

charter n contract 0.50000000000000000000000

charter n agreement 0.50000000000000000000000

chaplain n priest 0.28574826248456400000000

chaplain n minister 0.02970810174827440000000

chaplain n clergyman 0.02970810174827440000000

chaplain n father 0.65483553401888700000000

pick v make 0.01092831194549740000000

pick v take 0.58271320480812800000000

pick v improve 0.05008809641686300000000

pick v approve 0.01767815167653990000000

pick v draw 0.05282017440323730000000

pick v get 0.23713035856095300000000

pick v raise 0.00273207798637434000000

pick v start 0.02314230764928850000000

pick v pull 0.00273207798637434000000

pick v recover 0.02003523856674520000000

betoken v show 0.30769230769230800000000

betoken v portend 0.07692307692307680000000

betoken v signify 0.23076923076923100000000

betoken v promise 0.38461538461538500000000

meeting n rendezvous 0.44490309455916700000000

meeting n group 0.07118449512946670000000

meeting n engagement 0.01126336948251050000000

meeting n game 0.00700502343568665000000

meeting n match 0.00026724919330780200000

meeting n interview 0.07118449512946670000000

meeting n struggle 0.01335263432740090000000

meeting n reception 0.00026724919330780200000

meeting n appointment 0.00956780848514337000000

meeting n union 0.00026724919330780200000

meeting n council 0.00026724919330780200000

meeting n duel 0.00026724919330780200000

meeting n body 0.04044573586901520000000

meeting n company 0.16712263533324200000000

meeting n combat 0.07239759150181610000000

meeting n conference 0.00121309637234946000000

meeting n event 0.00026724919330780200000

meeting n gathering 0.07118449512946670000000

meeting n battle 0.01757203008542220000000

alien a strange 0.62500000000000000000000

alien a contrary 0.06249999999999990000000

alien a different 0.31250000000000000000000

forehead n brow 0.57065057366830500000000

forehead n front 0.42934942633169500000000

abolish v suppress 0.33333333333333300000000

abolish v kill 0.66666666666666700000000

trifling a slight 0.05915670232850850000000

trifling a vain 0.05915670232850850000000

trifling a foolish 0.05915670232850850000000

trifling a small 0.76337319068596600000000

trifling a low 0.05915670232850850000000

persuade v have 0.65536760945352100000000

persuade v sell 0.00016832016278799000000

persuade v satisfy 0.00197530109166550000000

persuade v bribe 0.00016832016278799000000

persuade v intimidate 0.00016832016278799000000

persuade v convince 0.01624411542036560000000

persuade v affect 0.00059303913439430100000

persuade v make 0.03140656888434330000000

persuade v rouse 0.00033664032557598000000

persuade v overcome 0.00016832016278799000000

persuade v advise 0.00197530109166550000000

persuade v draw 0.00521716684749623000000

persuade v move 0.00897135017308779000000

persuade v carry 0.00076135929718229100000

persuade v tempt 0.00092967945997028100000

persuade v get 0.01316403495725060000000

persuade v arouse 0.00059303913439430100000

persuade v provoke 0.00033664032557598000000

persuade v drive 0.00016832016278799000000

persuade v prompt 0.00118607826878860000000

persuade v assure 0.23434787209259400000000

persuade v bind 0.00762478887078387000000

persuade v force 0.01812781435740500000000

testimonial n letter 1.00000000000000000000000

either r also 0.00209424738733126000000

either r too 0.99790575261266900000000

contrary n opposite 1.00000000000000000000000

scourge n nuisance 0.00152718387293830000000

scourge n blow 0.00152718387293830000000

scourge n misery 0.00076359193646914700000

scourge n misfortune 0.98854612095296300000000

scourge n trial 0.00076359193646914700000

scourge n cat 0.00076359193646914700000

scourge n burden 0.00076359193646914700000

scourge n curse 0.00076359193646914700000

scourge n woe 0.00076359193646914700000

scourge n threat 0.00076359193646914700000

scourge n trouble 0.00229077580940745000000

scourge n worry 0.00076359193646914700000

ever r always 0.63484855507804700000000

ever r continually 0.01501489764616650000000

ever r frequently 0.02925765760372790000000

ever r forever 0.32038373692763400000000

ever r repeatedly 0.00049515274442523000000

travel v know 0.26129875991986800000000

travel v associate 0.01307138963298670000000

travel v wander 0.00065640380324321200000

travel v walk 0.29680140588524300000000

travel v sail 0.00009177424529881610000

travel v move 0.02073661535734640000000

travel v drive 0.00133943234011701000000

travel v explore 0.00071014978742294300000

travel v go 0.31531374212230500000000

travel v fly 0.07899913868512160000000

travel v cross 0.00497562332673723000000

travel v cover 0.00096466462351688000000

travel v proceed 0.00438300767202230000000

travel v advance 0.00014957589491335800000

travel v visit 0.00050831670385802900000

resigned a willing 0.10000000000000000000000

resigned a satisfied 0.40000000000000000000000

resigned a ready 0.50000000000000000000000

reproduce v bear 1.00000000000000000000000

projection n motion 0.00385356454720617000000

projection n figure 0.99614643545279400000000

prejudice v hurt 0.07884908009406540000000

prejudice v turn 0.92115091990593500000000

pocketbook n purse 0.03695652173913040000000

pocketbook n case 0.50000000000000000000000

pocketbook n pocket 0.46304347826087000000000

suggest v advocate 0.00196819659170289000000

suggest v show 0.00519517724283735000000

suggest v advise 0.01229126818907580000000

suggest v recommend 0.82546165056019400000000

suggest v hint 0.06160161571344730000000

suggest v move 0.00842215789397180000000

suggest v mention 0.00787278636681158000000

suggest v imply 0.00322698065113445000000

suggest v offer 0.06356981230515020000000

suggest v steer 0.00196819659170289000000

suggest v infer 0.00322698065113445000000

suggest v advance 0.00196819659170289000000

suggest v propose 0.00322698065113445000000

overdo v tire 1.00000000000000000000000

neutral a undecided 1.00000000000000000000000

loss n suppression 0.00984177829189844000000

loss n misfortune 0.01341399367459930000000

loss n harm 0.44703011266141300000000

loss n accident 0.00045529853742239800000

loss n debt 0.00072289770164419600000

loss n wound 0.00261867198227903000000

loss n end 0.01650448070745800000000

loss n dead 0.00091059707484479800000

loss n victim 0.00072289770164419600000

loss n sacrifice 0.00045529853742239800000

loss n death 0.01626751075443680000000

loss n impoverishment 0.00045529853742239800000

loss n prejudice 0.00072289770164419600000

loss n failure 0.01251991311291170000000

loss n lack 0.00045529853742239800000

loss n catastrophe 0.15159164803478800000000

loss n trouble 0.05255645643119080000000

loss n calamity 0.15329972294222200000000

loss n going 0.00072289770164419600000

loss n disadvantage 0.00045529853742239800000

loss n theft 0.00045529853742239800000

loss n need 0.11782173230084600000000

mutilate v change 1.00000000000000000000000

misplace v disturb 0.22727272727272700000000

misplace v lose 0.36363636363636400000000

misplace v confuse 0.04545454545454520000000

misplace v remove 0.04545454545454520000000

misplace v miss 0.31818181818181800000000

guardian n nurse 0.24491802394240200000000

guardian n overseer 0.02400376526525820000000

guardian n trustee 0.00242596996899214000000

guardian n guard 0.11953298214570100000000

guardian n steward 0.29714549052983500000000

guardian n patron 0.11568116192073200000000

guardian n protector 0.00385182022496870000000

guardian n keeper 0.02785558549022690000000

guardian n sentinel 0.16458520051188300000000

leak n fault 1.00000000000000000000000

enough a complete 0.04133670859898580000000

enough a comfortable 0.61638281028227000000000

enough a sufficient 0.21398897091987600000000

enough a full 0.05545282336434010000000

enough a decent 0.00740220281355464000000

enough a satisfactory 0.05659238303034820000000

enough a acceptable 0.00884410099062545000000

invincible a formidable 1.00000000000000000000000

direct v keep 0.01355094741891860000000

direct v manage 0.00212170073748366000000

direct v charge 0.00009620552940879350000

direct v deliver 0.00009620552940879350000

direct v mean 0.02305297623089090000000

direct v lay 0.02502498230981550000000

direct v run 0.02584531885503540000000

direct v take 0.00869583197883647000000

direct v set 0.00303824281211231000000

direct v warn 0.00019241105881758800000

direct v bid 0.00173897557780097000000

direct v show 0.00080651935237784100000

direct v send 0.00144912013022436000000

direct v advise 0.12551577085580500000000

direct v read 0.02198722055000540000000

direct v dispose 0.00159240612997109000000

direct v tell 0.17165546738843300000000

direct v beam 0.00405760968153561000000

direct v drive 0.02512118783922430000000

direct v aim 0.00009620552940879350000

direct v place 0.00125670907140678000000

direct v explain 0.00906107632505282000000

direct v dictate 0.00296331399176586000000

direct v address 0.00482967926628685000000

direct v contrive 0.00101274760403744000000

direct v fix 0.32934985019389900000000

direct v point 0.00110895313344623000000

direct v require 0.00138617787831150000000

direct v ordain 0.00009620552940879350000

direct v turn 0.00415381521094440000000

direct v see 0.16904676936746000000000

direct v inform 0.00148165699588293000000

direct v order 0.00173897557780097000000

direct v accompany 0.01677876435878240000000

husky a thick 1.00000000000000000000000

surely r absolutely 0.00596594060915349000000

surely r clearly 0.00550785597092072000000

surely r indeed 0.33992789605949400000000

surely r positively 0.01904043941842000000000

surely r precisely 0.00207913293605717000000

surely r sure 0.00831599261933767000000

surely r certainly 0.59846869943758500000000

surely r decidedly 0.00501838075555991000000

surely r assuredly 0.00463086342001484000000

surely r evidently 0.01104479877345730000000

hallucination n fancy 0.11905354919053500000000

hallucination n misapprehension 0.05952677459526770000000

hallucination n ghost 0.05952677459526770000000

hallucination n chimera 0.07737650477376470000000

hallucination n vision 0.05952677459526770000000

hallucination n dream 0.62498962224989700000000

guarantee n promise 0.72527472527472500000000

guarantee n plight 0.27472527472527500000000

goad v encourage 0.25000000000000000000000

goad v torment 0.75000000000000000000000

instance n request 0.00060564184207432000000

instance n example 0.69133568225333600000000

instance n case 0.03560807066932660000000

instance n point 0.07586888985002050000000

instance n type 0.00121128368414864000000

instance n persuasion 0.00060564184207432000000

instance n occasion 0.16554037662443300000000

instance n sample 0.02922441323458640000000

gaoler n jailer 1.00000000000000000000000

foggy a dark 1.00000000000000000000000

firstly r first 1.00000000000000000000000

finished a satisfied 0.50000000000000000000000

finished a smooth 0.50000000000000000000000

emblem n figure 1.00000000000000000000000

gun n pistol 0.51590855361973900000000

gun n cannon 0.11334788644265800000000

gun n howitzer 0.36838063093863800000000

gun n musket 0.00047258579979299300000

gun n piece 0.00189034319917197000000

dryly r drily 1.00000000000000000000000

drily r dryly 1.00000000000000000000000

discourse v talk 1.00000000000000000000000

know v prize 0.00004737332105125220000

know v associate 0.00025339445565714800000

know v realize 0.01677608665391680000000

know v savor 0.00012128784889660300000

know v understand 0.20922629568168100000000

know v recollect 0.01071734641788500000000

know v acknowledge 0.00428265098854788000000

know v live 0.03502171035906770000000

know v love 0.07383478406768670000000

know v distinguish 0.00052770703126565400000

know v experience 0.00431181622213441000000

know v comprehend 0.00848091143638894000000

know v remember 0.09088793933014750000000

know v receive 0.01120659653384460000000

know v read 0.02304558423221820000000

know v grasp 0.00220759695797911000000

know v penetrate 0.00008603883157200760000

know v twig 0.00004182339617124250000

know v undergo 0.00243062254524637000000

know v sustain 0.00023520681370589200000

know v taste 0.00241915403996941000000

know v appreciate 0.00341194382242017000000

know v discern 0.00013464741653407500000

know v notice 0.02274344174772190000000

know v learn 0.01055656336483810000000

know v identify 0.00007403731402114030000

know v feel 0.07987033139158040000000

know v apprehend 0.00173041186321022000000

know v recognize 0.01352182463941120000000

know v perceive 0.01315135162815070000000

know v possess 0.00317139280624008000000

know v see 0.35126235060724900000000

know v recall 0.00404767679401487000000

know v bang 0.00002255469063628130000

know v fathom 0.00013954474893859300000

keep v have 0.39953027889266500000000

keep v stand 0.08493120200782810000000

keep v prosecute 0.00000608181233251114000

keep v restrict 0.00002896909035922180000

keep v direct 0.00088399537129991500000

keep v house 0.00057855411888845600000

keep v manage 0.00089219868150914000000

keep v own 0.01856040678904860000000

keep v respect 0.00112988969230528000000

keep v bury 0.00241242073999956000000

keep v support 0.00068572690567111500000

keep v endure 0.00169759254328968000000

keep v preserve 0.00017418914246170000000

keep v deny 0.00108185286738658000000

keep v embrace 0.00128072101728453000000

keep v last 0.04330253105247040000000

keep v check 0.00008182149111012390000

keep v hold 0.00553451662112134000000

keep v acknowledge 0.00014271239836291600000

keep v afford 0.00034794333769717500000

keep v run 0.05559983214117520000000

keep v stick 0.00013096943542986200000

keep v obey 0.00135514876589336000000

keep v maintain 0.00322131735579518000000

keep v secure 0.00015961034301145400000

keep v conduct 0.00002896909035922180000

keep v follow 0.01414084278596050000000

keep v consummate 0.00034128192406341700000

keep v protect 0.00088019199018795500000

keep v remain 0.04434803245841990000000

keep v furnish 0.00004791736936211890000

keep v honor 0.00275017975090766000000

keep v clasp 0.00096425686481409400000

keep v interdict 0.00000608181233251114000

keep v plug 0.01349959610739730000000

keep v forbid 0.00151459793098577000000

keep v complete 0.00035689450187494700000

keep v restrain 0.00086481596242431900000

keep v grasp 0.00008044651660885940000

keep v defend 0.00051881644434086000000

keep v stop 0.02676661502063320000000

keep v persist 0.00044472832742324000000

keep v arrest 0.00103159064645282000000

keep v accomplish 0.00067465336800173400000

keep v guard 0.00043190945900431400000

keep v haunt 0.00124907901372409000000

keep v suppress 0.00004020042086005280000

keep v praise 0.00025871548795264100000

keep v feed 0.00046173947796542000000

keep v carry 0.01337592267888090000000

keep v execute 0.00129140176054647000000

keep v continue 0.00381691071449294000000

keep v disguise 0.00013519060996593800000

keep v tend 0.00073510077984912700000

keep v toil 0.00005428792000293830000

keep v detain 0.01100086232996710000000

keep v hallow 0.00001894827900289710000

keep v observe 0.00120206544904023000000

keep v delay 0.00033113054551465900000

keep v reserve 0.00001255192571585060000

keep v raise 0.02762168898773420000000

keep v control 0.00089077407313590000000

keep v heed 0.00468603193394154000000

keep v stifle 0.00010220799020770500000

keep v hinder 0.00049989627194771100000

keep v limit 0.00007650287946977570000

keep v effect 0.00005793818071844360000

keep v stay 0.03835495866483660000000

keep v practice 0.00006686564836501600000

keep v hunt 0.00101674441529074000000

keep v place 0.00084728815523814000000

keep v repress 0.00055168646423244100000

keep v abide 0.00029164102423090400000

keep v seek 0.01058204381033590000000

keep v administer 0.00020854888450621800000

keep v achieve 0.00006332883240374050000

keep v save 0.00329342111200059000000

keep v master 0.00395264430538988000000

keep v hush 0.00053998384429589300000

keep v breed 0.00025396235881584400000

keep v grind 0.00809975766443839000000

keep v hide 0.01350626019949820000000

keep v court 0.00000608181233251114000

keep v celebrate 0.00002896909035922180000

keep v finish 0.00230021837384194000000

keep v rear 0.00025223079309979900000

keep v avert 0.00118522303970396000000

keep v persevere 0.00045691328104790000000

keep v brace 0.00073634160585803800000

keep v mind 0.00949322766076792000000

keep v regard 0.00059235949085727000000

keep v cover 0.01591599638193620000000

keep v proceed 0.00229838827122218000000

keep v uphold 0.00004392493310432960000

keep v possess 0.00095155299419951000000

keep v silence 0.00002471555038087290000

keep v ordain 0.00020179860011861700000

keep v operate 0.00003411860852754170000

keep v encumber 0.00002896909035922180000

keep v attend 0.00394242201961387000000

keep v track 0.00016199394904497200000

keep v stall 0.00018924667440276600000

keep v mask 0.00034321007052705100000

keep v pursue 0.00064420187804564000000

keep v prevent 0.02860077912186800000000

keep v bless 0.01070007835666610000000

keep v dominate 0.00040097810219992000000

keep v put 0.03060092496947450000000

keep v stow 0.00002896909035922180000

keep v conceal 0.00358610741876816000000

keep v enjoy 0.00076218269575998900000

keep v accompany 0.00053776495848462600000

keep v perform 0.00093724626561274200000

keep v board 0.00034128192406341700000

keep v nourish 0.00026820389617345700000

keep v cramp 0.00008653587248331610000

keep v hamper 0.00030671083926617600000

keep v deter 0.00001894827900289710000

gold n treasure 0.20205948385336400000000

gold n money 0.64544934488016200000000

gold n wealth 0.01649807463715120000000

gold n fortune 0.13599309662932300000000

discontented a miserable 0.30679156908665100000000

discontented a unhappy 0.30679156908665100000000

discontented a resentful 0.38641686182669800000000

deceitful a false 0.73649754500818300000000

deceitful a artful 0.00818330605564648000000

deceitful a sly 0.25531914893617000000000

generally r always 0.90134954310824200000000

generally r often 0.08190861236294290000000

generally r mostly 0.01674184452881480000000

conciliate v reconcile 1.00000000000000000000000

conceivable a credible 0.06249999999999990000000

conceivable a possible 0.90625000000000000000000

conceivable a likely 0.03124999999999980000000

comprehensive a complete 1.00000000000000000000000

thin a weak 0.46654462788950800000000

thin a low 0.05327589551756210000000

thin a fine 0.05327589551756210000000

thin a superficial 0.21345179053768400000000

thin a haggard 0.21345179053768400000000

chaste a innocent 0.01107682569823560000000

chaste a nice 0.01107682569823560000000

chaste a elegant 0.69151040430413800000000

chaste a good 0.28633594429939100000000

broach v approach 1.00000000000000000000000

lot n group 0.04216498859421120000000

lot n race 0.01123314367953890000000

lot n article 0.15696817780006000000000

lot n part 0.01433194193596340000000

lot n cup 0.00192546936664499000000

lot n estate 0.00069370152908931100000

lot n heap 0.00069370152908931100000

lot n family 0.01177906918859970000000

lot n sort 0.06718426315318420000000

lot n ballot 0.00069370152908931100000

lot n end 0.00069370152908931100000

lot n portion 0.00777249523468682000000

lot n luck 0.03828137122369180000000

lot n crew 0.00357418207985328000000

lot n host 0.00192546936664499000000

lot n plenty 0.04216498859421120000000

lot n state 0.01615574562410460000000

lot n order 0.00192546936664499000000

lot n string 0.10390657903573500000000

lot n sight 0.01433194193596340000000

lot n pot 0.03548029528049480000000

lot n interest 0.01433194193596340000000

lot n stable 0.01415758741119500000000

lot n body 0.14824318837446700000000

lot n packet 0.00069370152908931100000

lot n load 0.00069370152908931100000

lot n company 0.00192546936664499000000

lot n party 0.04393665789081300000000

lot n bunch 0.00069370152908931100000

lot n chance 0.02008963073175290000000

lot n piece 0.00192546936664499000000

lot n mob 0.00192546936664499000000

lot n blood 0.00777249523468682000000

lot n fortune 0.00496158513803191000000

lot n number 0.04643287220315380000000

lot n mess 0.00900738146439806000000

lot n army 0.00069370152908931100000

lot n condition 0.05016179677587200000000

lot n crowd 0.04216498859421120000000

lot n field 0.00138740305817862000000

lot n property 0.00714836415970657000000

lot n situation 0.00777249523468682000000

bid n attempt 0.20000000000000000000000

bid n offer 0.20000000000000000000000

bid n advance 0.60000000000000000000000

aristocrat n nobleman 1.00000000000000000000000

guess v suggest 0.00006371504190716980000

guess v suspect 0.00469653342368527000000

guess v reckon 0.06783361735106590000000

guess v venture 0.04517609870880240000000

guess v believe 0.14187341713905200000000

guess v pretend 0.00007353264349860530000

guess v calculate 0.00101428724800752000000

guess v think 0.41516815567281600000000

guess v suppose 0.01230817363080710000000

guess v expect 0.08507774572841330000000

guess v select 0.00143667142881006000000

guess v allow 0.00696914255488733000000

guess v presume 0.00006690557980938170000

guess v divine 0.00145351309415403000000

guess v solve 0.00171708199684286000000

guess v daresay 0.00316722159624690000000

guess v feel 0.05173345259988560000000

guess v imagine 0.15351182123235900000000

guess v infer 0.00175418524568055000000

guess v speculate 0.00002083756127251010000

guess v predict 0.00003185752095358490000

guess v fancy 0.00155238061959821000000

guess v fathom 0.00115056948114460000000

guess v judge 0.00214908290030075000000

allot v give 1.00000000000000000000000

ajar a open 1.00000000000000000000000

airing n drive 0.98921319796954300000000

airing n walk 0.01078680203045680000000

experience n meeting 0.00508307619695393000000

experience n proof 0.02880712623778390000000

experience n observation 0.00110632798392611000000

experience n reality 0.00610993593994054000000

experience n trial 0.00087135796736422700000

experience n struggle 0.00110632798392611000000

experience n contact 0.00182872981133522000000

experience n adventure 0.01465079814467990000000

experience n incident 0.01465079814467990000000

experience n case 0.08896811178562200000000

experience n practice 0.01465079814467990000000

experience n wisdom 0.12022722225345100000000

experience n circumstance 0.00293505779526133000000

experience n sense 0.17139046371977500000000

experience n transaction 0.00091436490566761200000

experience n learning 0.00019196307825849500000

experience n trip 0.05557748426623140000000

experience n affair 0.01515920958214030000000

experience n event 0.00938627166405370000000

experience n acquaintance 0.05740621407756670000000

experience n action 0.00178572287303184000000

experience n knowledge 0.00789245788623032000000

experience n life 0.23909933018092000000000

experience n history 0.00110632798392611000000

experience n habit 0.00421171822958970000000

experience n impression 0.12782645956673600000000

experience n existence 0.00705634359626749000000

comply v satisfy 0.01742689009859280000000

comply v keep 0.18684710754528400000000

comply v obey 0.08767843587496790000000

comply v follow 0.05545659886514800000000

comply v meet 0.00580896336619759000000

comply v submit 0.02429486019106770000000

comply v approve 0.01161792673239520000000

comply v consent 0.04172166917701290000000

comply v discharge 0.01848589682487010000000

comply v observe 0.19153443099101500000000

comply v assent 0.19153443099101500000000

comply v agree 0.02904481683098800000000

comply v mind 0.09576721549550750000000

comply v acquiesce 0.00580896336619759000000

comply v perform 0.01848589682487010000000

comply v accord 0.01848589682487010000000

unendurable a intolerable 0.24728924317481400000000

unendurable a unbearable 0.57776528389474800000000

unendurable a insufferable 0.03617188512218760000000

unendurable a insupportable 0.13877358780825100000000

demand v direct 0.00008131574919915260000

demand v charge 0.00004102174629877170000

demand v cry 0.24646812511869800000000

demand v besiege 0.00116637277757535000000

demand v apply 0.00034125678498339200000

demand v insist 0.00261577496277536000000

demand v adjure 0.00001358221575051340000

demand v take 0.01336819321138420000000

demand v urge 0.00994335918829270000000

demand v question 0.00143182842169929000000

demand v entreat 0.00382750728509410000000

demand v bid 0.00024394724759745800000

demand v impose 0.00018608796451344500000

demand v request 0.00008131574919915260000

demand v expect 0.00944146873116280000000

demand v implore 0.00194889704669035000000

demand v appeal 0.00001358221575051340000

demand v beseech 0.00005460396204928520000

demand v need 0.00065297765257589000000

demand v seek 0.02926114703283960000000

demand v beg 0.00026395292212691100000

demand v inquire 0.13409241669397500000000

demand v ask 0.44716429552855300000000

demand v require 0.02953157915517940000000

demand v press 0.00004074664725154040000

demand v force 0.00127177860538605000000

demand v want 0.05949616919756700000000

demand v order 0.00695669618583201000000

uncouth a bold 1.00000000000000000000000

thoughtlessness n stupidity 1.00000000000000000000000

bottom n heart 0.20303860716529200000000

bottom n principle 0.01190735391935800000000

bottom n foot 0.69940210885689000000000

bottom n bed 0.02548012418906920000000

bottom n fanny 0.04676338773249940000000

bottom n cause 0.00686200785158048000000

bottom n ass 0.00654641028531078000000

thirst v want 0.50000000000000000000000

thirst v fancy 0.50000000000000000000000

temperate a refreshing 0.01408450704225350000000

temperate a nice 0.11267605633802800000000

temperate a dry 0.01408450704225350000000

temperate a severe 0.04225352112676060000000

temperate a reasonable 0.01408450704225350000000

temperate a cool 0.01408450704225350000000

temperate a modest 0.04225352112676060000000

temperate a mild 0.01408450704225350000000

temperate a good 0.42253521126760600000000

temperate a quiet 0.08450704225352110000000

temperate a moderate 0.02816901408450700000000

temperate a clear 0.02816901408450700000000

temperate a pleasant 0.07042253521126760000000

temperate a agreeable 0.08450704225352110000000

temperate a warm 0.01408450704225350000000

slight a trifling 0.00659140130857931000000

slight a weak 0.01318280261715860000000

slight a precarious 0.00659140130857931000000

slight a immaterial 0.00659140130857931000000

slight a narrow 0.00659140130857931000000

slight a delicate 0.00659140130857931000000

slight a faint 0.20804110380203500000000

slight a poor 0.43952662362208500000000

slight a foolish 0.01318280261715860000000

slight a feeble 0.00659140130857931000000

slight a silly 0.06361569552424910000000

slight a modest 0.01977420392573790000000

slight a little 0.02009953059976650000000

slight a small 0.03816130456210320000000

slight a short 0.06683248579062310000000

slight a tolerable 0.01318280261715860000000

slight a fine 0.03816130456210320000000

slight a moderate 0.01318280261715860000000

slight a scanty 0.00230557599420263000000

slight a deficient 0.00889697730278194000000

slight a light 0.00230557599420263000000

squeal v talk 0.86046511627907000000000

squeal v tell 0.09302325581395350000000

squeal v shout 0.04651162790697670000000

child n lass 0.00186699727550775000000

child n lad 0.04949168347880170000000

child n baby 0.08588383890322900000000

child n imp 0.00018632360846536300000

child n missy 0.00008036357580164370000

child n girl 0.29108969668694600000000

child n boy 0.27368224864396000000000

child n son 0.08263663760626790000000

child n daughter 0.15443929855480600000000

child n shaver 0.00018632360846536300000

child n infant 0.00861120007312503000000

child n heir 0.00044601440810119800000

child n issue 0.00074573718821790500000

child n cherub 0.00161224468461496000000

child n babe 0.00171683409018481000000

child n junior 0.00054701831002137900000

child n heiress 0.00097268797050652600000

child n brat 0.00132133828944240000000

child n kid 0.01561488630424670000000

child n kitten 0.00031004286885688300000

child n miss 0.00042606421538012200000

child n descendant 0.00007738267728489840000

child n urchin 0.00029820095372693200000

child n puppy 0.01296143500254570000000

child n youth 0.01479550102149410000000

sexton n servant 1.00000000000000000000000

stretch v spread 0.68270939752291600000000

stretch v open 0.29683017283605100000000

stretch v draw 0.01763347561402280000000

stretch v give 0.00282695402701001000000

revisit v stay 1.00000000000000000000000

revengeful a bitter 1.00000000000000000000000

ring v call 0.21394598951358900000000

ring v announce 0.00758048216080129000000

ring v sound 0.21929251965175200000000

ring v tinkle 0.13157551179105100000000

ring v toll 0.25190525334355100000000

ring v honor 0.00009515249574227510000

ring v confine 0.02558412729270440000000

ring v echo 0.00009515249574227510000

ring v strike 0.14155199479839400000000

ring v play 0.00114979549665885000000

ring v yell 0.00009576092561211830000

ring v pull 0.00009576092561211830000

ring v bang 0.00009515249574227510000

ring v trumpet 0.00009576092561211830000

ring v beat 0.00684158568743628000000

recompense v satisfy 1.00000000000000000000000

prophesy v guess 1.00000000000000000000000

quit v withdraw 0.00506600404311644000000

quit v depart 0.00026208354249903200000

quit v abandon 0.00026208354249903200000

quit v desert 0.11127771805951500000000

quit v abdicate 0.00026208354249903200000

quit v submit 0.00026208354249903200000

quit v flee 0.01230323022404340000000

quit v stop 0.01448505092086520000000

quit v release 0.00838344890174209000000

quit v halt 0.00026208354249903200000

quit v resign 0.00162522212234410000000

quit v lessen 0.00052416708499806500000

quit v drop 0.00445052146467983000000

quit v forsake 0.00026208354249903200000

quit v surrender 0.00090445622954994500000

quit v leave 0.57949480393871400000000

quit v rest 0.01947689745368500000000

quit v remove 0.00221487394204511000000

quit v end 0.10560554260598400000000

quit v cease 0.12003426246455700000000

quit v pause 0.00454183695811837000000

quit v abate 0.00201623769125039000000

quit v vacate 0.00602322463979856000000

playmate n comrade 1.00000000000000000000000

petite a little 1.00000000000000000000000

murmur v mutter 0.07225097467793130000000

murmur v sigh 0.39516943864644600000000

murmur v moan 0.16990641606157900000000

murmur v stammer 0.13998484367551300000000

murmur v whisper 0.21935682858438200000000

murmur v mumble 0.00333149835414860000000

parricide n murderer 0.96918542336548800000000

parricide n traitor 0.03081457663451220000000

mistake n confusion 0.01565710367598350000000

mistake n misapprehension 0.00079442815027403200000

mistake n misconception 0.00446626228183551000000

mistake n fall 0.00261311355543194000000

mistake n error 0.00365414789345014000000

mistake n perplexity 0.00181868540515791000000

mistake n failure 0.00500172871773096000000

mistake n slip 0.01871815540589490000000

mistake n trip 0.00313142073519670000000

mistake n break 0.00181868540515791000000

mistake n delusion 0.00181868540515791000000

mistake n blunder 0.13187791308698700000000

mistake n illusion 0.00446626228183551000000

mistake n misunderstanding 0.00079442815027403200000

mistake n fault 0.19919447272939400000000

mistake n impression 0.60417450712023800000000

natural n simpleton 1.00000000000000000000000

naively r ingenuously 1.00000000000000000000000

muddle n difficulty 1.00000000000000000000000

promise n guarantee 0.01714010491338310000000

promise n betrothal 0.00334767674089513000000

promise n possibility 0.00159368711421507000000

promise n understanding 0.00239053067132260000000

promise n profession 0.00159368711421507000000

promise n consent 0.05784741282716590000000

promise n oath 0.11686435168215700000000

promise n talent 0.00079684355710753000000

promise n hope 0.06642834017663340000000

promise n signature 0.00038609637137820300000

promise n pledge 0.11686435168215700000000

promise n agreement 0.01987836133832490000000

promise n sign 0.15366713908768100000000

promise n word 0.41922604144316400000000

promise n avowal 0.01836439812148190000000

promise n compact 0.00361097715871834000000

lunch v dine 1.00000000000000000000000

interrogation n question 1.00000000000000000000000

soft a weak 0.05140188553954110000000

soft a kind 0.12396925336007000000000

soft a untroubled 0.01414414300081330000000

soft a delightful 0.05304963949350610000000

soft a affectionate 0.00164775395396496000000

soft a low 0.00329550790792992000000

soft a fine 0.56904549048659200000000

soft a sunny 0.05545992913476800000000

soft a pale 0.05140188553954110000000

soft a light 0.01414414300081330000000

soft a agreeable 0.00164775395396496000000

soft a warm 0.06079261462849570000000

imperative a necessary 0.50000000000000000000000

imperative a important 0.50000000000000000000000

night n bedtime 0.00087927560052103300000

night n dark 0.01100569716829360000000

night n eventide 0.00045296015784416900000

night n evening 0.68219583569445300000000

night n midnight 0.04589473809314730000000

night n gloom 0.00278982493194365000000

night n twilight 0.03403554141977300000000

night n sunset 0.00288180012856294000000

night n obscurity 0.00151912001741309000000

night n nightfall 0.00006285255831926870000

night n darkness 0.21828235422972900000000

spring v come 0.26771293350092900000000

spring v jump 0.03922595190142630000000

spring v shoot 0.00245991948780665000000

spring v reveal 0.00172775234755887000000

spring v rise 0.20093727962143500000000

spring v grow 0.15607362872948500000000

spring v start 0.31938249246294600000000

spring v arrive 0.00482701944524179000000

spring v form 0.00073216714024777800000

spring v fly 0.00482701944524179000000

spring v begin 0.00036608357012388900000

spring v descend 0.00172775234755887000000

heathen n infidel 1.00000000000000000000000

harem n stable 1.00000000000000000000000

hapless a poor 1.00000000000000000000000

goblin n imp 0.01974454728781030000000

goblin n ghost 0.28118965051030300000000

goblin n devil 0.67932125491407600000000

goblin n ogre 0.01974454728781030000000

retire v withdraw 0.26975528575326500000000

retire v part 0.10790211430130600000000

retire v depart 0.26305126583370100000000

retire v separate 0.00100327395910093000000

retire v retreat 0.10790211430130600000000

retire v flee 0.03480713364558260000000

retire v leave 0.20930391469031900000000

retire v recover 0.00627489751541926000000

forestall v forbid 0.04837342247414160000000

forestall v foresee 0.16130126202068700000000

forestall v anticipate 0.64520504808274600000000

forestall v divert 0.04837342247414160000000

forestall v avoid 0.09674684494828360000000

fleeting a evanescent 0.25000000000000000000000

fleeting a short 0.75000000000000000000000

finger v make 1.00000000000000000000000

possibly r perhaps 0.90009060400150400000000

possibly r maybe 0.01244092059522380000000

possibly r probably 0.08746847540327240000000

erase v cut 1.00000000000000000000000

living n work 0.45586464221241900000000

living n conduct 0.00820710559341839000000

living n support 0.00487489730736882000000

living n profession 0.00805417816000066000000

living n life 0.52260971203727200000000

living n being 0.00038946468952097900000

disengaged a free 1.00000000000000000000000

despondent a miserable 0.33333333333333400000000

despondent a broken 0.11111111111111100000000

despondent a hopeless 0.11111111111111100000000

despondent a distressed 0.22222222222222200000000

despondent a sorrowful 0.11111111111111100000000

despondent a depressed 0.11111111111111100000000

lad n boy 0.24806252523767400000000

lad n son 0.02567098010882830000000

lad n chap 0.00009460760175378140000

lad n master 0.18251630080456100000000

lad n fellow 0.50157739294635200000000

lad n schoolboy 0.00204542863661829000000

lad n gamin 0.00018921520350756300000

lad n brat 0.00220230396018900000000

lad n feller 0.02841036706990720000000

lad n youth 0.00923087843060816000000

dashing a airy 1.00000000000000000000000

somewhat r pretty 1.00000000000000000000000

mutter v murmur 0.27485138286611200000000

mutter v groan 0.03543924949195910000000

mutter v grumble 0.04336496024095960000000

mutter v growl 0.64634440740096900000000

commoner n peasant 1.00000000000000000000000

excellent a unique 0.00010291263104497100000

excellent a priceless 0.00296471075059464000000

excellent a extraordinary 0.00026624112900032000000

excellent a wonderful 0.00120748136311825000000

excellent a worthy 0.02594053559256360000000

excellent a sharp 0.00018706907271357700000

excellent a splendid 0.00334097603097831000000

excellent a superior 0.00018706907271357700000

excellent a remarkable 0.00212415155592576000000

excellent a cool 0.00007917205628674280000

excellent a magnificent 0.00028499731837668500000

excellent a rare 0.16621087110601300000000

excellent a exquisite 0.00030873789313491300000

excellent a best 0.02657718377899880000000

excellent a admirable 0.01788784021189570000000

excellent a sublime 0.00020582526208994200000

excellent a bad 0.00772558029186966000000

excellent a fine 0.05543025984602860000000

excellent a attractive 0.00018706907271357700000

excellent a divine 0.02075297525416250000000

excellent a good 0.42535922256349700000000

excellent a first-rate 0.00020582526208994200000

excellent a accomplished 0.00345882920902708000000

excellent a foremost 0.00040792088951670700000

excellent a heavenly 0.00328760004026336000000

excellent a great 0.01134173287732640000000

excellent a perfect 0.00028499731837668500000

excellent a marvelous 0.00010291263104497100000

excellent a invaluable 0.00185791042692544000000

excellent a first 0.20049634784645700000000

excellent a estimable 0.02075297525416250000000

excellent a hot 0.00047206639109026200000

buckle n pin 1.00000000000000000000000

brute n tartar 0.11111111111111100000000

brute n animal 0.05555555555555540000000

brute n monster 0.16666666666666700000000

brute n beast 0.05555555555555540000000

brute n savage 0.05555555555555540000000

brute n devil 0.50000000000000100000000

brute n ogre 0.05555555555555540000000

push v persuade 0.00024932414380787200000

push v demand 0.27752658749499000000000

push v advocate 0.00013195433743575100000

push v fight 0.00043543090986469600000

push v support 0.00021826383388078200000

push v depart 0.01170158084739040000000

push v make 0.12241350995613300000000

push v hasten 0.00008630949644503100000

push v struggle 0.00008630949644503100000

push v rouse 0.00029817043330766600000

push v stick 0.00008576111749659660000

push v urge 0.00017261899289006300000

push v instigate 0.00013195433743575100000

push v send 0.00548749677426854000000

push v agitate 0.00305124672379474000000

push v encourage 0.00039033444782241000000

push v trouble 0.00008576111749659660000

push v persist 0.00008310804793595700000

push v move 0.05235535534127970000000

push v strive 0.00081514044371048100000

push v work 0.00095881645301972700000

push v squeeze 0.00025892848933509400000

push v provoke 0.00033773833086510800000

push v strain 0.00013195433743575100000

push v drive 0.00453821241800675000000

push v start 0.00038978606887397600000

push v beg 0.00008630949644503100000

push v impel 0.09223608186759010000000

push v depress 0.00016886916543255400000

push v leave 0.15353783123706300000000

push v oblige 0.00013195433743575100000

push v persevere 0.00021506238537170800000

push v plague 0.00137121485722566000000

push v press 0.22175747930898700000000

push v advance 0.00038768137826177100000

push v force 0.00051643426718844800000

push v stir 0.00266919149277782000000

push v hurry 0.04415499782907320000000

push v budge 0.00034523798578012500000

avaricious a close 0.08629071463296050000000

avaricious a stingy 0.15685464268352000000000

avaricious a selfish 0.40000000000000000000000

avaricious a mean 0.20000000000000000000000

avaricious a mercenary 0.15685464268352000000000

authorize v allow 1.00000000000000000000000

brow n forehead 0.50422045861364600000000

brow n temple 0.21667858448489000000000

brow n air 0.00904608893918180000000

brow n summit 0.00881831448485018000000

brow n appearance 0.00011388722716580700000

brow n head 0.26100877902310100000000

brow n top 0.00011388722716580700000

adventurer n scoundrel 0.50000000000000000000000

adventurer n thief 0.50000000000000000000000

much r generally 0.00162367159775475000000

much r greatly 0.04846668908897020000000

much r extremely 0.01593852101009940000000

much r around 0.00020971738171183500000

much r almost 0.00079994911401724700000

much r highly 0.01593852101009940000000

much r freely 0.00956311260605968000000

much r usually 0.00004800759340391400000

much r enough 0.01350968686969030000000

much r exceedingly 0.02231392941413930000000

much r frequently 0.00033911746829998800000

much r closely 0.00040868002589998600000

much r very 0.09130244388482300000000

much r often 0.04348537677851460000000

much r decidedly 0.00561576544361691000000

much r regularly 0.00002981949674480720000

much r well 0.57942181784504200000000

much r so 0.13766811963241200000000

much r nearly 0.01090530384901540000000

much r close 0.00241174988968610000000

blow n snow 0.00044434296730369900000

blow n misfortune 0.00141753656686567000000

blow n row 0.00027263243520458300000

blow n disappointment 0.38590361897636500000000

blow n shock 0.07532668394708260000000

blow n wound 0.00034342106419823400000

blow n cut 0.00035438414171641600000

blow n sound 0.00489564402409835000000

blow n tragedy 0.02223760489270520000000

blow n affliction 0.05831149892851080000000

blow n calamity 0.00392254007975308000000

blow n wind 0.40930658905781800000000

blow n box 0.01110801386302380000000

blow n thrust 0.00035438414171641600000

blow n surprise 0.02277668244861250000000

blow n breath 0.00106315242514925000000

blow n squall 0.00196127003987654000000

youngster n boy 1.00000000000000000000000

suspect v guess 0.00473700225383668000000

suspect v reckon 0.00346456924271658000000

suspect v assume 0.00064479296638174400000

suspect v believe 0.15192205824755400000000

suspect v conclude 0.00020932512379963200000

suspect v understand 0.17490093390847200000000

suspect v think 0.42754502678549400000000

suspect v suppose 0.04923930337109570000000

suspect v conceive 0.00181749622595337000000

suspect v expect 0.01494841272942210000000

suspect v presume 0.00013095911589758400000

suspect v divine 0.00004203422644222830000

suspect v wonder 0.00587306978891710000000

suspect v fear 0.00859123474976548000000

suspect v feel 0.14312632335821900000000

suspect v imagine 0.00558886720982922000000

suspect v speculate 0.00010551125422074200000

suspect v doubt 0.00570158475933959000000

suspect v fancy 0.00141149468264332000000

watchfulness n attention 1.00000000000000000000000

tongue n voice 0.60871995528228100000000

tongue n knife 0.39128004471771900000000

upstart n nobody 0.50000000000000000000000

upstart n intruder 0.50000000000000000000000

unheard a silent 1.00000000000000000000000

late r lately 0.04799814821205560000000

late r formerly 0.66686166855715200000000

late r previously 0.04632311398804730000000

late r slowly 0.04106704728467770000000

late r latterly 0.19775002195806800000000

dreadful a awful 0.08234061737199250000000

dreadful a outrageous 0.00111716777031041000000

dreadful a painful 0.00358670467461336000000

dreadful a dreaded 0.00189314170740890000000

dreadful a horrible 0.08704321307922560000000

dreadful a wicked 0.00369058280187417000000

dreadful a evil 0.00442839227060378000000

dreadful a unpleasing 0.00015465822027788500000

dreadful a fearful 0.00253525056319488000000

dreadful a disagreeable 0.01639560607509650000000

dreadful a enormous 0.07356207777360280000000

dreadful a hideous 0.02829631500756620000000

dreadful a unpleasant 0.01527456209806030000000

dreadful a ugly 0.02845844103891510000000

dreadful a frightful 0.00421100221866050000000

dreadful a monstrous 0.00236336862256066000000

dreadful a dark 0.00466237945633372000000

dreadful a bad 0.05397499618162330000000

dreadful a horrid 0.26028181568560000000000

dreadful a alarming 0.02262808420910270000000

dreadful a odious 0.00055858388515520200000

dreadful a infamous 0.00225595959283487000000

dreadful a abominable 0.00755399987938365000000

dreadful a vile 0.01273931153486540000000

dreadful a distressing 0.00055858388515520200000

dreadful a shocking 0.00544834691553068000000

dreadful a terrible 0.23174777478577700000000

dreadful a real 0.00445691989882907000000

dreadful a disgraceful 0.02493861403864850000000

dreadful a offensive 0.00007732911013894230000

dreadful a loathsome 0.00643683210907323000000

dreadful a unwelcome 0.00268242747697197000000

dreadful a appalling 0.00241915144771931000000

dreadful a grievous 0.00061389430664666100000

dreadful a deplorable 0.00061389430664666100000

target n point 1.00000000000000000000000

absence n loss 0.03126172046030940000000

absence n holiday 0.11287495146593500000000

absence n vacation 0.03126172046030940000000

absence n need 0.82460160761344600000000

signal v show 0.66666666666666700000000

signal v speak 0.33333333333333300000000

music n song 0.50400231477678600000000

music n air 0.03372324003710910000000

music n round 0.01281863795849980000000

music n folk 0.00155834814233478000000

music n soul 0.04084135885345700000000

music n opera 0.00760055983388158000000

music n medicine 0.05098024637066630000000

music n tune 0.00225081730484695000000

music n singing 0.30054302273068500000000

music n hymn 0.00119124085463169000000

music n passage 0.04449021313710140000000

sentimentality n sentiment 0.50000000000000000000000

sentimentality n treacle 0.50000000000000000000000

middle n heart 0.08275174476570290000000

middle n eye 0.91724825523429700000000

remedy v treat 0.33333333333333300000000

remedy v fix 0.66666666666666700000000

red-hot a violent 1.00000000000000000000000

detail n group 0.00009793038812754170000

detail n exactness 0.42129652972468700000000

detail n article 0.00009793038812754170000

detail n part 0.01345220651716760000000

detail n description 0.03281599614229300000000

detail n point 0.06129349464618460000000

detail n fact 0.02508851968616250000000

detail n commission 0.00009793038812754170000

detail n job 0.21064826486234300000000

detail n peculiarity 0.00009793038812754170000

detail n body 0.00072304896405838600000

detail n attention 0.00314435158739676000000

detail n account 0.18667210783763200000000

detail n circumstance 0.00194640037344907000000

detail n duty 0.00039172155251016900000

detail n company 0.00452807851889063000000

detail n party 0.00214226114970416000000

detail n trifle 0.00009793038812754170000

detail n fidelity 0.00009793038812754170000

detail n particular 0.00118786615523878000000

detail n item 0.03183129335697630000000

detail n relation 0.00019586077625508400000

detail n paragraph 0.00009793038812754170000

detail n respect 0.00195648543215799000000

ransom n price 0.32955415964455300000000

ransom n freedom 0.00188958684438996000000

ransom n amount 0.00377917368877993000000

ransom n pay 0.00188958684438996000000

ransom n penalty 0.65910831928910700000000

ransom n take 0.00377917368877993000000

preposterous a mad 0.66666666666666700000000

preposterous a ridiculous 0.33333333333333300000000

blow v destroy 0.00328054012654935000000

blow v pass 0.00231239523446752000000

blow v breathe 0.22307672860535600000000

blow v depart 0.00048389745901378700000

blow v burst 0.00749837743211280000000

blow v bear 0.01389670407418640000000

blow v scream 0.00374918871605640000000

blow v spoil 0.00048389745901378700000

blow v gasp 0.00374918871605640000000

blow v roar 0.00374918871605640000000

blow v drift 0.00048389745901378700000

blow v pant 0.00374918871605640000000

blow v go 0.57005319413849100000000

blow v split 0.00254944832691835000000

blow v vibrate 0.01062270136215980000000

blow v rush 0.00105974692924159000000

blow v miss 0.14920171652925100000000

peaceable a temperate 0.04166666666666660000000

peaceable a kind 0.54166666666666700000000

peaceable a civil 0.04166666666666660000000

peaceable a mild 0.04166666666666660000000

peaceable a quiet 0.25000000000000000000000

peaceable a moderate 0.08333333333333330000000

people n group 0.00217999538498815000000

people n children 0.00202537245827217000000

people n bourgeoisie 0.00021813634875238700000

people n mass 0.04308244073565230000000

people n race 0.02676063407360930000000

people n world 0.20555244085361900000000

people n family 0.23845233322732700000000

people n nation 0.07583766807114880000000

people n public 0.00427916363773537000000

people n rag 0.00130422726796746000000

people n humanity 0.00503857120029229000000

people n folk 0.01615120021737490000000

people n state 0.04699386563413790000000

people n society 0.07337539171002360000000

people n commons 0.00148955963862345000000

people n mob 0.01597595606659220000000

people n vulgar 0.00721863517179055000000

people n everybody 0.15383586091399300000000

people n crowd 0.02822594098170120000000

people n populace 0.00169935677082394000000

people n they 0.00214186764562139000000

people n anybody 0.04665589123220050000000

people n rabble 0.00150549075775311000000

sofa n settee 0.16336633663366300000000

sofa n couch 0.83663366336633700000000

nothingness n absence 0.25000000000000000000000

nothingness n wind 0.25000000000000000000000

nothingness n sleep 0.50000000000000100000000

nervousness n alarm 0.06582611622853980000000

nervousness n dread 0.13375360855239600000000

nervousness n agitation 0.06582611622853980000000

nervousness n fright 0.06582611622853980000000

nervousness n animation 0.06792749232385660000000

nervousness n trouble 0.40126082565719100000000

nervousness n delirium 0.06582611622853980000000

nervousness n fear 0.06582611622853980000000

nervousness n worry 0.06792749232385660000000

extraordinary a wonderful 0.29599469128529300000000

extraordinary a grand 0.00032503069498672000000

extraordinary a odd 0.19929495083399400000000

extraordinary a remarkable 0.10614540438197800000000

extraordinary a unexpected 0.00088584794598209000000

extraordinary a singular 0.00078654326326029700000

extraordinary a rare 0.00032503069498672000000

extraordinary a unusual 0.00221470051286537000000

extraordinary a strange 0.15347925082027300000000

extraordinary a monstrous 0.00107331796295226000000

extraordinary a striking 0.00078654326326029700000

extraordinary a sublime 0.05769294836014290000000

extraordinary a glorious 0.00277194188167605000000

extraordinary a tremendous 0.01254194529568320000000

extraordinary a surprising 0.00601723523843747000000

extraordinary a prodigious 0.00013756067801655400000

extraordinary a uncommon 0.00074828726796553600000

extraordinary a incredible 0.00338266847162503000000

extraordinary a astonishing 0.03189954019445890000000

extraordinary a exceptional 0.00221470051286537000000

extraordinary a particular 0.00443784842935941000000

extraordinary a important 0.06176322535446090000000

extraordinary a curious 0.05494322597746080000000

extraordinary a unreal 0.00013756067801655400000

moor v tie 1.00000000000000000000000

mindful a attentive 1.00000000000000000000000

weak a stale 0.00007475699243595120000

weak a miserable 0.01846785528296930000000

weak a gentle 0.00475831885835047000000

weak a trifling 0.00065767808069419900000

weak a thin 0.00689152674555994000000

weak a slight 0.00131535616138840000000

weak a soft 0.01176602127290720000000

weak a futile 0.00204409691605591000000

weak a empty 0.00044622930400123100000

weak a dull 0.00167757787049577000000

weak a distant 0.00065767808069419900000

weak a stupid 0.03432155913308870000000

weak a ailing 0.24120343609459800000000

weak a worn 0.00036684701033632500000

weak a delicate 0.00126754770419508000000

weak a faint 0.01928654554194540000000

weak a poor 0.03258122427560840000000

weak a infirm 0.00065767808069419900000

weak a frightened 0.07610453800511800000000

weak a foolish 0.00864848690972062000000

weak a nervous 0.00125031551581606000000

weak a useless 0.00229707849485015000000

weak a loose 0.00007475699243595120000

weak a flat 0.00034653551197916000000

weak a tired 0.00569550153502097000000

weak a timid 0.00065767808069419900000

weak a feeble 0.00073012242251567300000

weak a silly 0.01589057263857270000000

weak a tiny 0.00027177851954320800000

weak a small 0.00651688764672884000000

weak a hopeless 0.01053369467791150000000

weak a childish 0.00007475699243595120000

weak a careless 0.00689152674555994000000

weak a helpless 0.00266009829696381000000

weak a short 0.10426261052736200000000

weak a limited 0.00100190094205888000000

weak a defective 0.00060986962350087900000

weak a dead 0.01453818686811540000000

weak a uncertain 0.00153932622373829000000

weak a dumb 0.00066739442755781300000

weak a unsure 0.00075024396919004000000

weak a mealy-mouthed 0.00007244434182147360000

weak a low 0.05128352662915700000000

weak a tender 0.00326383616305767000000

weak a inferior 0.00059263743512186200000

weak a quiet 0.03780587638872220000000

weak a pathetic 0.00085533133366878600000

weak a accessible 0.00007244434182147360000

weak a lame 0.00007475699243595120000

weak a shallow 0.00054355703908641700000

weak a meek 0.00118527487024372000000

weak a unwell 0.08047590235730190000000

weak a indistinct 0.00027177851954320800000

weak a powerless 0.00063810432829258700000

weak a pale 0.10935074732678100000000

weak a slender 0.00007475699243595120000

weak a deficient 0.00516109582996719000000

weak a young 0.05319313086390140000000

weak a idiotic 0.00034653551197916000000

weak a simple 0.01106463268425180000000

weak a superficial 0.00204409691605591000000

weak a light 0.00060986962350087900000

weak a unprepared 0.00021964567607889900000

weak a green 0.00034422286136468200000

spring n principle 0.00994046885989136000000

spring n opening 0.15404098824488600000000

spring n well 0.25736116328718800000000

spring n rent 0.01006371740083420000000

spring n gyre 0.01353663591375840000000

spring n fountain 0.01353663591375840000000

spring n foundation 0.00503185870041711000000

spring n bloom 0.15404098824488600000000

spring n cause 0.20324726168740900000000

spring n bound 0.15404098824488600000000

spring n flower 0.00503185870041711000000

spring n youth 0.02012743480166850000000

hew v cast 1.00000000000000000000000

baby n child 0.87569149071453200000000

baby n sister 0.11778261481686400000000

baby n infant 0.00020879147901104600000

baby n coward 0.00243811382340979000000

baby n cherub 0.00312739808204403000000

baby n babe 0.00054279960512774000000

baby n brat 0.00020879147901104600000

wonderful a excellent 0.00165207469918845000000

wonderful a extraordinary 0.23308309855338700000000

wonderful a grand 0.01490234443967990000000

wonderful a delightful 0.03933833925593840000000

wonderful a splendid 0.00912527256621383000000

wonderful a lovely 0.01272104963767460000000

wonderful a remarkable 0.01844270166868700000000

wonderful a wicked 0.01303592929742650000000

wonderful a brilliant 0.00203335351989739000000

wonderful a cool 0.00273437615032620000000

wonderful a magnificent 0.00332329994334626000000

wonderful a strange 0.02070666394540740000000

wonderful a striking 0.00216853066296527000000

wonderful a admirable 0.01289248137084240000000

wonderful a sublime 0.00098563465589293700000

wonderful a glorious 0.00010832312205322400000

wonderful a bad 0.03608941651709580000000

wonderful a fine 0.01606400751361040000000

wonderful a surprising 0.00845398111527618000000

wonderful a prodigious 0.00010832312205322400000

wonderful a incredible 0.00538350748425830000000

wonderful a astonishing 0.00960560152200147000000

wonderful a amazing 0.40887645650210200000000

wonderful a first-rate 0.00098563465589293700000

wonderful a great 0.04024997196463940000000

wonderful a pleasant 0.07220017454752380000000

wonderful a magic 0.00137668840573098000000

wonderful a miraculous 0.00049281732794646700000

wonderful a marvelous 0.01112909708984920000000

wonderful a unreal 0.00173084874309331000000

futile a vain 0.53809012875536500000000

futile a forlorn 0.46190987124463500000000

wonder n curiosity 0.00529202075640773000000

wonder n sensation 0.33798455020764700000000

wonder n admiration 0.01154341369672560000000

wonder n astonishment 0.01781898794783530000000

wonder n spectacle 0.00123139347606992000000

wonder n perplexity 0.02067891802917420000000

wonder n sight 0.06802446166794150000000

wonder n phenomenon 0.06367495942387370000000

wonder n surprise 0.01234666922370650000000

wonder n beauty 0.45406661909426100000000

wonder n prodigy 0.00733800647635719000000

fantasy n chimera 0.20000000000000000000000

fantasy n phantasm 0.20000000000000000000000

fantasy n whim 0.20000000000000000000000

fantasy n phantom 0.20000000000000000000000

fantasy n dream 0.20000000000000000000000

fling v shoot 0.00046657132258818400000

fling v send 0.16364989139780600000000

fling v dispose 0.00011664283064704600000

fling v fire 0.00023328566129409200000

fling v throw 0.83541696595701800000000

fling v cast 0.00011664283064704600000

expiation n satisfaction 1.00000000000000000000000

due n right 1.00000000000000000000000

depend v reckon 0.06494699568199330000000

depend v count 0.01158599330246150000000

depend v calculate 0.01971230484899710000000

depend v look 0.36796074867914500000000

depend v bet 0.01058884030490010000000

depend v hang 0.00501052749545193000000

depend v turn 0.52019458968705100000000

disbelieve v question 0.18072289156626500000000

disbelieve v doubt 0.81927710843373500000000

depreciate v fall 0.19740869687127600000000

depreciate v despise 0.17149566558403600000000

depreciate v attack 0.03745589644133930000000

depreciate v condemn 0.09799752319087790000000

depreciate v tumble 0.03745589644133930000000

depreciate v lessen 0.04899876159543900000000

depreciate v drop 0.08645465803677830000000

depreciate v denounce 0.04899876159543900000000

depreciate v cut 0.11236768932401800000000

depreciate v sink 0.02449938079771940000000

depreciate v slight 0.03745589644133930000000

depreciate v knock 0.03745589644133930000000

depreciate v abate 0.02449938079771940000000

depreciate v ridicule 0.03745589644133930000000

yet r further 0.00475909595124882000000

yet r though 0.00553466028611428000000

yet r moreover 0.02739693597874050000000

yet r heretofore 0.00011956615039651200000

yet r furthermore 0.00014476340495466800000

yet r nevertheless 0.01963475002316450000000

yet r however 0.07871834530467730000000

yet r anyway 0.01643519634095820000000

yet r already 0.14281041552022200000000

yet r also 0.01045324813326330000000

yet r too 0.29731534210918700000000

yet r still 0.18558348381999000000000

yet r even 0.21109419697708300000000

vain a unsuccessful 0.00491339373588193000000

vain a fruitless 0.00315958761522246000000

vain a unavailing 0.00453608356019204000000

vain a trifling 0.00461338559768678000000

vain a deceitful 0.00461338559768678000000

vain a futile 0.09399773155286830000000

vain a empty 0.09904696255116180000000

vain a presumptuous 0.00052439459722659900000

vain a false 0.06205214594845760000000

vain a unfounded 0.00242054244342150000000

vain a pretentious 0.00304903289013657000000

vain a unproductive 0.00304903289013657000000

vain a useless 0.07825542841641780000000

vain a idle 0.02461334466080890000000

vain a romantic 0.10032462501135000000000

vain a haughty 0.04706553243652230000000

vain a affected 0.00814566769718070000000

vain a insolent 0.00052439459722659900000

vain a imaginary 0.00892508269884737000000

vain a petty 0.00052439459722659900000

vain a shallow 0.00381286573059522000000

vain a fanciful 0.00609806578027317000000

vain a unimportant 0.00190643286529761000000

vain a proud 0.42835891119461700000000

vain a unreal 0.00242054244342150000000

vain a showy 0.00304903289013657000000

contagion n circulation 1.00000000000000000000000

commissioner n chancellor 1.00000000000000000000000

combat v encounter 1.00000000000000000000000

clash v fight 0.11111111111111100000000

clash v contest 0.11111111111111100000000

clash v quarrel 0.11111111111111100000000

clash v meet 0.55555555555555500000000

clash v strike 0.11111111111111100000000

tall a pretentious 0.00074710983873846000000

tall a long-legged 0.15075770958058900000000

tall a absurd 0.00703662237497036000000

tall a lofty 0.01257902507246380000000

tall a large 0.01482035458867920000000

tall a high 0.02110986712491110000000

tall a unreasonable 0.00149421967747692000000

tall a incredible 0.00074710983873846000000

tall a impossible 0.01314842141202130000000

tall a hard 0.00298843935495385000000

tall a great 0.04978295979231220000000

tall a unlikely 0.00074710983873846000000

tall a big 0.72329394166666800000000

tall a difficult 0.00074710983873846000000

cashier n clerk 0.66666666666666700000000

cashier n banker 0.33333333333333300000000

reckon v treat 0.00075590836087295800000

reckon v guess 0.05862069844246740000000

reckon v depend 0.00304059822565371000000

reckon v believe 0.06247345275008130000000

reckon v conclude 0.00039833749210945500000

reckon v name 0.00079609092652632600000

reckon v count 0.00091590323462159100000

reckon v hold 0.00441865942556063000000

reckon v calculate 0.00014175915884655400000

reckon v think 0.30478560846120900000000

reckon v suppose 0.07221484053559830000000

reckon v handle 0.00005781870363519060000

reckon v look 0.10435309430410600000000

reckon v conceive 0.00172150094868842000000

reckon v foresee 0.00028310851372699900000

reckon v account 0.00084023938731475600000

reckon v anticipate 0.00147806386420221000000

reckon v argue 0.00127892974437089000000

reckon v consider 0.02553798868265700000000

reckon v tell 0.12666064096240300000000

reckon v value 0.00018007478346767900000

reckon v presume 0.00005781870363519060000

reckon v esteem 0.00189285165835206000000

reckon v bet 0.00206437167530768000000

reckon v settle 0.00437924495798685000000

reckon v daresay 0.00244699285867117000000

reckon v imagine 0.00662828379181359000000

reckon v infer 0.00018055348109378500000

reckon v regard 0.00084393878667754700000

reckon v number 0.00018055348109378500000

reckon v see 0.19622219260436700000000

reckon v trust 0.00962661056927210000000

reckon v fancy 0.00148865034947621000000

reckon v judge 0.00303462017413426000000

shame n misfortune 0.00070430708211488600000

shame n virtue 0.00051733623898399400000

shame n sin 0.00277614007937356000000

shame n abomination 0.00034125946845527400000

shame n disappointment 0.00225590350805130000000

shame n contempt 0.00123502543502374000000

shame n disgrace 0.00703372288255797000000

shame n reproach 0.00887674531423312000000

shame n remorse 0.00034125946845527400000

shame n embarrassment 0.07800413018950730000000

shame n perplexity 0.03070874054653620000000

shame n propriety 0.08115881430156000000000

shame n humiliation 0.37874113340728000000000

shame n scandal 0.00017607677052872000000

shame n honor 0.00446657687315209000000

shame n modesty 0.00017607677052872000000

shame n degradation 0.37931009855511300000000

shame n fitness 0.00017607677052872000000

shame n pity 0.01909249251519840000000

shame n baseness 0.00130002677370920000000

shame n dishonor 0.00017607677052872000000

shame n crime 0.00243198027858002000000

barrow n cart 1.00000000000000000000000

greatly r seriously 0.00186741363211951000000

greatly r immensely 0.99813258636788000000000

absolutely r quite 0.34537689374539800000000

absolutely r surely 0.00446940434121162000000

absolutely r clearly 0.02907500187214430000000

absolutely r truly 0.00150908314215282000000

absolutely r indeed 0.07046200783237940000000

absolutely r wholly 0.21806251404108200000000

absolutely r positively 0.00671486508579017000000

absolutely r really 0.20207563219243400000000

absolutely r certainly 0.07280697345620040000000

absolutely r decidedly 0.00150908314215282000000

absolutely r perfectly 0.01584537299260460000000

absolutely r exactly 0.03058408501429710000000

absolutely r entirely 0.00150908314215282000000

reflection n mark 0.00044193043985307500000

reflection n proof 0.00394479195573947000000

reflection n evidence 0.00044193043985307500000

reflection n observation 0.00044193043985307500000

reflection n notion 0.00044193043985307500000

reflection n indictment 0.00230087161765570000000

reflection n thought 0.75125019939120600000000

reflection n feeling 0.01622635671920240000000

reflection n thinking 0.00542954759959713000000

reflection n light 0.00416230313135958000000

reflection n idea 0.05355111628613420000000

reflection n reproach 0.00230087161765570000000

reflection n conjecture 0.02846824861391980000000

reflection n opinion 0.09558727125222670000000

reflection n appearance 0.01860745389035700000000

reflection n display 0.00044193043985307500000

reflection n imitation 0.00230087161765570000000

reflection n sign 0.00542954759959713000000

reflection n picture 0.00044193043985307500000

reflection n impression 0.00274280205750878000000

reflection n remark 0.00460423357121265000000

reflection n study 0.00044193043985307500000

post n work 0.00475014926731553000000

post n place 0.61395679280053200000000

post n living 0.01453152172308950000000

post n picket 0.00475014926731553000000

post n part 0.00475014926731553000000

post n position 0.07674459910006650000000

post n duty 0.02640674377636700000000

post n pillar 0.12279135856010600000000

post n seat 0.05457393713782510000000

post n berth 0.07674459910006650000000

unfriendly a suspicious 1.00000000000000000000000

uncanny a awful 0.30769230769230800000000

uncanny a dreadful 0.17948717948718000000000

uncanny a wonderful 0.17948717948718000000000

uncanny a odd 0.02564102564102550000000

uncanny a superhuman 0.02564102564102550000000

uncanny a strange 0.17948717948718000000000

uncanny a incredible 0.05128205128205120000000

uncanny a astonishing 0.02564102564102550000000

uncanny a marvelous 0.02564102564102550000000

period n spring 0.00005760103028181840000

period n year 0.04706702808369470000000

period n winter 0.00005760103028181840000

period n interval 0.00399547146506907000000

period n hour 0.19189783238387900000000

period n stage 0.00005760103028181840000

period n end 0.10122623163736500000000

period n time 0.26192103342649700000000

period n minute 0.12791268791249200000000

period n second 0.00005760103028181840000

period n point 0.00080641442394546200000

period n day 0.04445926323757080000000

period n term 0.00017280309084545600000

period n month 0.00188410227145625000000

period n week 0.00005760103028181840000

period n age 0.09034123694271990000000

period n quarter 0.00005760103028181840000

period n moment 0.12797028894277400000000

thereupon r then 0.82078040324409300000000

thereupon r suddenly 0.17921959675590700000000

kind a noble 0.04267062806420580000000

kind a gentle 0.02103841468934320000000

kind a temperate 0.00178612567925195000000

kind a soft 0.01185631842290770000000

kind a nice 0.09877406802379510000000

kind a helpful 0.00178612567925195000000

kind a friendly 0.00009080526198652830000

kind a delicate 0.00204093909668486000000

kind a soft-hearted 0.00178612567925195000000

kind a generous 0.00457886492656230000000

kind a affectionate 0.10670686580617000000000

kind a amiable 0.00154425219060583000000

kind a willing 0.00226567218722919000000

kind a thoughtful 0.00584238032479643000000

kind a kindhearted 0.00592815921145387000000

kind a christian 0.00030654925832261900000

kind a good-natured 0.00556945859435206000000

kind a mild 0.00178612567925195000000

kind a decent 0.00009080526198652830000

kind a patient 0.01213619421877050000000

kind a sensitive 0.00382706477593681000000

kind a tender 0.00221774050596270000000

kind a loving 0.13437160879295400000000

kind a good 0.32158421775265700000000

kind a cordial 0.00447248815740896000000

kind a human 0.00205527882535935000000

kind a genial 0.00158706624558608000000

kind a merciful 0.00013886377729159100000

kind a considerate 0.00494768627896813000000

kind a obliging 0.03063919650868150000000

kind a sweet 0.05945600548214070000000

kind a affable 0.00357225135850390000000

kind a pleasant 0.01793513250664670000000

kind a big 0.00096980357730744400000

kind a brotherly 0.00009080526198652830000

kind a congenial 0.00158706624558608000000

kind a solicitous 0.00025481341743291000000

kind a agreeable 0.05818777961224510000000

kind a warm 0.02352025266116400000000

tartar n beast 1.00000000000000000000000

tactic n arrangement 0.92000000000000000000000

tactic n method 0.08000000000000000000000

surrender n yield 0.50000000000000000000000

surrender n fall 0.50000000000000000000000

succumb v withdraw 0.03418113309110480000000

succumb v yield 0.28219620508679900000000

succumb v fall 0.06836226618220960000000

succumb v drop 0.03418113309110480000000

succumb v surrender 0.06836226618220960000000

succumb v go 0.37599246400215300000000

succumb v admit 0.13672453236441900000000

call v decree 0.00023856408277137900000

call v gather 0.00066170550501495800000

call v upbraid 0.00004594925798856100000

call v rebuke 0.00016368146790147400000

call v guess 0.01966131816222340000000

call v demand 0.00769943395703403000000

call v ring 0.02053167177518130000000

call v reckon 0.00156549733670002000000

call v invite 0.03091056189523960000000

call v denominate 0.00009959210441329120000

call v charge 0.00039001194447502800000

call v petition 0.00453271757265620000000

call v cry 0.15667369589444000000000

call v announce 0.00202664212317566000000

call v holler 0.00015992474821009000000

call v name 0.04219415889930160000000

call v baptize 0.00000884897558860644000

call v whoop 0.00022320200168382800000

call v rouse 0.00093954207437314000000

call v entreat 0.00041212721928824800000

call v collect 0.00022131523961843900000

call v bid 0.00002809982281570370000

call v command 0.00045283162092425600000

call v claim 0.00000884897558860644000

call v summon 0.00096274316016264800000

call v scream 0.00434675673790898000000

call v request 0.00168358081270087000000

call v anticipate 0.00722509862056006000000

call v appoint 0.00028193937054799300000

call v shake 0.00901678789887675000000

call v consider 0.01666598572316510000000

call v roar 0.00148551248179489000000

call v rally 0.00077533326900698300000

call v elect 0.00013260679912595100000

call v yell 0.00045780938531788100000

call v exclaim 0.03754624886569320000000

call v address 0.00111883297447869000000

call v shout 0.06860008082512170000000

call v ask 0.28598022840652400000000

call v howl 0.00016368146790147400000

call v challenge 0.00006404927004840280000

call v require 0.01075089879104660000000

call v promise 0.02682687230452300000000

call v christen 0.01978859444308080000000

call v exact 0.00007449472622570240000

call v designate 0.00002809982281570370000

call v ordain 0.00006392023851103570000

call v speak 0.16335373118969500000000

call v waken 0.00005901384921835830000

call v declare 0.02237469689561740000000

call v stir 0.00219659750519785000000

call v utter 0.00155160547190585000000

call v predict 0.00002809982281570370000

call v order 0.00020314069147523700000

call v visit 0.00322142330335677000000

call v judge 0.02312159221897130000000

close a stale 0.00043236084916174400000

close a private 0.01087012188587200000000

close a familiar 0.01907309680731770000000

close a stuffy 0.00825451053148151000000

close a secret 0.04402649459354850000000

close a narrow 0.00461639176463765000000

close a similar 0.00285278584830342000000

close a unrelenting 0.00539656587938250000000

close a near 0.16471225733512400000000

close a careful 0.01009750522759880000000

close a faithful 0.00180935444853486000000

close a intimate 0.00285278584830342000000

close a like 0.00613793254576860000000

close a uncomfortable 0.01426392924151710000000

close a motionless 0.00180935444853486000000

close a distinct 0.00542806334560457000000

close a true 0.29945134718478000000000

close a attentive 0.01317474930954790000000

close a solid 0.00043236084916174400000

close a limited 0.00285278584830342000000

close a convenient 0.00357296036486909000000

close a devoted 0.00647149474537314000000

close a breathless 0.00086472169832349100000

close a strong 0.02396428742306940000000

close a silent 0.07190024356662450000000

close a retired 0.01550013644244860000000

close a dear 0.12236949822985800000000

close a intent 0.00285278584830342000000

close a handy 0.00466214029683828000000

close a heavy 0.06282680776454330000000

close a oppressive 0.04540348819292160000000

close a crushed 0.00224171529769660000000

close a conscientious 0.00043236084916174400000

close a particular 0.00790153846210283000000

close a noticeable 0.00466214029683828000000

close a impending 0.00539656587938250000000

close a inseparable 0.00043236084916174400000

scandalous a shameful 0.90438171276494600000000

scandalous a infamous 0.04182111966543100000000

scandalous a wrong 0.01096220257896900000000

scandalous a disgraceful 0.02091055983271550000000

scandalous a contemptible 0.01096220257896900000000

scandalous a base 0.01096220257896900000000

withdraw v retract 0.18140552605912600000000

withdraw v quit 0.01091135020987400000000

withdraw v retire 0.15081168258245600000000

withdraw v yield 0.01846673664274970000000

withdraw v depart 0.02840664218401350000000

withdraw v abdicate 0.00056448539956003400000

withdraw v take 0.17743652523412800000000

withdraw v retreat 0.00056448539956003400000

withdraw v draw 0.00406494998561318000000

withdraw v flee 0.21403958150376400000000

withdraw v stop 0.06736054639379340000000

withdraw v repair 0.00056448539956003400000

withdraw v resign 0.00350046458605314000000

withdraw v start 0.00099599235973873500000

withdraw v repress 0.00732688336432986000000

withdraw v extract 0.00086301392035740200000

withdraw v dissolve 0.00043150696017870100000

withdraw v surrender 0.00086301392035740200000

withdraw v leave 0.10152928775394700000000

withdraw v remove 0.00950641740121601000000

withdraw v reverse 0.00891497631029298000000

withdraw v end 0.00043150696017870100000

withdraw v recall 0.00169345619868010000000

withdraw v cancel 0.00934648327047168000000

language n voice 0.14646935892737500000000

language n writing 0.00012153524058058100000

language n signal 0.00012153524058058100000

language n expression 0.04925975219781710000000

language n mode 0.00012153524058058100000

language n tone 0.79652107789565800000000

language n word 0.00726367001682668000000

language n talk 0.00012153524058058100000

pen v write 1.00000000000000000000000

peer n gentleman 0.17073170731707300000000

peer n duke 0.04878048780487800000000

peer n count 0.46341463414634200000000

peer n baronet 0.02439024390243900000000

peer n marquis 0.07317073170731710000000

peer n lord 0.02439024390243900000000

peer n squire 0.02439024390243900000000

peer n fellow 0.02439024390243900000000

peer n lady 0.14634146341463400000000

paradox n mystery 0.17391304347826100000000

paradox n nonsense 0.47826086956521800000000

paradox n knot 0.04347826086956500000000

paradox n riddle 0.13043478260869600000000

paradox n difficulty 0.13043478260869600000000

paradox n puzzle 0.04347826086956500000000

overture n beginning 1.00000000000000000000000

worthy a reliable 0.00099529938376801000000

worthy a honest 0.01149230333113130000000

worthy a noble 0.02109171866396090000000

worthy a excellent 0.05177753106301570000000

worthy a laudable 0.00526007825119938000000

worthy a proper 0.00526007825119938000000

worthy a meritorious 0.00526007825119938000000

worthy a precious 0.01518946606545800000000

worthy a seemly 0.00255895698706997000000

worthy a respectable 0.05066023132414010000000

worthy a moral 0.00224105935568869000000

worthy a honorable 0.00526007825119938000000

worthy a true 0.11769261245147300000000

worthy a right 0.14034285030013400000000

worthy a fit 0.10910431984784300000000

worthy a admirable 0.00409288376059154000000

worthy a decent 0.00099529938376801000000

worthy a valuable 0.01097194236446500000000

worthy a desirable 0.06094268575085910000000

worthy a suitable 0.00099529938376801000000

worthy a good 0.20478652572722800000000

worthy a virtuous 0.00552008448815290000000

worthy a upright 0.00142684576889370000000

worthy a acceptable 0.00109800662995600000000

worthy a deserving 0.00526007825119938000000

worthy a invaluable 0.07588215487248000000000

worthy a trustworthy 0.04241841561196310000000

worthy a estimable 0.04142311622819510000000

outstrip v pass 1.00000000000000000000000

outcome n conclusion 0.00703915530136383000000

outcome n end 0.92960844698636200000000

outcome n effect 0.00703915530136383000000

outcome n harvest 0.00703915530136383000000

outcome n flower 0.00703915530136383000000

outcome n consequence 0.01407831060272770000000

outcome n result 0.02815662120545530000000

hard r fast 0.00188470268576829000000

hard r constantly 0.74539991222136000000000

hard r heavily 0.14907998244427200000000

hard r strongly 0.00542109027070080000000

hard r near 0.00167977445007630000000

hard r hardly 0.00167977445007630000000

hard r emphatically 0.09317498902767000000000

hard r close 0.00167977445007630000000

negotiate v sell 0.13178294573643400000000

negotiate v talk 0.30232558139534900000000

negotiate v consult 0.13178294573643400000000

negotiate v do 0.43410852713178300000000

empty a uninhabited 0.06192989069333000000000

empty a weak 0.00242031352618773000000

empty a vain 0.07658581084596100000000

empty a dull 0.00441231280327551000000

empty a stupid 0.01670692571139060000000

empty a dry 0.04034828851462270000000

empty a foolish 0.01404832807997230000000

empty a useless 0.10732586944229400000000

empty a hollow 0.02907264313103550000000

empty a hungry 0.04396875006968690000000

empty a flat 0.00202738097148084000000

empty a untenanted 0.00506100170559612000000

empty a ignorant 0.04511272209988260000000

empty a idle 0.00078586510941378100000

empty a silly 0.00284058258444795000000

empty a dead 0.27334333508989800000000

empty a available 0.00078586510941378100000

empty a desolate 0.02372797909930150000000

empty a free 0.12723669310813000000000

empty a petty 0.00202738097148084000000

empty a clear 0.11305174127708300000000

empty a talkative 0.00718032005611594000000

label v name 1.00000000000000000000000

complete a over 0.00153261533957227000000

complete a explicit 0.00633724279299327000000

complete a comprehensive 0.00211241426433109000000

complete a extraordinary 0.08543566156277190000000

complete a intact 0.00308894619697157000000

complete a unreserved 0.00207131671055033000000

complete a faultless 0.00037121924310420600000

complete a pure 0.00700432098172940000000

complete a whole 0.00700432098172940000000

complete a full 0.00207131671055033000000

complete a flat 0.00082107422446598200000

complete a absolute 0.00323276353002896000000

complete a exquisite 0.00111365772931262000000

complete a independent 0.00174724855999377000000

complete a solid 0.00082107422446598200000

complete a positive 0.00657851809270043000000

complete a essential 0.00285175925684697000000

complete a sound 0.39924629595857600000000

complete a total 0.39924629595857600000000

complete a real 0.00657973411281175000000

complete a settled 0.00211241426433109000000

complete a clear 0.01780469890896580000000

complete a unlimited 0.01698920408334370000000

complete a perfect 0.02345466706817310000000

complete a spotless 0.00037121924310420600000

inveterate a old 0.60000000000000000000000

inveterate a obstinate 0.40000000000000000000000

intimidate v dishearten 0.03124999999999990000000

intimidate v frighten 0.37500000000000100000000

intimidate v cow 0.03124999999999990000000

intimidate v restrain 0.03124999999999990000000

intimidate v dispirit 0.03124999999999990000000

intimidate v alarm 0.25000000000000000000000

intimidate v terrify 0.03124999999999990000000

intimidate v drive 0.03124999999999990000000

intimidate v shock 0.03124999999999990000000

intimidate v dictate 0.03124999999999990000000

intimidate v force 0.06250000000000000000000

intimidate v discourage 0.06250000000000000000000

intact a complete 0.50000000000000000000000

intact a untouched 0.50000000000000000000000

feature n mark 0.01011818507304380000000

feature n aspect 0.00284731071621881000000

feature n article 0.00714832014153797000000

feature n specialty 0.00075896929288006700000

feature n quality 0.00151793858576014000000

feature n humor 0.00151793858576014000000

feature n look 0.03379149557581080000000

feature n point 0.19921728657279300000000

feature n expression 0.20104246841380300000000

feature n opinion 0.01726650521458170000000

feature n figure 0.21773067519522100000000

feature n story 0.00923666156487671000000

feature n appearance 0.00930090144153651000000

feature n profile 0.00284731071621881000000

feature n face 0.26860341590474300000000

feature n fiction 0.00075896929288006700000

feature n property 0.01629564771233440000000

initiate v teach 0.09090909090909070000000

initiate v introduce 0.09090909090909070000000

initiate v enter 0.09090909090909070000000

initiate v open 0.09090909090909070000000

initiate v conceive 0.18181818181818200000000

initiate v originate 0.09090909090909070000000

initiate v admit 0.36363636363636500000000

humiliated a abased 0.33333333333333300000000

humiliated a depressed 0.66666666666666700000000

off r aside 0.03308188922514750000000

off r far 0.01778448701039610000000

off r over 0.03942989274162160000000

off r out 0.27636063818756600000000

off r elsewhere 0.00794154965317091000000

off r forth 0.00143663188333040000000

off r away 0.62396491129876700000000

associate v relate 0.04019705566630860000000

associate v connect 0.04019705566630860000000

associate v marry 0.91960588866738300000000

goodly a beautiful 1.00000000000000000000000

gaol n jail 1.00000000000000000000000

fulfill v do 1.00000000000000000000000

forward v send 1.00000000000000000000000

peculiar a unique 0.00044929216610514300000

peculiar a droll 0.00044929216610514300000

peculiar a wonderful 0.09603373639373000000000

peculiar a secret 0.00044929216610514300000

peculiar a odd 0.00044929216610514300000

peculiar a remarkable 0.01141449743258470000000

peculiar a original 0.00044929216610514300000

peculiar a absurd 0.00740511851145365000000

peculiar a ridiculous 0.00133397738640409000000

peculiar a inner 0.00906025733711408000000

peculiar a strange 0.72861872171315300000000

peculiar a monstrous 0.00044929216610514300000

peculiar a limited 0.00021769652709689800000

peculiar a striking 0.11172855606080900000000

peculiar a unnatural 0.00021769652709689800000

peculiar a indescribable 0.00837942875108816000000

peculiar a uncommon 0.00065308958129069700000

peculiar a incredible 0.00111628085930719000000

peculiar a astonishing 0.00066698869320204100000

peculiar a characteristic 0.00044929216610514300000

peculiar a funny 0.00268185388471952000000

peculiar a queer 0.00021769652709689800000

peculiar a particular 0.00904641744429020000000

peculiar a inconceivable 0.00044929216610514300000

peculiar a fanciful 0.00043539305419379700000

peculiar a curious 0.00200096607960613000000

peculiar a unreal 0.00517728990692233000000

yes n permission 0.21010451402410000000000

yes n aye 0.12306465490028800000000

yes n consent 0.04887724234796340000000

yes n confession 0.38783718570569000000000

yes n recognition 0.00689098987563300000000

yes n nod 0.15558108127661600000000

yes n allowance 0.01189048091565090000000

yes n approbation 0.00553142552360527000000

yes n agreement 0.05022242543045270000000

access n means 0.09974093264248700000000

access n turn 0.90025906735751300000000

dupe n fool 0.61089370703331600000000

dupe n mark 0.03696456901110510000000

dupe n victim 0.11738057465186000000000

dupe n creature 0.11738057465186000000000

dupe n pigeon 0.11738057465186000000000

disquiet n anxiety 0.10526315789473700000000

disquiet n uneasiness 0.89473684210526300000000

lower a smaller 1.00000000000000000000000

demonstrative a convincing 0.14285714285714300000000

demonstrative a plain 0.14285714285714300000000

demonstrative a communicative 0.28571428571428600000000

demonstrative a sure 0.14285714285714300000000

demonstrative a open 0.14285714285714300000000

demonstrative a candid 0.14285714285714300000000

decisively r resolutely 1.00000000000000000000000

kitchen n galley 1.00000000000000000000000

curtsy n bow 1.00000000000000000000000

contention n strife 0.04166666666666650000000

contention n feud 0.04166666666666650000000

contention n game 0.08333333333333330000000

contention n struggle 0.12500000000000000000000

contention n belief 0.08333333333333330000000

contention n profession 0.08333333333333330000000

contention n argument 0.04166666666666650000000

contention n fight 0.08333333333333330000000

contention n opinion 0.25000000000000000000000

contention n war 0.12500000000000000000000

contention n fray 0.04166666666666650000000

confidant n confidante 0.50000000000000000000000

confidant n acquaintance 0.50000000000000000000000

confessor n priest 1.00000000000000000000000

compassionately r pityingly 1.00000000000000000000000

colloquy n conversation 1.00000000000000000000000

help n favor 0.00400605240087501000000

help n man 0.31860351935406900000000

help n maid 0.02214957845174100000000

help n restorative 0.00786040382699055000000

help n advice 0.03054301651568270000000

help n grace 0.00061378859635335400000

help n relief 0.00396129544786129000000

help n menial 0.00082346632684653000000

help n girl 0.01390936211698280000000

help n friendship 0.00020033270002873900000

help n assistance 0.09468826037802490000000

help n support 0.00431720210191385000000

help n blessing 0.00206611169126946000000

help n agent 0.00041345589632461600000

help n crew 0.00054627987284659300000

help n second 0.00185077630633275000000

help n staff 0.00085879572256109700000

help n gift 0.00047865642249421600000

help n aid 0.00194604733696773000000

help n charity 0.00200302620043751000000

help n hand 0.09708947900054490000000

help n improvement 0.00585555767227677000000

help n encouragement 0.00061759974513489800000

help n remedy 0.00034767147497820700000

help n benefactor 0.00062550731096320700000

help n use 0.01493621797468470000000

help n cure 0.00568313872208443000000

help n servant 0.00623090083246457000000

help n patron 0.00848518260170312000000

help n promotion 0.00044533982623648100000

help n means 0.00020033270002873900000

help n service 0.22864143110311300000000

help n way 0.09718866789031260000000

help n laborer 0.00354320172507671000000

help n assistant 0.00116387961798149000000

help n avail 0.00041345589632461600000

help n restoration 0.00021205141463859100000

help n benevolence 0.00082346632684653000000

help n force 0.00252654441191549000000

help n advocate 0.00431720210191385000000

help n advantage 0.00299171274874440000000

help n kindness 0.00109137323719029000000

help n comforter 0.00473065799823846000000

clog v check 0.25000000000000000000000

clog v stop 0.25000000000000000000000

clog v close 0.50000000000000000000000

clammy a close 1.00000000000000000000000

cheerless a miserable 0.14285714285714300000000

cheerless a unhappy 0.10714285714285700000000

cheerless a gloomy 0.07142857142857140000000

cheerless a lonely 0.10714285714285700000000

cheerless a dreary 0.03571428571428570000000

cheerless a dark 0.07142857142857140000000

cheerless a cold 0.25000000000000000000000

cheerless a depressed 0.07142857142857140000000

cheerless a serious 0.07142857142857140000000

cheerless a sad 0.07142857142857140000000

celebration n singing 1.00000000000000000000000

address n place 0.07542471252987480000000

address n apartment 0.02306019483991400000000

address n manners 0.00873277140181016000000

address n home 0.30000553444859400000000

address n air 0.00612160469232027000000

address n manner 0.00386617509927341000000

address n approach 0.06673613017515020000000

address n politeness 0.00051714931585416800000

address n sermon 0.20153896272027300000000

address n destination 0.00051714931585416800000

address n flat 0.00051714931585416800000

address n number 0.03439035517988200000000

address n house 0.16642043048564200000000

address n lecture 0.08342016271893780000000

address n talk 0.02281603715587500000000

address n speech 0.00103429863170834000000

address n situation 0.00488118197318290000000

witness n beholder 0.05072627307939780000000

witness n proof 0.08430114804430110000000

witness n evidence 0.00086735721172878600000

witness n profession 0.00276703054764420000000

witness n law 0.07029369791716160000000

witness n passer-by 0.00179273575541647000000

witness n word 0.78925175744435000000000

pair n couple 0.99583023082650800000000

pair n set 0.00416976917349216000000

alive a quick 0.01944020834043910000000

alive a conscious 0.05710245707010570000000

alive a safe 0.04862941805561620000000

alive a sharp 0.02352974595100760000000

alive a busy 0.02905476926794040000000

alive a cheerful 0.00716003568545141000000

alive a active 0.01774971164582780000000

alive a lively 0.00036467178989401400000

alive a full 0.04098233272837170000000

alive a existent 0.01774971164582780000000

alive a mortal 0.00355099155409296000000

alive a ready 0.08547646635634300000000

alive a awake 0.00731961286722220000000

alive a real 0.02255718793448960000000

alive a rich 0.11849632342556800000000

alive a aware 0.10365205655796100000000

alive a living 0.39352211978281900000000

alive a warm 0.00366217934102263000000

alienate v part 0.50000000000000000000000

alienate v separate 0.50000000000000000000000

advocate v push 0.16666666666666700000000

advocate v support 0.16666666666666700000000

advocate v encourage 0.16666666666666700000000

advocate v justify 0.16666666666666700000000

advocate v advance 0.16666666666666700000000

advocate v bless 0.16666666666666700000000

spare v excuse 0.04214269229351090000000

spare v keep 0.00759485612842947000000

spare v forestall 0.00060767899528637400000

spare v manage 0.00566664354951172000000

spare v yield 0.00034036722766284800000

spare v hold 0.00597832365130731000000

spare v afford 0.00375335171427575000000

spare v bestow 0.05263345911381410000000

spare v protect 0.02045815496093950000000

spare v grant 0.00900233011663882000000

spare v defend 0.00078307818816006500000

spare v stop 0.01663975858717290000000

spare v release 0.00088151287613805100000

spare v supply 0.00046629804443558100000

spare v guard 0.00104560107045320000000

spare v discharge 0.00442566073467311000000

spare v forget 0.03627428290969090000000

spare v sacrifice 0.00145409106584055000000

spare v halt 0.00019576954704001600000

spare v neglect 0.00223121513587529000000

spare v give 0.35061553814430900000000

spare v allow 0.02301551569078740000000

spare v refrain 0.00825838378697753000000

spare v pity 0.03633181197218040000000

spare v limit 0.00066763408794559000000

spare v stay 0.00937965474672850000000

spare v omit 0.00070548216914098100000

spare v save 0.11770447745891300000000

spare v free 0.00026437959890913700000

spare v drop 0.00290872046519323000000

spare v pardon 0.00612384695289774000000

spare v provide 0.00514209192194654000000

spare v rescue 0.00026437959890913700000

spare v forsake 0.00053613677470286400000

spare v leave 0.08320374709580720000000

spare v dispense 0.00029930241669541800000

spare v forgive 0.12316880198245500000000

spare v grudge 0.00089566533328508600000

spare v prevent 0.00918425595580679000000

spare v avoid 0.00801506215131021000000

spare v cancel 0.00044068336754621100000

spare v liberate 0.00029930241669541800000

sorrow n suffering 0.00042051527525533500000

sorrow n despair 0.00027330560428031500000

sorrow n scourge 0.00013665280214015700000

sorrow n grief 0.33108290448980600000000

sorrow n misery 0.02144309542087880000000

sorrow n cry 0.00068326401070078800000

sorrow n misfortune 0.00054661120856063000000

sorrow n evil 0.00021025763762766700000

sorrow n distress 0.00732306692805566000000

sorrow n trial 0.00013665280214015700000

sorrow n agony 0.00284464027508794000000

sorrow n burden 0.00131871785872251000000

sorrow n problem 0.00118206505658236000000

sorrow n weep 0.44104269147422500000000

sorrow n remorse 0.00832270894990782000000

sorrow n curse 0.00013665280214015700000

sorrow n vexation 0.00297912341962986000000

sorrow n woe 0.00013665280214015700000

sorrow n pressure 0.00744571234627445000000

sorrow n load 0.00021025763762766700000

sorrow n affliction 0.00297912341962986000000

sorrow n trouble 0.13929201017485000000000

sorrow n calamity 0.00471561781930715000000

sorrow n depression 0.00013242536900592200000

sorrow n unhappiness 0.00819028358090190000000

sorrow n pain 0.00413749580597016000000

sorrow n difficulty 0.01150433834024270000000

sorrow n hindrance 0.00021025763762766700000

sorrow n worry 0.00013665280214015700000

sorrow n grievance 0.00048356324190798200000

sorrow n impediment 0.00013242536900592200000

sorrow n penitence 0.00021025763762766700000

vexatious a wicked 0.33333333333333300000000

vexatious a disagreeable 0.33333333333333300000000

vexatious a offensive 0.33333333333333300000000

untrue a dishonorable 0.28679020502177900000000

untrue a mistaken 0.06337199823532240000000

untrue a wrong 0.58646579850757700000000

untrue a amiss 0.06337199823532240000000

invite v pray 0.00119176028276002000000

invite v persuade 0.01084101695635430000000

invite v suggest 0.01238463244395400000000

invite v push 0.00206587143257102000000

invite v call 0.38256745086912100000000

invite v insist 0.00423368586086835000000

invite v adjure 0.00011373905500384000000

invite v attract 0.00387922776959908000000

invite v urge 0.00400620775086067000000

invite v entreat 0.00985533541465712000000

invite v instigate 0.00017389003831297700000

invite v bid 0.00017389003831297700000

invite v encourage 0.00062740094568145900000

invite v receive 0.36595916010278100000000

invite v summon 0.00034778007662595400000

invite v draw 0.01327541122792340000000

invite v welcome 0.00017389003831297700000

invite v tempt 0.00077086008280197100000

invite v implore 0.00011373905500384000000

invite v issue 0.00017389003831297700000

invite v provoke 0.00110918662251973000000

invite v beseech 0.00011373905500384000000

invite v start 0.00306402895732741000000

invite v tease 0.00135227847418750000000

invite v seek 0.00095451614693364400000

invite v beg 0.00900757833164564000000

invite v court 0.00032856051389906400000

invite v ask 0.16495045638546700000000

invite v cause 0.00017389003831297700000

invite v press 0.00051510720332449900000

invite v propose 0.00550181879155903000000

unsettle v unhinge 0.29800853485064000000000

unsettle v trouble 0.29800853485064000000000

unsettle v confuse 0.29800853485064000000000

unsettle v shake 0.10597439544808000000000

undeceive v inform 1.00000000000000000000000

tried a true 1.00000000000000000000000

grief n suffering 0.30702740892270800000000

grief n anxiety 0.00057743012775135100000

grief n loss 0.00332380164360369000000

grief n blow 0.00410270737882828000000

grief n sorrow 0.32758231962670400000000

grief n misery 0.00540245772775413000000

grief n misfortune 0.01260956038216460000000

grief n distress 0.00494144597654396000000

grief n trial 0.00490166810692298000000

grief n accident 0.00993436143130089000000

grief n shock 0.00294762473997396000000

grief n burden 0.21812423075807300000000

grief n remorse 0.00283225408133198000000

grief n vexation 0.00294762473997396000000

grief n affliction 0.00294762473997396000000

grief n trouble 0.04652895097953050000000

grief n calamity 0.00049154756227171300000

grief n depression 0.00013102521745491600000

grief n unhappiness 0.00319277642614878000000

grief n repentance 0.00115486025550270000000

grief n disaster 0.01211801281989290000000

grief n pain 0.02028505687564200000000

grief n difficulty 0.00589524947994791000000

conscience n honesty 1.00000000000000000000000

staple n thing 1.00000000000000000000000

standstill n stop 0.33333333333333300000000

standstill n halt 0.66666666666666700000000

card n bill 0.01452593836756390000000

card n sheet 0.32594166968621400000000

card n list 0.03863601230313820000000

card n paper 0.26349687029251500000000

card n ticket 0.02468503242519480000000

card n notice 0.33271447692537400000000

snow v deceive 0.30000000000000000000000

snow v astonish 0.20000000000000000000000

snow v drown 0.09999999999999970000000

snow v amaze 0.09999999999999970000000

snow v overwhelm 0.20000000000000000000000

snow v overpower 0.09999999999999970000000

roll v wander 0.12756342161646300000000

roll v run 0.06378171080823180000000

roll v flow 0.19134513242469500000000

roll v move 0.26788318539457300000000

roll v go 0.28564483894780400000000

roll v turn 0.06378171080823180000000

savings n capital 0.62500000000000000000000

savings n property 0.37500000000000000000000

park n place 0.92772620365173700000000

park n chase 0.01100416692632600000000

park n yard 0.06126962942193670000000

refreshing a extraordinary 0.05000000000000000000000

refreshing a delightful 0.02500000000000000000000

refreshing a new 0.02500000000000000000000

refreshing a welcome 0.02500000000000000000000

refreshing a good 0.75000000000000000000000

refreshing a pleasant 0.12500000000000000000000

heart n pine 0.00003386286906845880000

heart n bottle 0.00671398211772616000000

heart n promise 0.00404233072651883000000

heart n middle 0.00022172354486417700000

heart n sorrow 0.01695989112802620000000

heart n courage 0.04867788347612100000000

heart n mind 0.08427389433897740000000

heart n sum 0.00007168744082876120000

heart n sentiment 0.00050646665356314800000

heart n sympathy 0.02157990389540860000000

heart n bosom 0.00939605633534585000000

heart n goodness 0.00806925267506313000000

heart n disposition 0.00129853173591756000000

heart n tenderness 0.02508862343872050000000

heart n clock 0.01886125610371850000000

heart n love 0.09041488157084600000000

heart n essential 0.00042465695880766000000

heart n backbone 0.00002329591870725420000

heart n center 0.00002329591870725420000

heart n feeling 0.03572699635876210000000

heart n understanding 0.00064113170918489100000

heart n concern 0.01021451027393550000000

heart n humanity 0.01296990874655980000000

heart n nerve 0.00077269826532105400000

heart n compassion 0.00046541350903378400000

heart n soul 0.18370940515205800000000

heart n meat 0.00333764907553146000000

heart n spirit 0.02562653022259800000000

heart n determination 0.00039839908352531800000

heart n substance 0.00002329591870725420000

heart n nature 0.07073739768991070000000

heart n character 0.00158231499606134000000

heart n seed 0.00758811561172435000000

heart n interior 0.00007168744082876120000

heart n response 0.00002329591870725420000

heart n eye 0.11182962453948200000000

heart n sensibility 0.00107497805233851000000

heart n gallantry 0.00079773527164434500000

heart n temperament 0.01274336960008160000000

heart n cheer 0.00285433981173251000000

heart n pump 0.00015093229258826500000

heart n pledge 0.00021598931525562000000

heart n vow 0.00053308001212025400000

heart n affection 0.09229245756579220000000

heart n please 0.00004688975777227440000

heart n life 0.04472270673535920000000

heart n boldness 0.00062119241081781700000

heart n pity 0.02597802258792890000000

heart n breast 0.01024660784002660000000

heart n passion 0.00403197771428357000000

heart n emotion 0.00022417262672939200000

heart n kindness 0.00073889582183910000000

heart n germ 0.00032680122482154900000

misery n suffering 0.06572835668121870000000

misery n despair 0.00093646728961888300000

misery n blow 0.00165690403495873000000

misery n sorrow 0.07347363952783670000000

misery n grief 0.01870905083563670000000

misery n misfortune 0.12464952284680700000000

misery n trial 0.00046823364480944000000

misery n agony 0.00190910713548914000000

misery n gloom 0.14434974364268200000000

misery n concern 0.00072043674533985000000

misery n uneasiness 0.03741810167127350000000

misery n burden 0.06030014869342990000000

misery n woe 0.00046823364480944000000

misery n sadness 0.14388150999787300000000

misery n load 0.00072043674533985000000

misery n wretchedness 0.06294816062406940000000

misery n trouble 0.07768177776765500000000

misery n unhappiness 0.01870905083563670000000

misery n pain 0.00306160496938706000000

misery n privation 0.04796050333262430000000

misery n worry 0.00046823364480944000000

misery n torture 0.11190784110945700000000

misery n need 0.00187293457923777000000

race v charge 0.50000000000000000000000

race v pursue 0.50000000000000000000000

manage v teach 0.00079705875374268400000

manage v treat 0.00604942168101356000000

manage v direct 0.00055736072862148900000

manage v run 0.00363283065028763000000

manage v maintain 0.00026490231013915000000

manage v conduct 0.00025886503101924800000

manage v experiment 0.00053208799198083700000

manage v show 0.02327327596783600000000

manage v use 0.01167466661297060000000

manage v advise 0.00264181798552056000000

manage v undertake 0.00069694431428259200000

manage v handle 0.00144322981895687000000

manage v deal 0.00468116568433210000000

manage v accomplish 0.00089913416608034800000

manage v impress 0.00125163711562111000000

manage v prevail 0.00013739256069260900000

manage v execute 0.00111055145406712000000

manage v work 0.00309847817147454000000

manage v do 0.90150556248537300000000

manage v lead 0.00358328418342628000000

manage v control 0.00157220396880422000000

manage v watch 0.00051673402043013700000

manage v influence 0.00050964052981914500000

manage v effect 0.00025886503101924800000

manage v govern 0.00038922427098359600000

manage v regulate 0.00056042944859837300000

manage v administer 0.00047898597690802100000

manage v achieve 0.00012750974944654100000

manage v master 0.00227391632730183000000

manage v care 0.00180091024173108000000

manage v contrive 0.00053208799198083700000

manage v fix 0.00423117240010171000000

manage v steer 0.00109086936148580000000

manage v cause 0.00101928105963829000000

manage v operate 0.00013739256069260900000

manage v arrange 0.00743976086022386000000

manage v succeed 0.00269892820893139000000

manage v order 0.00054216835753901500000

manage v perform 0.00573025196692621000000

plenty r enough 1.00000000000000000000000

wide a vast 0.25000000000000000000000

wide a large 0.25000000000000000000000

wide a general 0.25000000000000000000000

wide a liberal 0.25000000000000000000000

outrageous a dishonorable 0.00819672131147537000000

outrageous a enough 0.08196721311475410000000

outrageous a unendurable 0.01639344262295080000000

outrageous a dreadful 0.05737704918032790000000

outrageous a ignominious 0.00819672131147537000000

outrageous a horrible 0.06557377049180330000000

outrageous a wicked 0.01639344262295080000000

outrageous a execrable 0.00819672131147537000000

outrageous a hideous 0.01639344262295080000000

outrageous a ridiculous 0.01639344262295080000000

outrageous a black 0.02459016393442620000000

outrageous a monstrous 0.00819672131147537000000

outrageous a mean 0.00819672131147537000000

outrageous a furious 0.00819672131147537000000

outrageous a dark 0.02459016393442620000000

outrageous a bad 0.09836065573770500000000

outrageous a horrid 0.03278688524590160000000

outrageous a intolerable 0.02459016393442620000000

outrageous a frantic 0.00819672131147537000000

outrageous a shameful 0.01639344262295080000000

outrageous a odious 0.00819672131147537000000

outrageous a impossible 0.28688524590164000000000

outrageous a infamous 0.02459016393442620000000

outrageous a abominable 0.02459016393442620000000

outrageous a distressing 0.00819672131147537000000

outrageous a shocking 0.00819672131147537000000

outrageous a disgraceful 0.00819672131147537000000

outrageous a insulting 0.03278688524590160000000

outrageous a appalling 0.01639344262295080000000

outrageous a wild 0.01639344262295080000000

outrageous a insufferable 0.00819672131147537000000

outrageous a disorderly 0.00819672131147537000000

phrase n sentence 0.00069971766392260300000

phrase n part 0.00279887065569043000000

phrase n thought 0.19600341262300700000000

phrase n verse 0.00069971766392260300000

phrase n term 0.75324606521268700000000

phrase n expression 0.00139943532784521000000

phrase n remark 0.04515278085292580000000

oddity n exception 1.00000000000000000000000

niche n place 1.00000000000000000000000

liberty n sovereignty 0.00549711684180628000000

liberty n leave 0.02448007840033850000000

liberty n authority 0.14402446125532500000000

liberty n freedom 0.00562595551778612000000

liberty n choice 0.00816002613344615000000

liberty n right 0.11464606675573400000000

liberty n rest 0.38017132280666000000000

liberty n will 0.00816002613344615000000

liberty n opportunity 0.24004076875887400000000

liberty n life 0.06919417739658440000000

conscious a intentional 0.00564231025174499000000

conscious a sensible 0.00851629567862230000000

conscious a self-conscious 0.00196008253951030000000

conscious a sure 0.89981474184101100000000

conscious a certain 0.04545599078815970000000

conscious a attentive 0.00330017436930447000000

conscious a awake 0.00284191458892311000000

conscious a rational 0.00236461344391443000000

conscious a observant 0.00040453090440412500000

conscious a aware 0.02969934559440550000000

malefactor n murderer 0.50000000000000000000000

malefactor n scoundrel 0.50000000000000000000000

laudable a worthy 0.33333333333333300000000

laudable a meritorious 0.11111111111111100000000

laudable a respectable 0.11111111111111100000000

laudable a honorable 0.11111111111111100000000

laudable a true 0.11111111111111100000000

laudable a good 0.11111111111111100000000

laudable a deserving 0.11111111111111100000000

blame v upbraid 0.03236212884562830000000

blame v saddle 0.01829939853615070000000

blame v charge 0.04163623819516660000000

blame v lay 0.12407652624516700000000

blame v chide 0.01553247916985990000000

blame v condemn 0.32636422665856900000000

blame v accuse 0.14365194680499300000000

blame v reproach 0.17615362135699900000000

blame v attribute 0.01211783227460920000000

blame v scold 0.04637096640468360000000

blame v assign 0.00431087404714279000000

blame v denounce 0.05912376146103130000000

brain n mind 0.24534275945068000000000

brain n understanding 0.01895494114117970000000

brain n wit 0.00120756092736241000000

brain n apprehension 0.00112539591966393000000

brain n reason 0.00813410486148444000000

brain n head 0.72475272765544900000000

brain n cleverness 0.00048251004418023400000

implicate v betoken 0.00429939128893091000000

implicate v blame 0.01568317475326520000000

implicate v charge 0.00429939128893091000000

implicate v impute 0.00429939128893091000000

implicate v mean 0.91855534542875700000000

implicate v lay 0.00429939128893091000000

implicate v signify 0.01289817386679270000000

implicate v attribute 0.00429939128893091000000

implicate v concern 0.01568317475326520000000

implicate v entail 0.01568317475326520000000

clerk n cashier 0.02582854552483360000000

clerk n accountant 0.07188390044074430000000

clerk n secretary 0.12914272762416800000000

clerk n clergyman 0.21143781750329300000000

clerk n notary 0.21565170132223300000000

clerk n assistant 0.34605530758472800000000

gulp n draught 1.00000000000000000000000

gleefully r joyfully 1.00000000000000000000000

gape v gaze 1.00000000000000000000000

fuss v whimper 0.00158753066820608000000

fuss v disturb 0.01905036801847310000000

fuss v run 0.92538605859431300000000

fuss v complain 0.00793765334103044000000

fuss v worry 0.01270024534564870000000

fuss v object 0.01270024534564870000000

fuss v annoy 0.00158753066820608000000

fuss v bother 0.00476259200461826000000

fuss v fret 0.00793765334103044000000

fuss v irritate 0.00158753066820608000000

fuss v join 0.00317506133641218000000

fuss v fidget 0.00158753066820608000000

forthcoming a ready 0.50000000000000100000000

forthcoming a open 0.27777777777777800000000

forthcoming a available 0.11111111111111100000000

forthcoming a candid 0.05555555555555530000000

forthcoming a talkative 0.05555555555555530000000

fondle v kiss 1.00000000000000000000000

man n lad 0.01171743559543130000000

man n people 0.11782479056370200000000

man n mr 0.01359189677727490000000

man n everyone 0.01241753872395850000000

man n page 0.00185608599065509000000

man n lover 0.00298863718521990000000

man n gent 0.00771162743996365000000

man n satellite 0.00032403470129498900000

man n paragon 0.00020735858670884600000

man n herr 0.00005822326437886520000

man n guy 0.00015121716648681100000

man n world 0.04545970701803450000000

man n gentleman 0.15209643601002900000000

man n partner 0.00778232757780778000000

man n dependent 0.00004314346158113440000

man n someone 0.01489052190681750000000

man n person 0.04961642603141920000000

man n knight 0.00155786626344649000000

man n vassal 0.00021756942620587100000

man n humanity 0.00101359295132396000000

man n folk 0.00262513391489029000000

man n husband 0.03292244782694460000000

man n hero 0.01161280980368820000000

man n footman 0.00306086165906969000000

man n individual 0.00018026689377812000000

man n mankind 0.00175926837635045000000

man n waiter 0.00195512711130758000000

man n soul 0.03054936682085400000000

man n valet 0.00701645322364938000000

man n chap 0.04017564688971070000000

man n attendant 0.00912372464990051000000

man n subject 0.01144697457279540000000

man n homo 0.00003710486338394700000

man n master 0.04679010824404690000000

man n workman 0.00787147821260038000000

man n brick 0.00296564127178238000000

man n jewel 0.00010184100801125900000

man n squire 0.00320619978332658000000

man n fellow 0.12571232830040700000000

man n model 0.00027169829972349100000

man n groom 0.00028932729690185500000

man n bachelor 0.01005302707422570000000

man n blade 0.00010613901907897400000

man n society 0.00924644752991611000000

man n piece 0.00312690707428053000000

man n monsieur 0.01465588000633680000000

man n mortal 0.00203187403377058000000

man n aide-de-camp 0.00053187365766192400000

man n soldier 0.03324878967803880000000

man n buck 0.00077496286562540900000

man n don 0.00027525341123320600000

man n populace 0.00282460292817305000000

man n boots 0.00049081892145783300000

man n subordinate 0.00084471842705293300000

man n sir 0.10186427429545400000000

man n warrior 0.00433349854078468000000

man n human 0.00010013655599433400000

man n anybody 0.01828219137301710000000

man n anyone 0.01391294036167770000000

man n beau 0.00038809681431317800000

man n youth 0.00164965276250835000000

man n liege 0.00005766900453649590000

pass v squander 0.00000343670674597786000

pass v satisfy 0.00070722414505953300000

pass v hand 0.00117515690215687000000

pass v travel 0.00038771543885045700000

pass v retire 0.00268252644291847000000

pass v succumb 0.00002101684659533630000

pass v withdraw 0.00002789026008729200000

pass v outstrip 0.00137726022845063000000

pass v roll 0.00966201021805366000000

pass v return 0.01933082613648230000000

pass v announce 0.00009661260527530230000

pass v formulate 0.00003712917212335310000

pass v vanish 0.00384261203111154000000

pass v deliver 0.00095487758959805800000

pass v become 0.00066547196773220500000

pass v endure 0.00128100825876784000000

pass v clear 0.00739425132059921000000

pass v depart 0.00668582753870556000000

pass v last 0.00252706121131249000000

pass v fall 0.00820673452569804000000

pass v abandon 0.00083247556097274700000

pass v hasten 0.00059900869742733200000

pass v employ 0.01510369516141350000000

pass v answer 0.01665078818226860000000

pass v shoot 0.00085486607962696500000

pass v run 0.00769644985819278000000

pass v snub 0.00000343670674597786000

pass v connect 0.00000343670674597786000

pass v appear 0.00960294355100677000000

pass v post 0.01117548299657080000000

pass v pronounce 0.00098153618591807100000

pass v communicate 0.00161114086178122000000

pass v use 0.00133892068577137000000

pass v extend 0.00002789026008729200000

pass v send 0.00719781857731717000000

pass v convey 0.00437484983821537000000

pass v grant 0.00020918090726470800000

pass v alter 0.00096287659176398600000

pass v linger 0.00259248984178942000000

pass v undertake 0.00037561642594108100000

pass v retreat 0.00058983759879137900000

pass v report 0.00230879465517025000000

pass v decline 0.00026128377348592300000

pass v hap 0.00002390039441996750000

pass v approve 0.00043888230187876700000

pass v state 0.00262309234536906000000

pass v draw 0.00675828294367388000000

pass v overpass 0.00003712917212335310000

pass v dismiss 0.00002101684659533630000

pass v rise 0.00514026230132415000000

pass v adopt 0.00119324754463996000000

pass v exchange 0.00034827698901582800000

pass v stop 0.02581184175779920000000

pass v glide 0.01101808182760500000000

pass v grow 0.00047293791298150800000

pass v die 0.01989835774134260000000

pass v happen 0.03325075295941670000000

pass v accomplish 0.00082032862212911100000

pass v move 0.03861299350265640000000

pass v fade 0.00009200903405098160000

pass v discharge 0.01181120658793310000000

pass v forget 0.03337990730923240000000

pass v guide 0.00019526399311563200000

pass v carry 0.01174471853148230000000

pass v choke 0.00001365948467702470000

pass v melt 0.00000343670674597786000

pass v traverse 0.00149397719696340000000

pass v ford 0.00003539945968708450000

pass v throw 0.00072664052196391700000

pass v neglect 0.00752207270546786000000

pass v continue 0.00334614038448575000000

pass v overtake 0.00172630135946989000000

pass v issue 0.00010916150121617700000

pass v give 0.02302946190329500000000

pass v impart 0.00061383327049643800000

pass v crawl 0.00018834327910436000000

pass v overlook 0.00019913837128502300000

pass v tell 0.05790856774376080000000

pass v reach 0.01167122571200040000000

pass v perish 0.00001026644908449020000

pass v lead 0.00629644849290233000000

pass v develop 0.00009653031534875610000

pass v reject 0.00049869224725329700000

pass v suffice 0.00003077380791192330000

pass v convert 0.00000682974233851231000

pass v catch 0.00038510944477807500000

pass v empty 0.00067374151562773100000

pass v faint 0.00028558592600991800000

pass v drift 0.00002390039441996750000

pass v change 0.00949468063658346000000

pass v elapse 0.01093288706901800000000

pass v play 0.00537240925862740000000

pass v transfer 0.00008784504989443180000

pass v express 0.00095300553092908300000

pass v kill 0.01715997067141350000000

pass v close 0.01582716592505370000000

pass v omit 0.00000343670674597786000

pass v relinquish 0.00018241540888302900000

pass v offer 0.00201621710097750000000

pass v transform 0.00018807443052157300000

pass v drop 0.00309240234863221000000

pass v flit 0.00025077316868143200000

pass v dissolve 0.00002101684659533630000

pass v befall 0.00073876027579727300000

pass v fill 0.00057193588017883200000

pass v accept 0.00381569988955731000000

pass v expire 0.00047389599258516300000

pass v finish 0.00708374671417185000000

pass v dissipate 0.00068863011422531500000

pass v spend 0.01168304698103810000000

pass v shun 0.00009837573060361650000

pass v go 0.25435624921546900000000

pass v leave 0.05312814956374620000000

pass v present 0.00063824452422757900000

pass v fly 0.01886438432903940000000

pass v surpass 0.00001103463377827250000

pass v discontinue 0.00002101684659533630000

pass v engage 0.00083696470433797000000

pass v disregard 0.00005259227602675440000

pass v bide 0.00003539945968708450000

pass v promise 0.00142558501578620000000

pass v cross 0.00734283364547153000000

pass v recede 0.00367269394253502000000

pass v proceed 0.00216079319565914000000

pass v occur 0.00565961089089620000000

pass v sink 0.00126787340356707000000

pass v slight 0.00002101684659533630000

pass v ordain 0.00000682974233851231000

pass v end 0.00273147726789550000000

pass v cease 0.00315905801309400000000

pass v speak 0.02563447214996230000000

pass v fail 0.00469575514694326000000

pass v wane 0.00000343670674597786000

pass v turn 0.02074884532058770000000

pass v consign 0.00002101684659533630000

pass v rush 0.00187137882118092000000

pass v miss 0.00092028394090739100000

pass v occupy 0.00062598556451004000000

pass v advance 0.00143757095486716000000

pass v declare 0.00049626228738362000000

pass v succeed 0.00219194371821791000000

pass v refuse 0.00482180592782512000000

pass v prescribe 0.00002101684659533630000

pass v avoid 0.00128849373056643000000

pass v utter 0.00179330553401907000000

pass v trumpet 0.00000343670674597786000

pass v progress 0.00007108439888777450000

pass v arise 0.00047788355142369600000

pass v render 0.00044573414056678300000

pass v spurn 0.00002544591646098160000

pass v disappear 0.01326744473587470000000

feud n strife 0.14285714285714300000000

feud n contention 0.14285714285714300000000

feud n argument 0.14285714285714300000000

feud n fight 0.28571428571428600000000

feud n riot 0.14285714285714300000000

feud n fray 0.14285714285714300000000

executor n guardian 1.00000000000000000000000

embolden v cheer 1.00000000000000000000000

clearly r surely 0.01437173155317390000000

clearly r absolutely 0.10126877393402700000000

clearly r apparently 0.00047380898596706100000

clearly r plainly 0.04362603431748000000000

clearly r positively 0.00094761797193412600000

clearly r distinctly 0.75963973482295300000000

clearly r precisely 0.04822322568287000000000

clearly r obviously 0.00047380898596706100000

clearly r certainly 0.01757061196419450000000

clearly r decidedly 0.00045623415197789300000

clearly r simply 0.00189523594386825000000

clearly r evidently 0.01105318168558710000000

discernment n sense 1.00000000000000000000000

disapproval n negation 1.00000000000000000000000

guest n confidant 0.00252768084837748000000

guest n regular 0.00367768807038755000000

guest n fellow 0.00067078303895442700000

guest n patron 0.00156793173443488000000

guest n lodger 0.30332170180529700000000

guest n tenant 0.06635162226990870000000

guest n company 0.61020360900772900000000

guest n visitor 0.01167898322491130000000

demonstrate v try 0.50000000000000000000000

demonstrate v reveal 0.12500000000000000000000

demonstrate v strike 0.12500000000000000000000

demonstrate v settle 0.12500000000000000000000

demonstrate v determine 0.12500000000000000000000

roof n home 0.15471532940130100000000

roof n ceiling 0.02031732568897500000000

roof n cover 0.00193443833320381000000

roof n house 0.82221420651895300000000

roof n habitation 0.00081870005756718500000

cipher n nought 0.00140418793651341000000

cipher n obscurity 0.00140418793651341000000

cipher n figure 0.00140418793651341000000

cipher n blank 0.00140418793651341000000

cipher n trifle 0.00140418793651341000000

cipher n nobody 0.00421256380954026000000

cipher n nothing 0.95526905321008200000000

cipher n number 0.00561675174605369000000

cipher n sign 0.02788069155175730000000

chasm n hole 1.00000000000000000000000

cannonade n attack 1.00000000000000000000000

pace n rate 0.27453013432761400000000

pace n footstep 0.00021156778960678300000

pace n time 0.03554505304349600000000

pace n step 0.68262347316629000000000

pace n carriage 0.00021156778960678300000

pace n gallop 0.00322753415208648000000

pace n movement 0.00042313557921356800000

pace n yard 0.00322753415208648000000

batch n deal 1.00000000000000000000000

mark v deform 0.00392858848004193000000

mark v keep 0.30821562347965300000000

mark v make 0.00392858848004193000000

mark v show 0.01138488906461130000000

mark v enter 0.01138488906461130000000

mark v sign 0.01138488906461130000000

mark v remember 0.05149472899993420000000

mark v consider 0.00392858848004193000000

mark v observe 0.02276977812922260000000

mark v notice 0.13946489104148800000000

mark v form 0.01138488906461130000000

mark v recognize 0.01138488906461130000000

mark v mind 0.01138488906461130000000

mark v inscribe 0.01208360066785460000000

mark v see 0.38194768937401100000000

mark v determine 0.00392858848004193000000

allay v silence 1.00000000000000000000000

aggravation n affliction 1.00000000000000000000000

accountant n clerk 0.50000000000000000000000

accountant n inspector 0.50000000000000000000000

fight v push 0.00144241974867530000000

fight v clash 0.00091800793781585600000

fight v contest 0.00091800793781585600000

fight v charge 0.00318017467225185000000

fight v support 0.00087423073524952900000

fight v quarrel 0.01238460958381780000000

fight v conduct 0.00087423073524952900000

fight v meet 0.02037164030131810000000

fight v agitate 0.00056818901342577300000

fight v attack 0.01649640480057810000000

fight v encounter 0.00352776494897678000000

fight v resist 0.00883499687623260000000

fight v restrain 0.00091800793781585600000

fight v object 0.02733822748306070000000

fight v defend 0.03855772057376000000000

fight v persist 0.00397670683691597000000

fight v strive 0.30554364196971100000000

fight v traverse 0.00272401463866012000000

fight v argue 0.01051995116956980000000

fight v work 0.02243363678534140000000

fight v confound 0.01815110744374520000000

fight v stifle 0.00113637802685155000000

fight v contradict 0.00883499687623260000000

fight v insult 0.00805702796134294000000

fight v dispute 0.02120699261762090000000

fight v defy 0.00086352418832701900000

fight v persevere 0.00087423073524952900000

fight v engage 0.16893292440081800000000

fight v cross 0.00642956968015336000000

fight v uphold 0.00489471477473183000000

fight v press 0.26674548783191800000000

fight v force 0.00527622641947583000000

fight v withstand 0.00091800793781585600000

fight v beat 0.00527622641947583000000

well-being n success 0.98906439854192000000000

well-being n wealth 0.00364520048602673000000

well-being n happiness 0.00364520048602673000000

well-being n fortune 0.00364520048602673000000

mother n beginning 0.00884759706219625000000

mother n parent 0.28305743582053900000000

mother n stepmother 0.00084329760400690400000

mother n mamma 0.32078294710025200000000

mother n ma 0.01267053294557770000000

mother n mama 0.31927298875467600000000

mother n mum 0.03201752577105290000000

mother n superintendent 0.01749162449601420000000

mother n mother-in-law 0.00501605044568529000000

faith n intuition 0.00713810102177874000000

faith n conviction 0.00713810102177874000000

faith n principle 0.01325460770483390000000

faith n credit 0.00167183586627821000000

faith n expectation 0.00026905892122938200000

faith n belief 0.00268569263286452000000

faith n feeling 0.00833921257763161000000

faith n profession 0.00441663969247095000000

faith n trust 0.19934276519528100000000

faith n orthodoxy 0.00027764812590523100000

faith n religion 0.00611143943144612000000

faith n confession 0.00722774231512420000000

faith n certainty 0.00322381047532329000000

faith n hope 0.50958244485899500000000

faith n position 0.17162064400650100000000

faith n opinion 0.01565921608437120000000

faith n worship 0.01234255517593190000000

faith n persuasion 0.00026905892122938200000

faith n revelation 0.00026905892122938200000

faith n church 0.01029002794238600000000

faith n fidelity 0.00027764812590523100000

faith n doctrine 0.00055884696084418600000

faith n constancy 0.00082790588207356800000

faith n teaching 0.00713810102177874000000

faith n policy 0.00082790588207356800000

faith n confidence 0.00923993123473449000000

unprotected a open 1.00000000000000000000000

ungracious a rough 0.00814704894313200000000

ungracious a saucy 0.00814704894313200000000

ungracious a unpleasant 0.00814704894313200000000

ungracious a short 0.43549679805105700000000

ungracious a rude 0.18229022010257900000000

ungracious a insolent 0.17073886856535200000000

ungracious a impertinent 0.17888591750848400000000

ungracious a insulting 0.00814704894313200000000

unction n blessing 1.00000000000000000000000

trick v take 0.20000000000000000000000

trick v do 0.80000000000000000000000

dull a plain 0.02503487204313260000000

dull a empty 0.00129303007522261000000

dull a melancholy 0.01087584884683060000000

dull a gloomy 0.01966096150129440000000

dull a inactive 0.00057574523092842700000

dull a stupid 0.03563158209118080000000

dull a familiar 0.00057574523092842700000

dull a thick 0.00970604428544915000000

dull a slack 0.00970604428544915000000

dull a worn 0.00339414329832981000000

dull a dry 0.00286885798572076000000

dull a regular 0.01955927054279650000000

dull a faint 0.00104536809618334000000

dull a grave 0.01087584884683060000000

dull a broken 0.00057574523092842700000

dull a slow 0.00166690760554453000000

dull a ordinary 0.00219685855804020000000

dull a foolish 0.00381797188515198000000

dull a unsympathetic 0.00057574523092842700000

dull a indifferent 0.00104536809618334000000

dull a dirty 0.38338874927524100000000

dull a tired 0.01351188937255320000000

dull a ignorant 0.00144131108750091000000

dull a fatiguing 0.00104536809618334000000

dull a feeble 0.00162111332711177000000

dull a dismal 0.01087584884683060000000

dull a dreary 0.00104536809618334000000

dull a black 0.00144131108750091000000

dull a dead 0.24424790932918400000000

dull a dark 0.00486333998133532000000

dull a low 0.06598886107157360000000

dull a sober 0.00108320883023384000000

dull a old 0.00306242441461268000000

dull a sleepy 0.00909549521482268000000

dull a insensible 0.00115149046185686000000

dull a quiet 0.00793116263826234000000

dull a monotonous 0.00104536809618334000000

dull a tiresome 0.00104536809618334000000

dull a depressed 0.00057574523092842700000

dull a shallow 0.00175463958478371000000

dull a heavy 0.01179678047781580000000

dull a common 0.00219685855804020000000

dull a unfeeling 0.00057574523092842700000

dull a irksome 0.01111271737029690000000

dull a simple 0.03229529675112220000000

dull a drowsy 0.00983048075064722000000

dull a sad 0.01529418975503020000000

theology n scripture 1.00000000000000000000000

sublimity n importance 1.00000000000000000000000

space n place 0.42084292845354100000000

space n pause 0.00328089669786301000000

space n play 0.13902799757194500000000

space n course 0.01369953454495760000000

space n time 0.25861510044406500000000

space n air 0.01879012742084030000000

space n separation 0.00055694741140488700000

space n rest 0.00154475552857717000000

space n interruption 0.01823318000943540000000

space n age 0.11177934546896100000000

space n greatness 0.00055694741140488700000

space n infinite 0.00154475552857717000000

space n box 0.00614488387058320000000

space n length 0.00328089669786301000000

space n room 0.00055694741140488700000

space n land 0.00154475552857717000000

leaf n page 1.00000000000000000000000

self-willed a wild 1.00000000000000000000000

scoff v laugh 0.83333333333333400000000

scoff v tease 0.08333333333333300000000

scoff v sneer 0.08333333333333300000000

permit v have 0.59951341851722800000000

permit v stand 0.01175719508270510000000

permit v pass 0.00776399958165562000000

permit v support 0.00004778905934021860000

permit v endure 0.00662431438011225000000

permit v yield 0.00066469294683184900000

permit v brook 0.00015344188707535400000

permit v grant 0.01556729567627290000000

permit v sign 0.00189880863315994000000

permit v consent 0.11191972995014600000000

permit v bear 0.01274830050627950000000

permit v indulge 0.00004778905934021860000

permit v allow 0.04080867910269210000000

permit v favor 0.00015344188707535400000

permit v abide 0.00024612325179878100000

permit v suffer 0.00313432027460960000000

permit v accept 0.00305408009851954000000

permit v leave 0.06364411889077840000000

permit v admit 0.00455669544434155000000

permit v countenance 0.00139283385677038000000

permit v bless 0.00873463444806741000000

permit v order 0.00101869043018137000000

permit v let 0.10454960703501800000000

rebuild v reconstruct 1.00000000000000000000000

melancholy a funereal 0.00476019033649757000000

melancholy a miserable 0.10227040383225100000000

melancholy a unhappy 0.38162822121040500000000

melancholy a morose 0.00026786001893500900000

melancholy a ill-natured 0.00476019033649757000000

melancholy a bilious 0.00076578385781419600000

melancholy a dull 0.01593419488759540000000

melancholy a gloomy 0.03173842645728500000000

melancholy a sedate 0.00076578385781419600000

melancholy a unfortunate 0.00229735157344259000000

melancholy a grave 0.01516841102978120000000

melancholy a disagreeable 0.00382891928907099000000

melancholy a tired 0.02291186519451110000000

melancholy a thoughtful 0.01593419488759540000000

melancholy a earnest 0.00076578385781419600000

melancholy a dismal 0.01543627104871620000000

melancholy a dreary 0.00076578385781419600000

melancholy a distressed 0.00476019033649757000000

melancholy a grim 0.00076578385781419600000

melancholy a mournful 0.00076578385781419600000

melancholy a low 0.00206728775349841000000

melancholy a sorrowful 0.06608264231843690000000

melancholy a heavyhearted 0.00076578385781419600000

melancholy a sober 0.00026786001893500900000

melancholy a forlorn 0.05582405030983520000000

melancholy a quiet 0.00486256316582019000000

melancholy a pensive 0.06241138441185710000000

melancholy a heavy 0.00153156771562839000000

melancholy a serious 0.11048182909357800000000

melancholy a dreamy 0.06608264231843690000000

melancholy a sad 0.00333099545019180000000

query v inquire 0.01147687662308030000000

query v ask 0.98852312337692000000000

propitious a kind 0.05273682965946580000000

propitious a generous 0.04436875899534600000000

propitious a happy 0.20567160413187700000000

propitious a fit 0.00836807066411985000000

propitious a rosy 0.40808939959672900000000

propitious a suitable 0.04173090174235270000000

propitious a merciful 0.00836807066411985000000

propitious a advantageous 0.04173090174235270000000

propitious a favorable 0.00836807066411985000000

propitious a bright 0.04436875899534600000000

propitious a fair 0.04436875899534600000000

propitious a agreeable 0.09182987414882540000000

propitiate v yield 0.33333333333333300000000

propitiate v submit 0.33333333333333300000000

propitiate v please 0.33333333333333300000000

prejudiced a blind 0.14963348309293000000000

prejudiced a narrow 0.08394419484511710000000

prejudiced a interested 0.14963348309293000000000

prejudiced a unjust 0.07481674154646480000000

prejudiced a unreasonable 0.14963348309293000000000

prejudiced a partial 0.08394419484511710000000

prejudiced a inclined 0.22445022463939500000000

prejudiced a unfair 0.08394419484511710000000

plausible a acceptable 0.16666666666666700000000

plausible a superficial 0.16666666666666700000000

plausible a likely 0.66666666666666700000000

perpetrate v accomplish 0.06250000000000000000000

perpetrate v do 0.87500000000000000000000

perpetrate v produce 0.06250000000000000000000

pedigree n house 0.14494680851063800000000

pedigree n past 0.85505319148936200000000

passively r quietly 1.00000000000000000000000

year n period 0.01278153610017620000000

year n space 0.00053832213848364500000

year n class 0.00854162320108955000000

year n century 0.06565271284467170000000

year n time 0.78872806731290100000000

year n generation 0.01117198357089800000000

year n term 0.00164114250867516000000

year n spell 0.00122300369464570000000

year n age 0.06410824563315490000000

year n lifetime 0.00034770036779517800000

year n twelvemonth 0.04526566262750860000000

smoke n fog 0.82653265747329700000000

smoke n bullet 0.00824985809081267000000

smoke n cigarette 0.15696762634507700000000

smoke n vapor 0.00824985809081267000000

safe a reliable 0.00006751869446375410000

safe a honest 0.01620495119907420000000

safe a discreet 0.00056840326982824400000

safe a innocent 0.01025993132031120000000

safe a impregnable 0.00055739282372123200000

safe a secret 0.00055739282372123200000

safe a invulnerable 0.00006751869446375410000

safe a pure 0.00864628820065656000000

safe a careful 0.00606772471070013000000

safe a sure 0.20353619404612400000000

safe a inviolable 0.00006751869446375410000

safe a faithful 0.00447333236163607000000

safe a timid 0.00030648056334978900000

safe a true 0.31589352012058400000000

safe a sound 0.00786417163840840000000

safe a satisfactory 0.00267108924716224000000

safe a secure 0.01221905886835950000000

safe a responsible 0.00811699009713988000000

safe a healthy 0.22487101191153400000000

safe a fine 0.01101023966747830000000

safe a good 0.11289655689076300000000

safe a upright 0.00096793753807134800000

safe a prudent 0.00779665294394465000000

safe a cautious 0.00061296112669957900000

safe a considerate 0.00298799336498878000000

safe a clear 0.02333087075532200000000

safe a powerless 0.01369481358270860000000

safe a competent 0.00006751869446375410000

safe a trustworthy 0.00161079210327660000000

safe a inoffensive 0.00200717404658099000000

dance v sway 0.99391605698883300000000

dance v prance 0.00608394301116670000000

martyrdom n hell 1.00000000000000000000000

cry n hubbub 0.34749560042654800000000

cry n prayer 0.01076836941091920000000

cry n voice 0.08394460960559440000000

cry n cuckoo 0.00277996480341239000000

cry n password 0.00242157212840800000000

cry n request 0.00132159107966215000000

cry n signal 0.00053850240264457800000

cry n sob 0.23408530574610700000000

cry n scream 0.00078308867701757200000

cry n roar 0.23166373361769900000000

cry n calling 0.02106033941979080000000

cry n hey 0.00053850240264457800000

cry n shriek 0.04166492402131740000000

cry n groan 0.00053850240264457800000

cry n crow 0.00053850240264457800000

cry n halloa 0.01985689145294560000000

juvenile a young 1.00000000000000000000000

group n meeting 0.08374479031255700000000

group n lot 0.03034231533063660000000

group n detail 0.00032444130758002700000

group n family 0.37717599771408700000000

group n sort 0.03066675663821660000000

group n order 0.00064888261516005600000

group n kind 0.00097332392274008500000

group n pile 0.00032444130758002700000

group n league 0.00032444130758002700000

group n knot 0.00032444130758002700000

group n packet 0.00032444130758002700000

group n company 0.00064888261516005600000

group n party 0.25253213616799100000000

group n gang 0.19032906889217500000000

group n crowd 0.03066675663821660000000

group n school 0.00064888261516005600000

indignity n wound 1.00000000000000000000000

courage n heart 0.52012584274949800000000

courage n resolution 0.00189866301187187000000

courage n spirit 0.02539375310423970000000

courage n will 0.00241302289641002000000

courage n enterprise 0.00012525490549310600000

courage n blood 0.00379297900297669000000

courage n audacity 0.40156722701090100000000

courage n confidence 0.04468325731861010000000

impartial a equal 0.07071713147410360000000

impartial a true 0.69023904382470100000000

impartial a right 0.11952191235059800000000

impartial a fair 0.11952191235059800000000

gratifying a nice 0.02584814216478190000000

gratifying a delightful 0.00323101777059773000000

gratifying a lovely 0.00323101777059773000000

gratifying a fine 0.01615508885298870000000

gratifying a good 0.88691437802907900000000

gratifying a great 0.02584814216478190000000

gratifying a sweet 0.00323101777059773000000

gratifying a pleasant 0.01615508885298870000000

gratifying a agreeable 0.01938610662358640000000

introduce v create 0.00856838393953878000000

introduce v suggest 0.00301988505503682000000

introduce v initiate 0.00018014322744187200000

introduce v advocate 0.00057204279996920800000

introduce v announce 0.01405039955879430000000

introduce v acquaint 0.00829351900557649000000

introduce v shoot 0.00699511484506838000000

introduce v instigate 0.00057204279996920800000

introduce v enter 0.00138151904270019000000

introduce v open 0.00249695953497865000000

introduce v add 0.00488708687263341000000

introduce v embed 0.00057204279996920800000

introduce v feed 0.00375159891019681000000

introduce v carry 0.00765080576381818000000

introduce v mention 0.18398283685427900000000

introduce v start 0.00526046673319587000000

introduce v explain 0.10488103118527800000000

introduce v insinuate 0.00591178299309441000000

introduce v originate 0.00018014322744187200000

introduce v present 0.00378933582574588000000

introduce v admit 0.01342942850240780000000

introduce v begin 0.14413486170332900000000

introduce v advance 0.00131722312186959000000

introduce v force 0.09454872678979510000000

introduce v put 0.23490987128004100000000

introduce v propose 0.14466274762783100000000

enact v do 0.83333333333333300000000

enact v present 0.16666666666666700000000

one n person 0.23509276857865800000000

one n example 0.00147288318194781000000

one n individual 0.01638515644820810000000

one n identity 0.01182051829098140000000

one n thing 0.67825703698801600000000

one n item 0.02015497330974510000000

one n whole 0.03681666320244360000000

temper n disposition 0.57478387704235300000000

temper n humor 0.23205407399995900000000

temper n agitation 0.00140899505464630000000

temper n mood 0.02028726078681880000000

temper n sullenness 0.00140899505464630000000

temper n rage 0.00290794919227947000000

temper n fury 0.00290794919227947000000

temper n pet 0.00290794919227947000000

temper n spirit 0.13337407916608400000000

temper n type 0.00281799010929260000000

temper n nature 0.01136191952015730000000

temper n character 0.00796306330464545000000

temper n condition 0.00290794919227947000000

temper n passion 0.00290794919227947000000

economical a careful 0.40000000000000000000000

economical a reasonable 0.20000000000000000000000

economical a practical 0.10000000000000000000000

economical a prudent 0.30000000000000000000000

dog v haunt 1.00000000000000000000000

venture v guess 0.29727299031070000000000

venture v pretend 0.01022324292040420000000

venture v try 0.01290344216435750000000

venture v risk 0.00435246975819188000000

venture v dare 0.48537794515957000000000

venture v offer 0.06763068393498150000000

venture v go 0.12223922575179400000000

discreet a safe 0.14285714285714300000000

discreet a careful 0.28571428571428600000000

discreet a modest 0.14285714285714300000000

discreet a attentive 0.14285714285714300000000

discreet a sensitive 0.14285714285714300000000

discreet a serious 0.14285714285714300000000

destructive a harmful 0.02050695503489060000000

destructive a dreadful 0.17437266047414400000000

destructive a wicked 0.09743980775451720000000

destructive a evil 0.02050695503489060000000

destructive a fatal 0.07693285271962660000000

destructive a contrary 0.07693285271962660000000

destructive a bad 0.43586810850778700000000

destructive a unwholesome 0.02050695503489060000000

destructive a mischievous 0.07693285271962660000000

deputation n delegates 0.19695688926458100000000

deputation n appointment 0.30304311073541900000000

deputation n commission 0.19695688926458100000000

deputation n committee 0.30304311073541900000000

depose v can 0.99407846039970400000000

depose v fire 0.00592153960029607000000

decrease v fall 0.00691358024691358000000

decrease v deteriorate 0.98617283950617300000000

decrease v restrain 0.00345679012345679000000

decrease v drop 0.00345679012345679000000

swear v confess 0.00429360253204149000000

swear v admire 0.00045224490461285300000

swear v know 0.65259212556181900000000

swear v demonstrate 0.00030301814802612100000

swear v own 0.00350204027510192000000

swear v respect 0.00142941098295369000000

swear v insist 0.00086954677537127600000

swear v acknowledge 0.00179113182308788000000

swear v maintain 0.00016745859509477700000

swear v confirm 0.00422320390561064000000

swear v state 0.00154806304794321000000

swear v blaspheme 0.00016745859509477700000

swear v assert 0.13938834809201600000000

swear v vow 0.00082845972120068700000

swear v curse 0.00013959774470907900000

swear v affirm 0.00041422986060034300000

swear v prove 0.00399367986468893000000

swear v damn 0.00176327097270218000000

swear v promise 0.01461109838595580000000

swear v pledge 0.00030301814802612100000

swear v warrant 0.00352881893903837000000

swear v admit 0.14444002467197300000000

swear v declare 0.00415303095539352000000

swear v trust 0.01509711749693850000000

station n work 0.02220210070185310000000

station n place 0.28187095854663800000000

station n estate 0.00172990969738933000000

station n task 0.00345981939477866000000

station n bag 0.02047219100446380000000

station n window 0.04117185079786610000000

station n table 0.07410933143615900000000

station n point 0.02393201039924250000000

station n business 0.02007380361723070000000

station n order 0.02963777316421630000000

station n step 0.01372395026595540000000

station n philosophy 0.37429712941946700000000

station n position 0.02047219100446380000000

station n duty 0.00172990969738933000000

station n purpose 0.00345981939477866000000

station n view 0.00917194695992067000000

station n thing 0.03460212142747180000000

station n condition 0.01667289143726470000000

station n spot 0.00548038193606134000000

station n degree 0.00172990969738933000000

consistency n attachment 1.00000000000000000000000

collapse n bankruptcy 0.33333333333333300000000

collapse n calamity 0.66666666666666700000000

chuck v leave 0.57142857142857100000000

chuck v cast 0.42857142857142900000000

sharp a quick 0.00104379078280862000000

sharp a close 0.00171443119014680000000

sharp a bent 0.02579559544559340000000

sharp a ungracious 0.00057147706338226500000

sharp a diligent 0.00057147706338226500000

sharp a clever 0.00285738531691133000000

sharp a painful 0.00218674490957315000000

sharp a cruel 0.00293304566051405000000

sharp a bitter 0.00114295412676453000000

sharp a unmannerly 0.00047231371942635700000

sharp a piquant 0.00057147706338226500000

sharp a violent 0.11976526456882600000000

sharp a active 0.00057147706338226500000

sharp a severe 0.00218674490957315000000

sharp a earnest 0.00057147706338226500000

sharp a distinct 0.08383568519817840000000

sharp a fierce 0.00094462743885271400000

sharp a attentive 0.00275822197295542000000

sharp a intelligent 0.00882480896822931000000

sharp a keen 0.00057147706338226500000

sharp a mean 0.00383920870730501000000

sharp a ready 0.00188925487770543000000

sharp a smart 0.00057147706338226500000

sharp a rude 0.00208758156561725000000

sharp a stiff 0.00047231371942635700000

sharp a sensitive 0.00114295412676453000000

sharp a critical 0.41917842599089200000000

sharp a obvious 0.00094462743885271400000

sharp a fine 0.03755926109492410000000

sharp a animated 0.00047231371942635700000

sharp a sore 0.16918747489859200000000

sharp a cold 0.01122575615480260000000

sharp a distressing 0.00114295412676453000000

sharp a energetic 0.00057147706338226500000

sharp a clear 0.00161526784619089000000

sharp a insulting 0.00057147706338226500000

sharp a hard 0.08659390717113390000000

sharp a hot 0.00104379078280862000000

buffer n guard 0.33333333333333300000000

buffer n wall 0.66666666666666700000000

innocent a honest 0.14185271051916100000000

innocent a blameless 0.00046480768749625200000

innocent a unacquainted 0.03693108353379310000000

innocent a plain 0.20468946105312700000000

innocent a chaste 0.00046480768749625200000

innocent a safe 0.00927035013426302000000

innocent a faultless 0.01009720137164590000000

innocent a ignorant 0.14288295165976700000000

innocent a right 0.06506010507266450000000

innocent a sincere 0.00757507859372827000000

innocent a decent 0.00105380523702133000000

innocent a harmless 0.00700417101502972000000

innocent a open 0.00066344830250927500000

innocent a free 0.03150309456934260000000

innocent a good 0.05666898829258090000000

innocent a virtuous 0.00018886188696965200000

innocent a frank 0.00046910152294656300000

innocent a natural 0.00286305802192128000000

innocent a clear 0.07411917109755210000000

innocent a candid 0.00105380523702133000000

innocent a unaffected 0.20312095943586200000000

innocent a simple 0.00171725353953061000000

innocent a spotless 0.00018886188696965200000

innocent a green 0.00009686264160031560000

inn n bar 0.00146692802840108000000

inn n hotel 0.97277974103281300000000

inn n lodging 0.02575333093878600000000

dream v create 0.00041225514499386500000

dream v believe 0.23516662909451500000000

dream v wish 0.08703886201595410000000

dream v think 0.58608979864779200000000

dream v suppose 0.03838879010837210000000

dream v conceive 0.00136398776675896000000

dream v hope 0.00979336713270536000000

dream v long 0.00043997473271162100000

dream v consider 0.03135978620954060000000

dream v imagine 0.00874159779858487000000

dream v fancy 0.00120495134807077000000

auntie n aunty 0.19999999999999900000000

auntie n aunt 0.80000000000000000000000

annoying a disagreeable 0.07303123625164980000000

annoying a unpleasant 0.03651561812582490000000

annoying a intolerable 0.03651561812582490000000

annoying a hateful 0.03651561812582490000000

annoying a unbearable 0.78090629124505100000000

annoying a irksome 0.03651561812582490000000

display v betray 0.01283031771881730000000

display v show 0.74769782568280000000000

display v extend 0.01283031771881730000000

display v open 0.00641515885940862000000

display v dispose 0.08569583043026710000000

display v boast 0.01924547657822590000000

display v publish 0.08569583043026710000000

display v present 0.02958924258139630000000

acclamation n congratulation 1.00000000000000000000000

couple n pair 0.99583023082650800000000

couple n match 0.00416976917349216000000

20 n xx 1.00000000000000000000000

most r extremely 0.02635502157790540000000

most r almost 0.00052360970022328700000

most r very 0.94571912774351900000000

most r too 0.02740224097835200000000

regiment n corps 1.00000000000000000000000

past a over 0.00701812949771975000000

past a immemorial 0.00701812949771975000000

past a previous 0.03223581515054330000000

past a latter 0.00125871151150367000000

past a unfashionable 0.00148703134783585000000

past a older 0.00148703134783585000000

past a old 0.10376184714849500000000

past a earlier 0.01940727646818420000000

past a late 0.00117893264768762000000

past a former 0.01940727646818420000000

past a senior 0.00029473316192190300000

past a extinct 0.00204726920762331000000

past a last 0.78369580691463900000000

past a early 0.01970200963010610000000

unsuitable a amiss 0.75000000000000000000000

unsuitable a difficult 0.25000000000000000000000

unreserved a complete 0.28571428571428600000000

unreserved a full 0.14285714285714300000000

unreserved a free 0.14285714285714300000000

unreserved a candid 0.14285714285714300000000

unreserved a simple 0.14285714285714300000000

unreserved a straightforward 0.14285714285714300000000

unravel v have 0.50000000000000000000000

unravel v define 0.25000000000000000000000

unravel v solve 0.25000000000000000000000

unprincipled a wicked 0.16666666666666700000000

unprincipled a bad 0.50000000000000000000000

unprincipled a unjust 0.16666666666666700000000

unprincipled a treacherous 0.16666666666666700000000

maid n child 0.91829526550414600000000

maid n girl 0.08096978117414410000000

maid n barmaid 0.00036747666085520200000

maid n maiden 0.00036747666085520200000

typical a demonstrative 0.16666666666666700000000

typical a regular 0.16666666666666700000000

typical a natural 0.66666666666666700000000

interval n stoppage 0.00233921174952644000000

interval n parenthesis 0.00916027458217918000000

interval n period 0.10219431330743700000000

interval n separation 0.86759250636464600000000

interval n rest 0.00233921174952644000000

interval n interruption 0.00467842349905288000000

interval n spell 0.00467842349905288000000

interval n meanwhile 0.00467842349905288000000

interval n vacation 0.00233921174952644000000

comfortable a delightful 0.06510357555746270000000

comfortable a cheerful 0.00337335029871699000000

comfortable a pleased 0.00522084472906882000000

comfortable a useful 0.00484751409080029000000

comfortable a sufficient 0.00744684016611968000000

comfortable a amiable 0.02057274430025370000000

comfortable a loose 0.00012311699719410500000

comfortable a satisfied 0.04726844930672540000000

comfortable a happy 0.40140159166928500000000

comfortable a content 0.00778931951187746000000

comfortable a contented 0.01078027440231960000000

comfortable a convenient 0.00343049993009414000000

comfortable a satisfactory 0.00334603785917606000000

comfortable a healthy 0.00134916226671725000000

comfortable a serene 0.00459233516123446000000

comfortable a suitable 0.00029827075495328000000

comfortable a cordial 0.00029827075495328000000

comfortable a quiet 0.09405183987145540000000

comfortable a placid 0.00067138646441709300000

comfortable a prosperous 0.00029827075495328000000

comfortable a acceptable 0.00042138775214738500000

comfortable a easy 0.00976427954478323000000

comfortable a rich 0.01080926521771290000000

comfortable a pleasant 0.00975281924734893000000

comfortable a calm 0.07322571559631350000000

comfortable a agreeable 0.10490259631528200000000

comfortable a warm 0.10886024147863400000000

tally n amount 1.00000000000000000000000

spread v teach 0.00120092182193810000000

spread v stretch 0.48981598044115100000000

spread v preserve 0.00137691898549799000000

spread v set 0.31944520463553400000000

spread v show 0.04259269395140450000000

spread v open 0.03549391162617040000000

spread v feed 0.00413075695649397000000

spread v throw 0.04259269395140450000000

spread v reach 0.02777784388135070000000

spread v repeat 0.00120092182193810000000

spread v settle 0.00137691898549799000000

spread v prepare 0.03041739213418290000000

spread v advance 0.00120092182193810000000

spread v feast 0.00137691898549799000000

skirmish n battle 1.00000000000000000000000

owner n partner 0.01471957498869640000000

owner n landlady 0.00693739170552719000000

owner n lord 0.07136928083890890000000

owner n master 0.14152507421548600000000

owner n proprietor 0.00668006541760630000000

owner n heiress 0.00668006541760630000000

owner n mistress 0.03193439110855390000000

owner n lady 0.72015415630761500000000

own v have 0.33232356774638400000000

own v confess 0.02905283379217530000000

own v keep 0.09539597934790750000000

own v hold 0.00742051694006772000000

own v acknowledge 0.00157184998986710000000

own v reveal 0.00022625426932983900000

own v maintain 0.00529368436521770000000

own v use 0.00023408604485656300000

own v grant 0.00016719190986622600000

own v tell 0.30178652667967400000000

own v allow 0.00798943818166869000000

own v control 0.00016719190986622600000

own v boast 0.00038955819298213100000

own v profess 0.00043455934819092300000

own v express 0.00086551450544928700000

own v agree 0.01170632407138850000000

own v inherit 0.00035610700032388200000

own v accept 0.00254337741886877000000

own v recognize 0.01515415522535020000000

own v admit 0.00416771668904741000000

own v possess 0.00095397027216709800000

own v see 0.17888406359304500000000

own v occupy 0.00012985273099404300000

own v declare 0.00097027602857336100000

own v utter 0.00010423331386252000000

own v enjoy 0.00171117043287706000000

rate v blame 1.00000000000000000000000

darling n friend 0.30272381435684300000000

darling n baby 0.01136069838188700000000

darling n cheri 0.00033651461511075700000

darling n lover 0.01822270175360590000000

darling n treasure 0.00085921254447173600000

darling n love 0.36571500084033800000000

darling n flame 0.00048604859110005400000

darling n pearl 0.03514487258328710000000

darling n dear 0.05068873907562760000000

darling n hero 0.00065323685331066700000

darling n pet 0.00016718826221061300000

darling n favorite 0.00050156478663184100000

darling n jewel 0.01348582320056360000000

darling n dearest 0.12783884305680300000000

darling n sugar 0.00048604859110005400000

darling n lamb 0.00048604859110005400000

darling n princess 0.01811438997348100000000

darling n heroine 0.00865927477325984000000

darling n babe 0.00033437652442122700000

darling n idol 0.00653858094572783000000

darling n sweet 0.00138404856452225000000

darling n angel 0.03002669187215040000000

darling n sweetheart 0.00578628126244547000000

questionable a evil 1.00000000000000000000000

query n request 0.01737022318862300000000

query n question 0.59553622753742800000000

query n poser 0.01737022318862300000000

query n doubt 0.33498287970808000000000

query n reservation 0.01737022318862300000000

query n inquiry 0.01737022318862300000000

precarious a vain 0.18181818181818200000000

precarious a delicate 0.09090909090909090000000

precarious a infirm 0.09090909090909090000000

precarious a dangerous 0.18181818181818200000000

precarious a doubtful 0.09090909090909090000000

precarious a suspicious 0.09090909090909090000000

precarious a uncertain 0.09090909090909090000000

precarious a sensitive 0.18181818181818200000000

bring v gather 0.00575595684052389000000

bring v fetch 0.01288963290307510000000

bring v persuade 0.00379373634933857000000

bring v invite 0.00511858010416773000000

bring v return 0.03595863251540740000000

bring v support 0.00008251244874605330000

bring v deliver 0.00298642653918263000000

bring v realize 0.00289324235541127000000

bring v make 0.09956539546523610000000

bring v prefer 0.00154270200227024000000

bring v afford 0.00034247761036710700000

bring v attract 0.00038181621609601800000

bring v take 0.23235877775165000000000

bring v conduct 0.00005416801349001700000

bring v bestow 0.00256750193312567000000

bring v command 0.00016296116901561100000

bring v send 0.11978308961037300000000

bring v convey 0.00047492616865260000000

bring v encourage 0.00137321516234211000000

bring v draw 0.03318185790926500000000

bring v dispose 0.00005416801349001700000

bring v bear 0.02749289624282780000000

bring v lend 0.00065849463355690000000

bring v add 0.00278665370026144000000

bring v move 0.03502996548984240000000

bring v guide 0.00000682326621345936000

bring v carry 0.01238209354515990000000

bring v procure 0.00005416801349001700000

bring v work 0.01119958863775270000000

bring v earn 0.00022571273515644100000

bring v get 0.16648052213897700000000

bring v pack 0.01135903242885650000000

bring v impart 0.00040387670858156700000

bring v lead 0.02926243183612370000000

bring v provoke 0.00006997522260565880000

bring v land 0.00009100877258973720000

bring v appeal 0.00023789419698174000000

bring v influence 0.00047329301786902200000

bring v effect 0.00010833602698003400000

bring v play 0.00807972488059000000000

bring v transfer 0.00009921380516040390000

bring v prompt 0.00007104946007103400000

bring v court 0.00000682326621345936000

bring v produce 0.00021727549892820600000

bring v pull 0.02561845417285460000000

bring v serve 0.00608885245497450000000

bring v cause 0.00005416801349001700000

bring v begin 0.02392238228064120000000

bring v ride 0.03157245921833900000000

bring v attend 0.00095803016312077700000

bring v force 0.03680625893151210000000

bring v declare 0.00111019848455298000000

bring v precipitate 0.00007104946007103400000

bring v lure 0.00567951621442827000000

pipe v shriek 1.00000000000000000000000

pinion v bind 1.00000000000000000000000

pantaloons n trousers 1.00000000000000000000000

finally r absolutely 0.11946673429464300000000

finally r certainly 0.49005603404265400000000

finally r lastly 0.39047723166270200000000

obediently r devotedly 1.00000000000000000000000

fate n future 0.00046200246976584000000

fate n necessity 0.00061123918886274900000

fate n sentence 0.00113941713402373000000

fate n misfortune 0.00184800987906337000000

fate n end 0.00718304009142460000000

fate n luck 0.00046200246976584000000

fate n destiny 0.00082665138335480000000

fate n providence 0.06550014368298860000000

fate n death 0.20104918252961900000000

fate n predestination 0.00014923671909691000000

fate n certainty 0.00122247837772550000000

fate n destruction 0.00046200246976584000000

fate n circumstance 0.00402648775808276000000

fate n chance 0.66358478330284600000000

fate n destination 0.01075172196913140000000

fate n doom 0.00092400493953168200000

fate n effect 0.01645149898912020000000

fate n fortune 0.02334609664583070000000

merciless a relentless 0.91512513601741000000000

merciless a hard 0.08487486398258970000000

considerable a respectable 0.00137754846465962000000

considerable a large 0.60749887291489300000000

considerable a distinguished 0.09467514902569760000000

considerable a great 0.29507088113009100000000

considerable a pretty 0.00137754846465962000000

ledger n book 1.00000000000000000000000

imp n goblin 0.33333333333333300000000

imp n rascal 0.66666666666666700000000

relate v associate 0.00005880614901277730000

relate v respect 0.00030535725692146400000

relate v refer 0.00234734387024139000000

relate v understand 0.08917407301364210000000

relate v affect 0.00006107145138429270000

relate v tie 0.00005880614901277730000

relate v connect 0.01578348345144050000000

relate v communicate 0.00005880614901277730000

relate v comprehend 0.00664864534070336000000

relate v report 0.00210532336707573000000

relate v state 0.00216639481846002000000

relate v grasp 0.00006107145138429270000

relate v unite 0.00104983953425224000000

relate v respond 0.00801102386735683000000

relate v marry 0.03418265229985940000000

relate v tell 0.16214817467188400000000

relate v repeat 0.10419426575089500000000

relate v mention 0.00634820531944727000000

relate v touch 0.00096808201266262500000

relate v say 0.34847045966499100000000

relate v disclose 0.00051098876595975900000

relate v express 0.00222746626984432000000

relate v articulate 0.00005880614901277730000

relate v present 0.00031225178895715100000

relate v sympathize 0.00006107145138429270000

relate v regard 0.00006107145138429270000

relate v join 0.00012214290276858600000

relate v concern 0.01378776717401290000000

relate v speak 0.02902422332377450000000

relate v bind 0.00071458055889796700000

relate v describe 0.03838769581566860000000

relate v utter 0.13053004875869500000000

harmonious a amiable 0.33333333333333300000000

harmonious a agreeable 0.66666666666666700000000

harbor v have 1.00000000000000000000000

part v quit 0.02632121326606260000000

part v retire 0.03900857352553850000000

part v withdraw 0.00660474590992278000000

part v depart 0.05983242741868970000000

part v abandon 0.00455876345753979000000

part v separate 0.05193880760672930000000

part v divide 0.00070296273059988000000

part v dismiss 0.01400488071535860000000

part v release 0.00838894054312655000000

part v deal 0.01337436806589890000000

part v break 0.01885368562489620000000

part v start 0.03190760441297480000000

part v detach 0.00563979376272845000000

part v administer 0.00055806256831957700000

part v relinquish 0.00775004772030565000000

part v dissolve 0.00027903128415978800000

part v assign 0.00027903128415978800000

part v surrender 0.00337795944968380000000

part v go 0.68422527939482000000000

part v dispense 0.01365339935005870000000

part v loose 0.00035148136529993900000

part v vacate 0.00838894054312655000000

frowning a dark 0.50000000000000000000000

frowning a angry 0.50000000000000000000000

bridge n scaffold 1.00000000000000000000000

faultless a innocent 0.96394019349164500000000

faultless a exquisite 0.01802990325417760000000

faultless a perfect 0.01201993550278510000000

faultless a spotless 0.00600996775139252000000

return v come 0.21016227108654100000000

return v reflect 0.00132222922473036000000

return v revisit 0.00017014046501772100000

return v ring 0.01461075136373970000000

return v recompense 0.00008248712828288270000

return v retire 0.00988185706660338000000

return v pass 0.03574600887794790000000

return v announce 0.01283202557482870000000

return v sound 0.00000611933343921667000

return v deliver 0.00006618886623772180000

return v remit 0.00012109551670652000000

return v yield 0.00012440978436468600000

return v make 0.02108211086038580000000

return v fall 0.01571545445966740000000

return v restore 0.00283701986094592000000

return v reply 0.29489291996649600000000

return v redress 0.00008858400298205220000

return v answer 0.07778641966089030000000

return v requite 0.00008858400298205220000

return v pronounce 0.00026717847899056600000

return v retreat 0.00309096241652120000000

return v report 0.00082784571430770600000

return v state 0.00026440990901368400000

return v retaliate 0.00019450425475772900000

return v replace 0.00010814397382916200000

return v exchange 0.00029210495606985100000

return v release 0.00058439351624579200000

return v respond 0.00086889862232936800000

return v revive 0.00101109457931055000000

return v pay 0.00674329562526424000000

return v renew 0.00082610419427603900000

return v retort 0.02873626777130530000000

return v echo 0.00698731483604264000000

return v rejoin 0.01981721735103330000000

return v earn 0.00074088438857716400000

return v give 0.10340655083238600000000

return v repeat 0.03513845456293060000000

return v reappear 0.00612745539243989000000

return v repay 0.00127282350826542000000

return v recur 0.02037432068587200000000

return v recoil 0.00045529208236585500000

return v avenge 0.00025790279349205100000

return v recede 0.00145530862041943000000

return v revenge 0.00002087532857159020000

return v reverse 0.00020073222350612800000

return v wane 0.00006990565425595510000

return v turn 0.04313869687817720000000

return v recall 0.00074554416159751800000

return v render 0.00173398473922315000000

return v recover 0.01662485484583310000000

page n period 0.00579230346546036000000

page n leaf 0.38244203655586000000000

page n side 0.00244493971991633000000

page n boy 0.00017708308745766000000

page n stage 0.00017708308745766000000

page n matter 0.01622916260212600000000

page n time 0.50345940088487100000000

page n chapter 0.00579230346546036000000

page n incident 0.06936448702238630000000

page n point 0.00247916322440726000000

page n signature 0.00035416617491532200000

page n servant 0.00514140106930646000000

page n age 0.00017708308745766000000

page n affair 0.00579230346546036000000

page n event 0.00017708308745766000000

extract n note 1.00000000000000000000000

exactness n precision 1.00000000000000000000000

nurse n sister 0.27555291210438000000000

nurse n caretaker 0.00190788455773675000000

nurse n governess 0.01671009832850660000000

nurse n attendant 0.00285298659644712000000

nurse n servant 0.70297611841293000000000

commit v swear 0.00126892617209974000000

commit v charge 0.01961773780707190000000

commit v deliver 0.00054792135837995400000

commit v conclude 0.00054792135837995400000

commit v make 0.43968248913698800000000

commit v lay 0.00220654961355887000000

commit v employ 0.00054792135837995400000

commit v delegate 0.00054792135837995400000

commit v send 0.03837457151565590000000

commit v devote 0.00164376407513986000000

commit v complete 0.00054792135837995400000

commit v submit 0.02103410215931200000000

commit v confine 0.00123388039070768000000

commit v accomplish 0.00164376407513986000000

commit v discharge 0.00109584271675991000000

commit v execute 0.00191983942303541000000

commit v continue 0.00082931412758945400000

commit v do 0.42051225758768700000000

commit v give 0.00826480535919255000000

commit v effect 0.00109584271675991000000

commit v risk 0.00014335509526172900000

commit v practice 0.00083463154890341400000

commit v transfer 0.00168969277723936000000

commit v place 0.00219168543351982000000

commit v save 0.00300112231922953000000

commit v settle 0.00096203438022326900000

commit v assign 0.00054792135837995400000

commit v assure 0.00264616165806915000000

commit v produce 0.00082399670627549700000

commit v entrust 0.00109584271675991000000

commit v surrender 0.00109584271675991000000

commit v choose 0.00082931412758945400000

commit v oblige 0.00054792135837995400000

commit v pull 0.00069127645364168300000

commit v engage 0.00055215069579108600000

commit v promise 0.00043006528578518900000

commit v remove 0.00469026265891610000000

commit v decide 0.00316435465637100000000

commit v imprison 0.00109584271675991000000

commit v pursue 0.00068595903232772500000

commit v act 0.00109584271675991000000

commit v consign 0.00054792135837995400000

commit v determine 0.00013803767394777100000

commit v bind 0.00265662679427229000000

commit v put 0.00083463154890341400000

commit v intrust 0.00054792135837995400000

commit v resolve 0.00013803767394777100000

commit v trust 0.00178633544597203000000

commit v perform 0.00137191806465545000000

diligent a busy 0.33333333333333300000000

diligent a active 0.06666666666666660000000

diligent a careful 0.13333333333333300000000

diligent a earnest 0.06666666666666660000000

diligent a attentive 0.26666666666666700000000

diligent a keen 0.06666666666666660000000

diligent a intent 0.06666666666666660000000

derange v trouble 1.00000000000000000000000

remind v recollect 0.00180485050767706000000

remind v remember 0.08514880739873200000000

remind v revive 0.00029327677281361600000

remind v tell 0.85089949060825300000000

remind v mention 0.02576222153248030000000

remind v intimate 0.01365685335780330000000

remind v recall 0.02243449982224100000000

contest v clash 0.10000000000000000000000

contest v fight 0.10000000000000000000000

contest v quarrel 0.10000000000000000000000

contest v enter 0.20000000000000000000000

contest v strike 0.10000000000000000000000

contest v withstand 0.10000000000000000000000

contest v doubt 0.30000000000000000000000

conclusive a obvious 0.86450381679389300000000

conclusive a last 0.13549618320610700000000

commune v discourse 0.50000000000000000000000

commune v talk 0.50000000000000000000000

leave n liberty 0.06751543102398080000000

leave n departure 0.47510858868727200000000

leave n consent 0.00708049529158206000000

leave n holiday 0.08671982029302420000000

leave n farewell 0.14711695772509400000000

leave n rest 0.07459592631556290000000

leave n trip 0.14186278066348400000000

gloomy a miserable 0.09137800943647950000000

gloomy a unhappy 0.02351998299034370000000

gloomy a low-spirited 0.00178519046934631000000

gloomy a discontented 0.00480108042892378000000

gloomy a cheerless 0.00178519046934631000000

gloomy a dull 0.04062452670627820000000

gloomy a melancholy 0.04476122785913680000000

gloomy a painful 0.01404300584226030000000

gloomy a grave 0.04062452670627820000000

gloomy a dismal 0.09547510550363350000000

gloomy a dreary 0.00178519046934631000000

gloomy a dead 0.06268553124056710000000

gloomy a dark 0.08695756733578990000000

gloomy a bad 0.17016222424856800000000

gloomy a cold 0.09257325540379630000000

gloomy a distressing 0.00825185698721275000000

gloomy a depressed 0.00357038093869261000000

gloomy a sorry 0.04083269211750580000000

gloomy a heavy 0.00757633932439387000000

gloomy a sad 0.16680711552210100000000

blubber v cry 1.00000000000000000000000

bloodless a pale 1.00000000000000000000000

award v grant 0.05555555555555550000000

award v give 0.61111111111111100000000

award v assign 0.05555555555555550000000

award v present 0.11111111111111100000000

award v judge 0.16666666666666700000000

neighbor n friend 0.99558171000311000000000

neighbor n acquaintance 0.00441828999688958000000

auspicious a happy 0.11845102505694800000000

auspicious a right 0.19286256643887600000000

auspicious a lucky 0.11845102505694800000000

auspicious a good 0.21488230827638600000000

auspicious a clear 0.11845102505694800000000

auspicious a promising 0.11845102505694800000000

auspicious a encouraging 0.11845102505694800000000

annihilation n end 1.00000000000000000000000

wander v travel 0.00189254117025732000000

wander v roll 0.36991525393563800000000

wander v walk 0.00262698575120167000000

wander v move 0.55620610902997100000000

wander v drift 0.06935911011293220000000

abnormal a strange 1.00000000000000000000000

though r yet 0.17265403609083300000000

though r nevertheless 0.02094744902359160000000

though r rather 0.65361337664877600000000

though r however 0.07795057782948560000000

though r still 0.07483456040731360000000

mind n fancy 0.00084688149684888900000

mind n intuition 0.00047639174905243200000

mind n brain 0.03734654574910070000000

mind n faith 0.00259232890280956000000

mind n temper 0.00270823069302389000000

mind n hold 0.00280252333622251000000

mind n sentiment 0.00015537793933387400000

mind n conviction 0.02102985561319660000000

mind n recall 0.00011504906575897400000

mind n unanimity 0.01494679112652000000000

mind n consciousness 0.00476688044577039000000

mind n attitude 0.00160956470968378000000

mind n intelligence 0.01082948351997620000000

mind n disposition 0.00142205522947635000000

mind n judgment 0.00374987833602193000000

mind n recollection 0.00295470058597672000000

mind n notion 0.00997203767462442000000

mind n remembrance 0.00009978782821711030000

mind n humor 0.00139847422802429000000

mind n choice 0.00979295425472392000000

mind n thought 0.10984288013563600000000

mind n self 0.00017063917687573800000

mind n belief 0.00339866578914155000000

mind n feeling 0.01860605436658280000000

mind n thinking 0.00290926598159326000000

mind n understanding 0.01177671776705930000000

mind n wit 0.00011504906575897400000

mind n idea 0.09306400050336540000000

mind n genius 0.00081146861088699900000

mind n instinct 0.00013986392195745500000

mind n design 0.00052992753698572000000

mind n talent 0.00077590092728667000000

mind n reason 0.00649181535774436000000

mind n soul 0.13394127877171500000000

mind n faculty 0.02601079530485010000000

mind n wisdom 0.00062440902878412700000

mind n spirit 0.04555568377619720000000

mind n determination 0.03029995736717670000000

mind n temptation 0.00035388127834501300000

mind n nature 0.03953112693400250000000

mind n vein 0.00011564248453787300000

mind n grain 0.00033969979833000100000

mind n attention 0.00127306647352090000000

mind n position 0.00765343614783884000000

mind n opinion 0.03713666679687860000000

mind n accord 0.00042729274698696600000

mind n sense 0.00089755027175336900000

mind n persuasion 0.00006284879885006140000

mind n scholar 0.00088613638735846800000

mind n purpose 0.00477055510604174000000

mind n eye 0.11304484244115700000000

mind n view 0.02132873422428710000000

mind n ear 0.00116929966422332000000

mind n intent 0.00012029610564603700000

mind n control 0.00063603366495830000000

mind n temperament 0.00184417043478886000000

mind n will 0.00707351785572822000000

mind n liking 0.00045057360109442000000

mind n whim 0.00006284879885006140000

mind n partiality 0.00019545403307421900000

mind n cast 0.00062440902878412700000

mind n affection 0.01115206319160600000000

mind n head 0.08381360266433870000000

mind n turn 0.00197892211567128000000

mind n memory 0.04035044929157440000000

mind n desire 0.00476345205957341000000

mind n cleverness 0.00007344860504432450000

mind n impression 0.00104634194922752000000

mind n intention 0.00214347117196794000000

victuals n table 1.00000000000000000000000

unguarded a unwise 0.50000000000000000000000

unguarded a open 0.25000000000000000000000

unguarded a simple 0.25000000000000000000000

undeniable a impregnable 0.00518134715025904000000

undeniable a incontestable 0.01036269430051810000000

undeniable a evident 0.01036269430051810000000

undeniable a sure 0.51295336787564800000000

undeniable a certain 0.06217616580310880000000

undeniable a true 0.36269430051813500000000

undeniable a solid 0.00518134715025904000000

undeniable a positive 0.00518134715025904000000

undeniable a necessary 0.01036269430051810000000

undeniable a real 0.01554404145077720000000

unassuming a comfortable 0.50000000000000000000000

unassuming a simple 0.50000000000000000000000

necessity n fate 0.01019130299817950000000

necessity n distress 0.02689815665052840000000

necessity n call 0.00248825116334666000000

necessity n destiny 0.00248825116334666000000

necessity n poverty 0.00248825116334666000000

necessity n certainty 0.01011187021658190000000

necessity n hunger 0.00829707786516050000000

necessity n impoverishment 0.00248825116334666000000

necessity n demand 0.02689815665052840000000

necessity n restraint 0.00256768394494428000000

necessity n desire 0.69038602069689700000000

necessity n need 0.21469672632379300000000

swindler n scoundrel 0.18970016021973000000000

swindler n thief 0.05420004577706560000000

swindler n criminal 0.29539940489814600000000

swindler n law 0.13550011444266400000000

swindler n rascal 0.05420004577706560000000

swindler n villain 0.02710002288853270000000

swindler n spider 0.02710002288853270000000

swindler n robber 0.02710002288853270000000

swindler n rogue 0.10840009155413100000000

swindler n confidence 0.05420004577706560000000

swindler n impostor 0.02710002288853270000000

broad a complete 0.00251889168765743000000

broad a thick 0.00503778337531486000000

broad a dirty 0.00755667506297229000000

broad a good 0.07808564231738030000000

broad a deep 0.89672544080604500000000

broad a clear 0.00251889168765743000000

broad a great 0.00503778337531486000000

broad a fair 0.00251889168765743000000

swindle v do 0.88888888888888900000000

swindle v diddle 0.11111111111111100000000

subjection n humiliation 1.00000000000000000000000

stupefied a dazzled 0.50000000000000000000000

stupefied a astonished 0.50000000000000000000000

stingy a slight 0.04229454057537140000000

stingy a avaricious 0.01747777211945630000000

stingy a close 0.06344181086305710000000

stingy a narrow 0.02114727028768570000000

stingy a poor 0.06991108847782520000000

stingy a near 0.09472785693374020000000

stingy a selfish 0.03495554423891260000000

stingy a short 0.62108857226503900000000

stingy a mean 0.01747777211945630000000

stingy a shy 0.01747777211945630000000

pause n relief 0.00152267646241926000000

pause n delay 0.01419917805201090000000

pause n stop 0.01805546150814250000000

pause n hesitation 0.00206946739932425000000

pause n holiday 0.00304535292483854000000

pause n rest 0.00476181587450899000000

pause n doubt 0.02244115111121960000000

pause n stillness 0.61411445074947200000000

pause n dismay 0.31636198978003100000000

pause n break 0.00038310321319368100000

pause n halt 0.00152267646241926000000

pause n worry 0.00152267646241926000000

spoilt a bad 1.00000000000000000000000

shirk v abandon 0.01165254237288130000000

shirk v cut 0.01165254237288130000000

shirk v avoid 0.97669491525423700000000

jump v spring 0.05412129023616340000000

jump v take 0.09228845863400210000000

jump v snatch 0.08696412448204040000000

jump v leap 0.21533973681267200000000

jump v rise 0.07233269968540560000000

jump v shake 0.00615256390893347000000

jump v start 0.43452482606842600000000

jump v begin 0.02533408668384370000000

jump v wince 0.00356916690849732000000

jump v surprise 0.00937304658001584000000

score v gain 0.25000000000000000000000

score v make 0.50000000000000000000000

score v get 0.25000000000000000000000

savior n hero 1.00000000000000000000000

right v reconstruct 0.00949503668536900000000

right v improve 0.01899007337073800000000

right v alter 0.01899007337073800000000

right v change 0.03798014674147600000000

right v settle 0.91454466983167900000000

respect v admire 0.04498032817266550000000

respect v spare 0.02521355751099110000000

respect v acknowledge 0.00366599856333473000000

respect v love 0.14414311131249100000000

respect v obey 0.00996943174569093000000

respect v follow 0.02539096253171970000000

respect v honor 0.00785225698982057000000

respect v approve 0.02725513481208850000000

respect v praise 0.01742048935630440000000

respect v consider 0.23557116376718300000000

respect v adore 0.00493673169676112000000

respect v observe 0.01084351282814250000000

respect v value 0.00355908313424185000000

respect v appreciate 0.01078416483181840000000

respect v notice 0.03993750403181890000000

respect v recognize 0.04840126931453920000000

respect v regard 0.00177954156712092000000

respect v attend 0.04698932591787160000000

respect v enjoy 0.29130643191539500000000

rectify v correct 0.60161290322580700000000

rectify v fix 0.39838709677419300000000

real r truly 0.04347826086956520000000

real r exceedingly 0.04347826086956520000000

real r really 0.08695652173913040000000

real r very 0.82608695652173900000000

rankle v get 1.00000000000000000000000

expense n charge 0.00982519990489758000000

expense n capital 0.01622764304763650000000

expense n rate 0.08131452757113000000000

expense n debt 0.09964945434305810000000

expense n value 0.00128048862854778000000

expense n time 0.68319283677974200000000

expense n amount 0.00256097725709557000000

expense n obligation 0.00982519990489758000000

expense n responsibility 0.00128048862854778000000

expense n interest 0.02168570533244520000000

expense n account 0.04582414702091960000000

expense n worth 0.00982519990489758000000

expense n duty 0.01494715441908870000000

expense n figure 0.00128048862854778000000

expense n fare 0.00128048862854778000000

quaker n friend 1.00000000000000000000000

psalm n air 0.40000000000000000000000

psalm n tune 0.60000000000000000000000

circle n course 0.11389481787326400000000

circle n reach 0.00818783022499038000000

circle n district 0.00818783022499038000000

circle n crowd 0.84516603100178400000000

circle n turn 0.00818783022499038000000

circle n school 0.00818783022499038000000

circle n ball 0.00818783022499038000000

postman n courier 1.00000000000000000000000

project n work 0.00886804051256702000000

project n mind 0.07144043825508540000000

project n task 0.00088496038379047200000

project n occupation 0.01277292820604250000000

project n end 0.00480762186025498000000

project n proposal 0.63864641030212400000000

project n point 0.00446615520791453000000

project n hope 0.00176992076758094000000

project n program 0.25545856412085000000000

project n plan 0.00088496038379047200000

charge v prosecute 0.00031420645280192900000

charge v direct 0.00032423690829196400000

charge v demand 0.00098553969345645500000

charge v push 0.00163620243081638000000

charge v call 0.01384841311819910000000

charge v associate 0.00230972459224690000000

charge v invite 0.00258885224471221000000

charge v blame 0.00963677756159166000000

charge v implicate 0.00098553969345645500000

charge v commit 0.08930923902253860000000

charge v fine 0.00587724935170802000000

charge v try 0.02255780296598730000000

charge v refer 0.00426858460289378000000

charge v impute 0.00098553969345645500000

charge v shoot 0.03430573974403210000000

charge v rouse 0.00162617197532635000000

charge v entreat 0.00098553969345645500000

charge v excite 0.00063844336109389300000

charge v bid 0.00099775906972248700000

charge v command 0.00169891907305875000000

charge v send 0.27222957639643200000000

charge v agitate 0.00063844336109389300000

charge v attack 0.00099775906972248700000

charge v summon 0.00199551813944497000000

charge v condemn 0.00098553969345645500000

charge v accuse 0.08466474149319820000000

charge v impose 0.00230972459224690000000

charge v carry 0.00230972459224690000000

charge v expect 0.05041990018658620000000

charge v overwhelm 0.00064847381658393100000

charge v strain 0.00099775906972248700000

charge v load 0.00468544678315977000000

charge v attribute 0.00098553969345645500000

charge v hinder 0.00098553969345645500000

charge v arm 0.00096268026938586000000

charge v dictate 0.00031420645280192900000

charge v fill 0.00131196552252442000000

charge v assign 0.00099775906972248700000

charge v entrust 0.00199551813944497000000

charge v ask 0.36086038195382000000000

charge v point 0.00032423690829196400000

charge v require 0.00195040888361831000000

charge v cross 0.00194037842812828000000

charge v credit 0.00098553969345645500000

charge v ordain 0.00032423690829196400000

charge v encumber 0.00099775906972248700000

charge v consign 0.00099775906972248700000

charge v stow 0.00099775906972248700000

charge v prescribe 0.00099775906972248700000

charge v order 0.00230972459224690000000

charge v plough 0.00099775906972248700000

perfect v conclude 0.03225806451612900000000

perfect v make 0.41935483870967800000000

perfect v improve 0.06451612903225800000000

perfect v complete 0.03225806451612900000000

perfect v accomplish 0.09677419354838710000000

perfect v discharge 0.06451612903225800000000

perfect v execute 0.09677419354838710000000

perfect v effect 0.06451612903225800000000

perfect v finish 0.03225806451612900000000

perfect v end 0.03225806451612900000000

perfect v perform 0.06451612903225800000000

pardonable a permissible 1.00000000000000000000000

ostler n groom 1.00000000000000000000000

special a chief 0.15371684132373200000000

special a new 0.12987339022684700000000

special a remarkable 0.01138332503980330000000

special a rare 0.02327483423316930000000

special a true 0.02659981055219350000000

special a unusual 0.02833458080559740000000

special a strange 0.01095286316855030000000

special a good 0.26797511846838200000000

special a different 0.02659981055219350000000

special a express 0.03522677613668870000000

special a great 0.03522677613668870000000

special a important 0.02327483423316930000000

special a first 0.22756103912298500000000

departure n difference 0.03829561865969760000000

departure n leave 0.17717579526706100000000

departure n parting 0.02821491235907040000000

departure n flight 0.03241118119434290000000

departure n death 0.00419626883527250000000

departure n separation 0.12961225364947900000000

departure n change 0.08585928628472070000000

departure n going 0.37032072471279800000000

departure n expiration 0.10099656128530900000000

departure n decease 0.03291739775224870000000

mischance n harm 1.00000000000000000000000

masterly a admirable 1.00000000000000000000000

carefully r thoughtfully 0.88888888888888900000000

carefully r deliberately 0.11111111111111100000000

legible a sharp 0.01393908105317500000000

legible a certain 0.01393908105317500000000

legible a clear 0.01393908105317500000000

legible a simple 0.95818275684047500000000

board n sheet 0.00781863229860680000000

board n table 0.84200203318003900000000

board n commission 0.00984789564328337000000

board n beam 0.00732996777994387000000

board n committee 0.12900743292701200000000

board n mess 0.00399403817111493000000

weep v whimper 0.00820100754052836000000

weep v cry 0.30685484794151700000000

weep v sob 0.67700251346014400000000

weep v grieve 0.00064528671635058500000

weep v mourn 0.00074203472472950600000

weep v greet 0.00064528671635058500000

weep v lament 0.00590902290038069000000

inattentive a blind 0.66666666666666800000000

inattentive a absent 0.16666666666666600000000

inattentive a sleepy 0.16666666666666600000000

star n fate 0.00025037639702160100000

star n name 0.04496401509806660000000

star n luck 0.00008345879900719980000

star n genius 0.00016691759801440000000

star n hero 0.00008345879900719980000

star n destiny 0.00068085920353155900000

star n state 0.00016691759801440000000

star n favorite 0.04167804024267260000000

star n lead 0.00008345879900719980000

star n sun 0.89942340747428000000000

star n doom 0.00016691759801440000000

star n fortune 0.00008345879900719980000

star n heroine 0.00815999439384036000000

star n condition 0.00008345879900719980000

star n situation 0.00392526040150861000000

immaculate a white 1.00000000000000000000000

illegitimate a wrong 0.14062500000000000000000

illegitimate a natural 0.85937500000000000000000

hubbub n uproar 1.00000000000000000000000

proper a private 0.00147005520298012000000

proper a regular 0.00147005520298012000000

proper a useful 0.00294011040596024000000

proper a capable 0.00051884301281651200000

proper a respectable 0.03736919314178740000000

proper a moral 0.00246969274100660000000

proper a reasonable 0.00294011040596024000000

proper a happy 0.07148277458750530000000

proper a polite 0.00147005520298012000000

proper a true 0.09794192871064380000000

proper a exact 0.00147005520298012000000

proper a right 0.02733126227658570000000

proper a fit 0.00013249424576215600000

proper a correct 0.00173504369450443000000

proper a able 0.00013249424576215600000

proper a usual 0.07938298096092640000000

proper a good 0.63832823165120700000000

proper a own 0.00690453122798132000000

proper a natural 0.01372292283267480000000

proper a advantageous 0.00294011040596024000000

proper a fair 0.00784705464103478000000

gauze n veil 1.00000000000000000000000

many a various 0.02421930435013460000000

many a bountiful 0.00253979869242900000000

many a numerous 0.18834194678543800000000

many a frequent 0.00277745152871197000000

many a large 0.06818383983222780000000

many a usual 0.02024413956545070000000

many a innumerable 0.00302557344233635000000

many a several 0.46672140281663000000000

many a abundant 0.00145845813560774000000

many a crowded 0.00056591007484826300000

many a great 0.20671691341346400000000

many a common 0.00641068574910502000000

many a countless 0.00873469898135365000000

many a varied 0.00005987663226369020000

mass n people 0.88175131456832100000000

mass n section 0.00800263520595465000000

mass n world 0.02177582358996370000000

mass n storm 0.00288527658902722000000

mass n everything 0.02400790561786390000000

mass n mountain 0.00288527658902722000000

mass n body 0.00577055317805443000000

mass n company 0.03114539107182350000000

mass n flood 0.00800263520595465000000

mass n greatness 0.00288527658902722000000

mass n bouquet 0.00288527658902722000000

mass n whole 0.00800263520595465000000

feint n cloak 1.00000000000000000000000

sum n charge 0.00039125083497862100000

sum n lot 0.00039125083497862100000

sum n outcome 0.00039125083497862100000

sum n heart 0.00156500333991448000000

sum n price 0.22562457526129600000000

sum n everything 0.01117315437589390000000

sum n heap 0.00039125083497862100000

sum n count 0.06993608675242850000000

sum n amount 0.20511325023754200000000

sum n point 0.00391250834978621000000

sum n soul 0.04558072227500940000000

sum n meat 0.00039125083497862100000

sum n substance 0.00726064602610768000000

sum n sketch 0.00039125083497862100000

sum n answer 0.42670404670219300000000

sum n glance 0.00039125083497862100000

sum n whole 0.00039125083497862100000

eventful a busy 1.00000000000000000000000

entrap v deceive 0.38888888888888900000000

entrap v reveal 0.33333333333333300000000

entrap v capture 0.16666666666666700000000

entrap v catch 0.05555555555555550000000

entrap v fool 0.05555555555555550000000

enrapture v send 0.55555555555555600000000

enrapture v enamour 0.11111111111111100000000

enrapture v delight 0.11111111111111100000000

enrapture v charm 0.11111111111111100000000

enrapture v bewitch 0.11111111111111100000000

enquire v inquire 0.20000000000000000000000

enquire v ask 0.80000000000000000000000

grave n home 0.01930245021863470000000

grave n coffin 0.00116430596147862000000

grave n end 0.12735229085322100000000

grave n tomb 0.83299129721953700000000

grave n death 0.01102539403513260000000

grave n rest 0.00816426171199599000000

conduct n charge 0.01763168274076920000000

conduct n dealings 0.00662270888119111000000

conduct n manners 0.03409614216280890000000

conduct n rule 0.00662270888119111000000

conduct n attitude 0.00662270888119111000000

conduct n government 0.00662270888119111000000

conduct n behavior 0.07820284705981040000000

conduct n air 0.00662270888119111000000

conduct n goings-on 0.00662270888119111000000

conduct n manner 0.02747343328161770000000

conduct n performance 0.48729194820553600000000

conduct n operation 0.00662270888119111000000

conduct n execution 0.01763168274076920000000

conduct n carriage 0.03848240714119580000000

conduct n transaction 0.00662270888119111000000

conduct n accomplishment 0.00662270888119111000000

conduct n direction 0.08652847678416060000000

conduct n effect 0.00662270888119111000000

conduct n care 0.02747343328161770000000

conduct n cause 0.03973625328714670000000

conduct n boss 0.01763168274076920000000

conduct n plan 0.06159292176188710000000

disgusting a awful 0.31603053435114500000000

disgusting a sick 0.05190839694656490000000

disgusting a disagreeable 0.31603053435114500000000

disgusting a abominable 0.31603053435114500000000

apartment n home 0.10545362605886900000000

apartment n residence 0.05113801481297120000000

apartment n cabin 0.00158168857531705000000

apartment n story 0.00101235345489511000000

apartment n flat 0.00004754813092791340000

apartment n room 0.82615590759188500000000

apartment n chamber 0.01461086137513460000000

share v part 0.07056997573092850000000

share v yield 0.25516108683571700000000

share v divide 0.00168600207550256000000

share v experience 0.03207739377363300000000

share v receive 0.06363821013429310000000

share v deal 0.43304481594404600000000

share v give 0.07879781568364320000000

share v dispense 0.03207739377363300000000

share v split 0.03207739377363300000000

share v accord 0.00086991227497067200000

corroborate v confirm 0.50000000000000000000000

corroborate v prove 0.50000000000000000000000

confusing a unsettling 1.00000000000000000000000

bury v ensconce 0.00050912102495137500000

bury v lodge 0.05210586281271190000000

bury v embed 0.00050912102495137500000

bury v forget 0.59719619287536100000000

bury v plant 0.00050912102495137500000

bury v hide 0.07959250737444680000000

bury v engage 0.23349975820273200000000

bury v sink 0.01676635526756860000000

bury v conceal 0.00450152684377888000000

bury v beat 0.01430131252359520000000

bury v cancel 0.00050912102495137500000

police n law 1.00000000000000000000000

caste n place 0.61904761904762000000000

caste n family 0.14285714285714300000000

caste n order 0.14285714285714300000000

caste n position 0.04761904761904740000000

caste n blood 0.04761904761904740000000

castaway n second 1.00000000000000000000000

canvass v ventilate 0.06716417910447760000000

canvass v argue 0.64925373134328400000000

canvass v consider 0.28358208955223900000000

elder a chief 0.74868344060854300000000

elder a former 0.00175541252194266000000

elder a senior 0.24956114686951400000000

buckle v begin 0.88888888888888900000000

buckle v bind 0.11111111111111100000000

much a enough 0.03319777983886390000000

much a preposterous 0.00363603728075196000000

much a complete 0.00060876350780297600000

much a many 0.38065328233729200000000

much a sufficient 0.00388153495968280000000

much a generous 0.03049624791280370000000

much a bountiful 0.02563888942746160000000

much a full 0.00039882289080360700000

much a principal 0.00011513251094035800000

much a large 0.04297365534645800000000

much a liberal 0.00856144948375639000000

much a significant 0.00016457592054664600000

much a urgent 0.00016457592054664600000

much a first-rate 0.00054405131922286100000

much a extravagant 0.01281944471373080000000

much a abundant 0.01321826760453440000000

much a great 0.19629019252861900000000

much a serious 0.11203973659469700000000

much a plenty 0.08047762514731000000000

much a lavish 0.00027203704275147000000

much a important 0.05306908198419220000000

much a fraught 0.00077881572723203300000

win v have 0.38797749928782900000000

win v gain 0.00480619639882304000000

win v sell 0.00058190062151567900000

win v bring 0.00771776972625031000000

win v convince 0.00853325436994875000000

win v realize 0.00032800831593164600000

win v make 0.02894524128924990000000

win v take 0.32651396652315900000000

win v collect 0.00041789646354985600000

win v secure 0.00097429575501909300000

win v receive 0.00487791853193726000000

win v draw 0.04398044836116830000000

win v accomplish 0.00125368939064957000000

win v acquire 0.00041789646354985600000

win v attain 0.00083579292709971200000

win v carry 0.00350382808378353000000

win v procure 0.00041789646354985600000

win v get 0.02528909920215610000000

win v reach 0.12125616005276800000000

win v lead 0.00187594590521728000000

win v capture 0.00141769354861539000000

win v catch 0.00363329936508312000000

win v charm 0.00041789646354985600000

win v effect 0.00139219221856895000000

win v touch 0.00271704894179847000000

win v upset 0.00016400415796582300000

win v approach 0.00016400415796582300000

win v prompt 0.00158268002211491000000

win v slay 0.00041789646354985600000

win v vanquish 0.00041789646354985600000

win v advance 0.00074590477948150200000

win v succeed 0.00237793880594298000000

win v beat 0.00465832175159678000000

win v recover 0.00938851872706107000000

blink v forget 1.00000000000000000000000

slip v pass 0.53712997283028900000000

slip v fall 0.02210411410824230000000

slip v mistake 0.00587991212731569000000

slip v decline 0.01613002921412280000000

slip v move 0.35882410266940500000000

slip v sink 0.02984055404612720000000

slip v put 0.03009131500449800000000

barrister n solicitor 1.00000000000000000000000

visible a plain 0.02594158350432740000000

visible a bold 0.01447902335125250000000

visible a apparent 0.03553784901749140000000

visible a open 0.27399574556187000000000

visible a perceptible 0.02953328719370500000000

visible a clear 0.60873186134626200000000

visible a noticeable 0.00589032501254585000000

visible a unmistakable 0.00589032501254585000000

ail v hurt 0.50000000000000000000000

ail v suffer 0.50000000000000000000000

11 n xi 1.00000000000000000000000

winder n key 1.00000000000000000000000

wield v hold 1.00000000000000000000000

fashion n vogue 0.00191104230849473000000

fashion n cry 0.00116708161262439000000

fashion n quality 0.00031109323658331300000

fashion n style 0.00015554661829165600000

fashion n sort 0.06105592331668840000000

fashion n custom 0.00015554661829165600000

fashion n manner 0.09483586950427640000000

fashion n look 0.01527649451908210000000

fashion n order 0.00782001681284824000000

fashion n kind 0.07541921155435680000000

fashion n type 0.00031109323658331300000

fashion n character 0.02043774595240770000000

fashion n line 0.00015554661829165600000

fashion n figure 0.00575438408426001000000

fashion n appearance 0.04099239285625260000000

fashion n way 0.64149525772696600000000

fashion n thing 0.02093446447281970000000

fashion n decorum 0.00373929578052038000000

fashion n clothes 0.00729426007890243000000

fashion n taste 0.00077773309145828500000

unhinge v unsettle 0.24041159962581900000000

unhinge v overcome 0.09292173370751480000000

unhinge v trouble 0.24041159962581900000000

unhinge v confuse 0.24041159962581900000000

unhinge v change 0.13938260056127200000000

unhinge v remove 0.04646086685375740000000

unanswerable a exempt 1.00000000000000000000000

gently r softly 0.99486158719093400000000

gently r tenderly 0.00175509855557934000000

gently r mildly 0.00162821569790753000000

gently r blandly 0.00175509855557934000000

transpire v come 0.69473684210526300000000

transpire v appear 0.30526315789473700000000

thereafter r then 1.00000000000000000000000

distant a slight 0.00728502058686155000000

distant a faint 0.00364251029343077000000

distant a indifferent 0.03221771136542520000000

distant a cool 0.00364251029343077000000

distant a distinct 0.49734457103876000000000

distant a modest 0.01092753088029230000000

distant a uncertain 0.00364251029343077000000

distant a formal 0.00364251029343077000000

distant a different 0.10952999234349300000000

distant a cold 0.06638475009777590000000

distant a reserved 0.25445536192680700000000

distant a proud 0.00728502058686155000000

assume v suspect 0.00516175798694420000000

assume v believe 0.03083075030951880000000

assume v understand 0.09807340175193990000000

assume v copy 0.00386669325903706000000

assume v affect 0.09807340175193990000000

assume v take 0.21576148385426800000000

assume v think 0.08457746003131140000000

assume v suppose 0.04514186878050410000000

assume v adopt 0.00337127318522293000000

assume v hope 0.07866304098853510000000

assume v expect 0.02796102562941170000000

assume v strike 0.01160007977711120000000

assume v imply 0.03596024730904460000000

assume v find 0.08855446062361260000000

assume v imitate 0.00337127318522293000000

assume v feel 0.05052104429487890000000

assume v imagine 0.10481594812238600000000

assume v begin 0.00853303117216714000000

assume v fancy 0.00516175798694420000000

bucket n basin 1.00000000000000000000000

soon r surely 0.01761121435711880000000

soon r absolutely 0.00423130165362880000000

soon r finally 0.02611734870389190000000

soon r early 0.15075899077913800000000

soon r rapidly 0.09247661991451430000000

soon r freely 0.00303202032506604000000

soon r overnight 0.00030024876595621500000

soon r shortly 0.01687282251236870000000

soon r happily 0.00511803496749464000000

soon r willingly 0.00063997660840494400000

soon r speedily 0.10788938990026700000000

soon r immediately 0.06339445902046060000000

soon r quickly 0.17840995221920400000000

soon r directly 0.12014076032687700000000

soon r presently 0.07735054520763320000000

soon r instantly 0.13565631473797600000000

sedate a dull 0.10526315789473700000000

sedate a grave 0.05263157894736840000000

sedate a cool 0.05263157894736840000000

sedate a earnest 0.05263157894736840000000

sedate a dreary 0.05263157894736840000000

sedate a formal 0.05263157894736840000000

sedate a quiet 0.31578947368421100000000

sedate a serious 0.31578947368421100000000

neighborhood n closeness 0.11765546049838800000000

neighborhood n part 0.86964956148639800000000

neighborhood n parish 0.00257080736560865000000

neighborhood n ward 0.00257080736560865000000

neighborhood n quarter 0.00755336328399658000000

manners n conduct 0.02033729782829860000000

manners n conventionality 0.00165863880811434000000

manners n vanity 0.06903047389520900000000

manners n behavior 0.08758674384510150000000

manners n politeness 0.00131674723691386000000

manners n practice 0.00086126604984664700000

manners n carriage 0.01638705611755700000000

manners n intercourse 0.00429213328194205000000

manners n formality 0.00131674723691386000000

manners n taste 0.79721289570010300000000

repugnant a incompatible 1.00000000000000000000000

fully r quite 0.35879163536646500000000

fully r completely 0.01221942259255810000000

fully r thoroughly 0.43988069905543600000000

fully r positively 0.00052581238471250900000

fully r well 0.00450189253410035000000

fully r perfectly 0.01292168825879710000000

fully r entirely 0.14157498458384300000000

fully r altogether 0.02958386522408800000000

square n green 1.00000000000000000000000

pretense n side 0.50000000000000000000000

pretense n falsehood 0.50000000000000000000000

presumptuous a sure 0.91666666666666700000000

presumptuous a satisfied 0.05555555555555550000000

presumptuous a convinced 0.00925925925925921000000

presumptuous a positive 0.00925925925925921000000

presumptuous a insolent 0.00925925925925921000000

prayer n praise 0.00934484946668928000000

prayer n entreaty 0.91144880612313700000000

prayer n petition 0.07920634441017390000000

overseer n master 0.45658073270013600000000

overseer n keeper 0.31512890094979600000000

overseer n head 0.22829036635006800000000

bar n bank 0.00014233150967876700000

bar n tongue 0.02135965655714160000000

bar n inn 0.00028466301935753500000

bar n guard 0.00193442550959792000000

bar n hotel 0.00127564615549596000000

bar n barricade 0.00014233150967876700000

bar n table 0.01120079551167180000000

bar n stop 0.00121570513826220000000

bar n pale 0.00014233150967876700000

bar n commission 0.00014233150967876700000

bar n strut 0.00014233150967876700000

bar n pig 0.00056932603871507200000

bar n line 0.00961123681280537000000

bar n restraint 0.00014233150967876700000

bar n counter 0.03280232971275320000000

bar n flat 0.00014233150967876700000

bar n lever 0.45923261597854500000000

bar n crow 0.00014233150967876700000

bar n drawback 0.00014233150967876700000

bar n restaurant 0.45923261597854500000000

naught n none 0.02692211815818520000000

naught n nothing 0.97307788184181500000000

mortality n man 1.00000000000000000000000

murder n parricide 0.00010967568657304500000

murder n discovery 0.03984491375723100000000

murder n death 0.26190909634297000000000

murder n destruction 0.00005658843924453050000

murder n execution 0.00095547560907420200000

murder n ride 0.00183040199721783000000

murder n hanging 0.00262400904626653000000

murder n revelation 0.00010967568657304500000

murder n suicide 0.23296989126751300000000

murder n crime 0.45959027216733700000000

meritorious a honest 0.10000000000000000000000

meritorious a worthy 0.30000000000000000000000

meritorious a laudable 0.10000000000000000000000

meritorious a generous 0.10000000000000000000000

meritorious a honorable 0.10000000000000000000000

meritorious a right 0.10000000000000000000000

meritorious a good 0.10000000000000000000000

meritorious a deserving 0.10000000000000000000000

mediocrity n dick 0.04238154755141450000000

mediocrity n indifference 0.36427678672878100000000

mediocrity n tom 0.59334166571980500000000

cry v whimper 0.00152309807090056000000

cry v pray 0.01485853512176490000000

cry v squeal 0.00151327168300790000000

cry v snarl 0.01273803437005740000000

cry v call 0.09810198185127870000000

cry v pipe 0.00152374411750458000000

cry v blubber 0.00527091077381685000000

cry v weep 0.00836691848967229000000

cry v announce 0.00589188848664130000000

cry v holler 0.00011508538807460300000

cry v hey 0.00000554082826501243000

cry v adjure 0.00011508538807460300000

cry v whoop 0.00055903599116239300000

cry v regret 0.00180562017021697000000

cry v purr 0.00004880472938719300000

cry v entreat 0.00283991064397968000000

cry v snivel 0.00268236217888995000000

cry v sob 0.01414683321072490000000

cry v complain 0.00912338800588328000000

cry v grieve 0.00130330470635401000000

cry v scream 0.06150216227804250000000

cry v cheer 0.00003984708155810200000

cry v mourn 0.00035524811481792700000

cry v greet 0.01247474018619600000000

cry v groan 0.02808943356530060000000

cry v whistle 0.00076490085465236700000

cry v moan 0.01344882514769210000000

cry v implore 0.00093500484177944700000

cry v plead 0.02112419948350800000000

cry v roar 0.02020934787947270000000

cry v appeal 0.01309714340506800000000

cry v beseech 0.00038817967816728800000

cry v shriek 0.00220328752169553000000

cry v growl 0.00933390449530068000000

cry v hail 0.01438958641252000000000

cry v fret 0.00342407990403530000000

cry v beg 0.03493965666799720000000

cry v yell 0.02938532882145060000000

cry v exclaim 0.35087895888983500000000

cry v address 0.00612435628592668000000

cry v ejaculate 0.01208318821396510000000

cry v shout 0.17452959368784600000000

cry v howl 0.00085200447675652000000

cry v hiss 0.00614939590278633000000

cry v utter 0.00050926323735722400000

cry v bawl 0.00023500876061603800000

busy a diligent 0.00465008917730010000000

busy a eventful 0.01868953650164180000000

busy a active 0.00465008917730010000000

busy a lively 0.00065061333553641000000

busy a full 0.36397723964472300000000

busy a confused 0.07243126932203080000000

busy a animated 0.00153728018548716000000

busy a intent 0.00465008917730010000000

busy a energetic 0.00465008917730010000000

busy a forward 0.00465008917730010000000

busy a inquisitive 0.00105713324393293000000

busy a industrious 0.00153728018548716000000

busy a curious 0.51686920169466000000000

personal a physical 0.94393558127830900000000

personal a own 0.05244086562657270000000

personal a particular 0.00362355309511827000000

announce v deliver 0.00009558502707282360000

announce v reveal 0.00057351016243694200000

announce v communicate 0.00494918029065954000000

announce v report 0.00019117005414564700000

announce v issue 0.00009558502707282360000

announce v tell 0.03983166995870410000000

announce v say 0.88114408504385900000000

announce v publish 0.00009558502707282360000

announce v notify 0.00009558502707282360000

announce v speak 0.07283245935483080000000

announce v predict 0.00009558502707282360000

supper n meal 0.04646069380605790000000

supper n dinner 0.60469741913799800000000

supper n repast 0.00013071392208281700000

supper n tea 0.34871117313386200000000

indubitable a sure 1.00000000000000000000000

inconsolable a miserable 0.50000000000000100000000

inconsolable a hopeless 0.16666666666666600000000

inconsolable a distressed 0.33333333333333300000000

inactive a dull 0.45454545454545500000000

inactive a idle 0.18181818181818200000000

inactive a sleepy 0.09090909090909060000000

inactive a silent 0.09090909090909060000000

inactive a quiet 0.18181818181818200000000

impregnable a safe 0.06153846153846150000000

impregnable a faultless 0.00769230769230765000000

impregnable a undeniable 0.00769230769230765000000

impregnable a incontestable 0.01538461538461530000000

impregnable a sure 0.76153846153846200000000

impregnable a certain 0.09230769230769230000000

impregnable a solid 0.00769230769230765000000

impregnable a strong 0.02307692307692300000000

impregnable a immovable 0.00769230769230765000000

impregnable a perfect 0.01538461538461530000000

ignominious a dishonorable 0.05263157894736830000000

ignominious a outrageous 0.10526315789473700000000

ignominious a wicked 0.10526315789473700000000

ignominious a execrable 0.05263157894736830000000

ignominious a black 0.15789473684210500000000

ignominious a mean 0.05263157894736830000000

ignominious a shameful 0.10526315789473700000000

ignominious a infamous 0.15789473684210500000000

ignominious a abominable 0.15789473684210500000000

ignominious a disgraceful 0.05263157894736830000000

idolize v love 0.50000000000000000000000

idolize v adore 0.50000000000000000000000

mystery n wonder 0.23225517843140100000000

mystery n paradox 0.00127438658432167000000

mystery n mass 0.00285542244049575000000

mystery n dark 0.00127438658432167000000

mystery n mist 0.00030874067535019200000

mystery n question 0.45976365419492400000000

mystery n sacrifice 0.00158312725967186000000

mystery n fog 0.00285542244049575000000

mystery n puzzler 0.00127438658432167000000

mystery n miracle 0.00127438658432167000000

mystery n poser 0.00127438658432167000000

mystery n knot 0.00127438658432167000000

mystery n unknown 0.00127438658432167000000

mystery n obscurity 0.00127438658432167000000

mystery n revelation 0.00030874067535019200000

mystery n uncertainty 0.14685029693978100000000

mystery n prophet 0.00254877316864334000000

mystery n riddle 0.00382315975296501000000

mystery n secret 0.01621530791546370000000

mystery n enigma 0.05410274097781420000000

mystery n morality 0.05741718948526510000000

mystery n puzzle 0.00127438658432167000000

mystery n prodigy 0.00382138319174152000000

mystery n fix 0.00382138319174152000000

haul n prize 0.50000000000000000000000

haul n take 0.50000000000000000000000

farther r further 0.85550458715596300000000

farther r longer 0.14449541284403700000000

naturally r clearly 0.00118427257989315000000

naturally r usually 0.02145072254879920000000

naturally r obviously 0.00059213628994657500000

naturally r instinctively 0.05583496898731550000000

naturally r simply 0.01565983829895060000000

naturally r directly 0.83475292884557200000000

naturally r ordinarily 0.02709229445242090000000

naturally r easily 0.04343283799710270000000

formulate v say 0.70454545454545400000000

formulate v express 0.02272727272727270000000

formulate v imagine 0.18181818181818200000000

formulate v determine 0.06818181818181820000000

formulate v put 0.02272727272727270000000

lawyer n justice 0.03011287399216030000000

lawyer n judge 0.12678885736571100000000

lawyer n agent 0.00545287982145275000000

lawyer n sergeant 0.38032635392348400000000

lawyer n counsel 0.03288545185778950000000

lawyer n prosecutor 0.00823162043539490000000

lawyer n law 0.02726439910726390000000

lawyer n solicitor 0.30370106284143100000000

lawyer n notary 0.07558864783817160000000

lawyer n deputy 0.00964785281714121000000

fickle a unaccountable 0.50000000000000000000000

fickle a giddy 0.50000000000000000000000

nice a charming 0.01569436675283620000000

nice a gentle 0.00004156935447905570000

nice a chaste 0.00010230627172133800000

nice a excellent 0.00214746553383971000000

nice a wonderful 0.00251010735313690000000

nice a kind 0.04381853520335850000000

nice a close 0.00154597194387753000000

nice a gratifying 0.00146283323491942000000

nice a comfortable 0.03829989595431550000000

nice a faultless 0.00004156935447905570000

nice a unassuming 0.00043759713613923000000

nice a proper 0.00082861871030173700000

nice a delightful 0.01775977440837060000000

nice a cheerful 0.00315433968302133000000

nice a helpful 0.00048761107830647200000

nice a dry 0.00499996450700800000000

nice a friendly 0.00039938250498545100000

nice a delicate 0.00048761107830647200000

nice a piquant 0.00052918043278552700000

nice a superior 0.03000672728877200000000

nice a lovely 0.00458880026229943000000

nice a generous 0.00004156935447905570000

nice a respectable 0.01008202852967580000000

nice a welcome 0.00089201642596392700000

nice a amiable 0.00066288252780675500000

nice a careful 0.00359097071542812000000

nice a sure 0.05163767942683420000000

nice a wise 0.00234070721628987000000

nice a faithful 0.00097902779237106800000

nice a civil 0.00065923426733612500000

nice a polite 0.00218487943166917000000

nice a modest 0.00166433336220832000000

nice a true 0.02646185583573100000000

nice a exact 0.00179353203911577000000

nice a smooth 0.00218087028010852000000

nice a right 0.01408724940027240000000

nice a mild 0.00048761107830647200000

nice a elegant 0.00223539203711123000000

nice a exquisite 0.00101087936516836000000

nice a neat 0.00041284872207648300000

nice a graceful 0.00020414539151700800000

nice a keen 0.00360831160255625000000

nice a positive 0.00051699894251894700000

nice a admirable 0.00016627741791622400000

nice a decent 0.04474812412478260000000

nice a correct 0.00200256995608016000000

nice a satisfactory 0.00144571636405579000000

nice a amusing 0.00056946381534347100000

nice a definite 0.00017162318902965300000

nice a suitable 0.00004028338255794360000

nice a fine 0.12307760559281700000000

nice a attractive 0.00166433336220832000000

nice a good 0.27644620505663600000000

nice a cordial 0.00004028338255794360000

nice a virtuous 0.00038026511767772700000

nice a precise 0.00019249877606985900000

nice a genial 0.00070406048412952200000

nice a beautiful 0.01190308262942440000000

nice a fascinating 0.00072939458815331500000

nice a considerate 0.00072939458815331500000

nice a obliging 0.00117672228390184000000

nice a favorable 0.00072939458815331500000

nice a rich 0.01206418568174990000000

nice a good-looking 0.00455135083933836000000

nice a particular 0.00206365815119193000000

nice a great 0.01844497489573030000000

nice a sweet 0.06331623335992550000000

nice a handsome 0.01730126754366330000000

nice a affable 0.00097522215661294400000

nice a pleasant 0.01818324594705520000000

nice a fair 0.00077076790566962600000

nice a entertaining 0.00048761107830647200000

nice a perfect 0.02266984123869830000000

nice a well-bred 0.00558848009277809000000

nice a pretty 0.04638990973887750000000

nice a congenial 0.00070406048412952200000

nice a fastidious 0.00055396317806764200000

nice a difficult 0.00216087877004292000000

nice a agreeable 0.01863426916455830000000

nice a warm 0.00114352928215091000000

voice n airing 0.00007844841080074970000

voice n tongue 0.02882810993190120000000

voice n music 0.06213126099089450000000

voice n language 0.02284089702834200000000

voice n cry 0.01477215217628990000000

voice n whistle 0.02038351207306150000000

voice n song 0.03503894917413250000000

voice n ring 0.00081644312619743600000

voice n part 0.01277761862808480000000

voice n choice 0.00011517969721820200000

voice n sheet 0.00045284151146669400000

voice n feeling 0.00612495621080800000000

voice n rag 0.00062081762659070500000

voice n agent 0.00104315674553954000000

voice n newspaper 0.00022860013539882000000

voice n call 0.00167391126247552000000

voice n decision 0.00101695729216278000000

voice n vehicle 0.00105117543636293000000

voice n journal 0.00005832192295582680000

voice n speaker 0.00377955823255363000000

voice n expression 0.09514909367158170000000

voice n tone 0.54109924593154800000000

voice n sound 0.09912354417013940000000

voice n opinion 0.00384381441508832000000

voice n utterance 0.00008336814753808370000

voice n beat 0.00274833870648020000000

voice n view 0.00263546380421925000000

voice n paper 0.00086862232375090000000

voice n groan 0.00005685777426237480000

voice n representative 0.00093137561570255500000

voice n wish 0.00358032111809606000000

voice n vote 0.00005685777426237480000

voice n interpreter 0.00116477211846066000000

voice n noise 0.03345673081448280000000

voice n speech 0.00136872600115039000000

judge n justice 0.00433218046202901000000

judge n connoisseur 0.01850136368065730000000

judge n guardian 0.09845846316299160000000

judge n authority 0.15883841205382500000000

judge n referee 0.00216609023101450000000

judge n master 0.62453413196310900000000

judge n protector 0.08420349554660620000000

judge n chancellor 0.00896586289976692000000

ending n termination 1.00000000000000000000000

disturb v perturb 0.00028382075111138500000

disturb v incommode 0.00297965613924367000000

disturb v dishearten 0.00068760148179973100000

disturb v molest 0.00597226730517970000000

disturb v rouse 0.00159355004358537000000

disturb v inconvenience 0.00207208726917798000000

disturb v excite 0.00268523568293149000000

disturb v agitate 0.00097142223291111600000

disturb v worry 0.02761899432223900000000

disturb v rattle 0.00050216783109729000000

disturb v replace 0.00068760148179973100000

disturb v trouble 0.00028382075111138500000

disturb v alarm 0.00763837279470135000000

disturb v grieve 0.00983320413769120000000

disturb v move 0.08356557120361800000000

disturb v startle 0.03578870509687230000000

disturb v confuse 0.00068760148179973100000

disturb v vex 0.00444264649805129000000

disturb v shake 0.31378645740197200000000

disturb v arouse 0.02861711417065270000000

disturb v provoke 0.00194284446582224000000

disturb v confound 0.00171381345002037000000

disturb v annoy 0.00385137439032383000000

disturb v bother 0.01578268617636590000000

disturb v touch 0.05789704125641120000000

disturb v upset 0.00171381345002037000000

disturb v bewilder 0.00021834707998590500000

disturb v harass 0.00693748222318854000000

disturb v disconcert 0.00240141493182010000000

disturb v depress 0.00410754433380457000000

disturb v perplex 0.00097142223291111600000

disturb v plague 0.00383996814138312000000

disturb v irritate 0.06992122488021060000000

disturb v remove 0.04913602756820430000000

disturb v concern 0.04466881681143430000000

disturb v distort 0.04578738267304440000000

disturb v displease 0.00297965613924367000000

disturb v interrupt 0.15474364023645900000000

disturb v discompose 0.00068760148179973100000

sound v reflect 0.05770403434438370000000

sound v ring 0.20510773615189600000000

sound v murmur 0.00008624443027861620000

sound v cry 0.00356168310964723000000

sound v announce 0.00027386822185279400000

sound v tinkle 0.04102154723037920000000

sound v burst 0.00036837823042033900000

sound v appear 0.00173771933968431000000

sound v use 0.28902250337257300000000

sound v rattle 0.00027386822185279400000

sound v plunge 0.00027386822185279400000

sound v play 0.01249721945488260000000

sound v express 0.01675046511907150000000

sound v smash 0.00027386822185279400000

sound v seem 0.24322237574842500000000

sound v articulate 0.00008624443027861620000

sound v shout 0.01879972826137890000000

sound v go 0.10757617613275400000000

sound v sing 0.00036011265213141000000

sound v test 0.00027386822185279400000

sound v declare 0.00045462266069895500000

sound v utter 0.00027386822185279400000

diffident a self-conscious 0.12500000000000000000000

diffident a fearful 0.12500000000000000000000

diffident a doubtful 0.12500000000000000000000

diffident a suspicious 0.12500000000000000000000

diffident a shy 0.12500000000000000000000

diffident a embarrassed 0.12500000000000000000000

diffident a quiet 0.25000000000000100000000

decipher v trace 0.02500000000000000000000

decipher v read 0.97500000000000000000000

dawdle v wait 0.73184004166365400000000

dawdle v waste 0.02235850133916190000000

dawdle v delay 0.06707550401748600000000

dawdle v play 0.15636745164053600000000

dawdle v loiter 0.02235850133916190000000

stupid a mad 0.03003982036088410000000

stupid a fruitless 0.00061958292122117900000

stupid a imbecile 0.00083890430926407400000

stupid a vain 0.00912837805127597000000

stupid a empty 0.00423699960487863000000

stupid a outrageous 0.00061699052406125600000

stupid a dull 0.03083575987113320000000

stupid a thick 0.00224306470522101000000

stupid a nonsensical 0.00112153235261050000000

stupid a injudicious 0.00108798068786617000000

stupid a blind 0.01214702923551580000000

stupid a slow 0.22970777564559200000000

stupid a ordinary 0.04216517665449510000000

stupid a foolish 0.06049367860795280000000

stupid a useless 0.00901972216269865000000

stupid a tactless 0.00112153235261050000000

stupid a unconscious 0.00383568386490105000000

stupid a flat 0.00143002761464113000000

stupid a absurd 0.02343441797346780000000

stupid a ridiculous 0.03980700230017070000000

stupid a ignorant 0.05510658905207950000000

stupid a boring 0.04739809347342020000000

stupid a silly 0.14072907122887800000000

stupid a childish 0.00263002603396419000000

stupid a careless 0.02764888785949510000000

stupid a monstrous 0.00221905651907381000000

stupid a confused 0.00336459705783152000000

stupid a dumb 0.00265619775438149000000

stupid a crazy 0.00362955255941451000000

stupid a unreasonable 0.00754716195029993000000

stupid a insensible 0.00171943591947749000000

stupid a reckless 0.00112153235261050000000

stupid a giddy 0.00030849526203062700000

stupid a obstinate 0.00360006533636187000000

stupid a tedious 0.00112153235261050000000

stupid a funny 0.03556977844328560000000

stupid a monotonous 0.00166549589317149000000

stupid a tiresome 0.01140681014374870000000

stupid a shallow 0.00224306470522101000000

stupid a heavy 0.02590425235639960000000

stupid a commonplace 0.00030849526203062700000

stupid a thoughtless 0.00135700063114086000000

stupid a improper 0.02954309650761960000000

stupid a extravagant 0.00083890430926407400000

stupid a lifeless 0.02457678920844010000000

stupid a insane 0.00081520062484997800000

stupid a imprudent 0.00661675493525511000000

stupid a wild 0.00145589483332533000000

stupid a comic 0.00112153235261050000000

stupid a deficient 0.00247853298375137000000

stupid a idiotic 0.00900753684856475000000

stupid a simple 0.03554639350210040000000

stupid a inconsistent 0.00112153235261050000000

stupid a light 0.00083890430926407400000

stupid a absent-minded 0.00030849526203062700000

stupid a green 0.00142048413604400000000

stupid a stunned 0.00112369588688061000000

crave v pray 0.33333333333333300000000

crave v need 0.33333333333333300000000

crave v want 0.33333333333333300000000

cramp n stoppage 0.20000000000000000000000

cramp n barrier 0.40000000000000000000000

cramp n pain 0.20000000000000000000000

cramp n hindrance 0.20000000000000000000000

hold n clutch 0.16937090805579300000000

hold n judge 0.10861245532466700000000

hold n charm 0.16937090805579300000000

hold n point 0.16771040895720700000000

hold n have 0.16771040895720700000000

hold n think 0.10861245532466700000000

hold n effect 0.10861245532466700000000

compulsion n strength 1.00000000000000000000000

softly r gently 0.79541644325802100000000

softly r lightly 0.06531966511043850000000

softly r quietly 0.13926389163154100000000

blonde a fair 0.50000000000000000000000

blonde a light 0.50000000000000000000000

knife n tongue 0.94571122235639500000000

knife n point 0.03834737332385460000000

knife n harpoon 0.00093733204198110300000

knife n saw 0.01500407227776970000000

engagement n meeting 0.01365701746730160000000

engagement n arrangement 0.00051449898899228600000

engagement n match 0.00817351802967292000000

engagement n struggle 0.00051449898899228600000

engagement n business 0.18893401599606500000000

engagement n appointment 0.03134033841518730000000

engagement n fight 0.01530360822577060000000

engagement n contract 0.00102899797798457000000

engagement n conflict 0.00051449898899228600000

engagement n war 0.12142176140218000000000

engagement n action 0.01417151645629390000000

engagement n agreement 0.00349788671921301000000

engagement n vow 0.01530360822577060000000

engagement n word 0.43098053799761700000000

engagement n battle 0.15464369611996700000000

believe v gather 0.00006231438637806070000

believe v deduce 0.00092288113796999100000

believe v know 0.34679093128419100000000

believe v guess 0.01200434726374380000000

believe v suspect 0.01274477529422900000000

believe v reckon 0.00611683856353376000000

believe v assume 0.00032308596619726100000

believe v realize 0.00212033091509418000000

believe v conclude 0.00396616404230948000000

believe v understand 0.11680027281720600000000

believe v hold 0.00427610928317477000000

believe v acknowledge 0.00249872230890138000000

believe v maintain 0.00058053772531280300000

believe v think 0.25341447094736700000000

believe v suppose 0.05998762568524700000000

believe v comprehend 0.00454847329209915000000

believe v swallow 0.00030634144725454700000

believe v conceive 0.00553515200332005000000

believe v hope 0.03736137306567550000000

believe v trow 0.00018382132575474500000

believe v expect 0.02061751318567110000000

believe v consider 0.00465724401378050000000

believe v assert 0.00143670142415378000000

believe v presume 0.00077189461466854100000

believe v divine 0.00075188464334461700000

believe v affirm 0.00021803448656147300000

believe v accept 0.00258539200687401000000

believe v feel 0.03213356241520330000000

believe v imagine 0.02064164633702390000000

believe v infer 0.00014946210615590000000

believe v apprehend 0.00035458167579253800000

believe v recognize 0.00593722500889156000000

believe v regard 0.00121003418670696000000

believe v admit 0.00696965079358880000000

believe v perceive 0.00267397146073258000000

believe v credit 0.00068606789771547800000

believe v buy 0.00241420150919082000000

believe v trust 0.01706179632349720000000

believe v fancy 0.00243516094166022000000

believe v judge 0.00480730691929981000000

believe v surmise 0.00094209929452706700000

report n outcome 0.00104107445542776000000

report n address 0.02397173692618510000000

report n name 0.26261675287251000000000

report n article 0.00104107445542776000000

report n intelligence 0.00104107445542776000000

report n conclusion 0.00104107445542776000000

report n tale 0.00470502830394832000000

report n message 0.02943371301386310000000

report n communication 0.00208214891085553000000

report n decision 0.00208214891085553000000

report n discussion 0.00439262199805235000000

report n order 0.03055326846670010000000

report n distinction 0.00104107445542776000000

report n proceedings 0.00104107445542776000000

report n opinion 0.07382660856683900000000

report n rumor 0.00470502830394832000000

report n crack 0.00144482669293979000000

report n sketch 0.00104107445542776000000

report n announcement 0.00104107445542776000000

report n story 0.01308069477389420000000

report n paper 0.00104107445542776000000

report n piece 0.00144482669293979000000

report n issue 0.00144482669293979000000

report n review 0.07528045286317310000000

report n explosion 0.00104107445542776000000

report n crash 0.14554220886880100000000

report n word 0.26277339113859000000000

report n item 0.01455422088688010000000

report n news 0.01421894044958480000000

report n history 0.00537555453424713000000

report n relation 0.00208214891085553000000

report n noise 0.00144482669293979000000

report n speech 0.00678717721480384000000

report n explanation 0.00574610275937608000000

mysterious a peculiar 0.05882352941176450000000

mysterious a inviolable 0.05882352941176450000000

mysterious a strange 0.17647058823529400000000

mysterious a unnatural 0.05882352941176450000000

mysterious a dark 0.17647058823529400000000

mysterious a hidden 0.05882352941176450000000

mysterious a incredible 0.05882352941176450000000

mysterious a silent 0.05882352941176450000000

mysterious a funny 0.11764705882352900000000

mysterious a curious 0.17647058823529400000000

wheedle v move 0.25000000000000000000000

wheedle v appease 0.25000000000000000000000

wheedle v humor 0.50000000000000000000000

equal a enough 0.20043048521190300000000

equal a impartial 0.00774289603868453000000

equal a regular 0.00669756437645635000000

equal a sufficient 0.03562044452095890000000

equal a capable 0.16258559152199600000000

equal a steady 0.02148317976051200000000

equal a smooth 0.02148317976051200000000

equal a satisfactory 0.04421466964029610000000

equal a uniform 0.37165900985685700000000

equal a fair 0.05859400973275550000000

equal a competent 0.00223252145881877000000

equal a same 0.06725644812024930000000

unpromising a gloomy 0.33333333333333300000000

unpromising a bad 0.66666666666666700000000

pour v go 1.00000000000000000000000

nonsense n trash 0.20685642983703700000000

nonsense n thoughtlessness 0.00085985451832497700000

nonsense n paradox 0.00123415040271593000000

nonsense n smoke 0.00011291289838266200000

nonsense n foolery 0.00123415040271593000000

nonsense n childishness 0.05080684241611450000000

nonsense n confusion 0.00138963052673634000000

nonsense n fickleness 0.01905256590604290000000

nonsense n joke 0.00624020933006722000000

nonsense n folly 0.00510922857901578000000

nonsense n silliness 0.07239975044296310000000

nonsense n trick 0.01083491103539570000000

nonsense n stuff 0.38641735223322000000000

nonsense n madness 0.12683700275185200000000

nonsense n gewgaw 0.00123415040271593000000

nonsense n fiddle-de-dee 0.00021744931804463900000

nonsense n fun 0.00277047331860526000000

nonsense n mischief 0.00475150703577276000000

nonsense n jest 0.07175035864558790000000

nonsense n clack 0.00092879731164801900000

nonsense n trifle 0.00731816304498174000000

nonsense n contradiction 0.00017262696815203400000

nonsense n foolishness 0.00123415040271593000000

nonsense n refuse 0.00021744931804463900000

nonsense n rot 0.00123415040271593000000

nonsense n farce 0.01755158214771580000000

nonsense n truck 0.00123415040271593000000

tinkle v ring 1.00000000000000000000000

lover n man 0.73465116636760200000000

lover n darling 0.05962042905945400000000

lover n bug 0.02052207789553870000000

lover n partner 0.02094835251057960000000

lover n flame 0.00106015840157160000000

lover n dear 0.15883816307991000000000

lover n dearest 0.00055049865845799300000

lover n escort 0.00055049865845799300000

lover n admirer 0.00055049865845799300000

lover n mistress 0.00216115571848759000000

lover n sweetheart 0.00054700099148189100000

supplant v expel 0.50000000000000000000000

supplant v remove 0.50000000000000000000000

familiar a plain 0.02623320824058080000000

familiar a close 0.02796662474146220000000

familiar a conscious 0.00412183362165086000000

familiar a unreserved 0.00265303203762147000000

familiar a comfortable 0.03740352739749370000000

familiar a thick 0.00460954974874626000000

familiar a friendly 0.00296020844564161000000

familiar a broken 0.00030717640802014400000

familiar a ordinary 0.04652944282899150000000

familiar a near 0.02109123444358600000000

familiar a humble 0.00258595158155013000000

familiar a bold 0.01641460082778890000000

familiar a intimate 0.00230477487437313000000

familiar a frequent 0.00030717640802014400000

familiar a open 0.00153588204010073000000

familiar a free 0.02145041366427200000000

familiar a cordial 0.00030717640802014400000

familiar a habitual 0.00030717640802014400000

familiar a natural 0.53431387333628000000000

familiar a accessible 0.00030717640802014400000

familiar a easy 0.04398933965021480000000

familiar a courteous 0.00574575196911840000000

familiar a common 0.11683851000904300000000

familiar a impudent 0.00030717640802014400000

familiar a aware 0.03406376403605620000000

familiar a simple 0.04534541805730700000000

straightway r directly 1.00000000000000000000000

stone v sharpen 1.00000000000000000000000

solace v cheer 1.00000000000000000000000

thick a husky 0.60868630147527400000000

thick a dull 0.03852444946046040000000

thick a broad 0.00342921859986070000000

thick a stupid 0.01028765579958210000000

thick a familiar 0.00685843719972140000000

thick a tactless 0.00342921859986070000000

thick a dirty 0.01028765579958210000000

thick a intimate 0.00342921859986070000000

thick a ignorant 0.00685843719972140000000

thick a large 0.05533511831593400000000

thick a solid 0.00342921859986070000000

thick a confused 0.01028765579958210000000

thick a stiff 0.04195366806032110000000

thick a high 0.01028765579958210000000

thick a muddy 0.05533511831593400000000

thick a deep 0.00342921859986070000000

thick a heavy 0.10071800497611600000000

thick a hard 0.00342921859986070000000

thick a indistinct 0.00342921859986070000000

thick a rich 0.01371687439944280000000

thick a crowded 0.00685843719972140000000

grass n green 0.55176127742567500000000

grass n lawn 0.34625835267019400000000

grass n garden 0.10198036990413100000000

sane a right 0.83333333333333300000000

sane a healthy 0.16666666666666700000000

side n bank 0.00048591031785442500000

side n bottom 0.00155244066609901000000

side n lane 0.00201624667698747000000

side n aspect 0.00017379478398821500000

side n part 0.04755766469698420000000

side n english 0.00007355406332357700000

side n edge 0.25711925632776900000000

side n angle 0.00856904837719673000000

side n district 0.00033206215455229500000

side n slope 0.00243739030828747000000

side n interest 0.01078065941038040000000

side n position 0.00531249687251276000000

side n opinion 0.02845497724130530000000

side n rival 0.00040886465199649300000

side n party 0.18878758605289200000000

side n view 0.02746732172236020000000

side n verge 0.00035621910113058000000

side n foe 0.00007355406332357700000

side n half 0.00752930706479738000000

side n cause 0.02649443876751710000000

side n front 0.03849002133652200000000

side n face 0.19362186729599700000000

side n quarter 0.13272557863928300000000

side n perspective 0.00017379478398821500000

side n policy 0.00014076296216597100000

side n combatant 0.00014710812664715400000

side n top 0.00573466690202258000000

side n incline 0.01298340663211630000000

grand a noble 0.00808762850881315000000

grand a gentle 0.17598380427535100000000

grand a awful 0.00706810981130977000000

grand a excellent 0.01531693567993140000000

grand a extraordinary 0.00044409058590610800000

grand a wonderful 0.02585680895155440000000

grand a complete 0.09991397921489810000000

grand a splendid 0.00444342242241219000000

grand a solemn 0.00376258133643600000000

grand a chief 0.02599839187329040000000

grand a transcendent 0.00376258133643600000000

grand a pretentious 0.00011837525003504100000

grand a fancy 0.00106040082045133000000

grand a huge 0.01833164627868240000000

grand a bold 0.00266454351543665000000

grand a main 0.00376258133643600000000

grand a large 0.00097143598465034900000

grand a striking 0.00388095658647104000000

grand a sublime 0.00937476345658237000000

grand a high 0.00168739750782345000000

grand a fine 0.00505973265695742000000

grand a good 0.35450313672143400000000

grand a rich 0.08592833491322030000000

grand a ambitious 0.00023675050007008300000

grand a great 0.12986110809298000000000

grand a important 0.00788028842297713000000

grand a proud 0.00392183870941899000000

grand a showy 0.00011837525003504100000

reprobate n blackguard 0.50000000000000000000000

reprobate n monster 0.50000000000000000000000

refugee n stranger 1.00000000000000000000000

chief n duke 0.00711151408519225000000

chief n governor 0.39798139936020400000000

chief n prince 0.16755158185566600000000

chief n king 0.01659353286544860000000

chief n captain 0.01114100340355160000000

chief n lord 0.00237050469506408000000

chief n master 0.03004035341516870000000

chief n leader 0.07023201165180070000000

chief n elder 0.01114100340355160000000

chief n general 0.00237050469506408000000

chief n head 0.00237050469506408000000

chief n dean 0.27552558417244900000000

chief n emperor 0.00557050170177579000000

raspberry n bird 1.00000000000000000000000

quake v move 0.33333333333333300000000

quake v throb 0.66666666666666700000000

quail v start 0.50000000000000000000000

quail v wince 0.50000000000000000000000

attempt v try 0.05088333069667240000000

attempt v attack 0.00043530281821094400000

attempt v undertake 0.01327752741510330000000

attempt v strive 0.91365250266396700000000

attempt v aim 0.00043530281821094400000

attempt v seek 0.00125779569382741000000

attempt v force 0.02005823789400770000000

preferable a better 0.78262109000440600000000

preferable a best 0.10868945499779700000000

preferable a advantageous 0.10868945499779700000000

regard n favor 0.00460846585853342000000

regard n mind 0.01442846562843270000000

regard n observation 0.00460846585853342000000

regard n admiration 0.04971557107993630000000

regard n love 0.12642372017040700000000

regard n thought 0.05961889596985230000000

regard n value 0.04971557107993630000000

regard n concern 0.00189447326286131000000

regard n point 0.00189447326286131000000

regard n examination 0.06076347576436660000000

regard n sight 0.00990332488991595000000

regard n interest 0.00591685034626465000000

regard n attention 0.00378894652572263000000

regard n opinion 0.05609928254156240000000

regard n honor 0.00212790382054202000000

regard n view 0.33025067294812200000000

regard n esteem 0.04971557107993630000000

regard n liking 0.00212790382054202000000

regard n affection 0.13861229373268600000000

regard n wish 0.00935180892485176000000

regard n inquiry 0.01843386343413370000000

plain r surely 1.00000000000000000000000

opine v see 1.00000000000000000000000

interesting a provoking 0.01273857576676450000000

interesting a charming 0.23630921362406200000000

interesting a refreshing 0.00915617953899459000000

interesting a delightful 0.16241566496267300000000

interesting a pleasurable 0.00316198573062737000000

interesting a lovely 0.02004915948309270000000

interesting a intriguing 0.00017792766629186000000

interesting a unusual 0.00286460127611539000000

interesting a striking 0.00949236904151511000000

interesting a amusing 0.38888649859738200000000

interesting a fine 0.05892116561060570000000

interesting a readable 0.00033618950252052400000

interesting a attractive 0.04217205717734820000000

interesting a fascinating 0.01307476526928510000000

interesting a impressive 0.00316198573062737000000

interesting a exciting 0.00226129888614564000000

interesting a exceptional 0.00286460127611539000000

interesting a tempting 0.00316198573062737000000

interesting a entertaining 0.00915617953899459000000

interesting a winning 0.01963759559021210000000

nonsensical a mad 0.02245388933440260000000

nonsensical a stupid 0.03368083400160380000000

nonsensical a foolish 0.28468323977546100000000

nonsensical a absurd 0.01122694466720130000000

nonsensical a ridiculous 0.03368083400160380000000

nonsensical a silly 0.60304731355252600000000

nonsensical a idiotic 0.01122694466720130000000

cottage n home 0.99775111355481300000000

cottage n lodging 0.00224888644518656000000

malevolent a brutal 1.00000000000000000000000

convince v satisfy 0.16243571809743900000000

convince v persuade 0.30098892234211500000000

convince v demonstrate 0.00752472305855288000000

convince v overcome 0.13845490427737300000000

convince v change 0.10988338294249700000000

convince v assure 0.05560152017004070000000

convince v prove 0.20004937817177300000000

convince v turn 0.02506145094020950000000

joker n fool 0.97619047619047600000000

joker n fellow 0.02380952380952380000000

inhuman a cruel 0.11111111111111100000000

inhuman a unsympathetic 0.05555555555555530000000

inhuman a severe 0.11111111111111100000000

inhuman a cold 0.38888888888889000000000

inhuman a hard 0.22222222222222200000000

inhuman a inexorable 0.05555555555555530000000

inhuman a unfeeling 0.05555555555555530000000

inert a unconscious 1.00000000000000000000000

hurt v affront 0.00135778335123527000000

hurt v burn 0.00325567368959331000000

hurt v doctor 0.00040693812703303700000

hurt v ail 0.00406491669916661000000

hurt v incommode 0.00436471532773855000000

hurt v taint 0.00034016039323569800000

hurt v try 0.03290386963339550000000

hurt v dishearten 0.00016481866778419900000

hurt v molest 0.01108613645227260000000

hurt v burst 0.00028527955624219700000

hurt v disable 0.00037250952832731800000

hurt v pervert 0.00008722997208512030000

hurt v contaminate 0.00016481866778419900000

hurt v despond 0.00436471532773855000000

hurt v maim 0.00059680669318922500000

hurt v wound 0.00418173686675304000000

hurt v exhaust 0.00105594668590694000000

hurt v worry 0.00329976098782224000000

hurt v torment 0.01320908857832710000000

hurt v offend 0.10884587047349300000000

hurt v trouble 0.00420839145719618000000

hurt v grieve 0.00268399130495213000000

hurt v punish 0.02183747464949510000000

hurt v spoil 0.00722743133114998000000

hurt v torture 0.00257593608627907000000

hurt v break 0.02708004967061420000000

hurt v hit 0.00138262350525863000000

hurt v vex 0.00224598448236931000000

hurt v shake 0.00418148977750012000000

hurt v squeeze 0.00085583866872659400000

hurt v pierce 0.00034016039323569800000

hurt v bite 0.01096367944299890000000

hurt v strike 0.00845872378572741000000

hurt v injure 0.00208442073888963000000

hurt v kick 0.00008722997208512030000

hurt v wring 0.12194750097499800000000

hurt v strain 0.00008722997208512030000

hurt v bother 0.03944193322003560000000

hurt v scratch 0.00059348722509223500000

hurt v upset 0.00166144028522406000000

hurt v insult 0.00068817510655069000000

hurt v fret 0.02135734309866630000000

hurt v abuse 0.00483319092951154000000

hurt v whip 0.02948115221469590000000

hurt v smash 0.00008722997208512030000

hurt v suffer 0.01959896626985860000000

hurt v mortify 0.00034016039323569800000

hurt v thrash 0.00028527955624219700000

hurt v depress 0.00050497906101989700000

hurt v cut 0.00656541158858687000000

hurt v sting 0.00171802864782804000000

hurt v spend 0.00068403666527434600000

hurt v harm 0.37895505766933000000000

hurt v irritate 0.00301544748851828000000

hurt v stab 0.00026168991625536100000

hurt v degrade 0.00034016039323569800000

hurt v tear 0.00147694894687736000000

hurt v flog 0.00032963733556839800000

hurt v bruise 0.00059390016708603100000

hurt v mar 0.00018319604553079400000

hurt v displease 0.00460683478698739000000

hurt v beat 0.06879284770639520000000

hurt v cramp 0.00078171474983973300000

hurt v discompose 0.00016481866778419900000

indisputable a sure 1.00000000000000000000000

incommode v disturb 0.63547532295462100000000

incommode v tease 0.36452467704537900000000

ill n trouble 1.00000000000000000000000

hussy n adulteress 1.00000000000000000000000

name n favor 0.00571935167450936000000

name n mark 0.01547284007361700000000

name n somebody 0.07529088090833870000000

name n rank 0.04205473099513600000000

name n nickname 0.00174406124316384000000

name n star 0.03334482961552450000000

name n authority 0.01718404405136740000000

name n importance 0.00597650987728702000000

name n admiration 0.01095236220573880000000

name n credit 0.05369632901417190000000

name n style 0.00509096384970163000000

name n title 0.00058154379471301700000

name n merit 0.01244687116032880000000

name n reputation 0.10331346640394200000000

name n hero 0.00550161091809021000000

name n personage 0.01726280585904430000000

name n distinction 0.01975952803179660000000

name n appointment 0.03661143246920700000000

name n lion 0.00462174323814953000000

name n account 0.10039385438626400000000

name n character 0.19445674362831900000000

name n signature 0.04587130037325520000000

name n honor 0.08893640201707730000000

name n figure 0.06330038927225940000000

name n note 0.01483927358333020000000

name n sign 0.01434127059771520000000

name n respect 0.01123486075795170000000

support v stand 0.01232463840812140000000

support v keep 0.03386218418227920000000

support v advocate 0.00047684516251991100000

support v endure 0.00031189778721280500000

support v preserve 0.00143053548755973000000

support v hold 0.00762428403591437000000

support v assist 0.00477300384632103000000

support v take 0.31522378950634400000000

support v relieve 0.00047684516251991100000

support v experience 0.00047684516251991100000

support v encourage 0.00110064073694552000000

support v submit 0.00407108690677767000000

support v approve 0.00249518229770244000000

support v handle 0.00589937643542333000000

support v defend 0.00625984330827105000000

support v bear 0.10456674888552300000000

support v guard 0.00423603428208478000000

support v embed 0.00047684516251991100000

support v justify 0.00637622159794324000000

support v feed 0.00700048846148876000000

support v carry 0.02612270488161540000000

support v help 0.20223332822905900000000

support v continue 0.06918463849792200000000

support v raise 0.00143053548755973000000

support v watch 0.02461797575002400000000

support v strengthen 0.00047684516251991100000

support v stay 0.02140435354600690000000

support v reassure 0.00047684516251991100000

support v nurse 0.00047684516251991100000

support v comfort 0.00047684516251991100000

support v suffer 0.01044591462296660000000

support v subscribe 0.00078874294973271600000

support v accept 0.00141253852415833000000

support v assure 0.06908078528064750000000

support v oblige 0.00524984900884094000000

support v persevere 0.00047684516251991100000

support v mind 0.02099168878138690000000

support v sympathize 0.00031189778721280500000

support v advance 0.01420510374253150000000

support v befriend 0.00589937643542333000000

support v stake 0.00477300384632103000000

game n meeting 0.00401014622444898000000

game n engagement 0.03638463334353110000000

game n play 0.35102723044168300000000

game n foolery 0.00031575073101468000000

game n stratagem 0.00015299140015300600000

game n match 0.02037621850869910000000

game n backbone 0.00031575073101468000000

game n joke 0.05659315473720820000000

game n trade 0.00046874213116768500000

game n scheme 0.00015299140015300600000

game n profession 0.00383517986963587000000

game n round 0.00782758780940171000000

game n amusement 0.25469243340471700000000

game n nerve 0.00045897420045901900000

game n business 0.06994831220416060000000

game n trick 0.06540485340640580000000

game n hoax 0.00015299140015300600000

game n spirit 0.00597863914340889000000

game n fun 0.01307950616893270000000

game n entertainment 0.03528298221459680000000

game n duel 0.00015299140015300600000

game n gaiety 0.00065347641668946100000

game n tie 0.01389934717926630000000

game n line 0.00499275260499324000000

game n bout 0.00031575073101468000000

game n lark 0.00065347641668946100000

game n plan 0.05287313578024810000000

harangue n talk 1.00000000000000000000000

extremely r quite 0.02305612235081720000000

extremely r tremendously 0.00042239552072193200000

extremely r seriously 0.01485424247872130000000

extremely r exceedingly 0.00463624161084698000000

extremely r very 0.93289203525256600000000

extremely r awfully 0.01889628897030740000000

extremely r immensely 0.00524267381601928000000

sensation n suspicion 0.00047596959895012200000

sensation n experience 0.02984245277368000000000

sensation n wonder 0.08545040530049220000000

sensation n sentiment 0.00023798479947506100000

sensation n consciousness 0.00118681123543211000000

sensation n excitement 0.00023798479947506100000

sensation n agitation 0.00014988860925039800000

sensation n feeling 0.00902460868402981000000

sensation n genius 0.00014988860925039800000

sensation n apprehension 0.24952706224960200000000

sensation n perception 0.07129344635702910000000

sensation n sense 0.00014988860925039800000

sensation n response 0.00118681123543211000000

sensation n fear 0.00014988860925039800000

sensation n presentiment 0.00483940491284634000000

sensation n disturbance 0.00321970402902712000000

sensation n joy 0.00309011841795173000000

sensation n surprise 0.00186054070403388000000

sensation n foreboding 0.00049980383024457100000

sensation n terror 0.03696697218512620000000

sensation n impression 0.50046036445017100000000

vanish v quit 0.00568235792975113000000

vanish v retire 0.00165728440403960000000

vanish v depart 0.01342684589129480000000

vanish v evaporate 0.01056918574933710000000

vanish v die 0.07505582324595270000000

vanish v expire 0.00568235792975113000000

vanish v go 0.67019255690288700000000

vanish v leave 0.12510528155354700000000

vanish v end 0.00227515613086695000000

vanish v vacate 0.00284117896487556000000

vanish v disappear 0.08751197129769700000000

exaltation n excitement 0.66666666666666700000000

exaltation n blessing 0.33333333333333300000000

eradicate v destroy 0.12500000000000000000000

eradicate v crush 0.06249999999999990000000

eradicate v stop 0.12500000000000000000000

eradicate v conquer 0.06249999999999990000000

eradicate v extinguish 0.06249999999999990000000

eradicate v kill 0.25000000000000100000000

eradicate v efface 0.06249999999999990000000

eradicate v remove 0.06249999999999990000000

eradicate v annihilate 0.06249999999999990000000

eradicate v ruin 0.06249999999999990000000

eradicate v cancel 0.06249999999999990000000

snow n blow 0.07351047047151360000000

snow n snowstorm 0.41098973533237200000000

snow n horse 0.51549979419611400000000

encumbrance n son 0.06562554834181430000000

encumbrance n daughter 0.63537462712756600000000

encumbrance n difficulty 0.29899982453061900000000

electrify v charge 0.10000000000000000000000

electrify v disturb 0.05000000000000000000000

electrify v rouse 0.05000000000000000000000

electrify v send 0.25000000000000000000000

electrify v fire 0.10000000000000000000000

electrify v strike 0.25000000000000000000000

electrify v light 0.10000000000000000000000

electrify v stun 0.10000000000000000000000

mount v rouse 0.00054389978312862200000

mount v build 0.00027194989156431000000

mount v show 0.00135974945782156000000

mount v state 0.00432199123610203000000

mount v grow 0.00027194989156431000000

mount v raise 0.00054389978312862200000

mount v illuminate 0.00027194989156431000000

mount v produce 0.02604973538773070000000

mount v ride 0.36218286558535000000000

mount v arrange 0.60363810930891700000000

mount v increase 0.00054389978312862200000

dishonest a contemptible 1.00000000000000000000000

lamp n light 0.11045557631234300000000

lamp n lantern 0.00540297256043733000000

lamp n spot 0.00145161408705312000000

lamp n candle 0.88268983704016700000000

cue n place 0.25000000000000000000000

cue n part 0.25000000000000000000000

cue n idea 0.25000000000000000000000

cue n position 0.25000000000000000000000

crucifixion n suffering 1.00000000000000000000000

deliver v have 0.30158739000761800000000

deliver v direct 0.00004530013294301720000

deliver v keep 0.00631802053513520000000

deliver v fling 0.00006969998995738950000

deliver v pass 0.01266697324998120000000

deliver v bring 0.01921372839599090000000

deliver v return 0.00047482431510458000000

deliver v commit 0.00034849994978694800000

deliver v announce 0.00006969998995738950000

deliver v remit 0.02088012556294940000000

deliver v preserve 0.00020909996987216800000

deliver v abandon 0.01140614716631620000000

deliver v shoot 0.00078120995654316500000

deliver v take 0.23382520406298000000000

deliver v pronounce 0.00024626938321928500000

deliver v communicate 0.00024626938321928500000

deliver v send 0.02192741935710680000000

deliver v convey 0.00022855493188529500000

deliver v grant 0.00011500012290040700000

deliver v read 0.00320352105715751000000

deliver v bear 0.00457224887786192000000

deliver v release 0.00031915519777132900000

deliver v fire 0.00041325504474309100000

deliver v discharge 0.00013939997991477900000

deliver v carry 0.00047695397284929600000

deliver v help 0.03368901012070680000000

deliver v throw 0.00265747052619356000000

deliver v give 0.16397830978169100000000

deliver v tell 0.02007205259462810000000

deliver v say 0.03067122085717920000000

deliver v inflict 0.04872029298021530000000

deliver v transfer 0.00027385506482831200000

deliver v express 0.00024626938321928500000

deliver v administer 0.00036795491180007400000

deliver v relinquish 0.00033755399293451700000

deliver v save 0.01624054195369180000000

deliver v free 0.00024626938321928500000

deliver v drop 0.00016030025584342400000

deliver v address 0.00065670919070584600000

deliver v publish 0.00006969998995738950000

deliver v rescue 0.00024626938321928500000

deliver v acquit 0.00009060026588603480000

deliver v produce 0.00006969998995738950000

deliver v entrust 0.00013939997991477900000

deliver v surrender 0.00013939997991477900000

deliver v present 0.00826854296264580000000

deliver v dispense 0.00006969998995738950000

deliver v speak 0.03142215808389520000000

deliver v consign 0.00006969998995738950000

deliver v declare 0.00110394782420140000000

deliver v cast 0.00006969998995738950000

deliver v utter 0.00006969998995738950000

deliver v liberate 0.00006969998995738950000

credulous a easy 0.01288128870943610000000

credulous a simple 0.97181719171656600000000

credulous a green 0.01530151957399770000000

acquaint v teach 0.08099042747487560000000

acquaint v introduce 0.01403381501939650000000

acquaint v reveal 0.01084538247168790000000

acquaint v warn 0.00188735433505603000000

acquaint v communicate 0.00182896794175049000000

acquaint v accustom 0.00277264510927851000000

acquaint v advise 0.00897628888584102000000

acquaint v tell 0.67858833494923500000000

acquaint v mention 0.11891713787530700000000

acquaint v disclose 0.00182896794175049000000

acquaint v notify 0.00158705988065111000000

acquaint v present 0.01041249286914140000000

acquaint v inform 0.06733112524602900000000

hour n minute 0.60170769083788900000000

hour n term 0.00162504983748467000000

hour n moment 0.39666725932462600000000

sentiment n sentimentality 0.00212253968479416000000

sentiment n mind 0.03559581037911250000000

sentiment n conviction 0.25842921860333500000000

sentiment n attitude 0.00326579603862107000000

sentiment n notion 0.00865413176203629000000

sentiment n thought 0.03691399456338870000000

sentiment n belief 0.02923914613231970000000

sentiment n feeling 0.27361494244261400000000

sentiment n thinking 0.00212253968479416000000

sentiment n idea 0.28635018055137900000000

sentiment n theory 0.02499406676273130000000

sentiment n position 0.00751087540820940000000

sentiment n opinion 0.01926683018600710000000

sentiment n response 0.00212253968479416000000

sentiment n view 0.00979738811586320000000

parent n guardian 0.00139766792770943000000

parent n mother 0.73431868507575300000000

parent n agent 0.00021799181395677200000

parent n author 0.02629347991519730000000

parent n cause 0.01069930867611390000000

parent n father 0.22707286659127000000000

burned a burnt 1.00000000000000000000000

bug n fault 1.00000000000000000000000

beneficial a worthy 0.02564102564102560000000

beneficial a helpful 0.02564102564102560000000

beneficial a useful 0.02564102564102560000000

beneficial a valuable 0.02564102564102560000000

beneficial a good 0.76923076923076900000000

beneficial a obliging 0.05128205128205130000000

beneficial a favorable 0.02564102564102560000000

beneficial a profitable 0.05128205128205130000000

beholder n witness 1.00000000000000000000000

whisper n sigh 0.94659248091345700000000

whisper n suggestion 0.01068150381730860000000

whisper n story 0.03204451145192600000000

whisper n whispering 0.01068150381730860000000

behead v execute 0.33333333333333300000000

behead v kill 0.66666666666666700000000

beat a weary 0.01986326367787600000000

beat a dead 0.98013673632212400000000

bargain v foresee 0.83501542937910800000000

bargain v expect 0.16498457062089200000000

unable a useless 1.00000000000000000000000

truly r devotedly 0.03762180696603430000000

truly r completely 0.00084429705750552300000

truly r surely 0.01407190522718320000000

truly r absolutely 0.00130179262858250000000

truly r scrupulously 0.00017721058391914400000

truly r actually 0.00099561524532897700000

truly r indeed 0.22886437885488900000000

truly r utterly 0.00276630933573782000000

truly r positively 0.00223274818789521000000

truly r really 0.54944593823900800000000

truly r precisely 0.00049797229604281000000

truly r fairly 0.00072769452545520900000

truly r unfeignedly 0.00223939327178776000000

truly r heartily 0.00391893822562857000000

truly r sincerely 0.06113543631980570000000

truly r certainly 0.07034705873032920000000

truly r decidedly 0.00262823621034226000000

truly r rightly 0.00138825855963226000000

truly r assuredly 0.00017721058391914400000

truly r perfectly 0.00333127978262021000000

truly r honestly 0.00138825855963226000000

truly r simply 0.00030499415431388200000

truly r exactly 0.01309529415836480000000

truly r unquestionably 0.00049797229604281000000

agile a lively 0.50000000000000000000000

agile a ready 0.50000000000000000000000

yearn v mourn 1.00000000000000000000000

pretend v hazard 0.00009563125075084880000

pretend v guess 0.00035046820672354100000

pretend v venture 0.00740474998546403000000

pretend v deceive 0.00593987521992341000000

pretend v affect 0.00085109794831799300000

pretend v make 0.00984522919461488000000

pretend v suppose 0.30912799412586100000000

pretend v invent 0.02949493928318100000000

pretend v profess 0.03443556928976770000000

pretend v play 0.00163051307992105000000

pretend v offer 0.01464969950739770000000

pretend v cheat 0.00015183798253455100000

pretend v seem 0.56507862070378200000000

pretend v imagine 0.00825882532996144000000

pretend v feign 0.01188838122760100000000

pretend v act 0.00030367596506910100000

pretend v mimic 0.00019863022418899100000

pretend v delude 0.00019863022418899100000

pretend v beguile 0.00009563125075084880000

warden n chancellor 1.00000000000000000000000

vouch v show 0.66666666666666700000000

vouch v protest 0.33333333333333300000000

lane n path 0.00248480607481463000000

lane n course 0.08110407028194960000000

lane n street 0.62635393076046500000000

lane n terrace 0.00607308832981851000000

lane n way 0.23032619117766400000000

lane n highway 0.00543107613495198000000

lane n passage 0.01534092732725580000000

lane n pathway 0.00248480607481463000000

lane n walk 0.03040110383826640000000

vexed a angry 1.00000000000000000000000

become v suit 0.00080839610613259200000

become v fit 0.00109765483990915000000

become v grow 0.24123774550683900000000

become v happen 0.21011195857484400000000

become v get 0.17310585699977000000000

become v convert 0.00054882741995457400000

become v befall 0.02775888847889070000000

become v go 0.30651661745671100000000

become v occur 0.01053526701143110000000

become v turn 0.02437764935587580000000

become v enrich 0.00390113824964225000000

false a dishonorable 0.00131679401477642000000

false a deceitful 0.33091293821093600000000

false a disappointing 0.00977697317441401000000

false a perfidious 0.03246692978673330000000

false a hollow 0.43573761734743200000000

false a venal 0.00051681852491270600000

false a suspicious 0.00211676950464015000000

false a mean 0.00079997548986371800000

false a cunning 0.00297538292184458000000

false a mistaken 0.00082057571707051200000

false a wrong 0.11901969274052400000000

false a imaginary 0.00230663174088052000000

false a amiss 0.03326690527659710000000

false a queer 0.00051681852491270600000

false a fanciful 0.00103363704982542000000

false a assumed 0.00621208403861685000000

false a base 0.00217540743198087000000

false a unreal 0.01802804850403920000000

undoing n woe 0.14285714285714300000000

undoing n doom 0.14285714285714300000000

undoing n fault 0.71428571428571400000000

unbecoming a offensive 0.10082723894017500000000

unbecoming a improper 0.10082723894017500000000

unbecoming a unfit 0.79834552211965100000000

twilight a dark 1.00000000000000000000000

conviction n faith 0.04865954664341760000000

conviction n opinion 0.25986268526591100000000

conviction n persuasion 0.64281822144725400000000

conviction n teaching 0.04865954664341760000000

trustee n guardian 0.25000000000000000000000

trustee n governor 0.50000000000000100000000

trustee n officer 0.25000000000000000000000

top v climb 1.00000000000000000000000

breathe v expel 0.00088473990700086800000

breathe v blow 0.20349017861020000000000

breathe v pronounce 0.00034286466488660400000

breathe v sigh 0.00034286466488660400000

breathe v move 0.06607995861507940000000

breathe v break 0.00356639768225315000000

breathe v smell 0.00233879311036568000000

breathe v say 0.51894831017632400000000

breathe v express 0.00059675135061361600000

breathe v pant 0.05985005253241170000000

breathe v whisper 0.07399642858552720000000

breathe v rest 0.04116784372979010000000

breathe v pause 0.01071865347592060000000

breathe v exist 0.01767616289474050000000

telling a sure 1.00000000000000000000000

taint v hurt 0.75000000000000100000000

taint v degrade 0.12499999999999900000000

taint v ruin 0.12499999999999900000000

tableau n picture 1.00000000000000000000000

anywhere r everywhere 0.98984126984127000000000

anywhere r wherever 0.01015873015873020000000

realize v gain 0.00003974017603984760000

realize v register 0.00151675005218752000000

realize v know 0.58741552026916300000000

realize v manage 0.00930381157322441000000

realize v return 0.00461407942497237000000

realize v win 0.00011922052811954300000

realize v believe 0.02389565973952720000000

realize v clear 0.00026543125913281600000

realize v understand 0.09207413921710490000000

realize v make 0.00362860328528808000000

realize v experience 0.00210768745322007000000

realize v absorb 0.00163342313312502000000

realize v comprehend 0.06638312549056280000000

realize v receive 0.01313284688401260000000

realize v complete 0.00068868651018244200000

realize v read 0.00362191913861031000000

realize v grasp 0.00003974017603984760000

realize v penetrate 0.00374726483481623000000

realize v conceive 0.00378553094215479000000

realize v accomplish 0.00155374395589941000000

realize v discharge 0.00155374395589941000000

realize v attain 0.00027428849167653800000

realize v execute 0.00056912862560419700000

realize v get 0.00811983905041836000000

realize v reach 0.00015896070415939100000

realize v fulfil 0.00031255459901510200000

realize v catch 0.00035229477505495000000

realize v discern 0.00079629377739844900000

realize v learn 0.00067168995673892000000

realize v produce 0.00401973499433081000000

realize v imagine 0.00695027417774244000000

realize v apprehend 0.00001913305366928230000

realize v discover 0.00226156351975114000000

realize v recognize 0.00109211780674517000000

realize v perceive 0.00007948035207969540000

realize v see 0.15110960596864000000000

realize v obtain 0.00028456431280209800000

realize v perform 0.00180780783489067000000

schoolmistress n mistress 1.00000000000000000000000

savor v know 1.00000000000000000000000

endure v be 0.50893451835586400000000

endure v stand 0.10131346580200400000000

endure v know 0.13228316455473100000000

endure v permit 0.00229359302382490000000

endure v support 0.00001654641474247510000

endure v last 0.00098379116167413000000

endure v hold 0.00406992840318750000000

endure v live 0.01751004265403010000000

endure v run 0.00031529156412704600000

endure v brook 0.00265239028321877000000

endure v take 0.01213548140076610000000

endure v meet 0.01068905015574900000000

endure v remain 0.00108260827886480000000

endure v encounter 0.00094307210070000800000

endure v resist 0.01008048320149390000000

endure v submit 0.00413336802683260000000

endure v swallow 0.01357724298022090000000

endure v brave 0.00028443863627011000000

endure v bear 0.06651160592066630000000

endure v persist 0.00013100382622498400000

endure v prevail 0.00107257568318872000000

endure v continue 0.00113350867178344000000

endure v sustain 0.00303130318082145000000

endure v taste 0.00132370963531989000000

endure v allow 0.00418604598634663000000

endure v survive 0.00043036272608881400000

endure v stay 0.00694027469772650000000

endure v abide 0.00192474926815861000000

endure v wear 0.00085217358496988600000

endure v suffer 0.00117362481925576000000

endure v eat 0.00605208522211469000000

endure v feel 0.02361501888024080000000

endure v go 0.04132405443390000000000

endure v persevere 0.00001593266426321520000

endure v rest 0.00279346926787097000000

endure v bide 0.00085217358496988600000

endure v avoid 0.00112098742645763000000

endure v exist 0.00372921461503029000000

endure v face 0.00848764890630007000000

remit v spare 0.01585503963759910000000

remit v return 0.03171007927519820000000

remit v deliver 0.76217440543601400000000

remit v wait 0.04756511891279730000000

remit v stop 0.03171007927519820000000

remit v pay 0.01585503963759910000000

remit v forget 0.03171007927519820000000

remit v stay 0.03171007927519820000000

remit v disregard 0.01585503963759910000000

remit v desist 0.01585503963759910000000

early r soon 0.89474408266409800000000

early r beforehand 0.00315426598553418000000

early r first 0.09462797956602540000000

early r forward 0.00123682767658129000000

early r presently 0.00623684410776076000000

regal a sublime 1.00000000000000000000000

realism n reality 1.00000000000000000000000

ravish v steal 0.03012991409589090000000

ravish v excite 0.01506495704794540000000

ravish v cheer 0.03012991409589090000000

ravish v please 0.33333333333333300000000

ravish v charm 0.56121196733104800000000

ravish v enchant 0.01506495704794540000000

ravish v fascinate 0.01506495704794540000000

talk v criticize 0.00014668386405571100000

talk v persuade 0.00258858375374366000000

talk v discourse 0.00025533857817105300000

talk v mutter 0.00888170019382233000000

talk v depreciate 0.00156648548422794000000

talk v negotiate 0.00162215096720434000000

talk v relate 0.00068496265623736000000

talk v commune 0.00025533857817105300000

talk v breathe 0.00235224926144457000000

talk v count 0.00342174696957719000000

talk v reveal 0.00773408924057113000000

talk v pronounce 0.00088075229041859500000

talk v communicate 0.00001656498960352820000

talk v convey 0.00045899984262096500000

talk v advise 0.00127062827175661000000

talk v read 0.03896345110159070000000

talk v discuss 0.00138671558682807000000

talk v state 0.00443742011531853000000

talk v rattle 0.00020447444744662000000

talk v consult 0.00198375082500784000000

talk v exchange 0.00005917718120702520000

talk v digress 0.00026918557264887600000

talk v confide 0.00037536986265036200000

talk v argue 0.00915307964719047000000

talk v sway 0.00034907046129713500000

talk v consider 0.02243180979920670000000

talk v converse 0.00036963608441940800000

talk v tell 0.14664619463535900000000

talk v observe 0.00253364999188849000000

talk v stammer 0.00051259159741555300000

talk v influence 0.00064539312696806700000

talk v boast 0.00085603620848678700000

talk v say 0.45644321404966700000000

talk v notice 0.01547956484987710000000

talk v disclose 0.00089999771859204600000

talk v confer 0.00142195039631051000000

talk v express 0.01067105494458540000000

talk v preach 0.00052020060677458600000

talk v enlighten 0.00008978791495893720000

talk v exaggerate 0.00011665804184619200000

talk v address 0.00219845118240934000000

talk v articulate 0.00001656498960352820000

talk v pitch 0.00013194529398312800000

talk v notify 0.00010229925539384900000

talk v whisper 0.00225047181482898000000

talk v sing 0.02893062784214950000000

talk v air 0.00002574842805086250000

talk v mumble 0.00002574842805086250000

talk v speak 0.17743568024139800000000

talk v advertise 0.00005195773234568770000

talk v declare 0.01547710597278830000000

talk v pan 0.00023271364086475700000

talk v knock 0.00126732363074573000000

talk v describe 0.00351104674164817000000

talk v spill 0.00010206453181740400000

talk v inform 0.00062174751903291400000

talk v utter 0.00008679517463964790000

talk v remark 0.00530119298501010000000

talk v visit 0.01120498427497060000000

talk v brag 0.00005591006920651250000

talk v rehearse 0.00178119690103024000000

talk v crow 0.00023271364086475700000

drag v creep 1.00000000000000000000000

quarrelsome a quick 0.00930916217540420000000

quarrelsome a sharp 0.00930916217540420000000

quarrelsome a touchy 0.00930916217540420000000

quarrelsome a disagreeable 0.01861832435080840000000

quarrelsome a mean 0.00930916217540420000000

quarrelsome a contrary 0.00930916217540420000000

quarrelsome a irritable 0.87898089171974500000000

quarrelsome a cross 0.01861832435080840000000

quarrelsome a fiery 0.00930916217540420000000

quarrelsome a hot 0.00930916217540420000000

quarrelsome a difficult 0.01861832435080840000000

printer n devil 1.00000000000000000000000

delightful a appealing 0.02996607315111960000000

delightful a charming 0.15053353760179600000000

delightful a refreshing 0.00022469448170573600000

delightful a gratifying 0.00067408344511720900000

delightful a inspiring 0.00399547642014927000000

delightful a pleasurable 0.00279344749713826000000

delightful a prepossessing 0.00397140728508813000000

delightful a lovely 0.03409617986577990000000

delightful a welcome 0.02400312962350970000000

delightful a amiable 0.04738654920391680000000

delightful a merry 0.00118173589315278000000

delightful a glad 0.49086583684741400000000

delightful a satisfactory 0.00769520432282602000000

delightful a amusing 0.00289173327397883000000

delightful a delicious 0.02622560537266070000000

delightful a attractive 0.00388389078535027000000

delightful a joyous 0.00014850284036446000000

delightful a fascinating 0.01230456164343790000000

delightful a sweet 0.06771733138120000000000

delightful a pleasant 0.05583453415759670000000

delightful a tempting 0.00653920664102821000000

delightful a adorable 0.00442889022116724000000

delightful a entertaining 0.00022469448170573600000

delightful a agreeable 0.02241369356279620000000

predominate v prevail 0.25000000000000000000000

predominate v tell 0.75000000000000000000000

plebeian a plain 0.28571428571428600000000

plebeian a ordinary 0.07142857142857140000000

plebeian a rude 0.14285714285714300000000

plebeian a low 0.14285714285714300000000

plebeian a common 0.07142857142857140000000

plebeian a simple 0.28571428571428600000000

pioneer n guide 1.00000000000000000000000

pickle v can 1.00000000000000000000000

authority n liberty 0.23225943357632200000000

authority n police 0.38709905596053600000000

authority n judge 0.02639311745185470000000

authority n command 0.02639311745185470000000

authority n governor 0.06277281988549240000000

authority n right 0.00348912519143197000000

authority n master 0.01015156674338120000000

authority n power 0.00116304173047732000000

authority n court 0.00116304173047732000000

authority n confidence 0.23342247530679900000000

authority n force 0.01569320497137310000000

picket n post 0.50000000000000000000000

picket n watch 0.50000000000000000000000

pernicious a dangerous 0.25000000000000000000000

pernicious a wicked 0.25000000000000000000000

pernicious a fatal 0.25000000000000000000000

pernicious a bad 0.25000000000000000000000

peradventure n question 0.37500000000000000000000

peradventure n doubt 0.62500000000000000000000

priest n clerk 0.04143793037458810000000

priest n minister 0.09360556286731250000000

priest n clergyman 0.13912836607736900000000

priest n curate 0.02195241271708940000000

priest n parson 0.00755697355568770000000

priest n father 0.69631875440795400000000

parasite n dependent 1.00000000000000000000000

forest n plantation 1.00000000000000000000000

attach v conciliate 0.01421009959499070000000

attach v win 0.08566397525912550000000

attach v lay 0.00202592462845055000000

attach v stick 0.00101296231422527000000

attach v secure 0.28420199189981400000000

attach v send 0.00202592462845055000000

attach v fasten 0.01421009959499070000000

attach v unite 0.01752451960776330000000

attach v add 0.01597096188789970000000

attach v charm 0.00101296231422527000000

attach v attribute 0.00876225980388167000000

attach v fascinate 0.00101296231422527000000

attach v fix 0.01078818443233220000000

attach v bind 0.03774530065957470000000

attach v put 0.50383187106005000000000

aspect n feature 0.00181536151017030000000

aspect n side 0.00181536151017030000000

aspect n part 0.64445333611045600000000

aspect n prospect 0.00181536151017030000000

aspect n light 0.02558525128396270000000

aspect n air 0.00544608453051090000000

aspect n complexion 0.00181536151017030000000

aspect n nature 0.09313813081345950000000

aspect n expression 0.00181536151017030000000

aspect n position 0.00181536151017030000000

aspect n character 0.00181536151017030000000

aspect n appearance 0.14554463585887100000000

aspect n view 0.02013916675345180000000

aspect n presence 0.02013916675345180000000

aspect n face 0.02740061279413300000000

aspect n perspective 0.00181536151017030000000

aspect n situation 0.00363072302034060000000

missy n girl 1.00000000000000000000000

member n feature 0.00241754033429022000000

member n arm 0.01974628208460090000000

member n part 0.02483134918087840000000

member n comrade 0.00241754033429022000000

member n sister 0.38934558447444000000000

member n brother 0.19028595743103900000000

member n chapter 0.01274364750942730000000

member n foot 0.01274364750942730000000

member n hand 0.32854566880157600000000

member n fellow 0.00483508066858045000000

member n ear 0.00483508066858045000000

member n leg 0.00483508066858045000000

member n nose 0.00241754033429022000000

loath a afraid 1.00000000000000000000000

date n meeting 0.00807252292099004000000

date n baby 0.00065300743293922100000

date n man 0.04557167088416410000000

date n year 0.00497755996896056000000

date n lover 0.01008080224599920000000

date n hour 0.00312592818690448000000

date n girl 0.02291462446495820000000

date n course 0.00604836638150220000000

date n interview 0.00497755996896056000000

date n time 0.62302811008179400000000

date n trick 0.00163251858234806000000

date n day 0.08943383362492350000000

date n term 0.00115081063445803000000

date n tryst 0.00032650371646960900000

date n month 0.00465105625249095000000

date n epoch 0.00032650371646960900000

date n age 0.08268747775523550000000

date n while 0.00032650371646960900000

date n visit 0.00465105625249095000000

date n companion 0.00627715730691156000000

date n quarter 0.00032650371646960900000

date n woman 0.00986869818807728000000

date n moment 0.05265894675518080000000

date n sweetheart 0.00032650371646960900000

date n lady 0.01590577352836310000000

leak v pass 0.85714285714285700000000

leak v tell 0.14285714285714300000000

will v decree 0.00015077742773876800000

will v purpose 0.00019915521203997700000

will v crave 0.00004251372677109060000

will v wish 0.14697543333068200000000

will v prefer 0.01333550664974370000000

will v like 0.14623330930393100000000

will v command 0.00181661769646328000000

will v incline 0.00098005328030198400000

will v please 0.14245200177578700000000

will v effect 0.00033700772984462900000

will v elect 0.00417489329728509000000

will v dictate 0.00142868368016548000000

will v settle 0.01375977140775570000000

will v devise 0.00003107941971391640000

will v choose 0.02139116698056130000000

will v leave 0.09099984713450090000000

will v cause 0.00142582622869486000000

will v decide 0.01478019801724740000000

will v ordain 0.00013864491577227200000

will v determine 0.01280264614384440000000

will v enjoin 0.00064210387330130000000

will v want 0.35607444983811700000000

will v prescribe 0.00003107941971391640000

will v resolve 0.01733380381388870000000

will v order 0.00699836213665875000000

will v desire 0.00546506755947542000000

try v push 0.00954509322588754000000

try v venture 0.00239535655419959000000

try v sound 0.02430608530360840000000

try v attempt 0.00454891002515862000000

try v check 0.00340549667620635000000

try v struggle 0.05999707429042210000000

try v endeavor 0.08160593117508760000000

try v experience 0.00378830681504102000000

try v tire 0.05136900700575280000000

try v exhaust 0.00858718724731132000000

try v attack 0.00667917949179796000000

try v undertake 0.00059349768127336500000

try v complete 0.00015566224353855500000

try v torment 0.01850148276467880000000

try v trouble 0.00446248109710498000000

try v weigh 0.00061822675132642100000

try v torture 0.00410804164938833000000

try v strive 0.00026490863843882400000

try v vex 0.00414038279393383000000

try v work 0.07204816496974550000000

try v consider 0.02922880311939910000000

try v taste 0.00851411570867828000000

try v wring 0.00786563192049038000000

try v strain 0.00015566224353855500000

try v risk 0.00239783821143073000000

try v annoy 0.08258077392850870000000

try v bother 0.00677806806438057000000

try v upset 0.00156325906507831000000

try v aim 0.00098495396625870600000

try v seek 0.00495427014454515000000

try v labor 0.00070961679282684900000

try v hear 0.21295618670011500000000

try v harass 0.00149735653532271000000

try v feel 0.18370818573298200000000

try v speculate 0.00829801042810763000000

try v prove 0.00523088333004880000000

try v plague 0.00030838700556304800000

try v irritate 0.00048111885126025800000

try v weary 0.00039817615490582900000

try v drain 0.00037155095035095600000

try v test 0.00518276693338950000000

try v decide 0.01529273417631620000000

try v analyze 0.00081218558190517000000

try v render 0.00133780215041127000000

try v examine 0.01858491880392050000000

try v propose 0.01241398050422270000000

try v fathom 0.00081218558190517000000

try v judge 0.02546010101423620000000

clear v gain 0.00277127543623857000000

clear v satisfy 0.00531295799795770000000

clear v reckon 0.00161077969044968000000

clear v pass 0.48750194186359000000000

clear v permit 0.00518871838374311000000

clear v win 0.00346409429529821000000

clear v realize 0.00302675239051681000000

clear v make 0.18933842288702400000000

clear v ratify 0.00302675239051681000000

clear v separate 0.00161077969044968000000

clear v wash 0.00069281885905964200000

clear v relieve 0.00069281885905964200000

clear v show 0.00091796083139004100000

clear v wipe 0.01220874913821910000000

clear v exhaust 0.00022514197233039900000

clear v open 0.00207845657717893000000

clear v sweep 0.11175701134215900000000

clear v flush 0.00022514197233039900000

clear v dispose 0.00371957124957646000000

clear v lose 0.00801911256811154000000

clear v stop 0.00764689152941487000000

clear v release 0.00045028394466079900000

clear v discharge 0.00138563771811928000000

clear v justify 0.00069281885905964200000

clear v break 0.10743449390639600000000

clear v earn 0.00022514197233039900000

clear v allow 0.00325189436284721000000

clear v strain 0.00069281885905964200000

clear v empty 0.00069281885905964200000

clear v detach 0.00302675239051681000000

clear v pardon 0.00022514197233039900000

clear v explain 0.00091796083139004100000

clear v settle 0.00136824477605084000000

clear v acquit 0.00045028394466079900000

clear v lighten 0.00069281885905964200000

clear v leave 0.01428023845783190000000

clear v forgive 0.00322155938089937000000

clear v drain 0.00069281885905964200000

clear v remove 0.00787648440393431000000

clear v disappear 0.00069281885905964200000

clear v liberate 0.00069281885905964200000

intentional a conscious 0.93503558499291700000000

intentional a intended 0.06496441500708340000000

injudicious a stupid 0.06354610261333250000000

injudicious a foolish 0.76279905021026400000000

injudicious a silly 0.00582033024371731000000

injudicious a unreasonable 0.01164066048743470000000

injudicious a wrong 0.11010874456307100000000

injudicious a imprudent 0.04608511188218050000000

cheerful a alive 0.00267997317236754000000

cheerful a friendly 0.00017193600650773000000

cheerful a pleased 0.04080830162865420000000

cheerful a delighted 0.03373393367567710000000

cheerful a eager 0.00151444691446426000000

cheerful a generous 0.28783362611976800000000

cheerful a active 0.00207660850629407000000

cheerful a gay 0.06944482899572300000000

cheerful a amiable 0.00072273431193504500000

cheerful a lively 0.04417195165142760000000

cheerful a laughing 0.00349034094942171000000

cheerful a happy 0.06391693977742990000000

cheerful a earnest 0.00056216159182980900000

cheerful a excited 0.00160315928602284000000

cheerful a good-natured 0.00086916354220273400000

cheerful a contented 0.00137024722768116000000

cheerful a merry 0.00134904481752650000000

cheerful a sincere 0.00125938912752481000000

cheerful a positive 0.00311760620048710000000

cheerful a glad 0.01961149385200680000000

cheerful a joyful 0.19082031122249700000000

cheerful a sparkling 0.01060112840124980000000

cheerful a animated 0.02869478364248070000000

cheerful a attractive 0.02388738047293020000000

cheerful a joyous 0.00017193600650773000000

cheerful a sunny 0.01506476141230240000000

cheerful a hopeful 0.00124799039310076000000

cheerful a genial 0.01363002223017840000000

cheerful a good-humoured 0.03367417256867590000000

cheerful a sanguine 0.00034387201301546100000

cheerful a energetic 0.00039022558532207900000

cheerful a bright 0.00733924273932681000000

cheerful a fresh 0.04403545643596080000000

cheerful a pleasant 0.01032747544159300000000

cheerful a light-hearted 0.00017193600650773000000

cheerful a agreeable 0.03929141807339920000000

incontestable a undeniable 0.00869565217391300000000

incontestable a impregnable 0.00869565217391300000000

incontestable a sure 0.86086956521739100000000

incontestable a certain 0.10434782608695700000000

incontestable a solid 0.00869565217391300000000

incontestable a positive 0.00869565217391300000000

previous a past 0.01419153705377530000000

previous a older 0.02110842065981700000000

previous a old 0.15479508483865800000000

previous a preceding 0.41865034308637100000000

previous a last 0.39125461436137800000000

holler v call 0.41040963415746700000000

holler v cry 0.47167229267402600000000

holler v scream 0.07075084390110380000000

holler v yell 0.02358361463370120000000

holler v shout 0.02358361463370120000000

hinge v add 1.00000000000000000000000

helpful a kind 0.41935483870967800000000

helpful a beneficial 0.03225806451612900000000

helpful a useful 0.03225806451612900000000

helpful a fortunate 0.03225806451612900000000

helpful a valuable 0.03225806451612900000000

helpful a desirable 0.03225806451612900000000

helpful a significant 0.03225806451612900000000

helpful a handy 0.03225806451612900000000

helpful a considerate 0.03225806451612900000000

helpful a obliging 0.06451612903225810000000

helpful a favorable 0.03225806451612900000000

helpful a profitable 0.06451612903225810000000

helpful a important 0.16129032258064500000000

race n lot 0.00788320620804390000000

race n people 0.19407935950498500000000

race n man 0.17672366704855600000000

race n engagement 0.15432565429044600000000

race n progress 0.00779294048810446000000

race n course 0.02210197875337350000000

race n family 0.00704755720436643000000

race n order 0.00219779376521353000000

race n kind 0.00484976343915290000000

race n line 0.01770639122294650000000

race n event 0.07291965742440600000000

race n river 0.00415835113621867000000

race n house 0.32405532837796900000000

race n color 0.00415835113621867000000

latter a recent 0.18127345682209600000000

latter a second 0.01053737870322210000000

latter a last 0.80818916447468200000000

kindly r heartily 0.42920658566191900000000

kindly r tenderly 0.00487074626102542000000

kindly r thoughtfully 0.00108203340923173000000

kindly r well 0.52899090326597700000000

kindly r pray 0.00856249104505374000000

kindly r mildly 0.00150571354836818000000

kindly r graciously 0.02469949339919350000000

kindly r fondly 0.00108203340923173000000

feasible a practicable 0.03012189404594470000000

feasible a reasonable 0.16385372714486600000000

feasible a fit 0.03012189404594470000000

feasible a possible 0.44819503047351200000000

feasible a fitting 0.16385372714486600000000

feasible a likely 0.16385372714486600000000

expostulation n advice 1.00000000000000000000000

yield v retract 0.00123224948637966000000

yield v fetch 0.04057621523007310000000

yield v succumb 0.00111824215200989000000

yield v permit 0.00123224948637966000000

yield v return 0.00173436689519972000000

yield v fall 0.00061612474318983000000

yield v abandon 0.00061612474318983000000

yield v acknowledge 0.00061612474318983000000

yield v afford 0.07100837665262800000000

yield v bestow 0.07100837665262800000000

yield v submit 0.00641272691891456000000

yield v consent 0.00123224948637966000000

yield v bear 0.00246449897275932000000

yield v pay 0.00184837422956949000000

yield v break 0.00579660217572473000000

yield v give 0.19182017004643400000000

yield v allow 0.00677737217508814000000

yield v agree 0.00123224948637966000000

yield v offer 0.00061612474318983000000

yield v accept 0.34900043786464000000000

yield v forsake 0.00061612474318983000000

yield v go 0.18770675334890700000000

yield v leave 0.01816742054879110000000

yield v admit 0.00641272691891456000000

yield v cause 0.02840335066105120000000

yield v buy 0.00173436689519972000000

excitable a impatient 1.00000000000000000000000

essay v seek 1.00000000000000000000000

surround v confine 1.00000000000000000000000

sentence n judgment 0.00072364944440332000000

sentence n time 0.99479016795701000000000

sentence n decision 0.00036182472220165900000

sentence n order 0.00072364944440332000000

sentence n term 0.00195340954317461000000

sentence n doom 0.00072364944440332000000

sentence n verdict 0.00072364944440332000000

proof n mark 0.11842605100578700000000

proof n witness 0.09405179131284330000000

proof n conviction 0.00333795430318053000000

proof n evidence 0.01165907688936550000000

proof n attempt 0.00356239472127708000000

proof n trial 0.00296089644491400000000

proof n impenetrability 0.00178119736063854000000

proof n assurance 0.24901139101726800000000

proof n case 0.23814070814447500000000

proof n examination 0.00178119736063854000000

proof n reason 0.09942763878473330000000

proof n grounds 0.01082658221814210000000

proof n certainty 0.02269414453694030000000

proof n galley 0.00712478944255416000000

proof n trace 0.00691698308381299000000

proof n ground 0.00643440286866325000000

proof n information 0.08596469345067000000000

proof n strength 0.02520071545553420000000

proof n impression 0.01069739159856270000000

dominant a demonstrative 0.50000000000000000000000

dominant a powerful 0.50000000000000000000000

distinguishable a evident 0.96465304939881200000000

distinguishable a perceptible 0.00883673765029692000000

distinguishable a clear 0.01767347530059390000000

distinguishable a noticeable 0.00883673765029692000000

dissimilar a different 1.00000000000000000000000

preserve v keep 0.00408500054824579000000

preserve v spare 0.00271747706722014000000

preserve v support 0.00067936926680503400000

preserve v secure 0.00067936926680503400000

preserve v extend 0.00067936926680503400000

preserve v can 0.03051370142696830000000

preserve v guard 0.00477315476246641000000

preserve v continue 0.00067936926680503400000

preserve v nurse 0.00067936926680503400000

preserve v save 0.95247571206065900000000

preserve v hide 0.00135873853361007000000

preserve v fill 0.00067936926680503400000

deposition n statement 1.00000000000000000000000

damnation n oath 1.00000000000000000000000

resolution n mark 0.00308879817205056000000

resolution n promise 0.05260521109454540000000

resolution n outcome 0.00045661775552487900000

resolution n mind 0.02546055554357650000000

resolution n conclusion 0.00045661775552487900000

resolution n thought 0.00766937585430933000000

resolution n advance 0.01500042976504970000000

resolution n end 0.01045690426555800000000

resolution n idea 0.39132604194996300000000

resolution n decision 0.00091323551104975900000

resolution n proposal 0.00045661775552487900000
[truncated: 2,222,868 more chars]
