# Supplementary material for: Benchmarking Ontologies: Bigger or Better?
Source: PLoS Comput Biol. 2011 Jan 13;7(1):e1001055. doi: 10.1371/journal.pcbi.1001055 (PMC3020923; doi:10.1371/journal.pcbi.1001055)
Supplement: Dataset S2 — Probabilities – medicine. (11.39 MB DOC) [file pcbi.1001055.s002.doc]

word partofspeech synonym Prob

be v survive 0.02521141377032510000000

be v exist 0.01241903635905340000000

be v persist 0.00604723586807026000000

be v signify 0.00015516492250716200000

be v happen 0.00039695202746784100000

be v equal 0.00107317419688907000000

be v accompany 0.00169426256167943000000

be v denote 0.00002955748983955440000

be v remain 0.30650494515440200000000

be v obtain 0.02763822021992810000000

be v act 0.00482118431257299000000

be v arise 0.00182845282325673000000

be v prevail 0.00015040049071838300000

be v comprise 0.01116269988631140000000

be v appear 0.04561084209493850000000

be v live 0.00400733720165116000000

be v endure 0.00033582148232470200000

be v last 0.00603339654917936000000

be v attend 0.00128533598972596000000

be v constitute 0.00906665756441921000000

be v cost 0.00128879076696125000000

be v dwell 0.00001295437362634540000

be v imply 0.00136955204264847000000

be v come 0.00294987119008321000000

be v hold 0.00091386733975890000000

be v do 0.19293630051101700000000

be v look 0.00149255018137866000000

be v respire 0.00000988781022111737000

be v pass 0.00036037378446327400000

be v rest 0.00123244281913160000000

be v continue 0.02522029302332150000000

be v reside 0.00057358711374873200000

be v breathe 0.00041205212519979000000

be v represent 0.07184451432399120000000

be v move 0.00079245097893792500000

be v transpire 0.00000179252699914993000

be v seem 0.03533739321784670000000

be v stay 0.00100938567154020000000

be v follow 0.04578827257389450000000

be v occur 0.09954726520565900000000

be v abide 0.00015326105842731900000

be v occupy 0.00015430011645055500000

be v develop 0.04927567675937340000000

be v stand 0.00185107352005896000000

patient n case 0.47809212089314500000000

patient n inmate 0.00080846601572445500000

patient n inpatient 0.01097032021993630000000

patient n sufferer 0.00039092311602081700000

patient n victim 0.00633158248023225000000

patient n subject 0.48164922328087600000000

patient n outpatient 0.02175736399406500000000

year n span 0.00045001461773385600000

year n life span 0.00037915831628969300000

year n period 0.12175428619448000000000

year n spell 0.00006564203623073470000

year n generation 0.00115209812982475000000

year n decade 0.10036878637110200000000

year n session 0.00269544155611719000000

year n senescence 0.00019541723910733500000

year n space 0.00010592644087498300000

year n duration 0.03646149264910630000000

year n lifetime 0.00550814505320866000000

year n age 0.31133222985732500000000

year n time 0.09287217447446930000000

year n senility 0.00000656864669268430000

year n century 0.00353559575975010000000

year n term 0.00365718315367880000000

year n class 0.00109312034911742000000

year n yr 0.31089793240152600000000

year n stretch 0.00000105773877730628000

year n cycle 0.00746772901458738000000

hour n minute 0.65235206264585100000000

hour n term 0.00967509769871482000000

hour n hr 0.33769666708813100000000

hour n moment 0.00027617256730331700000

neonate n newborn 0.93074335492801800000000

neonate n newborn infant 0.06925664507198250000000

reassessment n review 0.89424449628954200000000

reassessment n reappraisal 0.10575550371045800000000

nicotinic acid n niacin 1.00000000000000000000000

fl n florida 1.00000000000000000000000

virilise v virilize 1.00000000000000000000000

daughter n girl 0.10030770540183800000000

daughter n infant 0.44403440716510500000000

daughter n offspring 0.45565788743305700000000

regime n management 0.01978513798201920000000

regime n method 0.08264262393476770000000

regime n administration 0.10878133216529400000000

regime n policy 0.00001999707175718180000

regime n system 0.00243645233818214000000

regime n plan 0.02537208308948420000000

regime n programme 0.00052667697516538200000

regime n rule 0.00003999414351436360000

regime n regimen 0.68860396201189300000000

regime n scheme 0.03635457120681040000000

regime n course 0.03114006714972980000000

regime n control 0.00307086927928072000000

regime n authority 0.00122623265210261000000

duplicate v repeat 0.12922765435144300000000

duplicate v parallel 0.00071324896451034000000

duplicate v replicate 0.00124288883585807000000

duplicate v reproduce 0.86868861880966600000000

duplicate v redo 0.00012758903852325200000

disclosure n communication 0.85232606493665400000000

disclosure n exposure 0.03706995833555830000000

disclosure n admission 0.11060397672778800000000

extensive a substantial 0.02744884909406990000000

extensive a liberal 0.00022152906893755800000

extensive a unrestricted 0.00011901058286284900000

extensive a wide 0.02900163317473180000000

extensive a diffuse 0.00752197258303505000000

extensive a global 0.00035060696493065700000

extensive a enormous 0.00000548409178128460000

extensive a sustained 0.00700092689623628000000

extensive a lengthy 0.01219858474344200000000

extensive a radical 0.03268905266253770000000

extensive a entire 0.00610419342162230000000

extensive a large 0.06392086296450210000000

extensive a considerable 0.05325627279939660000000

extensive a pervasive 0.00004223399218733550000

extensive a comprehensive 0.16671994884955400000000

extensive a exhaustive 0.02813239809522600000000

extensive a massive 0.07619796882469900000000

extensive a ample 0.00037282944754564300000

extensive a major 0.11991834558082000000000

extensive a widespread 0.05342669575168150000000

extensive a prevalent 0.03831758992368120000000

extensive a extended 0.00363844868626636000000

extensive a sizeable 0.00004728133621489150000

extensive a broad 0.00600876068069965000000

extensive a immense 0.00002975851643228120000

extensive a thorough 0.09489150463020160000000

extensive a wide-ranging 0.00002921816705016050000

extensive a universal 0.00293598662359643000000

extensive a complete 0.08742874412722930000000

extensive a long 0.01355831463767760000000

extensive a nationwide 0.00635878652929622000000

extensive a general 0.02462847675492490000000

extensive a large-scale 0.00449729525834345000000

extensive a all-inclusive 0.00010561545232417400000

extensive a prolonged 0.00489709639392934000000

extensive a vast 0.00081539835678284100000

extensive a exclusive 0.00055615917473883600000

extensive a great 0.02406397102616870000000

extensive a big 0.00000755564245490371000

extensive a worldwide 0.00246861795398513000000

extensive a bulky 0.00006602053820197690000

synovium n synovial membrane 1.00000000000000000000000

unsatisfying a disappointing 1.00000000000000000000000

laxity n tolerance 0.03771212760008650000000

laxity n imprecision 0.00126734299689665000000

laxity n relaxation 0.03755754664076400000000

laxity n weakness 0.86082062582235700000000

laxity n yield 0.00126734299689665000000

laxity n disorder 0.03089582227056170000000

laxity n failure 0.01819963802939870000000

laxity n release 0.00380202899069001000000

laxity n neglect 0.00847752465234910000000

unfruitful a unrewarding 0.25214484679665700000000

unfruitful a poor 0.73370473537604500000000

unfruitful a infertile 0.01415041782729800000000

iron deficiency anemia n iron deficiency anaemia 1.00000000000000000000000

synthesis n structure 0.00094463422918193700000

synthesis n organism 0.00552044775528591000000

synthesis n compound 0.00156784029152484000000

synthesis n combination 0.26536371998373000000000

synthesis n fusion 0.00020349449094294600000

synthesis n mixture 0.00009883778051889740000

synthesis n organization 0.47872078994328100000000

synthesis n incorporation 0.24684040731450400000000

synthesis n consolidation 0.00019767556103779500000

synthesis n unit 0.00054215264999239100000

debate v refute 0.00021040985613214200000

debate v contemplate 0.00009672202333853590000

debate v dispute 0.00042312103646793400000

debate v consider 0.06055775391249390000000

debate v weigh 0.00056388456369025300000

debate v question 0.78461206857843000000000

debate v answer 0.00616135213757026000000

debate v reason 0.00079963822483999800000

debate v prove 0.04777382235728670000000

debate v discuss 0.09532811632645470000000

debate v argue 0.00002330192404793480000

debate v explain 0.00344980905924793000000

wilms' tumor n nephroblastoma 0.89591870415318500000000

wilms' tumor n wilms tumour 0.10408129584681500000000

unify v combine 0.94516956754539100000000

unify v mix 0.05483043245460930000000

avoid v hide 0.00129294960742076000000

avoid v sidestep 0.00009347918400650460000

avoid v circumvent 0.07418567419922390000000

avoid v retreat 0.00037300223174152800000

avoid v divert 0.00004826625458238330000

avoid v elude 0.00025181670063153700000

avoid v evade 0.00208648123380416000000

avoid v prevent 0.79725068464160100000000

avoid v withdraw 0.00546746468063742000000

avoid v nullify 0.00013692533096839900000

avoid v preclude 0.01075522024670370000000

avoid v quit 0.00033981281318955800000

avoid v obviate 0.03466962533682140000000

avoid v bypass 0.00476326647443076000000

avoid v avert 0.01227039994676170000000

avoid v abandon 0.05601493111747530000000

platform n position 0.76841734226295400000000

platform n policy 0.07719421924568200000000

platform n program 0.15438843849136400000000

handgrip n grip 1.00000000000000000000000

colour n color 1.00000000000000000000000

suppress v conceal 0.00001037464131860520000

suppress v hamper 0.00473358815434817000000

suppress v impede 0.00054226317641124600000

suppress v delete 0.00065454477123315100000

suppress v hide 0.00001766035772096660000

suppress v hinder 0.00019991960195251100000

suppress v cut 0.00001037464131860520000

suppress v obliterate 0.00005651504490248100000

suppress v screen 0.00098378477471185400000

suppress v remove 0.01634391838069920000000

suppress v halt 0.00204963417237361000000

suppress v spike 0.00002851898471000280000

suppress v eliminate 0.06302847015562700000000

suppress v block 0.05501067999004390000000

suppress v restrain 0.01276454625958020000000

suppress v prohibit 0.00001112859009378820000

suppress v silence 0.00010835984923218700000

suppress v discharge 0.00250604940257798000000

suppress v stop 0.00101256927348120000000

suppress v kill 0.00200271106713288000000

suppress v secrete 0.00822187058642981000000

suppress v repress 0.00018020654527066700000

suppress v override 0.00000714799119188724000

suppress v overwhelm 0.00002732749848528630000

suppress v annihilate 0.00036969830914510900000

suppress v invalidate 0.00000661971869267118000

suppress v abolish 0.04641327557194500000000

suppress v restrict 0.00020375967639750300000

suppress v prevent 0.22838486838404800000000

suppress v curb 0.00002065431676371140000

suppress v confine 0.00628487125546686000000

suppress v excise 0.00712902634143081000000

suppress v extirpate 0.00002377515657805980000

suppress v nullify 0.00043208489881335000000

suppress v overcome 0.00876815021584015000000

suppress v contain 0.00035258302534897500000

suppress v subvert 0.00000357399559594361000

suppress v eradicate 0.02572465643502130000000

suppress v inhibit 0.46244001715190900000000

suppress v obviate 0.00002602912220058750000

suppress v withhold 0.00019633819733106900000

suppress v check 0.00007161210158494790000

suppress v erase 0.00000556429504689408000

suppress v mask 0.00841384041330867000000

suppress v arrest 0.00676195005446339000000

suppress v limit 0.02745488745222090000000

brushing n brush 1.00000000000000000000000

smear v stain 0.50000000000000000000000

smear v apply 0.50000000000000000000000

valve n lid 0.00111493178579806000000

valve n flap 0.99888506821420200000000

erroneous a untrue 0.00003538415174341060000

erroneous a spurious 0.00086827602537961100000

erroneous a wrong 0.00554862007931925000000

erroneous a mistaken 0.00230439288228962000000

erroneous a defective 0.00030407285143803200000

erroneous a unfounded 0.00046660909590242100000

erroneous a false 0.24885034042331400000000

erroneous a incorrect 0.73054881718215100000000

erroneous a inaccurate 0.01107348730846310000000

convincing a plausible 0.00087481701136588800000

convincing a impressive 0.00339310845935877000000

convincing a compelling 0.25606671131196500000000

convincing a reliable 0.07511334768330860000000

convincing a credible 0.00198220217476217000000

convincing a acceptable 0.00196579617611821000000

convincing a satisfactory 0.05874634150268690000000

convincing a probable 0.00154117406463605000000

convincing a dependable 0.00003088030762663310000

convincing a solid 0.01108564919959580000000

convincing a possible 0.02674687584424700000000

convincing a valid 0.00074112738303921400000

convincing a reasonable 0.00435300976722511000000

convincing a powerful 0.00554282459979791000000

convincing a persuasive 0.00637485598548252000000

convincing a cogent 0.00038860541770077600000

convincing a sound 0.00399528465715097000000

convincing a trustworthy 0.00003088030762663310000

convincing a strong 0.29463398250728300000000

convincing a conclusive 0.24639252563902300000000

conceal v suppress 0.31578947368421200000000

conceal v hide 0.02631578947368360000000

conceal v screen 0.28947368421052800000000

conceal v protect 0.21052631578947400000000

conceal v wrap 0.02631578947368360000000

conceal v secrete 0.02631578947368360000000

conceal v cover 0.05263157894736800000000

conceal v mask 0.02631578947368360000000

conceal v obscure 0.02631578947368360000000

intensive care unit n icu 1.00000000000000000000000

boost n attention 0.00151953449726730000000

boost n improvement 0.68268540777227300000000

boost n addition 0.31123645423865800000000

boost n growth 0.00303906899453460000000

boost n recommendation 0.00151953449726730000000

appreciation n recognition 0.17876433305205100000000

appreciation n understanding 0.09212983463761570000000

appreciation n rise 0.00001025708333676220000

appreciation n rating 0.00177403571909637000000

appreciation n evaluation 0.04858854360295240000000

appreciation n judgement 0.00001025708333676220000

appreciation n sensitivity 0.03650252428803110000000

appreciation n perception 0.00041807243987086800000

appreciation n assessment 0.06874996447920300000000

appreciation n awareness 0.16607421086940300000000

appreciation n realization 0.00952814661429634000000

appreciation n review 0.07475936289686120000000

appreciation n detection 0.00763277437477383000000

appreciation n opinion 0.00002051416667352440000

appreciation n growth 0.00002051416667352440000

appreciation n analysis 0.05022443482585720000000

appreciation n appraisal 0.14292219921444500000000

appreciation n knowledge 0.12187002048552200000000

neglect v scant 0.00010893183901706900000

neglect v discount 0.02514962926798240000000

neglect v postpone 0.00013603180887226500000

neglect v forget 0.33030790391001400000000

neglect v discard 0.00059885913479574200000

neglect v dismiss 0.00853748553130197000000

neglect v miss 0.03427485135347300000000

neglect v disregard 0.00231809411874249000000

neglect v drop 0.00010893183901706900000

neglect v underestimate 0.06009636566542890000000

neglect v fail 0.00221517941004360000000

neglect v overlook 0.41970705205249900000000

neglect v reject 0.00076199091352321000000

neglect v bypass 0.00054412723548907200000

neglect v defer 0.00035389548690640400000

neglect v ignore 0.11221644542052500000000

neglect v omit 0.00063214775870535500000

neglect v abandon 0.00193207725366323000000

mitigate v diminish 0.00354433297673971000000

mitigate v relieve 0.00102667165676926000000

mitigate v moderate 0.00074549483160059600000

mitigate v tame 0.00102667165676926000000

mitigate v curb 0.00102667165676926000000

mitigate v blunt 0.00102667165676926000000

mitigate v alleviate 0.90372772587114300000000

mitigate v check 0.00102667165676926000000

mitigate v reduce 0.06810075149620280000000

mitigate v modify 0.00662434794704750000000

mitigate v decrease 0.01212398859342030000000

incorrectly r wrongly 0.17566539923954400000000

incorrectly r inaccurately 0.00456273764258555000000

incorrectly r falsely 0.81977186311787100000000

body mass index n bmi 1.00000000000000000000000

inadequately r insufficiently 0.36115625655926300000000

inadequately r ineffectively 0.00012792591481205900000

inadequately r incompletely 0.63871581752592500000000

foam n spray 0.45735821818544100000000

foam n yeast 0.54264178181455900000000

specially r specifically 1.00000000000000000000000

ordinary a usual 0.19817168695819300000000

ordinary a traditional 0.01717524521927150000000

ordinary a customary 0.01165715805636430000000

ordinary a plain 0.05569531071374050000000

ordinary a current 0.01758799285697070000000

ordinary a conventional 0.27834021253880300000000

ordinary a standard 0.27614171343168200000000

ordinary a poor 0.00870834061911697000000

ordinary a low 0.00331958805578585000000

ordinary a formal 0.00795647295910578000000

ordinary a routine 0.12524627859096700000000

probability n outlook 0.00045091319902415500000

probability n possibility 0.08282214501149250000000

probability n likelihood 0.70540985222272700000000

probability n anticipation 0.00059261957859182600000

probability n feasibility 0.00016119860007207900000

probability n contingency 0.00005636414987801940000

probability n chance 0.08657065732587990000000

probability n odds 0.09588586343498820000000

probability n promise 0.00003307857444578740000

probability n expectation 0.00106502788188563000000

probability n hazard 0.00933239987886600000000

probability n prospect 0.01350432801749270000000

probability n appearance 0.00411555212465683000000

categorical a specific 0.00457724840725628000000

categorical a absolute 0.00058825758765180700000

categorical a definitive 0.00127941341734748000000

categorical a total 0.04908522647486630000000

categorical a positive 0.00413363273765827000000

categorical a definite 0.00058825758765180700000

categorical a actual 0.00025222519907709200000

categorical a unconditional 0.00094198390675730300000

categorical a categoric 0.74376788162945500000000

categorical a demonstrable 0.00025222519907709200000

categorical a accurate 0.19325423443585500000000

categorical a conclusive 0.00058825758765180700000

categorical a unequivocal 0.00069115582969567800000

blot n smear 0.25000000000000000000000

blot n defect 0.75000000000000000000000

anxious a careful 0.79664097870831700000000

anxious a agitated 0.15927141448872600000000

anxious a dying 0.04408760680295630000000

reconstructive surgery n plastic surgery 1.00000000000000000000000

lesion n mark 0.00014311236925272000000

lesion n papule 0.00088804515749910900000

lesion n rupture 0.00438131979680044000000

lesion n injury 0.13315400663008500000000

lesion n wound 0.00668476295887227000000

lesion n abrasion 0.00006628415631307400000

lesion n blister 0.00053423097544183100000

lesion n bulla 0.00004847420633120510000

lesion n abscess 0.01609390402819260000000

lesion n papilla 0.00010947761249025100000

lesion n ulcer 0.01943780817306520000000

lesion n burn 0.00323971832896914000000

lesion n mouse 0.00574799952760688000000

lesion n tear 0.02203602841559980000000

lesion n scald 0.00029834855226858400000

lesion n incision 0.00349677486308874000000

lesion n bruise 0.00003638375504244290000

lesion n chancroid 0.00001829230528762260000

lesion n pustule 0.00003953060160127090000

lesion n scratch 0.00003748097390309050000

lesion n scrape 0.00046409774797336300000

lesion n abnormality 0.10843314740225000000000

lesion n disorder 0.06547698437528060000000

lesion n swelling 0.00393604837288029000000

lesion n ulceration 0.01035851908645940000000

lesion n puncture 0.00180950631583980000000

lesion n stab 0.00000216697089096891000

lesion n rip 0.00000722626667472734000

lesion n laceration 0.00143570870542883000000

lesion n trauma 0.01314235326328760000000

lesion n fracture 0.01759287398631440000000

lesion n rent 0.00002290264480721800000

lesion n growth 0.00800537733172809000000

lesion n hurt 0.00000196402540333056000

lesion n chancre 0.00015494132289206100000

lesion n derangement 0.00000513656044077935000

lesion n impairment 0.00603616621522820000000

lesion n tumor 0.44658714479415600000000

lesion n spot 0.00017317860167261900000

lesion n vesicle 0.00125984237073598000000

lesion n inflammation 0.01079345201109730000000

lesion n harm 0.00023862199682485200000

lesion n contusion 0.00112425040402641000000

lesion n cut 0.00002514312887927780000

lesion n bleb 0.00000999181344953589000

lesion n neoplasm 0.08607822628425510000000

lesion n break 0.00033304461341114100000

open a extensive 0.03889342639616160000000

open a apparent 0.00012845030438054400000

open a straightforward 0.00271081769491952000000

open a liberal 0.00042734227993158000000

open a unproven 0.00372737433051434000000

open a wide 0.02723111314527550000000

open a doubtful 0.00000240751894464109000

open a obtainable 0.00060722190807248900000

open a objective 0.00501604007284056000000

open a unsafe 0.00000240751894464109000

open a evident 0.00712978629706040000000

open a unbiased 0.00000789450164134664000

open a clear 0.00065192454168227100000

open a controversial 0.10940959895771800000000

open a debatable 0.00075968111436278500000

open a susceptible 0.00000240751894464109000

open a unscreened 0.00745474866102868000000

open a unaffected 0.00052399248586640900000

open a uncertain 0.01718926836842930000000

open a practicable 0.00007893263288148010000

open a extended 0.02150390316176600000000

open a capable 0.00015612039080688300000

open a sensitive 0.01357781039560120000000

open a liable 0.00003485337036325090000

open a broad 0.00000240751894464109000

open a free 0.00609933981356891000000

open a available 0.04468712579859870000000

open a obvious 0.00177348911077288000000

open a prone 0.00031110879930486400000

open a palpable 0.00052490762264711300000

open a undetermined 0.00078496315357832900000

open a patent 0.15439133456743300000000

open a subject 0.00413910852385501000000

open a equivocal 0.00171132453125689000000

open a mild 0.00192089172499442000000

open a vulnerable 0.00372978184945898000000

open a unsettled 0.00079872307082450100000

open a undecided 0.00000240751894464109000

open a general 0.05872163091358500000000

open a experimental 0.11808211997409400000000

open a direct 0.07938637925169250000000

open a moderate 0.00019960472369161900000

open a problematic 0.00995334841905208000000

open a amenable 0.00447284919661720000000

open a perforated 0.00044432276787588100000

open a questionable 0.01197864074402370000000

open a simple 0.16398719506429200000000

open a admissible 0.00000789450164134664000

open a indeterminate 0.00001776054822364750000

open a unresolved 0.07464140920394620000000

open a genuine 0.00000240751894464109000

hyperalimentation n tpn 0.05357452916745750000000

hyperalimentation n total parenteral nutrition 0.94642547083254200000000

cannabis n marihuana 0.95538226206686200000000

cannabis n marijuana 0.04461773793313780000000

cage n crib 0.01287440553825970000000

cage n cell 0.98712559446174000000000

worthwhile a excellent 0.02567437008798550000000

worthwhile a productive 0.00019413628480279500000

worthwhile a invaluable 0.00389379959478831000000

worthwhile a effective 0.17625686765904100000000

worthwhile a helpful 0.04427690878719960000000

worthwhile a good 0.08038630452362230000000

worthwhile a positive 0.02793467638101930000000

worthwhile a valuable 0.04785729291729020000000

worthwhile a justifiable 0.00316314888796385000000

worthwhile a important 0.19593526954159900000000

worthwhile a rewarding 0.00032938924744786600000

worthwhile a fruitful 0.00008759080122464740000

worthwhile a useful 0.28066333024554300000000

worthwhile a profitable 0.01479987430371230000000

worthwhile a estimable 0.00001178035007539860000

worthwhile a advantageous 0.03806461978952750000000

worthwhile a beneficial 0.06047064059715770000000

radiograph n negative 0.01649037113475850000000

radiograph n x-ray 0.77076668166705700000000

radiograph n picture 0.00039108062430281100000

radiograph n radiogram 0.01994818526699400000000

radiograph n roentgenogram 0.19240368130688700000000

snag n block 0.11111111111111100000000

snag n delay 0.11111111111111100000000

snag n projection 0.11111111111111100000000

snag n complication 0.66666666666666700000000

maternity n pregnancy 1.00000000000000000000000

sketch v describe 0.57575757575757600000000

sketch v summarize 0.18181818181818200000000

sketch v outline 0.24242424242424200000000

hemophiliac n haemophiliac 0.97577361363026800000000

hemophiliac n bleeder 0.02422638636973220000000

gp n general practitioner 1.00000000000000000000000

diphenylhydantoin n phenytoin 1.00000000000000000000000

sentiment n view 1.00000000000000000000000

town n city 0.86353225199869000000000

town n center 0.06459377160950640000000

town n village 0.07187397639180390000000

origin n generator 0.00076931457322032300000

origin n issue 0.00169894581789005000000

origin n causation 0.00300610781212103000000

origin n etiology 0.34678916062974900000000

origin n rise 0.00000867664750432446000

origin n stock 0.00012186923562652800000

origin n ancestry 0.01930607515842920000000

origin n determinant 0.00109473974271750000000

origin n commencement 0.00001279194813668520000

origin n causality 0.00000375499453461913000

origin n entry 0.00812538624223774000000

origin n race 0.00370513024734953000000

origin n outset 0.00003345431597666540000

origin n antecedent 0.00089192209810184400000

origin n extraction 0.00066802395824911800000

origin n introduction 0.00100238310489709000000

origin n blood 0.00877466526946608000000

origin n cause 0.35092532222459400000000

origin n agent 0.00780945323662263000000

origin n element 0.00352247284915760000000

origin n outbreak 0.00030306507824521100000

origin n portal 0.00201428370595626000000

origin n infancy 0.00214030157009097000000

origin n beginning 0.00003329104639011040000

origin n principle 0.00173512362066839000000

origin n occasion 0.00024474355464005900000

origin n author 0.00122341398603068000000

origin n progenitor 0.00174014443853229000000

origin n root 0.00029327881093863700000

origin n heritage 0.00207124746831430000000

origin n nucleus 0.00003164260090618700000

origin n egg 0.00020731778014627800000

origin n onset 0.00282081553021382000000

origin n birthplace 0.00000681657100254202000

origin n ancestor 0.00644165959740221000000

origin n youth 0.00000127229195307207000

origin n sperm 0.00000248259135924106000

origin n activation 0.01612792834739670000000

origin n reservoir 0.00357379344329173000000

origin n lineage 0.05068035288438560000000

origin n invention 0.00016935818659836800000

origin n family 0.00529728236662670000000

origin n basis 0.01522161973903030000000

origin n induction 0.00862779720546383000000

origin n heart 0.00490174700334516000000

origin n alpha 0.00001019883905723840000

origin n ground 0.00240488624969682000000

origin n head 0.00004292053628465440000

origin n childhood 0.01365583549425190000000

origin n pedigree 0.00000043147808412817800

origin n influence 0.00436201794431339000000

origin n house 0.00000086295616825635700

origin n source 0.03399477571202130000000

origin n descent 0.03690123204796450000000

origin n parent 0.00005682531351041630000

origin n genesis 0.00004537521225843060000

origin n inception 0.00009249562498833940000

origin n birth 0.00186675612139896000000

origin n line 0.01005743232343570000000

origin n derivation 0.01232752462105550000000

mole n barrier 0.00180831826401447000000

mole n nevus 0.99819168173598600000000

hamper v impede 0.00354142045732357000000

hamper v hinder 0.01267455742621070000000

hamper v diminish 0.04585990693055150000000

hamper v block 0.04367751897365740000000

hamper v restrain 0.00083327540172319400000

hamper v handicap 0.01267455742621070000000

hamper v frustrate 0.14448995465880200000000

hamper v restrict 0.12040829554900100000000

hamper v reduce 0.00499965241033916000000

hamper v limit 0.60917430996273400000000

hamper v decrease 0.00166655080344639000000

new jersey n nj 1.00000000000000000000000

diarrheal a diarrhoeal 1.00000000000000000000000

cook v create 0.01018964043040760000000

cook v prepare 0.02037928086081530000000

cook v plan 0.00679309362027177000000

cook v make 0.27851683843114300000000

cook v combine 0.02909540169135510000000

cook v mix 0.04755165534190250000000

cook v reduce 0.60747408962410500000000

fever n fire 0.00006477033014423010000

fever n intensity 0.01522859548898400000000

fever n typhoid fever 0.00252307920080825000000

fever n temperature 0.29448713521360300000000

fever n redness 0.00068456760928112800000

fever n enthusiasm 0.00002252324479132830000

fever n pyrexia 0.21951285275645100000000

fever n malaria 0.02058183305041950000000

fever n hyperpyrexia 0.04834745644986880000000

fever n excitement 0.00015441374645439000000

fever n disease 0.29232710809434800000000

fever n delirium 0.04424770349898590000000

fever n confusion 0.01186768911177800000000

fever n hyperthermia 0.01002029066494640000000

fever n mania 0.00475789871788365000000

fever n inflammation 0.03279514991946230000000

fever n agitation 0.00061066159605342300000

fever n panic 0.00137918737570072000000

fever n flush 0.00038708393003581500000

pound v strike 1.00000000000000000000000

institution n origin 0.00213853385484946000000

institution n trust 0.00004137690016865900000

institution n university 0.02516873929625330000000

institution n innovation 0.00011139479864389000000

institution n prison 0.00081090255445444200000

institution n hospital 0.47931334651158500000000

institution n initiation 0.05968387585547780000000

institution n custom 0.00001767993321337210000

institution n commencement 0.00006955786139462330000

institution n concern 0.00220460770121703000000

institution n firm 0.00008319403731206420000

institution n school 0.00377306183388084000000

institution n installation 0.00000310342959455975000

institution n coalition 0.00010853266975701900000

institution n introduction 0.00444554541893692000000

institution n system 0.03728895251351690000000

institution n psychiatric hospital 0.00066676326504579800000

institution n association 0.01420034073435540000000

institution n society 0.00608704593958289000000

institution n centre 0.08586456334554480000000

institution n purpose 0.05075795595393280000000

institution n start 0.00518620871906007000000

institution n beginning 0.01215528938849730000000

institution n principle 0.00415749655174497000000

institution n confirmation 0.00151703475646663000000

institution n authorization 0.00057062625023448800000

institution n rationale 0.00298512336356152000000

institution n institute 0.03745729634558850000000

institution n rule 0.00054262301908634600000

institution n organization 0.00898244193024088000000

institution n motive 0.00000045135166639359200

institution n college 0.00018228338549157300000

institution n asylum 0.00001354427632135530000

institution n practice 0.04739943971155950000000

institution n code 0.00044775394686929600000

institution n union 0.00005823854512753890000

institution n establishment 0.00440575503423971000000

institution n library 0.00001214660227246020000

institution n foundation 0.00014657296186787700000

institution n erection 0.00001584821799701460000

institution n law 0.00262533210274504000000

institution n clinic 0.05945426567786780000000

institution n formation 0.00489020638188235000000

institution n appointment 0.00004983944478317610000

institution n construction 0.00024304451398876400000

institution n creation 0.00082027523471207700000

institution n company 0.00015239712328957500000

institution n source 0.00706986390157323000000

institution n inception 0.00012265797902236700000

institution n order 0.02544141231278010000000

institution n body 0.00005545656074434210000

family medicine n family practice 1.00000000000000000000000

dm n diabetes mellitus 1.00000000000000000000000

repeal v withdraw 1.00000000000000000000000

delusion n chimera 0.00012755888748743400000

delusion n error 0.01035283527991430000000

delusion n dream 0.00034709300337815300000

delusion n illusion 0.87228799679798300000000

delusion n vision 0.00012755888748743400000

delusion n deception 0.02038269578713350000000

delusion n phantom 0.07648833711285960000000

delusion n hallucination 0.01941115181476520000000

delusion n bubble 0.00047477242899194000000

proteinase n protease 1.00000000000000000000000

proposal n diagram 0.00072298182794795800000

proposal n offer 0.00123759701634787000000

proposal n design 0.17051167007436900000000

proposal n measure 0.07035112392991300000000

proposal n policy 0.00896761492837233000000

proposal n pattern 0.00881359937886549000000

proposal n proposition 0.00037724131511418400000

proposal n plan 0.01183280258408160000000

proposal n project 0.00173869865103696000000

proposal n program 0.12754796325669700000000

proposal n scheme 0.03713160516358360000000

proposal n suggestion 0.09053282522778910000000

proposal n presentation 0.00645694880929248000000

proposal n application 0.05226154474636040000000

proposal n idea 0.05887414849026340000000

proposal n arrangement 0.00123759701634787000000

proposal n recommendation 0.35140403758361800000000

neighborhood n area 0.61704975629903900000000

neighborhood n neighbourhood 0.11508469409080400000000

neighborhood n district 0.01825598141821470000000

neighborhood n region 0.05939258055074900000000

neighborhood n community 0.19021698764119200000000

genetic a familial 0.36561167668673200000000

genetic a hereditary 0.28085684405524100000000

genetic a congenital 0.35353147925802700000000

fire n strength 0.01708734574112070000000

fire n pep 0.46658298055879100000000

fire n attack 0.00839391064075834000000

fire n report 0.15467047655160600000000

fire n force 0.01528367089838800000000

fire n life 0.00180367484273270000000

fire n stress 0.00159552031843098000000

fire n trial 0.15912982247832100000000

fire n discharge 0.17545259796985100000000

equivalence n match 0.00115238783836337000000

equivalence n agreement 0.02132873813527900000000

equivalence n coincidence 0.00015535319920160800000

equivalence n comparison 0.69185511051783200000000

equivalence n twin 0.00007767659960080300000

equivalence n correlation 0.28432309397357600000000

equivalence n equation 0.00015535319920160800000

equivalence n similarity 0.00036921324538230400000

equivalence n parity 0.00058307329156300700000

edta n ethylenediaminetetraacetic acid 1.00000000000000000000000

tabulate v chart 0.00082682202840441700000

tabulate v place 0.00126856115655557000000

tabulate v enumerate 0.00110940220983011000000

tabulate v grade 0.00118503533309785000000

tabulate v rate 0.12266229621503900000000

tabulate v count 0.00336198793641693000000

tabulate v categorize 0.00287127004360416000000

tabulate v analyze 0.81475397115287900000000

tabulate v register 0.03452414368168640000000

tabulate v graph 0.00005486405777236340000

tabulate v catalogue 0.00037638199820728300000

tabulate v list 0.00200766846890500000000

tabulate v order 0.00065100513541295500000

tabulate v catalog 0.00052067976173604600000

tabulate v range 0.00406703236105430000000

tabulate v formulate 0.00156402388137764000000

tabulate v rank 0.00054150026522904000000

tabulate v group 0.00216599050933493000000

tabulate v classify 0.00548736380345691000000

ossification n calcification 1.00000000000000000000000

cyclical a cyclic 1.00000000000000000000000

capacity n position 0.00002692312163975450000

capacity n function 0.13821261333076100000000

capacity n spread 0.00002233209349369920000

capacity n understanding 0.00008885574745096120000

capacity n accommodation 0.00605467639714521000000

capacity n reason 0.00197285624508089000000

capacity n strength 0.00305165416803905000000

capacity n tolerance 0.00065851254495117500000

capacity n capacitance 0.00242187055885808000000

capacity n magnitude 0.00046574433824193900000

capacity n amplitude 0.00004863193893289320000

capacity n power 0.07794260448061580000000

capacity n role 0.00863195036438384000000

capacity n range 0.00002867867390638620000

capacity n measure 0.00822649819561387000000

capacity n skill 0.00060546763971452100000

capacity n office 0.00009845002271780830000

capacity n retention 0.00006339975284968800000

capacity n yield 0.00242904029097442000000

capacity n volume 0.03621002861652820000000

capacity n mass 0.00525349740565041000000

capacity n brain 0.00518310341008697000000

capacity n size 0.00016153268143991000000

capacity n room 0.00000086905072443594300

capacity n capability 0.01739979847898800000000

capacity n production 0.00074835461071157500000

capacity n adequacy 0.00004730215935269690000

capacity n performance 0.35846582100577100000000

capacity n judgment 0.00072231227194013000000

capacity n ability 0.27179175718906900000000

capacity n endurance 0.01897123580730910000000

capacity n competence 0.00040364509314301400000

capacity n resistance 0.00554596711710679000000

capacity n extent 0.00172137876382468000000

capacity n responsibility 0.00000893679173010362000

capacity n head 0.00496967770936289000000

capacity n content 0.00563841739484148000000

capacity n mind 0.00000086905072443594300

capacity n intelligence 0.00000086905072443594300

capacity n potential 0.01567782481668710000000

capacity n duty 0.00002604161891245250000

medulla n medulla oblongata 1.00000000000000000000000

membrane n tissue 0.93981607054818800000000

membrane n film 0.00042169102236333100000

membrane n layer 0.01845316348651250000000

membrane n skin 0.03845901950165860000000

membrane n sheath 0.00285005544127778000000

unidentified a unspecified 0.00042249370784278600000

unidentified a enigmatic 0.01101576676257860000000

unidentified a unknown 0.01130983156379150000000

unidentified a unexplained 0.31794124786420500000000

unidentified a silent 0.01260238374251050000000

unidentified a obscure 0.20847597956874600000000

unidentified a undefined 0.43823229679032500000000

spiral n coil 1.00000000000000000000000

refusal n option 0.53156334030289600000000

refusal n ban 0.00088209671256905900000

refusal n rejection 0.29774658615229900000000

refusal n reversal 0.01236913932279080000000

refusal n exclusion 0.05663161696358090000000

refusal n noncompliance 0.10080722054586500000000

precaution v advise 1.00000000000000000000000

cue n signal 0.37033779827695400000000

cue n stimulus 0.62950925450178600000000

cue n sign 0.00007647361062983140000

cue n part 0.00003823680531491540000

cue n idea 0.00003823680531491540000

citation n reference 0.53985198469173600000000

citation n record 0.02201274197007900000000

citation n illustration 0.00075980647621382100000

citation n evidence 0.01424122353778250000000

citation n schedule 0.00010878839721030200000

citation n calculation 0.00126835257704576000000

citation n documentation 0.00177283066183484000000

citation n abstract 0.41998427168809800000000

vitamin b6 n pyridoxine 1.00000000000000000000000

port wine n port 1.00000000000000000000000

tubing n tube 1.00000000000000000000000

scarce a limited 0.26851733871180100000000

scarce a unusual 0.01839235802683890000000

scarce a atypical 0.00174814837410925000000

scarce a deficient 0.08363420885444290000000

scarce a noteworthy 0.00160459095649992000000

scarce a scant 0.02968388695451370000000

scarce a exceptional 0.00062173094926757500000

scarce a unique 0.00274240438586503000000

scarce a uncommon 0.06538865463726990000000

scarce a inadequate 0.01595208602106010000000

scarce a scanty 0.03290334702787270000000

scarce a meagre 0.00056054871156484200000

scarce a singular 0.00003419997398671620000

scarce a insufficient 0.04131038994833400000000

scarce a sporadic 0.00285384378957869000000

scarce a infrequent 0.03278553237008840000000

scarce a meager 0.00094210787087727300000

scarce a short 0.00839787267775973000000

scarce a distinctive 0.00327719013968726000000

scarce a low 0.15857796826747300000000

scarce a lean 0.00145965405949705000000

scarce a occasional 0.00383428344719253000000

scarce a rare 0.15846033472958600000000

scarce a notable 0.00008803245074816360000

scarce a tight 0.00002399937890355320000

scarce a sparse 0.06620528728518150000000

purification n clarification 0.50000000000000000000000

purification n lavage 0.50000000000000000000000

plummet v decrease 1.00000000000000000000000

mri n magnetic resonance imaging 1.00000000000000000000000

realistic a prudent 0.00643631824803371000000

realistic a achievable 0.00030179625871902000000

realistic a true 0.00048578141399363500000

realistic a attainable 0.00121190103557573000000

realistic a feasible 0.05508534730194840000000

realistic a practical 0.06814313919317780000000

realistic a constant 0.00070823510538302700000

realistic a sensible 0.00028797453690452200000

realistic a practicable 0.00005528688725799210000

realistic a precise 0.40360246989442000000000

realistic a reasonable 0.09953630047699920000000

realistic a representative 0.00074823176126139800000

realistic a exact 0.00029392526358613500000

realistic a sound 0.00001382172181449770000

realistic a accurate 0.36282318908096800000000

realistic a truthful 0.00026628181995713900000

patent n protection 0.00024591412073231700000

patent n work 0.00169541183432476000000

patent n limitation 0.00024591412073231700000

patent n procedure 0.06732826263308690000000

patent n method 0.06303418106843790000000

patent n process 0.00098365648292926900000

patent n control 0.86646665973975700000000

nation n people 0.01171648102548220000000

nation n country 0.70553691586065400000000

nation n population 0.11266760477523800000000

nation n society 0.08417899709732180000000

nation n family 0.00102565935992674000000

nation n state 0.04075332399156060000000

nation n community 0.04412101788981710000000

peruse v examine 0.38618677042801600000000

peruse v scrutinize 0.00097276264591438400000

peruse v scan 0.00486381322957198000000

peruse v analyze 0.17898832684824900000000

peruse v study 0.42898832684824900000000

survive v exist 0.00586154408029741000000

survive v succeed 0.00310288641194980000000

survive v persist 0.06749584614073860000000

survive v bear 0.02416148847525120000000

survive v recover 0.13499344579399900000000

survive v remain 0.33380710665981600000000

survive v suffer 0.02845282924918040000000

survive v sustain 0.01045648788218880000000

survive v live 0.26218035490447800000000

survive v endure 0.00063538837520476900000

survive v last 0.05496731013247190000000

survive v keep 0.00005550664664611040000

survive v go 0.01074605479070860000000

survive v continue 0.06308375045706930000000

dictate v propose 0.14050581578727200000000

dictate v direct 0.15593519275456500000000

dictate v decide 0.01158651587030830000000

dictate v order 0.62374077101826200000000

dictate v suggest 0.06823170456959310000000

dead r immediately 0.00161854881894610000000

dead r directly 0.00044118079465525800000

dead r exactly 0.00028923400022791600000

dead r late 0.01215803311626200000000

dead r suddenly 0.00091387450321446600000

dead r still 0.98457912876669400000000

bay n cell 1.00000000000000000000000

pituitary gland n pituitary 1.00000000000000000000000

adolescent n girl 0.25311671670245800000000

adolescent n young man 0.00756542039711700000000

adolescent n slip 0.00008391521522197540000

adolescent n minor 0.00124496088833227000000

adolescent n youngster 0.00141600472876617000000

adolescent n juvenile 0.00195928270950654000000

adolescent n schoolgirl 0.00230025762860431000000

adolescent n youth 0.50956099678567900000000

adolescent n teen-ager 0.00019673456013151900000

adolescent n schoolboy 0.00032699383113515400000

adolescent n d 0.07103743957390500000000

adolescent n teenager 0.15119127697914300000000

insecticide n pesticide 1.00000000000000000000000

clomiphene citrate n clomiphene 1.00000000000000000000000

manage v survive 0.00099044759392236900000

manage v dictate 0.00001121812301489880000

manage v guide 0.00193711842302524000000

manage v cope 0.00336558536765148000000

manage v use 0.05555427374772510000000

manage v succeed 0.00000267492524273756000

manage v function 0.00205189768588384000000

manage v handle 0.00204378100333506000000

manage v arrange 0.00007522779111521340000

manage v undertake 0.00348296285202374000000

manage v watch 0.00236938370891924000000

manage v operate 0.00565908565008556000000

manage v regulate 0.00011848257796625100000

manage v cause 0.00002939766280889020000

manage v direct 0.00151924189939704000000

manage v upstage 0.00001618712493916870000

manage v execute 0.00000213899289884069000

manage v train 0.00024174196746375800000

manage v pilot 0.00003759369396612930000

manage v work 0.00102782004007330000000

manage v contend 0.00000052008809154762300

manage v lead 0.00000498775697328731000

manage v administer 0.01015517678137410000000

manage v care 0.00970493539672304000000

manage v master 0.00000257567083722796000

manage v achieve 0.02026277700438700000000

manage v distribute 0.00057434434397666800000

manage v show 0.00123054810331628000000

manage v accomplish 0.01452360850119690000000

manage v treat 0.71342183787297500000000

manage v do 0.00066741846994019100000

manage v advocate 0.00007728481350660840000

manage v fix 0.00181279683700325000000

manage v conduct 0.01043728214911060000000

manage v run 0.00025124406433415100000

manage v order 0.00091763278606310300000

manage v advise 0.00135476602574194000000

manage v teach 0.00037199175882971200000

manage v maintain 0.00516298059555270000000

manage v control 0.03561353079141970000000

manage v deal 0.00169971419387479000000

manage v influence 0.00049902972685683900000

manage v supervise 0.00011946127515375400000

manage v perform 0.08745252821270630000000

manage v counsel 0.00111830703477030000000

manage v effect 0.00000862509258450584000

manage v instruct 0.00188096483634206000000

manage v engineer 0.00000311065588577547000

manage v manipulate 0.00013375832901520700000

polarization n polarisation 1.00000000000000000000000

itching n itch 1.00000000000000000000000

cramp n pain 0.99664733118050100000000

cramp n obstruction 0.00021301072589207300000

cramp n restraint 0.00313965809360640000000

adapted a correct 1.00000000000000000000000

early a primary 0.07290143059193150000000

early a preliminary 0.06116080222251060000000

early a new 0.03209930308951750000000

early a fast 0.01058516511084840000000

early a unanticipated 0.00004381213790732700000

early a original 0.00325049269755118000000

early a first 0.04769872271242600000000

early a expeditious 0.00160953449010869000000

early a initial 0.11256806019512100000000

early a precocious 0.00001385330864424430000

early a premature 0.01885895916156630000000

early a former 0.00173837717879901000000

early a young 0.03485367136600910000000

early a previous 0.14805979267060600000000

early a unexpected 0.00187705949545823000000

early a immature 0.00123346832364089000000

early a northern 0.00000388187001850670000

early a rapid 0.09596798322757770000000

early a recent 0.10003186721703400000000

early a timely 0.02167288460968200000000

early a direct 0.00554564687122867000000

early a raw 0.00003788323986825550000

early a advanced 0.09182610619011390000000

early a quick 0.00476979693613344000000

early a antecedent 0.00085410826257224400000

early a prime 0.00001975032516172670000

early a primitive 0.00146911639475722000000

early a prior 0.01979167117088250000000

early a immediate 0.10945679893232400000000

determination n resolution 0.00009245488665712400000

determination n decision 0.00383272164263043000000

determination n goal 0.00006597587006826740000

determination n reason 0.00090622110358530100000

determination n nerve 0.00001762054258197120000

determination n measurement 0.33407242395124600000000

determination n conclusion 0.17380978833939800000000

determination n design 0.01361802115693130000000

determination n perception 0.00069957968308088100000

determination n purpose 0.01147058002743920000000

determination n stability 0.00162012709514185000000

determination n confirmation 0.00896599689748663000000

determination n plan 0.00005768933496923750000

determination n adjudication 0.00000659889678573125000

determination n diagnosis 0.12136579704274400000000

determination n persistence 0.00230810517167807000000

determination n rationale 0.00281156780017971000000

determination n project 0.00024693141785709000000

determination n tendency 0.00020214183561728900000

determination n intention 0.00004195149897308870000

determination n opinion 0.00001484964664049990000

determination n tack 0.00000295956260433224000

determination n course 0.00423721480192376000000

determination n judgment 0.00045568957174231900000

determination n discovery 0.00002018410112739310000

determination n outcome 0.01222852124115460000000

determination n basis 0.00852277571614493000000

determination n independence 0.00026104840295908800000

determination n establishment 0.00018417992278746200000

determination n prognosis 0.00316119525183273000000

determination n heart 0.00001913221558377990000

determination n aim 0.00041704733035901300000

determination n objective 0.01667750656746710000000

determination n ground 0.00114600840845532000000

determination n end 0.00103399517789826000000

determination n finding 0.02887230011317970000000

determination n certainty 0.00060488723526817600000

determination n solution 0.00032508783560150300000

determination n result 0.24203836360540700000000

determination n verification 0.00005615369956840770000

determination n energy 0.00350860539724310000000

impede v hamper 0.01035597437365380000000

impede v hinder 0.00454326617682878000000

impede v block 0.36250753875141200000000

impede v stop 0.01418099649522310000000

impede v deter 0.23473541913615400000000

impede v restrict 0.00009687134705391810000

impede v prevent 0.35244217841891900000000

impede v occlude 0.00007265351029043840000

impede v interfere 0.00002421783676347900000

impede v slow 0.00002421783676347900000

impede v obstruct 0.00007265351029043840000

impede v inhibit 0.00964354259921760000000

impede v neutralize 0.00002421783676347900000

impede v interrupt 0.00009687134705391810000

impede v disrupt 0.00002421783676347900000

impede v delay 0.00060544591908699100000

impede v cross 0.00007265351029043840000

impede v check 0.00004843567352695870000

impede v arrest 0.00004843567352695870000

impede v offset 0.01035597437365380000000

impede v counteract 0.00002421783676347900000

crp n c-reactive protein 1.00000000000000000000000

tail v terminate 1.00000000000000000000000

interpretation n appreciation 0.00012453800766351700000

interpretation n meaning 0.00011587395749716900000

interpretation n clarification 0.00061142026109100700000

interpretation n theme 0.00000638177956932922000

interpretation n inference 0.00624232948437412000000

interpretation n elucidation 0.00005246320087513500000

interpretation n understanding 0.00117849369862547000000

interpretation n illustration 0.00072516817014910200000

interpretation n rendering 0.00002278007406420340000

interpretation n definition 0.00283574859446005000000

interpretation n criticism 0.00001347021939458470000

interpretation n critique 0.00038580899105737400000

interpretation n key 0.00253743658122172000000

interpretation n examination 0.08310366690187450000000

interpretation n discussion 0.02266591872780050000000

interpretation n demonstration 0.00073851235813341100000

interpretation n description 0.00129581004506595000000

interpretation n diagnosis 0.26009784597194700000000

interpretation n reading 0.04230757125429440000000

interpretation n explanation 0.01600249937916960000000

interpretation n account 0.00029102774827177500000

interpretation n version 0.00022379727261418800000

interpretation n statement 0.00116614275664453000000

interpretation n transcription 0.00038953108307176400000

interpretation n performance 0.01398920878662130000000

interpretation n analysis 0.16437581861247200000000

interpretation n presentation 0.01648320339967520000000

interpretation n study 0.34600444072140100000000

interpretation n answer 0.00249215661807963000000

interpretation n conception 0.00041138984595022300000

interpretation n idea 0.00067802025739586100000

interpretation n construction 0.00008474824756752580000

interpretation n commentary 0.00507759979831842000000

interpretation n translation 0.00027097017248753700000

interpretation n diagnostics 0.00198505235942052000000

interpretation n solution 0.00467691869214224000000

interpretation n representation 0.00001928742824294230000

interpretation n argument 0.00031694854129555700000

polyarteritis nodosa n periarteritis nodosa 1.00000000000000000000000

oncologic a oncological 1.00000000000000000000000

ny n new york 1.00000000000000000000000

requirement n specification 0.00136387110195718000000

requirement n requisite 0.00017183911798014000000

requirement n obligation 0.00484013515644062000000

requirement n reservation 0.00042707074909770200000

requirement n condition 0.02855876743697980000000

requirement n preoccupation 0.00000299616317545667000

requirement n element 0.00022524726729773000000

requirement n extremity 0.00000344135837465977000

requirement n demand 0.31085569261146500000000

requirement n charge 0.00004514865009975830000

requirement n precondition 0.00043006691227315900000

requirement n claim 0.00176718520658736000000

requirement n provision 0.00209495909979628000000

requirement n need 0.62750962015953700000000

requirement n stress 0.00004325024320641280000

requirement n urgency 0.00008238467107847690000

requirement n term 0.01471133966328370000000

requirement n exigency 0.00005338384363721270000

requirement n constraint 0.00417292026337988000000

requirement n call 0.00000104208450332437000

requirement n lack 0.00263664207667302000000

requirement n prerequisite 0.00000299616317545667000

branch v develop 1.00000000000000000000000

urge n relief 0.97876441522537100000000

urge n prayer 0.01342207571939090000000

urge n provocation 0.00151355321942068000000

urge n recommendation 0.00629995583581743000000

span n period 0.49510920788085800000000

span n range 0.40742746298689100000000

span n measure 0.00048268601925982400000

span n pair 0.00047181986479781300000

span n duration 0.00188727945919125000000

span n course 0.00346274752947265000000

span n interval 0.09067611024027020000000

span n time 0.00048268601925982400000

disperse v scatter 0.00854952925945003000000

disperse v distribute 0.94420167040950800000000

disperse v diffuse 0.01709905851890010000000

disperse v circulate 0.03014974181214160000000

writing n hand 0.03838567995461100000000

writing n letter 0.00130835916183026000000

writing n record 0.02308005483236120000000

writing n work 0.00046456772312083700000

writing n item 0.00028126381290313900000

writing n reporting 0.02606282331605530000000

writing n thesis 0.00028126381290313900000

writing n paper 0.00009165195510884920000

writing n review 0.80374630982606700000000

writing n publication 0.05270482048357850000000

writing n literature 0.00500077460202258000000

writing n sign 0.02343263013737080000000

writing n scenario 0.00009165195510884920000

writing n comment 0.00028126381290313900000

writing n note 0.00333605662311426000000

writing n article 0.00065417958091512700000

writing n indication 0.02079664841002640000000

thoroughly r completely 0.05714619337246510000000

thoroughly r comprehensively 0.00873077570600277000000

thoroughly r fully 0.70256991448779600000000

thoroughly r carefully 0.22158248268611000000000

thoroughly r entirely 0.00840597200345288000000

thoroughly r highly 0.00130109995123392000000

thoroughly r exhaustively 0.00026356179293960500000

sit v tend 0.00319408185975512000000

sit v remain 0.00052048743196510200000

sit v stop 0.01549782474172440000000

sit v introduce 0.01066331943949020000000

sit v rest 0.00607996283830580000000

sit v sleep 0.00873701813857611000000

sit v stand 0.95530730555018300000000

pregnant a critical 0.00439658253798349000000

pregnant a productive 0.00036557562228995800000

pregnant a fertile 0.02093605095501300000000

pregnant a full 0.00035564573320713300000

pregnant a replete 0.02685706878830230000000

pregnant a climacteric 0.02576316706476380000000

pregnant a original 0.00302562969854718000000

pregnant a important 0.06535729200365950000000

pregnant a suggestive 0.00095524458070568800000

pregnant a parturient 0.21549422005353200000000

pregnant a fruitful 0.00113803239185067000000

pregnant a gravid 0.10944652962356700000000

pregnant a potential 0.00447092322012668000000

pregnant a crucial 0.04986593202052880000000

pregnant a consequential 0.00009181885284656220000

pregnant a gestational 0.10307484051768800000000

pregnant a significant 0.26472144406182800000000

pregnant a hopeful 0.00009096895829841550000

pregnant a meaningful 0.00063848249718520600000

pregnant a abundant 0.00017478943391636000000

pregnant a pivotal 0.00009096895829841550000

pregnant a expectant 0.10268879242586200000000

reuptake n re-uptake 1.00000000000000000000000

patchy a random 0.34833374420116100000000

patchy a variable 0.65166625579883900000000

igg n immunoglobulin g 1.00000000000000000000000

currently r presently 1.00000000000000000000000

connecticut n ct 1.00000000000000000000000

comfortable a substantial 0.00113354925074094000000

comfortable a sufficient 0.00088248768374400900000

comfortable a acceptable 0.66639556618883400000000

comfortable a satisfactory 0.01047534952253350000000

comfortable a suitable 0.00304841542709122000000

comfortable a happy 0.00008415803785624140000

comfortable a easy 0.11416879415577700000000

comfortable a gratifying 0.00102230918718711000000

comfortable a healthy 0.00062063986020306600000

comfortable a well-fixed 0.00008415803785624140000

comfortable a useful 0.04035253974019500000000

comfortable a appropriate 0.04127466008048860000000

comfortable a wealthy 0.00004090730518159340000

comfortable a convenient 0.11653339430894900000000

comfortable a adequate 0.00379891317550611000000

comfortable a complacent 0.00008415803785624140000

vocal cord n vocal fold 1.00000000000000000000000

remission n pause 0.01690688512595720000000

remission n rest 0.00831781599060630000000

remission n decline 0.02010105784588990000000

remission n inactivity 0.00082791317751472000000

remission n relief 0.04517277887276280000000

remission n delay 0.06378061992195500000000

remission n alleviation 0.00068534591995123700000

remission n transmission 0.02519178868081220000000

remission n modification 0.02290125417224250000000

remission n contraction 0.05885017593891590000000

remission n subsidence 0.00021096550857061600000

remission n extension 0.00727299976085381000000

remission n reduction 0.09553161494756170000000

remission n cessation 0.00296140684492511000000

remission n lapse 0.00077732805176814700000

remission n submission 0.01127125675063810000000

remission n relaxation 0.00403209872664707000000

remission n discontinuation 0.09232563500063780000000

remission n breath 0.00035725952638199200000

remission n attenuation 0.00270887801959950000000

remission n deferral 0.00009691073394386740000

remission n termination 0.01807932870897630000000

remission n arrest 0.00161091967539622000000

remission n decrease 0.15538376791809300000000

remission n moderation 0.00196021856532838000000

remission n suspension 0.00003902339325091090000

remission n freedom 0.03296199996569840000000

remission n interruption 0.00008736154220388400000

remission n loosening 0.00048385146838637600000

remission n palliation 0.10622674374486200000000

remission n release 0.04766122929405360000000

remission n compensation 0.00194086879748393000000

remission n reimbursement 0.00060916038442868800000

remission n payment 0.00241821406872968000000

remission n continuation 0.03779401923272770000000

remission n shrinkage 0.00128597951397988000000

remission n discharge 0.11088190902609500000000

remission n diminution 0.00029341518217072100000

reinforcement n increase 0.01530877130110770000000

reinforcement n addition 0.98379071274000400000000

reinforcement n stay 0.00090051595888868700000

moiety n fraction 0.02580543476881630000000

moiety n fragment 0.17096100534340800000000

moiety n group 0.26918394319598700000000

moiety n hemisphere 0.27353760854945300000000

moiety n segment 0.26051200814233600000000

mib n m 1.00000000000000000000000

mucoviscidosis n cystic fibrosis 1.00000000000000000000000

issue v deliver 0.00182423289817899000000

issue v start 0.00803287413758727000000

issue v dispense 0.00014949048306941600000

issue v result 0.00996932676072843000000

issue v cut 0.00003111343295742760000

issue v remove 0.00752066511566815000000

issue v herald 0.00205617364440024000000

issue v begin 0.00066397237305298400000

issue v provide 0.02657794874133050000000

issue v release 0.02044935183658000000000

issue v rise 0.00077856584830450600000

issue v discharge 0.00062907536523509000000

issue v appear 0.00680237897395567000000

issue v emerge 0.00085740629178311200000

issue v disseminate 0.00014949048306941600000

issue v distribute 0.30187111547817400000000

issue v promulgate 0.00528364434650305000000

issue v transmit 0.00009334029887228460000

issue v send 0.00014949048306941600000

issue v supply 0.00524190432535600000000

issue v assign 0.00973504791252221000000

issue v express 0.00059948870752349600000

issue v derive 0.01897283956082280000000

issue v publish 0.57156106250125600000000

supplementation n supplement 1.00000000000000000000000

hierarchical a hierarchal 0.82191780821917800000000

hierarchical a hierarchic 0.17808219178082200000000

position n advantage 0.00052281628010167500000

position n function 0.03856159930728370000000

position n air 0.00000879286248579949000

position n placement 0.09110773689138240000000

position n scene 0.00000258371196785991000

position n consequence 0.00012598436386018800000

position n respect 0.00013753276217984400000

position n sway 0.00041718509020741200000

position n stance 0.00588110004637029000000

position n viewpoint 0.00008694839380934710000

position n view 0.03863968998920200000000

position n side 0.02136424661619080000000

position n contention 0.00079173447567174900000

position n level 0.03208516976403770000000

position n prominence 0.00000214255788560118000

position n insertion 0.02821637349311750000000

position n manner 0.00143092857937643000000

position n way 0.06354575557763030000000

position n role 0.00577196057684483000000

position n localization 0.00001113434640792670000

position n installation 0.00071087477950748100000

position n business 0.00296499403397409000000

position n fixation 0.01287556408502590000000

position n policy 0.01654436518872160000000

position n step 0.00052427803645538400000

position n situation 0.00402485054796965000000

position n area 0.03888897016949860000000

position n light 0.00038740110450852200000

position n environment 0.00693662922228821000000

position n thesis 0.00026210692070784400000

position n portion 0.00002431665856703130000

position n space 0.00416315309752781000000

position n setting 0.01631261301711080000000

position n condition 0.02986143755716830000000

position n office 0.00000758795658086783000

position n deposition 0.00003243427165070390000

position n degree 0.05006406724414210000000

position n perspective 0.00037680385267517900000

position n angle 0.01727969324461440000000

position n place 0.04230932217408700000000

position n site 0.00991934610055831000000

position n attitude 0.00019952564584842200000

position n assertion 0.00013063216445511600000

position n proposition 0.00004449961551380250000

position n principle 0.00097186032091562700000

position n stage 0.00078328163606042000000

position n estimation 0.00000214255788560118000

position n locus 0.00044402334350350100000

position n status 0.01413279689133990000000

position n reputation 0.00000171471895553690000

position n set 0.00015976124134515100000

position n face 0.00016325278622934400000

position n odds 0.00001717289470316290000

position n thought 0.00345915970630311000000

position n surroundings 0.00000446855710588890000

position n circumstance 0.00669638868479927000000

position n opinion 0.00123527165800116000000

position n pressure 0.02265468693709310000000

position n belief 0.00085448592978788400000

position n habit 0.00518873955945466000000

position n distinction 0.00003029677578832900000

position n plane 0.01339129544094890000000

position n disposition 0.00001915547599244910000

position n edge 0.00025569295877052600000

position n assignment 0.00004788868998112280000

position n mode 0.00301898314867670000000

position n region 0.03491037798408360000000

position n stature 0.01042796609434640000000

position n form 0.00086267823953425900000

position n port 0.00020994643530230500000

position n postulate 0.00000931969386520817000

position n employment 0.00022891498056417700000

position n job 0.01087078999764790000000

position n grade 0.00571330333065187000000

position n state 0.02458898440433400000000

position n posture 0.11243560403060200000000

position n conception 0.00017574274142766400000

position n spot 0.00000258371196785991000

position n feeling 0.00000916738438066197000

position n carriage 0.00000771273067180181000

position n hypothesis 0.00354371236673987000000

position n name 0.00000223427855294445000

position n idea 0.00305442059559995000000

position n appointment 0.00000931969386520817000

position n class 0.00007340068834626780000

position n standing 0.04361108671253180000000

position n occupation 0.00431188279669413000000

position n point 0.00026430499366375700000

position n importance 0.00023602123285803100000

position n elevation 0.02321858661295250000000

position n station 0.00004571576704365340000

position n influence 0.00853004193474612000000

position n notion 0.00016942823051280500000

position n character 0.00039583385500802400000

position n mind 0.00045342902285335200000

position n lodgment 0.00000916738438066197000

position n order 0.00478481986011312000000

position n arrangement 0.00000071376842416321900

position n location 0.04962676421287790000000

position n lot 0.00011403823207592700000

position n duty 0.00000258371196785991000

miserable a gloomy 0.01190476190476190000000

miserable a dismal 0.07142857142857140000000

miserable a poor 0.89285714285714300000000

miserable a grim 0.02380952380952380000000

misapprehension n error 1.00000000000000000000000

enuresis n urinary incontinence 1.00000000000000000000000

mien n feature 1.00000000000000000000000

guide v manage 0.08023762571032820000000

guide v regulate 0.00100729887653245000000

guide v direct 0.37320352445876500000000

guide v navigate 0.07504376630166760000000

guide v see 0.00130965538188142000000

guide v draw 0.00385378393043512000000

guide v lead 0.00016939902099699200000

guide v point 0.00001077131710946850000

guide v show 0.00087832870724649700000

guide v drive 0.07504376630166760000000

guide v take 0.00009719159218432780000

guide v conduct 0.00009694185398521710000

guide v advise 0.00000538565855473427000

guide v control 0.00017478467955172700000

guide v influence 0.32245537533158900000000

guide v suggest 0.00363908175482360000000

guide v supervise 0.00011845898390160600000

guide v recommend 0.06252562983776750000000

guide v educate 0.00012384464245634100000

guide v counsel 0.00000538565855473427000

synergism n synergy 1.00000000000000000000000

hygiene n health 1.00000000000000000000000

distinctly r markedly 1.00000000000000000000000

consumption n uptake 0.00294238168555573000000

consumption n decline 0.00099914778998209100000

consumption n waste 0.00002505036868726710000

consumption n ingestion 0.06132573873803080000000

consumption n reduction 0.01150212113743570000000

consumption n depletion 0.00103332770834976000000

consumption n wastage 0.00096443919445977900000

consumption n decrease 0.00431640743654394000000

consumption n destruction 0.00125191626203914000000

consumption n loss 0.00240123266605316000000

consumption n intake 0.46135773447540700000000

consumption n use 0.43919238735130300000000

consumption n expenditure 0.00102117091178094000000

consumption n decrement 0.00000207356730772527000

consumption n harm 0.00113786482383317000000

consumption n damage 0.00000207356730772527000

consumption n tb 0.00000599030555565079000

consumption n tuberculosis 0.00007418763034305990000

consumption n misuse 0.01044268081271700000000

consumption n burning 0.00000207356730772527000

morbid a unusual 0.24251166951819300000000

morbid a pathological 0.00180867559618066000000

morbid a febrile 0.01464084321458090000000

morbid a weak 0.00901898712742785000000

morbid a abnormal 0.09995855224900820000000

morbid a pathogenic 0.00153505749030969000000

morbid a psychotic 0.01464084321458090000000

morbid a malignant 0.06840356231651840000000

morbid a diabetic 0.41381372627084900000000

morbid a pathologic 0.13366808300235200000000

reentrant a re-entrant 1.00000000000000000000000

plausible a convincing 0.00067321401492569300000

plausible a apparent 0.01755595407895350000000

plausible a credible 0.01221475986228660000000

plausible a acceptable 0.00151036786735233000000

plausible a feasible 0.00147081671766814000000

plausible a superficial 0.00002543457063701780000

plausible a likely 0.31612023760834600000000

plausible a probable 0.02542927008878640000000

plausible a conceivable 0.02501919778689060000000

plausible a possible 0.53496346317115700000000

plausible a reasonable 0.06501728423299640000000

about r almost 0.11448968497044000000000

about r most 0.01106902368351280000000

about r nearly 0.12755288793141100000000

about r virtually 0.00594983526536615000000

about r near 0.00446434203679459000000

about r approximately 0.69688450962557500000000

about r generally 0.00919083442749241000000

about r roughly 0.03039888205940900000000

template n guide 1.00000000000000000000000

sulfonamide n sulfa 1.00000000000000000000000

paralyze v block 0.05068632076374360000000

paralyze v extend 0.00223766222197557000000

paralyze v prevent 0.00223766222197557000000

paralyze v paralyse 0.19154388620110900000000

paralyze v anesthetize 0.48373608735106800000000

paralyze v obstruct 0.00447532444395113000000

paralyze v disable 0.06840853078611020000000

paralyze v debilitate 0.13681706157222000000000

paralyze v immobilize 0.05985746443784640000000

mark n period 0.35923087687650800000000

mark n letter 0.00437063638751371000000

mark n goal 0.00171448873276429000000

mark n record 0.01217025024055600000000

mark n rating 0.01462673200139530000000

mark n score 0.00437063638751371000000

mark n model 0.00119098072275992000000

mark n smear 0.01462673200139530000000

mark n purpose 0.02298973528024840000000

mark n image 0.00496612674889367000000

mark n characteristic 0.01200142112935000000000

mark n marker 0.00059549036137996200000

mark n figure 0.00437063638751371000000

mark n gauge 0.03900461867038750000000

mark n scale 0.03900461867038750000000

mark n sign 0.23402771202232500000000

mark n badge 0.00059549036137996200000

mark n objective 0.08155511176535570000000

mark n end 0.02228835352593570000000

mark n device 0.00178647108413989000000

mark n importance 0.00171448873276429000000

mark n symptom 0.11701385601116300000000

mark n target 0.00407004716560565000000

mark n indication 0.00171448873276429000000

uptake n consumption 0.13508968794354600000000

uptake n understanding 0.00515058533068558000000

uptake n ingestion 0.01219569126509980000000

uptake n sensitivity 0.54926237726459000000000

uptake n perception 0.00117108044080565000000

uptake n insight 0.00025644222501727500000

uptake n intake 0.28915885101294400000000

uptake n acuity 0.00771528451731268000000

gonorrhoea n gonorrhea 1.00000000000000000000000

lop v reduce 1.00000000000000000000000

stringent a compelling 0.01128798791012060000000

stringent a effective 0.03386396373036190000000

stringent a good 0.26865196714161400000000

stringent a influential 0.01128798791012060000000

stringent a important 0.06286172934737530000000

stringent a fixed 0.00044704902614339100000

stringent a crucial 0.01107500700615610000000

stringent a strict 0.42550375136798100000000

stringent a rigid 0.01084283315951170000000

stringent a rigorous 0.16102165178089200000000

stringent a strong 0.00229287254424325000000

stringent a firm 0.00086319907547981200000

pole n limit 1.00000000000000000000000

lengthen v prolong 0.24826095549565000000000

lengthen v extend 0.18172552543813100000000

lengthen v expand 0.00540384108318777000000

lengthen v augment 0.00001303649472384920000

lengthen v produce 0.00007500795601615130000

lengthen v enlarge 0.00167981579495706000000

lengthen v dilate 0.00035257025635811300000

lengthen v amplify 0.00009566427673946180000

lengthen v continue 0.00006377618449297450000

lengthen v increase 0.54718732773226300000000

lengthen v stretch 0.00009566427673946180000

lengthen v proceed 0.00003188809224648730000

lengthen v protract 0.00007500795601615130000

lengthen v reach 0.00016485712995682400000

lengthen v develop 0.01477506183252140000000

granulocytopenia n agranulocytosis 1.00000000000000000000000

afternoon n decline 0.00200542055459163000000

afternoon n evening 0.99799457944540800000000

latterly r recently 1.00000000000000000000000

nc n north carolina 1.00000000000000000000000

mold n block 0.27345627939342400000000

mold n manner 0.04263136314040720000000

mold n model 0.09146364715104040000000

mold n pattern 0.05260390520958230000000

mold n parasite 0.40499794983386800000000

mold n image 0.01910851439647060000000

mold n figure 0.00095957688149881800000

mold n depression 0.01083309840116860000000

mold n cup 0.00554791712101190000000

mold n fungus 0.08919597704674490000000

mold n formation 0.00604474551990848000000

mold n kidney 0.00315702590487471000000

languish v give 1.00000000000000000000000

macula n smear 0.01902682697423470000000

macula n defect 0.86325528740779000000000

macula n spot 0.09869105864374060000000

macula n nevus 0.01902682697423470000000

account v believe 0.00211027057494523000000

account v cause 0.05281323214976070000000

account v attribute 0.07288270466499950000000

account v rate 0.00077655579966220500000

account v describe 0.00560282409262163000000

account v count 0.00015939248173137500000

account v consider 0.07966304074315540000000

account v judge 0.00021551955149066100000

account v view 0.00029365282207275700000

account v hold 0.00000775856394505386000

account v estimate 0.00613661273380017000000

account v assign 0.00020204156669248900000

account v solve 0.00005095487572937820000

account v calculate 0.03764727860979030000000

account v charge 0.00000422914305660008000

account v report 0.08680408262596220000000

account v regard 0.00069951283783798500000

account v explain 0.65393033616274600000000

lag n delay 0.03598255695253800000000

lag n meantime 0.00242873479242673000000

lag n interval 0.95915997346260900000000

lag n interim 0.00242873479242673000000

travel v walk 0.21166237113402100000000

travel v progress 0.00048324742268041200000

travel v cover 0.10583118556701000000000

travel v visit 0.47036082474226800000000

travel v migrate 0.21166237113402100000000

protection n reassurance 0.00798953364365963000000

protection n maintenance 0.02539305421997370000000

protection n strength 0.00168557531289333000000

protection n pass 0.00003044854498130590000

protection n cushion 0.00005972458071254260000

protection n preservation 0.61925635739193500000000

protection n stability 0.02211624501184630000000

protection n barrier 0.01294603928470200000000

protection n immunity 0.15241706086091300000000

protection n screen 0.00050800662382157300000

protection n care 0.07445863057592810000000

protection n charge 0.00066385605373967200000

protection n safety 0.06274473375414040000000

protection n certainty 0.00378892773567629000000

protection n support 0.01594180640507720000000

cerebrovascular accident n stroke 1.00000000000000000000000

intermission n interval 0.50000000000000000000000

intermission n time 0.50000000000000000000000

insect n jerk 0.00108273904773871000000

insect n spider 0.09203281905779070000000

insect n bee 0.00483227212236610000000

insect n ant 0.04307919189939140000000

insect n mosquito 0.67490733975713200000000

insect n tick 0.18406563811558100000000

import v present 0.97306532750249700000000

import v concern 0.00013539163293965700000

import v indicate 0.00936249103343336000000

import v introduce 0.00324939919055178000000

import v mark 0.00227292694188851000000

import v suggest 0.01191446369868990000000

period n year 0.13727305244633500000000

period n hour 0.01658600310213540000000

period n determination 0.00047446445088067300000

period n span 0.00054786837956140200000

period n menstruation 0.00029758645635684600000

period n bout 0.00003436107034152240000

period n spell 0.00002597545076823410000

period n fall 0.00045303941811890500000

period n generation 0.00004276740413187440000

period n round 0.00007859624639965380000

period n epoch 0.00025788675638114000000

period n decade 0.00174816504590173000000

period n teens 0.00000968079199152908000

period n conclusion 0.05781675034794800000000

period n date 0.00230488228789545000000

period n middle age 0.00000014712859039852400

period n session 0.00687804627283282000000

period n limit 0.00019281181896427000000

period n space 0.00030086767745952500000

period n day 0.06692293893831190000000

period n duration 0.05941116422736120000000

period n fiscal year 0.00013921321100929000000

period n patch 0.00036463728929677300000

period n minute 0.00769243332172749000000

period n stage 0.00354651210103016000000

period n lifetime 0.00021223668331643700000

period n autumn 0.00000434138057382837000

period n month 0.16312867451426000000000

period n menstrual period 0.00007848395318965290000

period n recurrence 0.00645020565787794000000

period n semester 0.00000697396231079377000

period n era 0.00133783051690275000000

period n physiology 0.00000984023183725947000

period n circuit 0.00000982647832832889000

period n flow 0.00053621384215972000000

period n phase 0.03886244305577060000000

period n season 0.00086171538700333400000

period n age 0.09808978410812740000000

period n interval 0.08794864861417280000000

period n time 0.12536238785894100000000

period n century 0.00027601002209650400000

period n term 0.00268505174083268000000

period n quarter 0.00016697724432623700000

period n second 0.00417527907664844000000

period n end 0.02535544518460670000000

period n summer 0.00021923045799551500000

period n winter 0.00060128212082237800000

period n week 0.06509548715128610000000

period n point 0.00334084705025238000000

period n dot 0.00000039192125251430900

period n moment 0.00000172405541729044000

period n half 0.00243961208614087000000

period n spring 0.00034168965536632900000

period n menses 0.00075707169181384400000

period n stop 0.00000045580566666003200

period n cycle 0.00824398684897276000000

apparent a open 0.00005858743371673500000

apparent a plausible 0.00466485834069619000000

apparent a discernible 0.00355939625825102000000

apparent a certain 0.01658965317688870000000

apparent a distinct 0.00893769731477372000000

apparent a noticeable 0.00088483464466985100000

apparent a external 0.00290832943377943000000

apparent a observable 0.00148950319721181000000

apparent a conspicuous 0.01629649129473870000000

apparent a evident 0.33568622761973900000000

apparent a credible 0.00000650407027123339000

apparent a clear 0.12462872189001800000000

apparent a superficial 0.01558325772408530000000

apparent a likely 0.00193670627165900000000

apparent a probable 0.01546322237230620000000

apparent a prominent 0.02574898955089140000000

apparent a conceivable 0.00041946063062299800000

apparent a possible 0.14584830661367700000000

apparent a understandable 0.00006107434386802700000

apparent a visible 0.02062493942264560000000

apparent a plain 0.01610613190850160000000

apparent a overt 0.03352081895006990000000

apparent a obvious 0.09744556029300170000000

apparent a palpable 0.00617254515904240000000

apparent a patent 0.00239444558782036000000

apparent a striking 0.01745013152057260000000

apparent a seeming 0.00778289153772767000000

apparent a illusory 0.00000315904311202158000

apparent a manifest 0.01087313944262830000000

apparent a marked 0.06685441495301390000000

endorse v witness 0.00018888043548460100000

endorse v assist 0.00153371309589253000000

endorse v guarantee 0.00365936254660774000000

endorse v validate 0.00410889402330353000000

endorse v sustain 0.00628101903137648000000

endorse v affirm 0.00009444021774229920000

endorse v warrant 0.03206739314043390000000

endorse v defend 0.00039040839515363100000

endorse v encourage 0.00419818121509823000000

endorse v confirm 0.01958742041491020000000

endorse v authorize 0.00018888043548460100000

endorse v approve 0.00018888043548460100000

endorse v accept 0.34384587026947000000000

endorse v favor 0.00018888043548460100000

endorse v uphold 0.00009444021774229920000

endorse v advocate 0.00820925506180261000000

endorse v pass 0.00009444021774229920000

endorse v support 0.35580218811988900000000

endorse v assign 0.00705421206426123000000

endorse v prove 0.01202069180466800000000

endorse v note 0.00425979639928058000000

endorse v document 0.01031337123382840000000

endorse v recommend 0.17741684361495800000000

endorse v substantiate 0.00802365673841619000000

endorse v corroborate 0.00018888043548460100000

bar n blockage 0.00402950839035846000000

bar n block 0.00242231897512006000000

bar n restaurant 0.95197135722218600000000

bar n measure 0.00182895553741054000000

bar n pig 0.00968927590048026000000

bar n barrier 0.00182895553741054000000

bar n band 0.00645182736547852000000

bar n tablet 0.00402950839035846000000

bar n beam 0.00242231897512006000000

bar n strip 0.00726695692536020000000

bar n line 0.00402950839035846000000

bar n strut 0.00402950839035846000000

act n proposal 0.11195543034709200000000

act n determination 0.00114605686855628000000

act n command 0.00016372240979375400000

act n effect 0.00806885984483543000000

act n work 0.00016372240979375400000

act n conclusion 0.45560992523859900000000

act n task 0.00065488963917501700000

act n measure 0.00049116722938126300000

act n step 0.00016372240979375400000

act n exercise 0.01122637445590130000000

act n operation 0.08901587420486420000000

act n maneuver 0.00016372240979375400000

act n performance 0.00705231057304514000000

act n action 0.29854781425891100000000

act n order 0.01557640770046490000000

limited a topical 0.00010842144951679400000

limited a particular 0.04384290257980940000000

limited a little 0.14321862562858400000000

limited a slow 0.00456991079566837000000

limited a special 0.00571919966178687000000

limited a inadequate 0.03715277512790800000000

limited a scanty 0.00147192456562498000000

limited a faulty 0.00007830438020657320000

limited a small 0.33595130519070900000000

limited a slight 0.01184094906367920000000

limited a insufficient 0.11468503257261500000000

limited a restricted 0.01844474919626660000000

limited a poor 0.09174222377344060000000

limited a short 0.04687073973396790000000

limited a local 0.00850421757091688000000

limited a determinate 0.00001191108882015480000

limited a simple 0.00697764439904493000000

limited a minimal 0.09270506744420350000000

limited a sparse 0.03610409577723120000000

front n movement 0.00392014614234893000000

front n nerve 0.35351676878567300000000

front n presence 0.00392014614234893000000

front n expression 0.00784029228469788000000

front n look 0.00784029228469788000000

front n figure 0.00392014614234893000000

front n map 0.00392014614234893000000

front n display 0.00392014614234893000000

front n breast 0.00392014614234893000000

front n behavior 0.60336162364848900000000

front n appearance 0.00392014614234893000000

calif n ca 0.53530145532985100000000

calif n california 0.46469854467014900000000

overall r primarily 0.03147994596984620000000

overall r mainly 0.00240347562120709000000

overall r predominantly 0.00278981974979459000000

overall r mostly 0.00213789277249368000000

overall r altogether 0.95351335951343100000000

overall r throughout 0.00767550637322784000000

milieu n environment 0.50128323759973500000000

milieu n setting 0.01291967107215810000000

milieu n condition 0.06782827312883010000000

milieu n medium 0.40696963877298000000000

milieu n background 0.01099917942629680000000

ideal n value 0.39106480332762100000000

ideal n height 0.15806644727350100000000

ideal n type 0.03508641531016440000000

ideal n goal 0.00011924746539610100000

ideal n model 0.03847434057460800000000

ideal n design 0.26620000178509000000000

ideal n example 0.00118158447447278000000

ideal n purpose 0.00559348367835177000000

ideal n pattern 0.00594033589505184000000

ideal n principle 0.00023849493079220100000

ideal n belief 0.00060053166718149700000

ideal n aim 0.05357744454518890000000

ideal n objective 0.02828360993024670000000

ideal n target 0.00668227463497409000000

ideal n standard 0.00889098450735970000000

govern v direct 1.00000000000000000000000

fit a particular 0.00141774454853500000000

fit a able 0.34649491440110600000000

fit a eligible 0.00330189307708914000000

fit a healthy 0.57565693320876200000000

fit a occasional 0.00094207426427707000000

fit a appropriate 0.07218644050023050000000

curvature n shape 1.00000000000000000000000

deliver v dispense 0.00002359037263519460000

deliver v return 0.00013381801477494500000

deliver v yield 0.00000256589169023139000

deliver v convey 0.00001627281520234840000

deliver v bear 0.34214646905617400000000

deliver v address 0.00067862947630772100000

deliver v present 0.02298125959494260000000

deliver v protect 0.00087374954395635700000

deliver v preserve 0.00758008111914586000000

deliver v direct 0.00002618131434823820000

deliver v release 0.02869993640949710000000

deliver v give 0.14369388489664100000000

deliver v discharge 0.02282487533812120000000

deliver v grant 0.00000769767507069431000

deliver v launch 0.00000739699258776109000

deliver v produce 0.01837401830536080000000

deliver v voice 0.00000739699258776109000

deliver v expel 0.00000739699258776109000

deliver v administer 0.28769275583961200000000

deliver v keep 0.00624789855663116000000

deliver v have 0.08906342160961010000000

deliver v transfer 0.00001998109057691820000

deliver v distribute 0.00001479398517552220000

deliver v promulgate 0.00001532944519108520000

deliver v pronounce 0.00000256589169023139000

deliver v transmit 0.00249473096068902000000

deliver v render 0.00501591137404260000000

deliver v state 0.00004190599303039570000

deliver v take 0.00567149170733940000000

deliver v send 0.00001784095174921390000

deliver v save 0.00006559616519985870000

deliver v bring 0.00000385885692105248000

deliver v drop 0.00009212026519244930000

deliver v pass 0.00019605976916178800000

deliver v broach 0.00000549216036573794000

deliver v read 0.00135656361033727000000

deliver v express 0.00004449762379489440000

deliver v deal 0.00000809669002371674000

deliver v cast 0.00001059289530169450000

deliver v communicate 0.00151025667854965000000

deliver v transport 0.00000310135170579440000

deliver v speak 0.00000053546001556300900

deliver v help 0.00003627632977373400000

deliver v carry 0.00929306942111562000000

deliver v tell 0.00069642343940078600000

deliver v rescue 0.00000739699258776109000

deliver v publish 0.00153379804058308000000

deliver v abandon 0.00075241604300073200000

story n news 0.06581467309632120000000

story n myth 0.00001923876199596650000

story n level 0.00018909924583921100000

story n item 0.00017476561115861600000

story n report 0.28840096948261700000000

story n stage 0.00067241777532178600000

story n feature 0.02494596656782210000000

story n history 0.00013138295985131000000

story n information 0.00781383399073956000000

story n life 0.00001923876199596650000

story n statement 0.00001923876199596650000

story n episode 0.00018581087171875200000

story n word 0.57807221202935500000000

story n scenario 0.00012869213740941500000

story n incident 0.03109838994636990000000

story n article 0.00212006562633460000000

story n stratum 0.00017476561115861600000

story n intelligence 0.00001923876199596650000

sort v divide 0.71031025446248900000000

sort v screen 0.00045491114967239400000

sort v segregate 0.00599832844496599000000

sort v grade 0.00124965175936791000000

sort v separate 0.03864496850609240000000

sort v categorize 0.04246357650521010000000

sort v distribute 0.00229444257457716000000

sort v select 0.00267278015041163000000

sort v subdivide 0.01288165616869750000000

sort v group 0.03064566557552720000000

sort v classify 0.15238376470298900000000

papule n comedo 0.93606852556847400000000

papule n swelling 0.03594711390060710000000

papule n eruption 0.00042596974997427700000

papule n spot 0.02713242103097030000000

papule n inflammation 0.00042596974997427700000

elect v return 0.00179811339079057000000

elect v resolve 0.00090318597744471000000

elect v choose 0.41834019864661900000000

elect v appoint 0.00057638155266437300000

elect v judge 0.00737229176361743000000

elect v accept 0.00002990890911607840000

elect v mark 0.00005981781823215700000

elect v select 0.05490173283594620000000

elect v take 0.00489076071899163000000

elect v conclude 0.00052009707471568900000

elect v prefer 0.13573645929955700000000

elect v designate 0.00436762574875518000000

elect v decide 0.29069225459347100000000

elect v determine 0.04155491898715130000000

elect v admit 0.03825625268292810000000

selection n determination 0.01569191194373660000000

selection n variety 0.01721133529055990000000

selection n differentiation 0.02692305209071720000000

selection n survival 0.13549822356849300000000

selection n option 0.05645236853027320000000

selection n modification 0.01123843509065660000000

selection n range 0.00549974848888754000000

selection n reservation 0.00058634494781696500000

selection n collection 0.00007534337183524690000

selection n extract 0.00140538809398313000000

selection n pick 0.00001438239750304580000

selection n mixture 0.00054382000739494100000

selection n preference 0.03469529932846620000000

selection n set 0.00177329180601213000000

selection n separation 0.00051168077362513900000

selection n discrimination 0.00033159043380317900000

selection n adaptation 0.00037527024452510300000

selection n passage 0.00013726323753934200000

selection n series 0.05050780865255670000000

selection n group 0.42551342399394600000000

selection n adoption 0.00501010817018599000000

selection n choice 0.21000390953748400000000

attribute n dimension 0.00042763720446030400000

attribute n feature 0.00149661255133669000000

attribute n quality 0.03238254454093300000000

attribute n trait 0.53431198492539400000000

attribute n characteristic 0.32748722370904900000000

attribute n sign 0.05936799832504380000000

attribute n indication 0.04452599874378280000000

quiescent a inert 0.01756950140856300000000

quiescent a immobile 0.01756950140856300000000

quiescent a passive 0.01756950140856300000000

quiescent a stagnant 0.01756950140856300000000

quiescent a inactive 0.92972199436574800000000

nasal a adenoidal 1.00000000000000000000000

heartache n distress 1.00000000000000000000000

albuminuria n proteinuria 1.00000000000000000000000

schizophrenia n schizophrenic disorder 1.00000000000000000000000

fellow n man 0.02251562428032210000000

fellow n boy 0.00180576445237369000000

fellow n member 0.92519981033297200000000

fellow n duplicate 0.00050625262792008900000

fellow n instructor 0.00194689279510499000000

fellow n cat 0.00112177747207387000000

fellow n doctor 0.01182868919836760000000

fellow n male 0.00010125052558401800000

fellow n partner 0.00194689279510499000000

fellow n individual 0.02295401946427530000000

fellow n youth 0.00102052694648986000000

fellow n cohort 0.00306158083946957000000

fellow n person 0.00497039132345172000000

fellow n teenager 0.00102052694648986000000

facet n factor 0.03051811832091280000000

facet n side 0.02630227392939870000000

facet n element 0.02963890344164410000000

facet n detail 0.05681291168750130000000

facet n feature 0.71761893825551200000000

facet n characteristic 0.00009769054214096660000

facet n phase 0.06001363910651540000000

facet n component 0.05259756024483530000000

facet n point 0.02639996447153970000000

excitation n inflammation 1.00000000000000000000000

methotrexate n amethopterin 1.00000000000000000000000

oesophagitis n esophagitis 1.00000000000000000000000

numbness n paralysis 0.20129659238533000000000

numbness n confusion 0.79870340761467000000000

merely r solely 0.40000000000000000000000

merely r only 0.40000000000000000000000

merely r simply 0.20000000000000000000000

gloomy a miserable 0.06250000000000000000000

gloomy a dismal 0.37500000000000000000000

gloomy a bad 0.31250000000000000000000

gloomy a grave 0.06250000000000000000000

gloomy a hopeless 0.06250000000000000000000

gloomy a grim 0.12500000000000000000000

graduate v finish 1.00000000000000000000000

residual a redundant 0.00086719047984690200000

residual a excessive 0.09504374705815270000000

residual a unnecessary 0.16729888651788800000000

residual a net 0.72043354544687400000000

residual a supplementary 0.01635663049723810000000

childbirth n labor 0.18287700993732500000000

childbirth n labour 0.12380485781157800000000

childbirth n delivery 0.40157876770236900000000

childbirth n birth 0.29173936454872900000000

advisable a prudent 0.10594486753616600000000

advisable a correct 0.00253466119320385000000

advisable a good 0.12870184705927000000000

advisable a practical 0.00985907319044346000000

advisable a suitable 0.03170863137091460000000

advisable a proper 0.00096841755978095500000

advisable a sensible 0.00005065306141962470000

advisable a apt 0.00036548132008930200000

advisable a useful 0.32078409608075300000000

advisable a reasonable 0.05326204322943330000000

advisable a rational 0.00500254332372108000000

advisable a profitable 0.00022659841845536700000

advisable a right 0.00047344548801250500000

advisable a appropriate 0.18726245397875300000000

advisable a treatable 0.00160913522666079000000

advisable a desirable 0.06751594881384480000000

advisable a sound 0.00023905171867908700000

advisable a advantageous 0.01121295842458790000000

advisable a judicious 0.00039207357230520500000

advisable a commendable 0.00028599800387570600000

advisable a beneficial 0.07160002142963200000000

ace n one 0.05991472944805600000000

ace n sensation 0.00006338638992470830000

ace n angiotensin-converting enzyme 0.73870968086966300000000

ace n angiotensin converting enzyme 0.20131220329235600000000

packing n compression 0.99343363610661300000000

packing n arrangement 0.00656636389338675000000

transfusion n transmission 0.02704137911788700000000

transfusion n transfer 0.00064359602246394700000

transfusion n blood transfusion 0.85646053130640700000000

transfusion n bleeding 0.11585449355324200000000

dominance n control 1.00000000000000000000000

value n appreciation 0.00087986133999010600000

value n interpretation 0.00785027342483066000000

value n advantage 0.01072365838172930000000

value n meaning 0.00014169922436938100000

value n consequence 0.00293144296203423000000

value n estimate 0.01175569593995870000000

value n evaluation 0.04824316323890190000000

value n usefulness 0.06304140944218240000000

value n utility 0.06330873648066030000000

value n merit 0.00102456330647051000000

value n consideration 0.00692095013434350000000

value n power 0.01824091229647180000000

value n regard 0.00276815778758370000000

value n tax 0.00000582229848247972000

value n excellence 0.00004012045963696860000

value n drift 0.00000059260730332931300

value n assessment 0.02868381637379400000000

value n substance 0.00025803108552202600000

value n condition 0.03215429362584760000000

value n purpose 0.02922665324145630000000

value n force 0.00159848680626161000000

value n profit 0.00003561533085102190000

value n worth 0.00000942372022421975000

value n exchange 0.00065852184933162700000

value n preference 0.00151988088358921000000

value n estimation 0.00717928958934567000000

value n import 0.00016750632863462800000

value n equivalent 0.00028383725171470900000

value n quality 0.01000423149092050000000

value n charge 0.00062884833592733300000

value n intention 0.00170857923585521000000

value n gain 0.00154427520354369000000

value n distinction 0.00132382605613623000000

value n benefit 0.04594981481133470000000

value n use 0.11059265072207300000000

value n rate 0.06883302123161690000000

value n remuneration 0.00000218966858006603000

value n cost 0.01187098727871050000000

value n bearing 0.00001577326804866560000

value n connotation 0.00026774861365787800000

value n grade 0.00355217190600556000000

value n weight 0.01219630373278660000000

value n intent 0.00035544966663698100000

value n state 0.01877230445584580000000

value n sense 0.00000987061539607890000

value n expense 0.00068698690916665900000

value n superiority 0.00022268736022930000000

value n amount 0.00125383571860361000000

value n appraisal 0.00130565368162412000000

value n implication 0.07392166509377870000000

value n significance 0.17251265831136900000000

value n content 0.01448908683949120000000

value n point 0.03557985025394910000000

value n importance 0.07262947070972320000000

value n moment 0.00000511792404714469000

value n avail 0.00000297279710752494000

value n caliber 0.00001648321944811630000

value n price 0.00009306947686419450000

correspond v conform 0.00906467311377639000000

correspond v query 0.00004607090974114090000

correspond v equate 0.00063635444079950900000

correspond v compare 0.39628249875023800000000

correspond v concur 0.04074098305579640000000

correspond v relate 0.04128883532709500000000

correspond v correlate 0.22288521124021300000000

correspond v approach 0.00155365031087468000000

correspond v agree 0.00010858551631714300000

correspond v question 0.00453356498352899000000

correspond v write 0.00548207266360394000000

correspond v parallel 0.00011260916054643900000

correspond v fit 0.00012868960165031800000

correspond v answer 0.00183788171975477000000

correspond v represent 0.09060074711886800000000

correspond v belong 0.00545322568906806000000

correspond v respond 0.05196292403993900000000

correspond v communicate 0.00246828389158599000000

correspond v coincide 0.00015514927318540400000

correspond v check 0.00051321541579334100000

correspond v follow 0.04928718380833090000000

correspond v match 0.07415316372363520000000

correspond v amount 0.00061863317789117100000

correspond v comply 0.00008579306776687880000

motivate v cause 0.18424618396897100000000

motivate v trigger 0.00001983416649587020000

motivate v encourage 0.18434535480145000000000

motivate v draw 0.00001983416649587020000

motivate v lead 0.00012727113790166100000

motivate v persuade 0.27636927595345700000000

motivate v stimulate 0.06909231898836420000000

motivate v move 0.00001983416649587020000

motivate v suggest 0.27654778345192000000000

motivate v manipulate 0.00921230919844856000000

leak v release 0.07462686567164180000000

leak v discharge 0.08955223880597020000000

leak v relate 0.11940298507462700000000

leak v reveal 0.01492537313432830000000

leak v unveil 0.01492537313432830000000

leak v inform 0.01492537313432830000000

leak v communicate 0.01492537313432830000000

leak v expose 0.01492537313432830000000

leak v report 0.50746268656716500000000

leak v tell 0.04477611940298500000000

leak v uncover 0.01492537313432830000000

leak v publish 0.07462686567164180000000

intrauterine device n iud 1.00000000000000000000000

hope v believe 0.06695797504303050000000

hope v expect 0.13371729180886000000000

hope v aim 0.63983266967328300000000

hope v plan 0.00952055518736064000000

hope v assume 0.01958203018121380000000

hope v presume 0.00279743288303055000000

hope v anticipate 0.04782518139066940000000

hope v intend 0.00063681125000795600000

hope v wish 0.07913005258254400000000

cell membrane n plasma membrane 1.00000000000000000000000

critical a determinative 0.00000320369265212796000

critical a compelling 0.00069768765788092600000

critical a acute 0.03094367120113570000000

critical a severe 0.01646889573866950000000

critical a paramount 0.00267980239949507000000

critical a particular 0.00612897964060991000000

critical a essential 0.06181582376447830000000

critical a risky 0.00115669441613345000000

critical a correct 0.00669629989945263000000

critical a unfavourable 0.00001122415727906290000

critical a interpretive 0.00062786162986776700000

critical a high-priority 0.00005676933275756690000

critical a grave 0.00004129298534511470000

critical a climacteric 0.00000401163212122924000

critical a nice 0.00002949012750254530000

critical a decisive 0.00267141325191513000000

critical a important 0.60800432139987100000000

critical a editorial 0.00064025412328920600000

critical a evaluative 0.00047017482554783200000

critical a detailed 0.01263842008917630000000

critical a uncertain 0.00227146630662877000000

critical a precise 0.00573271180866154000000

critical a urgent 0.02002347987157980000000

critical a crucial 0.03158871004313940000000

critical a dubious 0.00006517479018045870000

critical a negative 0.00432966016421984000000

critical a significant 0.09851699328242380000000

critical a exact 0.00440572669787366000000

critical a subtle 0.00039000213309622700000

critical a picky 0.00000802326424245847000

critical a vital 0.01303484068626390000000

critical a integral 0.00133114483320988000000

critical a sharp 0.00000246420947356310000

critical a fine 0.00014376076379410300000

critical a fundamental 0.00482171177598515000000

critical a dangerous 0.00036088219677405000000

critical a discriminating 0.00003561898617658950000

critical a serious 0.00624337425215759000000

critical a pivotal 0.02585437888986470000000

critical a hazardous 0.00025821904221099700000

critical a accurate 0.00295556081334364000000

critical a difficult 0.01810523024550110000000

critical a meticulous 0.00008264719224900270000

critical a imperative 0.00760590051857299000000

critical a analytical 0.00004602526719658520000

yellow a low 1.00000000000000000000000

vigilance n surveillance 0.62703768517886300000000

vigilance n caution 0.22928896604166500000000

vigilance n observation 0.11696630909421000000000

vigilance n precaution 0.02670703968526250000000

gender n sex 1.00000000000000000000000

impressive a convincing 0.00835309371881347000000

impressive a exciting 0.00011110407556132600000

impressive a effective 0.17665315027900800000000

impressive a extraordinary 0.03266350187740110000000

impressive a important 0.29737366243230000000000

impressive a formidable 0.00114737205086583000000

impressive a prominent 0.05048102998108560000000

impressive a profound 0.00203351815661359000000

impressive a dramatic 0.06697175819692010000000

impressive a intense 0.00416961734309127000000

impressive a consequential 0.00008869068718376230000

impressive a powerful 0.00008136508713810900000

impressive a persuasive 0.00096147017956907600000

impressive a striking 0.28480256699019900000000

impressive a vital 0.00036344730471294200000

impressive a remarkable 0.02510260428663200000000

impressive a notable 0.02214065436697480000000

impressive a serious 0.02650139298592990000000

halo n air 0.06270823142675490000000

halo n band 0.09809746922805890000000

halo n ring 0.15273009973784400000000

halo n quality 0.65120086481630100000000

halo n flow 0.03526333479104130000000

pause n delay 0.99853264856933200000000

pause n interval 0.00146735143066764000000

immediately r dead 0.00008481578349383830000

immediately r directly 0.01055956504498290000000

immediately r rapidly 0.06902716545858740000000

immediately r now 0.01648858885832720000000

immediately r urgently 0.00017746894539113800000

immediately r abruptly 0.00345261929088311000000

immediately r closely 0.00006027579048152910000

immediately r just 0.43263007102834100000000

immediately r instantaneously 0.00003653828735288190000

immediately r instantly 0.04223826017993150000000

immediately r shortly 0.24306289993114000000000

immediately r promptly 0.17665302218409700000000

immediately r quickly 0.00419478432295881000000

immediately r precisely 0.00009162312403455850000

immediately r suddenly 0.00124230176999799000000

disparate a divergent 0.19844650799402100000000

disparate a different 0.69099301576324500000000

disparate a various 0.00186437351171386000000

disparate a discordant 0.05835614542712140000000

disparate a inconsistent 0.05033995730389890000000

contact lens n contact 1.00000000000000000000000

chill n fever 0.00152978135125480000000

chill n cough 0.99847021864874500000000

directly r dead 0.00022721662346524300000

directly r immediately 0.10378119276775000000000

directly r now 0.02444617764379500000000

directly r personally 0.28335660766296300000000

directly r soon 0.00070063252501434500000

directly r promptly 0.00022721662346524300000

directly r quickly 0.55401182886665900000000

directly r precisely 0.03324912728688830000000

thiamine n thiamin 1.00000000000000000000000

sign v indicate 0.00665216641243873000000

sign v confirm 0.00080880165397137500000

sign v show 0.00080880165397137500000

sign v write 0.86194835270448400000000

sign v assign 0.04933690089225390000000

sign v note 0.00080880165397137500000

sign v get 0.07400535133838080000000

sign v suggest 0.00443477760829248000000

sign v enroll 0.00119604608223646000000

dose v medicate 0.00001160399969979030000

dose v give 0.27191250159533900000000

dose v administer 0.47895280564474100000000

dose v treat 0.14131854641740300000000

dose v load 0.10780454234281700000000

aluminium n al 0.06704827681038900000000

aluminium n aluminum 0.93295172318961100000000

virulent a aggressive 0.46654548680851000000000

virulent a severe 0.48324868517765500000000

virulent a blistering 0.00039803845133720900000

virulent a toxic 0.00482568878635324000000

virulent a infectious 0.00602445970451864000000

virulent a malignant 0.00241284439317662000000

virulent a detrimental 0.00039803845133720900000

virulent a infective 0.03614675822711190000000

trust n protection 0.07979753055154880000000

trust n surveillance 0.00006091354234545010000

trust n reliability 0.15767470436120000000000

trust n interest 0.00018274062703635200000

trust n office 0.03044694640783100000000

trust n combination 0.00006091354234545010000

trust n confidence 0.00096017837094889300000

trust n care 0.61042440072755100000000

trust n guidance 0.00012182708469090100000

trust n charge 0.02682051998676900000000

trust n opinion 0.05558181581544910000000

trust n benefit 0.01186986224720160000000

trust n expectation 0.01618365157486440000000

trust n control 0.00981399516021818000000

preponderance n advantage 0.00043653035423910400000

preponderance n strength 0.01289161340510390000000

preponderance n power 0.00000753688107458423000

preponderance n prevalence 0.00265988414366618000000

preponderance n mass 0.02193081655082580000000

preponderance n weight 0.00713256500547659000000

preponderance n control 0.02936498891185710000000

preponderance n predominance 0.86446363006212500000000

preponderance n influence 0.00283381881535516000000

preponderance n bulk 0.02222704595328850000000

preponderance n majority 0.03605156991698780000000

disputable a controversial 1.00000000000000000000000

historic a critical 0.00109949654020528000000

historic a unusual 0.00358624848074770000000

historic a particular 0.00936815929664705000000

historic a noteworthy 0.00791447940578803000000

historic a salient 0.00088107448279406100000

historic a special 0.00078468342826616300000

historic a significant 0.01767249216714110000000

historic a historical 0.95869336619841100000000

flatten v depress 1.00000000000000000000000

delete v obliterate 0.00040730441690696000000

delete v remove 0.00686329691850518000000

delete v eliminate 0.82548903671492400000000

delete v excise 0.07314218066948220000000

delete v drop 0.00013576813896898700000

delete v eradicate 0.07264246651971730000000

delete v exclude 0.02067001153072470000000

delete v destroy 0.00024263067386396200000

delete v omit 0.00040730441690696000000

advantage n position 0.00047030563751325900000

advantage n protection 0.02851724641169280000000

advantage n value 0.07923402546599090000000

advantage n aid 0.00037084012559893100000

advantage n recognition 0.00182505706332875000000

advantage n accommodation 0.00001607205529994930000

advantage n lead 0.00073672321465304800000

advantage n utility 0.02728942569719750000000

advantage n reward 0.00001835023281113200000

advantage n power 0.00208918117754968000000

advantage n satisfaction 0.00720249560004298000000

advantage n interest 0.00389765105176991000000

advantage n right 0.00067138096183153400000

advantage n improvement 0.09685573629597670000000

advantage n worth 0.00004301928780362790000

advantage n return 0.00001266343445733020000

advantage n start 0.00009352255503170470000

advantage n acquisition 0.00036780058225941000000

advantage n preference 0.00024842107217457400000

advantage n enjoyment 0.00000128415784488438000

advantage n prevalence 0.00285296845606906000000

advantage n odds 0.00009553279213324340000

advantage n account 0.02004921077596760000000

advantage n gain 0.00124181419614503000000

advantage n jump 0.00002589510527985360000

advantage n drop 0.00098845206004850400000

advantage n benefit 0.47285222764382800000000

advantage n use 0.15900198726160100000000

advantage n edge 0.00024614245222880700000

advantage n assistance 0.00008249922851259310000

advantage n approval 0.00004743413077046980000

advantage n favor 0.00027058508560542700000

advantage n advancement 0.00015097589941364000000

advantage n compensation 0.00136753540874068000000

advantage n superiority 0.01162708254595070000000

advantage n convenience 0.00005365049111142100000

advantage n ground 0.00099474662603030000000

advantage n comfort 0.00017488367668605200000

advantage n service 0.01333682760076410000000

advantage n influence 0.01256099838132630000000

advantage n avail 0.00014277342458121700000

advantage n support 0.00943363251017788000000

advantage n success 0.02071574484167010000000

advantage n choice 0.02172519732452900000000

commit v remove 0.01385041551246540000000

commit v hospitalize 0.00692520775623268000000

commit v discharge 0.36807479224376700000000

commit v warrant 0.01385041551246540000000

commit v transmit 0.00692520775623268000000

commit v perform 0.59037396121883700000000

car n automobile 1.00000000000000000000000

alert a prudent 0.00462869977466273000000

alert a cautious 0.07174484650727230000000

alert a alive 0.00299569139541701000000

alert a aware 0.91905724055990700000000

alert a awake 0.00102690231775904000000

alert a rapid 0.00054147053967752700000

alert a active 0.00000514890530409588000

thalassemia n thalassaemia 1.00000000000000000000000

terminology n nomenclature 0.88057679073464500000000

terminology n technology 0.11942320926535500000000

complex a mosaic 0.00183375424882064000000

complex a multifaceted 0.00359050540842735000000

complex a deep 0.00034193593845372400000

complex a mixed 0.04990962991574430000000

complex a compact 0.00036497519089317000000

complex a enigmatic 0.00010995876708645800000

complex a multiform 0.00644541639738457000000

complex a perplexing 0.00267090517986551000000

complex a heterogeneous 0.16320594217512300000000

complex a multiple 0.14541503932668500000000

complex a irreducible 0.06585534145153800000000

complex a composite 0.04836578073369180000000

complex a complicated 0.11727443643470600000000

complex a paradoxical 0.00006225450480410080000

complex a obscure 0.00224624047114340000000

complex a miscellaneous 0.00141410809102381000000

complex a difficult 0.39040300801861600000000

complex a knotty 0.00049076774599374900000

rapid eye movement sleep n rapid eye movement 0.33685344963128100000000

rapid eye movement sleep n rem sleep 0.66314655036871900000000

proton magnetic resonance n nuclear magnetic resonance 1.00000000000000000000000

purge v remove 0.00154099585376767000000

purge v eliminate 0.00231149378065151000000

purge v retch 0.00109196394935855000000

purge v purify 0.00077049792688383700000

purge v treat 0.99186997121255600000000

purge v empty 0.00023114937806515100000

purge v vomit 0.00218392789871710000000

iud n intrauterine device 1.00000000000000000000000

increase n overstatement 0.00000840526593645925000

increase n reinforcement 0.00001232721741943630000

increase n advantage 0.00412922219764191000000

increase n intensification 0.00026557100946338600000

increase n enhancement 0.01194914730935410000000

increase n spread 0.00002994618068141050000

increase n propagation 0.00000257844682578640000

increase n rise 0.11604947958276800000000

increase n aggravation 0.00022959253516920000000

increase n accrual 0.00064158478208850200000

increase n generation 0.00351659743414251000000

increase n accumulation 0.00642051819500135000000

increase n accretion 0.00033797266835215400000

increase n access 0.00014029976213518300000

increase n advance 0.00463297124461943000000

increase n extension 0.00059538056552164300000

increase n elaboration 0.00061329708628129400000

increase n supplement 0.00039434584742620600000

increase n dilatation 0.00181407004744397000000

increase n exacerbation 0.00260610434401031000000

increase n multiplication 0.00000662908545275847000

increase n development 0.00986686057695372000000

increase n dilation 0.00354997030566054000000

increase n expansion 0.00269104356281705000000

increase n improvement 0.21295059045821800000000

increase n profit 0.00001885789526312750000

increase n return 0.00274227274497644000000

increase n thickening 0.00029402618559194500000

increase n yield 0.00159705292541149000000

increase n enlargement 0.00223643549927375000000

increase n augmentation 0.00658976324290899000000

increase n addition 0.03990441426068990000000

increase n swelling 0.00009414700398064850000

increase n exaggeration 0.00002509191022614490000

increase n prolongation 0.01373181681145050000000

increase n maturation 0.00029023297864388400000

increase n increment 0.01910802528456980000000

increase n amplification 0.00025655912946348400000

increase n cumulation 0.00001154418683408130000

increase n gain 0.00794945301620419000000

increase n jump 0.00001263295896267760000

increase n incorporation 0.00202408075824225000000

increase n maximization 0.00004768776655544790000

increase n build-up 0.00006196165586455880000

increase n inflation 0.00017778532373962700000

increase n acceleration 0.00290470589923367000000

increase n accession 0.00000168767156051327000

increase n progression 0.00672224300558472000000

increase n growth 0.00777274286373508000000

increase n upsurge 0.00155554354568618000000

increase n graft 0.00166043183707567000000

increase n advancement 0.00004904312384219320000

increase n distension 0.00002667335392685560000

increase n concentration 0.13387873835759900000000

increase n surge 0.00004187001988154850000

increase n lengthening 0.00457436140488879000000

increase n escalation 0.00404890122439861000000

increase n elevation 0.07531674224904000000000

increase n harvest 0.00000828601155543558000

increase n result 0.27433568864231100000000

increase n product 0.00647121909079052000000

increase n magnification 0.00000277444865319497000

category n position 0.00057851817416003400000

category n variety 0.00420354027091345000000

category n type 0.06840706117474520000000

category n designation 0.00061123150798422500000

category n level 0.04635971497169910000000

category n genus 0.00000454278341125398000

category n section 0.00132423696930886000000

category n concept 0.00164836552787234000000

category n specification 0.00020035415839055100000

category n species 0.00975248830673486000000

category n department 0.00412659693307267000000

category n status 0.00858330913759330000000

category n set 0.00507156393719827000000

category n bracket 0.00110342500500464000000

category n disposition 0.00014205135932616500000

category n division 0.00017429183138630000000

category n group 0.69706148743203700000000

category n form 0.01635846724743720000000

category n family 0.00967330920904957000000

category n grade 0.03053786895271370000000

category n rank 0.00106301131823344000000

category n kind 0.00001056241030032290000

category n class 0.04458464228983670000000

category n province 0.00027761156990131900000

category n denomination 0.00006829402323846090000

category n coordination 0.00000529800688803576000

category n order 0.00098615606612159200000

category n grouping 0.00271421445997810000000

category n arrangement 0.00001061165812472610000

category n classification 0.04435717330733840000000

drowsiness n somnolence 0.97712989329241600000000

drowsiness n sleepiness 0.02287010670758420000000

bread n meal 1.00000000000000000000000

social class n class 0.46996645357301000000000

social class n stratum 0.53003354642699000000000

scopolamine n hyoscine 1.00000000000000000000000

saline n saline solution 1.00000000000000000000000

corrode v impair 1.00000000000000000000000

hematological a hematologic 0.98293975023037400000000

hematological a haematological 0.01706024976962570000000

estriol n oestriol 1.00000000000000000000000

discourage v suppress 0.11400504210858400000000

discourage v impede 0.00019771795596431400000

discourage v distress 0.00094109043120745600000

discourage v deprecate 0.00063663547064046100000

discourage v disappoint 0.03562579997056050000000

discourage v stop 0.14096214196791800000000

discourage v divert 0.00094997401248024800000

discourage v repress 0.00094997401248024800000

discourage v bully 0.00019771795596431400000

discourage v prevent 0.06250407970401410000000

discourage v weigh 0.10054774133083900000000

discourage v curb 0.01667565870199010000000

discourage v depress 0.04689025812339840000000

discourage v blunt 0.00122978933853341000000

discourage v switch 0.00206295585872661000000

discourage v obstruct 0.00250306027981434000000

discourage v inhibit 0.06014173417772180000000

discourage v shift 0.02983298972674000000000

discourage v try 0.14535901115382800000000

discourage v confuse 0.02273454634114920000000

discourage v obviate 0.00094997401248024800000

discourage v control 0.13383503837783200000000

discourage v withhold 0.03819953468660110000000

discourage v scare 0.01272519416452070000000

discourage v check 0.02648245971161050000000

discourage v avert 0.00285988042440163000000

markedly r distinctly 0.03045272184889560000000

markedly r obviously 0.00206692682232323000000

markedly r clearly 0.06151445347203250000000

markedly r greatly 0.49146545284376300000000

markedly r considerably 0.28022661680192400000000

markedly r notably 0.00823507160262466000000

markedly r remarkably 0.03576309082699140000000

markedly r particularly 0.00832249330145089000000

markedly r strikingly 0.02023805024198510000000

markedly r appreciably 0.06171512223800950000000

resist v avoid 0.21699739850458000000000

resist v confront 0.00240763449069010000000

resist v remain 0.00468594625371314000000

resist v obtain 0.07042713387248020000000

resist v restrain 0.00240763449069010000000

resist v prohibit 0.00128641648086009000000

resist v encounter 0.01050787431482230000000

resist v stop 0.00454312491251978000000

resist v restrict 0.00568164422564365000000

resist v prevent 0.01472413524388840000000

resist v curb 0.00064320824043004400000

resist v hold 0.00064320824043004400000

resist v overcome 0.00545847722181024000000

resist v thwart 0.00240763449069010000000

resist v inhibit 0.00305084273112014000000

resist v continue 0.59833107805769100000000

resist v proscribe 0.00064320824043004400000

resist v face 0.00985089772970492000000

resist v quit 0.00985089772970492000000

resist v maintain 0.01446339258783090000000

resist v cross 0.00985089772970492000000

resist v check 0.00128641648086009000000

resist v stand 0.00985089772970492000000

e n vitamin e 0.99603202709613900000000

e n east 0.00396797290386146000000

hg n mercury 1.00000000000000000000000

hemopoietic a haemopoietic 0.02269910638575440000000

hemopoietic a hematopoietic 0.97730089361424600000000

fragile a delicate 0.99979358034884900000000

fragile a slight 0.00020641965115078900000

final a certain 0.02940952900217240000000

final a inevitable 0.00041739728064706300000

final a absolute 0.00158647729943186000000

final a definitive 0.54673563419096700000000

final a binding 0.00027296755191873400000

final a definite 0.15977561921231100000000

final a latter 0.01735886098785610000000

final a fixed 0.01857035459348660000000

final a net 0.00268682444936460000000

final a terminal 0.00798856507417444000000

final a thorough 0.00988734393985809000000

final a extreme 0.00220249499784534000000

final a complete 0.08210016654862410000000

final a ultimate 0.07732286366420360000000

final a eventual 0.04047877659054930000000

final a conclusive 0.00320612461658976000000

descriptor n form 1.00000000000000000000000

concentrated a intensive 0.33333333333333300000000

concentrated a concerted 0.66666666666666700000000

t cell n t lymphocyte 1.00000000000000000000000

nomenclature n terminology 0.15120436524987000000000

nomenclature n classification 0.84879563475013000000000

dealing n transaction 1.00000000000000000000000

rapidly r directly 0.05593570593254500000000

rapidly r abruptly 0.00132519434605018000000

rapidly r expeditiously 0.01685892130651490000000

rapidly r fast 0.07429014552852720000000

rapidly r promptly 0.24830259930655500000000

rapidly r quickly 0.53094298678680000000000

rapidly r swiftly 0.00012004813723917200000

rapidly r suddenly 0.07222439865576820000000

son n boy 0.99463578074477500000000

son n young man 0.00081677324487723200000

son n offspring 0.00454744601034752000000

january n jan 1.00000000000000000000000

lupus erythematosus n le 1.00000000000000000000000

generator n author 1.00000000000000000000000

sleep n lethargy 0.00986907743317216000000

sleep n rest 0.05325435496028110000000

sleep n dream 0.06028800702924820000000

sleep n death 0.32498148867676300000000

sleep n nap 0.08146487850157560000000

sleep n stupor 0.00110708021555684000000

sleep n latency 0.46776751477195000000000

sleep n coma 0.00123181732530949000000

sleep n blackout 0.00003578108614314830000

boast v have 1.00000000000000000000000

blossom v grow 0.27828152841021600000000

blossom v progress 0.02981587804395170000000

blossom v thrive 0.00993862601465056000000

blossom v evolve 0.62613343892298500000000

blossom v develop 0.05583052860819640000000

repletion n excess 0.99896667299616300000000

repletion n overdose 0.00103332700383744000000

prudent a advisable 0.25389931709591100000000

prudent a alert 0.01138649730393040000000

prudent a particular 0.00775979489349587000000

prudent a careful 0.06048354816105650000000

prudent a sensible 0.00001213912292529860000

prudent a reasonable 0.55314504479251300000000

prudent a suspicious 0.00171494448909619000000

prudent a rational 0.02447708336204350000000

prudent a moderate 0.00097240059620342500000

prudent a sound 0.00001213912292529860000

prudent a judicious 0.08613709105989950000000

distinguish v divide 0.01490553411078800000000

distinguish v observe 0.02257698336735670000000

distinguish v differentiate 0.41838850257740100000000

distinguish v segregate 0.00002957435890183340000

distinguish v discern 0.00132498756399434000000

distinguish v denote 0.00380030511888559000000

distinguish v label 0.00000147156054942326000

distinguish v detect 0.05867050880398170000000

distinguish v indicate 0.00118039850926054000000

distinguish v grade 0.00020773362050350700000

distinguish v separate 0.06828925672222980000000

distinguish v see 0.01239322317202490000000

distinguish v perceive 0.00000291151496595523000

distinguish v collate 0.00000224438512853127000

distinguish v rate 0.00390931979843939000000

distinguish v describe 0.01200768535375150000000

distinguish v ascertain 0.00161166693435245000000

distinguish v categorize 0.00527427815346212000000

distinguish v discover 0.00018788020058386900000

distinguish v define 0.02467140740474730000000

distinguish v judge 0.00022576471666315600000

distinguish v mark 0.00004886197241159710000

distinguish v discriminate 0.10292456734113000000000

distinguish v select 0.00480565746337653000000

distinguish v individualize 0.00050341333495993200000

distinguish v fix 0.00002635852208469380000

distinguish v designate 0.00002698613975496520000

distinguish v decide 0.00006354739280657840000

distinguish v honor 0.00000049052018314108200

distinguish v specify 0.00009547735843295850000

distinguish v determine 0.05957484379719390000000

distinguish v note 0.00238373031585128000000

distinguish v detail 0.00009079204432160950000

distinguish v know 0.01704991689850770000000

distinguish v commend 0.00000224438512853127000

distinguish v characterize 0.01141530254843120000000

distinguish v recognise 0.00006950713821358150000

distinguish v group 0.00090734698468345500000

distinguish v tell 0.00384650125369195000000

distinguish v recognize 0.00959578423983334000000

distinguish v identify 0.12122076735933300000000

distinguish v notice 0.00008785468660604350000

distinguish v classify 0.01559841035509250000000

bestow v yield 0.20000000000000000000000

bestow v confer 0.20000000000000000000000

bestow v offer 0.40000000000000000000000

bestow v add 0.20000000000000000000000

extradural a epidural 1.00000000000000000000000

chart v tabulate 0.00290713265085163000000

chart v describe 0.05924545204830860000000

chart v plan 0.00036606094184398000000

chart v draw 0.00035433260847935000000

chart v delineate 0.00139990892189282000000

chart v outline 0.00258246465068690000000

chart v register 0.00059810099010650800000

chart v record 0.66851764788566300000000

chart v detail 0.00076430550199000600000

chart v map 0.00054849585977988100000

chart v represent 0.00038651622678139300000

chart v document 0.23304301571290200000000

chart v follow 0.02477872530783230000000

chart v plot 0.00417546406023471000000

chart v trace 0.00033237663264601500000

localize v localise 0.16003191718222300000000

localize v place 0.00182192850363115000000

localize v differentiate 0.04365265352118780000000

localize v constrain 0.00000503426619502400000

localize v set 0.00489750785990607000000

localize v restrict 0.07324575311605300000000

localize v confine 0.10401851950205500000000

localize v contain 0.03691156974444940000000

localize v individualize 0.00054802777919015200000

localize v specify 0.00006578255740449990000

localize v encompass 0.00003487050796067430000

localize v limit 0.02853950567323890000000

localize v identify 0.50963033168177800000000

localize v circumscribe 0.03659659810472780000000

bacteriaemia n bacteremia 1.00000000000000000000000

achievable a obtainable 0.00045627509658308400000

achievable a manageable 0.00058059206582220400000

achievable a attainable 0.00072699943727051200000

achievable a feasible 0.08304310924751850000000

achievable a probable 0.00115802444693245000000

achievable a practicable 0.42580815385177400000000

achievable a possible 0.47787436164475300000000

achievable a reasonable 0.00092492251810137600000

achievable a potential 0.00810617112852716000000

achievable a accessible 0.00132139056271735000000

self-esteem n confidence 0.00653337437886700000000

self-esteem n independence 0.34620806650433400000000

self-esteem n self-sufficiency 0.64725855911679900000000

subsequently r afterward 0.00323731495813435000000

subsequently r afterwards 0.00386040578842518000000

subsequently r finally 0.07257160734360360000000

subsequently r thereafter 0.05794104997461830000000

subsequently r eventually 0.43508052280151700000000

subsequently r consequently 0.00673614451695625000000

subsequently r next 0.00697751267762149000000

subsequently r later 0.41359544193912400000000

roll v follow 1.00000000000000000000000

navigation n exploration 1.00000000000000000000000

ascendancy n control 1.00000000000000000000000

divergent a disparate 0.12143249401890700000000

divergent a contradictory 0.07966316096985010000000

divergent a different 0.25605230306324600000000

divergent a mixed 0.06638596747487510000000

divergent a diverse 0.02845112891780360000000

divergent a several 0.03410916963741450000000

divergent a various 0.34852632924309400000000

divergent a opposite 0.06061327465097290000000

divergent a contrary 0.00476617202383719000000

excellent a paramount 0.00020037014240284100000

excellent a refined 0.00001541286916091950000

excellent a noteworthy 0.00007539877542430870000

excellent a invaluable 0.00001907544335156720000

excellent a exceptional 0.00079989982349999100000

excellent a unique 0.02687787049080140000000

excellent a bad 0.04952925453134040000000

excellent a good 0.78181082122104200000000

excellent a valuable 0.04920961237257790000000

excellent a a-1 0.00003058378158883360000

excellent a first 0.00963784320459823000000

excellent a extraordinary 0.00010151234516329700000

excellent a outstanding 0.00461103998077310000000

excellent a exquisite 0.00001656067993690010000

excellent a classic 0.00008033106471603850000

excellent a frontline 0.00002839734414535970000

excellent a high-grade 0.00008926325083648900000

excellent a superb 0.00372255593489149000000

excellent a distinctive 0.00038064191473862400000

excellent a striking 0.00055805354811125000000

excellent a accomplished 0.00001251522063657090000

excellent a sharp 0.00008304191862096660000

excellent a fine 0.00002792592578405330000

excellent a rare 0.00905599332912701000000

excellent a remarkable 0.00399246064064805000000

excellent a desirable 0.00079492681926106500000

excellent a high-quality 0.00052737144250404400000

excellent a attractive 0.00010151234516329700000

excellent a notable 0.00085195932455164900000

excellent a prime 0.00110394675365086000000

excellent a great 0.01635633653898830000000

excellent a superior 0.03922586498617220000000

excellent a commendable 0.00001714868743535310000

excellent a competent 0.00005449734835568770000

simplicity n severity 0.01261106334193180000000

simplicity n ease 0.98738893665806800000000

malaise n worry 0.00663895282438204000000

malaise n anxiety 0.00056013814882951500000

malaise n pain 0.19029091421621100000000

malaise n weakness 0.76839951305771700000000

malaise n depression 0.03411048175285940000000

goat n score 1.00000000000000000000000

thickness n density 0.01773196597851270000000

thickness n girth 0.00005659353099263130000

thickness n measure 0.02134229120702890000000

thickness n width 0.21269939372640000000000

thickness n stiffness 0.02381387185950540000000

thickness n weight 0.00307907423385515000000

thickness n concentration 0.01143743643632150000000

thickness n diameter 0.67945685218611700000000

thickness n bulk 0.00312443452355152000000

thickness n body 0.02725808631771540000000

direct current n dc 1.00000000000000000000000

build v compile 0.00001234622300520600000

build v start 0.00199482071687675000000

build v raise 0.00027312577817722400000

build v begin 0.00005483660778058650000

build v form 0.00586531243864463000000

build v base 0.00761204042662824000000

build v block 0.00002469244601041200000

build v extend 0.00009683140248161780000

build v create 0.06231741714654570000000

build v accelerate 0.00001103194177284900000

build v expand 0.00011049190671918100000

build v progress 0.00001103194177284900000

build v fabricate 0.00013330837043266100000

build v produce 0.00371804281113327000000

build v make 0.01053950679390970000000

build v evolve 0.00014494487718001800000

build v enlarge 0.00176181378151684000000

build v model 0.01080679031524720000000

build v construct 0.17703432652833700000000

build v elevate 0.00018803860021278300000

build v wax 0.00001234622300520600000

build v amplify 0.00001103194177284900000

build v establish 0.01890405281557810000000

build v institute 0.00081035350579656100000

build v synthesize 0.00002337816477805500000

build v found 0.00002206388354569810000

build v reconstruct 0.00006586854955343560000

build v increase 0.00316446772912123000000

build v devise 0.01390589898470180000000

build v formulate 0.00306129981967808000000

build v strengthen 0.00003309582531854710000

build v heighten 0.00001103194177284900000

build v mount 0.00347531821523287000000

build v develop 0.67374573226982700000000

build v engineer 0.00003227713416053920000

build v boost 0.00001103194177284900000

brain-stem n brain stem 1.00000000000000000000000

orient v arrange 0.00171917237544920000000

orient v place 0.00377254719060798000000

orient v set 0.03174328993240140000000

orient v train 0.31743289932401400000000

orient v relate 0.01029909754469570000000

orient v determine 0.41271540733084600000000

orient v design 0.09875690201191560000000

orient v reassess 0.00377254719060798000000

orient v assess 0.11978813709946200000000

optic disc n optic disk 1.00000000000000000000000

anhydrosis n anhidrosis 1.00000000000000000000000

hemophilia a n classical haemophilia 0.18759059508920000000000

hemophilia a n haemophilia a 0.78038174233459600000000

hemophilia a n classical hemophilia 0.03202766257620480000000

exist v be 0.76722568447590800000000

exist v survive 0.00459238476489129000000

exist v manage 0.00134681070605019000000

exist v ensue 0.00011939510709827200000

exist v result 0.00202418421235745000000

exist v happen 0.00393108064504950000000

exist v remain 0.05061182184298640000000

exist v obtain 0.01195279798674900000000

exist v prevail 0.01262425597347210000000

exist v last 0.00078901599834200400000

exist v continue 0.03515854867698530000000

exist v move 0.00018948018708538000000

exist v lie 0.00041166052087408900000

exist v occur 0.10822275000242400000000

exist v stand 0.00080012889972710800000

comprehension n recognition 0.73337356561645500000000

comprehension n field 0.00226699711164283000000

comprehension n measure 0.00113349855582141000000

comprehension n insight 0.00023680128047027800000

comprehension n extent 0.00902022253109924000000

comprehension n knowledge 0.25396891490451100000000

altercation n combat 1.00000000000000000000000

clomiphene n clomiphene citrate 0.14871243929263300000000

clomiphene n clomid 0.85128756070736700000000

casualty n death 0.00420437606163652000000

casualty n victim 0.91413789237773200000000

casualty n loss 0.08092234858457350000000

casualty n hazard 0.00073538297605799400000

admirable a rare 1.00000000000000000000000

videotape v tape 1.00000000000000000000000

vial n vessel 1.00000000000000000000000

adjust v localize 0.00167179316386733000000

adjust v measure 0.07812901408953820000000

adjust v standardize 0.08808197684494660000000

adjust v arrange 0.00002617500593662390000

adjust v suit 0.00004984373139051800000

adjust v resolve 0.00397492683837018000000

adjust v correct 0.32563298382609200000000

adjust v remodel 0.00000297453640458395000

adjust v accommodate 0.00001023707382459030000

adjust v customize 0.00076603805619328000000

adjust v settle 0.00001819575539869680000

adjust v regulate 0.03601018844840830000000

adjust v focus 0.01038597016527030000000

adjust v rectify 0.00001023707382459030000

adjust v grade 0.00249888542362029000000

adjust v allocate 0.00022827870732634700000

adjust v prepare 0.01206047489725720000000

adjust v set 0.06376940015227460000000

adjust v collate 0.00000531578157996221000

adjust v reconcile 0.00003436542259173840000

adjust v tighten 0.00000247635935353760000

adjust v clarify 0.00317714763819928000000

adjust v heal 0.00003131678500993540000

adjust v agree 0.00004824737675413810000

adjust v repair 0.00044563332990498800000

adjust v stabilize 0.00074472394729664900000

adjust v change 0.01515642751980010000000

adjust v compromise 0.00011368680939978100000

adjust v conclude 0.04172904621391980000000

adjust v satisfy 0.00008708768233144470000

adjust v fix 0.01340408462316210000000

adjust v fit 0.00000531578157996221000

adjust v order 0.00009640541070742020000

adjust v tailor 0.12626728797104500000000

adjust v adapt 0.02318195556724350000000

adjust v organize 0.01253073036037730000000

adjust v redress 0.00001593793211203370000

adjust v readjust 0.00000247635935353760000

adjust v coordinate 0.00002125371369199590000

adjust v modify 0.08458436338393240000000

adjust v balance 0.00140904604537261000000

adjust v alter 0.01023450380298820000000

adjust v classify 0.01815838841541740000000

adjust v improve 0.02516676768546470000000

adjust v calibrate 0.00001841429146557130000

sequence n placement 0.00008145365387443660000

sequence n categorization 0.00006435218141302840000

sequence n section 0.00504416799671942000000

sequence n string 0.00003309714460203370000

sequence n extract 0.00023926346284185300000

sequence n pattern 0.07033876246638750000000

sequence n chain 0.00706297696486560000000

sequence n chronology 0.00166825369339298000000

sequence n flow 0.00051717182488157800000

sequence n course 0.03235822794924880000000

sequence n episode 0.00665164007734389000000

sequence n array 0.00010652205206506900000

sequence n series 0.01733094508408100000000

sequence n progression 0.00140595687357221000000

sequence n continuity 0.00001206946645556930000

sequence n train 0.00876630488247549000000

sequence n continuation 0.00013684893578614300000

sequence n file 0.00026840571848536000000

sequence n rank 0.00036475876343186600000

sequence n distribution 0.02326731042521730000000

sequence n clip 0.00008989524553878500000

sequence n order 0.81950290185204000000000

sequence n line 0.00252232714414965000000

sequence n arrangement 0.00000120480526725537000

sequence n classification 0.00216518133586337000000

all n total 0.97775696003162300000000

all n mass 0.02224303996837700000000

symmetrical a regular 0.01718240082905920000000

symmetrical a parallel 0.01718240082905920000000

symmetrical a uniform 0.20983425158514700000000

symmetrical a consistent 0.58425095928580000000000

symmetrical a equal 0.01008416407729370000000

symmetrical a symmetric 0.16146582339364100000000

predilection n propensity 1.00000000000000000000000

people n nation 0.00356600610059716000000

people n generation 0.00104293858903380000000

people n mortality 0.03770851629383750000000

people n population 0.64029232813576300000000

people n race 0.00150721400353543000000

people n society 0.00529404449891620000000

people n world 0.02067935477811940000000

people n mass 0.00060738526497740300000

people n group 0.18978529452853100000000

people n family 0.04397430541061100000000

people n state 0.01182674823908740000000

people n community 0.04371586415699060000000

gd n gadolinium 1.00000000000000000000000

ensuing a next 0.24106732699979600000000

ensuing a subsequent 0.75893267300020400000000

engagement n period 0.00124318945599625000000

engagement n agreement 0.00206658282581356000000

engagement n involvement 0.99504344097855500000000

engagement n action 0.00082339336981730600000

engagement n assault 0.00082339336981730600000

compelling a convincing 0.46564834507713200000000

compelling a powerful 0.02366475428816150000000

compelling a persuasive 0.02208919922564230000000

compelling a cogent 0.06827172369183470000000

compelling a strong 0.42032597771722900000000

function n capacity 0.08445428199773250000000

function n position 0.01374763842192060000000

function n profession 0.00008407201987119410000

function n work 0.01715114675385330000000

function n procedure 0.03313838882262360000000

function n thing 0.00000254466370395771000

function n concern 0.00169198417985831000000

function n administration 0.00425399751051385000000

function n power 0.00970955993611799000000

function n sphere 0.00369968655918396000000

function n role 0.09023019149416090000000

function n interest 0.00654790378409626000000

function n task 0.01482909432786820000000

function n situation 0.00339563372266632000000

function n office 0.00965712873932778000000

function n purpose 0.05279464045553660000000

function n realm 0.00001620892249368710000

function n mapping 0.00145328084592862000000

function n occasion 0.00018351873306436900000

function n exercise 0.02034399874459120000000

function n charge 0.00028050632558550400000

function n operation 0.05969378524042670000000

function n use 0.04996583138609250000000

function n map 0.00040697662751655600000

function n part 0.00939505291843339000000

function n assignment 0.00053124501028274000000

function n faculty 0.00007120667164935690000

function n matter 0.00344715165585648000000

function n employment 0.00081878792864166900000

function n job 0.00012994401064413000000

function n responsibility 0.00000127233185197883000

function n scope 0.00000234920803775492000

function n occupation 0.00009180386221013660000

function n service 0.00278425433373222000000

function n station 0.00000694125058008280000

function n character 0.00000366637411643688000

function n discharge 0.00606064330012184000000

function n line 0.01843774812803130000000

function n activity 0.48026078825123500000000

function n duty 0.00022514454984083000000

university n college 0.54310760124357000000000

university n education 0.45689239875643000000000

purely r fully 0.50000000000000000000000

purely r solely 0.50000000000000000000000

ingredient n factor 0.02226385069316510000000

ingredient n agent 0.79327318390584500000000

ingredient n element 0.17879918775955000000000

ingredient n feature 0.00459184006494301000000

ingredient n part 0.00053596878824805100000

ingredient n unit 0.00053596878824805100000

heart block n atrioventricular block 1.00000000000000000000000

serial a sequential 0.78879293396617900000000

serial a ongoing 0.00705865860802765000000

serial a consecutive 0.20352624520452000000000

serial a successive 0.00062216222127253400000

hasten v assist 0.00006717201232146640000

hasten v facilitate 0.00697581347958448000000

hasten v accelerate 0.98330766655388800000000

hasten v arouse 0.00006717201232146640000

hasten v advance 0.00013434402464293500000

hasten v prompt 0.00006717201232146640000

hasten v spin 0.00006717201232146640000

hasten v brush 0.00006717201232146640000

hasten v precipitate 0.00013434402464293500000

hasten v provoke 0.00013434402464293500000

hasten v stimulate 0.00047020408625027700000

hasten v express 0.00073889213553615000000

hasten v pace 0.00013434402464293500000

hasten v induce 0.00736549953527573000000

hasten v help 0.00006717201232146640000

hasten v tear 0.00006717201232146640000

hasten v boost 0.00013434402464293500000

ejection n exclusion 0.02022067279573640000000

ejection n discharge 0.97977932720426400000000

underestimation n underestimate 1.00000000000000000000000

si n ti 1.00000000000000000000000

periodically r intermittently 0.00233112377515215000000

periodically r weekly 0.36311428605640800000000

periodically r rarely 0.00061690686462121500000

periodically r alternately 0.00041423986073746300000

periodically r monthly 0.08246968957558350000000

periodically r occasionally 0.00033539542043429100000

periodically r cyclically 0.00013727514661950900000

periodically r systematically 0.00303727653076324000000

periodically r regularly 0.24837966495232600000000

periodically r infrequently 0.00120992776747186000000

periodically r annually 0.06559051307546180000000

periodically r biannually 0.00010425876496942200000

periodically r frequently 0.06776423145648290000000

periodically r repeatedly 0.14542616556234700000000

periodically r daily 0.01906904519062200000000

unusual a scarce 0.00107067563372028000000

unusual a peculiar 0.00066208178538156700000

unusual a atypical 0.07553666166529620000000

unusual a particular 0.01270839928094120000000

unusual a noteworthy 0.00389012555716325000000

unusual a new 0.07335422345903250000000

unusual a exceptional 0.00426338109271315000000

unusual a unique 0.10846052657797600000000

unusual a deep 0.00155990509585979000000

unusual a uncommon 0.08120693713662150000000

unusual a radical 0.00001783716025194550000

unusual a bizarre 0.00106705045152323000000

unusual a different 0.03458319501432170000000

unusual a conspicuous 0.00007263760587243400000

unusual a unfamiliar 0.00000100479396995005000

unusual a special 0.00729052947749598000000

unusual a surprising 0.00294226092750398000000

unusual a original 0.00294714795053438000000

unusual a disturbing 0.00262585044995055000000

unusual a uncomfortable 0.00012369066602297000000

unusual a extraordinary 0.00000708889737334209000

unusual a odd 0.00304169974590817000000

unusual a unprecedented 0.00005937423224244340000

unusual a outstanding 0.00015372481673208100000

unusual a important 0.03425674921269560000000

unusual a prominent 0.00644459869250969000000

unusual a perplexing 0.00049067298270140700000

unusual a unclassifiable 0.00014658931725009100000

unusual a profound 0.00008268033124225290000

unusual a singular 0.00074302690809667000000

unusual a exotic 0.00000375953618652226000

unusual a abnormal 0.03641720155006030000000

unusual a irrational 0.00000143709422514486000

unusual a significant 0.03796796181190910000000

unusual a unexpected 0.00892523623176725000000

unusual a fresh 0.00121667989836327000000

unusual a infrequent 0.00852271921354202000000

unusual a late 0.02491767586407910000000

unusual a extreme 0.00152634746936899000000

unusual a distinctive 0.02470530753998570000000

unusual a irregular 0.00504375422025077000000

unusual a striking 0.00423645985505033000000

unusual a aberrant 0.00163910515166099000000

unusual a recent 0.00665895681210517000000

unusual a anomalous 0.00247372036844162000000

unusual a inconsistent 0.00082662311584966200000

unusual a rare 0.33623940640480600000000

unusual a remarkable 0.00269291077471903000000

unusual a notable 0.00291058656235207000000

unusual a advanced 0.00606869485641960000000

unusual a individual 0.00683012716860608000000

unusual a eccentric 0.00004275302717528320000

unusual a great 0.00262771809576452000000

unusual a superior 0.00353136340377695000000

unusual a phenomenal 0.00002134526137479420000

unusual a foreign 0.00001998652810453010000

unusual a novel 0.00900079346002607000000

unusual a marked 0.00512104180512503000000

infer v demonstrate 0.15515301049288700000000

infer v believe 0.06015302661687080000000

infer v generalise 0.00061268511448581900000

infer v indicate 0.00631792960101528000000

infer v hypothesize 0.05977264884058260000000

infer v understand 0.00366927041066846000000

infer v think 0.08626503818302770000000

infer v deduce 0.00162731047951002000000

infer v generalize 0.00133074266094536000000

infer v imagine 0.00010537243197372200000

infer v assume 0.05261467005944860000000

infer v presume 0.01066462147727490000000

infer v judge 0.04669494776121750000000

infer v show 0.03661119771010200000000

infer v mark 0.00111999779699792000000

infer v conclude 0.00188167674961775000000

infer v estimate 0.03056131180108840000000

infer v designate 0.00061268511448581900000

infer v reason 0.00083843833954816600000

infer v suspect 0.12857812963375900000000

infer v calculate 0.02301888663360960000000

infer v derive 0.00347609769977597000000

infer v suggest 0.27981073883577200000000

infer v speculate 0.00071210105604381100000

infer v induce 0.00286199721111603000000

infer v collect 0.00371525732934090000000

infer v evidence 0.00071973755256886200000

infer v tell 0.00039509997429190700000

infer v extrapolate 0.00010537243197372200000

hyperaldosteronism n aldosteronism 1.00000000000000000000000

cope v survive 0.00043721929278816300000

cope v manage 0.91684733885423500000000

cope v handle 0.00434843798833221000000

cope v address 0.07785691468972560000000

cope v discharge 0.00043721929278816300000

cope v tackle 0.00007286988213136040000

experiment n clinical trial 0.00857175124562327000000

experiment n test 0.02407980218803540000000

experiment n agreement 0.00049859001175546800000

experiment n dissection 0.00015621936021200100000

experiment n effort 0.00097151576902084600000

experiment n evaluation 0.00942443678212585000000

experiment n evidence 0.00292924797954030000000

experiment n scrutiny 0.00000881304696670540000

experiment n essay 0.00031216516845131500000

experiment n speculation 0.00000753963839368283000

experiment n attempt 0.00070419652528847400000

experiment n search 0.00209003857999451000000

experiment n probe 0.00114658686562146000000

experiment n measure 0.00239320588284855000000

experiment n assessment 0.00534593915829109000000

experiment n examination 0.00984549132909424000000

experiment n experimentation 0.00069114325410991100000

experiment n demonstration 0.00013876127546596200000

experiment n confirmation 0.00017001140385329300000

experiment n exercise 0.00410858898490662000000

experiment n corroboration 0.00000600606963423896000

experiment n operation 0.01304590206863390000000

experiment n pilot project 0.00002142105801878210000

experiment n assay 0.01500811500233610000000

experiment n practice 0.00192993398037504000000

experiment n substantiation 0.00000202695368512592000

experiment n research 0.00859370481260115000000

experiment n analysis 0.05112739297398140000000

experiment n investigation 0.02754783229707940000000

experiment n observation 0.01418744651471380000000

experiment n pilot 0.00013681110650744600000

experiment n study 0.74124748804084300000000

experiment n inspection 0.00001433816105674300000

experiment n appraisal 0.00000724468733954268000

experiment n inquiry 0.00013438200964532800000

experiment n proof 0.00000201890604209676000

experiment n trial 0.05331386039870600000000

experiment n verification 0.00005436527873779060000

experiment n check 0.00002566523046377770000

tag v identify 1.00000000000000000000000

vaccination n protection 0.01732210153471920000000

vaccination n immunization 0.77241972676827000000000

vaccination n injection 0.08897741884887420000000

vaccination n prevention 0.09343644974309780000000

vaccination n inoculation 0.02784430310503890000000

contrary n opposite 0.87500000000000000000000

contrary n converse 0.12500000000000000000000

weight v bias 1.00000000000000000000000

ohio n oh 1.00000000000000000000000

nervous a sensitive 0.98931739996378800000000

nervous a active 0.01068260003621220000000

geographical a geographic 1.00000000000000000000000

cusp n leaflet 0.99924173354155600000000

cusp n point 0.00075826645844428900000

sunlight n sunshine 0.00899265451548368000000

sunlight n sun 0.99100734548451600000000

reaction time n latency 1.00000000000000000000000

hydrops n edema 1.00000000000000000000000

everyday a usual 0.00070299941675403700000

everyday a customary 0.00060934940564115500000

everyday a regular 0.00492099591727826000000

everyday a common 0.08881267587219830000000

everyday a standard 0.00060934940564115500000

everyday a normal 0.00070299941675403700000

everyday a daily 0.60815840453012900000000

everyday a routine 0.28434459083236800000000

everyday a frequent 0.00762363811946600000000

everyday a habitual 0.00351499708377019000000

confident a certain 0.56271273181104700000000

confident a absolute 0.00282949135982827000000

confident a positive 0.32563640249063700000000

confident a secure 0.10882137433848800000000

cannulate v intubate 1.00000000000000000000000

allergic reaction n allergy 1.00000000000000000000000

shock v alarm 0.03443113772455090000000

shock v insult 0.96556886227544900000000

mean v undertake 0.04694751504191100000000

mean v propose 0.00041056127622858500000

mean v indicate 0.12644466908224500000000

mean v expect 0.01260638059672700000000

mean v think 0.27269611790685400000000

mean v aim 0.00044764901572529600000

mean v plan 0.00471856386515289000000

mean v imply 0.00367072192894743000000

mean v show 0.10742229254535400000000

mean v determine 0.05980932909006780000000

mean v suggest 0.00523964890580993000000

mean v intend 0.26968677840219700000000

mean v design 0.08969449170466580000000

mean v pursue 0.00020528063811429300000

obesity n fatness 0.01285671617299430000000

obesity n overweight 0.98703451251724200000000

obesity n bulk 0.00010877130976346000000

digestive tract n gastrointestinal tract 1.00000000000000000000000

computerise v computerize 1.00000000000000000000000

isolate v divide 0.03763069035136740000000

isolate v remove 0.12829549825518200000000

isolate v dissociate 0.01971008640994060000000

isolate v sequester 0.00302161395309080000000

isolate v separate 0.42475058745441700000000

isolate v blacklist 0.00023366082783319400000

isolate v insulate 0.00017870221843698700000

isolate v detach 0.00023366082783319400000

isolate v confine 0.00051433326913540100000

isolate v abstract 0.02783651038404020000000

isolate v exclude 0.35759465604872400000000

poverty n hunger 0.00982052778259896000000

poverty n depletion 0.00258784682540014000000

poverty n insufficiency 0.01279003683553630000000

poverty n absence 0.18848475336579800000000

poverty n need 0.00723268095719882000000

poverty n defect 0.04887069509967390000000

poverty n deficiency 0.00258784682540014000000

poverty n lack 0.66702659941118600000000

poverty n deficit 0.06059901289720700000000

odor n smell 0.41242937853107300000000

odor n quality 0.58757062146892700000000

use v manage 0.01515914178878010000000

use v handle 0.00125669258203589000000

use v exploit 0.01532839406274910000000

use v devote 0.00034276142961259000000

use v operate 0.00522569388414615000000

use v regulate 0.00020890084238935700000

use v lose 0.00144004768120799000000

use v employ 0.16543394990543200000000

use v practise 0.00135484672524207000000

use v give 0.07948698531240040000000

use v utilize 0.11189787607999900000000

use v consume 0.00148118779020219000000

use v exhaust 0.00050848743015171200000

use v expend 0.00076357120846360600000

use v relate 0.01430085885113550000000

use v utilise 0.00074861356192863400000

use v exercise 0.00806628485952326000000

use v work 0.00160188188549176000000

use v make 0.09169565453098200000000

use v practice 0.00319093853680690000000

use v exert 0.00015212415177012000000

use v misuse 0.00005316592681682340000

use v deplete 0.00023123687827173200000

use v accept 0.01531507467533170000000

use v fill 0.00110480459507486000000

use v spend 0.00036494706040160100000

use v treat 0.09917644398418030000000

use v bleed 0.00040076582386551700000

use v do 0.01561545098651430000000

use v take 0.08379237307578780000000

use v would 0.00651585263347837000000

use v drain 0.00051534768066604900000

use v swallow 0.00023038880416629900000

use v pass 0.00177375090991830000000

use v adopt 0.01739063596872820000000

use v run 0.00301792449298439000000

use v absorb 0.00146938051568867000000

use v abuse 0.00240283872136479000000

use v control 0.01568321712574570000000

use v waste 0.00009366540935613890000

use v milk 0.00000209366838139418000

use v put 0.00064218385052020300000

use v play 0.00002030417517263700000

use v apply 0.20897280534180800000000

use v occupy 0.00001412696059656270000

use v manipulate 0.00555632763472906000000

measure v disperse 0.00000736299089149418000

measure v correspond 0.00059503545885541300000

measure v adjust 0.00220987883364235000000

measure v divide 0.00116057850540756000000

measure v dispense 0.00000313571992312841000

measure v partition 0.00011750388573416000000

measure v beat 0.00050918350484802200000

measure v appraise 0.00040540897750894600000

measure v split 0.00006364793810600270000

measure v indicate 0.00390754101579303000000

measure v regulate 0.00103906244066585000000

measure v quantify 0.03096810350745340000000

measure v align 0.00050918350484802200000

measure v gauge 0.00001295985148013190000

measure v equate 0.00000576515400283638000

measure v grade 0.00344294015184342000000

measure v average 0.00100901115430524000000

measure v affirm 0.00003052926868201940000

measure v allocate 0.00003183509283983230000

measure v figure 0.00004628940953163840000

measure v attest 0.00000648641407449730000

measure v rate 0.00484287871889423000000

measure v value 0.00000360335343438740000

measure v count 0.00194934790477699000000

measure v cover 0.00032970594577746600000

measure v reveal 0.00280913495344922000000

measure v confirm 0.00495591107783025000000

measure v verify 0.00237421588470303000000

measure v weigh 0.00020521301705754600000

measure v survey 0.00182289708892722000000

measure v distribute 0.00003179512220458280000

measure v show 0.00932766626248813000000

measure v mark 0.00052432056432857600000

measure v hold 0.00003861217680924970000

measure v contain 0.00002903659299343160000

measure v carve 0.00004628940953163840000

measure v compute 0.00373544119548533000000

measure v estimate 0.03782652166600750000000

measure v size 0.00013337324186352700000

measure v graph 0.00000124519262462736000

measure v tailor 0.00006645351086355000000

measure v determine 0.38046116089559900000000

measure v assign 0.00063835235716274200000

measure v read 0.00062174777089844400000

measure v map 0.00087281812614782400000

measure v adapt 0.00003702867266769750000

measure v calculate 0.05180348360539280000000

measure v scale 0.00222248618060078000000

measure v check 0.00096143364190539000000

measure v time 0.00018344527705091600000

measure v evaluate 0.14206621632980500000000

measure v probe 0.00010826862883615600000

measure v argue 0.00004251465887404690000

measure v rank 0.00010580522305591700000

measure v evidence 0.00079072896873512400000

measure v substantiate 0.00013218596733122600000

measure v balance 0.00006619353048866250000

measure v match 0.00056972373906264100000

measure v tell 0.00005594345868387510000

measure v assess 0.29776700123880700000000

measure v qualify 0.00000008938532517300470

measure v limit 0.00275781428499077000000

measure v pattern 0.00002717418784810690000

measure v stroke 0.00008486391747467030000

measure v plot 0.00033175210261364900000

measure v step 0.00006010866707260210000

measure v calibrate 0.00009655352308256110000

much r about 0.04211026741922620000000

much r exceedingly 0.00418938362185710000000

much r almost 0.00242420350311680000000

much r nearly 0.03522459154591150000000

much r exceptionally 0.00513213041808042000000

much r so 0.03809441474060230000000

much r extremely 0.03491930662992350000000

much r routinely 0.00002352919365962170000

much r greatly 0.01563130979213680000000

much r normally 0.00043972591429456900000

much r considerably 0.21722879288019700000000

much r vastly 0.01341164038598440000000

much r well 0.01137772246006360000000

much r often 0.03110355535111380000000

much r chronically 0.00000381337514528982000

much r approximately 0.07705540406162630000000

much r frequently 0.02857094031727830000000

much r persistently 0.00718285088948798000000

much r highly 0.01021839267503570000000

much r very 0.23948072870006300000000

much r notably 0.00378163432937924000000

much r usually 0.03245508123408950000000

much r commonly 0.00003967940942598920000

much r generally 0.12189288864151600000000

much r roughly 0.00038441007475751600000

much r appreciably 0.02762360243602750000000

er n emergency room 1.00000000000000000000000

structure n design 0.02100216180461830000000

structure n system 0.26925647281175400000000

structure n anatomy 0.30154781663398000000000

structure n pattern 0.15116327889961600000000

structure n composition 0.02559233142335840000000

structure n shape 0.00962965530951275000000

structure n organization 0.02089929999336480000000

structure n figure 0.00025608442978357300000

structure n configuration 0.00292344548858289000000

structure n pile 0.00001221668750120510000

structure n construct 0.00065243910512835900000

structure n form 0.09186009981130630000000

structure n framework 0.00001683427348877110000

structure n frame 0.00004350193964732410000

structure n erection 0.00001039342400772550000

structure n formation 0.04215682200941190000000

structure n construction 0.00022597763305346600000

structure n house 0.00001956580960343960000

structure n order 0.00282822687016004000000

structure n arrangement 0.00004661886057500410000

structure n conformation 0.05985675678154680000000

preemptive a pre-emptive 1.00000000000000000000000

paraffin n paraffin wax 1.00000000000000000000000

height n rise 0.00114310649361656000000

height n prominence 0.00005183337633943280000

height n extreme 0.00080745988498695600000

height n ceiling 0.00000329725952340919000

height n limit 0.02169433589127810000000

height n slope 0.02628744564055470000000

height n extremity 0.00011638716898569300000

height n crown 0.00003479652961310760000

height n tip 0.00003247550296320930000

height n peak 0.05319459943529630000000

height n crisis 0.00030918358437542300000

height n stature 0.16703289037995400000000

height n projection 0.00006467332738325120000

height n plateau 0.00001102682744392000000

height n length 0.60046743293717400000000

height n top 0.01497120686603950000000

height n head 0.00019772295986651800000

height n end 0.09703160085024000000000

height n maximum 0.01515188878858370000000

height n altitude 0.00061736935530059800000

height n elevation 0.00077926694048171900000

cerebral cortex n cortex 1.00000000000000000000000

brace n arm 0.38864112731433400000000

brace n splint 0.00132980712171525000000

brace n block 0.06578564649076240000000

brace n two 0.25999987645395400000000

brace n pair 0.01968798338117250000000

brace n clamp 0.00876255875630235000000

brace n couple 0.00417367174947976000000

brace n clip 0.01968798338117250000000

brace n mainstay 0.05739416062598230000000

brace n stay 0.17320737760341100000000

brace n peg 0.00132980712171525000000

now r currently 0.85380247404444200000000

now r immediately 0.00593530169626795000000

now r directly 0.00089535991165184400000

now r intermittently 0.00000400428273159391000

now r rarely 0.06176882965128740000000

now r sporadically 0.00153082457709381000000

now r sometimes 0.00883243562439333000000

now r soon 0.00968029862379279000000

now r occasionally 0.00339449671139980000000

now r randomly 0.00009116406951459130000

now r promptly 0.00078487566628021700000

now r infrequently 0.00047727044936827500000

now r presently 0.05255939650726650000000

now r away 0.00024326818450979500000

pace n measure 0.00023276177386639500000

pace n speed 0.21946110107402900000000

pace n gait 0.12801897562651700000000

pace n rate 0.59742188625708000000000

pace n velocity 0.05486527526850730000000

dry v preserve 0.28919513819932400000000

dry v exhaust 0.02912603445159780000000

dry v cure 0.62815973904426800000000

dry v drain 0.05351908830481090000000

percutaneous a transdermal 0.10097613882863300000000

percutaneous a transcutaneous 0.89902386117136700000000

profession n position 0.00719391311176908000000

profession n art 0.00296811173461091000000

profession n work 0.00164105179737968000000

profession n concern 0.00296811173461091000000

profession n trade 0.00296811173461091000000

profession n role 0.36915748752731400000000

profession n situation 0.00141508941564066000000

profession n office 0.06324361157594020000000

profession n assertion 0.00046927337356576600000

profession n specialty 0.01274677442615850000000

profession n job 0.13019919592433300000000

profession n occupation 0.03042270280633690000000

profession n service 0.37283844952026600000000

profession n line 0.00176811531746343000000

match n correspondence 0.00058320551146193300000

match n wife 0.00062146634857639000000

match n race 0.00201886321507852000000

match n duplicate 0.00127353040258014000000

match n candidate 0.00074533281249837800000

match n event 0.28188892824023300000000

match n association 0.35498478023822800000000

match n combination 0.07036939753092240000000

match n pair 0.19693515316752900000000

match n twin 0.02198202787167640000000

match n union 0.00644339350012671000000

match n copy 0.00145038906976006000000

match n couple 0.00149066562499676000000

match n tally 0.00145038906976006000000

match n spouse 0.00059023872997329500000

match n sister 0.00062146634857639000000

match n trial 0.05592930596944670000000

match n brother 0.00062146634857639000000

leishmaniasis n kala azar 1.00000000000000000000000

fuel n stimulus 0.04057434363412260000000

fuel n coal 0.00025204650584077000000

fuel n food 0.95917360986003700000000

reliance n dependence 0.99977871210444800000000

reliance n expectation 0.00022128789555211200000

compile v collate 0.00945083149265431000000

compile v accumulate 0.00187039666936930000000

compile v select 0.09243362666177270000000

compile v cull 0.17748382697611900000000

compile v order 0.00019221035206590800000

compile v collect 0.70791380156245600000000

compile v gather 0.00951778987313714000000

compile v group 0.00113751641242630000000

inhale v inspire 0.99996362198651900000000

inhale v puff 0.00003637801348140830000

survivor n orphan 1.00000000000000000000000

wart n verruca 1.00000000000000000000000

urgently r primarily 0.00020111956558173800000

urgently r vigorously 0.15680622129856200000000

urgently r desperately 0.84299265913585600000000

aggressive a disruptive 0.10080288582986800000000

aggressive a hard-hitting 0.00161236740184036000000

aggressive a combative 0.04267399056870820000000

aggressive a fast-growing 0.00010650746398180100000

aggressive a bold 0.00100017165395410000000

aggressive a vigorous 0.11166284991212100000000

aggressive a invasive 0.65677514398066900000000

aggressive a destructive 0.07841788705810910000000

aggressive a violent 0.00694819613074897000000

suitability n feasibility 0.03959683225341970000000

suitability n appropriateness 0.54643628509719200000000

suitability n fitness 0.41396688264938800000000

somnolence n drowsiness 0.26777542332417100000000

somnolence n lethargy 0.18991099774851500000000

somnolence n sleepiness 0.54231357892731300000000

penicillin g n benzylpenicillin 1.00000000000000000000000

peculiar a unusual 0.03873740855916590000000

peculiar a atypical 0.00309131475646511000000

peculiar a characteristic 0.46688136689226800000000

peculiar a distinct 0.08529653878278610000000

peculiar a particular 0.00051521912607751700000

peculiar a diagnostic 0.00303270052141647000000

peculiar a typical 0.00200226226926143000000

peculiar a specific 0.02988468110286500000000

peculiar a unique 0.04256859302168080000000

peculiar a uncommon 0.00281154523101441000000

peculiar a original 0.00051521912607751700000

peculiar a outstanding 0.00103043825215503000000

peculiar a suggestive 0.00103043825215503000000

peculiar a abnormal 0.02819827516965870000000

peculiar a significant 0.00992061682221827000000

peculiar a distinctive 0.24631269424531800000000

peculiar a striking 0.00309131475646511000000

peculiar a local 0.00654584912671666000000

peculiar a appropriate 0.00051521912607751700000

peculiar a rare 0.01799892073511380000000

peculiar a remarkable 0.00103043825215503000000

peculiar a symptomatic 0.00647146447754943000000

peculiar a notable 0.00251748139533895000000

notification n report 0.46089395409818600000000

notification n advice 0.53910604590181400000000

acute a critical 0.02944864394005310000000

acute a severe 0.50656101807885900000000

acute a essential 0.00354908325180291000000

acute a imminent 0.00002458464587760540000

acute a deep 0.00515529886865352000000

acute a distressing 0.00054149875041439800000

acute a grave 0.00001734275127234160000

acute a important 0.00570471915403310000000

acute a major 0.15702488482301600000000

acute a exquisite 0.00000395821700617064000

acute a precipitous 0.00147173890678286000000

acute a drastic 0.00011634664633098500000

acute a painful 0.00658816587899123000000

acute a sensitive 0.00024860740251247400000

acute a profound 0.01410915487282610000000

acute a aware 0.00000079440989699113100

acute a sudden 0.03460687608283850000000

acute a intuitive 0.00004209693471235060000

acute a intense 0.00277659564469853000000

acute a urgent 0.01003561360133720000000

acute a crucial 0.00091061633147367000000

acute a extreme 0.01202560253017660000000

acute a rapid 0.06163673841672670000000

acute a vital 0.00201682829694741000000

acute a sharp 0.00073253456946825700000

acute a abrupt 0.02388320683130880000000

acute a dangerous 0.00042842054052201500000

acute a quick 0.00105543029171679000000

acute a discriminating 0.00008795252430973250000

acute a serious 0.07523363849500070000000

acute a hazardous 0.00001553309029036790000

acute a violent 0.00013796286079011600000

acute a great 0.03938399118123370000000

acute a imperative 0.00018511540491891700000

acute a overwhelming 0.00048985078947116200000

acute a judicious 0.00005554896272193630000

acute a impending 0.00369400602100876000000

depend v count 0.02259018345918590000000

depend v look 0.00078224225054235600000

depend v calculate 0.97662757429027200000000

immunoglobulin m n igm 1.00000000000000000000000

reliable a substantial 0.00961140952952213000000

reliable a impeccable 0.00000090505009524751700

reliable a certain 0.02216330120265850000000

reliable a responsible 0.00572005281330817000000

reliable a true 0.00128326623907670000000

reliable a good 0.40754920414210400000000

reliable a absolute 0.00930850101766947000000

reliable a regular 0.00008284293975497620000

reliable a evident 0.02267035793652210000000

reliable a careful 0.00312784652866179000000

reliable a positive 0.04610916794015110000000

reliable a credible 0.00050979938762368500000

reliable a decisive 0.00001340166634131660000

reliable a clear 0.03134491127655490000000

reliable a definite 0.03928211322780600000000

reliable a secure 0.00000591370130231134000

reliable a constant 0.00377691689078507000000

reliable a dependable 0.01233279031206640000000

reliable a solid 0.00079152842843447400000

reliable a infallible 0.00012941371502517800000

reliable a workable 0.00002792920720621350000

reliable a real 0.00020591028456051900000

reliable a upright 0.00000181010019049503000

reliable a predictable 0.00261871721922541000000

reliable a safe 0.25571960737462700000000

reliable a incontrovertible 0.00002792920720621350000

reliable a self-evident 0.00001549258971397900000

reliable a faithful 0.00630548401358948000000

reliable a sound 0.00011761579089162800000

reliable a stable 0.01445550559582360000000

reliable a trustworthy 0.00003956825485659210000

reliable a strong 0.07899979953064340000000

reliable a explicit 0.00148899374054653000000

reliable a truthful 0.00004456172447766410000

reliable a firm 0.00300407637614448000000

reliable a conclusive 0.00755743498847768000000

reliable a unequivocal 0.00449555779244155000000

reliable a genuine 0.00650343543910939000000

reliable a competent 0.00255692682480403000000

broaden v extend 0.32338073317075000000000

broaden v expand 0.65181519118299600000000

broaden v open 0.00150359723108437000000

broaden v increase 0.00842178735459873000000

broaden v stretch 0.00002146569752212070000

broaden v develop 0.01485722536304870000000

admixture n combination 1.00000000000000000000000

remember v place 0.00111966493817215000000

remember v honour 0.00011202600298214600000

remember v think 0.00126014376063681000000

remember v mind 0.00006659809691899180000

remember v retain 0.00096341858931194900000

remember v review 0.01488995711292910000000

remember v learn 0.02153824866849700000000

remember v retrieve 0.00025190973379750000000

remember v get 0.00006659809691899180000

remember v cite 0.00011202600298214600000

remember v commend 0.00011202600298214600000

remember v recognize 0.70115971196798600000000

remember v identify 0.25834767102588500000000

saturnism n lead poisoning 1.00000000000000000000000

otolaryngology n otorhinolaryngology 1.00000000000000000000000

future a imminent 0.00014171300913403300000

future a expected 0.00587664254826516000000

future a likely 0.00013139769287277700000

future a probable 0.00051324731508168600000

future a forthcoming 0.01941379316978090000000

future a next 0.03559774917432730000000

future a pending 0.00157815883619952000000

future a prospective 0.33603692848612100000000

future a subsequent 0.59791088884535800000000

future a eventual 0.00276431464377057000000

future a intended 0.00003516627908974260000

manager n pilot 1.00000000000000000000000

induration n stiffness 0.50000000000000000000000

induration n resistance 0.50000000000000000000000

fluorine n f 1.00000000000000000000000

discriminatory a discriminative 1.00000000000000000000000

rude a defiant 1.00000000000000000000000

replacement n relief 0.02370026910897290000000

replacement n cover 0.00054175009023900000000

replacement n replenishment 0.04507360750788480000000

replacement n substitution 0.75966849379200900000000

replacement n substitute 0.17099528101469200000000

replacement n transposition 0.00002059848620230550000

accrue v result 0.00105629436817389000000

accrue v fall 0.00747721544239971000000

accrue v mature 0.00033773793103033300000

accrue v grow 0.01079983671849550000000

accrue v extend 0.00016152010993846400000

accrue v rise 0.01119167070661090000000

accrue v expand 0.10080066146604500000000

accrue v augment 0.00002530489450234440000

accrue v gain 0.00986777861487029000000

accrue v advance 0.00020391513548989400000

accrue v cumulate 0.00050084999999384700000

accrue v wax 0.00002913073364141340000

accrue v accumulate 0.08689435327882180000000

accrue v increase 0.18597991651629900000000

accrue v derive 0.00070422091853708700000

accrue v collect 0.55389838950216400000000

accrue v gather 0.03007120366298590000000

or n surgery 1.00000000000000000000000

substantial a worthwhile 0.00119419207475323000000

substantial a comfortable 0.00000306951816934500000

substantial a reliable 0.00117637327322242000000

substantial a constitutional 0.00000217394294800500000

substantial a minor 0.00233262718710528000000

substantial a tall 0.00000338968657243132000

substantial a responsible 0.00076835467026681400000

substantial a noteworthy 0.00022081554265440800000

substantial a essential 0.00073809106733244400000

substantial a true 0.00068772198716577300000

substantial a indispensable 0.00001398996308242730000

substantial a fast 0.00003843342428188370000

substantial a large 0.08679359000314450000000

substantial a absolute 0.00340007721300312000000

substantial a compact 0.00000068175772662700900

substantial a considerable 0.11374225621812200000000

substantial a positive 0.00597028453261966000000

substantial a influential 0.00000694842206772488000

substantial a sizable 0.00415974072081354000000

substantial a valuable 0.00072348324511785600000

substantial a unimportant 0.00000159523054061492000

substantial a extraordinary 0.00035332716955264100000

substantial a massive 0.00001514073153504440000

substantial a temporal 0.00141487401019103000000

substantial a close 0.00187088335347534000000

substantial a substantive 0.00005716664789198340000

substantial a important 0.03445042650423700000000

substantial a basic 0.00040818385555325900000

substantial a ample 0.00065711034044354100000

substantial a major 0.03688197560412420000000

substantial a actual 0.00119042149256863000000

substantial a sensible 0.00000031848809954264600

substantial a sizeable 0.00246804818675806000000

substantial a easy 0.00000701231827574169000

substantial a impermeable 0.00000159523054061492000

substantial a solid 0.00012279017538012500000

substantial a healthy 0.00095196765012854000000

substantial a existing 0.00074680375705522200000

substantial a factual 0.00000270789384751500000

substantial a visible 0.00001124149863738220000

substantial a valid 0.00036854607746210000000

substantial a useful 0.00156870311348244000000

substantial a principal 0.00046017492995110300000

substantial a consequential 0.00000031848809954264600

substantial a significant 0.54236480627417900000000

substantial a powerful 0.00000210667151160220000

substantial a palpable 0.00001630959398348750000

substantial a somatic 0.00014620871910538700000

substantial a well 0.00027448494893371000000

substantial a dense 0.00000031848809954264600

substantial a meager 0.00000478604493979396000

substantial a poor 0.00769830270540239000000

substantial a existent 0.00001191891500450620000

substantial a real 0.00014535166314781700000

substantial a independent 0.00412133186130781000000

substantial a plentiful 0.00000031848809954264600

substantial a vital 0.00169319920188071000000

substantial a insignificant 0.00067366788998188400000

substantial a abundant 0.00110059456625488000000

substantial a wealthy 0.00000011077217547606900

substantial a fundamental 0.00012946738827841100000

substantial a hard 0.00003638500517371610000

substantial a physical 0.00052425612767532000000

substantial a notable 0.00312014644726069000000

substantial a lasting 0.00106826807530471000000

substantial a long-lasting 0.00036754452880615500000

substantial a durable 0.00065126316969260700000

substantial a appreciable 0.00207278570260738000000

substantial a sound 0.00011058762731046400000

substantial a stable 0.00083820435659914500000

substantial a necessary 0.00170338193077200000000

substantial a serious 0.01154036575368590000000

substantial a tight 0.00000120661846094540000

substantial a strong 0.01271534018509500000000

substantial a great 0.05796048242611270000000

substantial a firm 0.00039756972707143500000

substantial a imperative 0.00011087705459314300000

substantial a big 0.00025080886530453900000

substantial a steady 0.00101628848239806000000

substantial a marked 0.04107686697605230000000

substantial a basal 0.00003669035391799190000

substantial a bulky 0.00000747259930734589000

substantial a genuine 0.00002229659651137930000

hydrolyze v hydrolyse 1.00000000000000000000000

ridicule v abuse 0.80000000000000000000000

ridicule v expose 0.20000000000000000000000

hide v suppress 0.02303468760296160000000

hide v conceal 0.00112765091144940000000

hide v obliterate 0.00112765091144940000000

hide v screen 0.05209851870088110000000

hide v shield 0.00338493168195793000000

hide v protect 0.02450928130454650000000

hide v hibernate 0.00242062846619862000000

hide v block 0.00225530182289883000000

hide v wrap 0.00112765091144940000000

hide v secrete 0.00578927064462063000000

hide v cover 0.00225530182289883000000

hide v withdraw 0.20999156673444200000000

hide v store 0.03339792271231030000000

hide v drop 0.00466161973317123000000

hide v obstruct 0.02603190495513870000000

hide v confuse 0.11031842243218400000000

hide v reserve 0.40987291503908200000000

hide v withhold 0.02318768263794480000000

hide v mask 0.01255913400853650000000

hide v obscure 0.05084795696587830000000

cornerstone n basis 0.98736713801304700000000

cornerstone n foundation 0.01263286198695270000000

meaning n interpretation 0.00945163747521261000000

meaning n effect 0.33593030520449300000000

meaning n core 0.00029810788124753700000

meaning n interest 0.00178864728748522000000

meaning n design 0.00036816600901930400000

meaning n purpose 0.11379683023252300000000

meaning n object 0.02033893918559340000000

meaning n explanation 0.00477467805812085000000

meaning n suggestion 0.00059621576249507400000

meaning n connotation 0.00766299018772739000000

meaning n aim 0.04698016863836020000000

meaning n application 0.00267953554499011000000

meaning n end 0.00066627389026684100000

meaning n implication 0.09706161145117290000000

meaning n significance 0.35103761588752600000000

meaning n point 0.00656827730376633000000

local anaesthetic n topical anesthetic 0.17148361859826800000000

local anaesthetic n local anesthetic 0.82851638140173200000000

ideally r theoretically 1.00000000000000000000000

prediction n anticipation 0.00008600364347744000000

prediction n speculation 0.01601847534948720000000

prediction n prognostication 0.01196654689786290000000

prediction n supposition 0.00035683809269714900000

prediction n guess 0.00025209817994325300000

prediction n conjecture 0.00030590996269566600000

prediction n projection 0.01281383644248680000000

prediction n prognosis 0.95820029143135000000000

ensue v succeed 0.00021045352254004700000

ensue v result 0.14191752746783000000000

ensue v happen 0.02654720862898020000000

ensue v arise 0.02613976365724140000000

ensue v appear 0.00154637588301165000000

ensue v emerge 0.00680913782238627000000

ensue v follow 0.04501147498634440000000

ensue v occur 0.64052259790949000000000

ensue v develop 0.11129546012217600000000

unwanted a unpleasant 0.00246382606252499000000

unwanted a undesired 0.16189390419174600000000

unwanted a undesirable 0.83564226974572900000000

sophisticated a excellent 0.00339565059122105000000

sophisticated a refined 0.78247195748826100000000

sophisticated a modern 0.13663397316450300000000

sophisticated a advanced 0.07749841875601440000000

retarded a limited 0.37975903172229000000000

retarded a deficient 0.01143360525615500000000

retarded a abnormal 0.60880736302155500000000

popularity n recognition 0.02569554169643140000000

popularity n prevalence 0.11651258157268300000000

popularity n demand 0.00093730446952368800000

popularity n distinction 0.00002435897531192480000

popularity n acceptance 0.52135183895774500000000

popularity n note 0.00014615385187154900000

popularity n favor 0.33533222047643400000000

dead a deceased 0.05300356506588240000000

dead a correct 0.49680947644351100000000

dead a poor 0.26728511685387100000000

dead a insensitive 0.15287709487342900000000

dead a complete 0.03002474676330640000000

reconstruction n reformation 0.36865685216559800000000

reconstruction n rehabilitation 0.15895640891988400000000

reconstruction n remodeling 0.03344260133196770000000

reconstruction n restoration 0.40167963217927600000000

reconstruction n regeneration 0.03726450540327430000000

cereal n meal 0.37820935382897900000000

cereal n maize 0.27635139829823200000000

cereal n grain 0.34543924787279000000000

lethargy n drowsiness 0.29228338484660100000000

lethargy n somnolence 0.30635703967402100000000

lethargy n rest 0.00619524050388419000000

lethargy n tiredness 0.02940424892949380000000

lethargy n fatigue 0.36332412003984700000000

lethargy n coma 0.00168193638523913000000

lethargy n sleepiness 0.00075402962091448300000

lb n pound 1.00000000000000000000000

air n climate 0.00638768935922049000000

air n aspect 0.00013060331403610700000

air n atmosphere 0.07377781209899670000000

air n manner 0.00878307286892818000000

air n way 0.00001450698522791890000

air n presence 0.04512339926967620000000

air n circulation 0.00056353409431415000000

air n breath 0.18866158128845600000000

air n space 0.02348463955132970000000

air n expression 0.00387413537604365000000

air n exposure 0.21983658374878100000000

air n attitude 0.00000695107901832957000

air n mood 0.00000453466169418680000

air n publication 0.00000241641732414277000

air n impression 0.00001450698522791890000

air n port 0.01773505098533570000000

air n ozone 0.01826159864288230000000

air n oxygen 0.35404374552762900000000

air n style 0.00004433096716178260000

air n behavior 0.00002599272594043540000

air n character 0.00001450698522791890000

air n appearance 0.03367586078044120000000

air n discharge 0.00095174397316818900000

air n line 0.00008886104126117330000

air n ventilation 0.00392946524503185000000

air n conduct 0.00001850528916287200000

air n strain 0.00054437073848321900000

apneic a apnoeic 1.00000000000000000000000

succeed v suppress 0.00037083592277284400000

succeed v manage 0.00027333818566268700000

succeed v ensue 0.00042267569212177700000

succeed v result 0.00302622968230712000000

succeed v benefit 0.31052733777084000000000

succeed v recover 0.02275821837402130000000

succeed v obtain 0.00320112714639376000000

succeed v grow 0.00053333215128977400000

succeed v progress 0.13473433644797900000000

succeed v gain 0.00013666909283134300000

succeed v replace 0.00098918289552279100000

succeed v acquire 0.00013666909283134300000

succeed v advance 0.00013666909283134300000

succeed v thrive 0.00003249924570338590000

succeed v work 0.00299975855530222000000

succeed v supervene 0.00003249924570338590000

succeed v click 0.00003249924570338590000

succeed v assume 0.00027333818566268700000

succeed v achieve 0.00912158115659792000000

succeed v accomplish 0.00068334546415671800000

succeed v attain 0.00123002183548209000000

succeed v inherit 0.00054667637132537300000

succeed v overcome 0.00095668364981940500000

succeed v do 0.03325747196096980000000

succeed v receive 0.18637396759204000000000

succeed v complete 0.00224444349657436000000

succeed v get 0.00003249924570338590000

succeed v reduce 0.00594176948315455000000

succeed v follow 0.23094379437055400000000

succeed v realize 0.00205003639247016000000

succeed v reach 0.03215034585177860000000

succeed v secure 0.00013666909283134300000

succeed v score 0.01371347801106210000000

shot n lead 0.00107543373718823000000

shot n range 0.00111986258731245000000

shot n report 0.00752803616031760000000

shot n injection 0.90872406075392800000000

shot n stroke 0.07272699261312680000000

shot n photograph 0.00107543373718823000000

shot n eruption 0.00215086747437646000000

shot n discharge 0.00559931293656224000000

sap n blood 0.54467289719626200000000

sap n secretion 0.45532710280373800000000

pb n lead 1.00000000000000000000000

test n experiment 0.02398872546487040000000

test n crack 0.00003411225633240790000

test n evaluation 0.07455380188328340000000

test n scrutiny 0.00004285906462857100000

test n essay 0.00001427738093051850000

test n attempt 0.00214576648609602000000

test n probing 0.00000249976204041667000

test n search 0.00127683553682320000000

test n model 0.04056102876874480000000

test n probe 0.00436866898542407000000

test n elimination 0.00020258151380520800000

test n measure 0.04348530230361930000000

test n assessment 0.03595631244986540000000

test n examination 0.04879425524446640000000

test n checkup 0.00003890737078362300000

test n criterion 0.04949477840997080000000

test n exploration 0.00133310724555792000000

test n pattern 0.00803616575515431000000

test n experience 0.00711691304167192000000

test n questionnaire 0.01532792616410270000000

test n analysis 0.20534230959815000000000

test n investigation 0.02908960212039300000000

test n study 0.36656009394000900000000

test n quiz 0.00001136362542036930000

test n appraisal 0.00089775086189408200000

test n inquiry 0.00112525440096196000000

test n proof 0.00014928627441227600000

test n exam 0.00134085625654878000000

test n trial 0.02756560935857240000000

test n standard 0.01114131793289130000000

test n check 0.00000173054257474158000

girl n daughter 0.18076094125094300000000

girl n number 0.39510549643540000000000

girl n bird 0.00035678438176390900000

girl n chick 0.00087893674578343600000

girl n schoolgirl 0.40866509727860500000000

girl n teenager 0.00676466293265200000000

girl n young woman 0.00746808097485271000000

outdoor a free 1.00000000000000000000000

maxilla n upper jaw 1.00000000000000000000000

prostitute v sell 0.33333333333333300000000

prostitute v abuse 0.66666666666666700000000

stocking n footwear 0.03669724770642200000000

stocking n hosiery 0.96330275229357800000000

enhancement n boost 0.00055386489473812600000

enhancement n reinforcement 0.00010106347732981300000

enhancement n increase 0.83269320768178900000000

enhancement n intensification 0.00016028514378027600000

enhancement n accretion 0.00107947137647941000000

enhancement n augmentation 0.10774276718138100000000

enhancement n increment 0.00355200014304243000000

enhancement n amplification 0.03202828515659390000000

enhancement n elevation 0.01981738670949410000000

enhancement n doubling 0.00039473207050366400000

enhancement n magnification 0.00003714147104186150000

enhancement n enrichment 0.00183979469382577000000

innovation n institution 0.04182985207730580000000

innovation n modification 0.03874252993161840000000

innovation n introduction 0.14052761802615800000000

innovation n design 0.19213240328566400000000

innovation n addition 0.35918933694749400000000

innovation n change 0.16805971969935200000000

innovation n variation 0.05386190436879540000000

innovation n alteration 0.00565663566361284000000

white blood cell n white cell 0.16743597094801200000000

white blood cell n leukocyte 0.66263379204893000000000

white blood cell n leucocyte 0.00281441131498471000000

white blood cell n wbc 0.16711582568807300000000

sleeve n arm 1.00000000000000000000000

market v deliver 0.06659988888197450000000

market v handle 0.00529935218070455000000

market v dispense 0.07490690377619890000000

market v sell 0.00529935218070455000000

market v transmit 0.79189331845985200000000

market v send 0.00529935218070455000000

market v deal 0.03480377579774710000000

market v offer 0.01589805654211370000000

athletic a active 1.00000000000000000000000

seemingly r apparently 1.00000000000000000000000

inh n isoniazid 1.00000000000000000000000

acknowledge v return 0.00009932787374116870000

acknowledge v believe 0.36108921085559900000000

acknowledge v address 0.01868928205534400000000

acknowledge v settle 0.00009932787374116870000

acknowledge v concern 0.00031622375248108000000

acknowledge v confirm 0.00270904575221577000000

acknowledge v reward 0.00002698654935412310000

acknowledge v approve 0.00005397309870824630000

acknowledge v agree 0.00219874570460272000000

acknowledge v accept 0.14424001675822400000000

acknowledge v answer 0.00122055435452976000000

acknowledge v support 0.00025807926936643100000

acknowledge v pay 0.00014963132999647600000

acknowledge v allow 0.00035837103835957100000

acknowledge v know 0.28120122352068200000000

acknowledge v respond 0.00014147515961966000000

acknowledge v consent 0.00007234132438704550000

acknowledge v recognise 0.00222204638076785000000

acknowledge v concede 0.00168373811021808000000

acknowledge v recognize 0.18243849576493700000000

acknowledge v notice 0.00013543448872407900000

acknowledge v admit 0.00059646898440047600000

function v serve 0.03325391891791320000000

function v operate 0.07785991228437720000000

function v act 0.08100303122593220000000

function v work 0.54809083380953100000000

function v practice 0.05209844736099720000000

function v go 0.00360832545208111000000

function v do 0.00848559535277215000000

function v perform 0.19559993559639500000000

polemic a controversial 1.00000000000000000000000

pointless a inadequate 0.12500000000000000000000

pointless a futile 0.03124999999999990000000

pointless a unnecessary 0.12500000000000000000000

pointless a weak 0.03124999999999990000000

pointless a ineffective 0.43750000000000000000000

pointless a unsuccessful 0.18750000000000000000000

pointless a meaningless 0.03124999999999990000000

pointless a insignificant 0.03124999999999990000000

leader n director 1.00000000000000000000000

inhabitant n people 0.88704182380949000000000

inhabitant n resident 0.06810592641060860000000

inhabitant n citizen 0.00963648161012200000000

inhabitant n villager 0.00052443437333997200000

inhabitant n community 0.03469133379643920000000

escape v discharge 1.00000000000000000000000

entrance n entry 0.77528722566571500000000

entrance n start 0.01189194167326820000000

entrance n beginning 0.01627318334236700000000

entrance n enrollment 0.01998927136613590000000

entrance n induction 0.00071078272070108900000

entrance n admission 0.17549220387146200000000

entrance n threshold 0.00035539136035054400000

c. trachomatis n chlamydia trachomatis 1.00000000000000000000000

anchor v remain 0.14285714285714300000000

anchor v bind 0.14285714285714300000000

anchor v stop 0.42857142857142900000000

anchor v drop 0.14285714285714300000000

anchor v continue 0.14285714285714300000000

resolution n proposal 0.00009166663008042600000

resolution n determination 0.00022760960850507800000

resolution n interpretation 0.01883342966607290000000

resolution n issue 0.00004926348710565730000

resolution n decision 0.00108485043661735000000

resolution n closure 0.04822168593062430000000

resolution n reason 0.00164224734543020000000

resolution n dream 0.00000606440327974156000

resolution n motion 0.01881086317515060000000

resolution n aspiration 0.00042370141430748300000

resolution n advance 0.00263503517488328000000

resolution n conclusion 0.32281321747745500000000

resolution n purpose 0.02925843455332330000000

resolution n expression 0.00355656345807399000000

resolution n plan 0.00099758053555579600000

resolution n project 0.00000334364715989964000

resolution n explanation 0.00030683961946252100000

resolution n firmness 0.00008666094379617070000

resolution n opinion 0.00006570181844159870000

resolution n statement 0.00000397515817086982000

resolution n suggestion 0.00007394846505093580000

resolution n judgment 0.00005347149364593450000

resolution n promise 0.00017357378407921000000

resolution n outcome 0.06621125338456920000000

resolution n analysis 0.01115359840945640000000

resolution n aim 0.00138437031617909000000

resolution n presentation 0.02749254327487620000000

resolution n intent 0.00000349290274878297000

resolution n answer 0.01783699605543170000000

resolution n objective 0.03872303847269620000000

resolution n end 0.01057892645527360000000

resolution n solvent 0.00013422847464586900000

resolution n idea 0.00058559833330766400000

resolution n finding 0.07337001271750910000000

resolution n point 0.00045821388890533300000

resolution n solution 0.01090692350295400000000

resolution n commitment 0.00000174645137439146000

resolution n target 0.00107150603563466000000

resolution n result 0.28943656790059400000000

resolution n recommendation 0.00123125519757140000000

organisation n system 0.04313803975891510000000

organisation n organization 0.95686196024108500000000

motor cortex n motor area 1.00000000000000000000000

menstruation n period 0.20756813062950800000000

menstruation n bleeding 0.49084550831810400000000

menstruation n discharge 0.00024252167285270600000

menstruation n menses 0.30134383937953500000000

cerebrospinal fluid n spinal fluid 1.00000000000000000000000

housing n case 0.97798295454545500000000

housing n capsule 0.01100852272727270000000

housing n home 0.01100852272727270000000

hostility n violence 0.01111763188852770000000

hostility n action 0.00039301822231494600000

hostility n resistance 0.00968735328594943000000

hostility n conflict 0.02140109338665560000000

hostility n aggression 0.95234865570617500000000

hostility n anger 0.00505224751037758000000

homogeneity n equivalence 0.00099637899882530800000

homogeneity n agreement 0.08155052271356760000000

homogeneity n uniformity 0.83205681716419900000000

homogeneity n correlation 0.07962827734184760000000

homogeneity n similarity 0.00157654667243658000000

homogeneity n parity 0.00139715236970787000000

homogeneity n identity 0.00279430473941574000000

hemoglobinopathy n haemoglobinopathy 1.00000000000000000000000

molecular a microscopic 1.00000000000000000000000

bacteriological a bacteriologic 1.00000000000000000000000

soybean n soy 1.00000000000000000000000

osteogenic sarcoma n osteosarcoma 1.00000000000000000000000

start v issue 0.00016041444661972100000

start v endorse 0.00008135021842775320000

start v divide 0.00009355241357025970000

start v undertake 0.07698902014998680000000

start v protrude 0.00000400124517293184000

start v initiate 0.32444750234344100000000

start v begin 0.33209532935512800000000

start v promote 0.00020878083773367900000

start v arise 0.00003261758628703910000

start v extend 0.00117345577908310000000

start v create 0.00009289664515286100000

start v extrude 0.00000895586039532962000

start v rise 0.00093679503508917000000

start v appear 0.04136324029409510000000

start v emerge 0.00849671558396487000000

start v separate 0.00030342834049652300000

start v launch 0.00006060127962121650000

start v leave 0.00012759464267579500000

start v introduce 0.00934933224355879000000

start v encourage 0.00404326680194418000000

start v jump 0.00000399005205480968000

start v prompt 0.00027354752637271600000

start v dilate 0.00050374913423252600000

start v implement 0.01071939773830900000000

start v open 0.00004576264984657800000

start v instigate 0.00003091655483894260000

start v go 0.00025219121517045000000

start v stare 0.00401487904537296000000

start v establish 0.04061597684764920000000

start v institute 0.07225271317864230000000

start v project 0.00104648476370623000000

start v found 0.00007784896858366620000

start v move 0.00022567665545644500000

start v organize 0.00935176330028236000000

start v get 0.00088239020305352000000

start v activate 0.00012665019493617400000

start v protract 0.00001145034139275880000

start v compete 0.00047986951415753400000

start v commence 0.03546799885398600000000

start v conceive 0.00006798392748564030000

start v develop 0.02347990823202590000000

ulcerate v degenerate 0.16629705195304400000000

ulcerate v infect 0.09810067319159840000000

ulcerate v eat 0.40300817094926900000000

ulcerate v deteriorate 0.33259410390608800000000

localise v localize 1.00000000000000000000000

expandable a expansile 1.00000000000000000000000

man n fellow 0.00003185759636710570000

man n people 0.06028549240955420000000

man n blade 0.00001084933011764690000

man n human 0.05936712734635160000000

man n husband 0.00092481036683713800000

man n soldier 0.00269929561866903000000

man n satellite 0.00000220917905498766000

man n model 0.01123479065674590000000

man n he 0.00001335246922758660000

man n society 0.00013440519391094400000

man n right hand 0.00000354338273250209000

man n world 0.00314941247005610000000

man n male 0.18424791805711900000000

man n partner 0.00348591901122022000000

man n individual 0.09386624007684800000000

man n youth 0.00854985308682706000000

man n person 0.10094350436094300000000

man n follower 0.00004236250106793900000

man n subject 0.46580916466016000000000

man n employee 0.00069841658121745800000

man n mr 0.00069868553952973000000

man n spouse 0.00017134717380529400000

man n gentleman 0.00094044752370825700000

man n lad 0.00003214983409782560000

man n sir 0.00189752797176074000000

man n page 0.00014678741728229500000

man n piece 0.00002258226741881760000

man n standard 0.00058994791736961600000

variety n selection 0.01449357236816080000000

variety n category 0.01160586330889020000000

variety n people 0.00832296804842698000000

variety n type 0.11597142278615300000000

variety n inconsistency 0.00009565955586485960000

variety n number 0.14540671436434500000000

variety n nature 0.03726340459484220000000

variety n multiplicity 0.00565002187731464000000

variety n modification 0.00769086637158952000000

variety n race 0.00231690642660591000000

variety n species 0.00003508222334821110000

variety n model 0.00883097273783368000000

variety n range 0.26976967334989000000000

variety n collection 0.00122665437244599000000

variety n shift 0.00105706772068124000000

variety n heterogeneity 0.00467960192559129000000

variety n contrast 0.03521612082256210000000

variety n mixture 0.00143315674911258000000

variety n description 0.00541783471196292000000

variety n set 0.00153417636186293000000

variety n quality 0.00531628487903165000000

variety n discrepancy 0.00026989569036343800000

variety n deviation 0.00004315699866201090000

variety n fluctuation 0.00013300401704783300000

variety n disparity 0.00000464294380094702000

variety n array 0.03403392909714880000000

variety n group 0.08978740704362690000000

variety n form 0.00547625561136463000000

variety n family 0.02419647031536360000000

variety n grade 0.00737472870955687000000

variety n imbalance 0.00056253133268307600000

variety n difference 0.03789274423748140000000

variety n style 0.00005880118460101840000

variety n kind 0.00369184045720865000000

variety n diversity 0.00855551651916529000000

variety n disagreement 0.00014666731905669700000

variety n class 0.01319578566085220000000

variety n change 0.06020792999224560000000

variety n variation 0.00574490921347258000000

variety n potpourri 0.00070316416585384500000

variety n kidney 0.01017104537019060000000

variety n order 0.00469675852956581000000

variety n classification 0.00873815320108462000000

variety n strain 0.00098063683308843600000

etiological a aetiological 0.19509982985895500000000

etiological a etiologic 0.80490017014104500000000

witness v endorse 0.00006475051291603070000

witness v observe 0.05748065599874040000000

witness v validate 0.00064750512916031400000

witness v affirm 0.00003237525645801490000

witness v see 0.88644061739211100000000

witness v warrant 0.00375417708430521000000

witness v confirm 0.00470653860755910000000

witness v verify 0.00022662679520611000000

witness v view 0.00003237525645801490000

witness v mark 0.00003237525645801490000

witness v note 0.01190165641049230000000

witness v document 0.00723223930945159000000

witness v follow 0.01592062189831960000000

witness v substantiate 0.00077700615499237700000

witness v find 0.01042807903761630000000

witness v notice 0.00032239989975554200000

green a active 1.00000000000000000000000

underlying a critical 0.01489431724041280000000

underlying a primary 0.43172078553476600000000

underlying a essential 0.01902045409672020000000

underlying a initial 0.06506096487431640000000

underlying a basic 0.06482815815087800000000

underlying a elementary 0.00003117156670606110000

underlying a principal 0.12889680560063700000000

underlying a intrinsic 0.01107958350073700000000

underlying a crucial 0.10829858391496200000000

underlying a vital 0.00055918588029984800000

underlying a fundamental 0.01931875926496870000000

underlying a central 0.04820730648235680000000

underlying a necessary 0.08716326745720100000000

underlying a basal 0.00092065643503739400000

con n side 0.00514123508744069000000

con n interest 0.99485876491255900000000

overhear v discover 0.91666666666666700000000

overhear v learn 0.08333333333333320000000

severe combined immunodeficiency n severe combined immunodeficiency disease 0.25749699668659600000000

severe combined immunodeficiency n scid 0.74250300331340400000000

imprint n print 0.00144030808979841000000

imprint n impression 0.99855969191020200000000

handle v manage 0.12117565069586500000000

handle v use 0.27305674781048800000000

handle v market 0.00001774903753119140000

handle v dispense 0.00001774903753119140000

handle v lift 0.00003054227328369930000

handle v address 0.08191955174280540000000

handle v examine 0.01032225306565910000000

handle v operate 0.00012424326271834000000

handle v regulate 0.00009218771095300810000

handle v employ 0.00034795077926160300000

handle v direct 0.00015383314862231700000

handle v palpate 0.00026376989710651700000

handle v sell 0.00001774903753119140000

handle v utilize 0.00015974133778072300000

handle v train 0.00071982207765387600000

handle v exercise 0.00354129352837213000000

handle v work 0.00380337635519712000000

handle v administer 0.06957650410153250000000

handle v care 0.00164847986953917000000

handle v treat 0.41851821583016400000000

handle v feel 0.00003054227328369930000

handle v conduct 0.01061883737496540000000

handle v supply 0.00073261531340638400000

handle v run 0.00003054227328369930000

handle v advise 0.00021435680408780600000

handle v control 0.00266413179304069000000

handle v supervise 0.00003054227328369930000

handle v check 0.00003054227328369930000

handle v play 0.00001774903753119140000

handle v manipulate 0.00012272998423670800000

random a casual 0.46686649710364900000000

random a sporadic 0.00113447467374651000000

random a fortuitous 0.00051697899428279300000

random a irregular 0.00130897148720649000000

random a blind 0.01994644909952890000000

random a accidental 0.00038533585289522200000

random a occasional 0.49562551269418000000000

random a incidental 0.00641424001032888000000

random a arbitrary 0.00780154008418221000000

suburban a rural 1.00000000000000000000000

pathology n etiology 0.28711320240941800000000

pathology n bacteriology 0.01482082596822490000000

pathology n nosology 0.00199372168496517000000

pathology n symptomatology 0.02032503290926390000000

pathology n diagnostics 0.00045201749757905400000

pathology n pathogenesis 0.67529519953054900000000

operational a functional 0.94877603941989200000000

operational a operative 0.05122396058010830000000

motor neuron n motoneuron 1.00000000000000000000000

litigation n case 0.44680935841244200000000

litigation n lawsuit 0.22798545893834300000000

litigation n cause 0.12614989805036200000000

litigation n allegation 0.15012472097600600000000

litigation n controversy 0.04691397530500200000000

litigation n action 0.00201658831784469000000

intensity n determination 0.00327778930603902000000

intensity n attention 0.00582419068232016000000

intensity n strength 0.01025212255063400000000

intensity n magnitude 0.05250499733172960000000

intensity n power 0.06199441930464810000000

intensity n force 0.00058816532553787600000

intensity n tension 0.00176449597661363000000

intensity n volume 0.04106520968372510000000

intensity n depth 0.00005044984957904520000

intensity n emphasis 0.00013144861607801500000

intensity n extremity 0.00129907407136250000000

intensity n saturation 0.00001419127995206050000

intensity n potency 0.01088224532430830000000

intensity n sharpness 0.00000383379786249272000

intensity n severity 0.53101613268907000000000

intensity n pressure 0.03524346200198000000000

intensity n violence 0.00003159824355283270000

intensity n concentration 0.24214158298984000000000

intensity n emotion 0.00000383379786249272000

intensity n energy 0.00020549655233884700000

intensity n strain 0.00170526062496592000000

suspend v remove 0.00452552097695411000000

suspend v postpone 0.00262478359023203000000

suspend v halt 0.05485785984754730000000

suspend v discontinue 0.29368826451524300000000

suspend v stop 0.33002736053519900000000

suspend v drop 0.00262478359023203000000

suspend v continue 0.02872291255769120000000

suspend v interrupt 0.05980291424298890000000

suspend v end 0.00058548158261585600000

suspend v delay 0.09722501872986850000000

suspend v withhold 0.00805679811396460000000

suspend v reject 0.00144863832575361000000

suspend v exclude 0.10620911181680600000000

suspend v defer 0.00408495865965438000000

suspend v dissolve 0.00186798099899808000000

suspend v omit 0.00364761191625113000000

modality n mood 0.00100057424748477000000

modality n mode 0.99899942575251500000000

degenerate v return 0.01186637991147960000000

degenerate v fall 0.01715836155917950000000

degenerate v decline 0.12058460177158100000000

degenerate v worsen 0.01225597254227110000000

degenerate v deteriorate 0.21743644372020300000000

degenerate v fail 0.52359196060208200000000

degenerate v regress 0.00735358352536265000000

degenerate v decrease 0.08975269636784170000000

commission n error 0.03396739130434780000000

commission n committee 0.96603260869565200000000

spread n proliferation 0.00097984754142753500000

spread n transmission 0.32628258757955200000000

spread n gap 0.00213061609345151000000

spread n dissemination 0.00141106143879131000000

spread n extent 0.66919588734677700000000

absorption n consumption 0.01053635445007520000000

absorption n engagement 0.00000860224075329455000

absorption n immersion 0.00004183775094620600000

absorption n adsorption 0.00787465870860735000000

absorption n ingestion 0.02799625270606830000000

absorption n inhalation 0.00557564633695506000000

absorption n conversion 0.00074782306050824500000

absorption n retention 0.20985133819666400000000

absorption n obsession 0.00000860224075329455000

absorption n fusion 0.00027496349165885900000

absorption n saturation 0.00317527107554642000000

absorption n assimilation 0.01622956088788240000000

absorption n incorporation 0.00194754730654589000000

absorption n intake 0.44310339064984800000000

absorption n penetration 0.00004253732275666650000

absorption n suction 0.00007072850726147990000

absorption n union 0.00089931347587739300000

absorption n involvement 0.02742736270833400000000

absorption n digestion 0.00140762439488873000000

absorption n concentration 0.24276071155637000000000

absorption n occupation 0.00001987293169944790000

player n athlete 0.63107437326084900000000

player n professional 0.00003505838894452970000

player n participant 0.36889056835020600000000

exchange v return 0.00021933031144904200000

exchange v reverse 0.00065799093434712500000

exchange v replace 0.00153531218014329000000

exchange v transfer 0.00043866062289808300000

exchange v change 0.99298143003363100000000

exchange v switch 0.00021933031144904200000

exchange v revise 0.00372861529463372000000

exchange v substitute 0.00021933031144904200000

divide v sort 0.00297586355759004000000

divide v isolate 0.00021065078509934200000

divide v dispense 0.00058680629005021600000

divide v cut 0.00087961593276721900000

divide v arrange 0.00003623244513631110000

divide v segregate 0.00545269167996605000000

divide v halve 0.00000130799717917575000

divide v split 0.00475888498626448000000

divide v dislocate 0.00025161035884171500000

divide v dichotomize 0.00213329244217827000000

divide v unravel 0.00000043599905972525000

divide v grade 0.00076778635643018400000

divide v allocate 0.18891236492250700000000

divide v separate 0.08404813235419680000000

divide v rate 0.00086430612819885800000

divide v insulate 0.00000050017369149032300

divide v detach 0.00000043599905972525000

divide v categorize 0.04630143789445200000000

divide v open 0.00000117447952686701000

divide v rupture 0.00003800403855232160000

divide v distribute 0.01495504129133640000000

divide v allot 0.00136901590288548000000

divide v shift 0.00000077268325502035000

divide v differ 0.00009449450138317750000

divide v assign 0.41055136313588500000000

divide v segment 0.00000707891699384898000

divide v deal 0.00000124318789609291000

divide v dissect 0.00000018148884444990600

divide v cross 0.00000614457654943795000

divide v vary 0.00035951655529802500000

divide v section 0.00005540821384008370000

divide v subdivide 0.04824208911007340000000

divide v group 0.01412120765521630000000

divide v assort 0.00172686050696700000000

divide v cleave 0.00000043599905972525000

divide v dissolve 0.00000161680273925149000

divide v classify 0.17028538325789500000000

divide v tear 0.00000006049628148330090

divide v break 0.00000055089685270506100

comparatively r similarly 0.02289507545062670000000

comparatively r relatively 0.97710492454937300000000

sterilize v cleanse 0.03060345406448610000000

sterilize v incapacitate 0.02633097867476110000000

sterilize v purify 0.03060345406448610000000

sterilize v autoclave 0.03060345406448610000000

sterilize v change 0.57484349788961700000000

sterilize v alter 0.25591457304772300000000

sterilize v castrate 0.05110058819444060000000

discernible a apparent 0.11215357656794600000000

discernible a recognizable 0.00927772828473863000000

discernible a distinct 0.43403799356152000000000

discernible a noticeable 0.00358265472299548000000

discernible a distinguishable 0.00053056316004833200000

discernible a detectable 0.00723233646633542000000

discernible a observable 0.01819306081118570000000

discernible a evident 0.06105737730461900000000

discernible a audible 0.00098290746482450900000

discernible a clear 0.15690994097362100000000

discernible a visible 0.00890450500128130000000

discernible a discernable 0.00027819740998062300000

discernible a obvious 0.02206166930050430000000

discernible a palpable 0.00066320395006041600000

discernible a patent 0.00025709799853003300000

discernible a appreciable 0.16387718702181000000000

type n gender 0.00432311224013811000000

type n category 0.02398698279623800000000

type n structure 0.00455954746331664000000

type n variety 0.01472866678406580000000

type n letter 0.00296376730318162000000

type n shadow 0.00002394220153132960000

type n case 0.13907142336880600000000

type n number 0.03047581789799620000000

type n designation 0.00091271047024418500000

type n nature 0.01120134225767250000000

type n genus 0.00010427111326974900000

type n stock 0.00002648338041371880000

type n manner 0.00221767169812800000000

type n race 0.00434776691470992000000

type n subdivision 0.00108793383776925000000

type n species 0.00431898822468261000000

type n religion 0.00013122168146978700000

type n model 0.02093867652370950000000

type n instance 0.00256930416137890000000

type n sample 0.02280953941904560000000

type n design 0.02462566827630200000000

type n suit 0.00000709697835763751000

type n fashion 0.00044201223152140400000

type n vein 0.00012468534297777100000

type n criterion 0.01497701018364640000000

type n example 0.00174289433217422000000

type n pattern 0.05393742022145570000000

type n expression 0.01577694527180590000000

type n description 0.00004555419018082240000

type n set 0.00099584242765834500000

type n color 0.00004435010601227740000

type n face 0.00017517699075777300000

type n sept 0.00000115230508817561000

type n image 0.00980777046333676000000

type n specimen 0.00903024299801839000000

type n rule 0.00019229853742746400000

type n shape 0.00037278404314446100000

type n habit 0.00166242168682558000000

type n humor 0.00085731498560261100000

type n figure 0.00085144652241078700000

type n disposition 0.00005379786155509800000

type n sign 0.00516692309092699000000

type n division 0.00156961814697915000000

type n voice 0.00041545488187886800000

type n group 0.34661579660572600000000

type n form 0.06490400226412680000000

type n family 0.02168223975907350000000

type n copy 0.00000887489633128997000

type n sex 0.00968816125594140000000

type n paradigm 0.00035921819209553400000

type n pilot 0.00061388518605861800000

type n grain 0.00002792556956360380000

type n style 0.00054419022139726700000

type n kind 0.00391210166644427000000

type n idea 0.00002979523427302480000

type n class 0.03738721393824160000000

type n cap 0.00011312454409885300000

type n clan 0.00000218702802449646000

type n norm 0.00004797686313245740000

type n prototype 0.00005164699990185430000

type n symptom 0.03235079759056310000000

type n representative 0.00001575869876914080000

type n character 0.00063276989698314800000

type n indication 0.01038721048545360000000

type n kidney 0.00455367041073146000000

type n standard 0.00311325521554593000000

type n order 0.00406027608674094000000

type n lot 0.00007265381233920440000

type n classification 0.01661597923609230000000

type n marital status 0.00012788365174624000000

type n brand 0.00009120372187261820000

type n strain 0.00831514915491988000000

adjustment n resolution 0.00124104436221313000000

adjustment n agreement 0.00052844420258429100000

adjustment n regulation 0.01961333654724430000000

adjustment n compliance 0.01806445237524930000000

adjustment n correspondence 0.01011421500125690000000

adjustment n alleviation 0.00011371640258632200000

adjustment n healing 0.00003544971518953420000

adjustment n modification 0.07403326532675640000000

adjustment n instrument 0.00199353459238974000000

adjustment n handle 0.00001024967290869040000

adjustment n apportionment 0.00292514412189830000000

adjustment n correction 0.47391674765630500000000

adjustment n allowance 0.02445863253505510000000

adjustment n satisfaction 0.02927188420505710000000

adjustment n acculturation 0.00017102888951379900000

adjustment n habituation 0.00725961728821674000000

adjustment n conversion 0.00192736328901895000000

adjustment n improvement 0.04593978087671160000000

adjustment n composition 0.00550212598281414000000

adjustment n remodeling 0.00000729423302708701000

adjustment n tweak 0.00036783775122310300000

adjustment n transformation 0.00001415407234074000000

adjustment n readjustment 0.00713039333140174000000

adjustment n organization 0.00182487835820863000000

adjustment n adaptation 0.03109811882831890000000

adjustment n benefit 0.02917658078395000000000

adjustment n acclimatization 0.00061098473931973100000

adjustment n familiarization 0.00001857192149400520000

adjustment n compromise 0.00026808463893207400000

adjustment n registration 0.00061072933844112200000

adjustment n compensation 0.00108638895016463000000

adjustment n reimbursement 0.00078830720818051000000

adjustment n payment 0.00067703896598202700000

adjustment n alignment 0.00000729423302708701000

adjustment n adaption 0.00720961992397285000000

adjustment n repair 0.00791570096828517000000

adjustment n calibration 0.00006264960085386320000

adjustment n change 0.16902602976203600000000

adjustment n standardization 0.00055825939682313300000

adjustment n coordination 0.00002474697914407150000

adjustment n arrangement 0.00060743456651717900000

adjustment n familiarity 0.00051497285171234400000

adjustment n alteration 0.02327389555367470000000

alert n alarum 0.23076923076923100000000

alert n caution 0.76923076923076900000000

perforate v cut 0.06167466986794720000000

perforate v penetrate 0.93832533013205300000000

rupture n litigation 0.00016048694935585000000

rupture n fissure 0.00009636381557304420000

rupture n variance 0.00007352657430998770000

rupture n tear 0.37558859403645000000000

rupture n space 0.01238253377276440000000

rupture n fault 0.00006703569778994370000

rupture n disruption 0.47530892968113700000000

rupture n controversy 0.04251169013334640000000

rupture n separation 0.01808316840943310000000

rupture n gap 0.00002749968452365860000

rupture n hernia 0.01284345481480650000000

rupture n slice 0.00001755327107011570000

rupture n fracture 0.05678726253927540000000

rupture n rent 0.00001118129742986890000

rupture n division 0.00539637367209047000000

rupture n burst 0.00016996452510521200000

rupture n disagreement 0.00042828321566835500000

rupture n cleft 0.00002202601498812440000

rupture n cut 0.00001074841015230270000

rupture n argument 0.00001332348473124070000

insert n supplement 0.50000000000000000000000

insert n ad 0.50000000000000000000000

hindlimb n hind limb 1.00000000000000000000000

defecation n diarrhea 1.00000000000000000000000

symmetry n agreement 0.09661068965687190000000

symmetry n balance 0.00416220567820381000000

symmetry n cadence 0.76583669795875500000000

symmetry n appropriateness 0.00070470365581665700000

symmetry n fitness 0.00140940731163332000000

symmetry n proportion 0.03690779267271110000000

symmetry n order 0.09436850306600780000000

arm n protection 0.00252498529656345000000

arm n sleeve 0.00001450932572368370000

arm n command 0.00010819101567988400000

arm n weapon 0.00002662272117906360000

arm n strength 0.10466257318850800000000

arm n limb 0.55628177846532300000000

arm n member 0.00651195791234593000000

arm n power 0.00129077673771632000000

arm n channel 0.00541857240760645000000

arm n department 0.01305287931903440000000

arm n force 0.00058367227554198200000

arm n forearm 0.25940475777324400000000

arm n potency 0.00157341418812789000000

arm n mouth 0.00005541846061518710000

arm n defense 0.00006317242578829980000

arm n division 0.00647908770662581000000

arm n projection 0.00004248191043493190000

arm n unit 0.04027916462014040000000

arm n sound 0.00042943287473582400000

arm n rod 0.00005033560180102320000

arm n support 0.00114621577326485000000

propagation n increase 0.00820937163073548000000

propagation n transmission 0.00410468581536774000000

propagation n extension 0.87877591774464000000000

propagation n growth 0.08814410623741800000000

propagation n distribution 0.02076591857183900000000

density n thickness 0.04216878242860040000000

density n substance 0.00073550685958669700000

density n frequency 0.05878640360500330000000

density n quantity 0.05944229750754500000000

density n mass 0.25434788907839800000000

density n weight 0.15542525675803800000000

density n concentration 0.36824683667855100000000

density n occurrence 0.05955045107042110000000

density n bulk 0.00116198967987715000000

density n body 0.00013458633397934700000

fluency n control 1.00000000000000000000000

elder n senior 0.17862611977065200000000

elder n master 0.10207206844037300000000

elder n mother 0.53637416392247400000000

elder n parent 0.18292764786650200000000

donate v provide 0.70821283205541900000000

donate v give 0.14478789977092400000000

donate v grant 0.00786333339960156000000

donate v will 0.00055284210068295000000

donate v contribute 0.00110568420136590000000

donate v offer 0.13747740847200600000000

molest v confuse 1.00000000000000000000000

strongly r completely 0.02030044787822450000000

strongly r firmly 0.03199840099954190000000

strongly r vigorously 0.17708901340578900000000

strongly r fully 0.04535206440879950000000

strongly r greatly 0.02976938804829970000000

strongly r well 0.06865179653726850000000

strongly r actively 0.52058548067860400000000

strongly r heavily 0.10625340804347300000000

globe n balloon 0.96783625730994100000000

globe n world 0.03216374269005850000000

mineworker n miner 1.00000000000000000000000

ending n conclusion 0.29812818483020000000000

ending n end 0.70187181516980000000000

last n ending 0.00071408486572330600000

last n death 0.05894490146098170000000

last n discontinuance 0.00033430540231125900000

last n model 0.01098170230615730000000

last n cessation 0.01838786779166550000000

last n conclusion 0.52057633891571600000000

last n completion 0.05863536509705100000000

last n example 0.01344308277074040000000

last n pattern 0.00111684213408057000000

last n termination 0.05272957070350100000000

last n shape 0.00001030525049682740000

last n matrix 0.00186653849623786000000

last n form 0.00208272937645580000000

last n paradigm 0.00077236075706394400000

last n end 0.25490644203974000000000

last n prototype 0.00001446721383620560000

last n standard 0.00448309541824060000000

culture medium n medium 1.00000000000000000000000

cardiac valve n heart valve 1.00000000000000000000000

antidote n protection 0.00184036435896820000000

antidote n vaccination 0.00201182687688450000000

antidote n antivenin 0.00201182687688450000000

antidote n medicine 0.02626249679824900000000

antidote n antibody 0.20955227384077400000000

antidote n complement 0.00100591343844225000000

antidote n antipyretic 0.00100591343844225000000

antidote n medication 0.09469170315996880000000

antidote n vaccine 0.00603548063065349000000

antidote n drug 0.60938036241972300000000

antidote n remedy 0.04620183816101020000000

water n distilled water 0.00026095188716897200000

water n tap water 0.01524318954704270000000

water n amniotic fluid 0.00237079958656311000000

water n water supply 0.01937567762229620000000

water n h2o 0.76977185512397900000000

water n liquid 0.00290197271444180000000

water n reservoir 0.00049857647405000800000

water n saliva 0.00010696483465071200000

water n fluid 0.11159227801008600000000

water n brook 0.00002873207146617380000

water n urine 0.01769598958730760000000

water n spit 0.00008787155384261310000

water n drinking water 0.05999904462094750000000

water n spring 0.00006609636615793040000

straightforward a open 0.02791680479682150000000

straightforward a apparent 0.05584491271745990000000

straightforward a distinct 0.00509513595250208000000

straightforward a true 0.02485372392567580000000

straightforward a objective 0.00196174307757154000000

straightforward a linear 0.00250964864646125000000

straightforward a clear-cut 0.00265172316437507000000

straightforward a evident 0.05483973445458030000000

straightforward a blunt 0.00042028036418572600000

straightforward a uncomplicated 0.07322356153634650000000

straightforward a equitable 0.00024778229109604800000

straightforward a free 0.00026137802138848800000

straightforward a palpable 0.00278483859618596000000

straightforward a right 0.00026137802138848800000

straightforward a routine 0.00234411205601348000000

straightforward a direct 0.08465370828574660000000

straightforward a sound 0.00026137802138848800000

straightforward a balanced 0.00071084456658573300000

straightforward a estimable 0.00026137802138848800000

straightforward a trustworthy 0.00041862447535328800000

straightforward a simple 0.65651073073428500000000

straightforward a manifest 0.00086259790102538400000

straightforward a unequivocal 0.00110398037217520000000

silver a white 1.00000000000000000000000

protrusion n swelling 0.00481346568456132000000

protrusion n projection 0.99518653431543900000000

standardize v regulate 0.00033658211178825300000

standardize v normalize 0.02887412850877970000000

standardize v stabilize 0.00033022649111981600000

standardize v institute 0.01134714419754900000000

standardize v assimilate 0.00004831246492422940000

standardize v standardise 0.95906360622583900000000

cu n copper 1.00000000000000000000000

constitutional a essential 1.00000000000000000000000

celiac a coeliac 1.00000000000000000000000

aid n reinforcement 0.00000705141557373756000

aid n protection 0.00560165890876505000000

aid n advantage 0.00058642576777141200000

aid n hand 0.00518208477455590000000

aid n maintenance 0.00434316129828512000000

aid n relief 0.00196511190020183000000

aid n adjuvant 0.00279521538951702000000

aid n attention 0.01141123893186550000000

aid n utility 0.01604404190727380000000

aid n satellite 0.00093727863515045400000

aid n advance 0.00673993438742663000000

aid n allowance 0.00000653481120124816000

aid n contribution 0.01211751917404710000000

aid n consort 0.00000635659760454411000

aid n advocate 0.00002814516397258270000

aid n adjunct 0.11150248667048100000000

aid n agent 0.03279803310009120000000

aid n right hand 0.00000635659760454411000

aid n parasite 0.00077891683608415500000

aid n crew 0.00002723641790335730000

aid n aide 0.00140591795272568000000

aid n care 0.04115711107616880000000

aid n guidance 0.00000101534758261361000

aid n restoration 0.00000705141557373756000

aid n collaborator 0.00702958976362841000000

aid n sponsorship 0.00010814753482505200000

aid n partner 0.00037543365210273600000

aid n gift 0.00001584770639143280000

aid n participant 0.02715831331588140000000

aid n principal 0.00004892175146187120000

aid n advice 0.00395266847690844000000

aid n benefit 0.00920668305876661000000

aid n use 0.12216173578830600000000

aid n assistance 0.00314059562207069000000

aid n helper 0.00008734993197408070000

aid n advancement 0.01974752664376140000000

aid n compensation 0.02961918029662070000000

aid n second 0.00190439568687422000000

aid n comfort 0.00001906979281363230000

aid n service 0.02774469501156380000000

aid n treatment 0.41727685090839600000000

aid n support 0.07050526442405920000000

aid n funding 0.00039121901753614600000

aid n cooperation 0.00390971129668910000000

aid n colleague 0.00008915819555352750000

aid n paramedic 0.00005175764638773210000

lid n report 0.01243078270998390000000

lid n eyelid 0.98636398270704100000000

lid n maximum 0.00120523458297537000000

primary a underlying 0.00454259264540646000000

primary a paramount 0.00084959341563589500000

primary a essential 0.00305007209291430000000

primary a radical 0.00130473467896678000000

primary a chief 0.00031856755042336900000

primary a cardinal 0.00001490110898303720000

primary a original 0.00420075138356998000000

primary a first 0.01465258979922480000000

primary a initial 0.03034801459295410000000

primary a important 0.02048736335401690000000

primary a basic 0.00147243096428357000000

primary a major 0.04786697894871830000000

primary a elementary 0.00059770554489155700000

primary a dominant 0.00111798708314490000000

primary a predominant 0.00192132936707583000000

primary a heavy 0.00000609568934439171000

primary a foremost 0.00036778854472376200000

primary a main 0.81341746906084800000000

primary a principal 0.02812206762544060000000

primary a ultimate 0.00236322704351656000000

primary a fundamental 0.00085215451722345600000

primary a direct 0.01480773140017970000000

primary a antecedent 0.00045293545188772000000

primary a central 0.00363043815092924000000

primary a senior 0.00012368163509862500000

primary a simple 0.00158041878146525000000

primary a prime 0.00117681889055675000000

primary a primitive 0.00005584727149347110000

primary a basal 0.00029731674203825100000

primary a elemental 0.00000039666504365895500

newborn n neonate 0.35222882061695900000000

newborn n chick 0.00000073068716562146100

newborn n infant 0.51453656102355400000000

newborn n baby 0.04810769558798910000000

newborn n weanling 0.00061119016295471100000

newborn n puppy 0.00046740882945553500000

newborn n kit 0.00005881272965139830000

newborn n kitten 0.00001331999721329960000

newborn n calf 0.00000508396840202275000

newborn n piglet 0.00714606694575586000000

newborn n lamb 0.00595927156924273000000

newborn n pup 0.00153636611279527000000

newborn n newborn infant 0.06932867176886190000000

hyaline membrane disease n respiratory distress syndrome 1.00000000000000000000000

click n dog 1.00000000000000000000000

circle n capacity 0.04927353126974100000000

circle n area 0.02210991787744790000000

circle n cycle 0.92861655085281100000000

disability n condition 0.10140185543830300000000

disability n abnormality 0.02910191457443470000000

disability n weakness 0.01689263911652680000000

disability n disorder 0.19602223728211800000000

disability n helplessness 0.00253965263478022000000

disability n incapacity 0.00126982631739011000000

disability n disablement 0.00003109778736465570000

disability n paralysis 0.00512003469328845000000

disability n inability 0.00152808319097471000000

disability n collapse 0.00030283448509949100000

disability n defect 0.04084812311772110000000

disability n impotence 0.01887661199255480000000

disability n impairment 0.51716939438267500000000

disability n disadvantage 0.00174134003584733000000

disability n impotency 0.00051653951893834900000

disability n handicap 0.06663781543198450000000

extrusion n expulsion 1.00000000000000000000000

reference n citation 0.04139681040536590000000

reference n indicator 0.00053091226832742800000

reference n informant 0.00003748509747308200000

reference n referral 0.01636421805936710000000

reference n respect 0.00257111235478337000000

reference n book 0.00665372876012773000000

reference n illustration 0.00049916763376042700000

reference n evidence 0.01528408846839710000000

reference n employer 0.00000261689584969880000

reference n consideration 0.00596925801157786000000

reference n concern 0.01437265569973460000000

reference n regard 0.00862977014866258000000

reference n extension 0.00019081269496840000000

reference n key 0.00126037508671950000000

reference n instance 0.00207588477831000000000

reference n innuendo 0.00012013239202814600000

reference n consultation 0.00037957219719336300000

reference n relevance 0.00029931643996048600000

reference n correlation 0.04507390016862690000000

reference n blame 0.00001897051049429600000

reference n remark 0.00008282224470737640000

reference n statement 0.00007692262234923380000

reference n suggestion 0.00376799033044647000000

reference n assignment 0.00028814036752688000000

reference n comment 0.01253061529566690000000

reference n note 0.00037972024388946700000

reference n applicability 0.00063927975210348400000

reference n article 0.72861124908701200000000

reference n booster 0.00028030891473233900000

reference n implication 0.00387117030966020000000

reference n source 0.04227690744491980000000

reference n authority 0.00002688871592933310000

reference n relation 0.00155705240114485000000

reference n text 0.00360412641732053000000

reference n indication 0.01692669832141180000000

reference n recommendation 0.02334931945945140000000

controller n control 1.00000000000000000000000

contributory a contributive 1.00000000000000000000000

trap v immobilize 1.00000000000000000000000

linearise v linearize 1.00000000000000000000000

negative n film 0.95220146608733500000000

negative n plate 0.04779853391266510000000

shoe n pump 1.00000000000000000000000

intent a alert 0.23590633130962700000000

intent a dedicated 0.76409366869037300000000

ebv n epstein-barr virus 1.00000000000000000000000

dispense v issue 0.00012491494088190700000

dispense v deliver 0.00119513030524725000000

dispense v measure 0.00232780314005354000000

dispense v divide 0.25218244648375800000000

dispense v undertake 0.01779899113188430000000

dispense v prescribe 0.15691347170612800000000

dispense v provide 0.01420882582361170000000

dispense v operate 0.00464756775126210000000

dispense v release 0.00018737241132286100000

dispense v give 0.25880790757491100000000

dispense v discharge 0.10824771876995000000000

dispense v allocate 0.00012491494088190700000

dispense v prepare 0.00037474482264572300000

dispense v clear 0.00006245747044095370000

dispense v forgo 0.00279994526747150000000

dispense v administer 0.15744379274278500000000

dispense v implement 0.00323714756055818000000

dispense v distribute 0.00012491494088190700000

dispense v assign 0.00088284295304247700000

dispense v detail 0.00018737241132286100000

dispense v deal 0.00082038548260152300000

dispense v apply 0.01729933136835660000000

placement n position 0.36084324711785700000000

placement n attribution 0.00000294971043996490000

placement n situation 0.00011871397860642200000

placement n stratification 0.00001474855219982450000

placement n assignment 0.00011496857418313600000

placement n disposal 0.02055063263523550000000

placement n induction 0.00816227057270079000000

placement n appointment 0.00021689322042464900000

placement n deployment 0.53700257910436700000000

placement n arrangement 0.00000282696645370864000

placement n location 0.07287794178324750000000

placement n classification 0.00009222778428425120000

bill v present 0.99484581067028700000000

bill v describe 0.00515418932971355000000

party n man 0.00561562848017435000000

party n association 0.00096980426055642600000

party n combination 0.00022996918503054100000

party n one 0.19402485762587600000000

party n woman 0.00022996918503054100000

party n group 0.79869980207830200000000

party n evening 0.00022996918503054100000

command n determination 0.00081902111596183700000

command n act 0.00023400603313195300000

command n advantage 0.00159844988043854000000

command n range 0.00011700301656597700000

command n caveat 0.00022834998291979100000

command n right 0.00022834998291979100000

command n facility 0.00011700301656597700000

command n direction 0.68837179473732100000000

command n center 0.06900199351299150000000

command n group 0.07879547501777230000000

command n control 0.08947037716092170000000

command n predominance 0.00022834998291979100000

command n unit 0.00011700301656597700000

command n sector 0.00130625510637587000000

command n knowledge 0.06868151848786810000000

command n elevation 0.00022834998291979100000

command n influence 0.00022834998291979100000

command n order 0.00022834998291979100000

art n profession 0.00515962231791950000000

art n technique 0.06128486045538000000000

art n trade 0.00515962231791950000000

art n design 0.08931325984769430000000

art n history 0.00291082562538432000000

art n scheme 0.00145541281269216000000

art n science 0.10208681300455000000000

art n literature 0.00517831660168008000000

art n performance 0.01284357598492870000000

art n craft 0.71460769103185100000000

organism n structure 0.00168881293833390000000

organism n man 0.04823132316289240000000

organism n human 0.20237388578680100000000

organism n entity 0.05558945604988230000000

organism n system 0.05437024762217000000000

organism n association 0.09579898872085920000000

organism n alliance 0.00002100553313730890000

organism n animal 0.08077776996850980000000

organism n organization 0.00000681089412332149000

organism n individual 0.08942936799947490000000

organism n plant 0.02144585648410620000000

organism n union 0.00051647890456795600000

organism n person 0.06421642222058590000000

organism n company 0.00007042597543360320000

organism n cell 0.28089467117964900000000

organism n body 0.00120312199513365000000

organism n being 0.00336535456433907000000

palate n soft palate 0.78812860676009900000000

palate n stomach 0.01483924154987630000000

palate n hard palate 0.06567738389667490000000

palate n tongue 0.13135476779335000000000

heart valve n cardiac valve 1.00000000000000000000000

isometrics n isometric exercise 1.00000000000000000000000

hand n boost 0.00003159983971790150000

hand n writing 0.00117348982308629000000

hand n man 0.04332426696882350000000

hand n aid 0.01370512077914760000000

hand n management 0.11302159084314200000000

hand n relief 0.00004406501351181420000

hand n palm 0.03498515212143240000000

hand n side 0.12686410897530300000000

hand n essay 0.00001887435434927110000

hand n attempt 0.00021111533351374600000

hand n forefoot 0.00269252427389430000000

hand n worker 0.00029936575803677200000

hand n possession 0.00012257959802658500000

hand n skill 0.03581456105519540000000

hand n foot 0.32750711975907900000000

hand n direction 0.00012257959802658500000

hand n cluster 0.00009001888672646120000

hand n care 0.00695385748439439000000

hand n extremity 0.10138086993368900000000

hand n set 0.00457355641751489000000

hand n grasp 0.00012018964177978700000

hand n script 0.00976040049286684000000

hand n fist 0.02602773464764490000000

hand n x 0.00002257566844131330000

hand n instruction 0.00376992431504131000000

hand n assistance 0.00007254555956296170000

hand n helper 0.00092406158512348700000

hand n disposal 0.00092406158512348700000

hand n duke 0.00000419802171736208000

hand n control 0.11708871941827600000000

hand n employee 0.00001831219604665440000

hand n pad 0.00002337820477333370000

hand n supervision 0.00036079024072135900000

hand n touch 0.00000419802171736208000

hand n grip 0.00443025270598211000000

hand n paw 0.02351224087856940000000

loop v curve 1.00000000000000000000000

li n lithium 1.00000000000000000000000

fe n iron 1.00000000000000000000000

minor a trivial 0.01042989863969080000000

minor a under-age 0.00002870310115246260000

minor a little 0.04819283517410250000000

minor a incompetent 0.00000562068576270017000

minor a unimportant 0.02679131949082220000000

minor a inferior 0.00027053255294294700000

minor a small 0.29531116735504400000000

minor a unknown 0.01450345100323410000000

minor a adolescent 0.00056770264877523900000

minor a modest 0.15315278235359900000000

minor a slight 0.09785804691491030000000

minor a inconsequential 0.01853391959692120000000

minor a light 0.01547993078711550000000

minor a subsidiary 0.00012002856002676500000

minor a lesser 0.03212537928677200000000

minor a secondary 0.07046668585643980000000

minor a insignificant 0.03071194006229180000000

minor a mediocre 0.00001109722074636400000

minor a negligible 0.01857135588792400000000

minor a moderate 0.12808432021330400000000

minor a low grade 0.02613704743175470000000

minor a ancillary 0.00025585327901905400000

minor a obscure 0.00058940519703385200000

minor a tolerable 0.01180097670061520000000

digest v review 0.00412683517431798000000

digest v analyze 0.12620437800530600000000

digest v support 0.02091635263564580000000

digest v absorb 0.81573775279018700000000

digest v study 0.03301468139454380000000

deploy v use 0.66482831629468800000000

deploy v place 0.28498100953880000000000

deploy v employ 0.00354995048018099000000

deploy v locate 0.00637346428647210000000

deploy v extend 0.00001261944216082860000

deploy v expand 0.00003630115491672280000

deploy v install 0.00123829518274213000000

deploy v utilize 0.03203281211195150000000

deploy v post 0.00002368171275589420000

deploy v position 0.00692354979533179000000

categorization n classification 1.00000000000000000000000

injury n lesion 0.14486870533478300000000

injury n cramp 0.00055799999321104000000

injury n deformation 0.00010541631513215600000

injury n wound 0.03607749063244630000000

injury n abrasion 0.00003738640720177390000

injury n maltreatment 0.00146753462478963000000

injury n sprain 0.01074478238861980000000

injury n burn 0.01366639351334880000000

injury n trouble 0.00015054628869702300000

injury n mouse 0.00470343914076516000000

injury n pain 0.03558508520077360000000

injury n bruise 0.00000478902427475404000

injury n distress 0.00101913171213623000000

injury n scratch 0.00003262279702397190000

injury n stab wound 0.00138096019945991000000

injury n abuse 0.00197534404042086000000

injury n swelling 0.01171757251556140000000

injury n scar 0.00234954508649433000000

injury n disfigurement 0.00059975824950685900000

injury n puncture 0.00435089870293447000000

injury n wrong 0.00001238919199956170000

injury n destruction 0.00641001632598181000000

injury n affliction 0.00013665751973822100000

injury n loss 0.00825394640178253000000

injury n accidental injury 0.00027688706004269700000

injury n laceration 0.01614412653950650000000

injury n trauma 0.31516831285912000000000

injury n fracture 0.06327797124147160000000

injury n bite 0.00135351177946479000000

injury n hurt 0.00000014245450293536200

injury n insult 0.02221524154070700000000

injury n impairment 0.02521764269325100000000

injury n disadvantage 0.00059011717173997500000

injury n harm 0.00090923677823414200000

injury n contusion 0.00873842782314711000000

injury n disservice 0.00000072559302480409400

injury n suffering 0.00001359133973203940000

injury n damage 0.24041760552619900000000

injury n hemorrhage 0.01945839372546530000000

injury n sting 0.00000034827986093188300

injury n cut 0.00000930598744680821000

indefensible a unwarranted 0.14179573593132800000000

indefensible a weak 0.38635632577804200000000

indefensible a unacceptable 0.47184793829063000000000

helmet n hat 0.21348314606741600000000

helmet n mask 0.78651685393258400000000

equip v suit 0.72972972972973000000000

equip v train 0.27027027027027000000000

impeccable a correct 0.09090909090909090000000

impeccable a ideal 0.09090909090909090000000

impeccable a complete 0.27272727272727300000000

impeccable a right 0.09090909090909090000000

impeccable a upright 0.18181818181818200000000

impeccable a accurate 0.27272727272727300000000

fraction n section 0.00691704783113847000000

fraction n portion 0.38328980042104500000000

fraction n fragment 0.14158567995439100000000

fraction n particle 0.13171604977272500000000

fraction n slice 0.00197629795082749000000

fraction n part 0.16719473887402900000000

fraction n division 0.00019316167930180200000

fraction n end 0.00441501551065102000000

fraction n mite 0.04690609445712090000000

fraction n half 0.10889951105763700000000

fraction n segment 0.00690660249113313000000

climate n air 0.03553835870579490000000

climate n area 0.00621424931559430000000

climate n environment 0.42369620990353200000000

climate n zone 0.13030731525458100000000

climate n mood 0.00029118953129515300000

climate n region 0.32576828813645300000000

climate n altitude 0.07818438915274880000000

bystander n observer 1.00000000000000000000000

shell n structure 0.00065561906491254900000

shell n test 0.54809753826689200000000

shell n case 0.05112795873903360000000

shell n capsule 0.04996016955567110000000

shell n cup 0.35015871437349100000000

package n case 0.00907248803143441000000

package n entity 0.00069300648974352400000

package n range 0.00221855231555893000000

package n combination 0.00353863390572173000000

package n software 0.98447731925754100000000

lump n cyst 0.09316373989143690000000

lump n nodule 0.01395123918416900000000

lump n node 0.00637676726341059000000

lump n enlargement 0.00007214108623361490000

lump n swelling 0.02148629344493530000000

lump n mass 0.69040158612108400000000

lump n growth 0.00031575504297157800000

lump n group 0.01540122843199590000000

lump n tumor 0.06691714370403590000000

lump n spot 0.03099662005170990000000

lump n inflammation 0.00018126678392812800000

lump n majority 0.00031575504297157800000

lump n neoplasm 0.01201914712109400000000

lump n tumour 0.04840131683002340000000

issue n disclosure 0.00000488575387795898000

issue n question 0.20212425267481600000000

issue n effect 0.02148785928181230000000

issue n consequence 0.01374959935527740000000

issue n case 0.03685572026391930000000

issue n number 0.01577448595765180000000

issue n concern 0.09499780750474160000000

issue n conclusion 0.08655321935362290000000

issue n event 0.00714736096286724000000

issue n situation 0.01253712473863800000000

issue n circulation 0.00014787566886970500000

issue n catastrophe 0.00000089661789758392300

issue n effusion 0.00001004376765161240000

issue n return 0.00000069450614377329700

issue n yield 0.00013847737581647500000

issue n termination 0.00007981632973591440000

issue n problem 0.23755023236051900000000

issue n offspring 0.00052838304870279900000

issue n b 0.00053260144327462600000

issue n publication 0.00573565227679831000000

issue n version 0.00000065067854992831400

issue n circumstance 0.00090522581680693600000

issue n emergence 0.00003551907105309650000

issue n impression 0.00048404258172220200000

issue n dissemination 0.00034427020082599900000

issue n delivery 0.00091525458007997800000

issue n opening 0.00000488575387795898000

issue n outcome 0.04087174087288030000000

issue n matter 0.01564701480698570000000

issue n topic 0.03721967693931710000000

issue n outlet 0.00000488575387795898000

issue n family 0.00365637577064910000000

issue n copy 0.00090097289880076300000

issue n release 0.00015251005577422700000

issue n incident 0.00045729892437203200000

issue n proceeds 0.00007166829876824250000

issue n affair 0.00001217530944325360000

issue n presentation 0.00361216085331078000000

issue n subject 0.05743062808742970000000

issue n distribution 0.00032435257177811600000

issue n end 0.00011462790647080100000

issue n point 0.00497945300489457000000

issue n occurrence 0.00439114445984591000000

issue n edition 0.00000312631045519101000

issue n seed 0.00000089661789758392300

issue n appearance 0.00007493510085169650000

issue n result 0.08847598902029380000000

issue n product 0.00227869688597043000000

issue n discharge 0.00009258193477554780000

issue n argument 0.00058024968960625100000

implicit a whole 0.00930297510482876000000

implicit a entire 0.02708582257887390000000

implicit a positive 0.00959760166486558000000

implicit a practical 0.03391948947686540000000

implicit a complete 0.00656244899910691000000

implicit a inherent 0.00847987236921634000000

implicit a tacit 0.82424359428782900000000

implicit a explicit 0.08080819551841460000000

broth n water 1.00000000000000000000000

drainage n effluent 0.07984230613327410000000

drainage n waste 0.00003269814660944010000

drainage n sewage 0.00020972105181764300000

drainage n drain 0.91991527466829900000000

subtract v remove 0.42272393538913400000000

subtract v withdraw 0.03327826725403820000000

subtract v take 0.54399779735682800000000

liberal a extensive 0.08765516521169320000000

liberal a wide 0.07912731022299580000000

liberal a large 0.15083643511258600000000

liberal a broad 0.31565626815976300000000

liberal a flexible 0.00213196374717435000000

liberal a reasonable 0.00426392749434871000000

liberal a rational 0.32003420783276400000000

liberal a abundant 0.02963490348280250000000

liberal a benign 0.01065981873587180000000

jeopardize v endanger 0.93543613255734800000000

jeopardize v compromise 0.03230686882590350000000

jeopardize v expose 0.03225699861674840000000

insoluble a difficult 1.00000000000000000000000

raise v motivate 0.00000051298159898756100

raise v build 0.00002612806502811880000

raise v start 0.00036881174241360100000

raise v propose 0.00836467583200398000000

raise v swell 0.00000668026691616143000

raise v initiate 0.00122573996363315000000

raise v begin 0.00721035848938233000000

raise v lift 0.00715442988600754000000

raise v occasion 0.00020429807212303100000

raise v form 0.00483629439510334000000

raise v present 0.02519851062103720000000

raise v promote 0.00005974751535012180000

raise v tend 0.00091924882068624000000

raise v obtain 0.03345172700320450000000

raise v propagate 0.00000474627197894601000

raise v provide 0.00066598450865739700000

raise v cause 0.00024333980921962300000

raise v grow 0.00054399393062172800000

raise v upgrade 0.00001671109289029720000

raise v shake 0.00001889725208273030000

raise v magnify 0.00000147576867973422000

raise v recruit 0.00022799119133506400000

raise v foster 0.00000474627197894601000

raise v extend 0.00096549943577394400000

raise v restore 0.00029266190660850700000

raise v incite 0.00003461445811279760000

raise v elicit 0.00727668991181839000000

raise v create 0.00519155042134270000000

raise v accelerate 0.00042149231234568600000

raise v rise 0.00087652170911321700000

raise v expand 0.00091273927880531200000

raise v augment 0.00788098663317490000000

raise v invoke 0.00004709356952038920000

raise v allocate 0.00021323278291437800000

raise v arouse 0.00000196654119273472000

raise v ferment 0.00000196654119273472000

raise v double 0.00003429293451921660000

raise v launch 0.00002206330210500670000

raise v train 0.00008265990524805030000

raise v produce 0.02173108648220040000000

raise v solicit 0.00000922605434048751000

raise v acquire 0.00012742558661830900000

raise v introduce 0.00369266722516647000000

raise v inflame 0.00000295153735946844000

raise v advance 0.00731056679133139000000

raise v make 0.01083602591066540000000

raise v encourage 0.00163366923742193000000

raise v prompt 0.00000196654119273472000

raise v amend 0.00000316605002227641000

raise v keep 0.00541648929323563000000

raise v enlarge 0.00081324615935200300000

raise v model 0.00002337574773059430000

raise v construct 0.00014836611127715000000

raise v agitate 0.00000051298159898756100

raise v open 0.00161884359683662000000

raise v evoke 0.00000805533704271266000

raise v instigate 0.00001226893577854140000

raise v elevate 0.37121949276109100000000

raise v ameliorate 0.00002492419003535030000

raise v accumulate 0.00016526607456276000000

raise v amplify 0.00004540319993084460000

raise v establish 0.01013764852897780000000

raise v institute 0.00061016184195908500000

raise v submit 0.00001889725208273030000

raise v prefer 0.00019176007198721800000

raise v sharpen 0.00009594477315829130000

raise v exacerbate 0.00002968745557980550000

raise v provoke 0.00001061334930163090000

raise v honor 0.00000461302717024372000

raise v harvest 0.00024259239599296000000

raise v support 0.01640856036043410000000

raise v prove 0.00661473036857489000000

raise v stimulate 0.00029398783291446900000

raise v exaggerate 0.00024534931224372600000

raise v resuscitate 0.00018676700263031300000

raise v increase 0.32960281489296400000000

raise v get 0.00000673975361363135000

raise v reinforce 0.00001096831263762620000

raise v enhance 0.00436610776400308000000

raise v suggest 0.01434713404367830000000

raise v nurse 0.00014089647388928300000

raise v wean 0.00003159455280094920000

raise v escalate 0.00005161757401192260000

raise v aggravate 0.00005083063204651000000

raise v strengthen 0.00013913810322833900000

raise v mobilize 0.00006050010868838100000

raise v heighten 0.01860768343175380000000

raise v activate 0.00031833722857193700000

raise v collect 0.00820298487245618000000

raise v gather 0.00001702367507356050000

raise v commence 0.00000316605002227641000

raise v intensify 0.00001817189832230760000

raise v effect 0.00003924757644796240000

raise v develop 0.00925869905412939000000

raise v inflate 0.00006401251463749450000

raise v boost 0.00002588466069487460000

raise v stand 0.00000051298159898756100

raise v improve 0.03968854307120980000000

fallopian tube n oviduct 0.77297297297297300000000

fallopian tube n uterine tube 0.22702702702702700000000

epidemic n increase 0.03405934460626460000000

epidemic n outbreak 0.09787303619366140000000

epidemic n disease 0.05331191582606320000000

epidemic n pandemic 0.01632930133670930000000

epidemic n illness 0.07887084220209700000000

epidemic n infection 0.71955555983520400000000

blockage n closure 0.00061446027711464900000

blockage n obstruction 0.56265789776364800000000

blockage n congestion 0.09677749364555730000000

blockage n occlusion 0.33995014831368000000000

irradiation n radiation therapy 0.25744186727552900000000

irradiation n perception 0.00012563467869502600000

irradiation n beam 0.01232624252280610000000

irradiation n radiation 0.37937779220427200000000

irradiation n radiotherapy 0.35072846331869700000000

hierarchal a hierarchical 1.00000000000000000000000

indicator n signal 0.00006009204812120440000

indicator n measure 0.32107582923331600000000

indicator n index 0.40034948060781100000000

indicator n pointer 0.00061872971089368700000

indicator n guide 0.02367667125102630000000

indicator n sign 0.25293272943311200000000

indicator n indicant 0.00128646771571955000000

gaze n regard 1.00000000000000000000000

chair n position 0.50909090909090900000000

chair n chairman 0.38181818181818200000000

chair n director 0.10909090909090900000000

afterward r subsequently 0.01064742605970250000000

afterward r afterwards 0.02490082295471740000000

afterward r thereafter 0.29838743738108500000000

afterward r soon 0.00014854771924031100000

afterward r ultimately 0.00042325165899551900000

afterward r eventually 0.00045038017310591200000

afterward r then 0.00877185075199679000000

afterward r next 0.00303870566007363000000

afterward r later 0.65323157764108300000000

hinder v hamper 0.01765148562571000000000

hinder v impede 0.00216373049605477000000

hinder v block 0.00004328857397721690000

hinder v handicap 0.01767312991269860000000

hinder v stop 0.00002164428698860830000

hinder v restrict 0.02098278346751920000000

hinder v prevent 0.33548644832343300000000

hinder v interfere 0.00918844457228740000000

hinder v shorten 0.00059543081716566500000

hinder v preclude 0.03675377828914960000000

hinder v inhibit 0.00518262325313851000000

hinder v confuse 0.00002164428698860830000

hinder v obviate 0.00002164428698860830000

hinder v delay 0.00004328857397721690000

hinder v control 0.00012986572193165100000

hinder v check 0.00004328857397721690000

hinder v exclude 0.22082568975273800000000

hinder v limit 0.33317179118527600000000

echinococcosis n hydatid disease 0.98055668081860400000000

echinococcosis n hydatidosis 0.01944331918139580000000

complementary dna n cdna 1.00000000000000000000000

britain n great britain 0.06064367413676590000000

britain n uk 0.31732210817022500000000

britain n united kingdom 0.20728386628835100000000

britain n england 0.20733113137508600000000

britain n scotland 0.20741922002957300000000

blade n needle 1.00000000000000000000000

anonymous a indistinguishable 1.00000000000000000000000

strand n cilium 0.25000000000000000000000

strand n edge 0.25000000000000000000000

strand n length 0.25000000000000000000000

strand n ligament 0.25000000000000000000000

sickness n injury 0.08282024737100200000000

sickness n disease 0.40857988703027700000000

sickness n morbidity 0.29136925843186400000000

sickness n illness 0.19738825623422100000000

sickness n infection 0.01984235093263590000000

grail n holy grail 1.00000000000000000000000

palmar a volar 1.00000000000000000000000

legislation n regulation 0.63241395710890600000000

legislation n rule 0.36758604289109400000000

result v issue 0.00008364697250056600000

result v accrue 0.00002274057740085270000

result v ensue 0.00584349384866837000000

result v succeed 0.00006204222559625440000

result v happen 0.00000027238993664512500

result v resolve 0.00326766353794671000000

result v finish 0.00000179201198168705000

result v arise 0.01033516998943670000000

result v rise 0.00321175242745811000000

result v appear 0.01232436220756800000000

result v emerge 0.00226997254534292000000

result v leave 0.00005814232632272780000

result v produce 0.34025335098673200000000

result v attend 0.00002862730867275670000

result v supervene 0.00189774068860659000000

result v evolve 0.00088512554176902000000

result v lead 0.31758380188083300000000

result v come 0.00152043408720726000000

result v terminate 0.00942823628691634000000

result v originate 0.00001012182443021790000

result v conclude 0.01379173658525300000000

result v end 0.00081803620615757100000

result v derive 0.00152908855991324000000

result v proceed 0.00389280141252634000000

result v follow 0.07311858904001360000000

result v occur 0.12234286045699500000000

result v effect 0.01198968744751180000000

result v develop 0.06342871062630180000000

property n holding 0.00000347232135097679000

property n nature 0.02235829575992410000000

property n stock 0.00003014874895303250000

property n field 0.00037395959053371100000

property n section 0.00216422198884322000000

property n subdivision 0.00001781686258050210000

property n power 0.05100526902160430000000

property n grant 0.00001256917219515820000

property n tract 0.00040226358662831000000

property n dimension 0.01692777061015340000000

property n substance 0.06822468755221750000000

property n wealth 0.00001781686258050210000

property n place 0.00023434285743058200000

property n income 0.00003336687384127770000

property n feature 0.10854437199666600000000

property n quality 0.12827612537985200000000

property n trait 0.00015321745693671900000

property n characteristic 0.58101518493454100000000

property n distinction 0.01763457110739950000000

property n claim 0.00009895202248556180000

property n part 0.00215315079395030000000

property n note 0.00005083074548029480000

property n inheritance 0.00016962049715008500000

property n character 0.00009797325670231980000

ether n diethyl ether 1.00000000000000000000000

disabled a impotent 0.00104542862573655000000

disabled a quadriplegic 0.00104542862573655000000

disabled a injured 0.99790914274852700000000

bout n period 0.27336958173221400000000

bout n test 0.05467331486251250000000

bout n session 0.24766181455132500000000

bout n season 0.00032042056917030300000

bout n time 0.00846966234030729000000

bout n encounter 0.00032042056917030300000

bout n trial 0.41518478537530100000000

fatal a critical 0.00843688896934743000000

fatal a final 0.00900631769599168000000

fatal a ill-fated 0.00003543699892919700000

fatal a inevitable 0.00087941542023456200000

fatal a injurious 0.00141523719333797000000

fatal a incurable 0.02660232093645400000000

fatal a climacteric 0.00003543699892919700000

fatal a black 0.00129525838876777000000

fatal a unavoidable 0.00063316272164857600000

fatal a infectious 0.03827578648536490000000

fatal a disastrous 0.03573961540600330000000

fatal a untreatable 0.00003543699892919700000

fatal a lethal 0.54465232085632500000000

fatal a septic 0.02277752059353630000000

fatal a terminal 0.02040681452462900000000

fatal a contagious 0.00003755512341534210000

fatal a crucial 0.00111581441177404000000

fatal a dire 0.00395628780902392000000

fatal a deleterious 0.01156651549593520000000

fatal a suicidal 0.00346651607269163000000

fatal a malignant 0.04880098316679700000000

fatal a harmful 0.02739527514262290000000

fatal a detrimental 0.00707265197186926000000

fatal a dangerous 0.07108662623799970000000

fatal a inoperable 0.00073810220772956200000

fatal a pivotal 0.00003543699892919700000

fatal a hazardous 0.04316397070153530000000

fatal a destructive 0.01142927589273580000000

fatal a infective 0.00000235833466388379000

fatal a catastrophic 0.05990566024384960000000

unintentional a random 0.00013143064529897800000

unintentional a unconscious 0.00013143064529897800000

unintentional a unanticipated 0.00019041278301817700000

unintentional a fortuitous 0.02228078014430930000000

unintentional a unexpected 0.00019041278301817700000

unintentional a accidental 0.81504323184123600000000

unintentional a automatic 0.00002628612905979560000

unintentional a inadvertent 0.13953772993224300000000

unintentional a spontaneous 0.00021379108126832200000

unintentional a involuntary 0.02225449401524950000000

runner n athlete 1.00000000000000000000000

cyst n blister 0.07868369910839500000000

cyst n vesicle 0.11476852306808900000000

cyst n sac 0.02674595933689750000000

cyst n bladder 0.77980181848661800000000

common carotid artery n common carotid 1.00000000000000000000000

attention deficit disorder n adhd 0.00648226597090800000000

attention deficit disorder n minimal brain dysfunction 0.27544566106069200000000

attention deficit disorder n attention deficit hyperactivity disorder 0.71801140879902200000000

attention deficit disorder n hyperkinetic syndrome 0.00006066416937797390000

ige n immunoglobulin e 1.00000000000000000000000

troublesome a refractory 0.00012678562115514400000

troublesome a strenuous 0.00017718942404919900000

troublesome a bothersome 0.43544292203510400000000

troublesome a enigmatic 0.00024416235771503700000

troublesome a worrisome 0.00532538922222505000000

troublesome a painful 0.00529755087126297000000

troublesome a perplexing 0.00283998768679825000000

troublesome a burdensome 0.00030407869684086400000

troublesome a intractable 0.00017729307568572100000

troublesome a arduous 0.00024416235771503700000

troublesome a dangerous 0.07548713461285790000000

troublesome a problematic 0.02166345164960580000000

troublesome a upsetting 0.00030795015674512700000

troublesome a difficult 0.45236194223224000000000

sexual intercourse n coitus 0.55343444751876300000000

sexual intercourse n intercourse 0.44619739215914300000000

sexual intercourse n relation 0.00036816032209390700000

renew v remodel 0.00015907479864479400000

renew v recover 0.00063629919457917600000

renew v prolong 0.00015907479864479400000

renew v restore 0.00031814959728958800000

renew v replace 0.03441597222609830000000

renew v replenish 0.00031814959728958800000

renew v repair 0.00031814959728958800000

renew v continue 0.96272068139829500000000

renew v stimulate 0.00015907479864479400000

renew v reconstruct 0.00015907479864479400000

renew v resuscitate 0.00015907479864479400000

renew v resume 0.00047722439593438200000

meet v correspond 0.00067538168931557400000

meet v adjust 0.00311930551188869000000

meet v acknowledge 0.00002194938521384970000

meet v handle 0.00000832507203504330000

meet v exchange 0.00000358414110522225000

meet v fulfill 0.09892968907080900000000

meet v yield 0.00264238586454311000000

meet v observe 0.01813171650478300000000

meet v confront 0.00001614823811819720000

meet v accommodate 0.00000431393725221607000

meet v settle 0.00002262234029076160000

meet v join 0.00000358414110522225000

meet v link 0.00001208183813572130000

meet v suffer 0.00003156208238126900000

meet v suffice 0.00000053268207903252600

meet v encounter 0.00030070816648905200000

meet v respect 0.00008778598491528120000

meet v converge 0.00000358414110522225000

meet v discharge 0.00309216742780522000000

meet v execute 0.00000358414110522225000

meet v contradict 0.00000021777116859543400

meet v appear 0.00181518779577901000000

meet v concur 0.00000053268207903252600

meet v challenge 0.00034436044261169400000

meet v see 0.00388078782423066000000

meet v dispute 0.00001204650931299960000

meet v approach 0.00025710695563691600000

meet v contend 0.00000021777116859543400

meet v tackle 0.00000914423906804183000

meet v welcome 0.00004649512291337070000

meet v agree 0.00315350467118208000000

meet v counter 0.00000358414110522225000

meet v fill 0.00002122607720376310000

meet v gratify 0.00000964799871819062000

meet v parallel 0.00002254059660406830000

meet v receive 0.01619371674925520000000

meet v fulfil 0.49492110057179800000000

meet v contact 0.00009051269666641150000

meet v satisfy 0.20099142915460500000000

meet v thwart 0.00000502804219255806000

meet v fit 0.05012084260560470000000

meet v answer 0.00023269216792413200000

meet v pay 0.00000970852891838089000

meet v heed 0.00000931686406349754000

meet v face 0.00000556009796281958000

meet v connect 0.00000021777116859543400

meet v cross 0.00001127029830203410000

meet v engage 0.00124426610350672000000

meet v adhere 0.01162684840054270000000

meet v perform 0.01772276486065910000000

meet v modify 0.00037646493375891800000

meet v collect 0.00022512882251257800000

meet v gather 0.00000234824822347007000

meet v play 0.00000401113478282722000

meet v find 0.01462118022900030000000

meet v match 0.01467911281198400000000

meet v reach 0.03515078388395250000000

meet v comply 0.00506808206335687000000

convalescence n rehabilitation 0.00131602784323262000000

convalescence n cure 0.00305752670490175000000

convalescence n improvement 0.00962030349267939000000

convalescence n recuperation 0.00013768068623640100000

convalescence n recovery 0.57402502183134600000000

convalescence n treatment 0.15693556789187900000000

convalescence n hospitalization 0.24418673637366500000000

convalescence n bed rest 0.01072113517606060000000

proliferation n boost 0.00007006621552844090000

proliferation n increase 0.07054351489289140000000

proliferation n spread 0.00007098141969601380000

proliferation n generation 0.01307030888964120000000

proliferation n extension 0.00003058897854607360000

proliferation n expansion 0.03207535148425520000000

proliferation n enlargement 0.00256494249734485000000

proliferation n augmentation 0.00276194743532922000000

proliferation n swelling 0.00558147472899559000000

proliferation n production 0.23522143559884700000000

proliferation n acceleration 0.00002229666691188370000

proliferation n growth 0.62672999338644600000000

proliferation n concentration 0.00751309860086611000000

proliferation n conception 0.00004854213604788140000

proliferation n elevation 0.00369545706865358000000

signal n cue 0.08596925050415990000000

signal n milestone 0.00001136290473389430000

signal n reason 0.00285117224545992000000

signal n evidence 0.02245989373546010000000

signal n stimulus 0.29845215085050700000000

signal n instrument 0.01013855596456020000000

signal n cry 0.00003517560074144680000

signal n signaling 0.12691651033754100000000

signal n light 0.07157380497553400000000

signal n cause 0.00435381028964017000000

signal n demonstration 0.00120274200046550000000

signal n tip 0.00931827860942999000000

signal n monitor 0.06938669283736530000000

signal n wave 0.00705315891790038000000

signal n marker 0.05494677531842360000000

signal n clue 0.00063995363684261800000

signal n sign 0.06871771693249290000000

signal n meter 0.00014917201615949800000

signal n flare 0.00004906764141957090000

signal n pilot 0.00006451059985884480000

signal n warning 0.00001136290473389430000

signal n reminder 0.00119597042520919000000

signal n alarm 0.01980613376541680000000

signal n symptom 0.13610576335548600000000

signal n landmark 0.00021337096391670400000

signal n grip 0.00780349497299617000000

signal n incentive 0.00001136290473389430000

signal n call 0.00019316938047620400000

signal n indication 0.00036961540833507000000

excerpt v select 0.94444444444444500000000

excerpt v cite 0.05555555555555540000000

inadequacy n drawback 0.17845115089080500000000

inadequacy n shortcoming 0.01865931759571780000000

inadequacy n scarcity 0.00054697670771127900000

inadequacy n weakness 0.00321171925112810000000

inadequacy n inability 0.03651729262245670000000

inadequacy n deficiency 0.75298878492535300000000

inadequacy n lack 0.00548026567035102000000

inadequacy n deficit 0.00414449233647696000000

estrange v separate 0.07692307692307690000000

estrange v leave 0.07692307692307690000000

estrange v withdraw 0.30769230769230800000000

estrange v rupture 0.07692307692307690000000

estrange v withhold 0.15384615384615400000000

estrange v wean 0.30769230769230800000000

recognition n appreciation 0.04486035845218920000000

recognition n comprehension 0.03586588542619590000000

recognition n attention 0.28239989745386800000000

recognition n memory 0.00978129496859965000000

recognition n regard 0.01489175748968050000000

recognition n perception 0.09237369284163540000000

recognition n realization 0.00090358258908043800000

recognition n notice 0.00034321421460474500000

recognition n recall 0.00007493814299782010000

recognition n acceptance 0.10938680414386900000000

recognition n admission 0.00608756570785645000000

recognition n identification 0.40119173239372000000000

recognition n verification 0.00183927617570235000000

fulfill v meet 0.90143982274512700000000

fulfill v observe 0.00004750762272858220000

fulfill v conform 0.00425375945046690000000

fulfill v close 0.00009501524545716450000

fulfill v verify 0.00004750762272858220000

fulfill v implement 0.00028504573637149300000

fulfill v achieve 0.00095015245457164500000

fulfill v accomplish 0.00009501524545716450000

fulfill v attain 0.00004750762272858220000

fulfill v render 0.00004750762272858220000

fulfill v do 0.00614431920622997000000

fulfill v fulfil 0.05091007342304830000000

fulfill v conclude 0.00004750762272858220000

fulfill v satisfy 0.03463910592505600000000

fulfill v answer 0.00004750762272858220000

fulfill v perform 0.00080762958638589800000

fulfill v follow 0.00009501524545716450000

farmer n operator 0.91369277358826700000000

farmer n gardener 0.08630722641173320000000

constellation n cluster 0.00187747456986319000000

constellation n configuration 0.36282196062606200000000

constellation n group 0.63520690311691800000000

constellation n body 0.00009366168715742030000

enviable a excellent 0.75000000000000000000000

enviable a good 0.25000000000000000000000

return v deliver 0.00037481742126278200000

return v exchange 0.00000308551003036491000

return v renew 0.00000345309970847018000

return v yield 0.00000431215890411879000

return v generate 0.01661129632069410000000

return v fall 0.11962388460127800000000

return v reconsider 0.00005137507864179630000

return v recover 0.18175283661751500000000

return v reappear 0.00053365571151325800000

return v reverse 0.02107353349453450000000

return v repeat 0.00831505434643988000000

return v retreat 0.00020155692658857400000

return v release 0.00021047217190032100000

return v restore 0.06204285014617490000000

return v give 0.05144246293858750000000

return v relapse 0.01120265907736590000000

return v adjudicate 0.00006009838855529940000

return v reimburse 0.00051402336465196200000

return v replace 0.00079473511778363200000

return v reply 0.00439961251277087000000

return v make 0.00832258394703494000000

return v retire 0.00000163932847785233000

return v recur 0.02664984499791270000000

return v react 0.00021765099878285200000

return v come 0.02091900484135260000000

return v pronounce 0.00000739766893448369000

return v render 0.00000739766893448369000

return v state 0.00164618517150652000000

return v reinstate 0.00000431215890411879000

return v pass 0.00007167400306415340000

return v reoccur 0.00001467438563023720000

return v answer 0.04601578117570130000000

return v pay 0.00000431215890411879000

return v remit 0.00006765887105087150000

return v compensate 0.00003831009979039700000

return v respond 0.17578059826042900000000

return v regress 0.02109224755563500000000

return v revisit 0.02090778355543080000000

return v recall 0.00001462350237423780000

return v regain 0.04966073952203880000000

return v resurface 0.00002934584365976660000

return v report 0.06617607797832900000000

return v reflect 0.00001898654453435600000

return v turn 0.00837011454290759000000

return v score 0.00148899042030167000000

return v rotate 0.00001467438563023720000

return v revert 0.07311758299613180000000

return v reexamine 0.00012403241171443200000

phage n bacteriophage 1.00000000000000000000000

music n air 0.14092803632133200000000

music n sensitivity 0.85675956198880100000000

music n measure 0.00231240168986688000000

hydatid disease n echinococcosis 0.68812787987795900000000

hydatid disease n hydatidosis 0.31187212012204100000000

count n number 0.66065688292431200000000

count n score 0.29057020491495800000000

count n enumeration 0.02208531976556370000000

count n measure 0.00290424198380748000000

count n total 0.02183018888625450000000

count n calculation 0.00090854479985561500000

count n sum 0.00051361208757124800000

count n aggregate 0.00047325685211922100000

count n tally 0.00005774778555783640000

haematoma n hematoma 1.00000000000000000000000

plasma n blood plasma 0.65353986923361400000000

plasma n plasm 0.34646013076638600000000

infarction n infarct 1.00000000000000000000000

human chorionic gonadotrophin n human chorionic gonadotropin 0.40630715390483900000000

human chorionic gonadotrophin n hcg 0.59369284609516100000000

fatigue v drain 0.27722772277227700000000

fatigue v wear 0.72277227722772300000000

cut v neglect 0.00013544616267346300000

cut v issue 0.00002257436044557650000

cut v divide 0.32823120087869200000000

cut v perforate 0.05049363484896100000000

cut v remove 0.00042891284846596500000

cut v distress 0.00002257436044557650000

cut v discount 0.00004514872089115370000

cut v halt 0.00002257436044557650000

cut v diminish 0.00011287180222788500000

cut v tunnel 0.00002257436044557650000

cut v abate 0.00002257436044557650000

cut v eliminate 0.00020316924401019400000

cut v puncture 0.00002257436044557650000

cut v discontinue 0.00013544616267346300000

cut v stop 0.00772305619802060000000

cut v curtail 0.00002257436044557650000

cut v amputate 0.00002257436044557650000

cut v separate 0.00006772308133673090000

cut v make 0.00119644110361559000000

cut v mar 0.00002257436044557650000

cut v disregard 0.00002257436044557650000

cut v affect 0.00765270819105066000000

cut v shorten 0.00006772308133673090000

cut v excise 0.00009029744178230810000

cut v mark 0.00002257436044557650000

cut v switch 0.00004514872089115370000

cut v obstruct 0.00006772308133673090000

cut v pass 0.00002257436044557650000

cut v record 0.00038376412757481100000

cut v harvest 0.00002257436044557650000

cut v interrupt 0.00009029744178230810000

cut v lower 0.00004514872089115370000

cut v injure 0.00013544616267346300000

cut v dissect 0.00006772308133673090000

cut v abstract 0.00002257436044557650000

cut v cast 0.00002257436044557650000

cut v cross 0.00006772308133673090000

cut v shape 0.00763275875623829000000

cut v reduce 0.21988932031355200000000

cut v impair 0.00115129238272443000000

cut v section 0.32820862651824600000000

cut v degrade 0.00002257436044557650000

cut v alter 0.00329585662505427000000

cut v strike 0.00006772308133673090000

cut v turn 0.00002257436044557650000

cut v tear 0.00002257436044557650000

cut v ignore 0.00036118976712923500000

cut v score 0.00004514872089115370000

cut v decrease 0.04145499116324680000000

differentiation n distinction 1.00000000000000000000000

melanoma n malignant melanoma 1.00000000000000000000000

midwife n obstetrician 1.00000000000000000000000

gall bladder n gallbladder 1.00000000000000000000000

regression n withdrawal 0.00886265526684459000000

regression n reversion 0.01706987461774240000000

regression n retirement 0.00417263601767036000000

regression n subsidence 0.00029071876026895100000

regression n reflux 0.01135671082179280000000

regression n deterioration 0.15620059205347000000000

regression n fixation 0.00293530512978074000000

regression n return 0.00456033934820304000000

regression n recidivism 0.00035096938466386200000

regression n reversal 0.34474634654428200000000

regression n rebound 0.00071319382602689600000

regression n relapse 0.44874065822925500000000

arrange v manage 0.05925148873050410000000

arrange v adjust 0.00207482877518548000000

arrange v place 0.00290119874337910000000

arrange v prescribe 0.01025299313744760000000

arrange v resolve 0.00325332239943684000000

arrange v form 0.05916496901261380000000

arrange v present 0.11356172798103900000000

arrange v promote 0.00333818971741666000000

arrange v accommodate 0.00040573391647714500000

arrange v provide 0.04911567952849920000000

arrange v regulate 0.00154049305260890000000

arrange v direct 0.05863471039462790000000

arrange v locate 0.02996235881795240000000

arrange v display 0.00081146783295429200000

arrange v compare 0.02522814907301240000000

arrange v allocate 0.00331210719748578000000

arrange v prepare 0.00194622696908605000000

arrange v set 0.00040573391647714500000

arrange v schedule 0.00031584023555779500000

arrange v plan 0.06097176914495170000000

arrange v class 0.00031584023555779500000

arrange v categorize 0.00081891890057395800000

arrange v distribute 0.23614097611704200000000

arrange v compromise 0.00081146783295429200000

arrange v do 0.00122465281705110000000

arrange v establish 0.05980773370529120000000

arrange v decide 0.00040573391647714500000

arrange v order 0.00268323691028424000000

arrange v determine 0.10067016243960100000000

arrange v assign 0.04138312417097730000000

arrange v adapt 0.00040573391647714500000

arrange v organize 0.00220807146499052000000

arrange v devise 0.00275769480204034000000

arrange v coordinate 0.00031584023555779500000

arrange v stage 0.00245769717705989000000

arrange v design 0.05758239387179580000000

arrange v group 0.00110403573249526000000

arrange v classify 0.00081891890057395800000

arrange v score 0.00163877827648593000000

unlabeled a unlabelled 1.00000000000000000000000

scientist n investigator 1.00000000000000000000000

saturate v infuse 0.15384615384615300000000

saturate v penetrate 0.07692307692307540000000

saturate v fill 0.07692307692307540000000

saturate v transfuse 0.69230769230769600000000

deduct v discount 0.08244274809160300000000

deduct v withhold 0.79389312977099200000000

deduct v reduce 0.12366412213740500000000

decision n determination 0.00360765390930771000000

decision n selection 0.01837183036654420000000

decision n resolution 0.00041478852737216600000

decision n weighing 0.00003649776460452820000

decision n command 0.00078845950212932400000

decision n issue 0.05214343765018760000000

decision n consequence 0.01189007631681250000000

decision n option 0.07122377165664740000000

decision n strength 0.00002404845099161730000

decision n evaluation 0.04531937317204430000000

decision n consideration 0.01671539073788830000000

decision n judgement 0.00066785775990767700000

decision n conclusion 0.12777768347695600000000

decision n edict 0.00022064557692737500000

decision n purpose 0.08288800438311410000000

decision n adjudication 0.00000303292889247251000

decision n declaration 0.00000034837108457027600

decision n persistence 0.00021381089055948100000

decision n opinion 0.00632690638804763000000

decision n judgment 0.01437020049214180000000

decision n outcome 0.19221671136073400000000

decision n disposal 0.00005401060019362760000

decision n finding 0.04502522940233780000000

decision n result 0.13209464839365300000000

decision n call 0.00815023367417294000000

decision n order 0.03096836771668150000000

decision n arrangement 0.00491410243260329000000

decision n choice 0.13357287809746400000000

czechoslovak n czech 1.00000000000000000000000

hunger n craving 0.00045052860742546300000

hunger n thirst 0.00020217279410328200000

hunger n itch 0.00020217279410328200000

hunger n malnutrition 0.00020217279410328200000

hunger n appetite 0.99894295301026500000000

collapse v decline 0.05931180807974740000000

collapse v give 0.02635371626884820000000

collapse v miscarry 0.16363819171587100000000

collapse v deteriorate 0.00741133212721755000000

collapse v fail 0.56574522458840900000000

collapse v abort 0.01204870247223020000000

collapse v subside 0.16363819171587100000000

collapse v break 0.00185283303180439000000

typhoid fever n typhoid 1.00000000000000000000000

reproduction n radiograph 0.01372837430832810000000

reproduction n image 0.00686418715416401000000

reproduction n production 0.97597534496042600000000

reproduction n delivery 0.00171604678854100000000

reproduction n photograph 0.00171604678854100000000

oxyhemoglobin n oxyhaemoglobin 1.00000000000000000000000

crib death n cot death 1.00000000000000000000000

elective a selective 1.00000000000000000000000

lymphopenia n lymphocytopenia 1.00000000000000000000000

intern n resident 0.97376156075675000000000

intern n student 0.01943389004446440000000

intern n novice 0.00671263564133379000000

intern n trainee 0.00009191355745177480000

informant n subject 0.83442330464825500000000

informant n source 0.08673064994615230000000

informant n reporter 0.07884604540559300000000

fissure n rupture 0.02059820869894650000000

fissure n crack 0.00846605274965874000000

fissure n interval 0.01604869129935310000000

fissure n fracture 0.95488704725204200000000

severe a critical 0.00977531911455912000000

severe a acute 0.31594011099113000000000

severe a fatal 0.05700901856964130000000

severe a troublesome 0.00412824177072997000000

severe a unalterable 0.00000307294031335845000

severe a risky 0.00013239324424513600000

severe a caustic 0.00077358468611140900000

severe a strenuous 0.00000206005784436525000

severe a clinical 0.06593938088564890000000

severe a abusive 0.00153595799996033000000

severe a distressing 0.00401373575181697000000

severe a grave 0.00092157479997620000000

severe a close 0.00013534427413509500000

severe a life-threatening 0.05661029604889990000000

severe a unremitting 0.00014600743429936500000

severe a uncertain 0.00504742515676553000000

severe a painful 0.00915869257911225000000

severe a modest 0.00172968309534072000000

severe a freezing 0.00000897894674608909000

severe a cold 0.00484862736142331000000

severe a heavy 0.00496568708973971000000

severe a unrelenting 0.00000136065967809864000

severe a intense 0.01152070604148840000000

severe a powerful 0.00096895553975843600000

severe a distant 0.00086609986582174900000

severe a excruciating 0.00010164427940914200000

severe a burdensome 0.00000754444846703786000

severe a intractable 0.01591162705067930000000

severe a exact 0.00003766797908219000000

severe a extreme 0.00279304316039584000000

severe a strict 0.00019604740962554400000

severe a rigid 0.00049560625906502300000

severe a unbearable 0.00078303741174448400000

severe a arduous 0.00000851611629207480000

severe a exacting 0.00021004553845611900000

severe a sharp 0.00046078739998810000000

severe a dangerous 0.00596648917577842000000

severe a hard 0.00242792107047022000000

severe a rigorous 0.00021431972092469800000

severe a persistent 0.09186109959899520000000

severe a serious 0.27095071786499600000000

severe a hazardous 0.00122231348151802000000

severe a simple 0.00308985233808748000000

severe a violent 0.00002421297248639660000

severe a dreadful 0.00153595799996033000000

severe a tight 0.00574180445607635000000

severe a difficult 0.02131837421094610000000

severe a strong 0.01290505806963450000000

severe a firm 0.00000206005784436525000

severe a overwhelming 0.00555193702389119000000

yield v deliver 0.00000372595026683405000

yield v bestow 0.00098493801690000800000

yield v return 0.00000223557016010042000

yield v generate 0.02167087194196030000000

yield v fall 0.00004094674176357110000

yield v provide 0.52958310257630200000000

yield v cause 0.00566377241948924000000

yield v release 0.00000149038010673361000

yield v permit 0.00929177477543078000000

yield v give 0.12835398855114700000000

yield v discharge 0.00000074519005336679200

yield v grant 0.00000223557016010042000

yield v produce 0.23804521106176300000000

yield v furnish 0.00034394660907619300000

yield v transfer 0.00000149038010673361000

yield v confer 0.00185168347177201000000

yield v agree 0.00000223557016010042000

yield v accept 0.00000624227404791785000

yield v render 0.00722362398065342000000

yield v pay 0.00000074519005336679200

yield v allow 0.01904713228306110000000

yield v fail 0.00002379105620816210000

yield v stretch 0.00000074519005336679200

yield v reproduce 0.00069973346011143700000

yield v realize 0.00001117785080050220000

yield v defer 0.00000074519005336679200

yield v conceive 0.00030305785135384800000

yield v offer 0.03682370709591780000000

yield v admit 0.00000447114032020086000

yield v comply 0.00000149038010673361000

yield v break 0.00000074519005336679200

yield v abandon 0.00000819709058703493000

paramount a excellent 0.00601818850403658000000

paramount a primary 0.25150224781846900000000

paramount a first 0.00155193435600269000000

paramount a important 0.39013866605026100000000

paramount a dominant 0.00036089723481911500000

paramount a prevalent 0.00239248257852951000000

paramount a predominant 0.00022527343319919300000

paramount a main 0.04448697493122710000000

paramount a principal 0.00122824406949173000000

paramount a significant 0.14462084081708400000000

paramount a central 0.13262973379602500000000

paramount a prime 0.02440258847395380000000

paramount a superior 0.00044192793690109700000

ice n distance 1.00000000000000000000000

refined a sophisticated 0.79045188226732600000000

refined a proper 0.14987171791752800000000

refined a precise 0.00537722368889337000000

refined a strict 0.00852239226164233000000

refined a meticulous 0.04577678386461060000000

question n proposal 0.00009884591011711630000

question n issue 0.38710502806491500000000

question n contention 0.00000474729418307492000

question n scrutiny 0.00010162272659417700000

question n essay 0.00006567545646547460000

question n motion 0.00007036962909182000000

question n consideration 0.00802421703866938000000

question n concern 0.06622096111835920000000

question n search 0.00073828424073958100000

question n enigma 0.00253679063546021000000

question n mystery 0.00171083456302805000000

question n examination 0.00932419303048866000000

question n thesis 0.00006473029882138780000

question n quest 0.00086205806044323700000

question n puzzle 0.00009837600831770620000

question n discussion 0.00259082347882228000000

question n query 0.00032462403152489000000

question n proposition 0.00006071568784778330000

question n conundrum 0.00109653021473724000000

question n exploitation 0.00000388882769901383000

question n controversy 0.04682251290335500000000

question n dispute 0.00363457045835478000000

question n problem 0.20921793508274300000000

question n hesitation 0.00023823800671595400000

question n uncertainty 0.01682159400303140000000

question n research 0.02613468396048140000000

question n matter 0.01567971553289050000000

question n topic 0.01548619504268010000000

question n riddle 0.00301967470828425000000

question n analysis 0.01733956654770630000000

question n investigation 0.00966690754594968000000

question n debate 0.01726825819868390000000

question n subject 0.07580926884643520000000

question n challenge 0.03996650533020470000000

question n head 0.00026304654594579300000

question n inquiry 0.00045846057552728900000

question n doubt 0.01403486674643380000000

question n argument 0.00703468364825148000000

mosaic a diverse 1.00000000000000000000000

hardware n kit 1.00000000000000000000000

unrestricted a total 0.07950107176202940000000

unrestricted a free 0.91479924447179100000000

unrestricted a complete 0.00569968376617938000000

cutaneous a dermal 1.00000000000000000000000

sale n purchase 0.98186188459246600000000

sale n demand 0.01769120512779220000000

sale n clearance 0.00044691027974167700000

obliterate v suppress 0.09492686657933250000000

obliterate v delete 0.01046583263176800000000

obliterate v hide 0.00145216817037987000000

obliterate v eliminate 0.54266807505456800000000

obliterate v cancel 0.00151521982411033000000

obliterate v kill 0.03190178753581530000000

obliterate v extirpate 0.01756495260328900000000

obliterate v eradicate 0.15855616448897000000000

obliterate v waste 0.00145216817037987000000

obliterate v obscure 0.03284966606568400000000

obliterate v destroy 0.01756495260328900000000

obliterate v dissolve 0.03510382160796160000000

obliterate v omit 0.05397832466445220000000

milestone n stone 0.00590563249311610000000

milestone n degree 0.07503060879613110000000

milestone n stage 0.77820856643534300000000

milestone n marker 0.13494955978229400000000

milestone n point 0.00590563249311610000000

completely r thoroughly 0.00564676521360688000000

completely r unequivocally 0.00109844429779056000000

completely r conclusively 0.00272035874934539000000

completely r clearly 0.20402911939483900000000

completely r absolutely 0.00593006513943484000000

completely r comprehensively 0.00169089778643465000000

completely r effectively 0.02176651220801470000000

completely r explicitly 0.00000708419927060407000

completely r radically 0.00190202879083197000000

completely r finally 0.00005554012228153590000

completely r fully 0.54896498027829900000000

completely r fundamentally 0.00014771309117429800000

completely r positively 0.00002669140170248850000

completely r wholly 0.00733538630520675000000

completely r perfectly 0.00053403963732246300000

completely r ultimately 0.00081867543443100100000

completely r essentially 0.00846506768553811000000

completely r quite 0.00446813163226459000000

completely r totally 0.07649884261032120000000

completely r entirely 0.08795320555202310000000

completely r extensively 0.01254795675513390000000

completely r solidly 0.00086781441064899900000

completely r exhaustively 0.00000474559903109850000

completely r simply 0.00049589394894228500000

completely r grossly 0.00118047106085488000000

completely r truly 0.00289271470216333000000

completely r altogether 0.00146293774603114000000

completely r really 0.00048791624706097600000

std n dose 0.00326229759246871000000

std n sexually transmitted disease 0.99673770240753100000000

shortage n limitation 0.00349211877194618000000

shortage n scarcity 0.20020757723370400000000

shortage n failure 0.19067388307971800000000

shortage n shortfall 0.10010378861685200000000

shortage n need 0.08935066846642590000000

shortage n paucity 0.12496094240051300000000

shortage n lack 0.06240236173518040000000

shortage n deficit 0.22880865969566100000000

numeric a binary 0.00380634305890166000000

numeric a digital 0.00018299330006270200000

numeric a numerical 0.99601066364103600000000

exploit v use 0.96082537325065000000000

exploit v promote 0.00002219727276182990000

exploit v employ 0.01329622643553600000000

exploit v utilize 0.00817525280712161000000

exploit v advance 0.00000457526861064032000

exploit v deceive 0.00001762200415118960000

exploit v maximize 0.00004439454552365990000

exploit v abuse 0.00001830107444256130000

exploit v abandon 0.00004049834720439130000

exploit v improve 0.01755555899399840000000

undertake v endorse 0.00011152706990285100000

undertake v start 0.05103846764113900000000

undertake v volunteer 0.00005835600806284690000

undertake v initiate 0.35574548350679000000000

undertake v begin 0.03234319260611210000000

undertake v devote 0.00007087076319763170000

undertake v guarantee 0.00004767706162229100000

undertake v attempt 0.03926206093927280000000

undertake v promise 0.00275489780016629000000

undertake v affirm 0.00019466933281607100000

undertake v warrant 0.49006870278033200000000

undertake v launch 0.00729716296128192000000

undertake v embark 0.00005623079333521040000

undertake v tackle 0.00003822446099293400000

undertake v assume 0.00128762251717413000000

undertake v accept 0.00238708179004224000000

undertake v state 0.00040474911659261100000

undertake v try 0.00621033607008942000000

undertake v move 0.00016093027556345800000

undertake v endeavor 0.00003882385406936730000

undertake v engage 0.00002892738454750560000

undertake v commence 0.00082311430169106000000

undertake v offer 0.00933996961741566000000

undertake v assure 0.00023092134779023700000

cf n cystic fibrosis 1.00000000000000000000000

cautiously r carefully 0.97311479502564500000000

cautiously r slowly 0.00054135656603430300000

cautiously r conservatively 0.02634384840832040000000

boy n fellow 0.00112736101816907000000

boy n son 0.20968676648256000000000

boy n young man 0.00842356796195045000000

boy n master 0.00036573345593370500000

boy n puppy 0.03699703265042860000000

boy n youngster 0.00449092185175738000000

boy n ad 0.02505305158635710000000

boy n youth 0.70582973286079600000000

boy n schoolboy 0.00331136371832727000000

boy n lad 0.00071892096171583800000

boy n page 0.00399554745200492000000

optical density n absorbance 1.00000000000000000000000

beneficent a benign 1.00000000000000000000000

bearable a tolerable 1.00000000000000000000000

effect n capacity 0.00256269740475528000000

effect n determination 0.00197874682805281000000

effect n value 0.01587021066804980000000

effect n meaning 0.00013182462320593400000

effect n issue 0.00132165686918984000000

effect n property 0.02109077852092930000000

effect n decision 0.00248610393859930000000

effect n consequence 0.00810445218259897000000

effect n inference 0.00027322237036517300000

effect n strength 0.00087674105422504600000

effect n core 0.00059694521365017300000

effect n execution 0.00002770813520874410000

effect n validity 0.00053544060355160700000

effect n outgrowth 0.00008705095954618810000

effect n power 0.00064792933432928200000

effect n reliability 0.00090287859671814700000

effect n conclusion 0.34114708170456800000000

effect n event 0.02831636847442040000000

effect n drift 0.00000118266430769046000

effect n efficacy 0.08480893115670070000000

effect n gist 0.00000309615127303361000

effect n burden 0.00077659842850698600000

effect n sequel 0.00012944273860015800000

effect n completion 0.00073427904101836400000

effect n realization 0.00000083288007068696800

effect n essence 0.00004683700142661060000

effect n purpose 0.00920410689314504000000

effect n force 0.00059046511796874000000

effect n punch 0.00000044867766861488100

effect n accomplishment 0.00000051262914087841000

effect n repercussion 0.00001126556512627780000

effect n fulfillment 0.00000228184642895562000

effect n import 0.00002403757391928660000

effect n potency 0.00188874674793043000000

effect n object 0.00164485418107124000000

effect n vigor 0.00000498212634506203000

effect n finish 0.00009697847323060440000

effect n operation 0.00493798175292696000000

effect n intention 0.00001614436009771700000

effect n feedback 0.00028472515473288300000

effect n sensation 0.00021819034874070800000

effect n capability 0.00022161533046986200000

effect n impression 0.00006041408127269780000

effect n production 0.00483306215328379000000

effect n performance 0.00642602572908907000000

effect n outcome 0.04273204129292150000000

effect n side effect 0.00104340677217334000000

effect n impact 0.06026032214547940000000

effect n bearing 0.00048200934506214700000

effect n aftereffect 0.00000157226977541194000

effect n ability 0.01181784497096050000000

effect n achievement 0.00028443608506862600000

effect n weight 0.00215202437278065000000

effect n intent 0.00003959391217568900000

effect n action 0.03690717754147310000000

effect n sense 0.00010152433916329000000

effect n feeling 0.00002421496311733750000

effect n reaction 0.01008563498564040000000

effect n end 0.00259600072327538000000

effect n implication 0.01444800293088150000000

effect n effectiveness 0.03611698888854930000000

effect n significance 0.00605849775018723000000

effect n impingement 0.00033719933638010600000

effect n influence 0.05716174592985620000000

effect n result 0.16957509439654000000000

effect n success 0.00382685126491109000000

effect n reality 0.00002193949717044770000

kg n kilogram 0.99955209026062700000000

kg n kilo 0.00044790973937321400000

mrna n messenger rna 1.00000000000000000000000

convalescent a ambulatory 1.00000000000000000000000

clarification n disclosure 0.00723361006305844000000

clarification n interpretation 0.04671303938942770000000

clarification n elucidation 0.34152037783981100000000

clarification n definition 0.25268650836324000000000

clarification n specification 0.01750871579223040000000

clarification n light 0.02723241435504350000000

clarification n demonstration 0.01322717268673540000000

clarification n description 0.01857665361007530000000

clarification n explanation 0.03408228545048390000000

clarification n presentation 0.00984688760929632000000

clarification n answer 0.22079203638627600000000

clarification n enucleation 0.00630854718294201000000

clarification n manifestation 0.00427175127137938000000

bacteriemia n bacteremia 1.00000000000000000000000

spine n resolution 0.08079039803899030000000

spine n needle 0.19546801242440600000000

spine n spur 0.00012402542745099600000

spine n spinal column 0.71097416038900400000000

spine n back 0.01264340372014940000000

productive a worthwhile 0.00211149172489561000000

productive a pregnant 0.00165794298828743000000

productive a effective 0.75864111992793400000000

productive a helpful 0.02207910400807180000000

productive a valuable 0.15685106548610400000000

productive a rewarding 0.00054892396419585700000

productive a fruitful 0.00088171792584428900000

productive a useful 0.01812547250582320000000

productive a fat 0.00013751951476589800000

productive a plentiful 0.00006406353074553980000

productive a advantageous 0.01988781876816610000000

productive a beneficial 0.01901375965516650000000

el n elevation 1.00000000000000000000000

autonomy n self-sufficiency 1.00000000000000000000000

yearly r per annum 0.00498570591063293000000

yearly r annually 0.99501429408936700000000

suit v adjust 0.00357394447294690000000

suit v equip 0.51631584485839700000000

suit v change 0.03585526700405530000000

suit v tailor 0.15185760142894000000000

suit v design 0.25268073878501500000000

suit v comply 0.03971660345064590000000

seat v place 0.00370517102534014000000

seat v situate 0.87048361776585000000000

seat v position 0.10551316578980000000000

seat v take 0.01576725043500410000000

seat v establish 0.00272854387017030000000

seat v contain 0.00092629275633503600000

seat v conduct 0.00087595835750022600000

plastic surgery n reconstructive surgery 1.00000000000000000000000

certain a confident 0.00240988397690722000000

certain a reliable 0.03870333047508960000000

certain a particular 0.08184648583899620000000

certain a specific 0.27389117129570000000000

certain a sanguine 0.00057184228383165600000

certain a inevitable 0.00109567122761209000000

certain a true 0.00339018876613843000000

certain a absolute 0.00024307326593884900000

certain a regular 0.00764662560092790000000

certain a special 0.03222477888325970000000

certain a evident 0.01940434878115040000000

certain a positive 0.01344206356902510000000

certain a decisive 0.00064771416854918600000

certain a clear 0.02736562649020500000000

certain a definite 0.01595143562069840000000

certain a secure 0.00002065395707448760000

certain a constant 0.00092896207334230100000

certain a actual 0.00132480770032949000000

certain a fixed 0.00016972856825181000000

certain a precise 0.00782654206446527000000

certain a undisturbed 0.00001138985349401200000

certain a dependable 0.00004791862941454500000

certain a solid 0.00044044563461344700000

certain a singular 0.00315504915774440000000

certain a invariable 0.00039773373528799800000

certain a valid 0.00086214753558312700000

certain a plain 0.00046362698380362800000

certain a obvious 0.00633103669601565000000

certain a several 0.30927279472311500000000

certain a infallible 0.00008179401702017370000

certain a real 0.00004880873806840920000

certain a cogent 0.00057184228383165600000

certain a safe 0.01168972487672830000000

certain a undisputed 0.00002186351654834550000

certain a appreciable 0.00020376247386578000000

certain a sound 0.00002726467234005740000

certain a individual 0.07117089160213010000000

certain a stable 0.03882868276135090000000

certain a trustworthy 0.00002726467234005740000

certain a firm 0.00015118841478698300000

certain a conclusive 0.01464829248353650000000

certain a marked 0.01157790498863670000000

certain a unequivocal 0.00075225839977891200000

certain a genuine 0.00011137854247227700000

dissatisfaction n anxiety 0.20164620531762500000000

dissatisfaction n discomfort 0.79835379468237500000000

letter n writing 0.00001930419338186870000

letter n type 0.02605588916750930000000

letter n correspondence 0.02223053260140680000000

letter n reply 0.05226159671704380000000

letter n report 0.09265189970173910000000

letter n communication 0.03140984301489960000000

letter n bulletin 0.01885524308332150000000

letter n learning 0.00264750408262771000000

letter n face 0.00226224197451504000000

letter n postcard 0.01884266033852120000000

letter n matrix 0.00008506844396623560000

letter n figure 0.00015336237395890500000

letter n jet 0.00000330776096524553000

letter n literature 0.03785106789627220000000

letter n sign 0.04488028467640760000000

letter n culture 0.02428025637812610000000

letter n semantics 0.00322609184583772000000

letter n note 0.01897452521119010000000

letter n study 0.52011155719443800000000

letter n answer 0.00222850521282787000000

letter n cap 0.00032121749657869000000

letter n message 0.00012078628422129000000

letter n mail 0.01679619558014330000000

letter n standard 0.03123554078186350000000

letter n line 0.03248199519482590000000

letter n body 0.00001352279341073710000

singly r independently 0.30572461100947400000000

singly r separately 0.58109358688325600000000

singly r individually 0.03695868659415540000000

singly r only 0.07164433255968520000000

singly r respectively 0.00457878295342848000000

right hemisphere n right brain 1.00000000000000000000000

atypical a divergent 0.00085770265693645600000

atypical a peculiar 0.00162021997679432000000

atypical a exceptional 0.08020858231606690000000

atypical a different 0.21700932776663800000000

atypical a odd 0.14924026230694300000000

atypical a abnormal 0.53803092982289000000000

atypical a irregular 0.00333933538987586000000

atypical a aberrant 0.00296867376662840000000

atypical a anomalous 0.00672496599722753000000

georgia n ga 1.00000000000000000000000

flow v leak 0.50000000000000000000000

flow v circulate 0.50000000000000000000000

crack n fissure 0.50834460983310800000000

crack n report 0.49165539016689200000000

colorado n co 1.00000000000000000000000

labor n childbirth 0.00122224962368356000000

labor n function 0.00105868215084903000000

labor n parturition 0.02508319126710970000000

labor n work 0.00540252476789802000000

labor n effort 0.00117189246025039000000

labor n role 0.00784957244476474000000

labor n business 0.00011204967594462700000

labor n confinement 0.00001146777067237120000

labor n task 0.00000511843945933103000

labor n labour 0.52806437937703900000000

labor n living 0.00000057898497689199800

labor n project 0.00003440074108708560000

labor n exercise 0.00360712956775649000000

labor n charge 0.00134459611133552000000

labor n operation 0.00758484851030424000000

labor n performance 0.00260400827014912000000

labor n delivery 0.24754369634471900000000

labor n industry 0.00000094415596337086100

labor n employment 0.00015604775636293300000

labor n achievement 0.00000094415596337086100

labor n stress 0.00015103915519991400000

labor n occupation 0.00001804826995081240000

labor n exertion 0.00134459611133553000000

labor n birth 0.16123488771590800000000

labor n energy 0.00018075234954690400000

labor n activity 0.00416239330987150000000

labor n strain 0.00004996051189850550000

rest n remission 0.00645987578221542000000

rest n sleep 0.01721616359114150000000

rest n brace 0.00020540609264485000000

rest n somnolence 0.00001507726738250630000

rest n cornerstone 0.00011255646595651900000

rest n lethargy 0.00037196284559515200000

rest n splint 0.00001920177714174600000

rest n inactivity 0.00028734833112880400000

rest n relief 0.16185132088190600000000

rest n immobility 0.00208006577928558000000

rest n demise 0.00483500551743556000000

rest n mortality 0.09175887619242160000000

rest n excess 0.03612997071686430000000

rest n holiday 0.00011080519182903100000

rest n death 0.12725110279819100000000

rest n unemployment 0.00001307647477330030000

rest n balance 0.05352489935882490000000

rest n cessation 0.04814935122788900000000

rest n domain 0.00036463794350420600000

rest n relaxation 0.00753217967089971000000

rest n nap 0.01167148020599130000000

rest n stability 0.00010479769742029800000

rest n residuum 0.00002258996168901540000

rest n quiescence 0.00108292084385486000000

rest n camp 0.00003431666836129420000

rest n decompression 0.01244172576051340000000

rest n surplus 0.00041792327448329100000

rest n coma 0.00210011622056344000000

rest n suspension 0.00003030678645525720000

rest n gap 0.00010118423008087000000

rest n remainder 0.09517096043535900000000

rest n remains 0.00161605110544495000000

rest n interval 0.07497775178263650000000

rest n residue 0.00001307647477330030000

rest n interruption 0.00442648375788631000000

rest n remnant 0.00002720624756641330000

rest n framework 0.00011368324875965500000

rest n status quo 0.00003030678645525720000

rest n ease 0.00007886135274318440000

rest n release 0.01346544096955820000000

rest n rib 0.00029803170472478700000

rest n catalepsy 0.00002125522498055880000

rest n frame 0.00756005934322073000000

rest n vegetation 0.00300688617198181000000

rest n end 0.13712406483750000000000

rest n interim 0.00293488016151075000000

rest n comfort 0.00004523180214751890000

rest n mainstay 0.00011255646595651900000

rest n stay 0.01831526043736910000000

rest n support 0.02771374527148600000000

rest n home 0.01706552522087000000000

rest n break 0.00954770423216902000000

rest n stop 0.00002716403461440950000

rest n impasse 0.00001156737384141850000

ireland n eire 1.00000000000000000000000

grave n rest 0.01730136364588020000000

grave n pit 0.00432534091147006000000

grave n demise 0.01730136364588020000000

grave n death 0.71022354911164800000000

grave n end 0.17739998634880700000000

grave n hole 0.00856828266426270000000

grave n home 0.06488011367205120000000

obese a full 0.00007134171521212680000

obese a large 0.02297473288635420000000

obese a porcine 0.00000338980714466615000

obese a heavy 0.12660004750119100000000

obese a fat 0.00068273349548279300000

obese a overweight 0.64580690510987400000000

obese a round 0.00010362892441296000000

obese a great 0.20370422629660200000000

obese a big 0.00003218864914726570000

obese a bulky 0.00002080561457881710000

agreeable a good 1.00000000000000000000000

screen v conceal 0.00000737976853831291000

screen v sort 0.00002113551753738490000

screen v hide 0.00003099565052348030000

screen v divide 0.00078862808111318400000

screen v flank 0.00072252158100023200000

screen v shield 0.00000201384150789782000

screen v examine 0.11879126808006200000000

screen v protect 0.00039006377016625900000

screen v inspect 0.00000737976853831291000

screen v eliminate 0.00212471498918971000000

screen v grade 0.00039643562089964500000

screen v choose 0.00327431124143430000000

screen v secrete 0.00001493372117606930000

screen v purify 0.00002493819349506860000

screen v separate 0.00266969272692421000000

screen v investigate 0.06780852722355290000000

screen v test 0.19630225142620200000000

screen v clarify 0.00396677692264061000000

screen v guard 0.00002494639729796200000

screen v cover 0.00011464791866702600000

screen v interview 0.00714857396835545000000

screen v analyze 0.10067264943217400000000

screen v blind 0.00013489952206869800000

screen v show 0.02775144413977710000000

screen v transmit 0.00011960464013382600000

screen v select 0.12991407964249700000000

screen v safeguard 0.00006337036260372000000

screen v strain 0.00980139730136514000000

screen v filter 0.00004976551705868940000

screen v order 0.00329425745616145000000

screen v reinforce 0.00009559646404368210000

screen v check 0.00149949298592192000000

screen v mask 0.00005811475248069970000

screen v evaluate 0.32029735695293400000000

screen v reject 0.00014390443568529300000

screen v obscure 0.00026183662440124600000

screen v fortify 0.00000559772817417682000

screen v refine 0.00019844824242103400000

screen v clean 0.00091473245165488300000

screen v secure 0.00009131493962087420000

premature infant n preterm infant 1.00000000000000000000000

license v release 0.17465240871698200000000

license v permit 0.00119471232483012000000

license v warrant 0.00456946672692758000000

license v approve 0.74832797652942800000000

license v allow 0.07001014223504520000000

license v document 0.00124529346678775000000

survival n selection 0.36515485010133100000000

survival n maintenance 0.15625088650214500000000

survival n living 0.03561507181862580000000

survival n remainder 0.13113597397507300000000

survival n remnant 0.00540070637000181000000

survival n continuance 0.00603608359000206000000

survival n endurance 0.02294633162726740000000

survival n durability 0.27746009601555400000000

wound n lesion 0.09641723449975580000000

wound n injury 0.47828266605765400000000

wound n abrasion 0.00018701758199122100000

wound n grief 0.00000952459169045392000

wound n shock 0.00336679906136950000000

wound n pain 0.06598229097855430000000

wound n tear 0.03098793447985540000000

wound n bruise 0.00004170159937366780000

wound n distress 0.00005153199047803220000

wound n scratch 0.02036340695219610000000

wound n scar 0.00572243833141566000000

wound n sore 0.00051228696735084400000

wound n puncture 0.00039129436256556900000

wound n stab 0.00001250206428165920000

wound n laceration 0.00455258742895795000000

wound n trauma 0.29159473521880800000000

wound n hurt 0.00000566559798878315000

wound n harm 0.00000566559798878315000

wound n contusion 0.00012906061234260400000

wound n damage 0.00137386717331543000000

wound n cut 0.00000978885206673434000

absurd a impractical 0.16153661464585800000000

absurd a inappropriate 0.20000000000000000000000

absurd a unwise 0.16153661464585800000000

absurd a meaningless 0.08076830732292920000000

absurd a untenable 0.08076830732292920000000

absurd a inconclusive 0.23462184873949600000000

absurd a impossible 0.08076830732292920000000

court n attention 0.08570058384883640000000

court n place 0.04611409090143000000000

court n courtroom 0.45376265447007100000000

court n trial 0.41442267077966200000000

unproven a unproved 1.00000000000000000000000

terminus n conclusion 0.02702702702702700000000

terminus n end 0.97297297297297300000000

presymptomatic a preclinical 1.00000000000000000000000

freeze v block 0.00007361652238714350000

freeze v stop 0.03506081774917020000000

freeze v ice 0.01529015169980990000000

freeze v restrict 0.00022624608323484400000

freeze v depress 0.00110521518712747000000

freeze v cool 0.00064711846255115600000

freeze v anesthetize 0.00015802607692111200000

freeze v fix 0.56286762237914800000000

freeze v arrest 0.30587665051858500000000

freeze v limit 0.07865772705987160000000

freeze v immobilize 0.00003680826119357120000

door n bar 0.00251245527230392000000

door n access 0.98739492201544300000000

door n course 0.01009262271225320000000

staining n spotting 1.00000000000000000000000

mathematical a statistical 0.76726125134988700000000

mathematical a particular 0.11147481665531400000000

mathematical a careful 0.03049449940922900000000

mathematical a differential 0.01723282189840610000000

mathematical a scientific 0.05816077390712050000000

mathematical a rigid 0.01537583678004330000000

listen v accept 0.06139595944861770000000

listen v receive 0.44743636496243900000000

listen v adopt 0.02046531981620590000000

listen v monitor 0.14325723871344100000000

listen v admit 0.32744511705929600000000

hay fever n pollinosis 1.00000000000000000000000

favour n favor 1.00000000000000000000000

vignette n scene 0.00041378693699972900000

vignette n illustration 0.00028900788962951800000

vignette n view 0.00033434583125372200000

vignette n instance 0.00040268349656910300000

vignette n design 0.41825205451214000000000

vignette n picture 0.01902254313193120000000

vignette n scenario 0.56128557820147700000000

unsuitable a inadequate 0.28774787703792800000000

unsuitable a impractical 0.15755653612571200000000

unsuitable a inappropriate 0.19009282747151500000000

unsuitable a unfeasible 0.02043942897901660000000

unsuitable a inapplicable 0.03562836244048770000000

unsuitable a unacceptable 0.08629981124473690000000

unsuitable a difficult 0.22223515670060400000000

tl n thallium 1.00000000000000000000000

theme n story 0.00043926953547100400000

theme n question 0.00018456029121466000000

theme n case 0.52424919959020200000000

theme n concept 0.01816986975368370000000

theme n report 0.03215445237035300000000

theme n paper 0.03641532740200570000000

theme n feature 0.05891439977480850000000

theme n problem 0.23190171580719400000000

theme n topic 0.00046087038237224000000

theme n subject 0.07898556482837840000000

theme n point 0.01777051946838300000000

theme n line 0.00006152009707155310000

theme n strain 0.00029273069886179900000

scene n position 0.00017363669150289200000

scene n sequence 0.00034727338300578400000

scene n view 0.60216087014403800000000

scene n focus 0.00017363669150289200000

scene n disturbance 0.00017363669150289200000

scene n event 0.00175149137797657000000

scene n situation 0.00017363669150289200000

scene n setting 0.00376753057790654000000

scene n extract 0.00017363669150289200000

scene n perspective 0.20509658788960700000000

scene n exposure 0.00761501700553929000000

scene n site 0.00455298847436862000000

scene n stage 0.00455298847436862000000

scene n episode 0.00638373596662281000000

scene n center 0.06466233240268500000000

scene n performance 0.00551902195588312000000

scene n part 0.00017363669150289200000

scene n region 0.00069454676601156900000

scene n incident 0.00074035219896111100000

scene n spot 0.00091398889046400300000

scene n segment 0.01809496461847280000000

scene n background 0.07193085303356960000000

scene n location 0.00017363669150289200000

remove v suppress 0.01397035891559030000000

remove v delete 0.00008974572362121710000

remove v purge 0.00003347131137243460000

remove v isolate 0.00877297430025664000000

remove v divide 0.02039158724557780000000

remove v subtract 0.00014519312095339900000

remove v raise 0.01366145752994760000000

remove v cut 0.00001404096073048170000

remove v obliterate 0.00313707562267432000000

remove v segregate 0.00005945269450283010000

remove v lift 0.00018739443286515900000

remove v dissociate 0.00038875768884600700000

remove v dislocate 0.00004118335247533980000

remove v discard 0.00614661074421981000000

remove v evacuate 0.01145208266928040000000

remove v diminish 0.00565752788436489000000

remove v sequester 0.00001288284286636870000

remove v eliminate 0.02777325703776100000000

remove v retreat 0.00118085609772550000000

remove v elicit 0.00104710970490876000000

remove v cancel 0.00036624229354253200000

remove v exfoliate 0.00005316753676299650000

remove v ejaculate 0.00002368322766469830000

remove v extrude 0.00005325986921952150000

remove v discharge 0.00968219299689260000000

remove v stop 0.01210285571987020000000

remove v execute 0.00009593854890452560000

remove v dismiss 0.00052191092963242000000

remove v kill 0.01685240126448370000000

remove v dislodge 0.01331227095601380000000

remove v curtail 0.00006123949776824190000

remove v amputate 0.00026939266805688900000

remove v separate 0.00319867163670051000000

remove v deter 0.00003563612255572640000

remove v resect 0.15405134964509400000000

remove v leave 0.01688546717295720000000

remove v extract 0.01165384536857350000000

remove v insulate 0.00001221983616781510000

remove v detach 0.00005325986921952150000

remove v retire 0.00005945269450283010000

remove v abolish 0.01611618183268160000000

remove v expel 0.00005325986921952150000

remove v prevent 0.02879337642708670000000

remove v transfer 0.00171064346291382000000

remove v withdraw 0.04310606542889470000000

remove v shorten 0.00101601501757133000000

remove v evoke 0.00014851968205671300000

remove v transplant 0.00444360217927024000000

remove v excise 0.27206091257478600000000

remove v extirpate 0.00061681816283927800000

remove v nullify 0.00047006272908662800000

remove v take 0.10594167337621400000000

remove v divorce 0.00001288284286636870000

remove v drop 0.00173831869779256000000

remove v switch 0.00097428841567956400000

remove v preclude 0.00031456935118725900000

remove v eradicate 0.02466827343169810000000

remove v shift 0.00024111996282641500000

remove v neutralize 0.01128946697291800000000

remove v displace 0.00122865591093846000000

remove v proscribe 0.00005945269450283010000

remove v shed 0.00004758031424092260000

remove v quit 0.00007507916843464170000

remove v can 0.02458569719505230000000

remove v obviate 0.01081531788585850000000

remove v end 0.00000077108446609383200

remove v migrate 0.00061424379518602900000

remove v abstract 0.00025756992983454200000

remove v transport 0.00005129789959827100000

remove v erase 0.00005945269450283010000

remove v lessen 0.00004316214442115380000

remove v sacrifice 0.00227332881929676000000

remove v exclude 0.08209975445104110000000

remove v disengage 0.00003937440568227750000

remove v uncouple 0.00013685110397448500000

remove v expose 0.00672027157507773000000

remove v destroy 0.00332017856423506000000

remove v uncover 0.00028113138099306300000

remove v counteract 0.00001404096073048170000

remove v truncate 0.00005325986921952150000

paradox n inconsistency 0.00089152996412870700000

paradox n difficulty 0.00785350742531178000000

paradox n anomaly 0.00704710858710637000000

paradox n enigma 0.03969001118785660000000

paradox n reversal 0.00218625934736012000000

paradox n conundrum 0.04648418428393610000000

paradox n problem 0.78202517618964700000000

paradox n confusion 0.00129472938323141000000

paradox n uncertainty 0.02076995226422940000000

paradox n dilemma 0.09175754136719260000000

worthy a good 1.00000000000000000000000

hand-held a handheld 1.00000000000000000000000

frail a susceptible 0.27252556595505200000000

frail a weak 0.35735725961559600000000

frail a slight 0.04466965745194950000000

frail a ill 0.00863345054267678000000

frail a unstable 0.04572740890345280000000

frail a vulnerable 0.26964774910749300000000

frail a insubstantial 0.00143890842377943000000

place v localize 0.00061591785162800200000

place v remember 0.00003651323699536050000

place v arrange 0.00002515335830932440000

place v seat 0.00007898981454435110000

place v target 0.00252374031804384000000

place v prescribe 0.00474092596567797000000

place v situate 0.00824785313533933000000

place v settle 0.00003724359966287630000

place v employ 0.04011860136106660000000

place v house 0.00002100471935994730000

place v direct 0.01418948352005750000000

place v locate 0.03404944178100920000000

place v aim 0.01237177970300900000000

place v attribute 0.00063062626908042800000

place v grade 0.00021413610442229700000

place v install 0.00333486924175906000000

place v allocate 0.07935663068961930000000

place v overwhelm 0.00002265290561938980000

place v set 0.00830304779032632000000

place v leave 0.00003090249516409860000

place v rate 0.00001276406187118330000

place v retain 0.00090251425136805600000

place v class 0.00001095332421691810000

place v site 0.00066874484881129700000

place v categorize 0.00832888265959021000000

place v stick 0.00002022485050129860000

place v post 0.00000159687379193402000

place v nail 0.00000425468729039434000

place v position 0.13075421577916100000000

place v point 0.00000355153716176516000

place v distribute 0.00152277399886454000000

place v store 0.00012562703089473600000

place v allot 0.00046685961143430200000

place v establish 0.01950269002064810000000

place v send 0.00003112731112679610000

place v fix 0.01275750388603100000000

place v pose 0.00000159687379193402000

place v rest 0.00000177576858088258000

place v specify 0.00016228451919963700000

place v order 0.00022055964476689300000

place v determine 0.03702108865905760000000

place v assign 0.36499807784753600000000

place v know 0.01884929854988550000000

place v range 0.00000752397488609955000

place v put 0.02092510888039790000000

place v deposit 0.00001839821832899550000

place v charge 0.00000355153716176516000

place v group 0.02245097041835130000000

place v assort 0.00012371779703009000000

place v recognize 0.01490997203891250000000

place v identify 0.09574536399237860000000

place v classify 0.04049513691769540000000

place v stand 0.00000177576858088258000

walker n ambulator 0.04225352112676060000000

walker n stroller 0.95774647887323900000000

vague a unproven 0.00111974569566847000000

vague a doubtful 0.00087580574238944600000

vague a unclear 0.07264031119392580000000

vague a imprecise 0.00095169348145866600000

vague a speculative 0.00073181985983708700000

vague a nonspecific 0.59289243277502400000000

vague a enigmatic 0.00213885472847414000000

vague a superficial 0.00058680486456983000000

vague a uncertain 0.02451611356500560000000

vague a unspecific 0.10186932448932300000000

vague a weak 0.00047183568545309200000

vague a dubious 0.00024393995327902900000

vague a undetermined 0.00121969976639514000000

vague a low 0.01588161609375600000000

vague a equivocal 0.00024393995327902900000

vague a undecided 0.00024393995327902900000

vague a conjectural 0.00024393995327902900000

vague a problematic 0.00684828931811309000000

vague a questionable 0.00146363971967417000000

vague a unreliable 0.00094367137090618300000

vague a obscure 0.17002614743548300000000

vague a undefined 0.00384643440142734000000

primarily r overall 0.00323889160363048000000

primarily r chiefly 0.01777820461391560000000

primarily r mainly 0.27874281168755700000000

primarily r predominantly 0.17158661119794300000000

primarily r predominately 0.02468378822427950000000

primarily r fundamentally 0.00754226862408540000000

primarily r largely 0.11349154230229800000000

primarily r mostly 0.10811823495610100000000

primarily r essentially 0.02828350734032020000000

primarily r originally 0.00613660772288065000000

primarily r particularly 0.07095006820363410000000

primarily r especially 0.00565670146806405000000

primarily r principally 0.04525361174451240000000

primarily r initially 0.07220425740967950000000

primarily r generally 0.04633289290109930000000

tall a untrue 0.00009395990537319500000

tall a large 0.14075057865014100000000

tall a excessive 0.00028187971611958500000

tall a high 0.41441687720655400000000

tall a unlikely 0.00075167924298556200000

tall a dubious 0.00009395990537319500000

tall a impossible 0.00037583962149278000000

tall a difficult 0.00339004366808867000000

tall a great 0.43984518208387200000000

wide a extensive 0.03147361477266900000000

wide a open 0.01074616470309080000000

wide a liberal 0.00021702201043536700000

wide a full 0.03691639582597900000000

wide a radical 0.05368200183899540000000

wide a large 0.33843432230838000000000

wide a comprehensive 0.00326314899762240000000

wide a exhaustive 0.00016144320288484600000

wide a massive 0.00241005724921943000000

wide a ample 0.00882556175770494000000

wide a widespread 0.20689044430792200000000

wide a extended 0.00399990473799292000000

wide a broad 0.23209439555422100000000

wide a composite 0.00012201237453048300000

wide a far 0.00950392188927852000000

wide a distant 0.00037823836104449700000

wide a universal 0.00529533705462298000000

wide a thick 0.01617613322164980000000

wide a general 0.03935281801004820000000

wide a progressive 0.00005706182170929920000

polio n poliomyelitis 1.00000000000000000000000

pernicious anemia n pernicious anaemia 1.00000000000000000000000

human n people 0.01988213652232310000000

human n man 0.52713465793336800000000

human n one 0.09144438423616340000000

human n woman 0.22380433617936700000000

human n individual 0.09829728136779140000000

human n person 0.03868974015683290000000

human n character 0.00074746360415396300000

ldl n beta-lipoprotein 0.02218595889453860000000

ldl n low-density lipoprotein 0.97781404110546100000000

propose v mean 0.00002790935173044820000

propose v suit 0.00025912759645116200000

propose v volunteer 0.00000057924526340618500

propose v urge 0.00082648974762116800000

propose v present 0.10603513320641900000000

propose v purport 0.00606554293557489000000

propose v aim 0.03299802440762150000000

propose v affirm 0.00011412101382780700000

propose v ask 0.00694580498833084000000

propose v solicit 0.00002005406479905200000

propose v introduce 0.03644458616602910000000

propose v advance 0.10302632284936000000000

propose v plan 0.00348955304699134000000

propose v contend 0.00000423076849380214000

propose v state 0.00226683127402458000000

propose v submit 0.00976003506772548000000

propose v prefer 0.01057448343487360000000

propose v request 0.00073559394901899500000

propose v project 0.00082350870206787100000

propose v advise 0.00890563371733820000000

propose v move 0.00000920442677501445000

propose v maintain 0.00943292901539495000000

propose v proffer 0.00315811758174853000000

propose v invite 0.00014085969915079600000

propose v suggest 0.41023064824810000000000

propose v recommend 0.08772905222256790000000

propose v counsel 0.00000481001375720833000

propose v intend 0.00050374164744072500000

propose v design 0.08706735403428020000000

propose v tender 0.00001391001859756410000

propose v offer 0.07238580755862550000000

abrasion n ulcer 0.00786200711190730000000

abrasion n erosion 0.98492244384890700000000

abrasion n laceration 0.00721554903918613000000

unworkable a infeasible 1.00000000000000000000000

untrue a erroneous 0.03225806451612900000000

untrue a spurious 0.06451612903225800000000

untrue a deceptive 0.03225806451612900000000

untrue a unfounded 0.06451612903225800000000

untrue a false 0.12903225806451600000000

untrue a incorrect 0.35483870967742000000000

untrue a inaccurate 0.16129032258064500000000

untrue a misleading 0.16129032258064500000000

own v see 0.34188034188034200000000

own v have 0.65811965811965800000000

statistical a mathematical 0.13754898674453700000000

statistical a demographic 0.76828772692516900000000

statistical a analytical 0.09416328633029340000000

hitherto r before 0.00081738509168302000000

hitherto r yet 0.17213026948410600000000

hitherto r heretofore 0.01236421692419640000000

hitherto r previously 0.80754533291771800000000

hitherto r formerly 0.00714279558229621000000

dummy n sham 1.00000000000000000000000

block n snag 0.00004111668651312580000

block n bar 0.00003332230191967060000

block n structure 0.00876788512585202000000

block n drawback 0.00010145205351636900000

block n complex 0.00396661914725283000000

block n closure 0.00033616429421776200000

block n blockade 0.87116212032108000000000

block n section 0.03552599349894430000000

block n lift 0.00002515613651713710000

block n development 0.00972073841308585000000

block n cul-de-sac 0.00201471763914316000000

block n barrier 0.00171475797926382000000

block n pulley 0.00011782630391414100000

block n charge 0.00003346223776455810000

block n mass 0.00045083910932765700000

block n blocking 0.01309566465443060000000

block n hindrance 0.00000540752127776632000

block n slice 0.00008367682862748310000

block n table 0.00002250290731540780000

block n obstruction 0.04444304050109120000000

block n restraint 0.00001469652822730200000

block n occlusion 0.00669633040920048000000

block n segment 0.00157145039648163000000

block n building 0.00005505900503645080000

army n host 0.00539065907104450000000

army n score 0.00572754240842671000000

army n detail 0.00046398083628610600000

army n mass 0.00024851187720115900000

army n herd 0.00046398083628610600000

army n group 0.97615256286290200000000

army n cohort 0.00223234518763404000000

army n military 0.00767996253415981000000

army n unit 0.00147477980125871000000

army n point 0.00008283729240038620000

army n body 0.00008283729240038620000

unalterable a unavoidable 1.00000000000000000000000

overproduction n excess 1.00000000000000000000000

multifaceted a complex 0.77811499017490100000000

multifaceted a multifarious 0.10800123095769500000000

multifaceted a multiplex 0.02155580124052760000000

multifaceted a difficult 0.09232797762687640000000

jugular vein n jugular 1.00000000000000000000000

gynecologist n gynaecologist 1.00000000000000000000000

evenly r equally 0.82782093377112200000000

evenly r equivalently 0.10330743973732700000000

evenly r uniformly 0.06887162649155120000000

distress v worry 0.00007507467308813210000

distress v depress 0.75087399208321600000000

distress v vex 0.00096423204242789800000

distress v torture 0.00007507467308813210000

distress v embarrass 0.22349980427247800000000

distress v strain 0.00131754043925475000000

distress v try 0.00007507467308813210000

distress v injure 0.02217205345202890000000

distress v disturb 0.00072192967206457400000

distress v stress 0.00022522401926440100000

day-to-day a daily 1.00000000000000000000000

phone n telephone 1.00000000000000000000000

offend v harm 1.00000000000000000000000

farm n field 1.00000000000000000000000

educator n mentor 0.62737457379444700000000

educator n doctor 0.03507062834875790000000

educator n reader 0.03507062834875790000000

educator n guide 0.30248416950803700000000

surveillance n command 0.00000174074647112548000

surveillance n management 0.04960753826634160000000

surveillance n regulation 0.00191235736708535000000

surveillance n attention 0.01003335316869580000000

surveillance n scrutiny 0.00380975640807209000000

surveillance n leadership 0.00002438605267680400000

surveillance n administration 0.00285699915145417000000

surveillance n regard 0.00192365439842976000000

surveillance n probe 0.00301653662797185000000

surveillance n examination 0.08885374837019620000000

surveillance n exploration 0.00001561938990561960000

surveillance n review 0.01348828855031460000000

surveillance n direction 0.00967594980449128000000

surveillance n care 0.05282817609208160000000

surveillance n guidance 0.00242695774687717000000

surveillance n charge 0.00006068208920531970000

surveillance n overview 0.00043948538322833400000

surveillance n note 0.00021741718747377300000

surveillance n survey 0.07660916501411090000000

surveillance n observation 0.03718001164934520000000

surveillance n control 0.07337563500861300000000

surveillance n study 0.24176322594312200000000

surveillance n inspection 0.00070538915335495900000

surveillance n monitoring 0.13415310680328000000000

surveillance n supervision 0.01135533032984400000000

surveillance n treatment 0.18366298132960200000000

surveillance n conduct 0.00000250796775620013000

cheap a prudent 0.00005174260718103110000

cheap a economical 0.02628694092688810000000

cheap a bad 0.00132235987071873000000

cheap a common 0.32735583321849400000000

cheap a uncomfortable 0.00005174260718103110000

cheap a inferior 0.00030990637911110500000

cheap a affordable 0.00005918733930828920000

cheap a modest 0.00053268605377460400000

cheap a substandard 0.00005918733930828920000

cheap a reasonable 0.00023674935723315700000

cheap a standard 0.00809850200272816000000

cheap a poor 0.00569278333351609000000

cheap a low 0.15459523472684700000000

cheap a inexpensive 0.47439010451246900000000

cheap a second 0.00060191568939195700000

cheap a moderate 0.00023674935723315700000

cheap a worthless 0.00011837467861657800000

generalist n nonspecialist 1.00000000000000000000000

generate v start 0.00085282049925941000000

generate v return 0.00993297664996460000000

generate v arrange 0.00051143783768583600000

generate v yield 0.02499532149455290000000

generate v initiate 0.00150591283144288000000

generate v form 0.01684267504257390000000

generate v bear 0.00005817984656543050000

generate v propagate 0.00119013061779596000000

generate v cause 0.00835673873945026000000

generate v create 0.04763764648272450000000

generate v give 0.03153021035388000000000

generate v launch 0.00001401422186123130000

generate v fabricate 0.00004459008559092800000

generate v produce 0.13361198024299000000000

generate v imagine 0.00000369005506772701000

generate v trigger 0.01251229043142680000000

generate v make 0.03517406872356740000000

generate v evolve 0.00030817610706944800000

generate v prompt 0.01249895000769540000000

generate v visualize 0.00116778455449672000000

generate v construct 0.17373489425077300000000

generate v achieve 0.03528744891802430000000

generate v accomplish 0.00249406045421523000000

generate v terminate 0.00013437776963537300000

generate v render 0.01273822767309180000000

generate v establish 0.01904828156135780000000

generate v precipitate 0.00016857998258900400000

generate v institute 0.00114920985501588000000

generate v bring 0.01249637148685760000000

generate v envisage 0.00003485475707127730000

generate v provoke 0.01250152852853310000000

generate v proliferate 0.00058394259284381100000

generate v found 0.00000738011013545403000

generate v devise 0.00955507720152218000000

generate v get 0.00000257852083778607000

generate v perform 0.07083373664324400000000

generate v induce 0.11929727895119500000000

generate v reproduce 0.00033739047394019800000

generate v design 0.00113777387249077000000

generate v effect 0.00018246383536187600000

generate v develop 0.18952494773560300000000

drawback n block 0.00117616712336116000000

drawback n shortcoming 0.01973604300300070000000

drawback n difficulty 0.14263403348805600000000

drawback n trouble 0.00078461168128808500000

drawback n fault 0.00027857313821879700000

drawback n barrier 0.02340045434789670000000

drawback n problem 0.47673797702785200000000

drawback n interference 0.00087094796808322000000

drawback n pitfall 0.04353271759191340000000

drawback n obstruction 0.00268914583454994000000

drawback n defect 0.00213634957446860000000

drawback n disadvantage 0.28160760150913100000000

drawback n liability 0.00147400349834218000000

drawback n handicap 0.00266355018121482000000

drawback n obstacle 0.00027782403262360200000

catheterisation n catheterization 1.00000000000000000000000

blister n bulla 0.13547970813635300000000

blister n pustule 0.00033194962790677700000

blister n swelling 0.33956110054531200000000

blister n growth 0.00014445519064865500000

blister n tumor 0.14883040936656100000000

blister n vesicle 0.03662115716442980000000

blister n wheal 0.33869927034088200000000

blister n bubble 0.00033194962790677700000

alertness n activity 1.00000000000000000000000

accentuate v emphasize 0.00527651278969844000000

accentuate v highlight 0.01582953836909540000000

accentuate v mark 0.24613577260197600000000

accentuate v exaggerate 0.71835466379494800000000

accentuate v stress 0.00785419341080076000000

accentuate v strengthen 0.00654931903348136000000

observe v peruse 0.00001319950143999180000

observe v distinguish 0.00207347564571504000000

observe v remember 0.00018041897707933200000

observe v acknowledge 0.00002242232608968710000

observe v witness 0.00008524235704850610000

observe v meet 0.00208551935415809000000

observe v fulfill 0.00000059969234841804500

observe v conform 0.00000006126936658988890

observe v watch 0.00071429951186181200000

observe v discern 0.00036577450695642500000

observe v convey 0.00000490394753016025000

observe v address 0.00207275858937178000000

observe v present 0.03164148096024300000000

observe v allege 0.00015928316584883300000

observe v examine 0.03871179002209170000000

observe v detect 0.06916325705736210000000

observe v honour 0.00000046378097165418200

observe v explore 0.00307081771277741000000

observe v inspect 0.00004328182120101020000

observe v impart 0.00002585340346513390000

observe v respect 0.00009947595099577500000

observe v discharge 0.00319286498520315000000

observe v execute 0.00000092728820509283900

observe v mind 0.00000006126936658988890

observe v catch 0.00000351109161453223000

observe v scrutinize 0.00014319556855288700000

observe v declare 0.00001337622049582330000

observe v investigate 0.02697227865236660000000

observe v contemplate 0.00005399030421349150000

observe v enunciate 0.00002053064392819430000

observe v see 0.14357008627769100000000

observe v perceive 0.00077786012146406900000

observe v scan 0.00019070824002001100000

observe v voice 0.00000957181469123011000

observe v review 0.02468219177860260000000

observe v comment 0.00001546395140072650000

observe v attend 0.00001222641253750030000

observe v consider 0.01623782156729180000000

observe v keep 0.00011493114666710100000

observe v discover 0.00272822168172712000000

observe v survey 0.00104992170144782000000

observe v accept 0.00041923376730110700000

observe v view 0.00008171578424603240000

observe v mark 0.00102156116398673000000

observe v hold 0.00007074240618365480000

observe v pronounce 0.00159494039797658000000

observe v state 0.00052164697930253800000

observe v look 0.00052212641472890600000

observe v fulfil 0.00045966078862343900000

observe v satisfy 0.00055647251274933100000

observe v adopt 0.00076185106010622000000

observe v honor 0.00001352683644283290000

observe v heed 0.00001165791663229070000

observe v interrupt 0.00041856425347053000000

observe v note 0.13331070047820600000000

observe v read 0.00000379983794038632000

observe v breathe 0.00000606992950920526000

observe v maintain 0.01450627398495040000000

observe v express 0.00294072892836729000000

observe v say 0.00020530904125425100000

observe v suggest 0.01036720299752600000000

observe v adhere 0.00002331606486042430000

observe v check 0.00036579774779023400000

observe v perform 0.05550164495435280000000

observe v follow 0.05158724989162920000000

observe v find 0.24998222917979200000000

observe v study 0.06822997711542100000000

observe v mention 0.00049475837872171300000

observe v tell 0.00017370075216354000000

observe v monitor 0.02328831749147410000000

observe v recognize 0.00605223385165804000000

observe v offer 0.00197515773176665000000

observe v regard 0.00116747469566772000000

observe v reflect 0.00032539394107638300000

observe v notice 0.00268267805560219000000

observe v comply 0.00003216439711055330000

texture n structure 0.14111365369946600000000

texture n composition 0.25400457665903900000000

texture n consistency 0.25400457665903900000000

texture n surface 0.35087719298245600000000

misinterpret v misunderstand 0.50000000000000000000000

misinterpret v misrepresent 0.50000000000000000000000

participate v use 0.11383896812677300000000

participate v join 0.05040181353425670000000

participate v enter 0.58624505453254500000000

participate v cooperate 0.00019270962520244000000

participate v concur 0.00001803471096907630000

participate v share 0.02935278029581220000000

participate v work 0.00212015714235594000000

participate v contribute 0.03808876911856310000000

participate v engage 0.00978426009860408000000

participate v perform 0.15528106266701300000000

participate v play 0.01467639014790610000000

hydatidiform mole n molar pregnancy 1.00000000000000000000000

herald v signify 0.11809544176952200000000

herald v indicate 0.35428632530856600000000

herald v portend 0.23619088353904400000000

herald v predict 0.23619088353904400000000

herald v advertise 0.00807490200133485000000

herald v disseminate 0.02422470600400450000000

herald v publicize 0.00403745100066742000000

herald v distribute 0.01211235300200230000000

herald v show 0.00274960283514603000000

herald v publish 0.00403745100066742000000

cleanse v sterilize 0.00032992411745298500000

cleanse v restore 0.00032992411745298500000

cleanse v purify 0.00016496205872649300000

cleanse v clear 0.00016496205872649300000

cleanse v clarify 0.00032992411745298500000

cleanse v brush 0.00016496205872649300000

cleanse v refine 0.00016496205872649300000

cleanse v clean 0.99835037941273500000000

assemble v form 0.19910290471508600000000

assemble v convene 0.00649719413501006000000

assemble v produce 0.00432414609661142000000

assemble v make 0.03391313212180040000000

assemble v combine 0.08532981630646550000000

assemble v construct 0.05119788978387930000000

assemble v collect 0.57133502081862000000000

assemble v gather 0.04829989602252760000000

unremarkable a routine 1.00000000000000000000000

before r hitherto 0.00059430293654325600000

before r heretofore 0.00118860587308651000000

before r first 0.00168243303555380000000

before r since 0.05710882396371040000000

before r already 0.00312427201588853000000

before r previously 0.76110351272132800000000

before r beforehand 0.00012709840184553000000

before r ago 0.04729994039181780000000

before r early 0.12777101066022600000000

topical a limited 0.00095657572831411200000

topical a relevant 0.00001539574389060460000

topical a particular 0.01266579992572280000000

topical a up-to-date 0.00000513191463020144000

topical a subjective 0.00005351472605953050000

topical a modern 0.01684293099498880000000

topical a current 0.16069197085897600000000

topical a recent 0.02420858700294960000000

topical a contemporary 0.03509361512309320000000

topical a local 0.68508197725314600000000

topical a popular 0.00203806328854297000000

topical a familiar 0.00010705779156689500000

topical a regional 0.03829394717608770000000

topical a well-known 0.00002052765852080620000

topical a immediate 0.02392490481351010000000

outlook n probability 0.00121433502225933000000

outlook n interpretation 0.00094848711492006900000

outlook n possibility 0.00865213703359773000000

outlook n light 0.02768683850751270000000

outlook n apprehension 0.00015624626697242000000

outlook n exposure 0.00014805795993322300000

outlook n attitude 0.00104085859050800000000

outlook n mood 0.00659210440655065000000

outlook n direction 0.03460854813439090000000

outlook n vision 0.00148603744622806000000

outlook n chance 0.07277050681078680000000

outlook n future 0.00031249253394483900000

outlook n picture 0.00666613338651726000000

outlook n disposition 0.00015624626697242000000

outlook n prognosis 0.83464840242957900000000

outlook n risk 0.00276127388983190000000

outlook n hope 0.00015129419949460500000

staunch v stop 1.00000000000000000000000

relevant a congruent 0.00000098054244208553000

relevant a important 0.35688256883965300000000

relevant a suitable 0.00351410894969611000000

relevant a proper 0.00041322048015889400000

relevant a related 0.02042164478227210000000

relevant a compatible 0.00006884338000818590000

relevant a applicable 0.00997221166794586000000

relevant a significant 0.45150463936321800000000

relevant a congruous 0.00000353969126393093000

relevant a timely 0.00047520355218272800000

relevant a relative 0.00011299395702082400000

relevant a pertinent 0.10645858681701600000000

relevant a appropriate 0.02773452003887220000000

relevant a approximate 0.00000447320569986880000

relevant a consistent 0.01282867724152680000000

relevant a comparable 0.00950618859346617000000

relevant a analogous 0.00009759889755741710000

soil n dust 1.00000000000000000000000

responsible a reliable 0.04232583146454420000000

responsible a able 0.00201962197406411000000

responsible a obligatory 0.00019006307469219000000

responsible a effective 0.28893971197085400000000

responsible a important 0.63470693866198300000000

responsible a binding 0.01166444010100030000000

responsible a sensible 0.00001283658034362750000

responsible a efficient 0.00070524167593750800000

responsible a dependable 0.00001283658034362750000

responsible a mature 0.00076645191628749500000

responsible a guilty 0.00001283658034362750000

responsible a reasonable 0.00395852067863047000000

responsible a rational 0.00136344321428472000000

responsible a subject 0.00108765835021574000000

responsible a ethical 0.00237305941819433000000

responsible a upright 0.00001339393150681820000

responsible a sound 0.00046749658132754800000

responsible a stable 0.00752390143684508000000

responsible a trustworthy 0.00001283658034362750000

responsible a culpable 0.00055661256668573600000

responsible a firm 0.00001283658034362750000

responsible a imperative 0.00125389653513055000000

responsible a competent 0.00001953354609703660000

press n strain 1.00000000000000000000000

nickel n coin 1.00000000000000000000000

decline n regression 0.00457478492048430000000

decline n fall 0.15054307980181200000000

decline n subsidence 0.00004304693255182900000

decline n reduction 0.18327383414047300000000

decline n deterioration 0.16891803419444300000000

decline n atrophy 0.00481227149985536000000

decline n weakness 0.00018087605926365400000

decline n decrease 0.47038836970762400000000

decline n debility 0.01005283494582900000000

decline n degradation 0.00086595909946056100000

decline n degeneration 0.00036396650538530800000

decline n erosion 0.00023244146148940800000

decline n drop 0.00406893168360153000000

decline n dissolution 0.00049809295319831500000

decline n decay 0.00012565525798724600000

decline n shrinkage 0.00073162732284991700000

decline n diminution 0.00032619351369207100000

gravity n consequence 0.00209533787323206000000

gravity n seriousness 0.00104766893661603000000

gravity n severity 0.40020953378732300000000

gravity n pressure 0.59559979046621300000000

gravity n importance 0.00104766893661603000000

similar a correlative 0.00003452009350340520000

similar a identical 0.08603081741399770000000

similar a congruent 0.00284720424965202000000

similar a alike 0.00186393741860922000000

similar a common 0.03409398680535290000000

similar a close 0.00396410599040112000000

similar a exchangeable 0.00000163871286984207000

similar a equivalent 0.05915176167610960000000

similar a consanguineous 0.00000180829977656751000

similar a interchangeable 0.00003555193295075180000

similar a related 0.00099958518971913000000

similar a same 0.17834231221607100000000

similar a parallel 0.00281747607845046000000

similar a complementary 0.00047216509414670900000

similar a like 0.00000608840137409484000

similar a homogeneous 0.00560422172528808000000

similar a collateral 0.00024786885450090100000

similar a congruous 0.00006265037220130470000

similar a concordant 0.00310770665299282000000

similar a matching 0.00047138296806521800000

similar a comparable 0.61617941859822500000000

similar a reciprocal 0.00063703194971046800000

similar a analogous 0.00302675930603114000000

goal n mark 0.00000270451468442781000

goal n ideal 0.00000006099905063192260

goal n aspiration 0.00006384708379961450000

goal n purpose 0.39764309619190800000000

goal n endpoint 0.00300745461823259000000

goal n plan 0.00096144365446138800000

goal n object 0.00577511104266614000000

goal n intention 0.00041601838627682100000

goal n aim 0.27154834076576500000000

goal n intent 0.00109313835447030000000

goal n tape 0.00000085910648127461500

goal n objective 0.31077530336436300000000

goal n end 0.00037474628943245500000

goal n target 0.00793015211980575000000

goal n line 0.00024897504485752000000

goal n home 0.00015874846374465400000

conform v correspond 0.09513090816628530000000

conform v observe 0.00007503095026698510000

conform v mind 0.00007503095026698510000

conform v concur 0.00007503095026698510000

conform v agree 0.00030012380106794000000

conform v accept 0.00007503095026698510000

conform v fit 0.71303162554553800000000

conform v adhere 0.00007503095026698510000

conform v follow 0.18996169253150300000000

conform v alter 0.00067527855240286600000

conform v match 0.00007503095026698510000

conform v comply 0.00045018570160191000000

transportation n transmission 0.00013586624654991000000

transportation n transfer 0.01109752448645610000000

transportation n transport 0.98767967929459500000000

transportation n cost 0.00108692997239930000000

sidestep v circumvent 1.00000000000000000000000

starvation n lack 1.00000000000000000000000

protrude v start 0.50000000000000000000000

protrude v extend 0.50000000000000000000000

characteristic a peculiar 0.02880474698497830000000

characteristic a particular 0.01594815044361050000000

characteristic a diagnostic 0.08341624832344000000000

characteristic a essential 0.01278596919045690000000

characteristic a discriminative 0.00003955745144237250000

characteristic a typical 0.18513140634664900000000

characteristic a specific 0.20334016760915600000000

characteristic a unique 0.07323543372044300000000

characteristic a idiosyncratic 0.00132119807144512000000

characteristic a usual 0.01020070685086930000000

characteristic a regular 0.00038927759976180100000

characteristic a special 0.00932362254499496000000

characteristic a inbred 0.00005236248473221190000

characteristic a original 0.00019395716458439600000

characteristic a natural 0.00003090169503545660000

characteristic a personal 0.00008105012054282390000

characteristic a fixed 0.00008247705928477500000

characteristic a native 0.00016495411856955000000

characteristic a representative 0.00096951427354087900000

characteristic a indicative 0.02636740916833670000000

characteristic a distinctive 0.20198012827997600000000

characteristic a normal 0.09302797195657670000000

characteristic a inherent 0.00045045163147838700000

characteristic a appropriate 0.00401473383964737000000

characteristic a symptomatic 0.01019577273353510000000

characteristic a individualistic 0.00010647038562216400000

characteristic a individual 0.01051775981029490000000

characteristic a discriminating 0.00062727559602663900000

characteristic a exclusive 0.01757003024690570000000

characteristic a marked 0.00963029429806301000000

preferably r first 1.00000000000000000000000

irrigate v douche 1.00000000000000000000000

deficient a scarce 0.04981635236963280000000

deficient a scant 0.00001028121603476020000

deficient a bad 0.06663138175541750000000

deficient a inadequate 0.05927135543590520000000

deficient a inferior 0.00011201969491328700000

deficient a incomplete 0.00085538290562913100000

deficient a weak 0.00851562819267028000000

deficient a insufficient 0.26664077541351300000000

deficient a substandard 0.00004411374858794740000

deficient a defective 0.34052708058897200000000

deficient a meager 0.00030881903791851900000

deficient a poor 0.05678606328481420000000

deficient a short 0.14152933936455300000000

deficient a unsatisfactory 0.00057750024567990900000

deficient a rare 0.00837390674575797000000

ball n balloon 0.33333333333333300000000

ball n sphere 0.29385470192588900000000

ball n spheroid 0.03947863140744440000000

ball n testis 0.03947863140744440000000

ball n pill 0.29385470192588900000000

attenuate v mitigate 0.01335998286955420000000

attenuate v cut 0.00000642786423123680000

attenuate v weaken 0.01320711837378150000000

attenuate v diminish 0.06302565527281730000000

attenuate v constrict 0.00037607532417687600000

attenuate v shrink 0.00002868028788029880000

attenuate v abate 0.00000642786423123680000

attenuate v extend 0.00032456873375647700000

attenuate v deflate 0.00000642786423123680000

attenuate v disable 0.00062898089899647400000

attenuate v lower 0.01929260186563910000000

attenuate v reduce 0.61673480230369700000000

attenuate v impair 0.03939012730450980000000

attenuate v debilitate 0.00003147345101262220000

attenuate v refine 0.00002689713256567900000

attenuate v thin 0.00038144782880797700000

attenuate v decrease 0.23317230476011100000000

volunteer v propose 0.00003964367284170620000

volunteer v present 0.00608843206819559000000

volunteer v extend 0.00003964367284170620000

volunteer v give 0.00443129459912486000000

volunteer v grant 0.00011893101852512100000

volunteer v advance 0.00007928734568341360000

volunteer v do 0.19262923745192300000000

volunteer v suggest 0.00011893101852512100000

volunteer v perform 0.60393170927539500000000

volunteer v tell 0.00011893101852512100000

volunteer v offer 0.19240395885841900000000

sulfur dioxide n sulphur dioxide 1.00000000000000000000000

papanicolaou test n pap test 1.00000000000000000000000

maintenance n extension 0.14148790837548200000000

maintenance n preservation 0.21629200539267500000000

maintenance n care 0.06309732197962130000000

maintenance n prolongation 0.00187175872574012000000

maintenance n persistence 0.00582330099978213000000

maintenance n continuity 0.00617192391645346000000

maintenance n conservation 0.00034848474915405100000

maintenance n continuation 0.51399898011997900000000

maintenance n food 0.02084789069064190000000

maintenance n service 0.02913274760571180000000

maintenance n support 0.00092767744475949300000

sawbones n surgeon 1.00000000000000000000000

fibroid n fibroid tumor 1.00000000000000000000000

fibreoptic a fiberoptic 1.00000000000000000000000

emphasise v underline 0.05538150766525920000000

emphasise v emphasize 0.72815448360247000000000

emphasise v underscore 0.21589234331894900000000

emphasise v stress 0.00057166541332135000000

yet r hitherto 0.02623222252593790000000

yet r heretofore 0.02895642529765680000000

yet r moreover 0.01043060038661720000000

yet r too 0.00187722260688726000000

yet r already 0.03714550927096800000000

yet r additionally 0.00734386452173886000000

yet r nevertheless 0.01312631518120490000000

yet r regardless 0.00019302641694230800000

yet r even 0.03555194148286220000000

yet r also 0.14566993823258500000000

yet r nonetheless 0.00281058235335846000000

yet r still 0.18020838856674100000000

yet r however 0.48847508219041300000000

yet r furthermore 0.02197888096608680000000

deliberate v evaluate 1.00000000000000000000000

snack n bite 1.00000000000000000000000

variability n variance 1.00000000000000000000000

neuroticism n neurosis 1.00000000000000000000000

insidious a subtle 1.00000000000000000000000

ineligible a unsuitable 0.01636274100409980000000

ineligible a inappropriate 0.75046477188173200000000

ineligible a objectionable 0.00114150230687098000000

ineligible a unacceptable 0.00114150230687098000000

ineligible a unavailable 0.22671235009003600000000

ineligible a undesirable 0.00417713241039022000000

record n writing 0.00007102617807809540000

record n story 0.00006142067882946020000

record n work 0.00050321032162853600000

record n book 0.00000813045713462094000

record n manuscript 0.00088639350158496800000

record n evidence 0.01405206987491160000000

record n registry 0.01576446894585210000000

record n recording 0.03227834557585940000000

record n entry 0.00068631794806582300000

record n instrument 0.00070032934796563500000

record n cassette 0.00001732575922460280000

record n administration 0.00109025898117673000000

record n case history 0.00166181671153873000000

record n policy 0.00180098513472028000000

record n report 0.07514445390720560000000

record n schedule 0.00002564818187201140000

record n lp 0.00002694080128872810000

record n birth certificate 0.00016542684506816000000

record n accomplishment 0.00002183200176256070000

record n index 0.01597070982205370000000

record n film 0.00307376526446721000000

record n enrolment 0.00002184580165340410000

record n reputation 0.00008361829186058310000

record n disc 0.00000195695740940988000

record n log 0.00629627629152577000000

record n history 0.02290424181576910000000

record n enrollment 0.00147642525647725000000

record n account 0.00404221155801584000000

record n life 0.00707005730878874000000

record n transcript 0.00101895578555272000000

record n list 0.00001566508250907370000

record n disk 0.00000413908343069764000

record n inventory 0.00000413472879268533000

record n journal 0.00555970702180577000000

record n experience 0.12991318794270900000000

record n course 0.03269364447752890000000

record n transcription 0.00000983744610124506000

record n diary 0.08644094287027610000000

record n register 0.00359434614299314000000

record n life history 0.00000262441383468942000

record n release 0.00005628816134460490000

record n data 0.38925329436152000000000

record n photograph 0.00124111604901914000000

record n file 0.04485605693923910000000

record n tape 0.00043859609755018100000

record n campaign 0.00000532167869661124000

record n treatment 0.03828739579522860000000

record n track 0.00000253634057273143000

record n cd 0.00001416237736017080000

record n videotape 0.00174460160760411000000

record n career 0.00262003981163160000000

record n background 0.05625880740807180000000

record n cut 0.00000458137124409930000

record n document 0.00002962663855955210000

record n conduct 0.00000103504338094479000

record n calendar 0.00002184580165340410000

corp n corporation 1.00000000000000000000000

contradictory a discrepant 0.04954634759752580000000

contradictory a negative 0.13290445799189600000000

contradictory a ambivalent 0.00010865020774608000000

contradictory a adverse 0.05250881181222620000000

contradictory a inconsistent 0.63720454346072500000000

contradictory a opposite 0.03652727868697530000000

contradictory a paradoxical 0.00022464637287357600000

contradictory a contrary 0.09097526387003230000000

causation n origin 0.29228309294968400000000

causation n reason 0.00056289473846833700000

causation n causality 0.00494222929029859000000

causation n cause 0.67560038598636300000000

causation n production 0.02661139703518650000000

protective a prophylactic 0.42080661820875900000000

protective a preventative 0.04303732108135480000000

protective a special 0.00567821734453672000000

protective a careful 0.00005264531700381270000

protective a vigilant 0.00004340215732668570000

protective a conservative 0.01597629777677290000000

protective a preventive 0.46985305714326100000000

protective a maternal 0.03450385626831070000000

protective a paternal 0.01004858470267460000000

swell v increase 1.00000000000000000000000

immersion n absorption 0.01793248945147680000000

immersion n submersion 0.98206751054852300000000

independently r separately 0.91310748171595400000000

independently r individually 0.08689251828404550000000

beat v tap 0.57142857142857100000000

beat v stop 0.28571428571428600000000

beat v switch 0.14285714285714300000000

bacterium n microbe 0.00345676772713882000000

bacterium n pathogen 0.60750455544887700000000

bacterium n bacteria 0.22445613951908700000000

bacterium n microorganism 0.00035288795108003200000

bacterium n virus 0.16422964935381700000000

management n regulation 0.00182510861849605000000

management n administration 0.00303780244828727000000

management n skill 0.00100477924655292000000

management n executive 0.00007457097607194710000

management n oversight 0.00000453299597697604000

management n direction 0.00060839843147870700000

management n care 0.10269850044581200000000

management n guidance 0.00008564644829215350000

management n charge 0.00028072614672428200000

management n operation 0.01204906152783600000000

management n rule 0.00015840155282930800000

management n organization 0.00075376940201227900000

management n capability 0.00011072002355817900000

management n governance 0.00015017596371389800000

management n control 0.04518632306742770000000

management n board 0.00002005507311025760000

management n supervision 0.00047604141304744500000

management n government 0.00027244813022368000000

management n treatment 0.83076683037859200000000

management n running 0.00033645729665748900000

management n conduct 0.00009965041329924380000

assist v endorse 0.00008634849307964880000

assist v hasten 0.00000265850585419166000

assist v benefit 0.09237736501362030000000

assist v facilitate 0.17860983321499500000000

assist v promote 0.00000531701170838340000

assist v accelerate 0.00001063402341676690000

assist v sustain 0.00048272282131038400000

assist v advance 0.00000531701170838340000

assist v cultivate 0.00008103148137126540000

assist v encourage 0.00010229952820479900000

assist v attend 0.00065511892566260400000

assist v aid 0.20333303285474700000000

assist v relieve 0.00000265850585419166000

assist v visit 0.00594646409447594000000

assist v ameliorate 0.00000797551756257514000

assist v favor 0.00000531701170838340000

assist v do 0.00000531701170838340000

assist v uphold 0.00000265850585419166000

assist v advocate 0.00025372846753056300000

assist v support 0.00168277087175411000000

assist v alleviate 0.00000265850585419166000

assist v maintain 0.03993404151266220000000

assist v contribute 0.09944412123020160000000

assist v enhance 0.06443066171357280000000

assist v help 0.31252463115387400000000

assist v boost 0.00000531701170838340000

sacrifice n reduction 0.80756649423162900000000

sacrifice n cessation 0.09381133406208100000000

sacrifice n loss 0.09862217170629030000000

overgrowth n hypertrophy 0.97692307692307700000000

overgrowth n abundance 0.02307692307692310000000

etiology n aetiology 1.00000000000000000000000

ratify v fix 1.00000000000000000000000

complex n delusion 0.00018633405128912900000

complex n structure 0.02288507647959950000000

complex n fixation 0.00000952730055900580000

complex n system 0.39399654750525400000000

complex n association 0.05821849340387840000000

complex n network 0.00007725589698843630000

complex n combination 0.06149887960449970000000

complex n syndrome 0.14318829766133300000000

complex n conglomerate 0.02713613381718830000000

complex n aggregation 0.02931361712704010000000

complex n group 0.20903013440054800000000

complex n mania 0.00002592417847354970000

complex n web 0.01145166473307910000000

complex n notion 0.00075136668132627800000

complex n complication 0.04223074715894400000000

relief n remission 0.01066955780073550000000

relief n replacement 0.00335957357022852000000

relief n variety 0.00000109864825293636000

relief n aid 0.00212633268936712000000

relief n rest 0.04922333132486660000000

relief n maintenance 0.00003807686996291330000

relief n alleviation 0.03710715032223210000000

relief n cover 0.00015358891297540500000

relief n sedation 0.04739325984415000000000

relief n satisfaction 0.02476818930375600000000

relief n unemployment 0.00000397690699600203000

relief n medicine 0.00035768258614627200000

relief n cure 0.03946799181194270000000

relief n relaxation 0.01065788376187300000000

relief n therapy 0.07389986718699300000000

relief n analgesic 0.00718122950294631000000

relief n freedom 0.00216112959187114000000

relief n rescue 0.01638037946478970000000

relief n medication 0.15319407224678400000000

relief n configuration 0.00000454172503538302000

relief n loosening 0.00010077758327723800000

relief n assistance 0.00015358891297540500000

relief n palliation 0.05860152108889180000000

relief n substitute 0.00003605699085652860000

relief n ease 0.00000454172503538302000

relief n release 0.00487880033844276000000

relief n stimulation 0.01599926292295010000000

relief n stimulant 0.00000109864825293636000

relief n food 0.00017559504576073000000

relief n change 0.04952814494367140000000

relief n treatment 0.35331058714214300000000

relief n amelioration 0.00010187623153017400000

relief n narcotic 0.00135858187659384000000

relief n support 0.00044739740445247300000

relief n drug 0.03712337060749980000000

relief n opiate 0.00002988446576135110000

cautious a alert 0.57332297738956900000000

cautious a tentative 0.01968791173029550000000

cautious a careful 0.15537208059337900000000

cautious a vigilant 0.00025323453064910300000

cautious a conservative 0.18775435299282900000000

cautious a thoughtful 0.00235935381641798000000

cautious a safe 0.03332173800810860000000

cautious a judicious 0.02792835093875120000000

autonomous a independent 0.67291254165258200000000

autonomous a self-sufficient 0.32708745834741900000000

ultraviolet light n ultraviolet radiation 1.00000000000000000000000

ubiquitous a global 0.00275351437718425000000

ubiquitous a widespread 0.14530276568757500000000

ubiquitous a universal 0.85194371993524100000000

health care n healthcare 1.00000000000000000000000

trivial a minor 0.27458447127400200000000

trivial a little 0.01636403695738040000000

trivial a irrelevant 0.00010147490804812800000

trivial a common 0.07650069774129850000000

trivial a unimportant 0.00200796465065555000000

trivial a inferior 0.00020294981609625700000

trivial a small 0.59812241728307400000000

trivial a superficial 0.00010147490804812800000

trivial a useless 0.00020294981609625700000

trivial a slight 0.00425025766172423000000

trivial a inconsequential 0.00092415757469186300000

trivial a evanescent 0.00029056344294322600000

trivial a insignificant 0.01238667373420620000000

trivial a negligible 0.01356787188074420000000

trivial a worthless 0.00020294981609625700000

trivial a insubstantial 0.00018908853489509800000

permissive a favorable 0.43716783713380300000000

permissive a susceptible 0.02499268826385110000000

permissive a free 0.00228186894839277000000

permissive a tolerant 0.53555760565395300000000

serve v deliver 0.00002165467902545780000

serve v function 0.00617605345938062000000

serve v handle 0.00957177968223562000000

serve v observe 0.00685094614353531000000

serve v present 0.00243410563395129000000

serve v provide 0.12608020416144800000000

serve v act 0.41792376589979200000000

serve v suffice 0.00062435795587737400000

serve v respect 0.00002378477519262540000

serve v give 0.01268290459833800000000

serve v discharge 0.00003175888743947780000

serve v enable 0.00069989642299055100000

serve v advance 0.00050318263680995400000

serve v work 0.00000237179640263539000

serve v make 0.00205458232495031000000

serve v encourage 0.00009555569007516580000

serve v attend 0.00172249341944994000000

serve v agree 0.00000711538920790618000

serve v distribute 0.00000474359280527079000

serve v hold 0.00000742390060964541000

serve v fill 0.00000237179640263539000

serve v spend 0.00956940788583295000000

serve v do 0.00130208278618965000000

serve v fulfil 0.00002378477519262540000

serve v satisfy 0.00003146763901626080000

serve v advocate 0.00008338819666024950000

serve v pass 0.00000237179640263539000

serve v answer 0.00000237179640263539000

serve v support 0.02895359309046890000000

serve v complete 0.00116944851799483000000

serve v recommend 0.00007178817786106260000

serve v perform 0.00904048193303278000000

serve v follow 0.01474602370495440000000

serve v play 0.12531646000008300000000

serve v help 0.20755854276665400000000

serve v apply 0.00019893731899170800000

serve v offer 0.01440879676834260000000

entail v cause 0.50000000000000000000000

entail v involve 0.50000000000000000000000

antisocial a disruptive 1.00000000000000000000000

target v place 0.03011812811679930000000

target v direct 0.86539055861538600000000

target v focus 0.03320209115279660000000

target v aim 0.07116991307037910000000

target v point 0.00006720250034499180000

target v intend 0.00005210654429431680000

aetiological a etiological 0.13309318042245500000000

aetiological a etiologic 0.86690681957754500000000

white cell n white blood cell 0.48605947955390300000000

white cell n leukocyte 0.00278810408921933000000

white cell n leucocyte 0.00092936802973977700000

white cell n wbc 0.51022304832713800000000

violation n disruption 1.00000000000000000000000

tag n bromide 0.00088637115708860400000

tag n chip 0.55870928601818400000000

tag n conclusion 0.43951797166763800000000

tag n fragment 0.00088637115708860400000

stanford n stanford university 1.00000000000000000000000

palm n hand 0.99919975977550600000000

palm n foot 0.00016004804489881100000

palm n extremity 0.00016004804489881100000

palm n influence 0.00032009608979762200000

palm n success 0.00016004804489881100000

news n story 0.20605800341813700000000

news n paper 0.00077637102351691500000

news n communication 0.15454350256360300000000

news n description 0.00077637102351691500000

news n information 0.59372645052683500000000

news n publication 0.00077637102351691500000

news n data 0.03189474753172710000000

news n message 0.00989544084211392000000

news n knowledge 0.00155274204703383000000

multiply v increase 0.25000000000000000000000

multiply v calculate 0.75000000000000000000000

context n meaning 0.00004680622802570610000

context n theme 0.00000396388015086620000

context n situation 0.00571022431333270000000

context n environment 0.00093707992236284000000

context n setting 0.05807670188784230000000

context n substance 0.00151817835820548000000

context n relationship 0.01354806508161340000000

context n circumstance 0.00329421019730001000000

context n connection 0.00016317582516414000000

context n topic 0.00098328486399864300000

context n subject 0.02683601145401970000000

context n relation 0.00906393995445124000000

context n text 0.00009790961198010150000

context n background 0.87972044842155300000000

demonstrate v signify 0.00024225755632794600000

demonstrate v present 0.01311699882299800000000

demonstrate v settle 0.00007425698465385940000

demonstrate v indicate 0.11788650938294400000000

demonstrate v validate 0.00194663360642999000000

demonstrate v display 0.00360750345475474000000

demonstrate v exemplify 0.00038069044565820100000

demonstrate v sustain 0.00189050640463093000000

demonstrate v certify 0.00000180789221140258000

demonstrate v attest 0.00049068727969609400000

demonstrate v manifest 0.00290463275775488000000

demonstrate v illustrate 0.01172816757051770000000

demonstrate v describe 0.03042039790438590000000

demonstrate v test 0.01215546192228310000000

demonstrate v ascertain 0.00224915197146684000000

demonstrate v reveal 0.07392260597671250000000

demonstrate v confirm 0.04449004772915290000000

demonstrate v verify 0.00162444692242733000000

demonstrate v show 0.50602414372699100000000

demonstrate v establish 0.03882884702073580000000

demonstrate v fix 0.00009253764981256590000

demonstrate v evince 0.00000081158310327620000

demonstrate v determine 0.06495005004294610000000

demonstrate v try 0.00031539470206809900000

demonstrate v prove 0.02095981409856810000000

demonstrate v teach 0.00002115873192577400000

demonstrate v express 0.00206308718336279000000

demonstrate v explicate 0.00000653173960248661000

demonstrate v cite 0.00051097946133124600000

demonstrate v document 0.03080476527122210000000

demonstrate v evidence 0.00066973967811312300000

demonstrate v substantiate 0.00154096069528955000000

demonstrate v name 0.00001667551102770830000

demonstrate v expose 0.00124836712672082000000

demonstrate v exhibit 0.01002966665059610000000

demonstrate v strike 0.00005742725792134400000

demonstrate v explain 0.00272627728365583000000

closure n valve 0.00013100738732162500000

closure n resolution 0.07648997955662830000000

closure n blockage 0.00004262077878174950000

closure n block 0.00051694844265111900000

closure n cover 0.00454317480967303000000

closure n blockade 0.00039266006593819200000

closure n cessation 0.00090444678337672900000

closure n conclusion 0.47799308797968300000000

closure n joint 0.00016896776545271200000

closure n completion 0.00458028480799486000000

closure n barrier 0.01013327045523980000000

closure n termination 0.00007980037171901210000

closure n connection 0.00001552342537392760000

closure n union 0.00377142418279708000000

closure n obstruction 0.07865548035824790000000

closure n occlusion 0.24563500678615200000000

closure n plug 0.00000530374452841099000

closure n closing 0.09594101229844060000000

synchronize v synchronise 1.00000000000000000000000

spell n period 0.06568279437662040000000

spell n formula 0.00013870900675808000000

spell n attack 0.92881862741989900000000

spell n space 0.00013870900675808000000

spell n interval 0.00027741801351616100000

spell n time 0.00110967205406464000000

spell n stroke 0.00341794310210939000000

spell n influence 0.00041612702027424100000

retardation n block 0.00009039313481748950000

retardation n delay 0.62711331512319700000000

retardation n limitation 0.07766897178112380000000

retardation n difficulty 0.00086049963250620600000

retardation n restriction 0.00018078626963498200000

retardation n prolongation 0.00009039313481748950000

retardation n obstruction 0.00027117940445247400000

retardation n mental retardation 0.13842994967287200000000

retardation n handicap 0.15461479848370800000000

retardation n stop 0.00067971336287122400000

epinephrine n adrenalin 0.00006340505075661380000

epinephrine n adrenaline 0.99993659494924300000000

consequence n value 0.00830810092343680000000

consequence n issue 0.01561294344440700000000

consequence n effect 0.14962109916919300000000

consequence n gravity 0.00001259720049482910000

consequence n inference 0.00004323604543917250000

consequence n prominence 0.00000073929064532985200

consequence n magnitude 0.00182420734163162000000

consequence n concern 0.00860838397752267000000

consequence n regard 0.00011990245014896200000

consequence n conclusion 0.10583716687594900000000

consequence n interest 0.00706831637838321000000

consequence n event 0.15107227387059200000000

consequence n report 0.00790299450464113000000

consequence n ramification 0.00189179163218148000000

consequence n sequel 0.00046705490095502100000

consequence n substance 0.00033299506514896900000

consequence n force 0.00005803135849581220000

consequence n repercussion 0.00158895822458686000000

consequence n emphasis 0.00109757586479883000000

consequence n import 0.00002006493466291620000

consequence n account 0.00001539121604543360000

consequence n corollary 0.00000078862553477703800

consequence n distinction 0.00029165989103855300000

consequence n outcome 0.21281000506766500000000

consequence n matter 0.00000025697963547115900

consequence n note 0.00002283581686515040000

consequence n weight 0.00069928402921080300000

consequence n stress 0.00698702684902283000000

consequence n urgency 0.00003182892658360130000

consequence n reaction 0.00784233939512350000000

consequence n name 0.00000025697963547115900

consequence n end 0.00302953877466637000000

consequence n standing 0.00002440120560937580000

consequence n implication 0.09130204946231050000000

consequence n significance 0.03851519221288960000000

consequence n importance 0.01990546843648870000000

consequence n elevation 0.00015006647097007400000

consequence n influence 0.00832729747018201000000

consequence n moment 0.00000157428584951221000

consequence n result 0.14829610795732200000000

consequence n product 0.00025819649403666800000

binding n dressing 1.00000000000000000000000

obstetrical a obstetric 1.00000000000000000000000

incompatible a discrepant 0.30985608466967700000000

incompatible a antagonistic 0.01214787593794010000000

incompatible a inappropriate 0.62584249790066100000000

incompatible a discordant 0.05215354149172170000000

fear v start 0.05961577556413890000000

fear v freeze 0.01987192518804630000000

fear v expect 0.45705427932506600000000

fear v anticipate 0.07948770075218520000000

fear v suspect 0.38397031917056300000000

disappointing a unsatisfying 0.03801886989086970000000

disappointing a limited 0.01190385267182770000000

disappointing a inadequate 0.20502107827658600000000

disappointing a inferior 0.00591946239920145000000

disappointing a insufficient 0.06169294470379120000000

disappointing a ineffective 0.23434731649675700000000

disappointing a unexpected 0.05010510759897970000000

disappointing a unsatisfactory 0.38929626465349500000000

disappointing a mediocre 0.00369510330849196000000

vapor n smoke 1.00000000000000000000000

utah n ut 1.00000000000000000000000

tocopherol n vitamin e 1.00000000000000000000000

short-lived a transitory 0.00391341781177547000000

short-lived a brief 0.01485450674615860000000

short-lived a temporary 0.59488072456375300000000

short-lived a sudden 0.00272553705622303000000

short-lived a evanescent 0.00008490238528156020000

short-lived a fleeting 0.00061502016632300400000

short-lived a transient 0.38292589127048500000000

refractory a recalcitrant 0.03455747849751640000000

refractory a uncontrollable 0.55291965596026300000000

refractory a rigid 0.00022618926404592400000

refractory a contrary 0.00069028671156087700000

refractory a difficult 0.40423412748714400000000

refractory a untoward 0.00737226207947017000000

chimera n delusion 1.00000000000000000000000

cad n dog 1.00000000000000000000000

petitioner n candidate 1.00000000000000000000000

parturition n labor 0.60355916660454600000000

parturition n delivery 0.21568029151235100000000

parturition n birth 0.18076054188310400000000

horizon n view 0.05950616450282090000000

horizon n range 0.69132602206271000000000

horizon n scope 0.24916781343447000000000

collision n accident 0.57755672281197800000000

collision n impact 0.01110276022727070000000

collision n crash 0.41134051696075200000000

weapon n arm 1.00000000000000000000000

waste n consumption 0.00570464883056201000000

waste n decline 0.00285232441528101000000

waste n residue 0.98833040989486900000000

waste n wear 0.00285232441528101000000

waste n feces 0.00026029244400707500000

tx n texas 1.00000000000000000000000

superoxide anion n superoxide 1.00000000000000000000000

smell n odor 0.93168880455408000000000

smell n nose 0.06831119544592030000000

incidence n number 0.04536031326729420000000

incidence n phenomenon 0.00201052467103633000000

incidence n range 0.00732604466364540000000

incidence n event 0.08088914667856160000000

incidence n measure 0.00573903877604677000000

incidence n bond 0.00000007571296928361770

incidence n area 0.00437928710325907000000

incidence n fact 0.00087679186194811600000

incidence n association 0.02823929820397600000000

incidence n alliance 0.00000015218005180711200

incidence n realization 0.00002308319577195180000

incidence n degree 0.00751297052518308000000

incidence n frequency 0.14882199869625400000000

incidence n direction 0.00077243140175436200000

incidence n occasion 0.00096973955255092100000

incidence n set 0.00005978283253994760000

incidence n prevalence 0.16114932866418300000000

incidence n relationship 0.01857798255758100000000

incidence n tendency 0.00046616124067326700000

incidence n circumstance 0.00042663707796304300000

incidence n link 0.00024100182779218100000

incidence n connection 0.00004732215651670060000

incidence n experience 0.00607561548148132000000

incidence n linkage 0.00005494016286540090000

incidence n course 0.00595020332918867000000

incidence n rate 0.37597073994580600000000

incidence n happening 0.00000029605977066078400

incidence n union 0.00017764909096709600000

incidence n percentage 0.00839109732543937000000

incidence n aim 0.00167290621234932000000

incidence n extent 0.00589713668684427000000

incidence n proportion 0.01650722186174130000000

incidence n scope 0.00085508595964030900000

incidence n trend 0.00776135766625054000000

incidence n occurrence 0.05040563935107170000000

incidence n relation 0.00628524628564582000000

incidence n line 0.00010575173338705300000

prostate n prostate gland 1.00000000000000000000000

paediatrician n pediatrician 1.00000000000000000000000

instantaneous a instant 0.83178923541378100000000

instantaneous a expeditious 0.00098247658102912000000

instantaneous a abrupt 0.09053488276612580000000

instantaneous a spontaneous 0.07669340523906400000000

initiate v start 0.36462485314966900000000

initiate v begin 0.22626855624932700000000

initiate v enter 0.00395578148659384000000

initiate v infuse 0.01447774765958300000000

initiate v create 0.00446263996189035000000

initiate v implant 0.00121414743843546000000

initiate v install 0.00006741116679349670000

initiate v drill 0.00002683971373192500000

initiate v prepare 0.01585811028474970000000

initiate v launch 0.00409599158258261000000

initiate v train 0.00079493094475260400000

initiate v trigger 0.00078006131547998400000

initiate v introduce 0.01954779980679670000000

initiate v prime 0.00004839764870156810000

initiate v exercise 0.00211022599961782000000

initiate v practice 0.00424885157429625000000

initiate v open 0.00118882975957998000000

initiate v instigate 0.00077081813039880800000

initiate v originate 0.01692020286516770000000

initiate v establish 0.07281532871106470000000

initiate v institute 0.19093383282860800000000

initiate v found 0.00001498910633264520000

initiate v teach 0.00275573129413712000000

initiate v organize 0.00109430764875111000000

initiate v enroll 0.02166994524094840000000

initiate v commence 0.00270362316545654000000

initiate v conceive 0.00035128281985656900000

initiate v ready 0.00008837617942241340000

initiate v instruct 0.00247857243281435000000

initiate v admit 0.02363181383445980000000

immobility n stability 1.00000000000000000000000

damaging a bad 0.00393092525456741000000

damaging a deleterious 0.85748724387740400000000

damaging a detrimental 0.13858183086802900000000

confront v resist 0.00086792875436764100000

confront v meet 0.00278747068111385000000

confront v address 0.25834681657624600000000

confront v present 0.06490923737992520000000

confront v encounter 0.14130098387212100000000

confront v challenge 0.00182769971774074000000

confront v tackle 0.00250946032213308000000

confront v thwart 0.00086792875436764100000

confront v face 0.52475477422424400000000

confront v pursue 0.00182769971774074000000

cheek n face 0.76131905088123600000000

cheek n lip 0.23868094911876400000000

apoptosis n programmed cell death 1.00000000000000000000000

socket n device 1.00000000000000000000000

unilateral a single 1.00000000000000000000000

membership n adherence 0.12414060150645500000000

membership n association 0.01149216148228630000000

membership n society 0.10364031868887500000000

membership n group 0.76072691832238400000000

correlative a similar 0.29258597782363100000000

correlative a complementary 0.70416135122938700000000

correlative a analogous 0.00325267094698185000000

ga n georgia 0.69372871263166200000000

ga n gallium 0.30627128736833800000000

vice versa r conversely 1.00000000000000000000000

opposite n contrary 0.49210060348868700000000

opposite n opposition 0.00112970128860629000000

opposite n converse 0.50676969522270700000000

work n capacity 0.00000023234201674839800

work n position 0.00038537913940389800000

work n act 0.00000029590107498146300

work n function 0.00375207750486026000000

work n profession 0.00000323099167801616000

work n art 0.00000586495228509454000

work n issue 0.00126467462589806000000

work n effect 0.00600056009731215000000

work n labor 0.00162599231871875000000

work n feat 0.00000148099435561399000

work n effort 0.00627561531069346000000

work n attempt 0.00014046493624838500000

work n movement 0.00001852259662831860000

work n field 0.00005945718615100330000

work n drill 0.00000147694053894716000

work n role 0.00086946576546543100000

work n task 0.00006970587125320480000

work n situation 0.00032475079547450100000

work n facility 0.00004165440947429430000

work n machinery 0.00000323099167801616000

work n office 0.00026758985297565300000

work n output 0.00000023012892105289800

work n place 0.00001196774669350140000

work n accomplishment 0.00000046475074093772400

work n mechanism 0.00101579713359994000000

work n child 0.00211313983759539000000

work n labour 0.00000609296713976509000

work n exercise 0.00811051004322676000000

work n charge 0.00006999529432581530000

work n operation 0.00244568800022177000000

work n offspring 0.00000785116908898124000

work n working 0.00006337550399976230000

work n enterprise 0.00032374536613721900000

work n arsenal 0.00000478913263516596000

work n artifact 0.00000354177322718727000

work n maneuver 0.00001924539586825480000

work n practice 0.00426375959100982000000

work n production 0.00004854907513031230000

work n performance 0.00076675275320002400000

work n industry 0.00000419959546340103000

work n outcome 0.00117655857400244000000

work n muscle 0.00004691762717564500000

work n stroke 0.00130703819060245000000

work n employment 0.00358462582677888000000

work n graft 0.00025271794203852400000

work n job 0.00033429695989219700000

work n achievement 0.00000323099167801616000

work n heart 0.00042853059650557400000

work n stress 0.00023867769022140900000

work n study 0.87328027205189000000000

work n action 0.00033102022920960800000

work n workshop 0.00018907318334274900000

work n doing 0.00006408353917223670000

work n occupation 0.00093311619776708400000

work n service 0.00013869121271158600000

work n station 0.00000049912487847189700

work n harvest 0.00000217366832198538000

work n trial 0.01445013472494370000000

work n exertion 0.00060702256150728500000

work n workplace 0.00096697669386347000000

work n result 0.05092524484979600000000

work n product 0.00002006284065137030000

work n call 0.00000940558773547473000

work n line 0.00000449973058721744000

work n training 0.00207104614837301000000

work n activity 0.00732365032637694000000

work n pursuit 0.00080936341534304700000

work n duty 0.00002997642279048320000

work n strain 0.00007967430943318980000

justification n case 0.36625814092462600000000

justification n reason 0.16205458008945900000000

justification n premise 0.00004957717320111860000

justification n validation 0.05649751316156590000000

justification n confirmation 0.25343842222537900000000

justification n rationale 0.14259990888726300000000

justification n response 0.00879284299708289000000

justification n delivery 0.00175477039509818000000

justification n approval 0.00310569928239736000000

justification n basis 0.00059492607841342600000

justification n answer 0.00480404161231274000000

justification n clearance 0.00004957717320111860000

inference n determination 0.06117914558241680000000

inference n sequence 0.00020423875041915300000

inference n reference 0.00209079203087917000000

inference n consequence 0.00184339981374912000000

inference n understanding 0.00004340859419767820000

inference n estimate 0.00027916753162895800000

inference n reason 0.00031520187012126500000

inference n conclusion 0.21426632064683200000000

inference n summation 0.00003152018701212650000

inference n suspicion 0.00003152018701212650000

inference n calculation 0.05788834400044810000000

inference n theory 0.00322616402186853000000

inference n opinion 0.00008041507811073690000

inference n belief 0.00004340859419767820000

inference n supposition 0.00004340859419767820000

inference n impression 0.00076604286712126000000

inference n suggestion 0.00340121308697787000000

inference n assumption 0.00004340859419767820000

inference n judgment 0.04834487483452440000000

inference n induction 0.00059648009891285800000

inference n answer 0.00003152018701212650000

inference n hypothesis 0.02246609695114870000000

inference n implication 0.15403289118368100000000

inference n finding 0.15527170472073800000000

inference n solution 0.00048017706088726800000

inference n result 0.27163426954126000000000

inference n derivation 0.00003152018701212650000

inference n argument 0.00133334520343437000000

respect n reference 0.00158930791853946000000

respect n aspect 0.01967950823588190000000

respect n compliance 0.00086690616184653800000

respect n consideration 0.00179512848684699000000

respect n way 0.00214681497222852000000

respect n regard 0.93387782592975000000000

respect n detail 0.01036057524001850000000

respect n estimation 0.00013582299346429500000

respect n feature 0.01878504799365590000000

respect n connection 0.00058341498534923100000

respect n point 0.00241701034904668000000

respect n relation 0.00776263673337142000000

american indian n indian 1.00000000000000000000000

spinal fusion n fusion 1.00000000000000000000000

possibility n probability 0.07016848986663820000000

possibility n option 0.10930956783628000000000

possibility n alternative 0.03038447345814860000000

possibility n likelihood 0.02389038848860190000000

possibility n instance 0.00886527486982889000000

possibility n feasibility 0.00124810144628144000000

possibility n occasion 0.00593552690033022000000

possibility n chance 0.02574974521003330000000

possibility n theory 0.02060213099266430000000

possibility n circumstance 0.00153561835537740000000

possibility n promise 0.00362921092184536000000

possibility n happening 0.00000156274332790814000

possibility n opening 0.00003855735375134500000

possibility n fear 0.00011101625983690700000

possibility n incident 0.00004327531176087810000

possibility n danger 0.04586975934353700000000

possibility n risk 0.18982974684917400000000

possibility n hypothesis 0.32684553412244400000000

possibility n hope 0.00610711780433287000000

possibility n hazard 0.00890970702146271000000

possibility n occurrence 0.02416061948857910000000

possibility n prospect 0.00212138423677005000000

possibility n solution 0.00420147057179750000000

possibility n potential 0.08355673652978990000000

possibility n choice 0.00688498401740625000000

equal n counterpart 1.00000000000000000000000

agreement n proposal 0.00040552987750190900000

agreement n dealing 0.00004930115999719150000

agreement n adjustment 0.00077331721582679300000

agreement n symmetry 0.00166368052633520000000

agreement n recognition 0.00512443704184707000000

agreement n understanding 0.00506833384911795000000

agreement n compliance 0.05580870275931290000000

agreement n uniformity 0.00001881992193970560000

agreement n correspondence 0.01518716357711060000000

agreement n concert 0.00003068774938743150000

agreement n coincidence 0.00008715040355741440000

agreement n satisfaction 0.01870181305302390000000

agreement n balance 0.00161678985910504000000

agreement n accord 0.01693332787081530000000

agreement n consensus 0.29924427462174900000000

agreement n unity 0.02010266771310230000000

agreement n analogy 0.00218710567684210000000

agreement n concurrence 0.00008035375658338930000

agreement n settlement 0.00010921789067700800000

agreement n alliance 0.00000583800810059702000

agreement n confirmation 0.00455717608420294000000

agreement n convention 0.00012835095181750600000

agreement n conformity 0.00430630540383635000000

agreement n peace 0.00005156621817888060000

agreement n kinship 0.00000463023270402834000

agreement n affinity 0.00204985688629207000000

agreement n concordance 0.37055575550263000000000

agreement n coherence 0.00004520329151413440000

agreement n acceptance 0.01362318242894480000000

agreement n similarity 0.00348676398157693000000

agreement n consistency 0.09308993538021590000000

agreement n congruity 0.00192410132435496000000

agreement n compromise 0.00005920565790908520000

agreement n promise 0.00023676987934239200000

agreement n word 0.00039510826775189300000

agreement n union 0.00075118711932377900000

agreement n approval 0.00002465057999859580000

agreement n consent 0.00057025291145790200000

agreement n note 0.00171122237351999000000

agreement n protocol 0.01385474230401340000000

agreement n accordance 0.04500016982065510000000

agreement n cooperation 0.00037172254117460400000

agreement n arrangement 0.00000362832665224525000

hospital n hospice 0.00312555391527989000000

hospital n public hospital 0.04775616092386040000000

hospital n infirmary 0.00809574867195289000000

hospital n mental hospital 0.00809574867195289000000

hospital n asylum 0.00000417737289574452000

hospital n nursing home 0.04026911724090340000000

hospital n dispensary 0.00404787433597644000000

hospital n sanatorium 0.00009561907880259320000

hospital n clinic 0.30767145248526400000000

hospital n home 0.58083854730311200000000

distinct a categorical 0.00011616198055621900000

distinct a apparent 0.00724896849702725000000

distinct a recognizable 0.00222925336702394000000

distinct a unusual 0.04042040173900360000000

distinct a peculiar 0.00371741962138845000000

distinct a certain 0.01936073759395210000000

distinct a particular 0.01128562104117950000000

distinct a noticeable 0.00011228314868500400000

distinct a specific 0.07735267679433720000000

distinct a distinguishable 0.00000682843024099621000

distinct a graphic 0.00001459978636380060000

distinct a exceptional 0.00150055817422514000000

distinct a unique 0.08015482247282550000000

distinct a uncommon 0.02180970450382900000000

distinct a different 0.26021505321254300000000

distinct a conspicuous 0.00005292033436772090000

distinct a separate 0.09883175363003030000000

distinct a dissimilar 0.00077561365057690700000

distinct a clear-cut 0.00003305526442775920000

distinct a special 0.00162270886183190000000

distinct a evident 0.00631310312861250000000

distinct a clear 0.02257259874291790000000

distinct a definite 0.03369219171120380000000

distinct a prominent 0.00365583784385286000000

distinct a singular 0.00000507662851676437000

distinct a visible 0.00062147417814041900000

distinct a diverse 0.00213952272211270000000

distinct a obvious 0.00071481313454653000000

distinct a palpable 0.00047683057211183300000

distinct a patent 0.00026534574281060800000

distinct a discrete 0.02700005969566570000000

distinct a distinctive 0.04638886225431800000000

distinct a striking 0.00336919914395308000000

distinct a various 0.01693391804893750000000

distinct a independent 0.03608723914007320000000

distinct a rare 0.08711343956035410000000

distinct a notable 0.00046081692092000400000

distinct a well-defined 0.01402892527225030000000

distinct a well defined 0.01325047755147090000000

distinct a individual 0.02085561416229740000000

distinct a explicit 0.00000366449679882785000

distinct a manifest 0.00026920725096422200000

distinct a marked 0.03686274830429500000000

distinct a unequivocal 0.00005789168846027200000

omission n error 0.10988215892663500000000

omission n flaw 0.01540228552475910000000

omission n elimination 0.00488895118393060000000

omission n fault 0.00488895118393060000000

omission n need 0.58336462093124100000000

omission n neglect 0.28157303224950300000000

money n percentage 0.02307317864676290000000

money n change 0.97692682135323700000000

traumatic a distressing 0.00038762817167181300000

traumatic a disturbing 0.02798998422946890000000

traumatic a painful 0.03077128931703100000000

traumatic a stressful 0.94085109828182800000000

exceedingly r excessively 0.00584389619113784000000

exceedingly r extremely 0.89932026608108100000000

exceedingly r highly 0.08405922203411510000000

exceedingly r remarkably 0.00628985329316425000000

exceedingly r especially 0.00448676240050160000000

elucidation n interpretation 0.00786362028689541000000

elucidation n clarification 0.67001738739422800000000

elucidation n definition 0.14713623637658400000000

elucidation n demonstration 0.01252756059498510000000

elucidation n description 0.01616459431610980000000

elucidation n diagnosis 0.00282064640725596000000

elucidation n explanation 0.03710740437518890000000

elucidation n commentary 0.05676550894602620000000

elucidation n delineation 0.04959704130272650000000

book n record 0.00192148594913400000000

book n publication 0.06879987190093670000000

book n diary 0.00048037148728349800000

book n text 0.92879827066264600000000

assistant n participant 1.00000000000000000000000

antimony n sb 1.00000000000000000000000

wakefulness n surveillance 0.03190994027113170000000

wakefulness n caution 0.78197223230824400000000

wakefulness n prudence 0.03190994027113170000000

wakefulness n insomnia 0.15420788714949200000000

sway n direction 0.83333333333333300000000

sway n influence 0.16666666666666700000000

unknown n question 0.80661243901838800000000

unknown n enigma 0.13606926783234400000000

unknown n mystery 0.03339495907188280000000

unknown n secret 0.00112671044929969000000

unknown n x 0.02279662362808530000000

non-living a nonliving 1.00000000000000000000000

understanding n appreciation 0.01789609583047800000000

understanding n capacity 0.00040742932396635600000

understanding n interpretation 0.00539943343648709000000

understanding n recognition 0.05654888049479500000000

understanding n agreement 0.00533472441697114000000

understanding n reason 0.06752852976115930000000

understanding n view 0.01695863939830670000000

understanding n consideration 0.05554640316109260000000

understanding n judgement 0.00000398484914377452000

understanding n concern 0.01952578929325730000000

understanding n sensitivity 0.01489727630418010000000

understanding n handle 0.00000789378560838129000

understanding n conclusion 0.30449191376973000000000

understanding n skill 0.00321679651167198000000

understanding n perception 0.00366894549226525000000

understanding n awareness 0.04969101379842640000000

understanding n realization 0.00002980401930722180000

understanding n efficiency 0.00016486869628038800000

understanding n insight 0.13472766624385500000000

understanding n approach 0.06077054873419090000000

understanding n grasp 0.00740331839591392000000

understanding n acumen 0.00067713278011407600000

understanding n brain 0.00537119810228212000000

understanding n opinion 0.00088417192786198700000

understanding n belief 0.00001248110285634510000

understanding n tolerability 0.00167414361479595000000

understanding n penetration 0.00000789378560838129000

understanding n impression 0.00000857216639173834000

understanding n consciousness 0.00002871729401052720000

understanding n compromise 0.00061016360405883700000

understanding n cognition 0.00068777109926531500000

understanding n faculty 0.00000190951537139255000

understanding n conception 0.00043318243184004500000

understanding n cognizance 0.00694061099616928000000

understanding n feeling 0.00002871729401052720000

understanding n head 0.00000796969828754904000

understanding n idea 0.00001439061822773770000

understanding n knowledge 0.14556686996674900000000

understanding n mind 0.00009971741460817830000

understanding n intelligence 0.00017819812857833000000

understanding n cooperation 0.00091821376335892900000

understanding n arrangement 0.00000190951537139255000

understanding n familiarity 0.01161284820492340000000
[truncated: 3,234,725 more chars]
