# Supplementary material for: Benchmarking Ontologies: Bigger or Better?
Source: PLoS Comput Biol. 2011 Jan 13;7(1):e1001055. doi: 10.1371/journal.pcbi.1001055 (PMC3020923; doi:10.1371/journal.pcbi.1001055)
Supplement: Dataset S3 — Probabilities – news. (25.12 MB DOC) [file pcbi.1001055.s003.doc]

word partofspeech synonym Prob

imminent a threatening 0.00027076205085245700000

imminent a nigh 0.00455075019139093000000

imminent a close 0.03253804380227420000000

imminent a immediate 0.38649901472027300000000

imminent a near 0.08468495815975710000000

imminent a nearby 0.00807596214991358000000

imminent a expected 0.00892419632924222000000

imminent a forthcoming 0.06094524483812910000000

imminent a inevitable 0.06224952187276920000000

imminent a coming 0.00004144311476534000000

imminent a impending 0.05117801538063760000000

imminent a next 0.21754740914528800000000

imminent a following 0.08249467824470820000000

collectible n collectable 1.00000000000000000000000

chopper n helicopter 1.00000000000000000000000

snow n opium 0.00023541400848593400000

snow n c 0.00002249227051276690000

snow n snowfall 0.40062909641638000000000

snow n blizzard 0.03518041022313550000000

snow n snowstorm 0.56301422357670800000000

snow n blow 0.00001095337353218880000

snow n horse 0.00022969083958449900000

snow n cocaine 0.00003843453411121860000

snow n heroin 0.00023637453677358700000

snow n coke 0.00040291022077606500000

reality n being 0.00001386103645443920000

reality n fact 0.30530749071401300000000

reality n presence 0.07893963182157750000000

reality n realism 0.00070371230735619800000

reality n realization 0.00268637742306206000000

reality n entity 0.00037742184138031200000

reality n verity 0.00041960574540504200000

reality n truth 0.20591049548022300000000

reality n fidelity 0.00148023068632204000000

reality n substance 0.00299282947626249000000

reality n existence 0.01114844940091280000000

reality n certainty 0.00451345275824879000000

reality n world 0.38550644130878200000000

guideline n regulation 0.12862811338570900000000

guideline n gauge 0.00000251004805998641000

guideline n limit 0.06358445753444470000000

guideline n direction 0.00598634136698828000000

guideline n bound 0.00000297004377918335000

guideline n pointer 0.00000530411117227371000

guideline n indication 0.00156282992005338000000

guideline n key 0.03840779246410650000000

guideline n rule 0.29853852800631800000000

guideline n measure 0.21953227574922500000000

guideline n stricture 0.00140940179055763000000

guideline n instruction 0.00226596282095230000000

guideline n restraint 0.00001060822234454760000

guideline n guide 0.00078174693398768300000

guideline n standard 0.19306934532002700000000

guideline n index 0.04585756820868750000000

guideline n margin 0.00031991467073171400000

guideline n marker 0.00001706234083214860000

guideline n prescription 0.00001726706202404930000

alarmed a nervous 0.02680885724573650000000

alarmed a concerned 0.97319114275426300000000

defendant n suspect 0.83478287491528600000000

defendant n appellant 0.02768866878432190000000

defendant n respondent 0.00208094105879007000000

defendant n defense 0.03079277379815600000000

defendant n offender 0.00143499531115084000000

defendant n party 0.10321974613229500000000

council n company 0.05385470673452000000000

council n senate 0.00786146374545254000000

council n caucus 0.00045659346743736600000

council n assembly 0.02717872116447830000000

council n government 0.09628746803502840000000

council n meeting 0.03814512541201520000000

council n panel 0.00920007456760765000000

council n commission 0.50100650840316500000000

council n chamber 0.00392105329068974000000

council n board 0.07812318643662350000000

council n ministry 0.01929006202563700000000

council n convention 0.00133444372397358000000

council n conference 0.00729830106113701000000

council n synod 0.00001876330256174370000

council n parliament 0.02234839184066350000000

council n authority 0.01204024948072130000000

council n executive 0.00175939297141744000000

council n consultation 0.00094985776146714500000

council n committee 0.06500676315737130000000

council n conclave 0.00000184641510125438000

council n gathering 0.00054057449562042000000

council n congress 0.01189187648296440000000

council n bureau 0.00172528883474372000000

council n directorate 0.01120195184065200000000

council n cabinet 0.02343919582031500000000

council n administration 0.00449037659866362000000

council n corporation 0.00062776292997211400000

advice n letter 0.00172639494704285000000

advice n news 0.00228500643233154000000

advice n tenet 0.00000447055406023156000

advice n encouragement 0.00021659342590551800000

advice n opinion 0.03792539883009130000000

advice n scheme 0.00082145728575150100000

advice n wisdom 0.00003138288082874490000

advice n plan 0.02127327842199740000000

advice n direction 0.00132393957572757000000

advice n idea 0.00190280890682224000000

advice n communication 0.00008292463786088290000

advice n appeal 0.02131945690464190000000

advice n doctrine 0.00000328636855384797000

advice n recommendation 0.17896809567328600000000

advice n data 0.00006994754923208500000

advice n warning 0.00878398726710612000000

advice n message 0.00601127454555464000000

advice n word 0.00578581340998827000000

advice n help 0.02613291905658690000000

advice n principle 0.00048432316968781700000

advice n pointer 0.00000166824204805274000

advice n counselling 0.00001874264981448050000

advice n notice 0.05667538628636050000000

advice n intelligence 0.00001733585351004300000

advice n law 0.01388831715471900000000

advice n motto 0.00000909201365589448000

advice n order 0.09712498582287950000000

advice n input 0.00728135052998645000000

advice n bidding 0.00006000019299693960000

advice n understanding 0.00034459989860788200000

advice n charge 0.00296567258501952000000

advice n suggestion 0.00158572802659036000000

advice n command 0.00004583126890823610000

advice n consultation 0.01882147873159850000000

advice n caution 0.00007999378858065030000

advice n judgment 0.00016313382033500400000

advice n counsel 0.00872926900590321000000

advice n knowledge 0.01263182848483310000000

advice n information 0.19668465357121000000000

advice n proposal 0.00852668491903791000000

advice n rule 0.00986737506312362000000

advice n memorandum 0.00002443379715709870000

advice n account 0.01264049766468400000000

advice n advisement 0.00003748529962896110000

advice n injunction 0.00327709879811380000000

advice n instruction 0.00070199082192616100000

advice n proposition 0.00000790782814951089000

advice n hint 0.00040249309648102500000

advice n lesson 0.00004230030763563780000

advice n view 0.01354675945822180000000

advice n guidance 0.00004319405236491560000

advice n tip 0.00009440973134504300000

advice n aid 0.01956548040267410000000

advice n announcement 0.17379879132606900000000

advice n prescription 0.00005471576522504420000

advice n notification 0.00515987265462722000000

advice n memo 0.00000118418550638359000

advice n report 0.01992549705741400000000

unsound a insolvent 0.00120315954687650000000

unsound a incorrect 0.03019381302700860000000

unsound a invalid 0.00120315954687650000000

unsound a flawed 0.18191772348772700000000

unsound a faulty 0.60639241162575600000000

unsound a untrue 0.00601579773438250000000

unsound a groundless 0.00601579773438250000000

unsound a unsafe 0.00240631909375300000000

unsound a erroneous 0.00120315954687650000000

unsound a defective 0.00120315954687650000000

unsound a incompetent 0.00360947864062950000000

unsound a untenable 0.03976343682791840000000

unsound a dangerous 0.06383078017113220000000

unsound a false 0.00240631909375300000000

unsound a weak 0.02021308038752520000000

unsound a unstable 0.01732549747502160000000

unsound a unfounded 0.01509690651350430000000

transpire v arrive 0.00331885552879649000000

transpire v prove 0.00199131331727789000000

transpire v occur 0.11902399290865300000000

transpire v intervene 0.00132754221151859000000

transpire v issue 0.00132754221151859000000

transpire v fall 0.13206458883418700000000

transpire v come 0.35264348564157600000000

transpire v happen 0.38830267934647200000000

researcher n investigator 1.00000000000000000000000

thirty n 30 1.00000000000000000000000

audience n access 0.00649840440715288000000

audience n discussion 0.00188571758840370000000

audience n assembly 0.00545920644594692000000

audience n review 0.00032048050223212100000

audience n meeting 0.36887472887141800000000

audience n hearing 0.00654206014972445000000

audience n crowd 0.30432585993158100000000

audience n house 0.01387920605821010000000

audience n conference 0.20041367056206600000000

audience n following 0.00550770358103911000000

audience n consultation 0.00007275558454045870000

audience n admission 0.00003568281584427090000

audience n turnout 0.00123603705997255000000

audience n reception 0.00052464232237080200000

audience n interview 0.07170157575111870000000

audience n trial 0.00493066040675634000000

audience n public 0.00779160796162243000000

sully v mar 1.00000000000000000000000

visitor n company 0.25456747908069500000000

visitor n caller 0.00003016390434018420000

visitor n patron 0.00000371330326684187000

visitor n foreigner 0.01344102474435460000000

visitor n tourist 0.67739498363573700000000

visitor n holidaymaker 0.00138956255836092000000

visitor n guest 0.03388654458988980000000

visitor n pilgrim 0.00064674396124227200000

visitor n traveller 0.01863978422211300000000

doubt v hesitate 0.00032266127497728500000

doubt v fear 0.41723167616081200000000

doubt v question 0.17631054537048800000000

doubt v query 0.00001698319517400190000

doubt v suspect 0.11656956186787700000000

doubt v challenge 0.00725529164874036000000

doubt v imagine 0.06618999437822550000000

doubt v object 0.00016737009722689900000

doubt v wonder 0.09409468299173720000000

doubt v fluctuate 0.00003396639034800380000

doubt v dispute 0.00008009170545804920000

doubt v deny 0.12172239807407800000000

doubt v contradict 0.00000477684485776937000

shilling n bob 1.00000000000000000000000

refill v replenish 1.00000000000000000000000

pudding n dessert 1.00000000000000000000000

poverty n deficiency 0.00021320655158004400000

poverty n starvation 0.14180631451110000000000

poverty n lack 0.00324342056147200000000

poverty n shortage 0.01399619085521940000000

poverty n failing 0.00039247451554212200000

poverty n debt 0.40404105555105900000000

poverty n absence 0.02330356848974200000000

poverty n deficit 0.26389566926171500000000

poverty n default 0.00111971148792372000000

poverty n hunger 0.03602367040720850000000

poverty n need 0.01421438345473350000000

poverty n famine 0.08369646470726520000000

poverty n crunch 0.01122757453390140000000

poverty n defect 0.00282629511153928000000

pillage v demolish 0.00027834477639636300000

pillage v plunder 0.99109296715531700000000

pillage v destroy 0.00167006865837818000000

pillage v damage 0.00111337910558545000000

pillage v violate 0.00250510298756727000000

pillage v sack 0.00111337910558545000000

pillage v burn 0.00027834477639636300000

pillage v ruin 0.00083503432918908800000

pillage v loot 0.00055668955279272500000

pillage v rob 0.00027834477639636300000

pillage v rape 0.00027834477639636300000

peel n shell 1.00000000000000000000000

key a pivotal 0.00458019843991826000000

key a primary 0.01145665856307240000000

key a material 0.00000475204601586866000

key a central 0.02993656322463560000000

key a indispensable 0.00000073897301692147800

key a major 0.19992880994549800000000

key a basic 0.00878120849433518000000

key a critical 0.03346931558523770000000

key a leading 0.01713424630163900000000

key a crucial 0.05178993714254480000000

key a fundamental 0.00559106879599238000000

key a vital 0.01749914113369850000000

key a principal 0.00557647092706671000000

key a essential 0.00977712439456876000000

key a main 0.33955532184773900000000

key a dominant 0.00590156768494956000000

key a chief 0.02308714160662950000000

key a decisive 0.01724256833082510000000

key a important 0.15508292669453200000000

key a prime 0.06360423986808540000000

nick n chip 0.00024336369563123700000

nick n mark 0.78570632089744100000000

nick n score 0.00092376449484553800000

nick n hole 0.19856542612485600000000

nick n spot 0.01456112478722670000000

battle v charge 0.01863909657403790000000

battle v assault 0.00000146276863152671000

battle v massacre 0.00000438830589458013000

battle v argue 0.00003325804906163820000

battle v attack 0.02115638840992890000000

battle v meet 0.00731748858803712000000

battle v struggle 0.03330445210458340000000

battle v spar 0.00000146276863152671000

battle v insult 0.00000438830589458013000

battle v engage 0.00000146276863152671000

battle v work 0.02602056008423910000000

battle v resist 0.00001615724931113000000

battle v rush 0.00000292553726305342000

battle v skirmish 0.00064065941586708100000

battle v clash 0.04820962104399780000000

battle v combat 0.01057088036180680000000

battle v confront 0.01409743268633880000000

battle v batter 0.00037788046901990500000

battle v encounter 0.00000292553726305342000

battle v dispute 0.00000146276863152671000

battle v raid 0.00000438830589458013000

battle v beat 0.00028240576892412800000

battle v strive 0.02538514372386250000000

battle v fight 0.79390908071793200000000

battle v slaughter 0.00001462768631526730000

hang v continue 0.00464202954343608000000

hang v hesitate 0.00013030781006727200000

hang v obstruct 0.00000084664863871824900

hang v cling 0.03671047331266400000000

hang v haunt 0.02090222546015490000000

hang v threaten 0.00081145669218374400000

hang v remain 0.02148530474351860000000

hang v suspend 0.03057171366036550000000

hang v deadlock 0.00017555976801850100000

hang v straddle 0.00000084664863871824900

hang v strangle 0.00000760035293042724000

hang v attend 0.00134465649227220000000

hang v lower 0.00064663879137592500000

hang v set 0.03636569118004390000000

hang v depend 0.00002232168402766600000

hang v block 0.00025062234714025100000

hang v flounder 0.00000726382692739018000

hang v clutch 0.00000816468686408985000

hang v pend 0.00060299296111952600000

hang v disconnect 0.00009279906342768370000

hang v overhang 0.02019732423628360000000

hang v project 0.00004543001306774360000

hang v float 0.00195441596622734000000

hang v stick 0.02458301778797950000000

hang v approach 0.00001787670895070430000

hang v flow 0.00776052516574237000000

hang v dangle 0.01631576591673960000000

hang v shrink 0.00011482988357061600000

hang v restrict 0.00013033376170707800000

hang v drift 0.00034824765823579600000

hang v rely 0.00061209754413239600000

hang v persevere 0.00000084664863871824900

hang v stall 0.00032452359207905300000

hang v absorb 0.00104391800252835000000

hang v freeze 0.00093664417331684800000

hang v execute 0.24512179030930100000000

hang v lean 0.02447449552374810000000

hang v doubt 0.00086820551044419900000

hang v hover 0.00000338659455487300000

hang v hamper 0.00003604489383489320000

hang v lynch 0.00009789459550043760000

hang v trail 0.00040120735860835000000

hang v display 0.00005661227810976380000

hang v stretch 0.00225393247817405000000

hang v persist 0.00005634556567683380000

hang v linger 0.00118770835413284000000

hang v attach 0.00042133086504194700000

hang v flap 0.02447364887510940000000

hang v near 0.00000143911848024867000

hang v loom 0.00243899777552192000000

hang v stalemate 0.00000167381245939945000

hang v drop 0.00505139890644010000000

hang v cover 0.01130944404782630000000

hang v hinder 0.00000084664863871824900

hang v wave 0.01319825453857860000000

hang v fall 0.00452757209566674000000

hang v thwart 0.00003067352606909900000

hang v hold 0.39230708841152800000000

hang v stay 0.01941539024129100000000

hang v idle 0.00002607129676396750000

hang v adhere 0.00018602022155848600000

hang v fix 0.00245963173373537000000

hang v extend 0.02041857035468790000000

hang v overlap 0.00000084664863871824900

hang v pause 0.00000816468686408985000

forestry n conservation 1.00000000000000000000000

fashion n quality 0.00066633258859609300000

fashion n type 0.00042504529362571400000

fashion n form 0.00024984660814795400000

fashion n formality 0.00001050159910979860000

fashion n structure 0.00020172927068241600000

fashion n kind 0.00045119855072367800000

fashion n system 0.02882463496463880000000

fashion n practice 0.01389676742679960000000

fashion n procedure 0.01218490463018620000000

fashion n approach 0.02450871386303300000000

fashion n figure 0.00490883819320181000000

fashion n vogue 0.00031175545126215100000

fashion n ceremony 0.00001188199562652300000

fashion n way 0.26769011124876900000000

fashion n convention 0.00007948413923575590000

fashion n method 0.00005314243298799580000

fashion n order 0.00337216518119792000000

fashion n design 0.12355470284954300000000

fashion n sort 0.00034318386871268000000

fashion n pattern 0.02201772325548710000000

fashion n fad 0.00001050159910979860000

fashion n line 0.01234797218899040000000

fashion n look 0.00034544219411735800000

fashion n style 0.11132308989927100000000

fashion n shape 0.00144507411087331000000

fashion n texture 0.00010319431313555500000

fashion n cut 0.00061994277898629400000

fashion n manner 0.23076398298633800000000

fashion n character 0.00009661386884826140000

fashion n protocol 0.00000309055284043658000

fashion n clothes 0.09169549923357880000000

fashion n trend 0.02295358291476070000000

fashion n stamp 0.00000875406948152762000

fashion n tendency 0.00003148375451249520000

fashion n thing 0.02395004858469900000000

fashion n precedent 0.00046766342348529400000

fashion n prescription 0.00007140011540478370000

remainder n claim 0.00089498493100137000000

remainder n carryover 0.00000866411437262081000

remainder n rest 0.69962155765457200000000

remainder n surplus 0.00613087784578863000000

remainder n rubble 0.00013993517838502600000

remainder n garbage 0.00005328285426516550000

remainder n right 0.00112389454694671000000

remainder n truck 0.00045658126732408400000

remainder n end 0.14550245395238600000000

remainder n precipitation 0.00027647320097429500000

remainder n remnant 0.00426924235710891000000

remainder n difference 0.01147303962698030000000

remainder n title 0.00065777443466846800000

remainder n estate 0.00006298980043871720000

remainder n coal 0.00256665343045198000000

remainder n appendix 0.00000609456439273220000

remainder n salvage 0.00003236798754414140000

remainder n skeleton 0.00004798178980006340000

remainder n relic 0.00007347806129974270000

remainder n fragment 0.00000319733559790969000

remainder n trash 0.00004578275986175780000

remainder n train 0.00109165742635258000000

remainder n waste 0.00015513135030357100000

remainder n wake 0.00053801906235965800000

remainder n sinter 0.00000598768173140785000

remainder n sediment 0.00000147691431238955000

remainder n slag 0.00012111325835769900000

remainder n consequence 0.00126048886768357000000

remainder n excess 0.00059356858043594000000

remainder n scrap 0.00012412857490322400000

remainder n slough 0.00000356661851053375000

remainder n balance 0.10789041452131100000000

remainder n property 0.01155861667996430000000

remainder n carbon 0.00012208735963135400000

remainder n remains 0.00066208859515436200000

remainder n stuff 0.00021962381567433100000

remainder n trail 0.00002325506504099270000

remainder n junk 0.00003670241609196740000

remainder n debris 0.00062360674430207700000

remainder n deposit 0.00149691444118993000000

remainder n filings 0.00000723539883471168000

remainder n residue 0.00001700893369366100000

regulate v reconcile 0.00009033971839764260000

regulate v establish 0.01238424267515230000000

regulate v align 0.00893324745849825000000

regulate v organize 0.00000639666718867045000

regulate v order 0.00068532292239226500000

regulate v equate 0.00001497093393498800000

regulate v determine 0.01297215922584070000000

regulate v set 0.03121520642888590000000

regulate v adjust 0.02694932035798650000000

regulate v conduct 0.00718138318993854000000

regulate v rectify 0.00022552589737237300000

regulate v handle 0.00831977271782592000000

regulate v legislate 0.00001746235206846960000

regulate v discipline 0.00002257936219408060000

regulate v baffle 0.00000538110470730535000

regulate v distribute 0.00405774418640193000000

regulate v correct 0.11565418245136500000000

regulate v monitor 0.01430546990540590000000

regulate v measure 0.00077442605836227500000

regulate v allocate 0.00016971205811369600000

regulate v influence 0.01620523276755630000000

regulate v balance 0.01078363079901550000000

regulate v discharge 0.00006915284377608850000

regulate v oversee 0.13324107562971200000000

regulate v govern 0.20656817750843500000000

regulate v arrange 0.00039531601872282900000

regulate v centralize 0.00000639666718867045000

regulate v direct 0.00003129593323597400000

regulate v coordinate 0.00005474409928224010000

regulate v run 0.00687912424042549000000

regulate v operate 0.00351474430848830000000

regulate v improve 0.08319212260945690000000

regulate v equalize 0.00010325132321104000000

regulate v guide 0.00021929598079942200000

regulate v manage 0.02338435018857840000000

regulate v administer 0.00071440386820144800000

regulate v adapt 0.00038793803605871400000

regulate v supervise 0.00271105585595198000000

regulate v dispose 0.00002451487117067460000

regulate v shape 0.00000257587369966445000

regulate v allot 0.00003340837702698980000

regulate v instruct 0.00026180919906265100000

regulate v police 0.03431949970474710000000

regulate v maintain 0.00590535004456809000000

regulate v overlook 0.00031642609805707800000

regulate v rule 0.00128981732490127000000

regulate v temper 0.00002983974077533740000

regulate v fix 0.00609552817107628000000

regulate v control 0.21491358637554000000000

regulate v classify 0.00002092210363562790000

regulate v settle 0.00434056776561106000000

decompose v resolve 0.28571428571428600000000

decompose v disintegrate 0.14285714285714300000000

decompose v reduce 0.14285714285714300000000

decompose v crumble 0.42857142857142900000000

deficit n indebtedness 0.00592622576877408000000

deficit n deficiency 0.00102579881251878000000

deficit n lack 0.00063521687688355900000

deficit n shortcoming 0.00167534457242040000000

deficit n shortage 0.03867447052932340000000

deficit n debt 0.56171316531141700000000

deficit n shortfall 0.22246712034931900000000

deficit n default 0.00034966572552601700000

deficit n arrears 0.00198609704219912000000

deficit n loss 0.16554689501161800000000

heifer n stick 1.00000000000000000000000

min n minute 1.00000000000000000000000

antagonist n rival 0.50000000000000000000000

antagonist n competitor 0.50000000000000000000000

amalgamate v combine 0.00039375814987798500000

amalgamate v unify 0.00003450329811735850000

amalgamate v merge 0.99736958702943700000000

amalgamate v consolidate 0.00031052968305622900000

amalgamate v mix 0.00134562862657700000000

amalgamate v unite 0.00018673836117383200000

amalgamate v incorporate 0.00013801319246943400000

amalgamate v join 0.00003450329811735850000

amalgamate v temper 0.00018673836117383200000

mild a patient 0.00001396982806907500000

mild a sunny 0.05842071336672920000000

mild a passive 0.00021446207341594600000

mild a flat 0.01278837288452030000000

mild a quiet 0.04624471601325430000000

mild a light 0.11065144860874600000000

mild a lukewarm 0.00040294795009156900000

mild a considerate 0.00002482432555467190000

mild a moderate 0.25966051187500000000000

mild a soft 0.00205122336146468000000

mild a lenient 0.00014428533042507800000

mild a gentle 0.00939279730965258000000

mild a obliging 0.00001416711509751520000

mild a modest 0.14294302071000500000000

mild a fair 0.00101937597946026000000

mild a tolerant 0.00026798660615448600000

mild a slight 0.00403058849951266000000

mild a neutral 0.00029977812731186300000

mild a tepid 0.00000081111775851062400

mild a humane 0.00029400094653777600000

mild a sensitive 0.00025144911954828900000

mild a easy 0.00352418806728039000000

mild a kind 0.00004964865110934390000

mild a reasonable 0.00075302320190504300000

mild a peaceful 0.00047453072023870400000

mild a average 0.03071954818211460000000

mild a medium 0.00541074818916639000000

mild a lovely 0.00000261417944992613000

mild a calm 0.01187488845274410000000

mild a fine 0.00553981334576644000000

mild a gracious 0.00000261417944992613000

mild a temperate 0.00721676427216405000000

mild a tropical 0.00293082193579846000000

mild a mild-mannered 0.00070516016500414100000

mild a forgiving 0.00003899144065218710000

mild a warm 0.07447997646333550000000

mild a cool 0.12317599755043800000000

mild a sweet 0.00003446789061786390000

mild a balmy 0.00066046691510951100000

mild a benign 0.01284254850976970000000

mild a clear 0.02006446351187190000000

mild a good-natured 0.00000081111775851062400

mild a smooth 0.00121215499074252000000

mild a delicate 0.00000261417944992613000

mild a vague 0.00045822107135893300000

mild a weak 0.04044351771872680000000

mild a tame 0.00824995394966672000000

vocal n song 1.00000000000000000000000

unfavourably r unfavorably 1.00000000000000000000000

tug v pull 1.00000000000000000000000

synchronise v synchronize 1.00000000000000000000000

russia n siberia 0.94470771635291600000000

russia n ussr 0.05529228364708450000000

sixty n 60 1.00000000000000000000000

repayment n restitution 0.00013560476715320100000

repayment n reimbursement 0.00016774456353807300000

repayment n indemnity 0.02306094670207380000000

repayment n compensation 0.00306849003034740000000

repayment n retribution 0.00013560476715320100000

repayment n vengeance 0.00027120953430640700000

repayment n refund 0.60436483729465800000000

repayment n return 0.03074373466493640000000

repayment n retaliation 0.00040681430145960800000

repayment n revenge 0.33764501337437400000000

sexuality n sex 1.00000000000000000000000

self-government n independence 0.18988692127160200000000

self-government n autonomy 0.81011307872839800000000

seduce v make 1.00000000000000000000000

softback n paperback 1.00000000000000000000000

pry v intervene 0.28571428571428600000000

pry v examine 0.14285714285714300000000

pry v ask 0.14285714285714300000000

pry v interfere 0.42857142857142900000000

plurality n bulk 0.00927548180974953000000

plurality n quantity 0.00927548180974953000000

plurality n lot 0.01855096361949910000000

plurality n majority 0.94434710914150300000000

plurality n world 0.01855096361949910000000

dress v align 0.00165932555137539000000

dress v heal 0.00046808492347883800000

dress v set 0.08154463566079680000000

dress v adjust 0.02209735978173080000000

dress v do 0.07307288497058120000000

dress v treat 0.01907126233957190000000

dress v clothe 0.09701397683893620000000

dress v groom 0.00160182417088218000000

dress v work 0.01107021067730820000000

dress v wear 0.68269394113730400000000

dress v bind 0.00008220743966417710000

dress v gear 0.00112350167541042000000

dress v cover 0.00078536039446165300000

dress v prepare 0.00741049375748408000000

dress v fix 0.00030493068101463900000

pickle n dilemma 0.31490741275989000000000

pickle n solution 0.51105555234406600000000

pickle n predicament 0.14087037786384600000000

pickle n impasse 0.03316665703219830000000

optimisation n optimization 1.00000000000000000000000

endorse v back 0.11791038851435500000000

endorse v prove 0.00010843158675418300000

endorse v encourage 0.01451855097882400000000

endorse v defend 0.00013139013218146700000

endorse v recommend 0.02177933586820040000000

endorse v note 0.00119113560241939000000

endorse v initial 0.00002107729866872970000

endorse v guarantee 0.00027044059687061500000

endorse v assign 0.00100589030262946000000

endorse v favor 0.00004269410403014780000

endorse v substantiate 0.00002107729866872970000

endorse v sustain 0.00113127406052007000000

endorse v assist 0.00009466566429878020000

endorse v countenance 0.00003338587989335150000

endorse v corroborate 0.00174455936804736000000

endorse v praise 0.00024284031073264600000

endorse v aid 0.00001453955255473640000

endorse v ratify 0.07638476980290330000000

endorse v sign 0.06385309952451200000000

endorse v subscribe 0.00000653807860737650000

endorse v approve 0.49496724935439200000000

endorse v uphold 0.00023975807189383500000

endorse v support 0.00714593625288561000000

endorse v underwrite 0.00001453955255473640000

endorse v confirm 0.02005297076975140000000

endorse v advocate 0.00009475856591833370000

endorse v certify 0.00082703330248265700000

endorse v affirm 0.00001453955255473640000

endorse v warrant 0.00009811042484373600000

endorse v accept 0.10051995048437800000000

endorse v sanction 0.00270445107743572000000

endorse v pass 0.07232620890095460000000

endorse v champion 0.00048840916428251400000

marine n sailor 0.00100602228183178000000

marine n soldier 0.18123101584028200000000

marine n naval 0.00051850852675154000000

marine n flotilla 0.00000670774290752302000

marine n shipping 0.41834474857476200000000

marine n armada 0.00000780165344768576000

marine n fleet 0.00069627738319312600000

marine n navy 0.19321799067427500000000

marine n tonnage 0.00000670774290752302000

marine n maritime 0.20496421957964100000000

misread v mistranslate 0.01999999999999980000000

misread v misunderstand 0.50000000000000100000000

misread v misinterpret 0.40000000000000000000000

misread v misconstrue 0.07999999999999920000000

false a fallacious 0.01382076820201580000000

false a specious 0.00192623947066422000000

false a dubious 0.00022489663314482400000

false a incorrect 0.04235096914966190000000

false a fake 0.10492541448873300000000

false a tricky 0.00103771131155958000000

false a invalid 0.01178925043553720000000

false a made-up 0.00064886235690214800000

false a flawed 0.00032310387380516100000

false a inaccurate 0.10161115267206600000000

false a untrue 0.35198398205388600000000

false a erroneous 0.00812969719655578000000

false a deceptive 0.00008935360078885220000

false a bogus 0.00110162010097267000000

false a malicious 0.00534997478787707000000

false a duplicitous 0.00307128182267017000000

false a wrong 0.12694509750864800000000

false a fictitious 0.00008405976639809870000

false a hollow 0.09213845468010510000000

false a lifeless 0.00012425655352578100000

false a disappointing 0.01267463570142850000000

false a simulated 0.00005883681652624840000

false a phony 0.02010293556656840000000

false a imaginary 0.00125074825357609000000

false a dishonest 0.00089149833485148300000

false a hypocritical 0.00041676922535609000000

false a unsound 0.00013711079565491800000

false a queer 0.00009647332264425340000

false a fanciful 0.00173492056016234000000

false a counterfeit 0.00014750019425843900000

false a crooked 0.00003768957786205550000

false a contrived 0.00008182811250453370000

false a spurious 0.00418811157636841000000

false a illusory 0.00015373490769761700000

false a affected 0.00011911181167006900000

false a sour 0.00012989443798887000000

false a deceitful 0.00007316446904190450000

false a insincere 0.00100514677832842000000

false a corrupt 0.00147867373130424000000

false a flash 0.00445831232323089000000

false a suspicious 0.00001554117643316730000

false a fraudulent 0.00527336773456912000000

false a misleading 0.07118606464666130000000

false a questionable 0.00029192633738119000000

false a artificial 0.00631985694241468000000

pound n cooler 0.00000163565392956849000

pound n can 0.00078233974457850700000

pound n punch 0.00000537933383723560000

pound n quid 0.00000272977378786094000

pound n hammer 0.00134404664831031000000

pound n wallop 0.00009603433686474710000

pound n cage 0.00000883467417935759000

pound n bop 0.00002862489197721910000

pound n jail 0.00255149009762966000000

pound n hit 0.00247931074084047000000

pound n hammering 0.00000170219222686316000

pound n pint 0.00001631593990679190000

pound n buffet 0.00000083052308056277100

pound n enclosure 0.00045062265759612100000

pound n blow 0.00080855668014009400000

pound n coop 0.00006276529873660360000

pound n t 0.00028223307114327600000

pound n yard 0.00035227417309602600000

pound n corral 0.00000266035174784267000

pound n pen 0.00010580987338995300000

pound n compound 0.00012728421737600600000

pound n brig 0.00000388879036331582000

pound n strike 0.01181867187322690000000

pound n stroke 0.00338688466107862000000

pound n punt 0.08780709258094300000000

pound n lb 0.88747198122001300000000

aid v back 0.02722952189119790000000

aid v collaborate 0.00847140681059489000000

aid v protect 0.00955583472278290000000

aid v combine 0.00037361441483810600000

aid v side 0.00726120583765278000000

aid v benefit 0.07228933811707640000000

aid v encourage 0.00001765667134809540000

aid v expedite 0.00000141678115909157000

aid v promote 0.02359891897237150000000

aid v subsidize 0.00000141678115909157000

aid v help 0.45819054930250400000000

aid v better 0.00000141678115909157000

aid v sustain 0.00021681238012013300000

aid v abet 0.03049706451814170000000

aid v assist 0.04151131015307410000000

aid v accelerate 0.00000425034347727472000

aid v enhance 0.00000566712463636629000

aid v cooperate 0.01270852699705140000000

aid v bolster 0.05541929635165840000000

aid v speed 0.00000141678115909157000

aid v endorse 0.00000850068695454945000

aid v join 0.00847849071639035000000

aid v improve 0.00063263002695600400000

aid v uphold 0.00000850068695454945000

aid v facilitate 0.03600489572618740000000

aid v boost 0.08655841736431350000000

aid v support 0.10807801096002300000000

aid v relieve 0.00000283356231818313000

aid v maintain 0.00007732155884452780000

aid v accommodate 0.00000141678115909157000

aid v ease 0.01278667307210000000000

aid v serve 0.00000566712463636629000

hazy a confused 0.00127310161109338000000

hazy a unclear 0.70335195490562200000000

hazy a uncertain 0.12192192175854000000000

hazy a dull 0.00594114085176910000000

hazy a thick 0.00021218360184889500000

hazy a vague 0.16729969727112700000000

sick a crazy 0.00073068882249413800000

sick a infected 0.00246579591027871000000

sick a grim 0.00110939829444705000000

sick a down 0.00021663838272148000000

sick a disgusted 0.00002758260575764200000

sick a invalid 0.00014850846652431800000

sick a cruel 0.00015755518743380400000

sick a disordered 0.00004477095061001350000

sick a frail 0.00006353889855024590000

sick a black 0.00883806612873201000000

sick a unhealthy 0.00001099581648290990000

sick a impaired 0.00146351262994067000000

sick a unbalanced 0.00000450419371113286000

sick a disturbed 0.00237532491392114000000

sick a diseased 0.00003402638509291130000

sick a annoyed 0.00002758260575764200000

sick a peaked 0.00030951572591434400000

sick a injured 0.02940513383010470000000

sick a mad 0.00175354572091513000000

sick a unwell 0.00000616533124389907000

sick a ailing 0.11210515483075600000000

sick a ill 0.76912523150551700000000

sick a corrupt 0.00041973703678174400000

sick a delicate 0.00015584608152106200000

sick a weak 0.06213220969798970000000

sick a tired 0.00114104233812369000000

sick a disabled 0.00572792770867720000000

prepared a ready 0.29200715671026700000000

prepared a armed 0.00451168546516414000000

prepared a planned 0.00019285081706697500000

prepared a inclined 0.00011625022342035100000

prepared a willing 0.51836878252815000000000

prepared a disposed 0.00012027027970257800000

prepared a frozen 0.00000394435564490519000

prepared a able 0.18445759556585600000000

prepared a fit 0.00022146405472745100000

controversy n dispute 0.26794587428313400000000

controversy n discussion 0.04688901238478840000000

controversy n wrangling 0.00222391919743824000000

controversy n debate 0.18379274576970700000000

controversy n spat 0.00126935502664054000000

controversy n brawl 0.00012223067344698700000

controversy n squabble 0.00016327890776569000000

controversy n row 0.40680267910582700000000

controversy n storm 0.07393558623222560000000

controversy n bickering 0.00196064331288126000000

controversy n quarrel 0.00000235390718298762000

controversy n argument 0.00649944330515583000000

controversy n hassle 0.00019281332477233600000

controversy n disagreement 0.00799961085971841000000

controversy n strife 0.00020045370931495900000

eerie a strange 0.06018382420465950000000

eerie a alarming 0.01905231755668070000000

eerie a fantastic 0.19560846326759700000000

eerie a awful 0.07620927022672270000000

eerie a odd 0.05065766542631910000000

eerie a bizarre 0.28089834809817200000000

eerie a curious 0.00952615877834033000000

eerie a frightening 0.07620927022672270000000

eerie a horrific 0.01905231755668070000000

eerie a scary 0.09828845931802080000000

eerie a awesome 0.07620927022672270000000

eerie a dreadful 0.00952615877834033000000

eerie a abnormal 0.02857847633502100000000

edo n tokyo 1.00000000000000000000000

draft v draw 0.14369935623169200000000

draft v write 0.39062306564180900000000

draft v haul 0.00003197373888029980000

draft v enlist 0.00239442715788445000000

draft v pull 0.00022001496560882900000

draft v frame 0.00747580579360465000000

draft v design 0.01278595078087330000000

draft v plan 0.25500671511203800000000

draft v tow 0.00009686670482371380000

draft v select 0.02016522873551620000000

draft v drag 0.00011984737391055700000

draft v outline 0.00103622722586411000000

draft v delineate 0.00040330715910665500000

draft v formulate 0.16453626258863800000000

draft v trace 0.00116003986574811000000

draft v impress 0.00018400317021201500000

draft v compose 0.00001446700745502530000

draft v conscript 0.00004644074633532510000

credibility n credence 0.01025401508580490000000

credibility n assurance 0.00371364681222458000000

credibility n reliance 0.00009362862228824590000

credibility n belief 0.00095990980264837900000

credibility n faith 0.03252399351788370000000

credibility n likelihood 0.00084294556430706300000

credibility n integrity 0.00390856947098540000000

credibility n reliability 0.06494209554343100000000

credibility n confidence 0.88276119558042700000000

cheat n fraud 0.28324426281043700000000

cheat n dodger 0.71675573718956300000000

judgment n result 0.01890031905152970000000

judgment n observation 0.00000273720846783800000

judgment n appraisal 0.00000073266309114411800

judgment n study 0.00050155834686382100000

judgment n opinion 0.49869791178162000000000

judgment n know-how 0.00000024055271696650100

judgment n review 0.00011020678389157300000

judgment n idea 0.00132779184662983000000

judgment n obligation 0.00007624620269454630000

judgment n breadth 0.00001274546730354180000

judgment n mentality 0.00000466566879910021000

judgment n diagnosis 0.00000069459630549654500

judgment n award 0.00628969832776475000000

judgment n exploration 0.00001528672896024890000

judgment n recommendation 0.00215827391897889000000

judgment n penalty 0.00011861121806799400000

judgment n correction 0.00151836633597835000000

judgment n tariff 0.00005064161035279640000

judgment n resolution 0.00052560603234866100000

judgment n apprehension 0.00000095424190093062200

judgment n inquiry 0.00022441732543418700000

judgment n outcome 0.00142741593243607000000

judgment n reasoning 0.00000064655018047828200

judgment n experience 0.00004497335700630950000

judgment n debt 0.00011700900063873200000

judgment n sentence 0.01129123430961400000000

judgment n search 0.00004340335246553660000

judgment n retribution 0.00000073266309114411800

judgment n sense 0.00685141110149650000000

judgment n genius 0.00000048110543393300700

judgment n consideration 0.00007918195883929340000

judgment n estimation 0.00000466566879910021000

judgment n summary 0.00001651750794949500000

judgment n decision 0.06209002936274050000000

judgment n intelligence 0.00000064655018047828200

judgment n mind 0.00017288152850257300000

judgment n misfortune 0.00000088710289744478300

judgment n analysis 0.00003778019818798350000

judgment n assessment 0.00081399999675572600000

judgment n damages 0.00446587838901076000000

judgment n decree 0.00018351135532319400000

judgment n conclusion 0.00088362342175585900000

judgment n inference 0.00000544248770879315000

judgment n intellect 0.00000036723852063183700

judgment n understanding 0.00397561745371587000000

judgment n inquest 0.00000339778252740580000

judgment n verdict 0.02237350345565100000000

judgment n inspection 0.00001670230093364960000

judgment n finding 0.00030030398139239100000

judgment n fate 0.00008376454070273620000

judgment n belief 0.00020024519208670000000

judgment n range 0.00013688757578028400000

judgment n perception 0.00009537036844999490000

judgment n knowledge 0.00002473198721204650000

judgment n call 0.00489306564421899000000

judgment n interpretation 0.00002672612652290680000

judgment n appreciation 0.00000069459630549654500

judgment n determination 0.00015610481453917800000

judgment n conviction 0.00178709146410853000000

judgment n upshot 0.00001182086341272060000

judgment n punishment 0.00076419518593771600000

judgment n estimate 0.00025816271666630900000

judgment n sanity 0.00000064655018047828200

judgment n comment 0.00865622257299897000000

judgment n regard 0.00003277401608828720000

judgment n commentary 0.00019926962068066800000

judgment n intuition 0.00005933175489522440000

judgment n discrimination 0.00000974059403343074000

judgment n evaluation 0.00015072761528179200000

judgment n reach 0.00003300654710814180000

judgment n adjudication 0.00004580760488234230000

judgment n examination 0.00005096877889448340000

judgment n depth 0.00000030769172045234000

judgment n capacity 0.00025756598368116000000

judgment n scrutiny 0.00003666941556649570000

judgment n judgement 0.27760553464304000000000

judgment n supposition 0.00001066143912834890000

judgment n view 0.00095352882831298800000

judgment n reason 0.00149290315764428000000

judgment n taste 0.00015427960164926700000

judgment n pursuit 0.00000256522779406422000

judgment n ruling 0.04041579565111610000000

judgment n quest 0.00001572807356203290000

judgment n penetration 0.00000046851427118886900

judgment n report 0.01563668401807100000000

blacklist v reject 0.09314655010483250000000

blacklist v relegate 0.00094083380360334300000

blacklist v exclude 0.00313611267867785000000

blacklist v avoid 0.00909472676816576000000

blacklist v eject 0.00031361126786778100000

blacklist v ban 0.73122513456705700000000

blacklist v discard 0.00031361126786778100000

blacklist v ignore 0.00595861408948791000000

blacklist v bar 0.00188166760720670000000

blacklist v oust 0.00094083380360334300000

blacklist v discharge 0.00282250141081005000000

blacklist v cut 0.06460592295481390000000

blacklist v dump 0.00156805633933892000000

blacklist v expel 0.00219527887507448000000

blacklist v isolate 0.00062722253573556200000

blacklist v excommunicate 0.00094083380360334300000

blacklist v boycott 0.00031361126786778100000

blacklist v deport 0.07966126558651780000000

blacklist v outlaw 0.00031361126786778100000

illness n bug 0.00002696943088141430000

illness n syndrome 0.05092450151164640000000

illness n disability 0.01371845050834610000000

illness n ailment 0.09347970707666110000000

illness n hurt 0.00024545935303203700000

illness n weakness 0.00497800263020121000000

illness n flu 0.00303557813007815000000

illness n disease 0.53726317564012800000000

illness n sickness 0.15358162663614200000000

illness n handicap 0.00012104515154423000000

illness n malaise 0.00007428763813183790000

illness n complaint 0.00419114090435323000000

illness n collapse 0.00111580060421699000000

illness n madness 0.00000085892416831967800

illness n convalescence 0.01159007326036000000000

illness n breakdown 0.00045912008221524900000

illness n pain 0.00299642965008281000000

illness n relapse 0.00000177967548497041000

illness n virus 0.02129003253911650000000

illness n wound 0.00386003205380898000000

illness n attack 0.01188408918585330000000

illness n infection 0.01408373469347950000000

illness n fit 0.00000177967548497041000

illness n disorder 0.06333394264228190000000

illness n disturbance 0.00774238240230051000000

management n regulation 0.01946744960998100000000

management n managers 0.33594931506663100000000

management n organization 0.00855278623386845000000

management n direction 0.00436014211156067000000

management n care 0.02472031005901700000000

management n government 0.21216688788629100000000

management n treatment 0.00815733521799971000000

management n leadership 0.00687775396495809000000

management n operation 0.05657928419221430000000

management n board 0.08348235842643770000000

management n address 0.00000731759528004136000

management n executive 0.06444757374919170000000

management n charge 0.01231699093912520000000

management n administrators 0.00001527524858866940000

management n command 0.00012645322520316600000

management n capability 0.00391699508857352000000

management n managing 0.00002873854143991430000

management n supervision 0.00080164582962521700000

management n skill 0.00007625940174956630000

management n rule 0.00954093776596182000000

management n control 0.08246617752824960000000

management n conduct 0.00079705383532633000000

management n running 0.00666031062091856000000

management n controllers 0.00051332509019581100000

management n directors 0.00008214244521802290000

management n guidance 0.00037136187579352400000

management n directorate 0.00001081500494727670000

management n oversight 0.00296498699400001000000

management n administration 0.05280333469453880000000

management n governance 0.00173868175711478000000

circulation n sterling 0.00696305284951648000000

circulation n distribution 0.13209085652834800000000

circulation n flow 0.29528734595705000000000

circulation n tour 0.00021215919056763200000

circulation n currency 0.08420017468958050000000

circulation n change 0.01378008886856200000000

circulation n money 0.05910703760369830000000

circulation n current 0.00020848745597125700000

circulation n vogue 0.00003375174425623120000

circulation n broadcasting 0.00056283491623248000000

circulation n turn 0.00035532672196594400000

circulation n declaration 0.00117175232955263000000

circulation n transmission 0.00059705761021666900000

circulation n acceptance 0.00189674323606881000000

circulation n publication 0.00025505891347194200000

circulation n publishing 0.00106777525197471000000

circulation n reporting 0.00000445795853758691000

circulation n revolution 0.00009616237717821200000

circulation n circuit 0.00008051749354455400000

circulation n issuance 0.05210194001082580000000

circulation n passage 0.00140169295893847000000

circulation n issuing 0.00072547409464310500000

circulation n advertisement 0.05074883950322050000000

circulation n printing 0.00147320493295444000000

circulation n utterance 0.00000262266420667336000

circulation n curling 0.00000445795853758691000

circulation n coin 0.21048693905576100000000

circulation n publicity 0.00000524532841334673000

circulation n journey 0.00001524479470349100000

circulation n round 0.00332486569767086000000

circulation n announcement 0.08107611879390510000000

circulation n orbit 0.00033960230790011000000

circulation n notification 0.00000708062274426027000

circulation n rolling 0.00031602957928110600000

annihilate v suppress 0.00055327566362091600000

annihilate v overwhelm 0.00018442522120697200000

annihilate v extinguish 0.00036885044241394400000

annihilate v demolish 0.00018442522120697200000

annihilate v massacre 0.00018442522120697200000

annihilate v rescind 0.00018442522120697200000

annihilate v withdraw 0.00055327566362091600000

annihilate v trample 0.00073770088482788800000

annihilate v cancel 0.00189070282786591000000

annihilate v destroy 0.98612165779368100000000

annihilate v eliminate 0.00092212610603486000000

annihilate v crush 0.00018442522120697200000

annihilate v execute 0.00073770088482788800000

annihilate v abolish 0.00110655132724183000000

annihilate v kill 0.00073770088482788800000

annihilate v murder 0.00018442522120697200000

annihilate v liquidate 0.00239752787569064000000

annihilate v break 0.00129097654844881000000

annihilate v dismantle 0.00055327566362091600000

annihilate v ruin 0.00055327566362091600000

annihilate v slaughter 0.00036885044241394400000

amazingly r surprisingly 1.00000000000000000000000

selection n diversity 0.00000653386884523938000

selection n excerpt 0.07088664491450180000000

selection n choice 0.01538083425538840000000

selection n set 0.00029905853216739700000

selection n election 0.10681725478921600000000

selection n quote 0.00036310489940210300000

selection n picking 0.02395738096775900000000

selection n group 0.03434394739425950000000

selection n variety 0.00001306773769047880000

selection n differentiation 0.00003887938375370860000

selection n piece 0.00001306773769047880000

selection n collection 0.41521457034922300000000

selection n preference 0.00029019240132118400000

selection n range 0.00277360883688654000000

selection n lot 0.00172331525288412000000

selection n adoption 0.00000591269789548922000

selection n passage 0.00067867365452109100000

selection n determination 0.00428584485221506000000

selection n option 0.01424235631213490000000

selection n series 0.17066692832194400000000

selection n reservation 0.00006932347599935400000

selection n discrimination 0.00001182539579097840000

selection n survival 0.01642358954564470000000

selection n batch 0.00000832961659597689000

selection n pick 0.12026656491549700000000

selection n modification 0.00089169148820717400000

selection n separation 0.00010504520565901800000

selection n array 0.00009597117874741590000

selection n line-up 0.00008760263440394900000

selection n quotation 0.00001665923319195410000

selection n appropriation 0.00002222015056175460000

overall r largely 0.05021861530520030000000

overall r primarily 0.00383632343847547000000

overall r altogether 0.16584691816584100000000

overall r principally 0.00099064750272057600000

overall r mainly 0.71074034576503000000000

overall r mostly 0.06836714982273230000000

consult v refer 0.01588415718406150000000

consult v negotiate 0.01037996206711470000000

consult v confer 0.00003020276578769610000

consult v argue 0.02899951902419420000000

consult v regard 0.01830769089258970000000

consult v promote 0.00078835035412225200000

consult v review 0.00580722505775702000000

consult v interrogate 0.00029892570644493800000

consult v question 0.05302541512354010000000

consult v canvass 0.00008183950146006990000

consult v examine 0.02970578178564640000000

consult v check 0.00398061306146582000000

consult v ask 0.23352837274194900000000

consult v respect 0.01490854718904070000000

consult v consider 0.19133540964168300000000

consult v debate 0.03512598172174640000000

consult v interview 0.02551567041303670000000

consult v discuss 0.22384104844578800000000

consult v communicate 0.10845528732257100000000

desire n excitement 0.00262953619057792000000

desire n disposition 0.08020085381262660000000

desire n frenzy 0.00021778914816735000000

desire n hope 0.11431684834653000000000

desire n requirement 0.00699834807289524000000

desire n application 0.09626111035717740000000

desire n request 0.00001004289101274670000

desire n appeal 0.00186848376551494000000

desire n demand 0.13268133214693400000000

desire n will 0.11113110248584300000000

desire n order 0.00610402981369650000000

desire n liking 0.00002678104270065790000

desire n heat 0.00021778914816735000000

desire n rut 0.00029897801980475900000

desire n call 0.00139534205903960000000

desire n love 0.00003625842080885790000

desire n ambition 0.01270019675868240000000

desire n hunger 0.00000334763033758218000

desire n wish 0.31325119750381800000000

desire n prayer 0.00002194052698085030000

desire n motive 0.00003347630337582240000

desire n appetite 0.05347058350542200000000

desire n burning 0.00217428590425967000000

desire n yen 0.00002008578202549340000

desire n need 0.06393026036360120000000

untoward a troublesome 0.00724345691217950000000

untoward a sinister 0.03425477160439570000000

untoward a hostile 0.01817173859501480000000

untoward a unusual 0.61308904761117300000000

untoward a unexpected 0.00856369290109892000000

untoward a surprising 0.00856369290109892000000

untoward a improper 0.03279267769445200000000

untoward a unfortunate 0.27732092178058700000000

anticipation n outlook 0.07713328467712790000000

anticipation n excitement 0.00001293031781040630000

anticipation n hope 0.24779092522380800000000

anticipation n apprehension 0.00000431010593680211000

anticipation n prospect 0.02221089678527790000000

anticipation n promise 0.00002555852023921040000

anticipation n forecast 0.02153821159088220000000

anticipation n preoccupation 0.00000173348745712434000

anticipation n feeling 0.00085361929041050900000

anticipation n suggestion 0.00007591827375318900000

anticipation n assurance 0.00051095712123966200000

anticipation n prediction 0.00002818236413090570000

anticipation n faith 0.00002729200769633470000

anticipation n thought 0.00015841038702792300000

anticipation n expectation 0.62446901023075000000000

anticipation n hint 0.00301986334509670000000

anticipation n trust 0.00092694253047982800000

anticipation n prevention 0.00000173348745712434000

anticipation n certainty 0.00013096256976427800000

anticipation n provision 0.00005261162767619680000

anticipation n confidence 0.00102233595004081000000

anticipation n joy 0.00000431010593680211000

travesty n atrocity 0.00680008552231541000000

travesty n corruption 0.00038742821963464700000

travesty n farce 0.99281248625805000000000

tombstone n cross 0.34876876806315900000000

tombstone n stone 0.65123123193684100000000

surmount v clear 0.84307692307692300000000

surmount v overcome 0.15692307692307700000000

suffocation n asphyxiation 1.00000000000000000000000

subversive n rebel 1.00000000000000000000000

sprawl v spread 0.35714285714285700000000

sprawl v stretch 0.64285714285714300000000

relax v allay 0.00010386906243240600000

relax v abate 0.00030939286208789000000

relax v mitigate 0.00001708687791321760000

relax v slow 0.00290783256484980000000

relax v downplay 0.00009514410900170380000

relax v lower 0.01484072259169600000000

relax v liberate 0.00023271425418325700000

relax v slacken 0.00010991293868736000000

relax v bend 0.00846410472711869000000

relax v ebb 0.00002427804108589840000

relax v blunt 0.00003842793367281430000

relax v dilute 0.00206968466839693000000

relax v weaken 0.04584976155845830000000

relax v halt 0.00084831731579479300000

relax v free 0.00867281866923314000000

relax v curb 0.00075410517948143300000

relax v screw 0.00000292629688792565000

relax v check 0.00068672550699706200000

relax v decrease 0.00155874644275495000000

relax v subdue 0.01110642776599680000000

relax v discontinue 0.00028441247260699000000

relax v modify 0.01401435386599300000000

relax v soften 0.04189436030508160000000

relax v mollify 0.00003726335414454770000

relax v reduce 0.04033017855194360000000

relax v release 0.02545598369680010000000

relax v rest 0.00287857248136542000000

relax v decline 0.03321308340595250000000

relax v slack 0.00127416630300712000000

relax v hush 0.00000614900452387840000

relax v calm 0.00007790191348553360000

relax v linger 0.00008889093435799000000

relax v moderate 0.00070207123349793000000

relax v relieve 0.00026082314593010400000

relax v cease 0.00005193460899035490000

relax v diminish 0.02153342759640720000000

relax v dwindle 0.00028623202101754500000

relax v wane 0.00346322205893861000000

relax v alleviate 0.00023336058914347200000

relax v flatten 0.00008787139499261060000

relax v ease 0.53685219026347100000000

relax v quell 0.00003074502261939230000

relax v stop 0.01727224545721630000000

relax v temper 0.00003895581920166710000

relax v idle 0.00003558190233936020000

relax v recede 0.02071629561485790000000

relax v loosen 0.13749483856049000000000

relax v subside 0.00267446910514358000000

relax v lessen 0.00001741994975003850000

seminary n institute 1.00000000000000000000000

dominant a primary 0.01508386461815150000000

dominant a rife 0.00010582484607978900000

dominant a central 0.00530971247236642000000

dominant a authoritative 0.00001733800937341160000

dominant a powerful 0.00662618036025965000000

dominant a major 0.19117329578605100000000

dominant a leading 0.01153874184168300000000

dominant a crucial 0.00474179232689393000000

dominant a aggressive 0.01710825175290090000000

dominant a predominant 0.02710277625251710000000

dominant a first 0.00115878216160913000000

dominant a prevailing 0.00028114913125017700000

dominant a principal 0.00082219768731357000000

dominant a strongest 0.02263709481987000000000

dominant a main 0.46343535525757200000000

dominant a key 0.10341267835667200000000

dominant a prominent 0.03643107461196340000000

dominant a chief 0.02277678070116320000000

dominant a influential 0.01696777641335440000000

dominant a sovereign 0.00013660673514373500000

dominant a outstanding 0.00507713237050913000000

dominant a superior 0.00004254554242171100000

dominant a pushy 0.00001023518740653960000

dominant a prime 0.00678116981394801000000

dominant a ruling 0.03444594888039750000000

dominant a core 0.00677569406312927000000

retrospect n consideration 0.00417107078022722000000

retrospect n today 0.99374339382965900000000

retrospect n view 0.00208553539011361000000

annual a year-long 0.00147298989200188000000

annual a twelve-month 0.00744308138518463000000

annual a yearly 0.87026853535510500000000

annual a seasonal 0.00473446424558565000000

annual a one-year 0.04252813789337520000000

annual a year-end 0.07355279122874790000000

austerity n resistance 0.00265677341306110000000

austerity n rigor 0.01152466031822030000000

austerity n trouble 0.00796482061362639000000

austerity n oppression 0.34327023947842000000000

austerity n rigidity 0.00870612926213384000000

austerity n burden 0.24028916763489400000000

austerity n simplicity 0.00087760835513109400000

austerity n control 0.02261849967174180000000

austerity n restraint 0.25286975232782000000000

austerity n hardship 0.10922234892495200000000

purposely r just 1.00000000000000000000000

erupt v detonate 0.00000123396368158475000

erupt v start 0.40633490919012300000000

erupt v blow 0.00000268291091909316000

erupt v explode 0.01747913852350800000000

erupt v expel 0.00000123396368158475000

erupt v appear 0.00071065242542347700000

erupt v break 0.24585593539257500000000

erupt v flare 0.32961421363008900000000

moor n heath 1.00000000000000000000000

deploy v place 0.15111206550402600000000

deploy v utilize 0.00069111665438830100000

deploy v use 0.26965314467362700000000

deploy v locate 0.00300587881565092000000

deploy v employ 0.00050169869197935700000

deploy v install 0.11482324837976500000000

deploy v base 0.06804388215185900000000

deploy v exploit 0.00121274669559614000000

deploy v position 0.00253419941286136000000

deploy v dispose 0.00003731148800777300000

deploy v post 0.04790849193292950000000

deploy v station 0.32435298877707500000000

deploy v expand 0.00353522177047023000000

deploy v extend 0.01258800505176400000000

modelling n model 0.00230149597238205000000

modelling n modeling 0.99769850402761800000000

constant a serious 0.01022536502329690000000

constant a stable 0.25610603961078900000000

constant a stubborn 0.00013449078879094900000

constant a firm 0.10040344247943900000000

constant a endless 0.00095975956965844700000

constant a irreversible 0.00013631935152094100000

constant a unbroken 0.00023128692743633800000

constant a regular 0.02884184608320970000000

constant a uninterrupted 0.00011564346371816900000

constant a eternal 0.00013449078879094900000

constant a recurrent 0.01671048050727540000000

constant a solid 0.01136343983082160000000

constant a fixed 0.05773565415776610000000

constant a true 0.00251117709328369000000

constant a same 0.08385874136791240000000

constant a steadfast 0.00442801376218024000000

constant a permanent 0.00031381184051221400000

constant a fast 0.00039473576001438000000

constant a consistent 0.00848973104242865000000

constant a loyal 0.00735606566930785000000

constant a sustained 0.00119818485253321000000

constant a persistent 0.00268580750600525000000

constant a continuous 0.00969773128804883000000

constant a frequent 0.01671048050727540000000

constant a steady 0.32873402631516600000000

constant a repeated 0.05013144152182620000000

constant a chronic 0.00039179289099271000000

cover n grove 0.00064201388737130500000

cover n jacket 0.00012007044412194100000

cover n mask 0.00081748712488525700000

cover n ambush 0.00094679414119040200000

cover n coating 0.00005516368465571940000

cover n screening 0.00005516368465571940000

cover n envelope 0.00019507830848569200000

cover n top 0.02032264661373180000000

cover n protection 0.19382687188624300000000

cover n cover-up 0.00042198457272645900000

cover n break 0.00162920782712828000000

cover n film 0.00390743045355071000000

cover n back 0.00264400781969135000000

cover n pall 0.00003245337973311090000

cover n facade 0.00005229755944114030000

cover n cap 0.00038702501644729500000

cover n sheet 0.00093182381053407400000

cover n shroud 0.00005516368465571940000

cover n ceiling 0.05297885359237400000000

cover n case 0.10290480884029200000000

cover n screen 0.00019481283317618400000

cover n cloak 0.46905681062759000000000

cover n covering 0.00013769464571483600000

cover n haze 0.00003245337973311090000

cover n surface 0.00317056956648496000000

cover n asylum 0.00787785490252860000000

cover n woods 0.00163102768022111000000

cover n haven 0.00003245337973311090000

cover n retreat 0.00045062342020733500000

cover n shingle 0.00001957870439852160000

cover n roof 0.04116476155107370000000

cover n umbrella 0.00127191286916309000000

cover n shelter 0.00003245337973311090000

cover n tarpaulin 0.00003245337973311090000

cover n spread 0.08531553368998860000000

cover n refuge 0.00041317490475894300000

cover n hat 0.00041317490475894300000

cover n tent 0.00008761706438883030000

cover n housing 0.00460393109707165000000

cover n seal 0.00069431144681244500000

cover n overhead 0.00003245337973311090000

cover n carpet 0.00009972505806901000000

cover n slate 0.00030827180301171100000

age v mature 0.04085024950064410000000

age v wither 0.00015993059035572400000

age v soften 0.00135075283198088000000

age v decline 0.13991044725986500000000

age v deteriorate 0.14276071742724300000000

age v fail 0.26607530749837300000000

age v develop 0.16174409099661100000000

age v fade 0.21612143268608200000000

age v wane 0.03102707120884420000000

innovator n pioneer 1.00000000000000000000000

unload v offload 0.32603633004066100000000

unload v discharge 0.58936907013017700000000

unload v dump 0.08150908251016530000000

unload v ease 0.00308551731899679000000

incarceration n imprisonment 1.00000000000000000000000

immaterial a insignificant 0.03447193324342200000000

immaterial a light 0.08829556140701450000000

immaterial a irrelevant 0.07872456436650720000000

immaterial a meaningless 0.01141272837823050000000

immaterial a slight 0.01793507553962560000000

immaterial a minor 0.01644965736070090000000

immaterial a small 0.07783819194241990000000

immaterial a unnecessary 0.11995624567176100000000

immaterial a foreign 0.55491604209031800000000

humility n respect 1.00000000000000000000000

holiday v vacation 1.00000000000000000000000

complex a labyrinthine 0.05675134088056780000000

complex a involved 0.00144590514444621000000

complex a disordered 0.00007716021873632600000

complex a mixed 0.01296612472873970000000

complex a confused 0.14258273560291800000000

complex a obscure 0.00027401398493836400000

complex a tortuous 0.00067294080095535100000

complex a deep 0.02980877354079460000000

complex a puzzling 0.00005758147379443750000

complex a combined 0.00101796127140032000000

complex a tangled 0.05675134088056790000000

complex a perplexing 0.00012017224114466500000

complex a sophisticated 0.01022184049286960000000

complex a aggregate 0.01621466882301940000000

complex a complicated 0.30336956531595300000000

complex a compact 0.00011516294758887500000

complex a difficult 0.36746637594028700000000

complex a knotty 0.00008633571127875440000

economist n statistician 0.00112676989283818000000

economist n banker 0.60672389289681500000000

economist n professor 0.39214933721034700000000

unusual a strange 0.00159863117738439000000

unusual a irregular 0.00000554146279330984000

unusual a great 0.00229878178261179000000

unusual a fantastic 0.00012624604250340200000

unusual a incredible 0.00006362384042572930000

unusual a exaggerated 0.00032180049421005600000

unusual a uncomfortable 0.00024501036251778600000

unusual a amazing 0.00069344618704972000000

unusual a mysterious 0.00004686745707426010000

unusual a inconsistent 0.00963167440719951000000

unusual a odd 0.00074071160265863100000

unusual a profound 0.00004989923034340380000

unusual a different 0.00168709624443711000000

unusual a bizarre 0.00009861649641510450000

unusual a fearful 0.00003293411078214860000

unusual a disturbing 0.00014386460579076800000

unusual a extraordinary 0.58547423920328700000000

unusual a outrageous 0.00010902888178043500000

unusual a neurotic 0.00000738620378722659000

unusual a freakish 0.00008348901968977810000

unusual a shocking 0.00004379611580769870000

unusual a exotic 0.00000895339896946389000

unusual a staggering 0.00003550637541889150000

unusual a phenomenal 0.00000784832221565903000

unusual a appalling 0.00012832169409270700000

unusual a distinctive 0.00000065874842151447500

unusual a individual 0.00340364391948648000000

unusual a curious 0.00002064745084493810000

unusual a astonishing 0.00000772629449583785000

unusual a alien 0.00028739747672481500000

unusual a funny 0.00008099172797887150000

unusual a novel 0.00000675944998630401000

unusual a particular 0.00014120049270362700000

unusual a startling 0.00014927615414568200000

unusual a new 0.01501943971297560000000

unusual a recent 0.01477195144612880000000

unusual a uncommon 0.00420513486492440000000

unusual a awesome 0.00003659856510203870000

unusual a inconceivable 0.00012196646105150800000

unusual a fabulous 0.00001372446191326450000

unusual a forward 0.00000813170819724793000

unusual a exceptional 0.13166024986289400000000

unusual a unbalanced 0.00000089647273476011300

unusual a unfamiliar 0.00001477240757445320000

unusual a deep 0.00003537876830448780000

unusual a weird 0.00001040850404284950000

unusual a puzzling 0.00001328499029341570000

unusual a noteworthy 0.00000022672713907417000

unusual a radical 0.00002118926807863850000

unusual a peculiar 0.00006088402441224920000

unusual a modern 0.00001721171241278090000

unusual a astounding 0.00005241436524653890000

unusual a unconventional 0.00000457482063775483000

unusual a late 0.00091674403471103900000

unusual a fine 0.00013243681937574100000

unusual a advanced 0.00014110551399696800000

unusual a prominent 0.00000288409496619200000

unusual a rare 0.02064026664924750000000

unusual a queer 0.00000457482063775483000

unusual a atypical 0.00000011336356953708500

unusual a infrequent 0.00000277073139665492000

unusual a unexpected 0.00144241268106246000000

unusual a unnatural 0.00014564195656994600000

unusual a unprecedented 0.00993790144532247000000

unusual a surprising 0.02519727446664210000000

unusual a extreme 0.00035969755503794600000

unusual a striking 0.00036224721340739100000

unusual a unique 0.00393372149210684000000

unusual a outstanding 0.00037905646466582400000

unusual a special 0.10342960049668900000000

unusual a perplexing 0.00001320058975986230000

unusual a outlandish 0.00000738620378722659000

unusual a superior 0.00000540086314413988000

unusual a abnormal 0.03441033588705030000000

unusual a important 0.00850393182102652000000

unusual a remarkable 0.00061818644540698000000

unusual a scarce 0.00372227082161745000000

unusual a marked 0.00000218462934854919000

unusual a notable 0.00000687281355584110000

unusual a fresh 0.00805789353810839000000

unusual a stupendous 0.00000022672713907417000

unusual a significant 0.00342649492490911000000

unusual a foreign 0.00015395883436751000000

unusual a wonderful 0.00017696089123710700000

unusual a irrational 0.00001618963203971190000

except v omit 1.00000000000000000000000

ensemble n company 0.97761194029850700000000

ensemble n body 0.02238805970149250000000

dread v expect 1.00000000000000000000000

cost-efficient a cost-effective 1.00000000000000000000000

colonisation n colonization 0.72413793103448300000000

colonisation n settlement 0.27586206896551700000000

hurricane n typhoon 0.09548714541447340000000

hurricane n violence 0.01510059784181920000000

hurricane n whirlwind 0.00023516823411115100000

hurricane n tornado 0.13101831487670500000000

hurricane n uproar 0.00000871275288973524000

hurricane n blizzard 0.00103564472329718000000

hurricane n gale 0.27045440708088800000000

hurricane n storm 0.38255442742260500000000

hurricane n wind 0.00498298851093817000000

hurricane n explosion 0.00224384129180587000000

hurricane n blast 0.00218892040725123000000

hurricane n blow 0.00005633213976093470000

hurricane n squall 0.00094067293644460400000

hurricane n cyclone 0.08667117190708220000000

hurricane n monsoon 0.00702165445992853000000

caption n banner 1.00000000000000000000000

appeasement n concession 0.16666666666666700000000

appeasement n adjustment 0.05555555555555470000000

appeasement n compromise 0.72222222222222400000000

appeasement n submission 0.05555555555555470000000

amuse v interest 0.02272727272727240000000

amuse v please 0.13636363636363600000000

amuse v absorb 0.06818181818181820000000

amuse v relax 0.47727272727272800000000

amuse v kill 0.15909090909090900000000

amuse v cheer 0.06818181818181820000000

amuse v divert 0.06818181818181820000000

alibi n account 1.00000000000000000000000

adept a able 1.00000000000000000000000

depress v bother 0.00005030719791018150000

depress v trouble 0.00701391411174549000000

depress v inhibit 0.00349006185501885000000

depress v slow 0.03641179340161850000000

depress v lower 0.14424255930861200000000

depress v humiliate 0.00001848917859365010000

depress v impair 0.00031871161602726000000

depress v weaken 0.17128218945478000000000

depress v sadden 0.01542000245534500000000

depress v press 0.00110585965831828000000

depress v daunt 0.00000289766953870068000

depress v dip 0.00220987278979891000000

depress v demoralise 0.00001559150905494940000

depress v demote 0.00001682729239005040000

depress v restrict 0.00626026158421290000000

depress v sink 0.00006879637650383160000

depress v slash 0.00144351078204550000000

depress v crush 0.00012136029861663800000

depress v try 0.00406060452090874000000

depress v disgrace 0.00000289766953870068000

depress v cut 0.04695231099332250000000

depress v tap 0.00060972606352371900000

depress v downgrade 0.00022865437408590800000

depress v degrade 0.00003118301810989890000

depress v dampen 0.15612885087085500000000

depress v humble 0.00018824603009617100000

depress v drain 0.01630648219531140000000

depress v discount 0.00225871552668963000000

depress v kill 0.02614701228878360000000

depress v reduce 0.04723554392108230000000

depress v cow 0.01861366322676720000000

depress v discourage 0.05584388734984020000000

depress v diminish 0.00007134139260984530000

depress v push 0.22964296116634700000000

depress v break 0.00092295391766078500000

depress v level 0.00000579533907740137000

depress v devalue 0.00039584797470785100000

depress v ail 0.00126911340182503000000

depress v flatten 0.00070530005545444300000

depress v beat 0.00193852001670880000000

depress v exhaust 0.00027254028055408400000

depress v depreciate 0.00020086686935360300000

depress v tire 0.00007359318178005510000

depress v settle 0.00040038181487426900000

not r no 1.00000000000000000000000

pct n percent 0.87217002821395700000000

pct n percentage 0.12782997178604300000000

expense n investment 0.02297573392139480000000

expense n price 0.00353010174415478000000

expense n capital 0.00879421007542687000000

expense n debit 0.00023896709647987900000

expense n drain 0.00000000886485332608243

expense n nut 0.00000048845481471880300

expense n fee 0.01632165634330030000000

expense n disbursement 0.00000034070562350227100

expense n obligation 0.00115317250335103000000

expense n consumption 0.00013313561134296700000

expense n depletion 0.00284355012965412000000

expense n mortgage 0.00205020944378975000000

expense n duty 0.00026502426486693200000

expense n cost 0.22912167221781200000000

expense n enterprise 0.00021689295872768700000

expense n responsibility 0.00014789587023657100000

expense n tariff 0.00026212058929650900000

expense n rate 0.09915714520074620000000

expense n expenditure 0.01196904067696900000000

expense n liability 0.02574280537671970000000

expense n worth 0.00010223245294658900000

expense n figure 0.00057237358527245100000

expense n compensation 0.00288609020542375000000

expense n bill 0.00138261889647744000000

expense n custom 0.00000002938850757575300

expense n debt 0.01461657700013520000000

expense n payroll 0.00090802499079860600000

expense n assessment 0.00074371412390234800000

expense n value 0.00095607218429430500000

expense n amount 0.00110947577335135000000

expense n charge 0.07296569085792400000000

expense n sum 0.00012868994028006600000

expense n loan 0.01291441821367570000000

expense n disbursal 0.00000155445307765501000

expense n fare 0.00010163431224475600000

expense n surcharge 0.00039590427912669200000

expense n allowance 0.00106612840554179000000

expense n account 0.00094526566965297200000

expense n insurance 0.00221495868850526000000

expense n interest 0.14952045527092700000000

expense n time 0.00170442412883700000000

expense n payment 0.00736478988316561000000

expense n settlement 0.00575751265026047000000

expense n excise 0.00000827251415639474000

expense n loss 0.22971189755214300000000

expense n risk 0.00127852969801580000000

expense n spending 0.00600903237080158000000

expense n lien 0.00000016076259151689000

expense n tax 0.05740409532912440000000

expense n quotation 0.00000002497703440663570

expense n overhead 0.00209039480195958000000

expense n outlay 0.00021478459028366600000

gunman n gangster 0.01983933834733360000000

gunman n hood 0.00001881786515985500000

gunman n bandit 0.02664883185327270000000

gunman n liquidator 0.00041142638561168800000

gunman n assassin 0.20019639773943600000000

gunman n sniper 0.04768549873775030000000

gunman n mobster 0.00072481768434023300000

gunman n thug 0.00001605196164796950000

gunman n bravo 0.00001413785901088180000

gunman n gun 0.02319894395792530000000

gunman n hooligan 0.00072032442755995700000

gunman n hitman 0.00091287346300585300000

gunman n shooter 0.05768794106951390000000

gunman n killer 0.43712751985072900000000

gunman n terrorist 0.18351881160799300000000

gunman n murderer 0.00127826718970992000000

viper n devil 0.17239701194734400000000

viper n turncoat 0.82760298805265600000000

vial n vessel 1.00000000000000000000000

usable a ready 0.64456394754321600000000

usable a good 0.12209373395689500000000

usable a profitable 0.00543711561060773000000

usable a operational 0.11537916694810800000000

usable a applicable 0.00543711561060773000000

usable a available 0.08579355085568610000000

usable a useful 0.00589020857815838000000

usable a valuable 0.00090618593510128400000

usable a suitable 0.00045309296755064200000

usable a practical 0.00045309296755064200000

usable a beneficial 0.00453092967550644000000

usable a desirable 0.00589020857815838000000

usable a helpful 0.00317165077285451000000

sheepherder n shepherd 1.00000000000000000000000

uncertain a unresolved 0.00676325255393577000000

uncertain a erratic 0.00112997840730298000000

uncertain a hazardous 0.00050668586257760800000

uncertain a irregular 0.00039556966579654400000

uncertain a poor 0.02167406463584760000000

uncertain a dubious 0.00054243776047287100000

uncertain a unpredictable 0.01860417806275360000000

uncertain a notional 0.00058730076605551200000

uncertain a casual 0.00002455280442357810000

uncertain a hesitant 0.00441953693552947000000

uncertain a contingent 0.00000050503672809081000

uncertain a mysterious 0.00000050503672809081000

uncertain a inconsistent 0.00048356984379882400000

uncertain a unlikely 0.06119204241655570000000

uncertain a moot 0.00000050503672809081000

uncertain a tricky 0.00055636033813473400000

uncertain a suspect 0.00046504514170308200000

uncertain a arbitrary 0.00000050503672809081000

uncertain a skeptical 0.01637231329379110000000

uncertain a problematic 0.00168139206227277000000

uncertain a precarious 0.00434925839386884000000

uncertain a unsettled 0.00182095066746274000000

uncertain a shadowy 0.00001296134698793340000

uncertain a unsafe 0.00033248342451370700000

uncertain a unreliable 0.00058591345034383100000

uncertain a speculative 0.00344139534487723000000

uncertain a restrained 0.00062298029308117500000

uncertain a feeble 0.00105728293253290000000

uncertain a treacherous 0.00037250099945818900000

uncertain a ambiguous 0.00040415991764534400000

uncertain a obscure 0.00003894662257479340000

uncertain a reserved 0.00009016964736137590000

uncertain a volatile 0.05691948052341940000000

uncertain a sticky 0.00006636560237549290000

uncertain a unconvinced 0.00424849349632463000000

uncertain a provisional 0.00042062280196107200000

uncertain a slippery 0.00019075513340288800000

uncertain a undetermined 0.00023701463212096600000

uncertain a improbable 0.00025853886583781700000

uncertain a baffling 0.00002588110676627980000

uncertain a skittish 0.00050023827639170500000

uncertain a hypothetical 0.00010064485867910500000

uncertain a indecisive 0.00012050490950528600000

uncertain a puzzling 0.00028507161627249700000

uncertain a fickle 0.00000597590626425436000

uncertain a unsteady 0.01442179643321220000000

uncertain a dangerous 0.00525275166582459000000

uncertain a blurred 0.00001183317106428930000

uncertain a abstract 0.00001227640221178910000

uncertain a unclear 0.45274412029281400000000

uncertain a ambivalent 0.00010178623006999500000

uncertain a unproven 0.00051590315430511700000

uncertain a hazy 0.00117568192271523000000

uncertain a debatable 0.00040638228788297100000

uncertain a possible 0.03568208225647430000000

uncertain a impulsive 0.00004548653162870600000

uncertain a transient 0.00000134902635892917000

uncertain a insecure 0.00010587500849878500000

uncertain a unknown 0.04699685275315950000000

uncertain a foggy 0.00007900487737365530000

uncertain a unsealed 0.00000134902635892917000

uncertain a variable 0.00198924618841159000000

uncertain a tentative 0.00053231832598246900000

uncertain a desultory 0.00001459372891334090000

uncertain a ill-defined 0.00011816418135419400000

uncertain a inadequate 0.00219811587056066000000

uncertain a changeable 0.00001227640221178910000

uncertain a imprecise 0.00065516761133726500000

uncertain a risky 0.00314250224764219000000

uncertain a perplexing 0.00005639616791347280000

uncertain a fortuitous 0.00007789324514958680000

uncertain a arguable 0.00035241021591949000000

uncertain a conditional 0.00012150721716570100000

uncertain a doubtful 0.00459959241567451000000

uncertain a shaky 0.00490990023129865000000

uncertain a sporadic 0.00009626101800467250000

uncertain a borderline 0.00000453076855017255000

uncertain a blurry 0.00024551062159249700000

uncertain a open 0.02777154749513960000000

uncertain a undecided 0.01323442189678380000000

uncertain a unfocused 0.00000453076855017255000

uncertain a fitful 0.00000134902635892917000

uncertain a delicate 0.00117762979256117000000

uncertain a suspicious 0.00022665473135432200000

uncertain a problematical 0.00049810758685695500000

uncertain a unsure 0.15545846788107900000000

uncertain a vague 0.00438822359020816000000

uncertain a unconfirmed 0.00023968067220287000000

uncertain a unstable 0.00318800619628651000000

uncertain a questionable 0.00515542023729969000000

uncertain a dim 0.00001459372891334090000

uncertain a indefinite 0.00005155743087676510000

totter v roll 1.00000000000000000000000

injection n investment 0.45425173119432200000000

injection n addition 0.11221735073379900000000

injection n infusion 0.17235213478540300000000

injection n fixing 0.00023595605597353600000

injection n shot 0.00079419335371511200000

injection n hit 0.00085368092731794400000

injection n introduction 0.00002497054401246430000

injection n inoculation 0.17012431635692000000000

injection n bang 0.00070649633038587800000

injection n vaccine 0.00008136103519973090000

injection n dose 0.08835780868295240000000

spotlight v mark 0.12499999999999900000000

spotlight v stress 0.37500000000000100000000

spotlight v intensify 0.37500000000000100000000

spotlight v heighten 0.12499999999999900000000

gang n company 0.07373537167682220000000

gang n organization 0.07958775732358220000000

gang n brotherhood 0.02579660018256040000000

gang n clan 0.00004942571870838590000

gang n set 0.00001135929340818270000

gang n society 0.00097363466673751300000

gang n unit 0.03981948143394680000000

gang n band 0.03035448718563210000000

gang n crew 0.06039308607046810000000

gang n crowd 0.00241738111819326000000

gang n machine 0.00002476748876443050000

gang n combination 0.00001543029012242750000

gang n group 0.52699159842742500000000

gang n load 0.00000870736321700597000

gang n shift 0.00005813947132112890000

gang n squad 0.00469633181039225000000

gang n collection 0.00000909924272657123000

gang n gathering 0.00001772257362227920000

gang n circle 0.00000909924272657123000

gang n team 0.01857941232969570000000

gang n ring 0.04742497283959940000000

gang n association 0.00080687448012785100000

gang n tribe 0.00001780660594357730000

gang n sect 0.00022273818780647800000

gang n pack 0.00000443064340556981000

gang n mob 0.00202890128480166000000

gang n family 0.05347813289239590000000

gang n party 0.01935459885890450000000

gang n herd 0.00045579414509052100000

gang n body 0.00529998488347227000000

gang n club 0.00729840179644910000000

gang n junta 0.00005847047193008660000

seventy n 70 1.00000000000000000000000

shaping n formation 1.00000000000000000000000

comply v fulfill 0.05049388905617160000000

comply v follow 0.02702524936466240000000

comply v yield 0.00517222297385385000000

comply v mind 0.00062498368352523100000

comply v obey 0.05340008644982560000000

comply v grant 0.00057332599285564100000

comply v adjust 0.00135551359491773000000

comply v submit 0.00029781209781740100000

comply v concur 0.00203500661036093000000

comply v meet 0.51282805868949800000000

comply v perform 0.00055411625856915600000

comply v consent 0.00007111124624266580000

comply v acknowledge 0.00039860710458267300000

comply v agree 0.09402516696426360000000

comply v keep 0.01735798459687720000000

comply v satisfy 0.01071156534842740000000

comply v discharge 0.00006174419966925020000

comply v respect 0.06582638214193650000000

comply v execute 0.00196995148430464000000

comply v accord 0.01091518741707060000000

comply v fit 0.00054015705005051600000

comply v surrender 0.02329097481432110000000

comply v coincide 0.00787671983893396000000

comply v approve 0.00594790489513797000000

comply v observe 0.01541554389574360000000

comply v defer 0.00004940119164753720000

comply v adapt 0.00023451657778017700000

comply v complete 0.02832642231254220000000

comply v bow 0.00762668115952591000000

comply v accept 0.03899570419608540000000

comply v accommodate 0.00004770453910127520000

comply v suit 0.00060631421161342300000

comply v conform 0.01534399004208450000000

reprehensible a unlawful 0.15304163257370200000000

reprehensible a illegal 0.83923782982412100000000

reprehensible a guilty 0.00772053760217758000000

publicised a publicized 1.00000000000000000000000

milk n juice 0.20356693974095900000000

milk n sap 0.00885828833343764000000

milk n latex 0.78757477192560300000000

pedigree n stock 0.46680488773214500000000

pedigree n blood 0.47278956577999300000000

pedigree n line 0.06040554648786150000000

luckily r fortunately 1.00000000000000000000000

lastly r finally 0.71305359553700700000000

lastly r ultimately 0.28694640446299300000000

column n company 0.07758838666659120000000

column n spar 0.00000349581032200443000

column n mast 0.00000205154295063336000

column n row 0.00002547551551321600000

column n article 0.85433879246023800000000

column n feature 0.00002688873112701920000

column n train 0.00001641234360506700000

column n editorial 0.01359305279007090000000

column n support 0.00135178693405993000000

column n piece 0.00002137242961194920000

column n line 0.03055718243913620000000

column n platoon 0.00000205154295063336000

column n file 0.00000615462885190008000

column n comment 0.00552585111671443000000

column n commentary 0.01607781838795260000000

column n standard 0.00047044798593211300000

column n list 0.00007018980585910540000

column n atlas 0.00001747905161002230000

column n convoy 0.00000410308590126672000

column n pole 0.00000205154295063336000

column n pillar 0.00010821701880015300000

column n monument 0.00000205154295063336000

column n post 0.00018868662630054800000

inventive a sharp 0.01668542673641000000000

inventive a talented 0.01668542673641000000000

inventive a clever 0.41657286631795000000000

inventive a fruitful 0.00417135668410248000000

inventive a new 0.01668542673641000000000

inventive a quick 0.02502814010461500000000

inventive a unusual 0.02085678342051240000000

inventive a constructive 0.04588492352512750000000

inventive a creative 0.01251407005230750000000

inventive a able 0.39154472621333500000000

inventive a productive 0.02085678342051240000000

inventive a fresh 0.00417135668410248000000

inventive a skilled 0.00834271336820495000000

prevail v lead 0.01458055745026990000000

prevail v remain 0.14180917957461300000000

prevail v prompt 0.03103800939863140000000

prevail v prove 0.00003875933759223350000

prevail v bear 0.00134154280894231000000

prevail v occur 0.00233818472033840000000

prevail v dominate 0.03147924929542020000000

prevail v win 0.25503225890358400000000

prevail v obtain 0.00008750276364374000000

prevail v press 0.00005495090953732050000

prevail v succeed 0.02147653741515090000000

prevail v last 0.07277210095249120000000

prevail v convert 0.00110487097197497000000

prevail v convince 0.00056803484869248500000

prevail v move 0.02948562871377100000000

prevail v influence 0.00007941252645308070000

prevail v overcome 0.00163813427562629000000

prevail v govern 0.02270821452810330000000

prevail v run 0.00545007286445788000000

prevail v carry 0.00520408706823560000000

prevail v persist 0.12963056640924400000000

prevail v gain 0.00430240938862563000000

prevail v inspire 0.00000429215445734185000

prevail v command 0.00001363179475731410000

prevail v hold 0.08061326719864550000000

prevail v stay 0.05509573455477120000000

prevail v rule 0.00973182242534207000000

prevail v exist 0.03115511628979060000000

prevail v sway 0.00005138357248517260000

prevail v urge 0.00202260625211477000000

prevail v sell 0.01693953310338520000000

prevail v control 0.00194201838085145000000

prevail v reign 0.03021032914800120000000

incidental a accidental 1.00000000000000000000000

advanced a old 0.00039032248750430400000

advanced a higher 0.00130515272726497000000

advanced a advance 0.02257919612011110000000

advanced a original 0.00022730734349105200000

advanced a new 0.06783877626701650000000

advanced a early 0.52031714807872000000000

advanced a exceptional 0.00021733985570160500000

advanced a polished 0.00010901972375590100000

advanced a first 0.07940566428847380000000

advanced a radical 0.00021768965160670400000

advanced a modern 0.00000572736859392349000

advanced a late 0.10432671448211200000000

advanced a improved 0.00006773758836033350000

advanced a refined 0.00021519149234576600000

advanced a extreme 0.03719000462410270000000

advanced a developed 0.05120236694266430000000

advanced a high-level 0.00000572736859392349000

advanced a sophisticated 0.11397484893397400000000

advanced a innovative 0.00040406465560728700000

grandiose a grand 0.00406091370558375000000

grandiose a ambitious 0.97563451776649800000000

grandiose a impressive 0.01421319796954310000000

grandiose a affected 0.00609137055837563000000

landing n touchdown 0.43784775001756300000000

landing n marina 0.00001721428865748060000

landing n china 0.02441783269056220000000

landing n docking 0.10352390566520800000000

landing n airfield 0.00079380439572721900000

landing n arrival 0.00003425380936562900000

landing n grounding 0.00183803517820172000000

landing n japan 0.00691659957140852000000

landing n bund 0.00101570639256373000000

landing n platform 0.00010986704378193100000

landing n harbor 0.00002357144000448580000

landing n port 0.16717128323977600000000

landing n runway 0.20859684071306000000000

landing n airport 0.02254231154554300000000

landing n lighting 0.00002136473872228630000

landing n slip 0.00014802967878134600000

landing n pier 0.00003050228774019730000

landing n wharf 0.00001068236936114320000

landing n stage 0.02494044493397240000000

move n walk 0.00002494823266327780000

move n hurry 0.00153635571621330000000

move n progress 0.02147269936958730000000

move n relocation 0.00430193809653427000000

move n run 0.02085761490639390000000

move n posting 0.00031178874824401900000

move n attempt 0.01602477782995520000000

move n drift 0.00198898076534815000000

move n fly 0.00000245958923118707000

move n advance 0.02299877447398160000000

move n proceeding 0.00005708339883619940000

move n ploy 0.00042329006473654500000

move n deed 0.00033164403838525400000

move n feat 0.00022872355466499600000

move n recourse 0.00001771431960276310000

move n excite 0.00022988181666246000000

move n stab 0.00006846844663437630000

move n stunt 0.00011408184785025100000

move n endeavor 0.00023460038399128300000

move n shot 0.00255240539202567000000

move n transfer 0.01186221809413000000000

move n initiative 0.02290650277858300000000

move n lure 0.00016663757112700500000

move n rouse 0.00000213559126383036000

move n gimmick 0.00013681261047385900000

move n influence 0.00372585608706856000000

move n commence 0.00001821099032764970000

move n turn 0.01524296795883580000000

move n urge 0.00002481981190496100000

move n travel 0.00061692801516210500000

move n device 0.00642788028192021000000

move n means 0.00051866353331275500000

move n motion 0.00782824697883427000000

move n coup 0.00369534335278610000000

move n go 0.00092064286011493300000

move n shift 0.01656907092631200000000

move n design 0.00128375043619085000000

move n resort 0.00025695224750435700000

move n essay 0.00023620703085069800000

move n exploit 0.00000417252803174506000

move n spur 0.00013954694167699800000

move n ruse 0.00000305100056600689000

move n working 0.00046822915303958800000

move n proceed 0.00001405066050134780000

move n activity 0.03999006338564450000000

move n transit 0.00020858110468426000000

move n action 0.18617400951443600000000

move n tactic 0.00134165806246813000000

move n bout 0.00006210802005251310000

move n bias 0.00187696751433619000000

move n measure 0.15187016142368500000000

move n try 0.00042237465325168200000

move n rush 0.00382675174968969000000

move n effort 0.08455717976089900000000

move n stroke 0.00015132151940735400000

move n stopgap 0.00000802587798039431000

move n gesture 0.00267793995413870000000

move n crack 0.00099441172883914900000

move n craft 0.00057267299608398000000

move n start 0.00827452209444418000000

move n active 0.00004742583917338030000

move n maneuver 0.00955991796832930000000

move n movement 0.13602710559741100000000

move n undertaking 0.00195586443011788000000

move n step 0.18228974436749300000000

move n busy 0.00000080136179251540600

move n trick 0.00026326264358110600000

etch v carve 1.00000000000000000000000

accusation n claim 0.06454577008698820000000

accusation n assertion 0.00530027948431282000000

accusation n assignment 0.00000024979174237596600

accusation n allegation 0.27694184432039800000000

accusation n beef 0.00004456898964951650000

accusation n complaint 0.01402577950183050000000

accusation n criticism 0.02370590317260900000000

accusation n arraignment 0.00000074937522712789900

accusation n recrimination 0.00262567454581490000000

accusation n charge 0.61131556268001100000000

accusation n prosecution 0.00001016863696433340000

accusation n count 0.00000352396534943433000

accusation n implication 0.00001769872254096290000

accusation n indictment 0.00146222672656246000000

embittered a bitter 1.00000000000000000000000

digger n excavator 1.00000000000000000000000

successfully r strongly 1.00000000000000000000000

debatable a dubious 0.02715879551272630000000

debatable a problematic 0.04600137568715820000000

debatable a unsettled 0.00071188647337609700000

debatable a controversial 0.37606018973526800000000

debatable a uncertain 0.24205370781499000000000

debatable a floating 0.00705970551499389000000

debatable a doubtful 0.09036500555541820000000

debatable a undecided 0.00096120004344590400000

debatable a unsure 0.00071188647337609700000

debatable a questionable 0.20891624718924800000000

daybreak n dawn 1.00000000000000000000000

conflicting a contradictory 1.00000000000000000000000

cloak n cover 1.00000000000000000000000

justify v back 0.07587739637943580000000

justify v deliver 0.13455468987461100000000

justify v forgive 0.00196672432272831000000

justify v verify 0.00215417182993048000000

justify v rationalise 0.00055984997524991900000

justify v vouch 0.00011319496220542300000

justify v liberate 0.00030576145396366000000

justify v defend 0.07777002558222290000000

justify v clear 0.00509479769720204000000

justify v dismiss 0.00382876147938520000000

justify v excuse 0.00050284212891460000000

justify v vindicate 0.00049692616031054500000

justify v condone 0.00064426667858916000000

justify v pardon 0.00065177123823553900000

justify v explain 0.09068624187385080000000

justify v redress 0.00023178195041226400000

justify v favor 0.00007439520051947980000

justify v substantiate 0.00186393193244995000000

justify v exonerate 0.00017245860651016000000

justify v free 0.00351230403720746000000

justify v sustain 0.02735340982819030000000

justify v countenance 0.00004635639008245190000

justify v discharge 0.00061481136761236900000

justify v authorize 0.00002171556389860340000

justify v release 0.02195503718053350000000

justify v uphold 0.00073948132290946500000

justify v rationalize 0.00002171556389860340000

justify v support 0.41986959142249500000000

justify v redeem 0.00027567725900952500000

justify v brief 0.00008461341074521310000

justify v apologise 0.00005664684119203560000

justify v confirm 0.06292840483856010000000

justify v advocate 0.00092336285649678600000

justify v warrant 0.04260240939982780000000

justify v maintain 0.02114751623600540000000

justify v assert 0.00006807195398105530000

justify v whitewash 0.00002171556389860340000

justify v acquit 0.00020716963672833900000

burglar n thief 0.99554463051159900000000

burglar n gangster 0.00445536948840066000000

bulge n growth 0.20000000000000000000000

bulge n rise 0.60000000000000000000000

bulge n surge 0.20000000000000000000000

bravery n enterprise 1.00000000000000000000000

boot v kick 1.00000000000000000000000

billet v live 0.89308176100628900000000

billet v remain 0.07662473794549270000000

billet v locate 0.01069182389937100000000

billet v stay 0.00712788259958069000000

billet v settle 0.01247379454926620000000

seal v establish 0.00274797694632917000000

seal v ensure 0.00002145089802318060000

seal v cinch 0.00000121646102071743000

seal v promise 0.00195525898095358000000

seal v secure 0.02071410623968620000000

seal v validate 0.00002480489069724100000

seal v consummate 0.00149985171886532000000

seal v shut 0.00962497943250613000000

seal v conclude 0.05822244011753830000000

seal v guarantee 0.00268180704950717000000

seal v pledge 0.00019328133118200700000

seal v vow 0.00000464388250770294000

seal v enclose 0.00000060823051035871300

seal v engage 0.00000604916504672057000

seal v finalize 0.03823386319959540000000

seal v plaster 0.00000604916504672057000

seal v assure 0.00186122674710784000000

seal v bind 0.00003022547126629190000

seal v authorize 0.00000947658653370608000

seal v ratify 0.01900469903330040000000

seal v authenticate 0.00000726562606743799000

seal v decide 0.01735133662584590000000

seal v sign 0.26931428904544600000000

seal v approve 0.01711750473110210000000

seal v clinch 0.02782963683697760000000

seal v confirm 0.00418477868396576000000

seal v complete 0.19268404619127900000000

seal v insure 0.00003853909080345850000

seal v certify 0.00018698404085151900000

seal v warrant 0.00001952437303862080000

seal v stamp 0.00000665739555707928000

seal v plug 0.00000604916504672057000

seal v close 0.31137315150507100000000

seal v sanction 0.00005028419353410060000

seal v stabilize 0.00009431509677617570000

seal v settle 0.00289162185141368000000

bald a clear 1.00000000000000000000000

legislative a parliamentary 0.81485466994708500000000

legislative a congressional 0.18436369971415600000000

legislative a judicial 0.00078163033875840300000

anemic a ineffective 0.00002377737184390940000

anemic a anaemic 0.86227023732220900000000

anemic a ineffectual 0.00001188868592195470000

anemic a weak 0.13769409662002500000000

acknowledgment n acknowledgement 0.97489539748954000000000

acknowledgment n admission 0.00209205020920502000000

acknowledgment n statement 0.02301255230125520000000

accredited a qualified 1.00000000000000000000000

job n graft 0.00000360019208139607000

job n employment 0.26723679266270700000000

job n profession 0.00023826248782553200000

job n assignment 0.00214049128124128000000

job n calling 0.00000327347856157342000

job n matter 0.00586211020206761000000

job n act 0.00165180300818106000000

job n deal 0.05548509200325660000000

job n proceeding 0.00002742914217983240000

job n mission 0.00261402288786236000000

job n deed 0.00089152427685502000000

job n contribution 0.00168891224353744000000

job n care 0.00211269482974146000000

job n affair 0.00131999707819382000000

job n obligation 0.00159873577318747000000

job n grind 0.00061652694262927600000

job n duty 0.00579050189618579000000

job n part 0.01369140595749710000000

job n livelihood 0.00239406001831092000000

job n faculty 0.00000349161742902620000

job n lookout 0.00000015324666247628300

job n enterprise 0.00485157142392127000000

job n accomplishment 0.00005852931705037430000

job n responsibility 0.00296864510790057000000

job n caper 0.00003343250458124920000

job n corruption 0.00122772723658689000000

job n billet 0.00000772653819102416000

job n operation 0.01382260768878540000000

job n commission 0.00707900023740714000000

job n task 0.01123166123329820000000

job n area 0.01240572381721520000000

job n career 0.00268789996892283000000

job n berth 0.00082917011766001100000

job n appointment 0.00151005490066512000000

job n problem 0.03432144069502060000000

job n dealings 0.00009005666484375300000

job n input 0.00022650342468459800000

job n exercise 0.00094213915480333400000

job n office 0.02139357060390080000000

job n burden 0.00015894111496888900000

job n line 0.00936606909227408000000

job n project 0.02691451886327350000000

job n charge 0.00745951260079757000000

job n position 0.06385096005439000000000

job n living 0.00127866884067203000000

job n function 0.00263039217913194000000

job n vocation 0.00001904695120378650000

job n department 0.00407907207820968000000

job n occupation 0.00098450215082421300000

job n chore 0.00064019887868485900000

job n activity 0.01347480534912690000000

job n place 0.01143036197182630000000

job n action 0.01307897375006010000000

job n trade 0.04255438054554030000000

job n concern 0.00621069252307924000000

job n output 0.00900448457375073000000

job n achievement 0.00046373153030693400000

job n effort 0.00346896822863876000000

job n work 0.07495386782624880000000

job n craft 0.00118860431973198000000

job n capacity 0.00918728696740960000000

job n situation 0.00720630876670584000000

job n venture 0.00607327562331416000000

job n labor 0.04085641211313010000000

job n performance 0.00161325681152742000000

job n business 0.05451584742824880000000

job n gig 0.00000456353579510592000

job n trust 0.00173142099371394000000

job n pursuit 0.00006957262384340840000

job n undertaking 0.00126817209136104000000

job n transaction 0.00790453094745195000000

job n throughput 0.00043900254431665800000

job n province 0.00121992396362368000000

job n station 0.00298750383402736000000

job n field 0.00381392685886212000000

job n role 0.01149307511512350000000

job n post 0.07534682449717270000000

steep a sharp 0.70205683264099100000000

steep a unreasonable 0.00004051233915102120000

steep a lofty 0.00017206116730666400000

steep a high 0.21808642436183400000000

steep a dramatic 0.05271799250813040000000

steep a precipitous 0.01671738458307450000000

steep a exorbitant 0.00002984918573296940000

steep a excessive 0.00052277500849022100000

steep a sudden 0.00848013381784425000000

steep a extreme 0.00065643861773774700000

steep a stiff 0.00017473641351254400000

steep a overpriced 0.00000535049241176079000

steep a bold 0.00019919783550369200000

steep a precipitate 0.00014031102827914700000

herald n adviser 0.00006041669403736590000

herald n indication 0.00017748004833154900000

herald n scout 0.00002963520051445210000

herald n signal 0.00002963520051445210000

herald n envoy 0.00198208423287404000000

herald n announcer 0.00005927040102890420000

herald n prediction 0.00003283654776614560000

herald n pioneer 0.00117437752338555000000

herald n reporter 0.99550265701172500000000

herald n broadcaster 0.00026255967352342200000

herald n intuition 0.00002963520051445210000

herald n sign 0.00053883467147968900000

herald n runner 0.00008979610078169420000

herald n symptom 0.00003078149352291380000

vehement a fierce 1.00000000000000000000000

unruffled a flat 0.10398084657988300000000

unruffled a quiet 0.31641730595390400000000

unruffled a regular 0.01390035215668340000000

unruffled a peaceful 0.07685244688063050000000

unruffled a balanced 0.02780070431336680000000

unruffled a calm 0.06661297251398350000000

unruffled a steady 0.37986271483075200000000

unruffled a controlled 0.01457265677079750000000

temporarily r briefly 0.88880812198979900000000

temporarily r tentatively 0.11119187801020100000000

forge v establish 0.38033444218285200000000

forge v falsify 0.00006011025096607620000

forge v invent 0.00000580704177227402000

forge v frame 0.00002613168797523330000

forge v duplicate 0.00006961704767411870000

forge v replicate 0.00002713128381933740000

forge v fake 0.00002236447944629460000

forge v work 0.00101576169130106000000

forge v cast 0.00000580704177227402000

forge v found 0.00132786199683840000000

forge v modify 0.00010078831984002600000

forge v build 0.09606458580905890000000

forge v alter 0.00007458721384163410000

forge v produce 0.00007340874032214490000

forge v create 0.01605027224668890000000

forge v construct 0.00000580704177227402000

forge v feign 0.00000290352088613701000

forge v formulate 0.00002577626054341850000

forge v trace 0.00003293832559161140000

forge v shape 0.01866490424903690000000

forge v beat 0.00024687598917803500000

forge v form 0.48565699604240400000000

forge v fabricate 0.00000871056265841103000

forge v drive 0.00009641097376065080000

summertime n summer 1.00000000000000000000000

contractor n manufacturer 0.18252193202573800000000

contractor n planner 0.00002505186562507700000

contractor n constructor 0.10003001178546500000000

contractor n worker 0.17380538832837300000000

contractor n maker 0.27522961969556500000000

contractor n builder 0.26282829524571200000000

contractor n entrepreneur 0.00018489835819864200000

contractor n mason 0.00015923932432906700000

contractor n architect 0.00521556337099336000000

shackle v fetter 1.00000000000000000000000

excess n rest 0.03848541101204440000000

excess n exuberance 0.00002744194274129340000

excess n surplus 0.31432851981349900000000

excess n overrun 0.00019146766059647700000

excess n waste 0.00109264255374075000000

excess n overvaluation 0.00043832049611891400000

excess n overcharge 0.00053743260017019400000

excess n remainder 0.01838142635257770000000

excess n balance 0.49886064733778900000000

excess n plenty 0.06890863084364020000000

excess n margin 0.05569928878103970000000

excess n oversupply 0.00135001082470189000000

excess n redundancy 0.00152318419084637000000

excess n residue 0.00017557559049384800000

recast v remodel 1.00000000000000000000000

pretender n liar 0.20000000000000000000000

pretender n sham 0.20000000000000000000000

pretender n bluff 0.20000000000000000000000

pretender n imposter 0.20000000000000000000000

pretender n hypocrite 0.20000000000000000000000

solidarity n union 0.66075037338507600000000

solidarity n stability 0.02697494687003400000000

solidarity n accord 0.00137382238125896000000

solidarity n concord 0.00452892516582190000000

solidarity n consensus 0.00112360526750489000000

solidarity n power 0.07521741755114980000000

solidarity n agreement 0.03722761671987030000000

solidarity n entirety 0.00037746616118529800000

solidarity n soundness 0.00007159274857147820000

solidarity n unity 0.18004572390639300000000

solidarity n integrity 0.00001792993807487680000

solidarity n strength 0.01225754319445240000000

solidarity n harmony 0.00003303671060657750000

disappear v melt 0.00001754858872830930000

disappear v vanish 0.34458066212828300000000

disappear v decamp 0.00000582496733123521000

disappear v fly 0.00162643851078532000000

disappear v evaporate 0.02303881979324710000000

disappear v dissipate 0.01161997487123160000000

disappear v vacate 0.00048626166333322200000

disappear v ebb 0.00793925356055011000000

disappear v withdraw 0.02492946792211800000000

disappear v clear 0.00089117154963101200000

disappear v perish 0.00082930388386424300000

disappear v flee 0.12502971747682000000000

disappear v expire 0.01318409952975870000000

disappear v retire 0.01667850217329500000000

disappear v leave 0.07621494460461050000000

disappear v quit 0.00076087682270343200000

disappear v sink 0.00454918544260584000000

disappear v escape 0.00520244404138057000000

disappear v retreat 0.02087873715060010000000

disappear v dissolve 0.00014114031552313700000

disappear v repair 0.00002068666342947450000

disappear v abscond 0.00026680428995906400000

disappear v cease 0.00561162465067726000000

disappear v end 0.03643297831323330000000

disappear v fade 0.01725831888547320000000

disappear v depart 0.02069210352666580000000

disappear v go 0.09154038905466900000000

disappear v wane 0.00289271710658898000000

disappear v disperse 0.00009073154987205120000

disappear v die 0.10892568726708500000000

disappear v pass 0.03658610167494670000000

disappear v recede 0.00107748202099972000000

screwy a crazy 1.00000000000000000000000

mystify v get 0.04772163865373350000000

mystify v puzzle 0.04772163865373350000000

mystify v fool 0.37596644430413900000000

mystify v amaze 0.14499104255986300000000

mystify v baffle 0.02646428699337110000000

mystify v stick 0.06897899031409590000000

mystify v lie 0.08685553324011220000000

mystify v astonish 0.00520693533300873000000

mystify v trick 0.04251470332072480000000

mystify v stump 0.00520693533300873000000

mystify v bewilder 0.02125735166036240000000

mystify v dupe 0.00520693533300873000000

mystify v beat 0.03167122232637990000000

mystify v confuse 0.06377205498108720000000

mystify v stun 0.02125735166036240000000

mystify v mislead 0.00520693533300873000000

prominent a primary 0.00002069742170192720000

prominent a great 0.00014610874719559400000

prominent a noticeable 0.00000412649549760941000

prominent a extraordinary 0.00295076730303862000000

prominent a distinctive 0.01767954049948430000000

prominent a relieved 0.00002092784028010310000

prominent a popular 0.08997663063270300000000

prominent a particular 0.01795667302265030000000

prominent a telling 0.00000523196007002576000

prominent a leading 0.04665864078797170000000

prominent a exceptional 0.00063398602228982000000

prominent a conspicuous 0.03093919587409750000000

prominent a first 0.00131849150024433000000

prominent a eminent 0.00515653264568291000000

prominent a identifiable 0.00000523196007002576000

prominent a noteworthy 0.00000214063936305655000

prominent a apparent 0.00149194786183822000000

prominent a principal 0.00237993814416134000000

prominent a peculiar 0.00000523196007002576000

prominent a main 0.01791052559659670000000

prominent a dominant 0.06654067570476070000000

prominent a chief 0.08812020569406500000000

prominent a top 0.22183755772443000000000

prominent a large 0.02129794135728330000000

prominent a obvious 0.00033055318581537700000

prominent a famous 0.12634194895979900000000

prominent a big 0.00243445517714421000000

prominent a spectacular 0.00003739781606855490000

prominent a glaring 0.01031306529136580000000

prominent a striking 0.00262111141325035000000

prominent a unique 0.00125653960461998000000

prominent a rough 0.00002615980035012880000

prominent a outstanding 0.01010856059333710000000

prominent a evident 0.03204124765943420000000

prominent a special 0.00003243917733851750000

prominent a important 0.06263106164279500000000

prominent a proud 0.00071306228415596200000

prominent a prime 0.00030184581340582900000

prominent a remarkable 0.00000995531638339014000

prominent a notable 0.03681057945617860000000

prominent a brilliant 0.00001111109600279460000

prominent a significant 0.00023211420687014000000

prominent a bold 0.00000523196007002576000

prominent a well-known 0.08066051499737240000000

prominent a respected 0.00002209715269695260000

mace n staff 0.11111111111111100000000

mace n cane 0.11111111111111100000000

mace n club 0.77777777777777900000000

inexcusable a outrageous 0.00396983322322157000000

inexcusable a intolerable 0.00793966644644314000000

inexcusable a immoral 0.00396983322322157000000

inexcusable a wrong 0.98015083388389200000000

inexcusable a unjustifiable 0.00396983322322157000000

immerse v draw 0.00147917700521976000000

immerse v soak 0.90303756168666400000000

immerse v saturate 0.00572448533557314000000

immerse v engage 0.00295835401043952000000

immerse v lose 0.00191611304571167000000

immerse v bury 0.01641886475793930000000

immerse v involve 0.00191611304571167000000

immerse v hold 0.06654933111274120000000

ill-advised a irresponsible 0.01143274234220500000000

ill-advised a unwise 0.00142909279277562000000

ill-advised a foolish 0.00285818558555124000000

ill-advised a spontaneous 0.08865756135660700000000

ill-advised a misguided 0.02243283430593350000000

ill-advised a wrong 0.78284637342425600000000

ill-advised a perverse 0.00142909279277562000000

ill-advised a foolhardy 0.00142909279277562000000

ill-advised a inappropriate 0.08748502460712000000000

issuance n issuing 0.00132997337151862000000

issuance n issue 0.99867002662848100000000

graze v range 1.00000000000000000000000

formation n evolution 0.00001011042321146260000

formation n organization 0.00401627078165169000000

formation n outline 0.00005561891332753450000

formation n accumulation 0.00000273765351379036000

formation n configuration 0.00019108965235103100000

formation n make-up 0.01820128938643570000000

formation n structure 0.04085682896099070000000

formation n origination 0.00006087387754660750000

formation n figure 0.00837156488608476000000

formation n constitution 0.00334906581837909000000

formation n production 0.00132963252586562000000

formation n generation 0.00435440028378088000000

formation n arrangement 0.00040723155174051900000

formation n development 0.01868095024993720000000

formation n creation 0.54209715929851400000000

formation n foundation 0.00049165050338441300000

formation n order 0.00950435372184454000000

formation n framework 0.00039207004680653200000

formation n pattern 0.20815487498886000000000

formation n origin 0.00002165943216082780000

formation n establishment 0.04584884422410580000000

formation n look 0.00030023302988483900000

formation n shape 0.03914785699500320000000

formation n shaping 0.00000505521160573127000

formation n grouping 0.00000505521160573127000

formation n construction 0.00186441413294153000000

formation n building 0.00681208740521451000000

formation n innovation 0.03640663748385060000000

formation n making 0.00011664000499934000000

formation n composition 0.00238073372516762000000

formation n deposit 0.00025942491325611000000

formation n array 0.00000505521160573127000

formation n inauguration 0.00238769904892421000000

formation n institution 0.00391083044544759000000

confederation n union 0.24139280128441500000000

confederation n assembly 0.00028551082143115200000

confederation n federation 0.53272937966760200000000

confederation n amalgamation 0.00000741455621158824000

confederation n interaction 0.00000359014175442026000

confederation n consolidation 0.00012294651461485200000

confederation n combine 0.00000105345103561457000

confederation n combination 0.00001640928897659830000

confederation n syndicate 0.00010602273678598700000

confederation n cartel 0.00020167661574652500000

confederation n conspiracy 0.00000210690207122914000

confederation n association 0.14861574984257800000000

confederation n plot 0.00002188123146075050000

confederation n coalition 0.00790846034716251000000

confederation n alliance 0.05994153629053730000000

confederation n league 0.00041016502682650800000

confederation n cooperation 0.00823329528078977000000

energetically r vigorously 1.00000000000000000000000

scandalize v scandalise 1.00000000000000000000000

street n walk 0.00000150302979792440000

street n circus 0.00001265964716714780000

street n interstate 0.02001162985285470000000

street n drag 0.00009021166856782880000

street n boulevard 0.00407437998628040000000

street n route 0.02492047009295060000000

street n via 0.00000612256274664715000

street n row 0.00252384395007265000000

street n alley 0.00001982716986408860000

street n pavement 0.00078375636168632400000

street n drive 0.00092646596805068500000

street n freeway 0.00434747010616535000000

street n section 0.00077418652467552600000

street n piazza 0.00000829673072409369000

street n precinct 0.00666192907433376000000

street n terrace 0.00078375636168632400000

street n roadway 0.00001760086941699800000

street n court 0.09212228402459010000000

street n pike 0.00000414836536204684000

street n way 0.03978211356154040000000

street n path 0.00069598296072619900000

street n thoroughfare 0.00249822340287518000000

street n highway 0.08291005226217490000000

street n square 0.04247028368420140000000

street n close 0.05413947008839720000000

street n strip 0.00011923006142931000000

street n passage 0.00242855051014387000000

street n place 0.15637311132844600000000

street n circle 0.00001112787312285530000

street n neighborhood 0.01769402429475190000000

street n lane 0.00003456108392268320000

street n avenue 0.00249822340287518000000

street n mall 0.02007968540704040000000

street n district 0.10484749105359700000000

street n quarter 0.02660151698945260000000

street n road 0.28622758628543500000000

street n artery 0.00249822340287518000000

conventional a normal 0.00002944585196428800000

conventional a predictable 0.00008833755589286410000

conventional a current 0.02772446072548830000000

conventional a moderate 0.00005889170392857600000

conventional a regular 0.50381940775624900000000

conventional a ordinary 0.04351886592807740000000

conventional a traditional 0.15017305628969100000000

conventional a general 0.14678545136042100000000

conventional a usual 0.00138395504232154000000

conventional a expected 0.00008667863465543930000

conventional a standard 0.08637835509966870000000

conventional a received 0.03987910623227460000000

conventional a routine 0.00002944585196428800000

conventional a correct 0.00004454196740305210000

dysfunction n disfunction 1.00000000000000000000000

boycott n abstention 0.00044763475765114000000

boycott n ban 0.18652760081100500000000

boycott n sanction 0.48292605096801300000000

boycott n restriction 0.00077893584539284700000

boycott n veto 0.05116049189275560000000

boycott n embargo 0.21073248487629300000000

boycott n restraint 0.00386581244989147000000

boycott n prevention 0.06319947306066900000000

boycott n exclusion 0.00036151533833008700000

joint n union 0.34332422698511300000000

joint n unit 0.24688501021901700000000

joint n member 0.10046576933830400000000

joint n connection 0.00194555930668835000000

joint n portion 0.00001879880965457660000

joint n segment 0.00018904639391486600000

joint n place 0.20819219494340500000000

joint n association 0.01988170670700140000000

joint n division 0.02328831033956820000000

joint n bond 0.04020874878348830000000

joint n club 0.01560062817384490000000

husband n spouse 0.58217747893802400000000

husband n overseer 0.00000861598573976718000

husband n provider 0.00101656323178197000000

husband n dad 0.00020015969648800800000

husband n groom 0.00000861598573976718000

husband n manager 0.00087984274964816500000

husband n lord 0.00001703368271289400000

husband n man 0.41533080424999600000000

husband n roommate 0.00035226949413000300000

husband n bridegroom 0.00000861598573976718000

centenary n century 1.00000000000000000000000

censor n judge 0.84071057729004300000000

censor n examiner 0.01448085660999590000000

censor n complainer 0.01448085660999590000000

censor n critic 0.10136599626997300000000

censor n inspector 0.02896171321999190000000

cannister n canister 1.00000000000000000000000

amenable a manageable 0.16666666666666700000000

amenable a reasonable 0.66666666666666700000000

amenable a flexible 0.16666666666666700000000

adjudicate v hear 0.00622892797285005000000

adjudicate v order 0.00325162551365068000000

adjudicate v determine 0.91341749097468600000000

adjudicate v resolve 0.02493417101282120000000

adjudicate v try 0.00508548795149216000000

adjudicate v decide 0.03533332216670430000000

adjudicate v judge 0.00014177630758092000000

adjudicate v rule 0.00014177630758092000000

adjudicate v settle 0.01146542179263360000000

abdication n resignation 1.00000000000000000000000

march v file 0.03002098894875680000000

march v range 0.01197442733562950000000

march v step 0.00001346302348634240000

march v edge 0.08602318488239560000000

march v parade 0.02993606833907370000000

march v advance 0.32890289743556900000000

march v pace 0.00000103561719125710000

march v travel 0.04386391000801270000000

march v stalk 0.00000103561719125710000

march v walk 0.00014306364797645700000

march v proceed 0.00124000159432987000000

march v stride 0.01842219590096840000000

march v demonstrate 0.10780975440142400000000

march v move 0.24730117763121800000000

march v process 0.00000828493753005684000

march v hike 0.00035012945425817200000

march v rise 0.06261448674018100000000

march v storm 0.02993606833907370000000

march v progress 0.00010278478399682000000

march v develop 0.00133297012735467000000

march v stamp 0.00000103561719125710000

march v evolve 0.00000103561719125710000

peace n rest 0.00582141629343193000000

peace n union 0.04343888285559820000000

peace n brotherhood 0.01711329809500360000000

peace n quiet 0.00122218400702590000000

peace n accord 0.01854319003862110000000

peace n concord 0.00095246922053806400000

peace n armistice 0.01261542100241200000000

peace n conciliation 0.00733472196576215000000

peace n well-being 0.00084248940957834000000

peace n agreement 0.12347032008576700000000

peace n composure 0.00000118404497915717000

peace n unanimity 0.00032682121374210900000

peace n cease-fire 0.00000570919035696532000

peace n resignation 0.00187485468776354000000

peace n ceasefire 0.13487740289896400000000

peace n order 0.12909715835066500000000

peace n inactivity 0.00003422659619000640000

peace n unity 0.00969798225380335000000

peace n calm 0.00948486233347240000000

peace n appeasement 0.00000285727610894348000

peace n truce 0.04141702300539320000000

peace n abeyance 0.00008209538146010180000

peace n reconciliation 0.04857323614472910000000

peace n love 0.00540519692530418000000

peace n sympathy 0.00009054655076721260000

peace n comfort 0.00003933068017815260000

peace n silence 0.00009294786038633350000

peace n settlement 0.27514475261946300000000

peace n friendship 0.02305265898459540000000

peace n satisfaction 0.00021541027344691100000

peace n quietness 0.00000570919035696532000

peace n ease 0.00372848521409537000000

peace n harmony 0.01828778270511170000000

peace n treaty 0.03480386064335990000000

peace n cooperation 0.03230351200156880000000

touch n drop 0.00506875356967334000000

touch n holding 0.00005063859272557240000

touch n addition 0.00209695066205918000000

touch n coating 0.00013559039546159000000

touch n bit 0.12675375173690300000000

touch n trace 0.00013001309400293300000

touch n tap 0.00000123012406240125000

touch n communication 0.00002120311481870270000

touch n ghost 0.00002322058863247050000

touch n contact 0.42753012057421400000000

touch n structure 0.00007296072818076650000

touch n system 0.00058750165346638700000

touch n procedure 0.00004706652104286600000

touch n operation 0.00563577918851369000000

touch n approach 0.00562464183954804000000

touch n ability 0.00001673344998108330000

touch n apprehension 0.00000123012406240125000

touch n connection 0.00000123012406240125000

touch n feature 0.00015809300718320000000

touch n sense 0.00033660114944161700000

touch n hit 0.00083244908714126300000

touch n way 0.02335395580888640000000

touch n motion 0.00066424843850708200000

touch n element 0.02878836062044210000000

touch n line 0.03791650644068190000000

touch n feeling 0.00397391159661605000000

touch n understanding 0.00156148159288616000000

touch n blow 0.00292935847700088000000

touch n suggestion 0.00006421013322292910000

touch n style 0.00018162328734520200000

touch n dab 0.00000788778764783506000

touch n suspicion 0.00008358233521240960000

touch n pressure 0.00173913908394922000000

touch n perception 0.00169719586456182000000

touch n knack 0.00000665766358543381000

touch n point 0.29312690955574700000000

touch n action 0.00163983948565799000000

touch n surface 0.00000246024812480249000

touch n manner 0.00000123012406240125000

touch n strike 0.00161232476047419000000

touch n detail 0.00697217210764577000000

touch n impression 0.01287653667299050000000

touch n mo 0.00022978081557222700000

touch n signature 0.00010622429039522600000

touch n technique 0.00002083202865169580000

touch n stroke 0.00021595701299987600000

touch n attack 0.00029823853659222100000

touch n gift 0.00000123012406240125000

touch n impact 0.00048931253207599200000

touch n hint 0.00000665766358543381000

touch n spot 0.00127110141763043000000

touch n grain 0.00002786077840413650000

touch n facility 0.00235548551727547000000

touch n movement 0.00043860491753532700000

touch n outside 0.00010622429039522600000

touch n trick 0.00010713866439906300000

ventilator n fan 1.00000000000000000000000

vacation v visit 0.00250821600613893000000

vacation v travel 0.00415492640397460000000

vacation v explore 0.00091939116091785500000

vacation v holiday 0.99241746642896900000000

acceptable a okay 0.00410278733728104000000

acceptable a due 0.00783175033703520000000

acceptable a competent 0.00009190705579039730000

acceptable a respectable 0.00183801373041142000000

acceptable a right 0.05290079440427640000000

acceptable a worthy 0.03096315216656050000000

acceptable a grateful 0.00070764116209486200000

acceptable a indifferent 0.00019910740385445700000

acceptable a pleasant 0.00054097478913128300000

acceptable a mediocre 0.00008512270872873820000

acceptable a satisfying 0.00082289867475810000000

acceptable a popular 0.05898577786025450000000

acceptable a attractive 0.05036518580407880000000

acceptable a fair 0.22022528541119300000000

acceptable a minimum 0.00522279740049832000000

acceptable a appropriate 0.06277803431466460000000

acceptable a sufficient 0.02225026162414960000000

acceptable a reasonable 0.16135648941570200000000

acceptable a comforting 0.00003742274061819850000

acceptable a deserving 0.00013055140226936700000

acceptable a adequate 0.03077365626266640000000

acceptable a tolerable 0.00549791595757249000000

acceptable a common 0.03564266895970250000000

acceptable a agreeable 0.10307952805838900000000

acceptable a satisfactory 0.03982831107935370000000

acceptable a welcome 0.01255902799697750000000

acceptable a impressive 0.00205457711425391000000

acceptable a proper 0.01338619569327120000000

acceptable a commendable 0.00003947930134810830000

acceptable a admissible 0.00010691111774428300000

acceptable a minimal 0.00726089536138958000000

acceptable a so-so 0.00013125407800163900000

acceptable a gratifying 0.00002609699652824130000

acceptable a suitable 0.05057917799471540000000

acceptable a timely 0.00041780186683842400000

acceptable a decent 0.00001077234322000250000

acceptable a desirable 0.01667523446475740000000

acceptable a fit 0.00049453960991977000000

southeast a southeastward 0.00543947966320666000000

southeast a southeastern 0.99456052033679300000000

samarang n semarang 1.00000000000000000000000

answer v say 0.31654176275665800000000

answer v retaliate 0.00398103327508669000000

answer v back 0.00003761383044577470000

answer v claim 0.00037220882604016500000

answer v argue 0.00047565537890984600000

answer v backfire 0.00000268893275480996000

answer v rectify 0.00011344419522917600000

answer v resolve 0.02114116245641240000000

answer v qualify 0.00064765258978167600000

answer v defend 0.00941768561178828000000

answer v respond 0.20298030305899800000000

answer v do 0.15746089942160300000000

answer v remark 0.00000356310450038019000

answer v meet 0.04612142794301120000000

answer v explain 0.00146719969469880000000

answer v redress 0.00002955665456127130000

answer v fill 0.00086165098373312900000

answer v react 0.00070518991883403300000

answer v solve 0.00570544231390714000000

answer v acknowledge 0.00235375989598366000000

answer v suffice 0.00005452042964925040000

answer v work 0.01212209911887660000000

answer v satisfy 0.01367163016363910000000

answer v crush 0.00010923844761051600000

answer v rebut 0.00018658855115985700000

answer v repay 0.00091771334825658800000

answer v reply 0.06009882318149600000000

answer v dope 0.00000591133091225415000

answer v justify 0.00068110616785115500000

answer v plead 0.00004847672430510240000

answer v disprove 0.00000356310450038019000

answer v contest 0.00182944846465186000000

answer v clarify 0.00091705618245032800000

answer v squelch 0.00000458252715074929000

answer v interpret 0.00022118581191221900000

answer v return 0.05088437816384860000000

answer v redeem 0.00046235495649446100000

answer v confirm 0.01426387219981070000000

answer v dispute 0.00023515532054195000000

answer v smash 0.00001425241800152070000

answer v deny 0.03807493214341500000000

answer v suit 0.01290612973494540000000

answer v conform 0.00003040049593867800000

answer v pass 0.00172139077924302000000

answer v field 0.00001248547925976740000

answer v rejoin 0.00003076370377546030000

answer v correspond 0.00321865250750754000000

answer v serve 0.00046096045566344200000

answer v quash 0.00031567486280414100000

answer v settle 0.01607675238139040000000

recall v commemorate 0.05717112794148540000000

recall v disqualify 0.00001112509624724030000

recall v place 0.00051626428185069100000

recall v cite 0.00146870221634200000000

recall v suspend 0.10852413519356900000000

recall v retrieve 0.00020478327931904000000

recall v arouse 0.00000370836541574676000

recall v overrule 0.00012115240573943500000

recall v retract 0.00001235284466176790000

recall v rescind 0.00011731584896545600000

recall v withdraw 0.34013818461263500000000

recall v review 0.00162154320063104000000

recall v retain 0.00034449904120284900000

recall v recognize 0.00032529479083052800000

recall v think 0.13968793742530800000000

recall v renew 0.00075425010575102400000

recall v cancel 0.00465784992001094000000

recall v nullify 0.00002575492742580760000

recall v revive 0.00015483979637099600000

recall v discharge 0.00016861663072942000000

recall v remember 0.14248882699426000000000

recall v echo 0.03628786201822780000000

recall v reassemble 0.00004683087151170160000

recall v return 0.08554028041666150000000

recall v veto 0.00000741673083149352000

recall v remind 0.00000701273954653330000

recall v summon 0.06508027906587520000000

recall v applaud 0.00010162962237749600000

recall v deny 0.01415233384856600000000

recall v revoke 0.00009197197230799330000

recall v extract 0.00003928824824786390000

recall v annul 0.00003460303715624860000

recall v repeal 0.00007493755144583580000

recall v retrace 0.00001728895849204220000

eat v melt 0.00051542437165450800000

eat v swallow 0.05442390519471600000000

eat v pick 0.07260180524289470000000

eat v demolish 0.00013267473106853800000

eat v dissipate 0.00100312342281038000000

eat v squander 0.00006104117066141480000

eat v chew 0.00090559753448158900000

eat v want 0.08899515578090220000000

eat v attack 0.00029290553243977300000

eat v disintegrate 0.00007348152149606740000

eat v have 0.59824553213591200000000

eat v worry 0.00998715554985311000000

eat v destroy 0.00120495611978026000000

eat v gobble 0.02173434082755820000000

eat v grieve 0.00000910275338303425000

eat v inhale 0.00164600626287650000000

eat v savor 0.00010738719386116600000

eat v feed 0.01400099425893580000000

eat v try 0.00786679330062366000000

eat v disappear 0.00059510936585931800000

eat v ingest 0.00000694782508517509000

eat v erode 0.09846994435503130000000

eat v dissolve 0.00019188828023230200000

eat v discuss 0.00908845413314975000000

eat v spill 0.00001015043679352500000

eat v digest 0.00002368435251822530000

eat v consume 0.00026016278697999600000

eat v devastate 0.00009950629591331150000

eat v ruin 0.00009013167497953240000

eat v exhaust 0.00015775574238135700000

eat v waste 0.00017000097002811800000

eat v deplete 0.01632245112613490000000

eat v desire 0.00062760858946978000000

eat v bite 0.00006252855923452270000

eat v disturb 0.00001629260029955590000

ornament n celebrity 0.06249999999999930000000

ornament n trimming 0.06249999999999930000000

ornament n star 0.06249999999999930000000

ornament n show 0.31250000000000200000000

ornament n sham 0.06249999999999930000000

ornament n gilt 0.18750000000000100000000

ornament n favorite 0.25000000000000000000000

crown n ridge 0.00031611875655786800000

crown n monarch 0.02253790073497770000000

crown n territory 0.00499076200397968000000

crown n crest 0.00008605874051150660000

crown n jacket 0.00001117443830476400000

crown n zenith 0.00050201366462879200000

crown n cluster 0.00000444508251282372000

crown n sovereign 0.00688856283865584000000

crown n distinction 0.00020498237153934000000

crown n meridian 0.00012061892015041300000

crown n king 0.01237869357904810000000

crown n apex 0.00006402894747440530000

crown n climax 0.00003858263567912350000

crown n extreme 0.00007859720901931740000

crown n top 0.00548053747260846000000

crown n power 0.00890758316253163000000

crown n award 0.01364068653357130000000

crown n pinnacle 0.00003265477339607060000

crown n peak 0.00762933429551256000000

crown n block 0.01331902167028480000000

crown n empress 0.00015936964670628100000

crown n palm 0.00022770394589496600000

crown n skull 0.00000796636612201832000

crown n rex 0.00052549327632829700000

crown n helmet 0.00000228427327911652000

crown n bloom 0.00007891865110412130000

crown n noodle 0.00001063598816779780000

crown n culmination 0.00032307289672092200000

crown n laurel 0.00010623704357386000000

crown n majesty 0.00000444508251282372000

crown n bay 0.00027114097066651000000

crown n vertex 0.00034387702439892700000

crown n consummation 0.00009845689963568030000

crown n emblem 0.00000111895932437475000

crown n heyday 0.00002854949881282920000

crown n cap 0.00115590919636840000000

crown n alp 0.00000326966124812285000

crown n bluff 0.00004601330810142320000

crown n design 0.00040412055495119200000

crown n pate 0.00000674754740179742000

crown n kingdom 0.00025866318088087500000

crown n sovereignty 0.00359132350332512000000

crown n authority 0.00535612051805014000000

crown n prize 0.00144542968800573000000

crown n prime 0.00072623875698953400000

crown n perfection 0.00009692409954478320000

crown n queen 0.00052337108543833300000

crown n head 0.01103252372413630000000

crown n symbol 0.00020388866270580200000

crown n point 0.84796096674705900000000

crown n ruler 0.00002384320602991620000

crown n apogee 0.00007463842524756520000

crown n circle 0.00043254435396543500000

crown n royalty 0.00076226893830777700000

crown n rule 0.00485657226386851000000

crown n ring 0.00003577448179354770000

crown n corolla 0.00001077532451875780000

crown n regalia 0.00000289786411095146000

crown n memento 0.00000229506979703740000

crown n honor 0.00134622231784430000000

crown n poll 0.01029756930086030000000

crown n kudos 0.00007347985760023270000

crown n spire 0.00000478108940118843000

crown n dominion 0.00022770247972583200000

crown n reward 0.00004772087704102420000

crown n jurisdiction 0.00002278476490167930000

crown n trophy 0.00300323973489996000000

crown n praise 0.00000131082674048621000

crown n hat 0.00001952184917004090000

crown n summit 0.00620237596348130000000

crown n emperor 0.00003166949418291210000

crown n monarchy 0.00028687292811175400000

rig n carriage 0.00027574121256803700000

rig n supplies 0.01663161856147700000000

rig n truck 0.15071270396094700000000

rig n belongings 0.06597632759313280000000

rig n wheeler 0.00003914644734160100000

rig n semi 0.00177908976864750000000

rig n swindle 0.00052371967733431500000

rig n victoria 0.00173054213460463000000

rig n turnout 0.07546580529217320000000

rig n materials 0.00035118929495840700000

rig n gear 0.00055148242513608400000

rig n dress 0.00052371967733431500000

rig n uniform 0.00181930361238880000000

rig n stuff 0.00479515216353620000000

rig n junk 0.00035118929495840700000

rig n equipment 0.67646763231309500000000

rig n kit 0.00200563657036666000000

recognition n confession 0.00006564173559305210000

recognition n verification 0.00018061343280413000000

recognition n attention 0.36281383033726500000000

recognition n notice 0.00018242390247679000000

recognition n acclaim 0.18025220593852200000000

recognition n acknowledgement 0.05546221721185290000000

recognition n realization 0.00119372321813591000000

recognition n credit 0.05983910193329110000000

recognition n memory 0.00137457892223994000000

recognition n acceptance 0.30050320843358500000000

recognition n thanks 0.00110899890685540000000

recognition n perception 0.02316169905230860000000

recognition n appreciation 0.00053015354687800600000

recognition n admission 0.00013751916924496800000

recognition n honor 0.00001247139611772580000

recognition n regard 0.01311597112723590000000

recognition n identification 0.00006564173559305210000

mitigation n improvement 0.50000000000000000000000

mitigation n easing 0.50000000000000000000000

lumpy a crude 0.92423599058576600000000

lumpy a uneven 0.00390731373376919000000

lumpy a bumpy 0.00130243791125639000000

lumpy a rough 0.00651218955628198000000

lumpy a heavy 0.02474632031387160000000

lumpy a choppy 0.03929574789905470000000

afford v confer 0.00002638126704209690000

afford v grant 0.04455889550783780000000

afford v supply 0.00497019417142178000000

afford v bear 0.00642897005813038000000

afford v generate 0.00047730501723286600000

afford v award 0.00002735183008667210000

afford v sustain 0.02835610627889430000000

afford v offer 0.03311272150283860000000

afford v allow 0.05344487010615490000000

afford v provide 0.12519608695962200000000

afford v open 0.00239536746515711000000

afford v accord 0.04444254199079270000000

afford v produce 0.09255702825464260000000

afford v support 0.17303796398950500000000

afford v manage 0.01442541534758970000000

afford v administer 0.00087142239197632700000

afford v stand 0.01212508859683080000000

afford v spare 0.00742378585002552000000

afford v give 0.31079627842714300000000

afford v render 0.00005746570808571860000

afford v extend 0.04526875927898970000000

turnaround n reversal 0.96842121042321500000000

turnaround n turnabout 0.00250930029008964000000

turnaround n reverse 0.02906948928669510000000

evaluate v account 0.00252787733813712000000

evaluate v determine 0.05178655664657110000000

evaluate v price 0.01237174108876090000000

evaluate v valuate 0.00002768877202470200000

evaluate v value 0.01216279767156870000000

evaluate v test 0.05293941687963490000000

evaluate v calculate 0.15785490266150600000000

evaluate v ascertain 0.00108676105904660000000

evaluate v weigh 0.09140778368975970000000

evaluate v assess 0.36279199028127000000000

evaluate v rate 0.00019682888494792500000

evaluate v appraise 0.00001628959773933500000

evaluate v measure 0.00818666169285072000000

evaluate v assay 0.00002396280343364150000

evaluate v balance 0.00241344037024944000000

evaluate v count 0.00503358782866174000000

evaluate v rank 0.00004440637920274740000

evaluate v survey 0.00049025562268627000000

evaluate v judge 0.21249458676769000000000

evaluate v estimate 0.02265366464401480000000

evaluate v figure 0.00021000122959174600000

evaluate v reckon 0.00025555707291353000000

evaluate v compute 0.00022707382920336000000

evaluate v gauge 0.00279616718853574000000

hoy n barge 1.00000000000000000000000

highness n grace 1.00000000000000000000000

efficient a economic 0.00034864840476506800000

efficient a effective 0.48504774187874300000000

efficient a ready 0.03205660599198250000000

efficient a systematic 0.00103641295045742000000

efficient a competent 0.00001677259141942580000

efficient a talented 0.00017711223961977200000

efficient a tough 0.04362619754789250000000

efficient a conducive 0.00021616951088685900000

efficient a potent 0.00043914043023096300000

efficient a cost-effective 0.01206249414243290000000

efficient a energetic 0.00013761320757374300000

efficient a economical 0.01404561561224240000000

efficient a clever 0.00049163928841218300000

efficient a powerful 0.02258998330011110000000

efficient a fitting 0.00123210831501584000000

efficient a shrewd 0.00001846241693122520000

efficient a profitable 0.13269631107129700000000

efficient a polished 0.00030152722265722000000

efficient a virtuous 0.00001656011175770010000

efficient a skilful 0.00001677259141942580000

efficient a adequate 0.00066351733203468700000

efficient a businesslike 0.00006709036567770350000

efficient a prepared 0.00072623701504822800000

efficient a eligible 0.00026139956338476200000

efficient a valid 0.00565197054453204000000

efficient a capable 0.03834056926780740000000

efficient a decisive 0.00193379913385996000000

efficient a useful 0.01222426274077240000000

efficient a methodical 0.00012679446536149500000

efficient a valuable 0.00645600984748211000000

efficient a suitable 0.00096717299125676800000

efficient a dynamic 0.06775268267801270000000

efficient a practical 0.00007405635711369150000

efficient a able 0.00578587582337233000000

efficient a productive 0.01817867624700170000000

efficient a experienced 0.00799590924289601000000

efficient a practiced 0.00007531110050020870000

efficient a active 0.06280330612979800000000

efficient a skilled 0.00003354518283885160000

efficient a orderly 0.00044297994799905200000

efficient a fit 0.02256341797474430000000

efficient a vigorous 0.00030152722265722000000

how r surely 0.01138868292786120000000

how r why 0.73240344503438800000000

how r certainly 0.10079494074926200000000

how r absolutely 0.11174877648767300000000

how r positively 0.04366415480081610000000

mohammad n mohammed 0.78301644713856400000000

mohammad n muhammad 0.21698355286143600000000

flattering a favourable 0.86454002980799500000000

flattering a favorable 0.13545997019200500000000

father-in-law n father 0.09375000000000000000000

father-in-law n relative 0.90625000000000000000000

ministerial a pastoral 0.00023119959993097100000

ministerial a diplomatic 0.07326100128539820000000

ministerial a ambassadorial 0.01059262952321780000000

ministerial a valid 0.00274936084758677000000

ministerial a official 0.91316580874386600000000

flotation n floatation 1.00000000000000000000000

congratulatory a positive 1.00000000000000000000000

clobber v batter 1.00000000000000000000000

exception n shutout 0.00003490493086520630000

exception n difference 0.07858052626230360000000

exception n challenge 0.00496260966725436000000

exception n omission 0.00035729846848259800000

exception n anomaly 0.00001303071265791650000

exception n complaint 0.00393842043186630000000

exception n segregation 0.00001537947008336170000

exception n freak 0.00010353112771061900000

exception n rarity 0.00190577345375074000000

exception n quirk 0.02116984056974760000000

exception n objection 0.00100888441919049000000

exception n irregularity 0.00036609559199906300000

exception n exemption 0.51143916033529200000000

exception n allowance 0.00023919000158285000000

exception n reservation 0.00094456388389716200000

exception n repudiation 0.00007081003646921910000

exception n rejection 0.00007477380352531160000

exception n deviation 0.00001325462208269680000

exception n privilege 0.00038658522103077900000

exception n question 0.32521376024069700000000

exception n limitation 0.03721734186212890000000

exception n dissent 0.00056287827312231700000

exception n exclusion 0.00047396997789853500000

exception n waiver 0.00038061138414352900000

exception n protest 0.01052680525221820000000

chastise v reprimand 0.11111111111111100000000

chastise v correct 0.11111111111111100000000

chastise v trim 0.22222222222222200000000

chastise v punish 0.55555555555555500000000

cavern n cave 1.00000000000000000000000

broadly r generally 0.98827368811041100000000

broadly r loosely 0.01172631188958880000000

ruanda n rwanda 1.00000000000000000000000

enforce v intimidate 0.00008713968076637220000

enforce v constrain 0.00003250209126755300000

enforce v fulfill 0.00005390062698416420000

enforce v whip 0.00000046957582528172200

enforce v threaten 0.00131872066343234000000

enforce v demand 0.00017963111014702700000

enforce v emphasize 0.00004500977451746540000

enforce v apply 0.29651488842848800000000

enforce v compel 0.00004737798810996950000

enforce v harass 0.00002641882736912710000

enforce v impose 0.41069270108574400000000

enforce v invoke 0.00012892316899887900000

enforce v implement 0.08895364534753650000000

enforce v conduct 0.00311333309888802000000

enforce v perform 0.00134730088412871000000

enforce v expect 0.02945428825753520000000

enforce v stress 0.00392804312406437000000

enforce v hound 0.00000046957582528172200

enforce v press 0.00017540632916152000000

enforce v realize 0.00326642752467442000000

enforce v coerce 0.00001755137364604010000

enforce v make 0.03433782028763760000000

enforce v pressure 0.00002086638556612350000

enforce v discharge 0.00039662627047573900000

enforce v oversee 0.04880101982427250000000

enforce v reinforce 0.00015145954098071500000

enforce v execute 0.00130024474197704000000

enforce v direct 0.00001965854248886320000

enforce v squeeze 0.00050770481357611100000

enforce v support 0.00359653466137725000000

enforce v manage 0.00871418267222296000000

enforce v administer 0.00250221364614784000000

enforce v force 0.01381683275640210000000

enforce v cause 0.00000284255659327052000

enforce v supervise 0.00083531037498500200000

enforce v complete 0.02651202585638820000000

enforce v prosecute 0.00124131419455515000000

enforce v strain 0.00043050225333693300000

enforce v dictate 0.00004933852480556000000

enforce v impress 0.00004515810968667830000

enforce v lash 0.00000798176737240151000

enforce v accomplish 0.00036808423889119000000

enforce v require 0.00277544851033374000000

enforce v exert 0.00001664753367472050000

enforce v effect 0.00018062442385129400000

enforce v sanction 0.00012373517167985600000

enforce v urge 0.00048894015315429800000

enforce v control 0.01068134797058520000000

enforce v drive 0.00269138567987143000000

arctic a polar 0.89473684210526300000000

arctic a cool 0.10526315789473700000000

custody n holding 0.00004423534234218060000

custody n retention 0.00038509629832325100000

custody n safeguard 0.00000924371976607128000

custody n direction 0.00009133465862786180000

custody n care 0.02238665922714470000000

custody n protection 0.01188067271400060000000

custody n responsibility 0.00117951574344584000000

custody n jail 0.35867755800990200000000

custody n arrest 0.07691107672525620000000

custody n hold 0.01916230370461760000000

custody n aegis 0.00002639438785182420000

custody n charge 0.05006892605614610000000

custody n duress 0.00004648635793631560000

custody n watch 0.00105304420523189000000

custody n supervision 0.00093138660169949100000

custody n detention 0.33296879539370400000000

custody n control 0.06500797290963790000000

custody n surveillance 0.01380320833977570000000

custody n guardianship 0.04453393090299040000000

custody n ward 0.00030072264088908900000

custody n guidance 0.00003105481460983170000

custody n imprisonment 0.00050038124610101600000

car n carriage 0.00868424965314887000000

car n van 0.01090418893438060000000

car n motor 0.04109468330529760000000

car n bomb 0.01157256805328990000000

car n cab 0.00002721851915169010000

car n limousine 0.00123124155968838000000

car n railcar 0.00266454983772056000000

car n coach 0.00139125922577033000000

car n wreck 0.00018934293613622600000

car n auto 0.26447091716569800000000

car n cart 0.00131121961685661000000

car n coupe 0.00471969046526999000000

car n heap 0.00010065536982473900000

car n saloon 0.00530757111374937000000

car n bus 0.04167255743667850000000

car n machine 0.00331992650505960000000

car n crate 0.00013090855884603500000

car n wheels 0.00000974695474989606000

car n landau 0.00000232622011257731000

car n diner 0.00000189946871475287000

car n automobile 0.11095153885037400000000

car n sleeper 0.00219334632051283000000

car n cabriolet 0.00000026274839475951900

car n smoker 0.00018891225801787800000

car n taxi 0.00117458900095516000000

car n convertible 0.00000960090639423399000

car n hansom 0.00000143141339201958000

car n pullman 0.00000057340182920638000

car n limo 0.00004410039911143530000

car n vehicle 0.45674541119792000000000

car n sedan 0.02573295829627320000000

car n wagon 0.00415055430668071000000

massachusetts n ma 1.00000000000000000000000

windshield n windscreen 1.00000000000000000000000

vacationer n vacationist 1.00000000000000000000000

inch n length 1.00000000000000000000000

festival n event 0.01205516241037250000000

festival n rite 0.00286068569001835000000

festival n ritual 0.00000098948859640069600

festival n bust 0.00000353531537419237000

festival n reunion 0.00001032586718839600000

festival n symposium 0.00013284016453568700000

festival n gala 0.00000042047883264519100

festival n festivity 0.00708845054228408000000

festival n feast 0.09114824030139710000000

festival n sport 0.00090358181155485800000

festival n ball 0.00018475825947683900000

festival n repast 0.00000042047883264519100

festival n ceremony 0.00516534554462930000000

festival n entertainment 0.00000084095766529039700

festival n fiesta 0.00051691539961187500000

festival n orgy 0.00476710868530951000000

festival n remembrance 0.00000042047883264519100

festival n anniversary 0.00000049474429820034800

festival n fair 0.00052358873852112700000

festival n celebration 0.07848840341489370000000

festival n occasion 0.00008850579160825120000

festival n reception 0.00099984630250264800000

festival n birthday 0.00002000998899793040000

festival n spree 0.00204304657941836000000

festival n spread 0.00046489007869311500000

festival n party 0.00289800522059593000000

festival n fun 0.00000190471172724624000

festival n holiday 0.78961333353085000000000

festival n carnival 0.00001792902338084100000

working n operation 0.95239420478624600000000

working n activity 0.00447389194754044000000

working n action 0.03319770370919120000000

working n process 0.00927898269363660000000

working n performance 0.00065521686338606000000

smattering n handful 1.00000000000000000000000

shoddy a poor 0.57142857142857100000000

shoddy a cheap 0.42857142857142800000000

alcohol n ethanol 0.00135894210319778000000

alcohol n methanol 0.00076805066346126100000

alcohol n drink 0.52289713509988900000000

alcohol n medicine 0.00236869721695658000000

alcohol n liquor 0.47260717491649500000000

scanty a irregular 0.00008467376339551500000

scanty a poor 0.00384138411373904000000

scanty a negligible 0.00144904067182856000000

scanty a light 0.00747251722097136000000

scanty a tight 0.00598985993995995000000

scanty a paltry 0.00002598299516576070000

scanty a thin 0.00992346538427207000000

scanty a limited 0.33631873691147600000000

scanty a ragged 0.00000866099838858689000

scanty a modest 0.01945956642896550000000

scanty a sparse 0.00004233688169775750000

scanty a slight 0.00048399580569768500000

scanty a narrow 0.00046570569867533400000

scanty a spotty 0.00000866099838858689000

scanty a few 0.00181676135942423000000

scanty a rare 0.00073801709588423100000

scanty a infrequent 0.00004233688169775750000

scanty a small 0.00408680337656800000000

scanty a minimal 0.00067758239383960100000

scanty a little 0.00014433264187044700000

scanty a inadequate 0.00032047349687975400000

scanty a slim 0.01787618661086920000000

scanty a short 0.00064371422385495200000

scanty a insufficient 0.00228001753543498000000

scanty a mere 0.00001732199677717380000

scanty a lean 0.00016891430333050000000

scanty a sporadic 0.00061360271136654600000

scanty a scattered 0.00008467376339551500000

scanty a scarce 0.00021265251873396500000

scanty a scant 0.58418001081099900000000

scanty a delicate 0.00038103193527981900000

scanty a stingy 0.00004233688169775750000

scanty a meager 0.00005630476777683320000

scanty a shy 0.00004233688169775750000

push n progress 0.03747469394528160000000

push n advance 0.01025722018524410000000

push n sally 0.00000693119247920155000

push n enterprise 0.02119509081382070000000

push n drive 0.19468956510197500000000

push n weight 0.00953352708593997000000

push n salvo 0.00003483657346369230000

push n trouble 0.00017749544559040600000

push n difficulty 0.00863932740718301000000

push n mass 0.00009129966642096660000

push n hit 0.00006967314692738470000

push n force 0.07807713669910430000000

push n reserve 0.02558675543999360000000

push n broadside 0.03108293267297960000000

push n spirit 0.00020712646060910300000

push n raid 0.00009306884596381510000

push n blow 0.00000693119247920155000

push n charge 0.03166920138217480000000

push n assault 0.09946538455353460000000

push n pressure 0.08957209766994110000000

push n instance 0.00000693119247920155000

push n energy 0.00097404741270351600000

push n strike 0.00426694985335705000000

push n ambition 0.00192722576493100000000

push n attack 0.11333937161323500000000

push n press 0.00010933084279969700000

push n impact 0.10140712296289700000000

push n stress 0.00174069197790171000000

push n insistence 0.00008378148968458100000

push n need 0.03329167542201920000000

push n thrust 0.10066271321187100000000

push n plight 0.00401070099006188000000

push n urgency 0.00024916178495374400000

outlive v outlast 1.00000000000000000000000

state n outlook 0.00125249135133376000000

state n territory 0.00692641145850998000000

state n level 0.00709568580124384000000

state n event 0.00128613992958603000000

state n dilemma 0.00002511808358235090000

state n disposition 0.00003008026955255350000

state n domination 0.00021799639062576400000

state n requirement 0.00359079218467543000000

state n canton 0.00022524448115281000000

state n dos 0.00158817352451973000000

state n form 0.00109013423664414000000

state n environment 0.00211851714404688000000

state n grade 0.00124756972354286000000

state n government 0.20655962607203000000000

state n estate 0.00180423120184867000000

state n class 0.00096318814675042900000

state n humor 0.00000850800048010222000

state n prerequisite 0.00000480344951217211000

state n power 0.01222450743189090000000

state n status 0.00179276182382697000000

state n resources 0.00023385456254619000000

state n occurrence 0.00002175636191694170000

state n phase 0.00067184451659134300000

state n welfare 0.00182369174356008000000

state n realm 0.00000325694040672824000

state n juncture 0.00001143176747074630000

state n caste 0.00000013796627579021200

state n stipulation 0.00002500106595238810000

state n essential 0.00001264904762612890000

state n regime 0.00311451464917340000000

state n standing 0.00081912664928860100000

state n imperative 0.00000525988744962781000

state n order 0.01014819208117430000000

state n element 0.00008249328699647960000

state n commonwealth 0.00267338441660806000000

state n category 0.00123812928666893000000

state n shire 0.00003563551678452450000

state n nature 0.00009825908095021950000

state n kingdom 0.01135703500433550000000

state n circumstance 0.00035855060942406000000

state n rank 0.00042116333053647600000

state n parliament 0.02021674702259360000000

state n sovereignty 0.00066790342087579500000

state n predicament 0.00000197032903420720000

state n position 0.00521633328090448000000

state n region 0.05119992546876720000000

state n command 0.00027160214588602500000

state n case 0.00932002789421585000000

state n fix 0.00001768785174438780000

state n style 0.00068735835717784100000

state n shape 0.00156654534210769000000

state n reputation 0.00056285378789968600000

state n pickle 0.00000364343758866959000

state n department 0.01074076263457070000000

state n condition 0.00790774125213311000000

state n place 0.00299550835421014000000

state n proviso 0.00000017610054740845600

state n empire 0.00024790740897762700000

state n corner 0.00006192457229520560000

state n rule 0.01077739596600220000000

state n occasion 0.00030988097386064400000

state n quandary 0.00000072372076051286400

state n character 0.00005413715458453720000

state n country 0.32677497315478700000000

state n nation 0.08827826234459490000000

state n property 0.00558256994329550000000

state n dominion 0.00011412265782110500000

state n time 0.01399687264231370000000

state n stamp 0.00015144334930930900000

state n situation 0.00702393611666099000000

state n people 0.02378424803410380000000

state n republic 0.05656286702604430000000

state n glory 0.00000491637481370948000

state n county 0.01857966111900150000000

state n district 0.01078519877090560000000

state n authorities 0.00066856659699941800000

state n limitation 0.00007623495899283930000

state n mean 0.00004058349454300620000

state n administration 0.01260140624714210000000

state n land 0.00170795268953551000000

state n contingency 0.00001562736091537980000

state n province 0.01892785844532890000000

state n station 0.00543647147973891000000

state n footing 0.00000651912506446214000

state n plight 0.00048942479033319400000

state n dignity 0.00001225953757839420000

state n monarchy 0.00031299312106088900000

state n stage 0.00202892566528537000000

possible a feasible 0.02309813842973070000000

possible a affordable 0.00136077946482033000000

possible a accessible 0.00005137798743876970000

possible a potential 0.38200432654679800000000

possible a casual 0.00001333167447692010000

possible a contingent 0.00142548073620959000000

possible a achievable 0.00210904549404038000000

possible a thinkable 0.00003125181360845660000

possible a understandable 0.00132937712228833000000

possible a workable 0.00096537532723527300000

possible a conceivable 0.00487953316258557000000

possible a permissible 0.00009616587573371520000

possible a attainable 0.00082824242867065000000

possible a practicable 0.18811490809429600000000

possible a plausible 0.00025125260738592900000

possible a hypothetical 0.00004433986793838960000

possible a reasonable 0.01770708223607120000000

possible a likely 0.24060201705455300000000

possible a tolerable 0.00039225342328349000000

possible a incidental 0.00000560438109837733000

possible a welcome 0.00999163175886251000000

possible a promising 0.00929720456556687000000

possible a admissible 0.00002241323731412930000

possible a hopeful 0.02930482071388470000000

possible a doable 0.00029821196058890100000

possible a prospective 0.02065455742393670000000

possible a believable 0.00002416933359067100000

possible a tenable 0.00026348266807623800000

possible a obtainable 0.00002443189674613900000

possible a viable 0.00744978526200443000000

possible a fortuitous 0.00003088844140024690000

possible a credible 0.00599770863492062000000

possible a probable 0.04010922127909520000000

possible a liable 0.00105448135511987000000

possible a desirable 0.01016710774063000000000

mute a quiet 0.09768989982231160000000

mute a dumb 0.78565338627848100000000

mute a calm 0.02129430747404120000000

mute a silent 0.09536240642516630000000

best r better 0.86020798180147700000000

best r extremely 0.00032164673428357500000

best r most 0.13706634963639200000000

best r first 0.00240402182784653000000

monstrous a strange 0.80477276429385900000000

monstrous a great 0.08342156703046100000000

monstrous a desperate 0.02838410164521910000000

monstrous a terrible 0.08342156703046100000000

miraculous a amazing 0.07820339104456230000000

miraculous a extraordinary 0.13033898507427100000000

miraculous a staggering 0.05213559402970820000000

miraculous a astounding 0.02606779701485410000000

miraculous a unusual 0.25405600207724600000000

miraculous a unprecedented 0.02606779701485410000000

miraculous a surprising 0.15640678208912500000000

miraculous a heavy 0.13033898507427100000000

miraculous a remarkable 0.07820339104456230000000

miraculous a wonderful 0.06818127553654730000000

mettle n fire 0.00235173072683178000000

mettle n heart 0.00014698317042698600000

mettle n temper 0.00014698317042698600000

mettle n resolution 0.00014698317042698600000

mettle n constitution 0.00014698317042698600000

mettle n inclination 0.00029396634085397200000

mettle n will 0.00029396634085397200000

mettle n blood 0.00073491585213492900000

mettle n force 0.00382156243110164000000

mettle n bias 0.00088189902256191700000

mettle n life 0.00014698317042698600000

mettle n physical 0.00044094951128095700000

mettle n courage 0.99044609392224600000000

lynching n execution 1.00000000000000000000000

sight n raft 0.00024922740687870100000

sight n slew 0.00033982021935502100000

sight n mess 0.00047576096115554700000

sight n deal 0.19963036845554800000000

sight n visibility 0.00020753779681644900000

sight n wonder 0.00885052045117860000000

sight n show 0.00450805393187000000000

sight n glance 0.28938071132026900000000

sight n mass 0.00319897496049463000000

sight n mountain 0.00009699550861727380000

sight n scene 0.00171196721954004000000

sight n vision 0.23766368967457600000000

sight n inspection 0.00015593181838235600000

sight n appearance 0.00226570369889728000000

sight n look 0.00025267141481868200000

sight n lot 0.09133714738256280000000

sight n perception 0.00569488647667738000000

sight n display 0.00006917926560548180000

sight n plenty 0.00005270446758212490000

sight n representation 0.00003959739712366450000

sight n examination 0.00039958207590390700000

sight n landmark 0.00053134200774674500000

sight n batch 0.00042859561637473500000

sight n view 0.15049407919771200000000

sight n survey 0.00140450395200193000000

sight n marvel 0.00025775908174826400000

sight n eyesore 0.00006917926560548180000

sight n monument 0.00023350897495721500000

joint v cut 1.00000000000000000000000

legislator n representative 0.01788020207012640000000

legislator n politician 0.17330854145805400000000

legislator n assemblyman 0.01665416079917980000000

legislator n senator 0.06492170651843910000000

legislator n lawmaker 0.56947417529488300000000

legislator n deputy 0.12395035473846400000000

legislator n congressman 0.03381085912085320000000

interrupted a irregular 0.62121726927572000000000

interrupted a broken 0.37878273072428000000000

instill v instil 0.72222222222222200000000

instill v inject 0.27777777777777800000000

hostess n owner 0.90909090909090900000000

hostess n manager 0.09090909090909070000000

rigour n rigor 1.00000000000000000000000

rebound v rally 0.62203600194613900000000

rebound v bound 0.01248763760357970000000

rebound v repeat 0.00004295858727584780000

rebound v bounce 0.31318633414907200000000

rebound v return 0.02484079674378060000000

rebound v reflect 0.02740627097015300000000

chicago n newmarket 0.00012597970190242600000

chicago n michigan 0.18837776736406500000000

chicago n chi 0.81149625293403200000000

intent n plan 0.50266456903279600000000

intent n purpose 0.00514272464323053000000

intent n aim 0.06861543440132010000000

intent n intention 0.14047704854136100000000

intent n objective 0.10028763628539600000000

intent n design 0.00003294782601093620000

intent n spirit 0.07958958192545240000000

intent n desire 0.00060472371051155500000

intent n motive 0.01509315939577910000000

intent n object 0.00108398732668222000000

intent n goal 0.08640818691146100000000

filibuster n opposition 1.00000000000000000000000

definitive a last 0.04712282208031740000000

definitive a conclusive 0.00084581517433440100000

definitive a clear-cut 0.00001433605239748970000

definitive a specific 0.03890682294034480000000

definitive a actual 0.01011639517222320000000

definitive a terminal 0.00001961350796691630000

definitive a complete 0.00388689582380914000000

definitive a exhaustive 0.00000358401309937235000

definitive a final 0.71611591259499600000000

definitive a exact 0.00390882879703891000000

definitive a absolute 0.00209482955940041000000

definitive a decisive 0.00003672975908355870000

definitive a reliable 0.00005457635115890110000

definitive a perfect 0.00010009854738760900000

definitive a real 0.00091963875516889000000

definitive a precise 0.01727970993988650000000

definitive a accurate 0.00097033372965354800000

definitive a positive 0.00913247079615374000000

definitive a ultimate 0.00128707842217282000000

definitive a best 0.00644960673896962000000

definitive a classic 0.00000252452057592065000

definitive a thorough 0.00000431652712560682000

definitive a definite 0.14072706019673600000000

expediency n opportunism 0.41666666666666700000000

expediency n interest 0.58333333333333300000000

evasive a ambiguous 0.53247763516141600000000

evasive a unclear 0.46752236483858400000000

epitomise v epitomize 1.00000000000000000000000

enquire v investigate 1.00000000000000000000000

e-mail v email 1.00000000000000000000000

doubly r again 1.00000000000000000000000

deactivate v inactivate 1.00000000000000000000000

modify v limit 0.00025684878710304100000

modify v revise 0.02559157215380570000000

modify v mitigate 0.00000783676257418578000

modify v reorganize 0.00000582830361923116000

modify v lower 0.01260095840433530000000

modify v reshape 0.00001848822610010440000

modify v adjust 0.03042732436063120000000

modify v qualify 0.00004989440193683340000

modify v reconstruct 0.00003472481603195930000

modify v blunt 0.00001944379259016640000

modify v remodel 0.00017339240531259400000

modify v convert 0.00034674967324260700000

modify v narrow 0.00017217951674595600000

modify v amend 0.43022724435828700000000

modify v curb 0.00026894668673898800000

modify v trim 0.00007321038118931540000

modify v rework 0.00006424565943403010000

modify v restrict 0.00021511887324182400000

modify v decrease 0.00072244387072458500000

modify v balance 0.00013184451904845500000

modify v subdue 0.00008377680646631780000

modify v reform 0.00039922458162272900000

modify v relax 0.00505530180979498000000

modify v recast 0.00000377026744179471000

modify v change 0.09963199180141790000000

modify v soften 0.00006257623871705580000

modify v alter 0.35463182897912000000000

modify v vary 0.00201910292324808000000

modify v reduce 0.01318776237411660000000

modify v transform 0.01074007256985590000000

modify v doctor 0.00002033247566195560000

modify v refine 0.00000105558301830990000

modify v moderate 0.00063534164015308000000

modify v turn 0.01146564101797670000000

modify v diminish 0.00024244186440807800000

modify v adapt 0.00008598447361002480000

modify v restrain 0.00009902288942234200000

modify v revamp 0.00007823106149040750000

modify v accommodate 0.00013864611870402100000

modify v temper 0.00000959857106102586000

conclave n meeting 0.97032435894561900000000

conclave n conference 0.00890394551061546000000

conclave n council 0.00081457629583392700000

conclave n congress 0.01995711924793120000000

compliant a gentle 0.12409893992932900000000

compliant a flexible 0.87590106007067100000000

coin v make 0.50000000000000000000000

coin v die 0.50000000000000000000000

dip n drop 0.25052568878447200000000

dip n sinking 0.00000684741045932295000

dip n reduction 0.00408920636329997000000

dip n solution 0.00227034954648624000000

dip n inclination 0.00003137291493689170000

dip n preparation 0.00001873390476256440000

dip n suspension 0.02153173222524130000000

dip n slump 0.01587918252519260000000

dip n decrease 0.04347719543791920000000

dip n downturn 0.02344968929446480000000

dip n basin 0.00000159148093739468000

dip n plunge 0.00679814353898861000000

dip n fall 0.35399225752726400000000

dip n dilution 0.00012563282428209900000

dip n depression 0.00027664714889470200000

dip n spread 0.02239419452926270000000

dip n slope 0.00002240542300109860000

dip n decline 0.19612364241536900000000

dip n slip 0.05256811771020430000000

dip n dive 0.00641736899456013000000

revengeful a vengeful 1.00000000000000000000000

bland a flat 0.00253229950692709000000

bland a thin 0.00062028045577125000000

bland a indifferent 0.00015507011394281100000

bland a mediocre 0.00015507011394281100000

bland a soft 0.11060281322023600000000

bland a ordinary 0.00063307487673177200000

bland a boring 0.00253229950692709000000

bland a tender 0.00015507011394281100000

bland a calm 0.13098451535396900000000

bland a dull 0.51951933169971400000000

bland a cool 0.00521966912779699000000

bland a so-so 0.00189922463019532000000

bland a clear 0.00315257996269834000000

bland a gray 0.00063307487673177200000

bland a weak 0.22120562644047300000000

audible a clear 1.00000000000000000000000

apartment n accommodation 0.02003075393091300000000

apartment n condominium 0.00820463915966248000000

apartment n room 0.15778611429789400000000

apartment n residence 0.00157265290184722000000

apartment n tenement 0.00011923067816019700000

apartment n home 0.32661274149660800000000

apartment n efficiency 0.00178170643466018000000

apartment n apartments 0.04006150786182610000000

apartment n townhouse 0.00035769203448059100000

apartment n cell 0.00011923067816019700000

apartment n flat 0.32326779473481700000000

apartment n story 0.00092817243537764700000

apartment n rent 0.00000750075039539899000

apartment n studio 0.00005464588826256930000

apartment n suite 0.01037090116942510000000

apartment n lodgings 0.04006150786182610000000

apartment n housing 0.06866320768568480000000

must v should 0.98594007491068200000000

must v ought 0.01405992508931810000000

firmly r decisively 0.00142472224202040000000

firmly r solidly 0.07800354275061690000000

firmly r strongly 0.43522817151536900000000

firmly r steadfastly 0.17680803023473200000000

firmly r resolutely 0.04209715005588850000000

firmly r adamantly 0.07800354275061700000000

firmly r substantially 0.09100413320905310000000

firmly r fast 0.00015779616776081000000

firmly r steadily 0.00035618056050510000000

firmly r strictly 0.09171649433006330000000

firmly r tightly 0.00520023618337446000000

interior a central 0.59137686835247500000000

interior a local 0.11657299811183700000000

interior a domestic 0.28989097842670300000000

interior a secret 0.00062927394392354800000

interior a private 0.00121524419309974000000

interior a confidential 0.00031463697196177400000

well-balanced a moderate 0.20801524820944900000000

well-balanced a mixed 0.14748962842668400000000

well-balanced a reasonable 0.00326590588449488000000

well-balanced a balanced 0.05641852415464910000000

well-balanced a sound 0.45134819323719200000000

well-balanced a prudent 0.00326590588449488000000

well-balanced a orderly 0.13019659420303600000000

drain v draw 0.00007189767367538680000

drain v suck 0.04661365843287580000000

drain v dissipate 0.00001353245194615970000

drain v filter 0.00001353245194615970000

drain v sap 0.05593639011945100000000

drain v siphon 0.05966548279408100000000

drain v remove 0.00010825961556927700000

drain v withdraw 0.13523922440153200000000

drain v surge 0.00532727524947153000000

drain v flow 0.54170923277136900000000

drain v rush 0.00000470884671432367000

drain v dump 0.00015624689977500300000

drain v evacuate 0.00002706490389231930000

drain v draft 0.00002706490389231930000

drain v run 0.00960294741493630000000

drain v reduce 0.03454485101053040000000

drain v tax 0.00329037588937947000000

drain v decline 0.00013698707784355300000

drain v divert 0.02796819505972550000000

drain v leak 0.00015624689977500300000

drain v dry 0.02237455604778040000000

drain v diminish 0.00014933427075887300000

drain v drop 0.00041674441867291700000

drain v strain 0.02836073113073920000000

drain v exhaust 0.00007189767367538680000

drain v deprive 0.00000941769342864733000

drain v deplete 0.02800414389656320000000

junior a second 0.00046531721075325900000

junior a lower 0.00165664768753759000000

junior a subordinate 0.31115338867424300000000

junior a minor 0.26967439910039400000000

junior a low-ranking 0.18156086444008500000000

junior a younger 0.23548938288698700000000

traumatise v shock 1.00000000000000000000000

intense a concentrated 0.00011641024796907200000

intense a upset 0.00023860691389834100000

intense a sharp 0.03706301024509320000000

intense a heightened 0.03908321431799220000000

intense a impetuous 0.00001608563863268430000

intense a great 0.02209705847343470000000

intense a serious 0.01706649884729230000000

intense a exaggerated 0.00005793166074955050000

intense a hard 0.00385655525523985000000

intense a profound 0.00003409568574985820000

intense a extraordinary 0.00160031215309769000000

intense a supreme 0.00000659585472863215000

intense a rigorous 0.00015210271643594500000

intense a shocking 0.00018949861666491600000

intense a stubborn 0.00004387360037890030000

intense a frantic 0.00000088426530644870400

intense a potent 0.00025454091029975700000

intense a cruel 0.00044089044909979700000

intense a strained 0.00002880324415940890000

intense a hasty 0.00000265279591934616000

intense a high 0.03418704005689030000000

intense a energetic 0.00026530448570235000000

intense a responsive 0.00066215845414262200000

intense a passionate 0.00004298933507245160000

intense a strong 0.30575955907572100000000

intense a powerful 0.00880369956640114000000

intense a forceful 0.00057727319209923100000

intense a acrimonious 0.00000088426530644870400

intense a critical 0.00247288242103757000000

intense a malicious 0.00000088426530644870400

intense a volatile 0.01168095835533120000000

intense a aggressive 0.02767197962487380000000

intense a severe 0.17692656761914500000000

intense a ecstatic 0.00006969940991484030000

intense a feverish 0.00002523500426354410000

intense a impatient 0.00002242963057622580000

intense a vivid 0.00002857691670433240000

intense a exceptional 0.00209480630570866000000

intense a resolute 0.00007970078514574810000

intense a deep 0.00026812313719890500000

intense a sensitive 0.04411183786362410000000

intense a pointed 0.00032425692978436600000

intense a keen 0.00861628825504018000000

intense a touchy 0.00000659585472863215000

intense a nervous 0.00217484552368006000000

intense a violent 0.00163350711640250000000

intense a grievous 0.00001412319071949680000

intense a full 0.00331661531638824000000

intense a poignant 0.00032425692978436600000

intense a excessive 0.00056916729416554700000

intense a hostile 0.00002509315602458880000

intense a bitter 0.01357375497536150000000

intense a rich 0.00007786215703373070000

intense a busy 0.00139475748304708000000

intense a fierce 0.07649753662779350000000

intense a angry 0.00123595521643299000000

intense a ambitious 0.00063502328812441100000

intense a mad 0.00003773621329979590000

intense a brisk 0.00129250551079171000000

intense a distressing 0.00006386423969471630000

intense a eager 0.00103029400464314000000

intense a dark 0.00002463851053974960000

intense a extreme 0.01263246799754880000000

intense a diligent 0.00071092086345078000000

intense a warm 0.00005046945510753240000

intense a wild 0.00032667935406780200000

intense a rapturous 0.00022044522454989900000

intense a bright 0.00202015996492019000000

intense a harsh 0.00022132948985634700000

intense a tense 0.00252280051149007000000

intense a intensive 0.02167871168573010000000

intense a touching 0.00003561785825572260000

intense a committed 0.00004515490607230870000

intense a dynamic 0.00014007048650515500000

intense a drastic 0.00714622299508499000000

intense a emotional 0.02165589725440210000000

intense a painful 0.00082058517746408800000

intense a acute 0.04367663079039170000000

intense a marked 0.00044936730288845600000

intense a grave 0.00658287971929953000000

intense a enthusiastic 0.00372485717926998000000

intense a active 0.01579779933980890000000

intense a brilliant 0.00011409290520583700000

intense a moving 0.00009063010087377660000

intense a vigorous 0.00003409568574985820000

intense a heated 0.00371196105459092000000

intense a harmful 0.00023323093642367200000

intense a excited 0.00438603230520208000000

harvest v amass 0.00004107328050811390000

harvest v pick 0.00124448500245650000000

harvest v earn 0.00046164571861111200000

harvest v get 0.00430040297236838000000

harvest v procure 0.03440724520086670000000

harvest v secure 0.01550678648022830000000

harvest v obtain 0.00335545890184043000000

harvest v cull 0.00003120136357363610000

harvest v glean 0.00009085436049952590000

harvest v gather 0.15056544605014200000000

harvest v collect 0.17891275162981900000000

harvest v make 0.29320905883051700000000

harvest v accumulate 0.00021779670453930700000

harvest v cut 0.21992744153433700000000

harvest v sack 0.00129666679352928000000

harvest v store 0.00990182193722404000000

harvest v reap 0.08507852707475130000000

harvest v net 0.00004557854943322860000

harvest v acquire 0.00128808524596532000000

harvest v garner 0.00011767236879088200000

ruble n rouble 1.00000000000000000000000

psyche n mind 0.94609164420485200000000

psyche n reason 0.05390835579514820000000

belief n guess 0.01024965387968230000000

belief n affiliation 0.00000184818871995581000

belief n sentiment 0.04331554036282350000000

belief n ideology 0.00000508816747841142000

belief n tenet 0.00003963279600426200000

belief n hope 0.02007624467755950000000

belief n opinion 0.01577486208934650000000

belief n catechism 0.00000165583489766716000

belief n idea 0.06943629505967380000000

belief n viewpoint 0.02397700418955690000000

belief n doctrine 0.00000184818871995581000

belief n security 0.04339041095138490000000

belief n apprehension 0.00000165583489766716000

belief n principle 0.05398420035572570000000

belief n concession 0.00021492876498336200000

belief n experience 0.00116992905052670000000

belief n hypothesis 0.00013738655801632100000

belief n presumption 0.00095239599647109400000

belief n mind 0.00026970760616947900000

belief n dogma 0.00140777621968872000000

belief n mindset 0.00006961240276496730000

belief n declaration 0.00018255531819838300000

belief n conclusion 0.00183037204253410000000

belief n inference 0.00001393837841275960000

belief n feeling 0.02354482423099840000000

belief n credit 0.00069171645266976100000

belief n understanding 0.00235072459029045000000

belief n attitude 0.00054698630238727600000

belief n position 0.01337792874594970000000

belief n acceptance 0.00000496750469300148000

belief n assurance 0.00067244796472473500000

belief n permission 0.00004646428974639990000

belief n reliance 0.00000350402361762297000

belief n compliance 0.00011414559158254700000

belief n judgment 0.00153850228717875000000

belief n suspicion 0.03963723359551690000000

belief n fancy 0.00002886585072032340000

belief n perception 0.04362854138733350000000

belief n concept 0.00049013269718695900000

belief n assumption 0.02043983867261790000000

belief n faith 0.00022624956001004400000

belief n knowledge 0.00023621262519165800000

belief n rule 0.00103426107094070000000

belief n conjecture 0.00001336434216652700000

belief n conviction 0.07352386899016870000000

belief n theory 0.00157130453241526000000

belief n impression 0.00505478709543565000000

belief n admission 0.00004646428974639990000

belief n expectation 0.16654425755696500000000

belief n credo 0.00001545756254167420000

belief n religion 0.02404407713916770000000

belief n judgement 0.00046050290464094500000

belief n supposition 0.00002672868433305390000

belief n thinking 0.00005173872273148040000

belief n denomination 0.00005314576483320950000

belief n view 0.20699662093258700000000

belief n trust 0.00080102757266013400000

belief n cult 0.00000099894378523250400

belief n premise 0.00058729314686400500000

belief n certainty 0.00000899049406709268000

belief n notion 0.00031792732740923700000

belief n confidence 0.08476335363988770000000

range n ridge 0.00281326002592404000000

range n diversity 0.00022197824342499800000

range n sphere 0.00024483329838924300000

range n run 0.00488994674555466000000

range n limit 0.02702363256118440000000

range n race 0.01262016876501350000000

range n span 0.00028523040293286700000

range n pasture 0.00121215631308902000000

range n choice 0.00235130638024267000000

range n compass 0.00003625238577315650000

range n class 0.00355292394065358000000

range n sequence 0.00000286848828634285000

range n kind 0.00330503491114690000000

range n chain 0.00663603239542026000000

range n mead 0.00001274838789544020000

range n row 0.00578834333536716000000

range n grasp 0.00001241579655885180000

range n area 0.41508028148501800000000

range n pastureland 0.00000538628598046836000

range n image 0.00031593049655834800000

range n scale 0.02593775241599950000000

range n extent 0.00153968892192093000000

range n lea 0.00010972822112543400000

range n order 0.01488481367113280000000

range n assortment 0.00168322892642599000000

range n variety 0.02838215155468350000000

range n sort 0.00057290791291839000000

range n line 0.15831062927490000000000

range n collection 0.00168415747630419000000

range n rank 0.00057636780914922700000

range n scope 0.00303957162707870000000

range n string 0.00013358959733539600000

range n selection 0.00024718520626334000000

range n lot 0.05673065667977750000000

range n sierra 0.00012947928464134000000

range n place 0.02009034874351420000000

range n file 0.00003350133714978180000

range n horizon 0.00630803588236871000000

range n series 0.02658190495666040000000

range n register 0.00030449781466789900000

range n gamut 0.00076510405746635800000

range n genus 0.00001409936277894230000

range n reach 0.01379827326835840000000

range n stretch 0.00058019718086838000000

range n spectrum 0.08226933536893450000000

range n species 0.00027119354376352400000

range n succession 0.00036620420264117000000

range n sweep 0.00004009711771748770000

range n tier 0.00015383056240549000000

range n plain 0.00004090489340654520000

range n margin 0.04303841514758680000000

range n orbit 0.00003781954532120180000

range n stride 0.00001010103671671090000

range n array 0.01829743652335190000000

range n grass 0.00060003362560242300000

range n field 0.00519032871511273000000

range n highlands 0.00066215808584707000000

range n queue 0.00001056188566585030000

range n mixture 0.00016297791802366900000

pedal n brake 1.00000000000000000000000

pay-off n result 0.00545483715706722000000

pay-off n conclusion 0.00128349109578052000000

pay-off n settlement 0.99326167174715200000000

coordinate v reconcile 0.00042402524352613700000

coordinate v file 0.00087024284412970800000

coordinate v place 0.00903133001096077000000

coordinate v organize 0.00001369940282528910000

coordinate v number 0.00070864758978334600000

coordinate v order 0.00143860487344835000000

coordinate v orchestrate 0.00228694272987538000000

coordinate v distinguish 0.00033094772579994600000

coordinate v combine 0.00234609728259958000000

coordinate v adjust 0.00062631020593990600000

coordinate v peg 0.00005479761130115720000

coordinate v rate 0.00110235381612842000000

coordinate v organise 0.73471110329962600000000

coordinate v agree 0.00356851511925970000000

coordinate v cooperate 0.00029734785506939400000

coordinate v balance 0.00232519778559080000000

coordinate v arrange 0.23309057254821700000000

coordinate v unite 0.00019202709309237000000

coordinate v regulate 0.00035172760107169500000

coordinate v fit 0.00436831705603782000000

coordinate v dispose 0.00002739880565057860000

coordinate v suit 0.00182009409724118000000

coordinate v classify 0.00001369940282528910000

incorporate v charter 0.00001850773435728650000

incorporate v express 0.03448882107935520000000

incorporate v link 0.00921006188966135000000

incorporate v combine 0.01410462161323900000000

incorporate v identify 0.00182246121015741000000

incorporate v contain 0.13903944355723300000000

incorporate v unify 0.00000477180554875977000

incorporate v merge 0.00247958065591408000000

incorporate v start 0.00171645262633998000000

incorporate v consolidate 0.00004294624993883830000

incorporate v offer 0.04827479587615530000000

incorporate v associate 0.00002747185761705350000

incorporate v assimilate 0.00000477180554875977000

incorporate v mix 0.00018610041640163300000

incorporate v integrate 0.11134552154960000000000

incorporate v absorb 0.21415587920896100000000

incorporate v unite 0.00117894185551475000000

incorporate v amalgamate 0.00000477180554875977000

incorporate v join 0.00034884857131832900000

incorporate v embrace 0.00867577063409086000000

incorporate v include 0.29372671427867000000000

incorporate v form 0.11914274371882800000000

menage n household 0.00041902372567373700000

menage n house 0.01061672217651480000000

menage n home 0.98645011174376900000000

menage n family 0.00251414235404247000000

rare a strange 0.00087249685312947300000

rare a occasional 0.00363296038552351000000

rare a great 0.08489975768089260000000

rare a odd 0.00010812564942117800000

rare a superb 0.00003870934906478570000

rare a light 0.04096416878130680000000

rare a extraordinary 0.00809955313249022000000

rare a phenomenal 0.00009146329556145890000

rare a thin 0.04728282752515720000000

rare a limited 0.02638262675532180000000

rare a individual 0.00882897163769711000000

rare a curious 0.00024432944587797200000

rare a sparse 0.00107750941313222000000

rare a original 0.00505212588082796000000

rare a volatile 0.02100088119709990000000

rare a uncommon 0.04046246253599180000000

rare a expensive 0.01543174337282010000000

rare a exceptional 0.00029425072280468400000

rare a unfamiliar 0.00001000836420513610000

rare a tenuous 0.00313025714497448000000

rare a excellent 0.00022157179457320700000

rare a precious 0.01480767161232200000000

rare a deficient 0.00005285802398559590000

rare a priceless 0.07992512848151730000000

rare a noteworthy 0.00001935467453239290000

rare a invaluable 0.00001437715665238470000

rare a peculiar 0.00010812564942117800000

rare a exclusive 0.00001501254630770430000

rare a rich 0.00198463764016871000000

rare a few 0.14407947976370400000000

rare a fine 0.00266825226225100000000

rare a unusual 0.25170951232977300000000

rare a scanty 0.00011780298668737400000

rare a atypical 0.00000967733726619643000

rare a infrequent 0.00201110489898111000000

rare a anomalous 0.00000234990969309408000

rare a bloody 0.00001366568554551790000

rare a unprecedented 0.04806086325202160000000

rare a unique 0.04322868204038730000000

rare a short 0.01185735312825110000000

rare a outstanding 0.00047337515175419400000

rare a valuable 0.01342140053618450000000

rare a special 0.02106040330486480000000

rare a superior 0.00002733137109103590000

rare a isolated 0.00219608196897813000000

rare a sporadic 0.02164747674814980000000

rare a remarkable 0.00012822213125377100000

rare a scattered 0.00255486092140220000000

rare a scarce 0.00471376150889982000000

rare a notable 0.02441933303138330000000

rare a scant 0.00018980173299738000000

rare a undercooked 0.00001935467453239290000

rare a meager 0.00008462162888461330000

rare a wonderful 0.00025126499228175000000

luster n lustre 0.98812137792016100000000

luster n oil 0.01187862207983870000000

litigant n prosecutor 0.26465407980196500000000

litigant n plaintiff 0.24412725117342300000000

litigant n party 0.49121866902461200000000

leaving n going 0.08129809300769460000000

leaving n departure 0.91870190699230600000000

jealousy n insecurity 1.00000000000000000000000

itch v annoy 1.00000000000000000000000

notably r particularly 0.31753266652933500000000

notably r especially 0.44174099600134400000000

notably r very 0.04915297221928200000000

notably r significantly 0.00350470081771752000000

notably r primarily 0.09357730407316470000000

notably r obviously 0.00082857285604597500000

notably r conspicuously 0.05263723354115520000000

notably r greatly 0.02807319122194940000000

notably r principally 0.01220573531389110000000

notably r exceptionally 0.00074662742611567600000

incurable a terminal 0.45795601552393300000000

incurable a fatal 0.23673997412677900000000

incurable a deadly 0.30530401034928900000000

immigrate v arrive 0.20601700457815600000000

immigrate v migrate 0.19097449313276700000000

immigrate v emigrate 0.36625245258338800000000

immigrate v move 0.23675604970569000000000

hq n headquarters 1.00000000000000000000000

chief a highest 0.01390150097249350000000

chief a primary 0.00795311575879131000000

chief a necessary 0.00122400005555985000000

chief a star 0.00879084463579122000000

chief a central 0.00412280330569270000000

chief a foremost 0.00040192059472517900000

chief a indispensable 0.00000040414219509586200

chief a major 0.06991447639803300000000

chief a leading 0.00155457144316913000000

chief a crucial 0.00112096321830765000000

chief a first 0.04421719863475900000000

chief a vital 0.00003164503067289470000

chief a principal 0.10061378234416500000000

chief a essential 0.00001027891542756770000

chief a main 0.44415815607063400000000

chief a key 0.16414177833098100000000

chief a grand 0.00000779675799183252000

chief a premier 0.00000040414219509586200

chief a outstanding 0.00147384196223304000000

chief a superior 0.00006531008158848960000

chief a prime 0.13560287267523400000000

chief a paramount 0.00000080828439019173800

chief a significant 0.00069152624496842000000

dairy n cooperative 1.00000000000000000000000

gracious a civil 0.25855979403559100000000

gracious a charitable 0.14981335601494200000000

gracious a pleasant 0.00926098093364027000000

gracious a lenient 0.03090676597529650000000

gracious a obliging 0.00231524523341006000000

gracious a cordial 0.00694573570023021000000

gracious a diplomatic 0.09514128517022270000000

gracious a polite 0.02849365803906440000000

gracious a polished 0.00734506581961933000000

gracious a easy 0.01915775727478300000000

gracious a kind 0.03312414850588450000000

gracious a comfortable 0.08515556924634780000000

gracious a friendly 0.01429080151984950000000

gracious a thoughtful 0.00231524523341006000000

gracious a compassionate 0.00734506581961933000000

gracious a mild 0.00152650269681909000000

gracious a forthcoming 0.11406707343537200000000

gracious a refined 0.00734506581961933000000

gracious a familiar 0.00966031105302940000000

gracious a warm 0.00463049046682012000000

gracious a nice 0.04083468928842740000000

gracious a benign 0.07176539271800220000000

boom n improvement 0.02232312750420110000000

boom n growth 0.45149850595713000000000

boom n addition 0.00504763824306264000000

boom n upsurge 0.00349408018926149000000

boom n advance 0.00194493026072129000000

boom n bonanza 0.00002087585119825240000

boom n shot 0.00048232737034941600000

boom n rise 0.11098648436633800000000

boom n development 0.07395932018087200000000

boom n explosion 0.05877867090655200000000

boom n boost 0.00350200211796520000000

boom n gain 0.07554698200491730000000

boom n inflation 0.05864260778053050000000

boom n increase 0.01421535073187770000000

boom n rumbling 0.00003024554162180180000

boom n blast 0.00016616533814468700000

boom n windfall 0.00000612548754874172000

boom n upturn 0.03770596318960210000000

boom n rush 0.00036600807277275100000

boom n prosperity 0.00452112673062624000000

boom n bang 0.00033536612179672200000

boom n expansion 0.01981808615204350000000

boom n upswing 0.02605739709748230000000

boom n report 0.03055061280338390000000

comprehensive a pregnant 0.00000916470406565403000

comprehensive a wise 0.00001221960542087200000

comprehensive a far-reaching 0.00062562382672455100000

comprehensive a intelligent 0.00133374215985769000000

comprehensive a whole 0.00072916246550478700000

comprehensive a substantial 0.00162362588786297000000

comprehensive a broad 0.15930034846982900000000

comprehensive a wide 0.00127491588006601000000

comprehensive a complete 0.00036116328021095600000

comprehensive a widespread 0.01126849386964950000000

comprehensive a general 0.56874023778952700000000

comprehensive a exhaustive 0.00000610980271043592000

comprehensive a aware 0.00007026273117001450000

comprehensive a radical 0.00509246060568741000000

comprehensive a full 0.05526831694224370000000

comprehensive a detailed 0.03772181164013640000000

comprehensive a extensive 0.00580296087629094000000

comprehensive a capable 0.00000610980271043592000

comprehensive a absolute 0.04271121228508550000000

comprehensive a ample 0.00010505168219314400000

comprehensive a large 0.00064623489102649400000

comprehensive a catholic 0.00000305490135521796000

comprehensive a infinite 0.00000305490135521796000

comprehensive a sweeping 0.04934215034973440000000

comprehensive a discerning 0.00007615795964374160000

comprehensive a able 0.00002964593505999970000

comprehensive a global 0.00108452214855966000000

comprehensive a overall 0.04092892288049550000000

comprehensive a significant 0.00148702083799809000000

comprehensive a universal 0.00045883692627691300000

comprehensive a wide-ranging 0.01387219723732970000000

comprehensive a thorough 0.00000520672421825555000

reformable a redeemable 1.00000000000000000000000

symbol n letter 0.00072945537090828300000

symbol n crest 0.00000853264034022157000

symbol n logo 0.00518444482068053000000

symbol n representative 0.00052149626699652600000

symbol n type 0.00017078073507406400000

symbol n number 0.01321821946110740000000

symbol n mark 0.01101621608220470000000

symbol n brand 0.12349296363550900000000

symbol n figure 0.01927207395174850000000

symbol n feature 0.00009361132192610840000

symbol n emblem 0.08052937885942940000000

symbol n image 0.00531319859848056000000

symbol n badge 0.00001052440244810950000

symbol n device 0.00005005451975643330000

symbol n flag 0.06245593739928840000000

symbol n code 0.00034130230930195800000

symbol n note 0.00537198275221605000000

symbol n banner 0.00000284421344674048000

symbol n chevron 0.00006045591807602500000

symbol n ensign 0.00000284421344674048000

symbol n signature 0.00000284421344674048000

symbol n stamp 0.00017739913010535200000

symbol n trademark 0.00330376938910480000000

symbol n standard 0.02580077000638980000000

symbol n sign 0.64243324136721300000000

symbol n seal 0.00043565842135406500000

elastic a tolerant 0.05295400758203290000000

elastic a volatile 0.22348452431788000000000

elastic a flexible 0.65893896652830500000000

elastic a variable 0.06462250157178160000000

dismember v injure 0.70788007429026300000000

dismember v mutilate 0.13823295303794100000000

dismember v maim 0.15388697267179600000000

islands n archipelago 1.00000000000000000000000

discontinuance n delay 0.06461439426772550000000

discontinuance n break 0.03230719713386270000000

discontinuance n suspension 0.01132934329587870000000

discontinuance n disruption 0.03230719713386270000000

discontinuance n termination 0.82713467103480800000000

discontinuance n pause 0.03230719713386270000000

despicable a ugly 0.07317073170731700000000

despicable a awful 0.19512195121951200000000

despicable a miserable 0.02439024390243900000000

despicable a worthless 0.04878048780487800000000

despicable a shameful 0.07317073170731700000000

despicable a cheap 0.07317073170731700000000

despicable a distasteful 0.02439024390243900000000

despicable a corrupt 0.02439024390243900000000

despicable a sorry 0.46341463414634200000000

decor n furnishings 1.00000000000000000000000

watch n observation 0.00026023067708713700000

watch n study 0.00253258393313710000000

watch n picket 0.00009435754683125680000

watch n attention 0.00063210797058798100000

watch n vigilance 0.14175627560926900000000

watch n guard 0.00283465173679388000000

watch n consideration 0.01036554999255610000000

watch n watching 0.15924051009936500000000

watch n wariness 0.00003573920832641080000

watch n wake 0.00037548048243586800000

watch n charge 0.00639636683790499000000

watch n inspection 0.00007092233882763430000

watch n monitoring 0.00002970331069987150000

watch n custody 0.00250969179429013000000

watch n supervision 0.10635094917731700000000

watch n wristwatch 0.04245460557094350000000

watch n regard 0.00003883794790348470000

watch n surveillance 0.14346517727782900000000

watch n clock 0.00005448795453439860000

watch n examination 0.00514287940835042000000

watch n timepiece 0.14151535190314500000000

watch n scrutiny 0.21256881258327700000000

watch n view 0.01909501631963270000000

watch n survey 0.00217971031895475000000

categorise v categorize 1.00000000000000000000000

shutdown n closing 0.03206101577618630000000

shutdown n closedown 0.00002488587279027820000

shutdown n closure 0.96791409835102300000000

discipline n regulation 0.05622984222551270000000

discipline n organization 0.00047041752313821700000

discipline n study 0.00040682477867105000000

discipline n course 0.00884493379712764000000

discipline n direction 0.00150970358459445000000

discipline n major 0.00000928542835878296000

discipline n government 0.07809407258095650000000

discipline n rigor 0.36837733399402800000000

discipline n system 0.06390763297885360000000

discipline n penalty 0.00002931314254662800000

discipline n practice 0.00011896276951749200000

discipline n correction 0.00209797797172823000000

discipline n procedure 0.00001582528376511880000

discipline n training 0.00181945935318828000000

discipline n restriction 0.04570181858900870000000

discipline n area 0.01351291697897300000000

discipline n development 0.00030322878939581200000

discipline n subject 0.00346730738335118000000

discipline n method 0.00004441248430191450000

discipline n coercion 0.00001230856502105730000

discipline n preparation 0.00013756576220709600000

discipline n order 0.05296645570208610000000

discipline n exercise 0.00006043846806678250000

discipline n drilling 0.00001031982228981790000

discipline n check 0.00056223039008169200000

discipline n authority 0.02260652935188480000000

discipline n compulsion 0.00006588051236945290000

discipline n rule 0.04226271670735380000000

discipline n control 0.01763554056459240000000

discipline n punishment 0.00000168155831426149000

discipline n instruction 0.00000661597199110674000

discipline n restraint 0.21847834820238200000000

discipline n speciality 0.00006384458528355060000

discipline n drill 0.00001375673052878560000

discipline n limitation 0.00002599092471865830000

discipline n field 0.00012850654381080100000

blot n blemish 1.00000000000000000000000

auspicious a rosy 0.04869271054922250000000

auspicious a right 0.07498989557340190000000

auspicious a successful 0.04869271054922250000000

auspicious a fortunate 0.02434635527461120000000

auspicious a optimistic 0.15825130928497300000000

auspicious a good 0.27190448290620600000000

auspicious a happy 0.00608658881865280000000

auspicious a favourable 0.04260612173056970000000

auspicious a encouraging 0.04869271054922250000000

auspicious a favorable 0.02010038438049950000000

auspicious a promising 0.03043294409326400000000

auspicious a hopeful 0.00608658881865280000000

auspicious a bright 0.01825976645595840000000

auspicious a clear 0.20085743101554300000000

amplifier n speaker 0.50000000000000200000000

amplifier n mike 0.16666666666666400000000

amplifier n horn 0.33333333333333300000000

agreeable a beautiful 0.00006157738449151860000

agreeable a ready 0.00132391376656768000000

agreeable a responsive 0.00003078869224575930000

agreeable a pleasant 0.00006157738449151860000

agreeable a satisfying 0.00003078869224575930000

agreeable a attractive 0.00024630953796607900000

agreeable a willing 0.00003078869224575930000

agreeable a good 0.00704245965605746000000

agreeable a appropriate 0.00113918161309312000000

agreeable a friendly 0.00009236607673727940000

agreeable a fine 0.00299845566860892000000

agreeable a acceptable 0.98537156953071600000000

agreeable a welcome 0.00101602684411008000000

agreeable a proper 0.00006157738449151860000

agreeable a tasty 0.00003078869224575930000

agreeable a nice 0.00006157738449151860000

agreeable a likeable 0.00003078869224575930000

agreeable a suitable 0.00024630953796607900000

agreeable a compatible 0.00012315476898303900000

aggressiveness n aggression 1.00000000000000000000000

incur v earn 0.06457028097463060000000

incur v get 0.42481501472974300000000

incur v welcome 0.00001726229797561740000

incur v obtain 0.00489366977352309000000

incur v assume 0.00032798366153673700000

incur v invite 0.00029820890432490600000

incur v sustain 0.04261031135325340000000

incur v experience 0.34998911373931400000000

incur v find 0.02133392268199380000000

incur v contract 0.00180689548962460000000

incur v receive 0.01174385070702370000000

incur v acquire 0.07759348568705620000000

afghan n afghani 1.00000000000000000000000

wrench n blow 0.33333333333333300000000

wrench n pain 0.66666666666666700000000

bind v constrain 0.00509873294396004000000

bind v compel 0.00069528176508546000000

bind v inhibit 0.00000351159735261772000

bind v set 0.15333133091251100000000

bind v cinch 0.00000351159735261772000

bind v secure 0.00002277475687998570000

bind v treat 0.00079284550476643500000

bind v press 0.00684462435361111000000

bind v bond 0.00001575156217475020000

bind v tie 0.04260566828290530000000

bind v handcuff 0.00000702319470523552000

bind v stick 0.27242998946949700000000

bind v detain 0.00446906509153292000000

bind v engage 0.00001575156217475020000

bind v trim 0.00055341613460889600000

bind v swathe 0.00001575156217475020000

bind v restrict 0.00003852631905473580000

bind v connect 0.00000877899338154447000

bind v unite 0.00214911362978916000000

bind v dress 0.00008549704631640310000

bind v enforce 0.00157697582153632000000

bind v join 0.05751549082117310000000

bind v oblige 0.11217212476712100000000

bind v force 0.00281619240795599000000

bind v confine 0.07012508534030160000000

bind v restrain 0.04971615780096310000000

bind v cover 0.04368987923971870000000

bind v hinder 0.00000526739602892666000

bind v require 0.11463588336650000000000

bind v hold 0.03149632083514300000000

bind v adhere 0.00002628635423260350000

bind v fix 0.02703738956949130000000

vilify v attack 0.17610062893081800000000

vilify v denounce 0.82389937106918200000000

simple a unconditional 0.00016441060518435900000

simple a accessible 0.00001577991096126790000

simple a natural 0.00090554848307333900000

simple a direct 0.00106143176256731000000

simple a casual 0.00020244348017916800000

simple a plain 0.00796441666731842000000

simple a preliminary 0.00112012632468738000000

simple a stark 0.00561638133596336000000

simple a insignificant 0.00038225297299357800000

simple a absurd 0.00236383128005489000000

simple a shallow 0.00011605125670695300000

simple a unreasonable 0.00065401764935827300000

simple a manageable 0.00091875430709934700000

simple a stupid 0.00078872738102378200000

simple a equitable 0.00003699042517085170000

simple a frank 0.00057307800650719000000

simple a blunt 0.00224691000019980000000

simple a paltry 0.00016677572651075700000

simple a slow 0.08145660263866030000000

simple a understandable 0.00037478307982129800000

simple a sincere 0.00002366986644190220000

simple a straightforward 0.00196055712421502000000

simple a limited 0.01961566714878210000000

simple a mediocre 0.00002121051420958380000

simple a unproblematic 0.00000788995548063395000

simple a soft 0.00241442660208365000000

simple a ordinary 0.00029050586518005900000

simple a silly 0.00078988607800953000000

simple a modest 0.00789244242642027000000

simple a foolish 0.00051041796211572200000

simple a restrained 0.00093009706983302400000

simple a naive 0.00037437915310322800000

simple a fair 0.00498442496504878000000

simple a humble 0.00014074073874245700000

simple a basic 0.01456857190295090000000

simple a feeble 0.00012690406742349200000

simple a pointless 0.00003947284744843530000

simple a true 0.00647054685232529000000

simple a sole 0.00021002623158587900000

simple a fundamental 0.00207676509040034000000

simple a senseless 0.00042222221622737100000

simple a commonplace 0.00025380813484698400000

simple a easy 0.05897600745029790000000

simple a deficient 0.00001292778500340530000

simple a candid 0.00010122174008958400000

simple a green 0.00026799746660034100000

simple a unambiguous 0.00020778801853826000000

simple a average 0.00876129310471893000000

simple a underlying 0.00486090283975443000000

simple a ignorant 0.00293059858026808000000

simple a radical 0.00160925361986518000000

simple a illogical 0.00008956342498331740000

simple a low 0.18242861057513900000000

simple a square 0.00001577991096126790000

simple a trivial 0.00000788995548063395000

simple a controllable 0.00001577991096126790000

simple a common 0.00706155340967166000000

simple a mild 0.00202076811651769000000

simple a rudimentary 0.00019989806305762600000

simple a unimportant 0.00035069315107104200000

simple a dull 0.00645224841317783000000

simple a clean 0.00015181717212564400000

simple a absolute 0.20544553689452800000000

simple a obvious 0.00837096473107298000000

simple a childish 0.00014074073874245700000

simple a user-friendly 0.00001690125254178210000

simple a mean 0.00001903561011352380000

simple a innocent 0.00186586242247182000000

simple a evident 0.00076001280896788200000

simple a single 0.00043265324932719000000

simple a insufficient 0.00065279954489003200000

simple a mere 0.00360904264300318000000

simple a pure 0.00423179956365681000000

simple a clear 0.10487360878708400000000

simple a ultimate 0.00608910042957480000000

simple a isolated 0.00093572510434424800000

simple a transparent 0.00156563077470400000000

simple a genetic 0.00909068763580672000000

simple a decent 0.00094033299097296300000

simple a scarce 0.00071865592692165500000

simple a open 0.00249673449189861000000

simple a classic 0.00014074073874245700000

simple a only 0.14991115454790900000000

simple a honest 0.00515475219560265000000

simple a unaffected 0.00032735591082083800000

simple a prosaic 0.00010122174008958400000

simple a thick 0.00043477319311109700000

simple a weak 0.04219849050603750000000

simple a amateur 0.00000788995548063395000

simple a unaware 0.00094998774372955800000

simple a irrational 0.00074126905265604500000

unwittingly r unknowingly 1.00000000000000000000000

unenthusiastic a unmoved 0.00026843767922031500000

unenthusiastic a lukewarm 0.97102297251464700000000

unenthusiastic a neutral 0.00241593911298285000000

unenthusiastic a unimpressed 0.00053687535844063000000

unenthusiastic a apathetic 0.00026843767922031500000

unenthusiastic a unresponsive 0.02495046229704860000000

unenthusiastic a cool 0.00053687535844063000000

tramp n turn 0.33333333333333300000000

tramp n march 0.66666666666666700000000

wary a alert 0.00017737176071224600000

wary a mindful 0.00067616158455044200000

wary a vigilant 0.00000382521710237907000

wary a watchful 0.06224601472378970000000

wary a cautious 0.77166216366347600000000

wary a apprehensive 0.00009253587033604500000

wary a safe 0.00175320327525463000000

wary a circumspect 0.00072812515026562700000

wary a leery 0.06342888414858980000000

wary a thoughtful 0.00000382521710237907000

wary a careful 0.06320298442821840000000

wary a canny 0.00000382521710237907000

wary a prudent 0.01335240291412890000000

wary a suspicious 0.02266867682937080000000

run v overshoot 0.00000057524588438189000

run v draw 0.00183993536195572000000

run v melt 0.00000038655287809203700

run v continue 0.01775952857099920000000

run v spring 0.00003868407366182770000

run v dash 0.00000600171522032116000

run v prescribe 0.00000018869300628985300

run v rotate 0.00000117546562929103000

run v slip 0.00144446134441719000000

run v span 0.00060954693248806900000

run v haunt 0.00002045825508831290000

run v lead 0.01692094570959640000000

run v up 0.00004545387463548250000

run v amass 0.00003196662118292260000

run v follow 0.00899402430269627000000

run v overrun 0.00001245812662457910000

run v fly 0.00481641765583385000000

run v enlarge 0.00000111680065239920000

run v order 0.00013063406772061600000

run v compel 0.00003169171109573360000

run v oppose 0.00024893127819690100000

run v straddle 0.00000018869300628985300

run v empty 0.00000818179995763617000

run v stream 0.00090907573105295200000

run v climb 0.00344198920960928000000

run v act 0.00086235006105286600000

run v range 0.00579699374036092000000

run v incline 0.00001027204215879040000

run v get 0.03257728131529140000000

run v shoot 0.00898407222375344000000

run v stray 0.00001351475505200780000

run v use 0.01203213306981850000000

run v unravel 0.00019908728102215600000

run v cost 0.00156334261448683000000

run v sneak 0.00004228259214199780000

run v conduct 0.00699492363326214000000

run v exceed 0.00525179618426257000000

run v hurry 0.00000665234340548117000

run v hasten 0.00000068301630633216700

run v wander 0.00000144527143251661000

run v hunt 0.00003670403122124190000

run v thrust 0.00000037738601257970600

run v withdraw 0.00066555852707262600000

run v review 0.00152752179644586000000

run v handle 0.00420014673905017000000

run v duplicate 0.00000211096790453111000

run v surge 0.00199560659761264000000

run v scuttle 0.00000048110558505683300

run v veer 0.00000172336631479233000

run v trek 0.00000608756069901530000

run v increase 0.00875100589693218000000

run v flee 0.00392528892290530000000

run v bleed 0.00000947713938858864000

run v trample 0.00007235172560664170000

run v meet 0.01367888623028200000000

run v perform 0.00472337268040844000000

run v invite 0.00013235484833628000000

run v amount 0.00157084604225234000000

run v have 0.08329170263116380000000

run v advance 0.00018342838636705600000

run v clip 0.00000564087336374470000

run v maneuver 0.00003830504907980250000

run v pace 0.00004347801353262090000

run v poach 0.00000018869300628985300

run v scurry 0.00381811807042241000000

run v spread 0.00178416685447515000000

run v hound 0.00000018869300628985300

run v press 0.00040929939044446700000

run v raise 0.00742120644551248000000

run v bound 0.00036890539337506600000

run v ship 0.00167543710304129000000

run v travel 0.00346224767658357000000

run v overflow 0.00124096052175482000000

run v last 0.01013038907092730000000

run v slide 0.00039171209876288000000

run v repeat 0.00075561639425328700000

run v steer 0.00151610005423418000000

run v reproduce 0.00018337374713628500000

run v summarize 0.00000101431043911068000

run v zoom 0.00000242833911982684000

run v abandon 0.00129679701315982000000

run v tumble 0.00057503423215921400000

run v survive 0.00110542808983909000000

run v split 0.00339320762868625000000

run v proceed 0.00349749327616282000000

run v reiterate 0.00006758083132433780000

run v shift 0.00033928727507328500000

run v feed 0.00136484371281028000000

run v surround 0.00002532873930110300000

run v reach 0.02419503975366690000000

run v find 0.00598698338841388000000

run v roll 0.00218086760739635000000

run v keep 0.00702394144350036000000

run v accelerate 0.00204266513238162000000

run v meander 0.00000027346791939623300

run v scorch 0.00004132162413877050000

run v flow 0.00143581470089254000000

run v tend 0.00000797422676941317000

run v move 0.02528422943637250000000

run v work 0.02870640760958350000000

run v drift 0.00093657131134011800000

run v escape 0.00118421820600865000000

run v sweep 0.00003965127476936490000

run v cast 0.00011553825353521500000

run v speed 0.00131318759831441000000

run v accumulate 0.00120415030512774000000

run v persevere 0.00000195947602270661000

run v ordain 0.00000136603261266433000

run v oversee 0.01432482970144050000000

run v gush 0.00000018869300628985300

run v govern 0.00787514178092571000000

run v issue 0.00215147480705442000000

run v mobilize 0.00000323393351245200000

run v blow 0.00005515769664873690000

run v bring 0.00673343054860271000000

run v pour 0.00020356049395716100000

run v rush 0.00191228072729506000000

run v execute 0.00166843625065962000000

run v contract 0.00034269594955981300000

run v direct 0.00016881048060878400000

run v lean 0.00009956506843762760000

run v regulate 0.00017246403451213500000

run v prevail 0.00016400115832159700000

run v coordinate 0.00116506832104210000000

run v incur 0.00202791788318285000000

run v retreat 0.00005330225366784600000

run v operate 0.18382457647670600000000

run v race 0.00388088480899745000000

run v contest 0.01200058209797180000000

run v trot 0.00090907573105295200000

run v transport 0.00011946055302824700000

run v tear 0.00072726058484236100000

run v echo 0.00000478775895222094000

run v play 0.01631229748197910000000

run v trail 0.00230393922788387000000

run v dissolve 0.00012950024239795000000

run v carry 0.00858879052552428000000

run v stretch 0.00306530941495067000000

run v surpass 0.00052786204958305500000

run v sail 0.00143606920270644000000

run v own 0.02361150017072880000000

run v flood 0.00012251207128646600000

run v progress 0.00106599191383065000000

run v persist 0.00009919843802826430000

run v leap 0.00252021491846028000000

run v fetch 0.00049033689775777300000

run v boost 0.00344527123571902000000

run v abscond 0.00000737108388072817000

run v percolate 0.00033057299311016400000

run v leak 0.00088880791813794800000

run v guide 0.00121501028835508000000

run v appear 0.00190080114612444000000

run v gain 0.00422869164113469000000

run v chase 0.00227315049978833000000

run v turn 0.00723812370055915000000

run v manage 0.02262325357847960000000

run v drop 0.01353021345758320000000

run v administer 0.00650085256187109000000

run v push 0.00532433661075017000000

run v force 0.00293958527752324000000

run v scramble 0.00268071634180266000000

run v spill 0.00001135629541118950000

run v supervise 0.00227578668957805000000

run v go 0.09038552798416540000000

run v campaign 0.00013192027506242700000

run v encounter 0.00094904455402066100000

run v spout 0.00000103186802616680000

run v command 0.00001483591988412770000

run v creep 0.00090951558867049300000

run v consume 0.00001410100552895680000

run v post 0.00525801967810903000000

run v manipulate 0.00095860295500345500000

run v cover 0.00441538889629060000000

run v prosecute 0.00033069283186043600000

run v fall 0.02905388235765360000000

run v redo 0.00001712300178353170000

run v lash 0.00000016036852835227600

run v maintain 0.00432902215349342000000

run v acquire 0.00129928600825644000000

run v total 0.00378630528135832000000

run v function 0.00377780270459950000000

run v dog 0.00001396256771921340000

run v tick 0.00049458048701333200000

run v finish 0.00728909317892605000000

run v rip 0.00005865004716470660000

run v head 0.03039685712683290000000

run v pass 0.00191942677388689000000

run v desert 0.00001227248952086300000

run v deplete 0.00002596984827649570000

run v endure 0.00003334762709359830000

run v extend 0.01203026541215350000000

run v dispatch 0.00004043692648017340000

run v roar 0.00065987248928823300000

run v control 0.01307214399556380000000

run v overlap 0.00000349208954106746000

run v trickle 0.00030302524368431700000

run v drive 0.00663449856893588000000

run v convey 0.00006176528052574480000

run v possess 0.00000064578797403775300

jack n buck 0.00001275396528595720000

jack n labourer 0.00049156333005136900000

jack n seaman 0.01549390285354740000000

jack n flag 0.98316492199859700000000

jack n standard 0.00083685785251841900000

reactionary a rigid 0.02216845313867360000000

reactionary a conservative 0.97783154686132600000000

cook n kitchener 1.00000000000000000000000

perfect v conclude 0.33333333333333300000000

perfect v make 0.33333333333333300000000

perfect v complete 0.33333333333333300000000

secret n plan 0.19018226202368800000000

secret n solution 0.01014731214094060000000

secret n puzzle 0.00011323370703928800000

secret n key 0.20273319165809500000000

secret n way 0.50547236844902800000000

secret n method 0.00010979978035917400000

secret n code 0.00005611838469011570000

secret n answer 0.04943543708527400000000

secret n plot 0.00478884960960762000000

secret n formula 0.01422970251793730000000

secret n mystery 0.00420480488390087000000

secret n blueprint 0.00357835166259894000000

secret n confidence 0.01494856809684070000000

okay v condone 0.00001462338790856680000

okay v endorse 0.07006796316389900000000

okay v authorize 0.27880322056764400000000

okay v approve 0.64753877453690400000000

okay v confirm 0.00299779452125624000000

okay v certify 0.00004387016372570100000

okay v accept 0.00036558469771417600000

okay v sanction 0.00001462338790856680000

okay v pass 0.00015354557303995400000

three n trio 0.00037738187347648300000

three n 3 0.99960474266277400000000

three n troika 0.00001787546374983420000

nada n nothing 0.97127389025894000000000

nada n zero 0.02872610974105990000000

mutter v mumble 1.00000000000000000000000

mag n magazine 1.00000000000000000000000

department n territory 0.01635600425575390000000

department n staff 0.00197170135665122000000

department n sphere 0.00004994133939378530000

department n assignment 0.00000889826432629801000

department n domain 0.00020296848063556500000

department n canton 0.00000029000784238878100

department n terrain 0.00000107802372133890000

department n zone 0.00001673924151345180000

department n duty 0.00250010518695981000000

department n class 0.00003533410810137870000

department n unit 0.01260936133695270000000

department n branch 0.00132292173069936000000

department n responsibility 0.00091240774394100200000

department n commission 0.03504067302516010000000

department n sector 0.01216219638915140000000

department n realm 0.00061624522971389800000

department n section 0.01185610081214050000000

department n area 0.00621386141690496000000

department n board 0.01660950619675400000000

department n ministry 0.51212768557922500000000

department n diocese 0.00002966220141016990000

department n compartment 0.00000016342144852473700

department n berth 0.00000855221051414359000

department n group 0.04599830694880650000000

department n force 0.00936245993404327000000

department n portion 0.00003139391206061010000

department n wing 0.00128623733364465000000

department n scene 0.00017039927495008300000

department n segment 0.00124268632739199000000

department n office 0.09095199331333580000000

department n shire 0.00003559380159492490000

department n agency 0.03926894215094430000000

department n line 0.00277521627955018000000

department n authority 0.01878541984250180000000

department n region 0.00514381901290098000000

department n circuit 0.00000177113717711028000

department n function 0.00174068878355338000000

department n vocation 0.00000057843554760508700

department n head 0.04132444473173280000000

department n state 0.02744037678350430000000

department n range 0.00150623024209260000000

department n occupation 0.00000245931542722547000

department n activity 0.00413283000524809000000

department n specialty 0.00140404542051890000000

department n circle 0.00025366032378117500000

department n control 0.00433635350900300000000

department n constituency 0.00011852802431260200000

department n arena 0.00029354796877359900000

department n interest 0.00480655890698162000000

department n neighborhood 0.00001781503303597240000

department n division 0.02248275896136160000000

department n dominion 0.00027812340149978000000

department n capacity 0.00053510040413161400000

department n theater 0.00000507593295162885000

department n ward 0.00027627270001188400000

department n jurisdiction 0.00100309983027542000000

department n bureau 0.01912381319492420000000

department n spot 0.00003136870481702720000

department n business 0.00665279521726192000000

department n township 0.00013073332106552200000

department n county 0.00242796618793205000000

department n district 0.00090637909803574800000

department n administration 0.00778619282531694000000

department n quarter 0.00262924129923663000000

department n province 0.00043813902069627100000

department n station 0.00182797799627468000000

department n corps 0.00001538655663599160000

department n field 0.00036482103624156500000

suspicion n guess 0.01764568474232020000000

suspicion n speculation 0.03874438500623540000000

suspicion n bit 0.00102264793394591000000

suspicion n idea 0.03373759105241520000000

suspicion n uncertainty 0.09857821022192550000000

suspicion n dash 0.00009429001137677310000

suspicion n doubt 0.33773976513881700000000

suspicion n distrust 0.04330530689176570000000

suspicion n misgiving 0.00029731399405591700000

suspicion n conclusion 0.01073406625254890000000

suspicion n inference 0.00007566448495940420000

suspicion n feeling 0.05969981464811430000000

suspicion n suggestion 0.04806448360470000000000

suspicion n dab 0.00030537427709062200000

suspicion n touch 0.00030537427709062200000

suspicion n conjecture 0.00003627416464478480000

suspicion n theory 0.00547373777485451000000

suspicion n impression 0.00961969914220433000000

suspicion n thought 0.00315685663128727000000

suspicion n reservation 0.00079576382258150700000

suspicion n hunch 0.00033772772817640000000

suspicion n hint 0.00029731399405591700000

suspicion n supposition 0.00007254832928956960000

suspicion n question 0.28984482704211100000000

suspicion n taste 0.00001527883343235260000

boss n owner 0.03379355129507090000000

boss n leader 0.28443924889842700000000

boss n overseer 0.00000730279456687560000

boss n employer 0.02920245697819970000000

boss n foreman 0.00000178954335617565000

boss n director 0.03550555774238290000000

boss n executive 0.01257122559932740000000

boss n head 0.07574372660488800000000

boss n manager 0.12323343621348500000000

boss n principal 0.00027512728924343800000

boss n master 0.00022459058598810200000

boss n chief 0.34648655215616900000000

boss n president 0.05595509811135160000000

boss n controller 0.00001079136353309110000

boss n chair 0.00000012305003680843500

boss n overman 0.00001227591333564470000

boss n supervisor 0.00237129725933538000000

boss n governor 0.00016584860130199900000

intransigent a rigid 0.00031995706845948100000

intransigent a adamant 0.01386514923353190000000

intransigent a tough 0.92529298790850200000000

intransigent a firm 0.05988199165258780000000

intransigent a unaffected 0.00063991413691896200000

plead v claim 0.10488846964890100000000

plead v demand 0.00008274741408892770000

plead v declare 0.00017596870885711700000

plead v cite 0.00008274741408892770000

plead v present 0.05239979997181360000000

plead v beg 0.10479959994362700000000

plead v argue 0.10510710250870500000000

plead v admit 0.00008886970527337460000

plead v allege 0.00528441484981806000000

plead v question 0.00017161711936230200000

plead v press 0.00004443485263668720000

plead v appeal 0.00004443485263668720000

plead v acknowledge 0.10488846964890100000000

plead v request 0.13977723477747300000000

plead v ask 0.01582357642501030000000

plead v testify 0.00013153385622043000000

plead v reply 0.19462782846673700000000

plead v seek 0.00133031951452168000000

plead v answer 0.00039460156866128900000

plead v own 0.00039398345843468900000

plead v push 0.00039460156866128900000

plead v assert 0.00008886970527337460000

plead v hold 0.00852550317685860000000

plead v suggest 0.00008886970527337460000

plead v pray 0.01905447271702310000000

plead v urge 0.14130992842114300000000

fierce a threatening 0.00019814775757881800000

fierce a dire 0.00003783530205292430000

fierce a formidable 0.00021616654486125300000

fierce a vicious 0.01116995924612730000000

fierce a awful 0.00000894903278865457000

fierce a frightful 0.00000231850121864505000

fierce a hard 0.00204170022251679000000

fierce a grim 0.00006011184346504440000

fierce a bad 0.00279954951776380000000

fierce a outrageous 0.00018239211060857300000

fierce a tough 0.11857394597981600000000

fierce a cruel 0.00016732862398516100000

fierce a horrible 0.00011629767719953600000

fierce a passionate 0.00007434249082281070000

fierce a ferocious 0.00500924325073409000000

fierce a strong 0.14489520445985600000000

fierce a powerful 0.00251799452994933000000

fierce a forceful 0.00009298613316235000000

fierce a frightening 0.00020854920451037700000

fierce a cut-throat 0.00171845526863497000000

fierce a aggressive 0.01031812284016500000000

fierce a severe 0.18570707168880100000000

fierce a dreadful 0.00003625545652464950000

fierce a deep 0.00009081267679778290000

fierce a keen 0.03277496116527370000000

fierce a savage 0.00016924180675950500000

fierce a dangerous 0.00366105628276312000000

fierce a violent 0.04268173342353420000000

fierce a hostile 0.00005028490755233980000

fierce a bitter 0.18390769429984100000000

fierce a vehement 0.02233991849225460000000

fierce a intense 0.08066868019014010000000

fierce a angry 0.00047304563811910600000

fierce a informal 0.00002321814573950810000

fierce a bloody 0.07796843872965030000000

fierce a mad 0.00053653982368144700000

fierce a rough 0.00004649306658117500000

fierce a harsh 0.01799277035120770000000

fierce a desperate 0.00128485616560277000000

fierce a acute 0.00217377908862531000000

fierce a grave 0.00402526540094870000000

fierce a brutal 0.02980774986462170000000

fierce a terrible 0.00061687071301133100000

fierce a enthusiastic 0.00081085641027088600000

fierce a devastating 0.00015337463893785900000

fierce a rampant 0.00034418609951755400000

fierce a turbulent 0.00007003109245220880000

fierce a furious 0.01117521384297370000000

guardsman n guard 1.00000000000000000000000

indeed r surely 0.00481978285886626000000

indeed r quite 0.08030265822422240000000

indeed r certainly 0.10545359613669100000000

indeed r so 0.48070483948093000000000

indeed r seriously 0.00492731426866016000000

indeed r exactly 0.03839094772080440000000

indeed r absolutely 0.22160255625358200000000

indeed r admittedly 0.01814779745654350000000

indeed r positively 0.00040929544609731900000

indeed r yes 0.01203593222069000000000

indeed r truly 0.00028490745435123000000

indeed r naturally 0.00643035623637972000000

indeed r rather 0.02649001624218180000000

fraternisation n fraternization 1.00000000000000000000000

depository n bank 0.15609688075309500000000

depository n magazine 0.00000355022599030726000

depository n storehouse 0.00000112025875538219000

depository n warehouse 0.00000403562059810433000

depository n treasurer 0.00005047568967840070000

depository n collection 0.00000287754738932566000

depository n bunker 0.00000334041223043259000

depository n treasury 0.00057433128707717800000

depository n depositary 0.83473493470377900000000

depository n depot 0.00000145520168160005000

depository n reservoir 0.00000383579860732441000

depository n deposit 0.00852316250111807000000

flinch v avoid 0.01063829787234020000000

flinch v withdraw 0.04255319148936170000000

flinch v flee 0.02127659574468070000000

flinch v start 0.88297872340425600000000

flinch v shrink 0.01063829787234020000000

flinch v escape 0.03191489361702120000000

electioneer v back 0.01470516470109770000000

electioneer v promote 0.01470516470109770000000

electioneer v advance 0.01470516470109770000000

electioneer v canvass 0.01470516470109770000000

electioneer v support 0.86765351769011900000000

electioneer v push 0.07352582350549010000000

proportion n distribution 0.00104937970396654000000

proportion n ratio 0.08352181259370220000000

proportion n bit 0.00734095998366999000000

proportion n size 0.00256792509027698000000

proportion n part 0.15678994385935500000000

proportion n agreement 0.00189656506946617000000

proportion n rate 0.09265303488770240000000

proportion n bulk 0.00127659602866041000000

proportion n relationship 0.00001090798243761350000

proportion n volume 0.02147868067800410000000

proportion n section 0.00011977608746629000000

proportion n adjustment 0.00000151970586881225000

proportion n arrangement 0.00493669681209109000000

proportion n correspondence 0.00020570039681446300000

proportion n extent 0.00022954353951293900000

proportion n portion 0.14136976505208500000000

proportion n comparison 0.00182045052264725000000

proportion n segment 0.01325917591423740000000

proportion n piece 0.00984266340128964000000

proportion n amount 0.07417373544625730000000

proportion n contrast 0.00152562361100672000000

proportion n quantity 0.00492451528334590000000

proportion n analogy 0.00000273276923972364000

proportion n opposition 0.00180447647006704000000

proportion n balance 0.00416661984805307000000

proportion n perspective 0.00015372155692564400000

proportion n percentage 0.21321658072076300000000

proportion n dimension 0.00051113431935714800000

proportion n share 0.15594752898958600000000

proportion n relation 0.00320223367614433000000

distaste n dislike 1.00000000000000000000000

desecrate v spoil 0.11111111111111100000000

desecrate v misuse 0.55555555555555600000000

desecrate v devastate 0.11111111111111100000000

desecrate v infect 0.11111111111111100000000

desecrate v abuse 0.11111111111111100000000

proceedings n proceeding 0.37120810652725100000000

proceedings n lawsuit 0.04137686511763160000000

proceedings n prosecution 0.01703605907317420000000

proceedings n case 0.50418616129457900000000

proceedings n litigation 0.02616567942077300000000

proceedings n story 0.00011511104822078800000

proceedings n account 0.00099652532804426300000

proceedings n report 0.03891549219032560000000

confide v admit 0.00100624019103545000000

confide v disclose 0.00100624019103545000000

confide v believe 0.00402496076414180000000

confide v tell 0.99295631866275200000000

confide v suggest 0.00100624019103545000000

pressure n intimidation 0.00027159782358905500000

pressure n requirement 0.00346206303832819000000

pressure n obligation 0.01617260458665950000000

pressure n humiliation 0.00010837404533937000000

pressure n density 0.00002932667348122910000

pressure n demand 0.39139254872779000000000

pressure n compression 0.00810963984737877000000

pressure n tension 0.20127598012879900000000

pressure n weight 0.01717157360330340000000

pressure n trouble 0.02607760104817600000000

pressure n difficulty 0.03441845237571170000000

pressure n mass 0.00021586201423371200000

pressure n confinement 0.00002717974793710660000

pressure n encumbrance 0.00080648352073380100000

pressure n misfortune 0.00001837452929162920000

pressure n force 0.07651438284134320000000

pressure n load 0.00213962219007397000000

pressure n coercion 0.00001210027788047710000

pressure n persuasion 0.00001539090688423280000

pressure n burden 0.05210647617859300000000

pressure n hassle 0.00069817259202724000000

pressure n duress 0.00096502634878599600000

pressure n discipline 0.00273516540420698000000

pressure n strain 0.02673077192968690000000

pressure n squeeze 0.03489989849839400000000

pressure n press 0.01709954724427000000000

pressure n constraint 0.03111942923317200000000

pressure n harassment 0.00004330282140325270000

pressure n stress 0.02329766224592020000000

pressure n insistence 0.00812488813492692000000

pressure n thrust 0.01714544530479930000000

pressure n necessity 0.00142738018282662000000

pressure n hardship 0.00186905144111510000000

pressure n urgency 0.00349862451293709000000

comb n card 0.50000000000000000000000

comb n beard 0.50000000000000000000000

churchman n clergyman 0.97136038186157500000000

churchman n minister 0.02863961813842480000000

continent n mainland 0.08556849676352780000000

continent n main 0.91443150323647200000000

truth n validity 0.00668605035928974000000

truth n fact 0.35646358046514900000000

truth n openness 0.00012201636672939000000

truth n axiom 0.00006071663367922040000

truth n actuality 0.00005951186588508340000

truth n maxim 0.00008193145303315310000

truth n honesty 0.00004463163205854180000

truth n sincerity 0.00041814485051063700000

truth n principle 0.05156322228441420000000

truth n fundamental 0.02879614891226860000000

truth n soundness 0.00004552657740208880000

truth n gospel 0.00003007701656160900000

truth n intelligence 0.00013753576765504600000

truth n law 0.04151154767891890000000

truth n motto 0.00619275254359012000000

truth n verity 0.00002362439800845220000

truth n perfection 0.00003990642285062490000

truth n reality 0.13876409454409800000000

truth n faith 0.00037372449107679900000

truth n rule 0.01034972346064520000000

truth n story 0.33759808545080800000000

truth n fidelity 0.00269929908122393000000

truth n honor 0.00441561686378380000000

truth n integrity 0.00014479757358826200000

truth n proposition 0.00147793005382187000000

truth n loyalty 0.00027372000633374600000

truth n precision 0.00002396884509008560000

truth n revelation 0.00069320007125642800000

truth n certainty 0.01090891433026900000000

blacklist n mark 0.99206979901327800000000

blacklist n label 0.00793020098672219000000

sexual a physical 1.00000000000000000000000

analyse v analyze 1.00000000000000000000000

queer a strange 0.00613496932515337000000

queer a poor 0.01226993865030670000000

queer a fantastic 0.06134969325153380000000

queer a uncomfortable 0.00613496932515337000000

queer a ridiculous 0.15337423312883400000000

queer a absurd 0.06748466257668720000000

queer a bad 0.03067484662576690000000

queer a bizarre 0.01840490797546010000000

queer a extraordinary 0.05521472392638040000000

queer a useless 0.01226993865030670000000

queer a worthless 0.01226993865030670000000

queer a curious 0.00613496932515337000000

queer a funny 0.01226993865030670000000

queer a bogus 0.00613496932515337000000

queer a wrong 0.30674846625766900000000

queer a exceptional 0.01840490797546010000000

queer a preposterous 0.00613496932515337000000

queer a unconventional 0.00613496932515337000000

queer a sick 0.01840490797546010000000

queer a eerie 0.00613496932515337000000

queer a unusual 0.03067484662576690000000

queer a unprecedented 0.01226993865030670000000

queer a giddy 0.01226993865030670000000

queer a unique 0.02453987730061350000000

queer a abnormal 0.01840490797546010000000

queer a doubtful 0.00613496932515337000000

queer a remarkable 0.00613496932515337000000

queer a wonderful 0.06748466257668720000000

opt v pick 0.02150678884556240000000

opt v elect 0.14394737802150900000000

opt v choose 0.21456877920031500000000

opt v prefer 0.03322030777037750000000

opt v select 0.00003495144449441330000

opt v take 0.03590607212620890000000

opt v decide 0.55081572259153300000000

audit n review 0.08478811426952060000000

audit n accounting 0.01376633655482990000000

audit n analysis 0.00938064973474488000000

audit n record 0.02290341465415070000000

audit n check 0.00346650194657179000000

audit n inspection 0.03758361762287130000000

audit n account 0.05189126486294890000000

audit n examination 0.00156469478881363000000

audit n scrutiny 0.03667049300669590000000

audit n statement 0.25936047695039200000000

audit n voucher 0.00005087817699378490000

audit n report 0.36680795211970300000000

audit n investigation 0.11176560531176400000000

caution v prescribe 0.00713622537034045000000

caution v reprimand 0.00000106962130475225000

caution v dissuade 0.00000365077612022304000

caution v advise 0.15415388912634400000000

caution v warn 0.65510872763187900000000

caution v urge 0.18357910257703300000000

caution v alert 0.00001733489697901940000

pair n set 0.13463513385393600000000

pair n twins 0.02748796359281150000000

pair n duo 0.46708042919006000000000

pair n couple 0.01591459938011510000000

pair n suit 0.00251206867038595000000

pair n combination 0.00602932491732164000000

pair n group 0.10976194089548100000000

pair n brace 0.00003269111990080440000

pair n collection 0.00157500756801986000000

pair n team 0.22782309134082700000000

pair n duet 0.00045451993491133700000

pair n two 0.00000534540673775524000

pair n grouping 0.00590623635007388000000

pair n tandem 0.00078164777941865100000

national a civil 0.00080339763176647500000

national a social 0.00133677793722606000000

national a civic 0.00661600403023507000000

national a governmental 0.00000930471140788378000

national a communal 0.00000810927166408944000

national a internal 0.00941492465492385000000

national a ethnic 0.00301064020382949000000

national a political 0.00826288185541606000000

national a public 0.25472098802257000000000

national a widespread 0.00319359821324962000000

national a general 0.12777366299741400000000

national a nationalist 0.00213628479188494000000

national a domestic 0.47779359528622500000000

national a nationwide 0.04391897835220170000000

national a countrywide 0.00043732143617053800000

national a country-wide 0.00061225001063875300000

national a royal 0.00001065153115237920000

national a federal 0.05869107920636020000000

national a tribal 0.00077096355506330300000

national a imperial 0.00047858630060067300000

capability n resistance 0.00028211888394537600000

capability n know-how 0.00022573328814695900000

capability n potential 0.01479118905590820000000

capability n power 0.12177069026627100000000

capability n ability 0.13902955332324700000000

capability n sense 0.00003725222545840740000

capability n turn 0.00009749638182469550000

capability n extent 0.00016391365786653800000

capability n intelligence 0.00000085462319711373900

capability n mind 0.00000512773918268248000

capability n understanding 0.00019071964766382200000

capability n head 0.01180607252735950000000

capability n judgment 0.00000512773918268248000

capability n range 0.00552143560215290000000

capability n skill 0.03224509574009560000000

capability n brain 0.00000085462319711373900

capability n capacity 0.52581439731746400000000

capability n reason 0.01610083972119300000000

capability n talent 0.00133247256854246000000

capability n facility 0.12642426523422200000000

capability n strength 0.00388349069763833000000

capability n tolerance 0.00000085462319711373900

capability n readiness 0.00020672941796961200000

capability n qualification 0.00006371509507336970000

temperate a patient 0.00364030680167090000000

temperate a stable 0.00182015340083545000000

temperate a quiet 0.00364030680167090000000

temperate a conservative 0.00026002191440506200000

temperate a moderate 0.09478584892829110000000

temperate a soft 0.00208017531524051000000

temperate a modest 0.00104008765762025000000

temperate a fair 0.00104008765762025000000

temperate a good 0.01248105189144310000000

temperate a severe 0.00078006574321518600000

temperate a strict 0.00026002191440506200000

temperate a reasonable 0.00130010957202533000000

temperate a peaceful 0.00130010957202533000000

temperate a comfortable 0.00026002191440506200000

temperate a lucid 0.00052004382881012400000

temperate a calm 0.00052004382881012400000

temperate a mild 0.77116499306825300000000

temperate a constant 0.09322571744186070000000

temperate a discreet 0.00026002191440506200000

temperate a warm 0.00026002191440506200000

temperate a cool 0.00052004382881012400000

temperate a steady 0.00416035063048104000000

temperate a nice 0.00026002191440506200000

temperate a clear 0.00208017531524051000000

temperate a smooth 0.00052004382881012400000

temperate a sane 0.00026002191440506200000

temperate a content 0.00026002191440506200000

temperate a tame 0.00130010957202533000000

taiwanese n amoy 1.00000000000000000000000

pain n passion 0.00009052825905352330000

pain n smart 0.03747854344881850000000

pain n burn 0.00001059291330066140000

pain n distress 0.00026684448655823400000

pain n pest 0.00001059291330066140000

pain n care 0.00042301927999010200000

pain n hurt 0.13626501438912000000000

pain n misery 0.00348549501998940000000

pain n ordeal 0.00017358506140905500000

pain n despair 0.00068847126367051600000

pain n sickness 0.00002556227054789290000

pain n pinch 0.12851374282918500000000

pain n penalty 0.00036622716028838000000

pain n cross 0.00808114129680153000000

pain n bitterness 0.00000852075684929751000

pain n irritation 0.00073796721897938600000

pain n worry 0.00524674328130680000000

pain n sadness 0.00001059291330066140000

pain n trouble 0.14024144286918700000000

pain n discomfort 0.02041360322170490000000

pain n agony 0.16729620697100100000000

pain n curse 0.00003234499459110450000

pain n load 0.00023109539218444800000

pain n cramp 0.13919217908665500000000

pain n burden 0.01889336224403260000000

pain n bale 0.00010097962759164100000

pain n annoyance 0.00002849867823775630000

pain n shock 0.00204669510888610000000

pain n nuisance 0.00027884825183031700000

pain n strain 0.01385145205086800000000

pain n gripe 0.00000852075684929751000

pain n wound 0.00010667476691192400000

pain n effort 0.01028740746411400000000

pain n woe 0.01458114515836060000000

pain n fever 0.00035317652632707400000

pain n catch 0.00010460631670325700000

pain n labor 0.00135355206283821000000

pain n suffering 0.10227009984817100000000

pain n burning 0.00008642709235833780000

pain n anxiety 0.00061859119416434500000

pain n disorder 0.03712416297813090000000

pain n trial 0.00861574257583000000000

quarantine v hospitalize 0.09999999999999980000000

quarantine v detain 0.90000000000000000000000

pouch n budget 1.00000000000000000000000

lag v delay 0.01640810059702710000000

lag v abate 0.00043883846912787100000

lag v stagger 0.00044137140607347000000

lag v drag 0.00002267476750085820000

lag v decrease 0.01297578569331270000000

lag v trail 0.93113070156237100000000

lag v jail 0.00145977076916063000000

lag v linger 0.00000667170771301844000

lag v fail 0.00245895327749304000000

lag v diminish 0.00016971688068686700000

lag v procrastinate 0.00000667170771301844000

lag v wane 0.02688216904902980000000

lag v falter 0.00681791120998196000000

lag v idle 0.00007811592560784000000

lag v imprison 0.00070254697720152600000

left-wing a socialist 0.31782974745213400000000

left-wing a communist 0.01857186811177420000000

left-wing a labour 0.00002289804492413950000

left-wing a leftist 0.66357548639116700000000

greatest a sterling 1.00000000000000000000000

all r only 0.06945635151957840000000

all r quite 0.04787400905356880000000

all r very 0.64173157444472000000000

all r just 0.11800308566373300000000

all r alone 0.00001279198538694690000

all r utterly 0.01203342065350100000000

all r completely 0.08847667490772040000000

all r exactly 0.00106219283304737000000

all r totally 0.00019922721757052100000

all r entirely 0.01157950034362740000000

all r wholly 0.00194087429895178000000

all r purely 0.00100838720560064000000

all r altogether 0.00451966906463862000000

all r fully 0.00210224080835494000000

minister n monk 0.00005471855853587030000

minister n representative 0.00578476933247466000000

minister n cleric 0.00001702791313353360000

minister n diplomat 0.01828783313953340000000

minister n superintendent 0.00674515730242919000000

minister n pastor 0.00005577348080848230000

minister n shepherd 0.00003936074423537630000

minister n agent 0.00243257555377962000000

minister n canon 0.00006181045935892240000

minister n cure 0.00000460808556534470000

minister n dignitary 0.00093739731252411800000

minister n ambassador 0.00417577628053728000000

minister n lecturer 0.00001764783585424060000

minister n commissioner 0.02323058548611120000000

minister n chancellor 0.01173662540850090000000

minister n missionary 0.00002847647164583410000

minister n premier 0.01533953696371380000000

minister n clergyman 0.00002083728799244220000

minister n rector 0.00005611863589547460000

minister n subordinate 0.00004909718436371590000

minister n father 0.00075687226425885500000

minister n envoy 0.00441129394031396000000

minister n dean 0.00012738655937637000000

minister n emissary 0.00000443238469950213000

minister n abbot 0.00002642421495530580000

minister n delegate 0.00781237896380690000000

minister n churchman 0.00000940928840385803000

minister n servant 0.00000533367740998655000

minister n official 0.34501157924622900000000

minister n bishop 0.00027700015211881000000

minister n prelate 0.00000634540850055817000

minister n officer 0.00447627138356478000000

minister n metropolitan 0.00001426959594422300000

minister n priest 0.00009042172376296780000

minister n deputy 0.03692612713367590000000

minister n secretary 0.51071571649760200000000

minister n substitute 0.00000312810621762568000

minister n proxy 0.00007475848231895910000

minister n archbishop 0.00002240632747542640000

minister n reader 0.00015271121237199000000

private a personal 0.07567377587751490000000

private a clandestine 0.00065702518869750700000

private a quiet 0.00240008413207516000000

private a classified 0.00002792711942045000000

private a separate 0.01680953180683740000000

private a covert 0.00001281087296298600000

private a individual 0.10998282823194900000000

private a particular 0.00658983339037399000000

private a reserved 0.00000069107440182506200

private a privatized 0.00768293673336196000000

private a non-state 0.01497683789618390000000

private a secret 0.00738805778753330000000

private a peculiar 0.00000069107440182506200

private a exclusive 0.00009144110074539420000

private a remote 0.00002292316259806140000

private a unofficial 0.01327490008323500000000

private a undisclosed 0.00005795048887113620000

private a own 0.01381995832479740000000

private a commercial 0.28595714410800900000000

private a single 0.02524797831933880000000

private a confidential 0.01274112478153770000000

private a special 0.01118034369666910000000

private a isolated 0.00002590267355801870000

private a retired 0.00007153905427032860000

private a independent 0.39530576302065600000000

muharram n moharram 1.00000000000000000000000

upgrade n raise 0.02152129615521070000000

upgrade n bank 0.51891128744438600000000

upgrade n ascent 0.00002645279913078910000

upgrade n ramp 0.00456174901687365000000

upgrade n hill 0.00463576835162578000000

upgrade n rise 0.44457119371808100000000

upgrade n climb 0.00577225251469187000000

mold n shell 0.00178359096313912000000

mold n figure 0.00356718192627824000000

mold n mould 0.99464922711058300000000

psychiatrical a psychiatric 1.00000000000000000000000

discount v reject 0.06995167814651940000000

discount v neglect 0.00778520528495957000000

discount v deduct 0.00006541647306867370000

discount v abate 0.00004627828205249640000

discount v discredit 0.00191747864967591000000

discount v lower 0.17555977919200600000000

discount v disregard 0.00065145418210279800000

discount v remove 0.13058056888107500000000

discount v dismiss 0.01768212118345270000000

discount v question 0.00773250535062244000000

discount v ignore 0.36459702447055800000000

discount v allow 0.00677239232092002000000

discount v slash 0.00465658633182696000000

discount v cut 0.14187510965724600000000

discount v doubt 0.00379240254127676000000

discount v modify 0.00105405152846088000000

discount v reduce 0.04723323452301170000000

discount v belittle 0.00001389630884083540000

discount v redeem 0.00040535073060564700000

discount v diminish 0.00580689565356194000000

discount v omit 0.00063827822645500600000

discount v minimize 0.00032514181911029600000

discount v depreciate 0.00108885384074398000000

discount v overlook 0.00971071061605015000000

discount v slight 0.00005758580579702090000

servant n scullion 0.00019456068622404900000

servant n helper 0.05378708255220170000000

servant n secretary 0.57374556648284600000000

servant n hand 0.05245537030679700000000

servant n employee 0.31981741997193100000000

leftover n rest 0.12711864406779700000000

leftover n remainder 0.87288135593220300000000

finish n closing 0.02932958214353430000000

finish n end 0.01486302162820440000000

finish n education 0.00009894721830079940000

finish n class 0.00002215217441698010000

finish n finale 0.00327817264863598000000

finish n summary 0.00000331845991639283000

finish n completion 0.00409771581079498000000

finish n conclusion 0.00000373422545059034000

finish n close 0.94695710028690400000000

finish n texture 0.00000041576553419751000

finish n knowledge 0.00000869177169130124000

finish n surface 0.00000767499616468729000

finish n ending 0.00001154286143885910000

finish n goal 0.00069165232995800200000

finish n grain 0.00062627767905452300000

intoxicate v thrill 0.07692307692307580000000

intoxicate v inflame 0.07692307692307580000000

intoxicate v fire 0.38461538461538600000000

intoxicate v overcome 0.38461538461538600000000

intoxicate v electrify 0.07692307692307580000000

beverage n draft 0.00015153251466951500000

beverage n drink 0.99984171065817700000000

beverage n liquor 0.00000675682715335880000

hum v move 1.00000000000000000000000

unemployed a idle 0.05476969963460830000000

unemployed a inactive 0.00549614498608137000000

unemployed a unused 0.00157588922674974000000

unemployed a redundant 0.00916601707745513000000

unemployed a untapped 0.00005038770317448600000

unemployed a jobless 0.92807638322232800000000

unemployed a unexercised 0.00001495998600318600000

unemployed a free 0.00085051816360004000000

handbook n plan 0.50000000000000000000000

handbook n abc 0.50000000000000000000000

gull n mark 0.99869035441259100000000

gull n sap 0.00130964558740940000000

change v replace 0.00762981757968538000000

change v renovate 0.00003120278787589590000

change v rotate 0.00005071463007674950000

change v substitute 0.00010385880443985600000

change v disguise 0.00002192159091887260000

change v cloud 0.00002290134838471900000

change v revise 0.04354963673582330000000

change v tailor 0.00002989222427037310000

change v reorganize 0.00004194402637593810000

change v deflect 0.00000699982383059856000

change v reshape 0.00034421972697121100000

change v adjust 0.02488935238877350000000

change v graduate 0.00001004271517930160000

change v bend 0.00002651567502303320000

change v resolve 0.00977603819968494000000

change v reconstruct 0.00003662762544937900000

change v increase 0.08724541656503130000000

change v trade 0.12164149150283800000000

change v twist 0.00001490939368994790000

change v merge 0.00228562917953164000000

change v deviate 0.00004705979337344920000

change v renew 0.00228934532918577000000

change v mature 0.00295863178245349000000

change v diverge 0.00001814793008951740000

change v correct 0.00938120666219450000000

change v transpose 0.00000936210882694797000

change v remodel 0.00000978032324144128000

change v transfer 0.00331436778812716000000

change v better 0.00002202124400633660000

change v reverse 0.01107895989763140000000

change v convert 0.00096000312050459300000

change v commute 0.00210610084045926000000

change v amend 0.03608003434319310000000

change v edit 0.00003025903235649290000

change v shift 0.00960938942122404000000

change v switch 0.04423166262341990000000

change v readjust 0.00002056739102010990000

change v grow 0.03182153449404250000000

change v swerve 0.00184283823540185000000

change v barter 0.00062824433128588100000

change v exchange 0.20290109162423700000000

change v reform 0.00909371541691653000000

change v dress 0.00001617890760154020000

change v recast 0.00000335928409369965000

change v modify 0.01718157392395610000000

change v alter 0.07650863250415690000000

change v vary 0.00319435832819230000000

change v regenerate 0.00000680738710242908000

change v intensify 0.00084911664607066200000

change v reduce 0.02415451194084920000000

change v worsen 0.01610303223088470000000

change v improve 0.08905311379735300000000

change v decline 0.01495109527114280000000

change v deteriorate 0.01992649941496700000000

change v bandy 0.00000034503617962962900

change v translate 0.00006746874765626180000

change v transform 0.00308924402225679000000

change v swap 0.01868968012163400000000

change v moderate 0.00066079677936824000000

change v diversify 0.00046925990222221600000

change v develop 0.02382160114625570000000

change v modernize 0.00038792053807977600000

change v turn 0.00377038863217664000000

change v displace 0.00045283220592880100000

change v diminish 0.00474057398482678000000

change v fluctuate 0.00240535759016600000000

change v adapt 0.00177278806836627000000

change v break 0.00936082476407965000000

change v deepen 0.00038968130543502000000

change v mellow 0.00004422811764964440000

change v revamp 0.00047905229390486900000

change v shape 0.00004536507521103200000

change v redo 0.00000315554492363330000

change v invert 0.00000746995636563375000

change v accommodate 0.00012811227408784400000

change v evolve 0.00061696938366641500000

change v temper 0.00005313836554841730000

change v grade 0.00003056416482736240000

change v reorder 0.00000671856818739941000

change v distort 0.00034472751758082200000

proposed a recommended 0.77982358076366800000000

proposed a intended 0.06608674413251430000000

proposed a suggested 0.08637286495800120000000

proposed a expected 0.06608674413251420000000

proposed a prospective 0.00163006601330198000000

flashlight n torch 0.24999999999999900000000

flashlight n spotlight 0.75000000000000100000000

flammable a inflammable 1.00000000000000000000000

extricate v deliver 0.00036005203145837300000

extricate v remove 0.00102789307060381000000

extricate v withdraw 0.00108015609437512000000

extricate v clear 0.00066784103914543300000

extricate v relieve 0.99686405776441700000000

eventful a critical 0.00003444463038380220000

eventful a crucial 0.00274183881295389000000

eventful a noteworthy 0.00006888926076760450000

eventful a busy 0.00003444463038380220000

eventful a impressive 0.00044778019498943300000

eventful a decisive 0.00003444463038380220000

eventful a outstanding 0.00020666778230281500000

eventful a exciting 0.00003444463038380220000

eventful a important 0.00006888926076760450000

eventful a notable 0.00003444463038380220000

eventful a active 0.99601815449322900000000

eventful a significant 0.00027555704307042000000

engaging a beautiful 0.05172413793103450000000

engaging a pleasant 0.06896551724137920000000

engaging a attractive 0.70689655172413800000000

engaging a tempting 0.01724137931034480000000

engaging a fair 0.05172413793103450000000

engaging a appealing 0.03448275862068950000000

engaging a lovely 0.01724137931034480000000

engaging a charming 0.01724137931034480000000

engaging a enjoyable 0.01724137931034480000000

engaging a sweet 0.01724137931034480000000

favorite n jewel 0.00017218775278102700000

favorite n ideal 0.00233350572773763000000

favorite n darling 0.26545397442520700000000

favorite n choice 0.29464505213693400000000

favorite n star 0.00343042247285683000000

favorite n idol 0.00178461303535221000000

favorite n front-runner 0.00499244580996005000000

favorite n mistress 0.00130622950880369000000

favorite n etc 0.00028304350502097300000

favorite n honey 0.00001897392754100690000

favorite n preference 0.00252154629378329000000

favorite n dear 0.00000393136378551257000

favorite n god 0.00089702785658131100000

favorite n love 0.00066508527602873400000

favorite n hero 0.41857597020349100000000

favorite n favourite 0.00018940327877653400000

favorite n pick 0.00239423280438240000000

favorite n pet 0.00001270748296582910000

favorite n duck 0.00031964713801014100000

punish v ground 0.00061697888452225900000

punish v penalize 0.31987073418849800000000

punish v whip 0.00000762694647518753000

punish v bruise 0.00792942950851191000000

punish v stone 0.00005093717468291670000

punish v strike 0.00427800791807767000000

punish v misuse 0.00024785404844916500000

punish v hurt 0.05135608579141310000000

punish v lecture 0.00001942079611730970000

punish v strap 0.00000762694647518753000

punish v dismiss 0.00930242666479155000000

punish v behead 0.00002704774259249720000

punish v discipline 0.03227668034820680000000

punish v correct 0.01256627292464590000000

punish v rebuke 0.00000762694647518753000

punish v pummel 0.00000762694647518753000

punish v penalise 0.15747737734643600000000

punish v handicap 0.00011168608322442300000

punish v switch 0.00004154668265655200000

punish v sentence 0.25260566406168200000000

punish v kick 0.00016855645250594100000

punish v execute 0.00554596002184431000000

punish v hang 0.00176873525587677000000

punish v blacklist 0.00000762694647518753000

punish v chastise 0.00001555562575655020000

punish v train 0.00278892866484784000000

punish v expel 0.00759612206709986000000

punish v electrocute 0.00002288083942556260000

punish v fine 0.00744577052587204000000

punish v pillory 0.00001018117842873350000

punish v incarcerate 0.00002629278970617690000

punish v batter 0.01585766958619440000000

punish v exile 0.00006780511403506410000

punish v disadvantage 0.00002686181276698270000

punish v smash 0.00003050778590075010000

punish v lash 0.00002778032059504290000

punish v beat 0.09011689537337290000000

punish v criticize 0.01345806707038970000000

punish v fix 0.00595955921603401000000

punish v warm 0.00005496592577537040000

punish v imprison 0.00019461947668719500000

coexistence n accord 1.00000000000000000000000

voice v say 0.08844797913265790000000

voice v express 0.81798016381265900000000

voice v vent 0.03067929739497810000000

voice v announce 0.05522273531096050000000

voice v speak 0.00766982434874452000000

charming a beautiful 0.00160188219135212000000

charming a elegant 0.93557830504304000000000

charming a attractive 0.02323537291276070000000

charming a tempting 0.00053396073045070500000

charming a fair 0.00249720416753990000000

charming a appealing 0.00106792146090141000000

charming a sexy 0.01786464938908210000000

charming a lovely 0.00053396073045070500000

charming a engaging 0.00106792146090141000000

charming a enjoyable 0.00053396073045070500000

charming a sweet 0.00053396073045070500000

charming a nice 0.00800941095676060000000

charming a smooth 0.00053396073045070500000

charming a delicate 0.00480564657405636000000

charming a desirable 0.00160188219135212000000

proceed n result 0.26666666666666700000000

proceed n product 0.26666666666666700000000

proceed n earnings 0.26666666666666700000000

proceed n outcome 0.06666666666666660000000

proceed n yield 0.06666666666666660000000

proceed n total 0.06666666666666660000000

beckon v call 1.00000000000000000000000

couple v link 0.00009208883587765120000

couple v combine 0.79219621132619300000000

couple v pair 0.00019089486266181100000

couple v match 0.10277221096028200000000

couple v weld 0.00065352382160739200000

couple v incorporate 0.00019089486266181100000

couple v marry 0.00019089486266181100000

couple v attach 0.00009208883587765120000

couple v fix 0.10362119163217700000000

atypical a strange 0.00117660901282502000000

atypical a different 0.01882574420520060000000

atypical a exceptional 0.97999764678197400000000

apportionment n distribution 0.01742305731199320000000

apportionment n listing 0.00035937061122343300000

apportionment n apportioning 0.97569120947162200000000

apportionment n allocation 0.00652636260516135000000

pen n prison 0.77316468295368000000000

pen n pound 0.21950967251538500000000

pen n compound 0.00589284452104346000000

pen n wall 0.00143280000989136000000

lot n company 0.03437858865400250000000

lot n chip 0.00136417083673517000000

lot n raft 0.00000920195533290357000

lot n slew 0.00006342640610734800000

lot n mess 0.00011809158999338400000

lot n pile 0.00106967796849805000000

lot n packet 0.00000358593153080508000

lot n hazard 0.00009730377031166270000

lot n cluster 0.00005158110158522840000

lot n consignment 0.00154741263448465000000

lot n package 0.00124502063790641000000

lot n race 0.00207829194189860000000

lot n type 0.00145212704612852000000

lot n straw 0.00001311822792785410000

lot n zillion 0.00000170048460352837000

lot n bundle 0.00002860515278356190000

lot n deal 0.01190114275521720000000

lot n ration 0.00000224075618335175000

lot n end 0.00830530215484289000000

lot n clan 0.00000004385603409813180

lot n outfit 0.00000150648565580193000

lot n destiny 0.00006412046106745200000

lot n bunch 0.00098946888197817400000

lot n set 0.00070585391259385500000

lot n number 0.01708588396489630000000

lot n tossup 0.00000204137857359381000

lot n mint 0.00071605799093838300000

lot n estate 0.00009302924223712460000

lot n class 0.00053447466778891500000

lot n flock 0.00000475908705503614000

lot n award 0.00106642223763994000000

lot n kind 0.00346420600282189000000

lot n part 0.00603080986239306000000

lot n block 0.12312665575378100000000

lot n status 0.00017849912686742900000

lot n peck 0.00000144232324324895000

lot n band 0.00026336789816631900000

lot n bind 0.00000581830182937265000

lot n commission 0.00316182678112214000000

lot n dole 0.00020498964262910200000

lot n abundance 0.00022497308010121700000

lot n heap 0.00000064753893668686300

lot n article 0.00016385630187653000000

lot n caste 0.00000009043754253989540

lot n crew 0.00065793140621999200000

lot n volume 0.28595177623152000000000

lot n section 0.00105578439156181000000

lot n crowd 0.00036100136201561400000

lot n mass 0.00013369495401720400000

lot n blood 0.00035064025888265200000

lot n group 0.00973600357830635000000

lot n mountain 0.00006716126275312250000

lot n load 0.00186127098658639000000

lot n portion 0.00047880529352789200000

lot n order 0.00346557745153050000000

lot n fluke 0.00001544279836527440000

lot n ballot 0.00008393319881391500000

lot n variety 0.00014918781191286700000

lot n sort 0.00303699275347781000000

lot n affiliate 0.00018086628674424000000

lot n support 0.00443506323431303000000

lot n piece 0.00063571777431357100000

lot n check 0.00013781789831941500000

lot n collection 0.00015389317459947000000

lot n square 0.00001190583997125080000

lot n predicament 0.00000451003892339354000

lot n draw 0.00036545738082399100000

lot n quantity 0.00237149966078465000000

lot n fate 0.00082805036355422900000

lot n host 0.00056618428965640200000

lot n string 0.00034019808070666000000

lot n gang 0.00003481651885104030000

lot n state 0.00520285468562086000000

lot n sight 0.00041262105881043100000

lot n proportion 0.00062909619453659600000

lot n apportionment 0.00000033165763930442900

lot n fortune 0.00029954085135664500000

lot n cut 0.00118412989278486000000

lot n condition 0.00190993302290861000000

lot n pittance 0.00000294656973999820000

lot n parcel 0.00043178158900864000000

lot n circle 0.00000091897735765078500

lot n team 0.00339085925084414000000

lot n allowance 0.00002859890775447800000

lot n counter 0.00052763873354914200000

lot n clearing 0.00051250912823206300000

lot n measure 0.00160331305917810000000

lot n strain 0.00000426615071839743000

lot n plenty 0.01754408605463530000000

lot n pot 0.00016451646410287600000

lot n interest 0.00888051645004562000000

lot n property 0.00634154359408662000000

lot n bone 0.00001074925255734260000

lot n army 0.00088214748407497700000

lot n stroke 0.00003547825095116190000

lot n division 0.00110170767354493000000

lot n plot 0.00056458710587704700000

lot n troop 0.00000135598987781360000

lot n lottery 0.00013230285791328500000

lot n tribe 0.00013926700529437500000

lot n spate 0.00006293342735772590000

lot n pack 0.00002508625031572550000

lot n acreage 0.00007673138033280540000

lot n situation 0.00375403129468781000000

lot n mob 0.00020384840113217000000

lot n species 0.00021772053273882500000

lot n family 0.00101708834285933000000

lot n heritage 0.00001208862721221470000

lot n batch 0.00172286910798920000000

lot n gamble 0.00000829171990745120000

lot n stable 0.00000829528297379626000

lot n cup 0.00020378418113348600000

lot n ilk 0.00001053797847900860000

lot n party 0.00286793088383563000000

lot n percentage 0.00060157791440584700000

lot n luck 0.00029912323396753100000

lot n shipment 0.01174280458224690000000

lot n die 0.00001543029299617320000

lot n chance 0.00358417334763547000000

lot n body 0.00093150685167365400000

lot n station 0.00038440089205864000000

lot n league 0.00060053507199158300000

lot n share 0.37789665838153100000000

lot n patch 0.00000189586694786504000

lot n field 0.00210281352482198000000

lot n item 0.00012158879473328300000

lot n plight 0.00000402341261964005000

lot n contingent 0.00000056606158653264500

lot n allotment 0.00002204573802697720000

lot n breed 0.00026740120390483700000

parity n congruence 0.00003625721369027950000

parity n equilibrium 0.00038491568130083500000

parity n agreement 0.12394372521898500000000

parity n par 0.67960495643124100000000

parity n connection 0.00056564478928003500000

parity n comparison 0.00374266228008572000000

parity n proportion 0.00054218732393760300000

parity n concert 0.00003598181483469440000

parity n balance 0.03166027196099900000000

parity n harmony 0.15948339728564600000000

untold a many 1.00000000000000000000000

lion n celebrity 0.00068142704460543800000

lion n soldier 0.00742400974377679000000

lion n vip 0.00002732548348459620000

lion n warrior 0.00032970715932772700000

lion n wonder 0.00000988287807052250000

lion n champion 0.00115306583630527000000

lion n star 0.60102168570920700000000

lion n wildcat 0.00000988287807052250000

lion n face 0.00007020393670313650000

lion n prodigy 0.00031027291286464000000

lion n idol 0.00152478094144375000000

lion n fighter 0.00146128122555970000000

lion n great 0.16806822446730900000000

lion n cavalier 0.00071752442221451600000

lion n big 0.00224187613439326000000

lion n favorite 0.00014555575567385400000

lion n tiger 0.21176540548335300000000

lion n victor 0.00001744260541407370000

lion n hero 0.00030405580106379200000

lion n heavy 0.00003320358067216060000

lion n knight 0.00077031186778403000000

lion n somebody 0.00105097454210972000000

lion n cat 0.00082869600992128200000

lion n leo 0.00003320358067216060000

twenty n 20 1.00000000000000000000000

trees n woods 1.00000000000000000000000

three-fourths n three-quarters 1.00000000000000000000000

sweat v fret 0.00028849743746393200000

sweat v worry 0.36584020636052000000000

sweat v last 0.00144248718731968000000

sweat v work 0.02971523605878560000000

sweat v take 0.36208973967348900000000

sweat v chafe 0.00028849743746393200000

sweat v fuss 0.00057699487492787200000

sweat v suffer 0.23975834097003000000000

retreat v relinquish 0.01275030144278740000000

retreat v evade 0.00052051365527801800000

retreat v fly 0.00005907877268788200000

retreat v avoid 0.00179091344052094000000

retreat v resign 0.05541317598819780000000

retreat v vacate 0.00027567789300256100000

retreat v remove 0.00060163473099609300000

retreat v retract 0.00029003341657863300000

retreat v ebb 0.00018718787603058000000

retreat v backtrack 0.00032161819350511600000

retreat v withdraw 0.20423835554925800000000

retreat v flee 0.23801085554851100000000

retreat v reverse 0.09268421363489400000000

retreat v retire 0.00063313599438290000000

retreat v abandon 0.00050812661852586800000

retreat v leave 0.19454908723659600000000

retreat v quit 0.00472168726930947000000

retreat v sequester 0.00002589155241980010000

retreat v shrink 0.09713780501632390000000

retreat v escape 0.00073662220318211700000

retreat v hide 0.00023798891185754600000

retreat v evacuate 0.00193674134264821000000

retreat v run 0.00347922477034350000000

retreat v depart 0.00000743047772535025000

retreat v go 0.07288723726298780000000

retreat v reel 0.00024519669058691600000

retreat v recede 0.01575026451086120000000

occupation n employment 0.00139864719517652000000

occupation n holding 0.00001930786230246000000

occupation n assignment 0.00001758220040879690000

occupation n bag 0.00001059907928246470000

occupation n takeover 0.01386464342016700000000

occupation n mission 0.00012707076957363700000

occupation n care 0.00000047924830754380200

occupation n affair 0.00010019346526177300000

occupation n duty 0.00013289651724969600000

occupation n diversion 0.00000047924830754380200

occupation n enterprise 0.00002422853119731990000

occupation n commission 0.00050503644829408600000

occupation n oppression 0.00182281463184409000000

occupation n task 0.00003111067168914060000

occupation n area 0.02037065863760590000000

occupation n annexation 0.01385339120201510000000

occupation n conquest 0.00000047924830754380200

occupation n career 0.01294082686906100000000

occupation n berth 0.00019860338892862000000

occupation n defeat 0.00333961576778970000000

occupation n appointment 0.00002184179354702160000

occupation n preoccupation 0.00006447815881177690000

occupation n office 0.00058602332315142600000

occupation n line 0.00125870436982869000000

occupation n charge 0.00153325882381556000000

occupation n position 0.00193966453798091000000

occupation n living 0.00000205585682303406000

occupation n function 0.00000510523723026605000

occupation n vocation 0.00000510523723026605000

occupation n job 0.01217362192431790000000

occupation n activity 0.00090042452034953400000

occupation n place 0.00057569913542187400000

occupation n trade 0.00194844527723505000000

occupation n invasion 0.29178478653687800000000

occupation n skill 0.00000231299446136113000

occupation n rule 0.29019134531123200000000

occupation n control 0.28333906305861900000000

occupation n concern 0.00025979822382826700000

occupation n interest 0.00061404695747050800000

occupation n work 0.00231580963179828000000

occupation n attack 0.02115473402403870000000

occupation n craft 0.00000462598892272225000

occupation n situation 0.00191541896857533000000

occupation n thing 0.00101116970151665000000

occupation n business 0.00979144220555148000000

occupation n occupancy 0.00000205585682303406000

occupation n undertaking 0.00437706811088717000000

occupation n province 0.00160899065099788000000

occupation n station 0.00001907964421292800000

occupation n seizure 0.00011044859389466400000

occupation n field 0.00056320454742054400000

occupation n capture 0.00000047924830754380200

occupation n use 0.00016284097003841400000

occupation n post 0.00099818617601177700000

serene a patient 0.00085807529842711600000

serene a quiet 0.07187259095624150000000

serene a fair 0.00085807529842711600000

serene a self-possessed 0.00294670631663691000000

serene a cheerful 0.84599073797426800000000

serene a peaceful 0.00057205019895140800000

serene a balanced 0.00114410039790282000000

serene a comfortable 0.03230850757267390000000

serene a calm 0.01046981422488510000000

serene a bright 0.00314627609423276000000

serene a cool 0.00028602509947570400000

serene a steady 0.00085807529842711600000

serene a satisfied 0.00809515810719960000000

serene a clear 0.01973573186382370000000

serene a smooth 0.00028602509947570400000

serene a content 0.00057205019895140800000

depositary n warehouse 0.00001463621452863170000

depositary n depository 0.99527428761146300000000

depositary n trustee 0.00000035698084216174100

depositary n deposit 0.00471071919316644000000

covering n mask 0.00045245214805985900000

covering n darkness 0.00045245214805985900000

covering n application 0.00246141520913582000000

covering n top 0.00056836387569510500000

covering n protection 0.61696374909442400000000

covering n chase 0.00034773518290574500000

covering n ceiling 0.34515389615471200000000

covering n cover 0.00045245214805985900000

covering n roof 0.00045245214805985900000

covering n housing 0.00091609905860085400000

covering n carpet 0.03177893283228680000000

rut n course 0.09857473537959870000000

rut n track 0.00626994156386643000000

rut n system 0.88683576185696800000000

rut n scar 0.00039187134774165100000

rut n score 0.00019593567387082500000

rut n trench 0.00019593567387082500000

rut n line 0.00137391919896851000000

rut n heat 0.00039187134774165100000

rut n depression 0.00330905398089865000000

rut n round 0.00246097397647448000000

rudimentary a basic 0.99173255503104000000000

rudimentary a early 0.00433449543282797000000

rudimentary a simple 0.00393294953613198000000

reproach n brand 0.96808510638297900000000

reproach n spot 0.03191489361702130000000

remodeling n reconstruction 1.00000000000000000000000

behavior n front 0.00015679951641159400000

behavior n strategy 0.01645418292818400000000

behavior n course 0.00595948014420333000000

behavior n response 0.00110426046782365000000

behavior n speech 0.00012397424018108100000

behavior n form 0.00019855241888413800000

behavior n act 0.12166436433810300000000

behavior n formality 0.00003213133394833290000

behavior n talk 0.05796865655031380000000

behavior n reaction 0.00787505105806630000000

behavior n practice 0.26606040948475100000000

behavior n disobedience 0.04220492626156400000000

behavior n operation 0.02412218050985230000000

behavior n presence 0.00006765486475214080000

behavior n adjustment 0.00001314044717811130000

behavior n tone 0.00106366375680365000000

behavior n way 0.04972535705646870000000

behavior n convention 0.00003094954735718850000

behavior n dealings 0.00434895946858612000000

behavior n code 0.01521740845661500000000

behavior n attitude 0.05166673253897830000000

behavior n style 0.00002569336848594400000

behavior n compliance 0.00000131404471781112000

behavior n function 0.00010602917792118300000

behavior n execution 0.00027616068159419400000

behavior n management 0.00979821779402392000000

behavior n air 0.00041285342052760800000

behavior n action 0.04483311979864710000000

behavior n delivery 0.00232343483815440000000

behavior n manner 0.00000131404471781112000

behavior n conduct 0.05349474403653240000000

behavior n tact 0.00128760791984433000000

behavior n life 0.05405565893953450000000

behavior n protocol 0.00000131404471781112000

behavior n port 0.00011374109942987700000

behavior n expression 0.00002569336848594400000

behavior n performance 0.11771560003380100000000

behavior n decorum 0.00000131404471781112000

behavior n mode 0.00330299422916588000000

behavior n role 0.04616435972595420000000

relapse n sinking 0.00046140576883118700000

relapse n setback 0.84480298780035900000000

relapse n fall 0.00738249230129908000000

relapse n retreat 0.00092281153766237500000

relapse n deterioration 0.00046140576883118700000

relapse n return 0.01040603237825740000000

relapse n decline 0.13556286444476000000000

hit v achieve 0.00953646315378121000000

hit v solicit 0.00000105681535020518000

hit v overwhelm 0.00102331705420950000000

hit v slap 0.00131552374793540000000

hit v whip 0.00001604929085143210000

hit v thrash 0.00000983280194042238000

hit v cuff 0.00000496798998465032000

hit v punch 0.00164487194489934000000

hit v jolt 0.00797725284218588000000

hit v demand 0.00009208066624073700000

hit v pop 0.00019683025774033700000

hit v delight 0.00000017172145832490100

hit v beg 0.00000046725998008644700

hit v bump 0.00005920759858547370000

hit v slay 0.00004685118557089480000

hit v score 0.01278385613942010000000

hit v shoot 0.01970490069875820000000

hit v tally 0.00000594212250989142000

hit v remove 0.00039100487236418000000

hit v bat 0.00149160473983583000000

hit v strike 0.07057618415455740000000

hit v hook 0.00000177008039280867000

hit v unearth 0.00000958427748090279000

hit v crown 0.00003252823421627960000

hit v smack 0.00452452403409235000000

hit v scrape 0.00131552374793540000000

hit v occur 0.01210530904063420000000

hit v strap 0.00000082486181071140200

hit v secure 0.00010665552907302000000

hit v buffet 0.00140675965592444000000

hit v respond 0.00013849065584659700000

hit v censure 0.00000029265077147521900

hit v attack 0.00624368800635201000000

hit v double 0.00273275018705578000000

hit v catch 0.00283433272575968000000

hit v meet 0.02855717106248520000000

hit v belt 0.01005827532275610000000

hit v please 0.00059729500123783900000

hit v bash 0.00001628123450415100000

hit v appeal 0.00094679443125881900000

hit v succeed 0.00015737687199779300000

hit v bang 0.00191847213240580000000

hit v touch 0.16010180894154300000000

hit v gratify 0.00054895975678379700000

hit v pummel 0.00082302720427033900000

hit v hatch 0.00000659889448569126000

hit v whack 0.00000022956927054575600

hit v clap 0.00000332875442291347000

hit v thump 0.00082220234245962800000

hit v fell 0.00000101359039421148000

hit v revile 0.00082654687267077500000

hit v club 0.00109626978994617000000

hit v condemn 0.00069346065772277000000

hit v request 0.00007590564264471120000

hit v switch 0.00025372910604720900000

hit v reach 0.32688285707199100000000

hit v make 0.03594027528086450000000

hit v trim 0.00098024157418794400000

hit v find 0.00686380144341111000000

hit v discover 0.00020585086700824700000

hit v slug 0.00191847213240580000000

hit v influence 0.00104101653917551000000

hit v sink 0.02919451883086130000000

hit v slander 0.00000388631543874827000

hit v bludgeon 0.00014053684149309900000

hit v crush 0.00209942402468965000000

hit v connect 0.00086141964811788700000

hit v tap 0.00724763837093993000000

hit v kick 0.00004302409419953380000

hit v crack 0.00088444388034174000000

hit v knock 0.00433596757543590000000

hit v discern 0.00000108255739626021000

hit v contact 0.00002545299714668280000

hit v castigate 0.00000109626978994617000

hit v accord 0.00025830141407028400000

hit v seek 0.00073035614511561100000

hit v run 0.00886459919983003000000

hit v punish 0.00121646877802193000000

hit v fit 0.00007106569224270090000

hit v concoct 0.00000082486181071140200

hit v denounce 0.00001760217377062530000

hit v thresh 0.00000257340326278443000

hit v tickle 0.00000058530154295043800

hit v belittle 0.00000058342647696184900

hit v jar 0.00000373599128296964000

hit v perceive 0.00093769366594410800000

hit v impact 0.00457876848053561000000

hit v murder 0.00017487925777118900000

hit v hammer 0.00724128350528000000000

hit v detect 0.00011790952651722800000

hit v blast 0.00619392431319586000000

hit v upset 0.00192955849437296000000

hit v gain 0.00773216399437197000000

hit v propel 0.00095229311889368400000

hit v single 0.00001200295390452010000

hit v drop 0.02594840043835760000000

hit v batter 0.01873404636770250000000

hit v go 0.02307431270613620000000

hit v malign 0.00007474566749633030000

hit v dispute 0.00000960821127027199000

hit v uncover 0.00002894303932655840000

hit v minimize 0.00005085148001898520000

hit v spot 0.00007253059251038820000

hit v box 0.00000070842910890314000

hit v dong 0.00000082486181071140200

hit v devastate 0.01267891674115300000000

hit v notice 0.00007063885942132400000

hit v plug 0.00000111751258218662000

hit v lash 0.00569284693209857000000

hit v affect 0.06088394323324360000000

hit v beat 0.00470517845916482000000

hit v accomplish 0.00023792360866076500000

hit v depreciate 0.00055932054757526100000

hit v triple 0.00013486653942906400000

hit v suit 0.00000245246639730768000

hit v function 0.00010764203572436200000

hit v criticize 0.00111614313104039000000

hit v shatter 0.00055733164354909400000

hit v dig 0.00000164972362142280000

hit v jab 0.00000100085495125944000

hit v stumble 0.00000258437159881738000

hit v attain 0.00067953682664498200000

hit v dispatch 0.00000925562132609184000

hit v pound 0.00528078075369510000000

hit v drive 0.01373750040646160000000

extensive a expanded 0.00054923210346619200000

extensive a huge 0.00236065561090145000000

extensive a great 0.05788283172509430000000

extensive a far-reaching 0.00016935493300794900000

extensive a inclusive 0.00005893048320452700000

extensive a unbounded 0.00000458505356123286000

extensive a immense 0.00005739338991008350000

extensive a substantial 0.02889178429007810000000

extensive a prevalent 0.00320064302552540000000

extensive a expansive 0.00005893048320452700000

extensive a major 0.09319914033312500000000

extensive a entire 0.00628185348862145000000

extensive a broad 0.00016211561998687000000

extensive a wide 0.11739021568133400000000

extensive a massive 0.04017327755038210000000

extensive a pervasive 0.00048476758325355100000

extensive a complete 0.03949507952696630000000

extensive a widespread 0.23927629601330000000000

extensive a gigantic 0.00002984295834535000000

extensive a general 0.00039120535894546300000

extensive a exhaustive 0.00000458505356123286000

extensive a nationwide 0.00003714227953862350000

extensive a vast 0.02812779758564900000000

extensive a radical 0.00201647738412138000000

extensive a considerable 0.02742849323074490000000

extensive a exclusive 0.03384239172874880000000

extensive a liberal 0.00079391163243252000000

extensive a comprehensive 0.00435478651153277000000

extensive a enormous 0.03135910629407980000000

extensive a ample 0.00023667010679210000000

extensive a large 0.03039047860000740000000

extensive a catholic 0.00000229252678061643000

extensive a infinite 0.00000229252678061643000

extensive a long 0.01647582193800890000000

extensive a big 0.02833823268835040000000

extensive a sustained 0.00005893048320452700000

extensive a sweeping 0.00467649288500166000000

extensive a extended 0.00006637245963337660000

extensive a lengthy 0.00692461495239287000000

extensive a generous 0.00098216382550859500000

extensive a large-scale 0.05702689438681060000000

extensive a unlimited 0.00000229252678061643000

extensive a prolonged 0.00005893048320452700000

extensive a global 0.05952998750051610000000

extensive a stupendous 0.00005510086312946700000

extensive a universal 0.00003901306546781590000

extensive a wide-ranging 0.03661547356441280000000

extensive a thorough 0.00029694494280325200000

extensive a protracted 0.00013817276179035800000

offense n atrocity 0.00071246215044825700000

offense n crime 0.06602149260820520000000

offense n offence 0.93255358309089800000000

offense n attack 0.00035623107522412900000

offense n fault 0.00035623107522412900000

muffler n stock 1.00000000000000000000000

preeclampsia n pre-eclampsia 1.00000000000000000000000

eligible a licensed 0.00002155052676386460000

eligible a worthwhile 0.00017734829136973500000

eligible a worthy 0.00001077526338193230000

eligible a privileged 0.00004310105352772980000

eligible a preferable 0.00013157618971894800000

eligible a appropriate 0.00535327482182608000000

eligible a likely 0.28497640807673900000000

eligible a usable 0.00006578809485947390000

eligible a acceptable 0.01218851307951100000000

eligible a capable 0.00123597108092797000000

eligible a satisfactory 0.00345635210011979000000

eligible a proper 0.00032928360467009000000

eligible a available 0.08421074969837040000000

eligible a single 0.00809636357879494000000

eligible a suitable 0.00067198063329813900000

eligible a able 0.57732494020588500000000

eligible a prime 0.00124497697145288000000

eligible a desirable 0.00291913297042628000000

eligible a fit 0.01754191375835720000000

manipulator n driver 0.32890261977813900000000

manipulator n performer 0.41943586263866300000000

manipulator n operator 0.25166151758319800000000

soften v melt 0.00002751171347014900000

soften v mute 0.01380333885136470000000

soften v allay 0.00000281812684292842000

soften v abate 0.00032654202273275700000

soften v yield 0.00000204211936489710000

soften v mitigate 0.00000196043529675801000

soften v lower 0.00137119334881887000000

soften v appease 0.00000302754612499018000

soften v deliberate 0.00000491565191800091000

soften v slacken 0.00004725968077707990000

soften v qualify 0.00006791944753361920000

soften v blunt 0.00006276229650897110000

soften v weaken 0.48000502053376500000000

soften v silence 0.00002360790212587130000

soften v disintegrate 0.00003472296639510030000

soften v tame 0.00000281812684292842000

soften v touch 0.00132893478544665000000

soften v agree 0.00114069135719371000000

soften v move 0.03122757692372870000000

soften v subdue 0.00570633381580281000000

soften v throttle 0.00000302754612499018000

soften v dampen 0.00011038992442656100000

soften v relax 0.01920044470362470000000

soften v modify 0.00007950440102530190000

soften v mollify 0.00003415605181765670000

soften v reduce 0.00662465995955560000000

soften v dissolve 0.00001925259736313970000

soften v calm 0.00004537883526918050000

soften v moderate 0.00098024084331999300000

soften v still 0.00000453370530231227000

soften v relieve 0.00003096340403573470000

soften v diminish 0.01394512542377000000000

soften v break 0.00032731184784570400000

soften v mellow 0.00003620541492671600000

soften v alleviate 0.00000641245653093121000

soften v affect 0.02005575411499320000000

soften v ease 0.39297673361527100000000

soften v quell 0.00000469687807154741000

soften v give 0.01019662346959020000000

soften v compose 0.00000453370530231227000

soften v temper 0.00003236551894798250000

soften v disarm 0.00001204871021678810000

soften v loosen 0.00006805042306222850000

soften v stifle 0.00000605509224998036000

soften v enfeeble 0.00000453370530231227000

kindle v raise 1.00000000000000000000000

concede v relinquish 0.00009061690143538640000

concede v deliver 0.00113220132925038000000

concede v yield 0.00022609015394688400000

concede v renounce 0.00001390138884364170000

concede v grant 0.04975816676993240000000

concede v resign 0.00295373920175373000000

concede v admit 0.23975505797614300000000

concede v recognize 0.08318023899683990000000

concede v transfer 0.01405499832267040000000

concede v abandon 0.00086517367306007200000

concede v allow 0.10337429105024400000000

concede v acknowledge 0.03397008001732360000000

concede v permit 0.00120923395863636000000

concede v agree 0.17554593050664000000000

concede v quit 0.00022696993955200900000

concede v tender 0.00036323404935455500000

concede v capitulate 0.00002780277768728340000

concede v release 0.01181011657167690000000

concede v surrender 0.03822442838234510000000

concede v own 0.00197967103025624000000

concede v donate 0.00075715936671811600000

concede v confess 0.00011459371767528600000

concede v accept 0.16328258914535400000000

concede v give 0.07555567467508780000000

concede v waive 0.00091806551854889200000

concede v suffer 0.00060997457902286100000

intimately r well 0.50000000000000000000000

intimately r fully 0.50000000000000000000000

hefty a huge 0.06629297287992490000000

hefty a hard 0.00181150462644272000000

hefty a respectable 0.00472207908609938000000

hefty a sizable 0.00022794568852694700000

hefty a whopping 0.02089739380881730000000

hefty a substantial 0.03333800822297150000000

hefty a strong 0.12389541074139400000000

hefty a powerful 0.00004530436582342230000

hefty a solid 0.06774954998244470000000

hefty a major 0.00835175811370301000000

hefty a goodish 0.00000772052183769506000

hefty a healthy 0.08740348666116180000000

hefty a sturdy 0.00025531907907057700000

hefty a massive 0.04396435494914670000000

hefty a robust 0.04658459957296170000000

hefty a weighty 0.00949881536764423000000

hefty a enormous 0.00474940768382211000000

hefty a ample 0.00452582214921699000000

hefty a large 0.09850597147750830000000

hefty a big 0.09620678307846970000000

hefty a hearty 0.00000772052183769506000

hefty a stiff 0.00949881536764423000000

hefty a heavy 0.24546197488185800000000

hefty a sizeable 0.02325249361413870000000

hefty a fat 0.00237470384191106000000

hefty a vigorous 0.00037008371562250200000

immortalise v immortalize 1.00000000000000000000000

setback n stalemate 0.00007642404750037120000

setback n glitch 0.00379416039948808000000

setback n failure 0.00422890519431196000000

setback n block 0.00015726262200483700000

setback n hitch 0.00543553232602472000000

setback n recurrence 0.00000242185125901765000

setback n mishap 0.00080526478711169500000

setback n hiccup 0.00012694615878228200000

setback n trouble 0.00761522888646643000000

setback n difficulty 0.00843584820320093000000

setback n defeat 0.03879977233969700000000

setback n hindrance 0.00007090869704532420000

setback n problem 0.19558275820821500000000

setback n check 0.00040210805230430800000

setback n blow 0.23424543038623800000000

setback n undoing 0.00037055167182381400000

setback n relapse 0.00137584148867268000000

setback n disappointment 0.00316598091712311000000

setback n reversal 0.00040447730970447000000

setback n abortion 0.00014615737807546500000

setback n reverse 0.00014350901334537700000

setback n rejection 0.00003472490150822930000

setback n deadlock 0.00572554548002723000000

setback n loss 0.46134062660643700000000

setback n frustration 0.00002600374291803470000

setback n complication 0.00009034206493813090000

setback n upset 0.02739726726577630000000

hospitalization n hospitalisation 1.00000000000000000000000

hardy a strong 0.97477214670601100000000

hardy a vigorous 0.02522785329398870000000

session n caucus 0.00001968694883000970000

session n assembly 0.00184204812219356000000

session n meeting 0.12733952114903500000000

session n period 0.02674471131575290000000

session n term 0.01713387225560440000000

session n sitting 0.00001651344152507130000

session n year 0.78570977985761900000000

session n time 0.04119386690944030000000

flesh v fill 1.00000000000000000000000

fancy n sentiment 0.00743701775587989000000

fancy n opinion 0.00148740355117597000000

fancy n idea 0.00074370177558798300000

fancy n inclination 0.00148740355117597000000

fancy n sense 0.00446221065352793000000

fancy n mood 0.01115552663381980000000

fancy n fad 0.92488612066561300000000

fancy n feeling 0.01859254438969970000000

fancy n liking 0.00074370177558798300000

fancy n belief 0.00223110532676397000000

fancy n perception 0.00892442130705587000000

fancy n concept 0.00148740355117597000000

fancy n impression 0.00371850887793995000000

fancy n trend 0.00148740355117597000000

fancy n tendency 0.00074370177558798300000

fancy n view 0.00892442130705587000000

fancy n yen 0.00148740355117597000000

fanciful a strange 0.00074749450180706700000

fanciful a crazy 0.00747494501807076000000

fanciful a erratic 0.00074749450180706700000

fanciful a irresponsible 0.18214048583784400000000

fanciful a fantastic 0.70751146291815000000000

fanciful a incredible 0.00149498900361413000000

fanciful a unpredictable 0.02246319939672110000000

fanciful a notional 0.00151231366516842000000

fanciful a ridiculous 0.03730256379601860000000

fanciful a odd 0.00520451945627246000000

fanciful a absurd 0.00854328031604887000000

fanciful a frivolous 0.00074749450180706700000

fanciful a fictitious 0.00074749450180706700000

fanciful a impractical 0.00074749450180706700000

fanciful a preposterous 0.01507052441876880000000

fanciful a grotesque 0.00074749450180706700000

fanciful a ideal 0.00679674916248075000000

face-off n showdown 1.00000000000000000000000

harbor n mole 0.00000077255272055469700

harbor n anchorage 0.27665129769825500000000

harbor n port 0.12518404903748600000000

harbor n haven 0.03163709616670600000000

harbor n retreat 0.00017542974574581100000

harbor n shelter 0.00009833274660902560000

harbor n harbour 0.56535936396744800000000

harbor n pier 0.00089327180866858400000

harbor n wharf 0.00000038627636027734800

disbursal n expense 0.81882995308690600000000

disbursal n spending 0.18117004691309500000000

crossover n crossing 1.00000000000000000000000

complainant n prosecutor 0.80776440931571900000000

complainant n plaintiff 0.19223559068428100000000

circumstantial a particular 0.04614782948768090000000

circumstantial a indirect 0.95385217051231900000000

chore n job 1.00000000000000000000000

receipts n earnings 0.41443611646550400000000

receipts n gross 0.00240371618150760000000

receipts n proceeds 0.03161992282967150000000

receipts n revenue 0.19780796309259700000000

receipts n gain 0.10141240696526000000000

receipts n gate 0.00000559608987980237000

receipts n royalty 0.00008178685659970160000

receipts n net 0.03439387565246410000000

receipts n return 0.00335123702791930000000

receipts n profit 0.15045924647671300000000

receipts n income 0.06402813236188440000000

pennsylvania n penn 0.51383150594159400000000

pennsylvania n pa 0.48616849405840600000000

plaintiff n complainant 1.00000000000000000000000

ass n butt 0.99977810650887600000000

ass n seat 0.00022189349112425900000

argosy n abundance 0.00020369062575696800000

argosy n fleet 0.00015924488762457600000

argosy n navy 0.00056662613913851200000

argosy n trader 0.99907043834748000000000

approximate v approach 0.00080163984571948900000

approximate v judge 0.00040081992285974500000

approximate v estimate 0.99879754023142100000000

rating n assignment 0.00510750055810525000000

rating n consignment 0.00023673276346846800000

rating n classification 0.00015648232740582300000

rating n grade 0.04057961791870620000000

rating n number 0.05740792825092130000000

rating n mark 0.04486794364368470000000

rating n class 0.45106033552606000000000

rating n designation 0.00136044908494125000000

rating n score 0.00802346336307382000000

rating n reprimand 0.00022531766748365800000

rating n assessment 0.02583386341186190000000

rating n sort 0.00286793832677209000000

rating n category 0.15592917200573100000000

rating n rank 0.00048360357033515100000

rating n valuation 0.05840124843769910000000

rating n position 0.10676381185678200000000

rating n reproach 0.00262292623082963000000

rating n evaluation 0.00269232533463784000000

rating n ranking 0.03537791778932500000000

rating n relegation 0.00000142193217566892000

gathering n company 0.02255464059683580000000

gathering n dance 0.00001638997195965070000

gathering n luncheon 0.00443505950035073000000

gathering n senate 0.00048169365325933300000

gathering n union 0.00495779365258478000000

gathering n caucus 0.00001099128438208810000

gathering n discussion 0.00999795192325585000000

gathering n cluster 0.00000115894704255187000

gathering n assembly 0.00228273277019203000000

gathering n rout 0.00001271871780012380000

gathering n reunion 0.00001209426089730180000

gathering n accumulation 0.00000062986204943831700

gathering n number 0.00076167967951775900000

gathering n meeting 0.60931675858061300000000

gathering n social 0.00012984923521521700000

gathering n panel 0.00252880861045546000000

gathering n society 0.00004932182844810370000

gathering n banquet 0.00022635880192923000000

gathering n mound 0.00000604713044865090000

gathering n band 0.00022316002023132900000

gathering n heap 0.00000115894704255187000

gathering n crew 0.00013056725269617700000

gathering n ball 0.00006324877841766990000

gathering n crowd 0.00842801982482918000000

gathering n bloc 0.00016085709455062700000

gathering n mass 0.00009870568811237970000

gathering n combination 0.00034769166475061100000

gathering n house 0.02183801960591740000000

gathering n stock 0.00593507219303100000000

gathering n forum 0.01787061934434180000000

gathering n convention 0.00087691461310951300000

gathering n group 0.01783795051715120000000

gathering n conference 0.12985056748078800000000

gathering n legislature 0.00011584466925581800000

gathering n tea 0.00000463578817020749000

gathering n collection 0.00000178880909199019000

gathering n parliament 0.00774893451507272000000

gathering n levy 0.00035227122679557200000

gathering n convergence 0.00001469346609014300000

gathering n host 0.00003808266255438910000

gathering n committee 0.00314690102058226000000

gathering n function 0.00908968568859848000000

gathering n council 0.00690225869363850000000

gathering n audience 0.00151433410454342000000

gathering n gang 0.00000579473521275943000

gathering n moot 0.00002866608900643990000

gathering n attendance 0.00000652537431852248000

gathering n circle 0.00000115894704255187000

gathering n cloud 0.00000231789408510375000

gathering n association 0.00058550089885931600000

gathering n dinner 0.00160403689021203000000

gathering n turnout 0.00000672692843117207000

gathering n reception 0.01261857710607070000000

gathering n confab 0.00304555478959328000000

gathering n congestion 0.00000115894704255187000

gathering n school 0.00014773739234409300000

gathering n pack 0.00000178880909199019000

gathering n press 0.00016857039756231500000

gathering n congress 0.03768321759168020000000

gathering n batch 0.00001722126594526700000

gathering n rally 0.03255038143800070000000

gathering n crush 0.00000463578817020749000

gathering n community 0.00350964679635635000000

gathering n coffee 0.00544681257901539000000

gathering n party 0.01001806607400260000000

gathering n herd 0.00000115894704255187000

gathering n array 0.00000116307119447041000

gathering n body 0.00191530577803224000000

gathering n store 0.00024787095198951800000

gathering n muster 0.00000115894704255187000

gathering n queue 0.00000342394901236793000

gathering n junta 0.00000115894704255187000

preserve v continue 0.02773847423903460000000

preserve v bottle 0.00001119508568773650000

preserve v shelve 0.00023808038800877700000

preserve v protect 0.14697975210830800000000

preserve v lengthen 0.00000329309371950338000

preserve v cure 0.00004065083881256720000

preserve v defend 0.02411899833452440000000

preserve v save 0.07817164810558240000000

preserve v secure 0.00050606989657630400000

preserve v retain 0.00389891776216329000000

preserve v nurse 0.00000329309371950338000

preserve v fill 0.00225709673246283000000

preserve v sustain 0.00465048466205317000000

preserve v keep 0.15752151671947700000000

preserve v process 0.00023556798549546100000

preserve v freeze 0.00065252897801340600000

preserve v hide 0.00015309126967706400000

preserve v refrigerate 0.00012692241813710100000

preserve v rescue 0.00023622684786208900000

preserve v guard 0.00003002736078638940000

preserve v jar 0.00000470152682386651000

preserve v uphold 0.08805841325054850000000

preserve v reserve 0.00025525987549332100000

preserve v store 0.00006993400273128910000

preserve v doctor 0.00001646546859751710000

preserve v dry 0.00000329309371950338000

preserve v support 0.02117531243447850000000

preserve v safeguard 0.07580777591320660000000

preserve v cover 0.00389403902006602000000

preserve v spare 0.00038577668125241500000

preserve v maintain 0.30827137534804000000000

preserve v hold 0.04780967653075220000000

preserve v prolong 0.00006366203698442870000

preserve v smoke 0.00000329309371950338000

preserve v screen 0.00002456221922117950000

preserve v can 0.00382438945074220000000

preserve v extend 0.00275295435893757000000

preserve v bank 0.00000527977458359236000

patrol n patrolling 0.62940820297990900000000

patrol n ranger 0.00000841289010148308000

patrol n vigil 0.00000218211357782125000

patrol n protection 0.04915917072153090000000

patrol n guard 0.03086771779351110000000

patrol n bull 0.00016203854710688600000

patrol n force 0.23123781356050400000000

patrol n squad 0.00004206445050741550000

patrol n constable 0.00009471060981575190000

patrol n policeman 0.00051449643272789300000

patrol n detachment 0.00069315677073945900000

patrol n watchdog 0.00002087444314880670000

patrol n watch 0.00000623077652366183000

patrol n officer 0.02418390342812480000000

patrol n guardian 0.00004476739967160450000

patrol n defense 0.00180034715086890000000

patrol n party 0.02272822824539300000000

patrol n bodyguard 0.00000841289010148308000

patrol n garrison 0.00901726879613663000000

yearly n annual 1.00000000000000000000000

successive a consecutive 0.88772089157047700000000

successive a subsequent 0.00873885260608772000000

successive a continuous 0.02009936099400180000000

successive a next 0.08093313342842640000000

successive a following 0.00250776140100734000000

settler n founder 0.04160003435297170000000

settler n immigrant 0.36343303526204500000000

settler n pioneer 0.24786000379440300000000

settler n pilgrim 0.28034704399593200000000

settler n newcomer 0.06675988259464830000000

seclusion n retirement 1.00000000000000000000000

angry a threatening 0.00441560476540139000000

angry a upset 0.06534597034987640000000

angry a uptight 0.00001067296981574180000

angry a over-excited 0.00038192333735719100000

angry a frantic 0.00001067296981574180000

angry a sore 0.00014278803538218000000

angry a passionate 0.00362811158793936000000

angry a strong 0.46967562754114700000000

angry a outraged 0.00084014355768123500000

angry a volatile 0.07118752964130040000000

angry a windy 0.00099451196589070700000

angry a touchy 0.00253166014218455000000

angry a violent 0.04983434801325000000000

angry a restless 0.00023830165364458500000

angry a hostile 0.00170839585336802000000

angry a bitter 0.02529524781056720000000

angry a annoyed 0.00472065286064252000000

angry a fierce 0.00541437380693383000000

angry a mad 0.00359873760774679000000

angry a wild 0.00485129593605819000000

angry a resentful 0.00001067296981574180000

angry a rough 0.00661691786392712000000

angry a livid 0.00002525783319609210000

angry a contentious 0.00415274351850946000000

angry a surly 0.00051182186694373200000

angry a heated 0.01589443198987710000000

angry a furious 0.25796158355172800000000

ruinous a damaging 0.62500000000000000000000

ruinous a fatal 0.25000000000000000000000

ruinous a disastrous 0.12500000000000000000000

recognise v recognize 0.98051310894618700000000

recognise v acknowledge 0.00054957679599947300000

recognise v know 0.01893731425781390000000

quiet v dampen 0.18629550321199100000000

quiet v cool 0.12847965738758000000000

quiet v settle 0.68522483940042800000000

operate v achieve 0.00499480515926885000000

operate v fulfill 0.00001451118606682990000

operate v fly 0.04675639735470100000000

operate v apply 0.00406208558098537000000

operate v compel 0.00000739760986579245000

operate v lift 0.00113361157903848000000

operate v concern 0.00046215150121262500000

operate v determine 0.00069625257738927500000

operate v buy 0.01041668137608020000000

operate v act 0.01560366025828990000000

operate v utilize 0.00118108960287335000000

operate v use 0.04543199954510850000000

operate v contain 0.00012363881134649900000

operate v benefit 0.00252569783742900000000

operate v conduct 0.00804148479268147000000

operate v spark 0.00005032208694955330000

operate v promote 0.00019020824513424900000

operate v handle 0.00274435242957913000000

operate v transact 0.00000306035929367580000

operate v perform 0.00116248818165893000000

operate v advance 0.00030342699918762600000

operate v manufacture 0.00409661261467757000000

operate v succeed 0.00109517942419774000000

operate v ply 0.00190679247396360000000

operate v employ 0.00299887709996280000000

operate v sustain 0.00073982562982692900000

operate v react 0.00053339193664144600000

operate v proceed 0.00725438742823324000000

operate v engage 0.00264748288331189000000

operate v roll 0.00315199136198589000000

operate v move 0.00621963646942403000000

operate v work 0.14301384738723600000000

operate v influence 0.00051681000207194600000

operate v cut 0.00745373482922814000000

operate v contact 0.00012748974322105700000

operate v explode 0.00010295794247655000000

operate v lock 0.00010863821052154200000

operate v explore 0.00050111558130290900000

operate v amputate 0.00001547041987273840000

operate v execute 0.00033316917386011600000

operate v direct 0.00279992335604257000000

operate v burn 0.00005907253694281030000

operate v enforce 0.00019484038528174900000

operate v run 0.37660646766283500000000

operate v hit 0.00198799266382008000000

operate v pump 0.00620409665818082000000

operate v transport 0.00008810737594305460000

operate v produce 0.13017114368447900000000

operate v progress 0.00035061371104745100000

operate v constitute 0.00000524786497369736000

operate v spin 0.00002450590082817350000

operate v guide 0.00000242099339430800000

operate v construct 0.00362175990552413000000

operate v turn 0.00372548691748230000000

operate v manage 0.02464580582482040000000

operate v administer 0.00231320166295517000000

operate v go 0.02065987587838710000000

operate v complete 0.01862012496320660000000

operate v engineer 0.00000210485030368055000

operate v manipulate 0.00019086008361036400000

operate v accomplish 0.00041549962647336700000

operate v behave 0.00053297444486471000000

operate v exert 0.00015193299235407100000

operate v function 0.02407132545214110000000

operate v effect 0.00003870187535064170000

operate v finish 0.00124124507921247000000

operate v sell 0.02120943326370670000000

operate v control 0.01284235867248600000000

operate v serve 0.01033345384694390000000

operate v drive 0.00623243378106785000000

operate v convey 0.00187037377554945000000

operate v practice 0.00006388062363412270000

wireless n radio 1.00000000000000000000000

prom n dance 1.00000000000000000000000

grower n breeder 0.00005948143033026420000

grower n raiser 0.00000600502986865829000

grower n producer 0.95425440935601900000000

grower n cultivator 0.04568010418378240000000

chapter n company 0.29560344592348500000000

chapter n event 0.00812745757752694000000

chapter n episode 0.00000646664373249238000

chapter n cluster 0.00000215554791083079000

chapter n assembly 0.00160884360271688000000

chapter n part 0.00549252775506714000000

chapter n branch 0.01240849197958980000000

chapter n age 0.00000215554791083079000

chapter n period 0.02309554219135720000000

chapter n phase 0.00245012932062408000000

chapter n article 0.00110713137342221000000

chapter n section 0.00000215554791083079000

chapter n arrangement 0.02589949018178960000000

chapter n convention 0.00215722031869267000000

chapter n group 0.10635443745608800000000

chapter n era 0.06354933266322580000000

chapter n council 0.00001939993119747730000

chapter n department 0.00048067931715209500000

chapter n gathering 0.00000862219164332318000

chapter n passage 0.00000215554791083079000

chapter n grouping 0.00000215554791083079000

chapter n division 0.00001077773955415410000

chapter n time 0.06070528391038730000000

chapter n body 0.00014226616211483400000

chapter n page 0.14539278435949400000000

chapter n stage 0.24536889166158500000000

open-minded a sympathetic 0.04614975862593820000000

open-minded a objective 0.04614975862593820000000

open-minded a equitable 0.04614975862593820000000

open-minded a fair 0.06457999967119770000000

open-minded a receptive 0.09229951725187640000000

open-minded a neutral 0.04614975862593820000000

open-minded a good 0.13939478424448300000000

open-minded a reasonable 0.23648845285137600000000

open-minded a liberal 0.04614975862593820000000

open-minded a catholic 0.00573965972168465000000

open-minded a open 0.23074879312969100000000

tiny a negligible 0.00009059397988322920000

tiny a minute 0.00002056359616269460000

tiny a insignificant 0.00004931578213631770000

tiny a paltry 0.00289694170502521000000

tiny a slight 0.00101556857491578000000

tiny a diminutive 0.00039419922935754200000

tiny a minor 0.00011842163011802600000

tiny a miniature 0.04953770315593120000000

tiny a miniscule 0.00990754063118624000000

tiny a small 0.91775895680677700000000

tiny a little 0.01820651918667260000000

tiny a delicate 0.00000367572183393419000

miscarriage n failure 0.99014424310867000000000

miscarriage n fiasco 0.00015399620142703000000

miscarriage n accident 0.00200195061855140000000

miscarriage n malfunction 0.00015399620142703000000

miscarriage n misfortune 0.00015399620142703000000

miscarriage n casualty 0.00246393922283250000000

miscarriage n error 0.00092397720856218600000

miscarriage n mistake 0.00200195061855140000000

miscarriage n undoing 0.00030799240285405900000

miscarriage n disappointment 0.00107797340998922000000

miscarriage n abortion 0.00030799240285405900000

miscarriage n frustration 0.00030799240285405900000

run-up n runup 1.00000000000000000000000

litigate v charge 0.00783305319042742000000

litigate v claim 0.00052232045290742300000

litigate v process 0.00275317981637794000000

litigate v contest 0.00585060301161269000000

litigate v dispute 0.00006529005661342740000

litigate v prosecute 0.00242672953331080000000

litigate v accuse 0.00084877073597456400000

litigate v maintain 0.02164469562995700000000

litigate v hold 0.95805535757281900000000

fortune n fortuna 0.00001202459966788760000

fortune n level 0.00683697262548915000000

fortune n treasure 0.00002001335744953630000

fortune n pile 0.00000697552606661578000

fortune n packet 0.00001648499343510970000

fortune n capital 0.00472239119266730000000

fortune n end 0.00614522470963336000000

fortune n grade 0.00162412271260647000000

fortune n success 0.05296521592397690000000

fortune n mint 0.00025560462302820500000

fortune n providence 0.00002516473091118280000

fortune n class 0.00001592786127162420000

fortune n uncertainty 0.04316704328639160000000

fortune n coincidence 0.00003487763033307930000

fortune n status 0.00005492539843304610000

fortune n revenue 0.05290038866858890000000

fortune n worth 0.00019114647294748100000

fortune n resources 0.00000352836401442661000

fortune n heap 0.00000176418200721330000

fortune n money 0.19322136049211200000000

fortune n accident 0.00219104261941264000000

fortune n stock 0.01969259158004940000000

fortune n standing 0.00296200295993480000000

fortune n promise 0.00000176418200721330000

fortune n means 0.00002092657819984760000

fortune n destination 0.01486206991192170000000

fortune n portion 0.00741310084935761000000

fortune n decree 0.00118348761235520000000

fortune n rank 0.05135301621044480000000

fortune n position 0.04992492225097080000000

fortune n affluence 0.00001416367926441090000

fortune n fate 0.00003009154053603520000

fortune n judgment 0.00031387666986283100000

fortune n anticipation 0.00000529254602163991000

fortune n lot 0.02856285431767070000000

fortune n wealth 0.00737370003838832000000

fortune n condition 0.01248379131857090000000

fortune n place 0.00036846586063146900000

fortune n determination 0.00000705672802885322000

fortune n story 0.00132146923535462000000

fortune n life 0.02397508224607800000000

fortune n property 0.08901095110076010000000

fortune n substance 0.00003659265698398730000

fortune n expectation 0.00699696206241256000000

fortune n prosperity 0.10210958648725300000000

fortune n situation 0.02037822081912380000000

fortune n income 0.04128785410138250000000

fortune n goal 0.01928300626849510000000

fortune n cup 0.00057387716700572900000

fortune n luck 0.02191389815587150000000

fortune n die 0.00000352836401442661000

fortune n chance 0.10915533064116400000000

fortune n station 0.00170656939295628000000

fortune n footing 0.00126169649648416000000

siemens n s 1.00000000000000000000000

manager n skipper 0.00015695319170226400000

manager n superintendent 0.00000181803776106385000

manager n agent 0.01938494258930790000000

manager n leader 0.00587448343104477000000

manager n handler 0.00001040649209131600000

manager n mastermind 0.00000163525534333858000

manager n conductor 0.00000031887015927162200

manager n overseer 0.00000045047716900274700

manager n coach 0.20356032613682200000000

manager n pilot 0.00081815186301354900000

manager n mayor 0.00069930949178566500000

manager n adviser 0.00571470799238129000000

manager n foreman 0.00000380804269528464000

manager n organizer 0.00199662154233250000000

manager n director 0.24057528777504400000000

manager n treasurer 0.00134414646358352000000

manager n executive 0.05565854110936420000000

manager n head 0.12720777226023300000000

manager n husband 0.00002114303377401540000

manager n boss 0.05183869197170100000000

manager n principal 0.00150793676468917000000

manager n helmsman 0.00000105580036035910000

manager n official 0.21512785112969700000000

manager n master 0.00021593341701905000000

manager n chief 0.04587217991574560000000

manager n administrator 0.00244934412814358000000

manager n producer 0.00243622380211886000000

manager n officer 0.00730930698783337000000

manager n controller 0.00005105855508396180000

manager n guide 0.00384809622577267000000

manager n bailiff 0.00000031887015927162200

manager n factor 0.00069056259882323500000

manager n comptroller 0.00286350070789409000000

manager n supervisor 0.00004795545410646600000

manager n governor 0.00270915961524304000000

eye v regard 0.00006537810766862600000

eye v watch 0.40120919923332400000000

eye v view 0.00000668614887490766000

eye v study 0.05451338118429070000000

eye v scrutinize 0.00294007430988741000000

eye v survey 0.00001777094955076220000

eye v consider 0.36852189689061300000000

eye v see 0.17267212398479200000000

eye v observe 0.00002674459549963080000

eye v inspect 0.00002674459549963080000

switch n button 0.00000802947295542680000

switch n changeover 0.00107467326559970000000

switch n transfer 0.00502483648802829000000

switch n change 0.19923071543502900000000

switch n conversion 0.00452687869265928000000

switch n transition 0.08473110756466700000000

switch n shift 0.01807581934449850000000

switch n replacement 0.04132542252236500000000

switch n move 0.54929369558747000000000

switch n turnaround 0.00064143270791713200000

switch n belt 0.00002622728235493650000

switch n control 0.01601536890322230000000

switch n reversal 0.00050534278518015600000

switch n transformation 0.00000909890469975471000

switch n cat 0.00001712837765518150000

switch n stick 0.00016909783602692200000

switch n switching 0.07932512482967100000000

greatness n size 1.00000000000000000000000

grace v favor 0.07692307692307580000000

grace v honor 0.92307692307692400000000

glee n fun 1.00000000000000000000000

foetal a fetal 1.00000000000000000000000

engineer n fireman 0.00287257693313670000000

engineer n mechanic 0.14063507330712000000000

engineer n handler 0.00000826858845787255000

engineer n conductor 0.02574007503682220000000

engineer n pilot 0.02189798100001260000000

engineer n driver 0.29608030754207400000000

engineer n pioneer 0.00075876500290802400000

engineer n designer 0.00828234265914253000000

engineer n controller 0.00024675509493123600000

engineer n builder 0.06640756339569740000000

engineer n technician 0.43453652709218200000000

engineer n operator 0.00253376434751445000000

detonation n explosion 0.99475472688696200000000

detonation n blast 0.00437106092753190000000

detonation n firing 0.00087421218550638100000

fare n passenger 0.03467924858214380000000

fare n price 0.52537352059635900000000

fare n fee 0.02864724554737500000000

fare n menu 0.00000085286536231203000

fare n cost 0.08504086654147160000000

fare n tariff 0.03315842507693630000000

fare n table 0.00009347796896710020000

fare n transportation 0.00516260709889144000000

fare n board 0.00282413389464783000000

fare n toll 0.00074719333002920600000

fare n check 0.00009042102018087280000

fare n charge 0.00178690194228026000000

fare n ticket 0.22678370331972400000000

fare n expense 0.00977798293412505000000

fare n food 0.02366074924679740000000

fare n passage 0.00007953798599823830000

fare n admission 0.00032224028637474100000

fare n payment 0.00078513834766864200000

fare n rental 0.00002816536673020120000

fare n rider 0.00000445733235941725000

fare n diet 0.02043252191759090000000

fare n book 0.00052060879798691600000

collectible a payable 1.00000000000000000000000

massacre n bloodbath 0.00003509311748284760000

massacre n slaughter 0.04904772393714390000000

massacre n killing 0.28282362258137100000000

massacre n liquidation 0.00001133672100528560000

massacre n genocide 0.64546201823823500000000

massacre n holocaust 0.02262020540476180000000

cavalcade n company 0.23833234767432700000000

cavalcade n train 0.01833325751340980000000

cavalcade n march 0.74333439481226300000000

cautiousness n caution 1.00000000000000000000000

flexible a ready 0.07071403526752450000000

flexible a conciliatory 0.08390607527504970000000

flexible a resilient 0.00304687037767724000000

flexible a manageable 0.00766109100392765000000

flexible a stretchable 0.37950355791262700000000

flexible a adjustable 0.00119428513826070000000

flexible a soft 0.00654280696539593000000

flexible a gentle 0.04630108767561460000000

flexible a willing 0.08446930198226190000000

flexible a receptive 0.00526446233492525000000

flexible a easy 0.01691328613413950000000

flexible a mild 0.00461489722725898000000

flexible a amenable 0.00027059077213021500000

flexible a compliant 0.02334264890382230000000

flexible a elastic 0.00190227347324625000000

flexible a agreeable 0.00002112093264670740000

flexible a variable 0.07246522251178760000000

flexible a obedient 0.00008462561220038460000

flexible a cooperative 0.00347846643616792000000

flexible a open 0.18729733542285800000000

flexible a plastic 0.00002625731637608300000

flexible a open-ended 0.00097970132410213900000

buff n enthusiast 0.66666666666666700000000

buff n fan 0.33333333333333300000000

because r therefore 0.60606060606060600000000

because r consequently 0.39393939393939400000000

smoking n smoke 1.00000000000000000000000

antidote n solution 1.00000000000000000000000

animate v inform 1.00000000000000000000000

afghani n afghan 1.00000000000000000000000

absolve v clear 0.84693877551020400000000

absolve v acquit 0.15306122448979600000000

background n ground 0.00070654447433439500000

background n environment 0.13791979113568000000000

background n education 0.06063087513498230000000

background n history 0.00548448239074968000000

background n class 0.00271409439576589000000

background n past 0.25564407248065300000000

background n offing 0.00334762716423838000000

background n training 0.00044010165804632100000

background n experience 0.00208295847245120000000

background n culture 0.03275490471484690000000

background n preparation 0.00104672349840547000000

background n scene 0.00032492676266770600000

background n framework 0.00013943615032395600000

background n record 0.12000234074455900000000

background n distance 0.00012329178070208300000

background n scope 0.00142971710843507000000

background n tradition 0.00034476499115216900000

background n horizon 0.12117883178473200000000

background n backdrop 0.19268359957963900000000

background n setting 0.01559369702259340000000

background n stage 0.04540721855504220000000

polish n class 0.01432648487110550000000

polish n sheen 0.03881471457460460000000

polish n culture 0.04295395936001910000000

polish n grace 0.90390484119427100000000

packer n boxer 1.00000000000000000000000

wintry a stormy 0.54760815234608800000000

wintry a frigid 0.09926431764581650000000

wintry a cold 0.26850645922067000000000

wintry a overcast 0.03970572705832660000000

wintry a dismal 0.04491534372909990000000

wilful a intentional 0.07692307692307690000000

wilful a willful 0.92307692307692300000000

vocabulary n language 1.00000000000000000000000

compound n union 0.01770833971824310000000

compound n prison 0.04423055432356710000000

compound n camp 0.09785508127020180000000

compound n village 0.11001366401293700000000

compound n consolidation 0.00253245522218596000000

compound n combine 0.00005916793150681270000

compound n combination 0.00533498147769120000000

compound n court 0.59568744337515400000000

compound n colony 0.00476163784068497000000

compound n square 0.00200668396385654000000

compound n enclosure 0.00750148163202501000000

compound n encampment 0.00084838185124092300000

compound n mix 0.00267129119836962000000

compound n ring 0.00175936832257346000000

compound n merger 0.04030911557911250000000

compound n aggregate 0.00002514315259469700000

compound n settlement 0.06663260450796330000000

compound n homes 0.00006260462009161140000

tyrant n king 1.00000000000000000000000

bracket n rest 0.02875223982650400000000

bracket n grade 0.19671311482949100000000

bracket n class 0.05618513684585940000000

bracket n link 0.00046157062990739100000

bracket n kind 0.04672654786119710000000

bracket n section 0.00075482805579179500000

bracket n group 0.36742262096011500000000

bracket n order 0.01344521720343160000000

bracket n sort 0.00607576775262187000000

bracket n category 0.02386775921087850000000

bracket n support 0.10236170723483000000000

bracket n rank 0.00023924003621942600000

bracket n genus 0.00016452553656525000000

bracket n grouping 0.14731205230211100000000

bracket n division 0.00016452553656525000000

bracket n station 0.00935314617791183000000

townspeople n town 1.00000000000000000000000

perception n conscience 0.00000396830448841870000

perception n idea 0.13839709845641800000000

perception n sensation 0.00000793660897683754000

perception n instinct 0.00003930401055914370000

perception n consciousness 0.00004902650645117990000

perception n apprehension 0.00001634216881706000000

perception n sense 0.02740059766598220000000

perception n image 0.01985220244471780000000

perception n intelligence 0.00001634216881706000000

perception n realization 0.00044692043794734900000

perception n feeling 0.22965466287463000000000

perception n understanding 0.00935680346129437000000

perception n sight 0.00324640595007257000000

perception n belief 0.12302554863900400000000

perception n concept 0.00907951162278189000000

perception n knowledge 0.00018616137125199500000

perception n ken 0.00001634216881706000000

perception n impression 0.04970149313903140000000

perception n thought 0.00696111605247124000000

perception n view 0.38166970826693500000000

perception n awareness 0.00084381203425886700000

perception n notion 0.00002869564627619840000

programme n strategy 0.00310794670431850000000

programme n scheme 0.00257866842949656000000

programme n course 0.00002097351349182220000

programme n package 0.00101090347394330000000

programme n plan 0.00845840999379213000000

programme n agenda 0.00219326334915279000000

programme n show 0.00018186888761362200000

programme n schedule 0.01445664151948230000000

programme n initiative 0.00065498147018023100000

programme n production 0.00081133117030965800000

programme n order 0.00001324778641179210000

programme n transmission 0.00000128762118000501000

programme n program 0.96381828228167400000000

programme n timetable 0.00004874058219336000000

programme n prog 0.00218000617410463000000

programme n proposal 0.00045869265211803000000

programme n performance 0.00000475439053784553000

performer n promoter 0.00001640263824188410000

performer n mover 0.00188776341177445000000

performer n agent 0.00003280527648376890000

performer n dancer 0.00001640263824188410000

performer n entertainer 0.00060377330290365100000

performer n participant 0.00880454065569813000000

performer n worker 0.10846219143570100000000

performer n player 0.51326873114420900000000

performer n singer 0.00026012738508687000000

performer n actor 0.09922648154854450000000

performer n maker 0.01777040192426760000000

performer n actress 0.23245272161790600000000

performer n operator 0.01719765702094130000000

truce n rest 0.00037136099877657900000

truce n moratorium 0.00001238002143734180000

truce n pact 0.00760890749028527000000

truce n accord 0.00222563862279622000000

truce n armistice 0.19414394893378300000000

truce n respite 0.00475589507551622000000

truce n cessation 0.00428030556796459000000

truce n break 0.00239992382656677000000

truce n agreement 0.00994518844239181000000

truce n freedom 0.00036354599520276700000

truce n relief 0.00003772014671410200000

truce n cease-fire 0.01343233279248150000000

truce n ceasefire 0.71225781027343800000000

truce n suspension 0.00648212110023574000000

truce n amnesty 0.00833435344977699000000

truce n peace 0.01726508367882070000000

truce n halt 0.00837314331416942000000

truce n reconciliation 0.00000461904936261134000

truce n interval 0.00000337377281308788000

truce n lull 0.00014018468017351300000

truce n pause 0.00003036395531779090000

truce n treaty 0.00753179881197643000000

concept n twist 0.00019357754286740300000

concept n opinion 0.01082969913734270000000

concept n idea 0.41320329243070100000000

concept n approach 0.18332638691518000000000

concept n apprehension 0.00001347036920684890000

concept n hypothesis 0.00126621681378502000000

concept n consideration 0.00163138123114432000000

concept n image 0.00025830573111053300000

concept n presumption 0.00002694073841369770000

concept n conception 0.17305832332345800000000

concept n conclusion 0.04052135948061050000000

concept n inference 0.00007559332701956530000

concept n judgment 0.00066306739928707700000

concept n belief 0.00265818305144579000000

concept n perception 0.01746260703462030000000

concept n conjecture 0.00003624005095423520000

concept n conviction 0.00078927976774328100000

concept n theory 0.00140740973929676000000

concept n impression 0.00085939385311613600000

concept n thought 0.00075018138762728000000

concept n supposition 0.00007248010190847040000

concept n view 0.12224260291926100000000

concept n notion 0.02865400765389970000000

reviewer n judge 0.00018252884790734100000

reviewer n publicist 0.00000760536866280579000

reviewer n critic 0.99141353877969200000000

reviewer n investigator 0.00022055569122137100000

reviewer n commentator 0.00009886979261647640000

reviewer n analyst 0.00807690151989986000000

refrigerate v freeze 1.00000000000000000000000

litigation n dispute 0.05867639174756740000000

litigation n lawsuit 0.59532844766315000000000

litigation n allegation 0.02712118116425700000000

litigation n cause 0.00034307658767112300000

litigation n arraignment 0.00000583747077925305000

litigation n charge 0.10626736983558200000000

litigation n prosecution 0.00139703232565746000000

litigation n case 0.15302948622570300000000

litigation n controversy 0.00782058263896697000000

litigation n accusation 0.00007811452043637270000

litigation n proceedings 0.01675889145115470000000

litigation n action 0.02881752595950320000000

litigation n contest 0.00004482560918881040000

litigation n indictment 0.00429616446744530000000

litigation n contention 0.00001507233293766620000

politicize v politicise 1.00000000000000000000000

penitentiary n prison 1.00000000000000000000000

painless a easy 0.99860904229573200000000

painless a quick 0.00005888595797416130000

painless a simple 0.00133207174629431000000

overlap n addition 0.06189946394255070000000

overlap n coincidence 0.90715080408617400000000

overlap n extension 0.01547486598563760000000

overlap n parallel 0.01547486598563760000000

seminar n study 0.32383986931416800000000

seminar n workshop 0.14883141936597700000000

seminar n research 0.52721236988455300000000

seminar n lesson 0.00011634143530192500000

license n access 0.00018801935314705400000

license n document 0.00018873462391665900000

license n right 0.00024204418599393500000

license n indemnity 0.00001157170684178980000

license n title 0.00019260358521604000000

license n independence 0.00050365707409213800000

license n protection 0.00020582754829265500000

license n warrant 0.01164631362904870000000

license n complacency 0.00001924805641762130000

license n favor 0.00024811793221067400000

license n sanction 0.00392041067233057000000

license n freedom 0.00000477920643902118000

license n licence 0.64397522715591300000000

license n commission 0.11149985417335000000000

license n sauce 0.00000170724591810728000

license n certificate 0.00614599732237703000000

license n warranty 0.00000178302472396888000

license n instrument 0.00003192212027842320000

license n departure 0.00000684028488332209000

license n authority 0.00045917635115990300000

license n grant 0.00233095270555168000000

license n charter 0.00001032751775393720000

license n acceptance 0.00022656180768712100000

license n permission 0.00638363996276351000000

license n patent 0.00195600895331964000000

license n franchise 0.17204970696024300000000

license n permit 0.01532861602645560000000

license n excess 0.00426374899906378000000

license n exception 0.00004443654399060970000

license n pass 0.00000103053922215044000

license n passage 0.00041480466522470100000

license n liberty 0.00002423883907861410000

license n irregularity 0.00000103053922215044000

license n exemption 0.00040582519393060800000

license n allowance 0.00002572528442112400000

license n paper 0.00015505038507137400000

license n admission 0.00000103053922215044000

license n consent 0.00011527999719229900000

license n nerve 0.00000807935217207749000

license n entrance 0.00032757663573627500000

license n passport 0.00009986506888473300000

license n excuse 0.00000203012008787057000

license n immunity 0.00033388885575271500000

license n leave 0.00000067670669595684500

license n privilege 0.00016651163906784500000

license n approval 0.01547166642947640000000

license n certification 0.00009634420639866550000

license n copyright 0.00000466969091710381000

license n authorization 0.00007394662452274560000

license n release 0.00018289395832326600000

mollify v subdue 0.08215355998759940000000

mollify v relax 0.02004753539320030000000

mollify v soften 0.04009507078640070000000

mollify v reduce 0.18239123695360100000000

mollify v moderate 0.02004753539320030000000

mollify v diminish 0.08215355998759940000000

mollify v restrain 0.08215355998759940000000

mollify v alleviate 0.08215355998759940000000

mollify v ease 0.08019014157280150000000

mollify v lessen 0.32861423995039800000000

mix-up n scramble 0.03609973948641610000000

mix-up n uproar 0.07219947897283220000000

mix-up n skirmish 0.03609973948641610000000

mix-up n row 0.03609973948641610000000

mix-up n fight 0.07219947897283220000000

mix-up n mistake 0.74730182359508700000000

rescue v reclaim 0.00005285185473987310000

rescue v recover 0.10651507494102800000000

rescue v franchise 0.00004984478340225980000

rescue v deliver 0.00499142424053335000000

rescue v retrieve 0.05777914343413670000000

rescue v protect 0.01216594044239600000000

rescue v liberate 0.00139904196807112000000

rescue v withdraw 0.00719895990351606000000

rescue v save 0.47756412780370800000000

rescue v retain 0.00068615603221635000000

rescue v dismiss 0.00479987684097831000000

rescue v emancipate 0.00001005750545454030000

rescue v free 0.11108108697284000000000

rescue v discharge 0.01565681143532420000000

rescue v preserve 0.00035755666459623400000

rescue v release 0.15277233684380100000000

rescue v redeem 0.00031266643263128700000

rescue v relieve 0.00027967528474157800000

rescue v safeguard 0.00018295875170050100000

rescue v unleash 0.00000765833013403548000

rescue v acquit 0.00300346198847518000000

rescue v recapture 0.00852686732740895000000

rescue v salvage 0.03460642021816510000000

air n fire 0.03162217067209060000000

air n cast 0.00005525422036439070000

air n ozone 0.00000085634340799635900

air n draft 0.00501724248847079000000

air n feel 0.00001350089485860210000

air n space 0.00147179357843133000000

air n airwave 0.00041085295806219200000

air n breeze 0.00000085634340799635900

air n stratosphere 0.00000085634340799635900

air n presence 0.00687649502272747000000

air n wind 0.02890482858006920000000

air n aviation 0.46740060249246600000000

air n aura 0.00025008374463186700000

air n tone 0.00015661314997308700000

air n way 0.04718147427159650000000

air n sort 0.00048238464837360400000

air n mood 0.00184679238908382000000

air n climate 0.00089007080225176100000

air n line 0.20233930288668800000000

air n semblance 0.01237694536162350000000

air n sky 0.04786859877420180000000

air n exposure 0.00031387934011846800000

air n attitude 0.01021020929296580000000

air n publication 0.00036163213811830800000

air n aspect 0.00000171268681599272000

air n appearance 0.00001802905369500910000

air n look 0.00009310941529441620000

air n outlet 0.00011788986535341700000

air n style 0.00007087721511746190000

air n fashion 0.00059456868513666000000

air n circulation 0.00277587463868261000000

air n behavior 0.00026905043667500500000

air n atmosphere 0.01462387072267860000000

air n theme 0.00007258759025547420000

air n manner 0.00002785813312520060000

air n character 0.00001405261240086160000

air n strain 0.00000256903022398908000

air n song 0.00115401804560674000000

air n impression 0.00108985471313992000000

air n port 0.07692393125001960000000

air n publicity 0.00003873246357059950000

air n pretense 0.00291222243802907000000

air n oxygen 0.03305555159991490000000

air n tune 0.00009034266687316440000

god n christ 0.00008847406319602350000

god n icon 0.00012309596970469800000

god n power 0.39872794168652200000000

god n son 0.06945246059652760000000

god n idol 0.00000718090733693364000

god n shiva 0.00004258965864003120000

god n father 0.02933360579830520000000

god n brahma 0.00000718090733693364000

god n joss 0.00010458881729736800000

god n allah 0.10378744896753700000000

god n spirit 0.01546140059718460000000

god n lord 0.34877673016162200000000

god n ruler 0.00001436181467386730000

god n author 0.00472783998021859000000

god n master 0.00637283075211728000000

god n maker 0.00016388981813459400000

god n jesus 0.02280837950364450000000

lees n deposit 1.00000000000000000000000

train v ground 0.00002997029772611960000

train v lead 0.00515969779393685000000

train v order 0.00613495438942404000000

train v mark 0.00008853209162321870000

train v cultivate 0.00839375914334871000000

train v encourage 0.00016968286744244300000

train v qualify 0.00216189846323651000000

train v condition 0.00671138537456712000000

train v nurture 0.13087654222919900000000

train v nurse 0.00000452742513981482000

train v peg 0.00004074682625833370000

train v discipline 0.00134000887537739000000

train v accustom 0.00000905485027962965000

train v practise 0.00000452742513981482000

train v sight 0.00000905485027962965000

train v tame 0.00104407058096321000000

train v study 0.00302324619174585000000

train v groom 0.08896607855686870000000

train v hone 0.00000905485027962965000

train v learn 0.00719627317674364000000

train v check 0.00685046033466550000000

train v inform 0.04490744689468590000000

train v initiate 0.00056853293084608500000

train v school 0.13087201480405900000000

train v take 0.02811322504892230000000

train v instil 0.00000452742513981482000

train v update 0.02823785369309120000000

train v exercise 0.00246789091944407000000

train v direct 0.00000452742513981482000

train v reduce 0.02476059802453460000000

train v drill 0.00380343200367149000000

train v trail 0.00028235601899473300000

train v improve 0.00288189859002376000000

train v position 0.11532801950808800000000

train v develop 0.01660964801083380000000

train v equip 0.00135843646886464000000

train v level 0.00000905485027962965000

train v aim 0.00009305951676303360000

train v shape 0.00001859373656376440000

train v teach 0.00003498175887043950000

train v instruct 0.13087201480405900000000

train v prepare 0.10610541106061400000000

train v form 0.04729541798498160000000

train v educate 0.01570450651558800000000

train v coach 0.00233491258265330000000

train v focus 0.02910358540390400000000

train v practice 0.00000452742513981482000

knack n command 0.38479827089337200000000

knack n capacity 0.03544668587896250000000

knack n tendency 0.01772334293948120000000

knack n facility 0.54430835734870300000000

knack n trick 0.01772334293948120000000

hover v threaten 0.00029800472927501500000

hover v fly 0.15495757444357700000000

hover v protect 0.00028072540919659800000

hover v waver 0.05792740335037130000000

hover v float 0.00065134038290356200000

hover v oscillate 0.00000641266465055215000

hover v seesaw 0.03089461512019800000000

hover v drift 0.27008441259369400000000

hover v hang 0.00000641266465055215000

hover v linger 0.21039410762229000000000

hover v fluctuate 0.26814907315908700000000

hover v wait 0.00634991786010731000000

joyous a gay 0.00008336980762416730000

joyous a ecstatic 0.00004168490381208360000

joyous a satisfied 0.00120886221055045000000

joyous a joyful 0.99866608307801300000000

clearance n headway 0.00000053238144166157800

clearance n leeway 0.00014507345735946600000

clearance n opening 0.00040317739433354800000

clearance n room 0.00000300083685887412000

clearance n space 0.00000324717930301473000

clearance n gap 0.00001518157032762830000

clearance n break 0.00098726897877199000000

clearance n sanction 0.00003157459277385860000

clearance n licence 0.01153175640883690000000

clearance n split 0.00005990614396437550000

clearance n berth 0.00004474877080104850000

clearance n hole 0.00000161137381403958000

clearance n withdrawal 0.00009676920170190970000

clearance n permission 0.01741355534822880000000

clearance n passage 0.00001684366520275460000

clearance n removal 0.00000053238144166157800

clearance n allowance 0.00001581632491612600000

clearance n clearing 0.03899975825149480000000

clearance n consent 0.00039410051938828100000

clearance n crack 0.00000079857216249236800

clearance n leave 0.00000026619072083078900

clearance n approval 0.92525754530735700000000

clearance n separation 0.00001824351082919550000

clearance n margin 0.00005432834516063900000

clearance n authorization 0.00450381668187824000000

clearance n interval 0.00000054661093071642700

infrequent a occasional 0.94027833513791800000000

infrequent a irregular 0.00031801076693596500000

infrequent a casual 0.00031801076693596500000

infrequent a odd 0.00015900538346798200000

infrequent a limited 0.00938131762461100000000

infrequent a sparse 0.00015900538346798200000

infrequent a few 0.00015900538346798200000

infrequent a unusual 0.00079502691733991400000

infrequent a rare 0.04731924495157910000000

infrequent a isolated 0.00031801076693596500000

infrequent a sporadic 0.00015900538346798200000

infrequent a scarce 0.00063602153387193200000

merchandise n supplies 0.00754555432778186000000

merchandise n product 0.21425363910042800000000

merchandise n freight 0.00018918910687751800000

merchandise n stock 0.02174401395512060000000

merchandise n products 0.01937997910888640000000

merchandise n cargo 0.03450846497172750000000

merchandise n hardware 0.00055540404114764100000

merchandise n goods 0.69874694125550200000000

merchandise n shipment 0.00307681413252890000000

gambit n move 1.00000000000000000000000

build v establish 0.09843518077232910000000

build v devise 0.00004094616943253670000

build v enlarge 0.00311438041854462000000

build v model 0.00004400540036615130000

build v invent 0.00005248159206251340000

build v strengthen 0.00464405041430169000000

build v frame 0.00009732056788831970000

build v block 0.00108909962408411000000

build v reconstruct 0.00091298588138290300000

build v quicken 0.00002466084791609710000

build v increase 0.05025276380925930000000

build v weave 0.00000246110963089424000

build v start 0.02570232582229910000000

build v manufacture 0.02002768908393300000000

build v raise 0.01514252095503490000000

build v begin 0.02500610887372940000000

build v magnify 0.00004338522606803050000

build v fashion 0.00830501444945249000000

build v amplify 0.00000798086268155709000

build v make 0.10877521618618000000000

build v mount 0.01121703856028430000000

build v accelerate 0.00119767095411061000000

build v compile 0.00020203670410946400000

build v cast 0.00003156633716424030000

build v base 0.03348001710133770000000

build v found 0.00388841467789954000000

build v forge 0.01069389128091630000000

build v intensify 0.00018007380619890400000

build v hammer 0.00050764601950612600000

build v produce 0.06894588880621580000000

build v progress 0.00050754414083756700000

build v constitute 0.00314577048654562000000

build v boost 0.01160060800373460000000

build v assemble 0.01610550942885030000000

build v create 0.03566419912784140000000

build v develop 0.09076898628176090000000

build v construct 0.12617567310320000000000

build v formulate 0.00013404813147105200000

build v originate 0.00000429569712902706000

build v engineer 0.00000323219388567766000

build v shape 0.00094187579454604000000

build v swell 0.00260604887255983000000

build v compound 0.00001574610492291770000

build v evolve 0.00018747755745784300000

build v compose 0.00005924952296676520000

build v expand 0.11906406963542300000000

build v heighten 0.00216537112296656000000

build v carve 0.00000323219388567766000

build v form 0.02008163019586070000000

build v inaugurate 0.00038920036167159700000

build v institute 0.00005011340966470310000

build v extend 0.02184869183388470000000

build v fabricate 0.00009836575826214700000

build v erect 0.05631423872635180000000

bullet n cartridge 0.03123763851776030000000

bullet n missile 0.13585959706778700000000

bullet n shot 0.24615962355527000000000

bullet n shell 0.00632962202573605000000

bullet n ball 0.15057449048790500000000

bullet n smoke 0.00142714292548931000000

bullet n slug 0.24097606856558000000000

bullet n pellet 0.18742583110656200000000

bullet n hummer 0.00000998574791006027000

dishearten v intimidate 0.04733041177683340000000

dishearten v frighten 0.00591630147210407000000

dishearten v weaken 0.11791594827307000000000

dishearten v daunt 0.00591630147210407000000

dishearten v crush 0.01599994954299480000000

dishearten v dampen 0.01183260294420810000000

dishearten v discourage 0.07691191913735440000000

dishearten v tire 0.71817656538133100000000

diffuse v spread 0.18718007377963300000000

diffuse v distribute 0.14974405902370700000000

diffuse v strew 0.11230804426778000000000

diffuse v broadcast 0.03743601475592670000000

diffuse v scatter 0.03743601475592670000000

diffuse v expand 0.19565615259229600000000

diffuse v publish 0.28023964082473100000000

compulsion n task 1.00000000000000000000000

grand a highest 0.00069518475043930600000

grand a huge 0.00861928554811838000000

grand a great 0.40542705442190700000000

grand a fantastic 0.06760671698022250000000

grand a sensational 0.00211270990563195000000

grand a high 0.00128774699009948000000

grand a complete 0.00111787707521201000000

grand a good 0.01373770280549790000000

grand a widespread 0.00066937343544774800000

grand a illustrious 0.00112560610997249000000

grand a main 0.00425739309449320000000

grand a grandiose 0.00056280305498624400000

grand a chief 0.00015339016898519000000

grand a comprehensive 0.00343783285807810000000

grand a ambitious 0.00158028849040695000000

grand a impressive 0.00605233129053567000000

grand a large 0.00122712135188152000000

grand a big 0.46705776368922100000000

grand a important 0.00838550152088716000000

grand a notable 0.00112560610997249000000

grand a unrealistic 0.00376071034800417000000

categorical a unconditional 0.00109288644794598000000

categorical a flat 0.05299481055350500000000

categorical a pronounced 0.06516832987835250000000

categorical a sure 0.25480413622919500000000

categorical a confident 0.17199785478626500000000

categorical a conclusive 0.00814604123479406000000

categorical a clear-cut 0.00814604123479406000000

categorical a specific 0.00814604123479406000000

categorical a actual 0.00218577289589197000000

categorical a true 0.00874309158356789000000

categorical a absolute 0.00109288644794598000000

categorical a official 0.00218577289589197000000

categorical a accurate 0.00814604123479406000000

categorical a positive 0.11766730433268500000000

categorical a marked 0.00814604123479406000000

categorical a definite 0.00218577289589197000000

categorical a certain 0.27915117487889000000000

cataract n falls 1.00000000000000000000000

cabaret n nightclub 0.55692307692307700000000

cabaret n production 0.44307692307692300000000

differ v oppose 0.03967089715756650000000

differ v argue 0.01209616757942060000000

differ v qualify 0.00375125441274885000000

differ v reverse 0.00006093839809150000000

differ v change 0.24957489955678100000000

differ v vary 0.38442988436022800000000

differ v counter 0.00006093839809150000000

differ v disagree 0.30447785018780200000000

differ v conflict 0.00587716994926912000000

bisect v split 1.00000000000000000000000

prediction n guess 0.00138125398230235000000

prediction n speculation 0.01066743403084730000000

prediction n augury 0.00000448429248339853000

prediction n prognosis 0.00030102905361525100000

prediction n forecast 0.89265888927731200000000

prediction n surmise 0.00000100295358698823000

prediction n projection 0.08109151396767320000000

prediction n anticipation 0.00000610435920643018000

prediction n announcement 0.01388378235872610000000

prediction n forecasting 0.00000450572424753707000

above a earlier 0.03734189704803060000000

above a former 0.00575131138759096000000

above a prior 0.46951445632778800000000

above a previous 0.48739233523659000000000

visa n permission 0.86818881931533500000000

visa n endorsement 0.13181118068466500000000

fluctuation n uncertainty 0.04535652256924480000000

fluctuation n fickleness 0.00017777635052942000000

fluctuation n change 0.63959996233160000000000

fluctuation n instability 0.08009346929566680000000

fluctuation n variation 0.23477226945295900000000

untried a new 1.00000000000000000000000

unjustifiable a incorrect 0.06175132826435560000000

unjustifiable a ridiculous 0.03329972498020380000000

unjustifiable a unreasonable 0.08482214871349590000000

unjustifiable a invalid 0.00139869992164294000000

unjustifiable a outrageous 0.01521811186757820000000

unjustifiable a groundless 0.13572506434771900000000

unjustifiable a unjust 0.16993725769167300000000

unjustifiable a baseless 0.11998713335254100000000

unjustifiable a erroneous 0.02985030320579470000000

unjustifiable a unwarranted 0.01632385152454030000000

unjustifiable a wrong 0.11612837040575500000000

unjustifiable a illogical 0.00139869992164294000000

unjustifiable a excessive 0.03183492365679940000000

unjustifiable a preposterous 0.00087888079404264800000

unjustifiable a inexcusable 0.00029296026468088000000

unjustifiable a unfounded 0.18115254108753500000000

torso n body 1.00000000000000000000000

unexpected a impetuous 0.00005509243333260610000

unexpected a unpredictable 0.01297011615823240000000

unexpected a amazing 0.00513478785595363000000

unexpected a abrupt 0.00010297105206166700000

unexpected a staggering 0.00048269621181455700000

unexpected a unforeseen 0.07255673469904230000000

unexpected a astonishing 0.09695947958471870000000

unexpected a accidental 0.00049709723094113600000

unexpected a startling 0.00786489617954778000000

unexpected a unusual 0.03193730301691230000000

unexpected a unintentional 0.00001004019645395210000

unexpected a sudden 0.08213144259977350000000

unexpected a surprising 0.68769113257116400000000

unexpected a instantaneous 0.00010297105206166700000

unexpected a swift 0.00001004019645395210000

unexpected a unseasonable 0.00045490115798772600000

unexpected a stunning 0.00030358466401272900000

unexpected a wonderful 0.00073471313953548800000

texture n strategy 0.30171756090829000000000

texture n organization 0.05589469198750880000000

texture n structure 0.10891614171451800000000

texture n constitution 0.00143662113024983000000

texture n sense 0.00430986339074950000000

texture n web 0.00143662113024983000000

texture n feeling 0.00143662113024983000000

texture n appearance 0.05445807085725890000000

texture n finish 0.00143662113024983000000

texture n surface 0.00287324226049966000000

texture n balance 0.02511734323124600000000

texture n network 0.43809335886843000000000

texture n grain 0.00287324226049966000000

task v charge 0.96808011265842600000000

task v load 0.00531998122359567000000

task v test 0.01063996244719130000000

task v push 0.01595994367078700000000

submerged a underwater 1.00000000000000000000000

suppose v say 0.19024774357212300000000

suppose v dream 0.00003657462097441770000

suppose v presume 0.00037969651567779700000

suppose v get 0.01190980741545250000000

suppose v regard 0.00003265425991771330000

suppose v pretend 0.00009378099737678090000

suppose v think 0.20971635642689500000000

suppose v assume 0.00676323267701162000000

suppose v conclude 0.00025635160327825100000

suppose v calculate 0.00021768570404517200000

suppose v expect 0.44340632406933900000000

suppose v understand 0.01167637090092480000000

suppose v apprehend 0.00000202216378245999000

suppose v realize 0.00405600264865909000000

suppose v daresay 0.00000269328891691676000

suppose v gather 0.00001653116666197250000

suppose v allow 0.03585479660828110000000

suppose v suspect 0.00387383435420135000000

suppose v believe 0.05960234524528320000000

suppose v speculate 0.00003337860961112980000

suppose v imply 0.00010505580299006200000

suppose v consider 0.00765691718062568000000

suppose v judge 0.00058234314761542100000

suppose v surmise 0.00000345052395191571000

suppose v deem 0.00004400111578650680000

suppose v imagine 0.00408576394635706000000

suppose v deduce 0.00000070537804279601200

suppose v guess 0.00605757704839769000000

suppose v figure 0.00005554986203231220000

suppose v reckon 0.00006449575191840190000

suppose v opine 0.00000225183686272912000

suppose v dig 0.00316370555700611000000

stupidity n incompetence 1.00000000000000000000000

pioneer n antecedent 0.00000942930974716294000

pioneer n pacemaker 0.00006641852530508530000

pioneer n pathfinder 0.00036607061979724500000

pioneer n leader 0.27339137354283700000000

pioneer n forerunner 0.22593666626093200000000

pioneer n scout 0.00033915981142579600000

pioneer n miner 0.00313313194791855000000

pioneer n developer 0.03786147663244920000000

pioneer n immigrant 0.00052074647050955300000

pioneer n innovator 0.00000942930974716294000

pioneer n settler 0.00147379758622929000000

pioneer n engineer 0.00121897595467525000000

pioneer n point 0.28301658475775100000000

pioneer n predecessor 0.00059470368078958600000

pioneer n explorer 0.17061948256406300000000

pioneer n pilgrim 0.00121316782307964000000

pioneer n guide 0.00020707052024499900000

pioneer n architect 0.00002231468249791670000

activity n employment 0.00965976410113576000000

activity n event 0.01332251827107170000000

activity n doing 0.00000109185536886229000

activity n progress 0.01413164982626560000000

activity n swiftness 0.00000034727905293669100

activity n do 0.00000018967360646378500

activity n service 0.05285330706027440000000

activity n scheme 0.00694243804178849000000

activity n industry 0.05079294363359170000000

activity n act 0.00640516905573277000000

activity n vigor 0.00004765166033546660000

activity n affair 0.01270712232015810000000

activity n application 0.00261290545130785000000

activity n diversion 0.00021295511124481300000

activity n animation 0.00049243933115300800000

activity n enterprise 0.00486984903181132000000

activity n operation 0.17096710552572000000000

activity n occurrence 0.00006085639015163700000

activity n task 0.00513231045949233000000

activity n vitality 0.00029911949426191900000

activity n fuss 0.00003627278227650600000

activity n motion 0.00051952001998804700000

activity n exercise 0.00562214912565075000000

activity n hobby 0.00000029551530548876500

activity n spirit 0.00190746633026286000000

activity n project 0.03861508931862360000000

activity n pastime 0.00003212331239664430000

activity n function 0.00662702299336504000000

activity n execution 0.00004967574614002600000

activity n working 0.00006085639015163700000

activity n occupation 0.00009012806555646820000

activity n energy 0.00151125750931957000000

activity n action 0.10269612239550100000000

activity n life 0.00520790933757175000000

activity n effort 0.01681559956979870000000

activity n process 0.00884395911929668000000

activity n work 0.03221589131165930000000

activity n venture 0.01232484004923820000000

activity n flurry 0.00036447056908760100000

activity n labor 0.00112404884091203000000

activity n performance 0.02647428826635420000000

activity n bustle 0.00003014470257511100000

activity n business 0.34084814597469900000000

activity n happening 0.00001629443382438490000

activity n pursuit 0.00225876151301277000000

activity n movement 0.04042284973059460000000

activity n undertaking 0.00021728233495924200000

activity n engagement 0.00256105875955057000000

activity n readiness 0.00006348286016893060000

activity n recreation 0.00093125954863415600000

lunch n luncheon 0.14278180904199600000000

lunch n bite 0.00001036489059030150000

lunch n restaurant 0.00001416019929824290000

lunch n meal 0.00014671394841583600000

lunch n diner 0.00003965203721192850000

lunch n tea 0.85697418442771600000000

lunch n bar 0.00003311545477195490000

pusher n dealer 1.00000000000000000000000

oversupply v flood 1.00000000000000000000000

overplay v stretch 0.00417178972955245000000

overplay v overdo 0.67666275817903200000000

overplay v exaggerate 0.18583123849474800000000

overplay v overstate 0.13333421359666700000000

gore n bloodshed 0.00061738229243258300000

gore n panel 0.41648889854965700000000

gore n murder 0.57787127107765900000000

gore n slaughter 0.00086199805942777900000

gore n blood 0.00200807187809946000000

gore n massacre 0.00215237814272358000000

mortuary n morgue 1.00000000000000000000000

steam n vapor 0.01516189494202270000000

steam n smoke 0.66138273244377800000000

steam n cloud 0.03474600924213540000000

steam n moisture 0.05319027152080250000000

steam n party 0.23551909185126100000000

pave v cover 1.00000000000000000000000

licenced a licensed 1.00000000000000000000000

final a supreme 0.00000028666818411880100

final a finishing 0.02877776234474230000000

final a last 0.58295495546560900000000

final a unavoidable 0.00043667295241286600000

final a sure 0.00894975546931416000000

final a conclusive 0.00603230991706664000000

final a irrevocable 0.00188990136867177000000

final a fixed 0.00136329537584381000000

final a terminal 0.00123098414118790000000

final a complete 0.01788603949019140000000

final a unalterable 0.00000753824036929829000

final a exhaustive 0.00001088180907864710000

final a crowning 0.00276219128807674000000

final a definitive 0.09214801440428870000000

final a latest 0.13635096645562000000000

final a absolute 0.00560507705568490000000

final a indisputable 0.00000400414224483044000

final a net 0.04518802904963560000000

final a decisive 0.01167486176954700000000

final a extreme 0.00035559128532175800000

final a inevitable 0.00099761851573052700000

final a coming 0.00434519101518710000000

final a eventual 0.00146527292074316000000

final a binding 0.00226980268567314000000

final a developed 0.00015204722686661000000

final a latter 0.00151395699854371000000

final a last-minute 0.00223344249262329000000

final a ultimate 0.02198263629138850000000

final a finished 0.00232602631359783000000

final a thorough 0.00004101854118650720000

final a definite 0.01247649367655380000000

final a certain 0.00656105382495167000000

final a end 0.00000632080386287583000

objection n grievance 0.00596724920197691000000

objection n dispute 0.05912597128027620000000

objection n dissatisfaction 0.00000753089576060544000

objection n ban 0.02310207855739540000000

objection n difference 0.00878841614346224000000

objection n contradiction 0.00004288200654072190000

objection n resentment 0.00000743158013841752000

objection n challenge 0.00381439254894030000000

objection n hesitation 0.00009746868025007650000

objection n beef 0.00032172481270423900000

objection n complaint 0.00429227323364755000000

objection n criticism 0.05570029102035420000000

objection n reprimand 0.00012642981532105900000

objection n abuse 0.00002038923417531970000

objection n problem 0.13821608479122900000000

objection n charge 0.02488305417285710000000

objection n answer 0.00547002833651767000000

objection n disdain 0.00013665456187733400000

objection n opposition 0.28987806889558700000000

objection n accusation 0.00087573500788325700000

objection n exception 0.00042656897146673900000

objection n veto 0.03269215894706510000000

objection n plea 0.00007068355658631720000

objection n disagreement 0.00248076580924386000000

objection n reply 0.00001145290386290830000

objection n indictment 0.00025594707705832500000

objection n crack 0.00010490649793059400000

objection n rejection 0.02688530190275540000000

objection n reluctance 0.00017085443102160800000

objection n question 0.05977607035992380000000

objection n defense 0.00077231460201874300000

objection n denial 0.00182700195625230000000

objection n issue 0.13836604508236500000000

objection n admonition 0.02386899680790760000000

objection n insistence 0.00006006920382894500000

objection n frown 0.00002260511946198840000

objection n blame 0.00001058959747452190000

objection n regret 0.00057182203935754300000

objection n dissent 0.00006027524446441320000

objection n condemnation 0.00037166258005392500000

objection n discontent 0.00001887605621396300000

objection n protest 0.09030087647679220000000

ninth a 9th 1.00000000000000000000000

principal n mover 0.00009186540345987160000

principal n star 0.00377484588744005000000

principal n chancellor 0.00066503838758245400000

principal n foreman 0.00009964308376895600000

principal n captain 0.00008320911316662970000

principal n chairman 0.22971644206980500000000

principal n director 0.00541043420814374000000

principal n head 0.00041035649107629800000

principal n boss 0.01767371457759820000000

principal n manager 0.23027766732138300000000

principal n chief 0.03264174639365230000000

principal n president 0.02375761802440910000000

principal n superior 0.00003246319880025030000

principal n lead 0.00228600702609933000000

principal n supervisor 0.00010190309945615600000

principal n dealer 0.45297704571415800000000

enforcement n intimidation 0.00057452563271527000000

enforcement n domination 0.00001982562117978570000

enforcement n threat 0.00463202850776234000000

enforcement n requirement 0.00077787808744600900000

enforcement n obligation 0.28681425325706000000000

enforcement n demand 0.14956735491704400000000

enforcement n implementation 0.01406023768014400000000

enforcement n force 0.26036340934108300000000

enforcement n completion 0.00478876802503330000000

enforcement n spur 0.00001982562117978570000

enforcement n prosecution 0.00280746362693416000000

enforcement n execution 0.00038854766739788800000

enforcement n crackdown 0.00015462259133937000000

enforcement n push 0.00231707271684697000000

enforcement n pressure 0.06127109594369760000000

enforcement n supervision 0.00026959394031917300000

enforcement n control 0.03902764553323170000000

enforcement n press 0.16705315524897300000000

enforcement n constraint 0.00013479697015958400000

enforcement n insistence 0.00231707271684697000000

enforcement n prescription 0.00001982562117978570000

enforcement n provocation 0.00262100073242572000000

sponsor v back 0.47551259278807600000000

sponsor v finance 0.04854013004005860000000

sponsor v promise 0.00195226651519597000000

sponsor v recommend 0.04032844539871240000000

sponsor v fund 0.00033387129847209300000

sponsor v advance 0.00053948817379124000000

sponsor v guarantee 0.01490980978336200000000

sponsor v bankroll 0.00013864464695249700000

sponsor v assure 0.00515956150444648000000

sponsor v support 0.41258518985093300000000

finalise v finalize 1.00000000000000000000000

extraneous a irrelevant 0.16666666666666700000000

extraneous a additional 0.83333333333333300000000

exclaim v declare 0.12499999999999800000000

exclaim v call 0.12499999999999800000000

exclaim v cry 0.75000000000000300000000

erection n structure 0.01401228560365850000000

erection n construction 0.98085999225609300000000

erection n building 0.00512772214024843000000

endow v will 1.00000000000000000000000

survey v follow 0.00013799487558371500000

survey v determine 0.00000332469893146433000

survey v scan 0.00000060332853917568800

survey v chart 0.00000011147588700594400

survey v regard 0.00014034757298970700000

survey v watch 0.00002417704254370640000

survey v review 0.00002396919764319890000

survey v investigate 0.00007513280860641160000

survey v note 0.00100853103859366000000

survey v calculate 0.00006587071518755360000

survey v question 0.03223434302967660000000

survey v ascertain 0.00000060332853917568800

survey v canvass 0.00109624159151037000000

survey v assess 0.00001900131779425910000

survey v view 0.00002181282176628220000

survey v study 0.00004454744552743630000

survey v monitor 0.00001517221680912530000

survey v appraise 0.00000033442766101783100

survey v measure 0.00000120665707835138000

survey v scrutinize 0.00000095492005563412100

survey v examine 0.00002302688256166930000

survey v check 0.00001068845502872310000

survey v delineate 0.00000011147588700594400

survey v poll 0.94604318494280800000000

survey v eye 0.00000095492005563412100

survey v consider 0.00125227584372638000000

survey v judge 0.00000914101356129826000

survey v estimate 0.00025188874272025400000

survey v observe 0.00000384142458531162000

survey v interview 0.01746227759118480000000

survey v figure 0.00000645013056427826000

survey v trace 0.00000022295177401188800

survey v inspect 0.00000220918493261906000

survey v reckon 0.00000961137702704108000

survey v notice 0.00000438577325634454000

survey v compute 0.00000089105196726174500

survey v contemplate 0.00000455772743524765000

disguised a hidden 1.00000000000000000000000

decapitate v execute 1.00000000000000000000000

reporter n columnist 0.00061630383083115000000

reporter n pundit 0.00000169472667935116000

reporter n interviewer 0.02876207117527980000000

reporter n cameraman 0.01231187539447710000000

reporter n anchor 0.00000602742106024507000

reporter n staffer 0.00006840879027749370000

reporter n casey 0.00001028750156757890000

reporter n journalist 0.81857937453762500000000

reporter n investigator 0.01882035007407920000000

reporter n announcer 0.00000168208867234149000

reporter n stringer 0.00010685250779583000000

reporter n reviewer 0.00000273878041044859000

reporter n newsperson 0.00167690860381647000000

reporter n editor 0.00525233472154197000000

reporter n newsman 0.00284165082739172000000

reporter n commentator 0.00072475252019455800000

reporter n correspondent 0.10906455058455300000000

reporter n writer 0.00005095910535956030000

reporter n reader 0.00110117680838654000000

cupboard n closet 1.00000000000000000000000

counterfeit v clone 0.00495049504950486000000

counterfeit v act 0.02970297029702960000000

counterfeit v duplicate 0.00495049504950486000000

counterfeit v assume 0.02475247524752480000000

counterfeit v match 0.02475247524752480000000

counterfeit v favor 0.00495049504950486000000

counterfeit v reproduce 0.00495049504950486000000

counterfeit v fake 0.00990099009900972000000

counterfeit v bluff 0.01980198019801970000000

counterfeit v affect 0.87128712871287200000000

congresswoman n representative 1.00000000000000000000000

authorize v establish 0.00424658499654971000000

authorize v empower 0.00163497856714014000000

authorize v let 0.00000031815523983059900

authorize v qualify 0.00000113473061502207000

authorize v defend 0.00000031815523983059900

authorize v clear 0.00169170292588715000000

authorize v validate 0.00000042420698644079800

authorize v excuse 0.00000010605174661020000

authorize v explain 0.00015752763284946000000

authorize v allow 0.01599323001272770000000

authorize v acknowledge 0.00000137867270593261000

authorize v permit 0.00264778718806551000000

authorize v entitle 0.00204400952556732000000

authorize v justify 0.00000137867270593261000

authorize v ratify 0.00017599285042592100000

authorize v authorise 0.37892068106419500000000

authorize v approve 0.58186624379138300000000

authorize v uphold 0.00000138920865947241000

authorize v license 0.00000031815523983059900

authorize v commission 0.00000085894992642140400

authorize v support 0.00017621548987268100000

authorize v underwrite 0.00000010605174661020000

authorize v confirm 0.00053163722116728000000

authorize v tolerate 0.00000057263328428093800

authorize v certify 0.00003183426391067650000

authorize v affirm 0.00000074236222627140300

authorize v accept 0.00212258542932348000000

authorize v sanction 0.00306615744667205000000

authorize v invest 0.00161231524872082000000

authorize v pass 0.00307086586733706000000

authorize v institute 0.00000010605174661020000

authorize v suffer 0.00000049842013536086500

complexion n front 0.00415800415800416000000

complexion n kind 0.00415800415800416000000

complexion n turn 0.98752598752598800000000

complexion n aspect 0.00415800415800416000000

coincidental a unpredictable 0.01430900779043360000000

coincidental a simultaneous 0.00024935868736328600000

coincidental a fortunate 0.06272799604666450000000

coincidental a accidental 0.00099743474945315600000

coincidental a unexpected 0.92171620272608500000000

expose v relate 0.00077779844874041600000

expose v unfold 0.00003173675531997840000

expose v present 0.00092670095210010000000

expose v betray 0.00009521026595993530000

expose v report 0.03716624608517620000000

expose v prove 0.00121354552689493000000

expose v show 0.08164621458258080000000

expose v unearth 0.00009521026595993530000

expose v ditch 0.00006347351063995690000

expose v jeopardize 0.00031004321314688400000

expose v disclose 0.00218248756409974000000

expose v reveal 0.20520059057499000000000

expose v unveil 0.00041020292500520300000

expose v abandon 0.00116041357913938000000

expose v condemn 0.00015868377659989400000

expose v find 0.37709487388574000000000

expose v inform 0.00135782487395762000000

expose v discover 0.00057054466214536400000

expose v open 0.07998360921414950000000

expose v feature 0.00002858984248239260000

expose v tell 0.00426766614003466000000

expose v broadcast 0.00005870954485475920000

expose v release 0.14239709945877500000000

expose v print 0.00015850077236640600000

expose v display 0.00004045918430217100000

expose v strip 0.00003173675531997840000

expose v detect 0.00093327553982757400000

expose v leak 0.00047605132979968200000

expose v strand 0.00043977799249910200000

expose v break 0.00407035855354924000000

expose v divest 0.00020669468475956600000

expose v endanger 0.00108192152356888000000

expose v uncover 0.05126316989751500000000

expose v air 0.00006655849233161710000

expose v communicate 0.00003173675531997840000

expose v advertise 0.00006966433021405700000

expose v desert 0.00001510344771500240000

expose v subject 0.00009521026595993530000

expose v publish 0.00318891942140243000000

expose v indicate 0.00063338540505641400000

lord n monarch 0.00011385497336791200000

lord n magnate 0.00000222345200430871000

lord n superintendent 0.00250101292951325000000

lord n sovereign 0.00000420947521915361000

lord n king 0.00241599973511110000000

lord n christ 0.00947752268026075000000

lord n owner 0.00031058833699147700000

lord n providence 0.00001110738532722390000

lord n leader 0.01010209611601330000000

lord n earl 0.03752315519583460000000

lord n landlord 0.00000307890659174763000

lord n rex 0.00000420947521915361000

lord n overseer 0.00005904288902090580000

lord n kingpin 0.40066728303399700000000

lord n premier 0.00230355993157178000000

lord n foreman 0.00000861198032704959000

lord n captain 0.00610295021077398000000

lord n commander 0.00006213947718812660000

lord n prince 0.00067807643431943400000

lord n chairman 0.01592240480219310000000

lord n dean 0.00001217072835483590000

lord n director 0.00132619646220230000000

lord n executive 0.00266958758657919000000

lord n count 0.00004844076050501560000

lord n super 0.00012592512652094700000

lord n head 0.00270870583796934000000

lord n boss 0.07589080558541100000000

lord n manager 0.00914559986595767000000

lord n principal 0.00007762466881297930000

lord n khan 0.00111221179717452000000

lord n ruler 0.00001110738532722390000

lord n don 0.00002518502530418940000

lord n general 0.00033765780550158600000

lord n master 0.00002518334119596180000

lord n chief 0.06770418314136760000000

lord n rajah 0.00005545667124468710000

lord n administrator 0.00566316707739186000000

lord n president 0.04930804180081300000000

lord n maker 0.01667872188868440000000

lord n superior 0.00001638020357398950000

lord n duke 0.00001832854153833120000

lord n supervisor 0.00004593047407347230000

lord n kaiser 0.00001984143696735580000

lord n emperor 0.00024708016800767500000

lord n baron 0.27679821342379900000000

lord n governor 0.00162380873564606000000

lord n noble 0.00003131703923010310000

burned-out a burnt 0.00435729847494553000000

burned-out a burnt-out 0.99564270152505500000000

entirely r only 0.03964219012741710000000

entirely r quite 0.11275741312368200000000

entirely r explicitly 0.01716630183677800000000

entirely r clearly 0.00173518347581549000000

entirely r extensively 0.00065579130612410200000

entirely r just 0.01337587760925920000000

entirely r well 0.00077603642954300500000

entirely r alone 0.00003520423827079950000

entirely r completely 0.29821872076161000000000

entirely r substantially 0.00095654448568268300000

entirely r absolutely 0.02199617208368290000000

entirely r totally 0.19330476643147400000000

entirely r all 0.01123275737998620000000

entirely r wholly 0.05512290256476480000000

entirely r essentially 0.00005675146658742820000

entirely r fundamentally 0.00916411273009464000000

entirely r purely 0.02431892760210210000000

entirely r solely 0.01459135656126130000000

entirely r altogether 0.04271869052284500000000

entirely r outright 0.00729567828063063000000

entirely r perfectly 0.01091286331052310000000

entirely r fully 0.12396575767186600000000

blemish n mark 0.00087441705529646700000

blemish n blister 0.00006245836109260370000

blemish n blot 0.99756412391738800000000

blemish n fault 0.00124916722185210000000

blemish n spot 0.00018737508327781400000

blemish n defect 0.00006245836109260370000

bison n buffalo 1.00000000000000000000000

psychological a personal 0.60126439951148100000000

psychological a unconscious 0.00016410664570920800000

psychological a emotional 0.39451552141949000000000

psychological a mental 0.00227953847075647000000

psychological a intellectual 0.00058922100411782400000

psychological a irrational 0.00118721294844509000000

belligerent a aggressive 1.00000000000000000000000

valid a convincing 0.00047624868979301400000

valid a supportable 0.00001915059231060270000

valid a compelling 0.03001459274002100000000

valid a kosher 0.00001915059231060270000

valid a material 0.00034736522018272200000

valid a effective 0.21604120009947100000000

valid a licensed 0.00003830118462120530000

valid a accountable 0.00002194690084432760000

valid a objective 0.00012132706146711500000

valid a rational 0.00013229154009189300000

valid a approved 0.00012969234433966300000

valid a equitable 0.00014508858445698900000

valid a obligatory 0.00000075580350651281500

valid a authentic 0.00018361305008056400000

valid a right 0.02233036092917510000000

valid a proved 0.00000075580350651281500

valid a substantial 0.00186721049105313000000

valid a impartial 0.00032765712934345400000

valid a conclusive 0.00018680226749459100000

valid a strong 0.04389509417861880000000

valid a powerful 0.00003008008665567080000

valid a solid 0.02349076468692040000000

valid a actual 0.00177271449025148000000

valid a fair 0.00456488421613020000000

valid a original 0.00661087111581692000000

valid a true 0.01038766555686620000000

valid a good 0.12272125991361700000000

valid a lawful 0.00003208592199674130000

valid a operative 0.00105289802703677000000

valid a appropriate 0.00734328734424377000000

valid a justifiable 0.00037518838566955000000

valid a forcible 0.00000075580350651281500

valid a sufficient 0.00489973432872601000000

valid a trustworthy 0.00001915059231060270000

valid a reasonable 0.00211221323483927000000

valid a persuasive 0.00029712880999638700000

valid a related 0.00046531472138060100000

valid a adequate 0.00668127334455216000000

valid a sustainable 0.00646010560438507000000

valid a efficient 0.00373491556901710000000

valid a definitive 0.00081742103638086200000

valid a final 0.03285631268480740000000

valid a well-founded 0.00041904202999924300000

valid a sound 0.02273044604699890000000

valid a applicable 0.00332221945224454000000

valid a scientific 0.00001915059231060270000

valid a proper 0.00450823521207914000000

valid a available 0.20603040901680500000000

valid a relevant 0.01272725746861620000000

valid a official 0.03597157430896560000000

valid a decisive 0.03073838835088870000000

valid a correct 0.00439267420495754000000

valid a binding 0.07184936208010030000000

valid a legitimate 0.00408273703925894000000

valid a real 0.00687650794043645000000

valid a genuine 0.00195285653977011000000

valid a accurate 0.00481411053693816000000

valid a credible 0.00044475228264948600000

valid a important 0.00980046543096601000000

valid a pure 0.00000982689039065012000

valid a ultimate 0.00042439903676477600000

valid a heavy 0.01718955342222730000000

valid a grave 0.00003653857853887770000

valid a stringent 0.00009830206551636880000

valid a honest 0.00028419432583069400000

valid a logical 0.00054194939842795300000

valid a significant 0.00457519094706153000000

valid a legal 0.00305990070494760000000

valid a definite 0.00007532541851402150000

capable a accessible 0.00000410955495995234000

capable a effective 0.00178221250053831000000

capable a intelligent 0.00000621706136232417000

capable a competent 0.00078554849779552400000

capable a talented 0.00001664913552939210000

capable a subject 0.00001438344235983340000

capable a susceptible 0.00000205477747997617000

capable a clever 0.00009158714236923020000

capable a equal 0.00002632477619065780000

capable a receptive 0.00001243412272464830000

capable a gifted 0.00223760597620591000000

capable a skilful 0.00000416228388234800000

capable a adequate 0.04184628174649110000000

capable a efficient 0.02854377052050760000000

capable a impressive 0.00019678617004337900000

capable a useful 0.00566289575575929000000

capable a suitable 0.00001643821983980970000

capable a able 0.90334572074343400000000

capable a open 0.00080337907628129100000

capable a experienced 0.01406906140539710000000

capable a smart 0.00049033228187181700000

capable a practiced 0.00001868919190452920000

capable a brilliant 0.00001332806446056480000

capable a skilled 0.00001002755261158270000

shuttle n birdie 0.00656988136278092000000

shuttle n bird 0.99343011863721900000000

west n westward 0.22482948501993400000000

west n w 0.77517051498006600000000

shall v intend 0.01709782598977100000000

shall v must 0.98290217401022900000000

willful a intentional 0.14285714285714300000000

willful a wilful 0.85714285714285700000000

watchword n word 0.93053020318037800000000

watchword n signal 0.05770729942203890000000

watchword n motto 0.00582833558460992000000

watchword n keyword 0.00120063572040531000000

watchword n slogan 0.00296708090648659000000

watchword n sign 0.00176644518608128000000

unintentional a erratic 0.09767122144041110000000

unintentional a accidental 0.85684717011706700000000

unintentional a spontaneous 0.03032107229501490000000

unintentional a unexpected 0.01516053614750730000000

unbiased a neutral 0.75000000000000000000000

unbiased a independent 0.25000000000000000000000

subsidiary a extra 0.18325915990977300000000

subsidiary a junior 0.81674084009022700000000

squabble v argue 1.00000000000000000000000

propagate v deliver 0.22222222222222200000000

propagate v generate 0.11111111111111100000000

propagate v grow 0.11111111111111100000000

propagate v issue 0.22222222222222200000000

propagate v tell 0.11111111111111100000000

propagate v extend 0.11111111111111100000000

propagate v sow 0.11111111111111100000000

militant n soldier 0.13068456955522600000000

militant n warrior 0.00012183906219599800000

militant n rioter 0.00203652186284551000000

militant n activist 0.26066077904739100000000

militant n fighter 0.03937785248059520000000

militant n demonstrator 0.06573363502441440000000

militant n zealot 0.08031224849752890000000

militant n contender 0.00202467853355115000000

militant n extremist 0.24131573219763800000000

militant n attacker 0.01555140293487320000000

militant n radical 0.16151062133504100000000

militant n serviceman 0.00067011946870040000000

consider v moot 0.00248389365411969000000

consider v sense 0.00003999719440552340000

consider v deal 0.00688562357849932000000

consider v attend 0.00190747485390541000000

consider v envision 0.00000032233763789139300

consider v mark 0.00062410324047771700000

consider v grant 0.00724397310244746000000

consider v regard 0.17132203009278900000000

consider v ponder 0.00060500625714629900000

consider v deliberate 0.00799117525214920000000

consider v admit 0.00174833103650700000000

consider v investigate 0.01148289299346790000000

consider v recognize 0.00869396724967548000000

consider v value 0.00273823229014868000000

consider v note 0.00853036732527574000000

consider v conceive 0.00001008452184061600000

consider v think 0.09450374672823040000000

consider v weigh 0.02180268191828640000000

consider v view 0.10447329521988100000000

consider v study 0.07402892676449050000000

consider v reason 0.00000233134259876394000

consider v rate 0.00411074974559594000000

consider v appraise 0.00000423827744874423000

consider v acknowledge 0.00218365496491660000000

consider v scrutinize 0.00091485725742902500000

consider v examine 0.02561609090126720000000

consider v find 0.01250684297421170000000

consider v take 0.05639411131514260000000

consider v believe 0.03899252159880570000000

consider v speculate 0.00305167475611746000000

consider v respect 0.00212642184934306000000

consider v count 0.00330640990569169000000

consider v evaluate 0.00895604190639798000000

consider v concede 0.00090468429719817000000

consider v suppose 0.00137293231519593000000

consider v survey 0.00044084375642447300000

consider v debate 0.01100232514393270000000

consider v judge 0.01007332056563030000000

consider v estimate 0.00331338068513121000000

consider v envisage 0.00457798414001394000000

consider v see 0.13330622891498800000000

consider v honor 0.00169369633120184000000

consider v observe 0.00022925388524843000000

consider v deem 0.01780772472938630000000

consider v deduce 0.00000442675165851555000

consider v discuss 0.06924989793707800000000

consider v analyze 0.00110808744440385000000

consider v credit 0.00076358344500784400000

consider v reflect 0.00488260552065486000000

consider v inspect 0.00006721143721891040000

consider v reckon 0.00005880615258248060000

consider v opine 0.00001332435029753610000

consider v contemplate 0.01031886871384750000000

consider v hold 0.03135848123873220000000

consider v gauge 0.00000770070191535015000

consider v feel 0.01124220507555770000000

consider v heed 0.00092035406034561200000

pad v increase 1.00000000000000000000000

oblivion n nothing 0.75000000000000000000000

oblivion n darkness 0.25000000000000000000000

nab v arrest 0.23281485831203900000000

nab v seize 0.76650273650214000000000

nab v capture 0.00020070740759430900000

nab v detain 0.00026091962987260200000

nab v take 0.00022077814835374000000

metaphor n hope 1.00000000000000000000000

liberator n savior 1.00000000000000000000000

fiber n nap 0.00000948412228293184000

fiber n quality 0.00971869675631405000000

fiber n fibre 0.81086758016821400000000

fiber n constitution 0.00006101868827683470000

fiber n filament 0.12611037399614800000000

fiber n spirit 0.02293864303067350000000

fiber n grain 0.02910089382153540000000

fiber n hand 0.00059984883304352200000

fiber n tooth 0.00059346058351128400000

inequity n abuse 1.00000000000000000000000

far r markedly 0.00064100671648135800000

far r clearly 0.03560911991892420000000

far r extensively 0.00057910855606283900000

far r abroad 0.00176627548293935000000

far r visibly 0.00231643422425131000000

far r widely 0.00645276008904074000000

far r significantly 0.08860632181206600000000

far r obviously 0.02823539070879400000000

far r substantially 0.01055513503890530000000

far r easily 0.01555802682493960000000

far r definitely 0.01408516827517090000000

far r plainly 0.00051476316094474600000

far r considerably 0.03198764591748550000000

far r greatly 0.01177857030635600000000

far r much 0.75131427296763800000000

glance v touch 1.00000000000000000000000

nevertheless r anyhow 0.00002215728491628120000

nevertheless r still 0.25273538013951600000000

nevertheless r however 0.65731457109720300000000

nevertheless r yet 0.03549075142114780000000

nevertheless r regardless 0.00134735393146713000000

nevertheless r anyway 0.00300620159043867000000

nevertheless r nonetheless 0.05008358453531110000000

necessarily r certainly 0.17784625975330900000000

necessarily r significantly 0.07191704838813660000000

necessarily r automatically 0.75023669185855500000000

pollution n contamination 0.85909358321425900000000

pollution n corruption 0.06798557991928010000000

pollution n abuse 0.06258888573116380000000

pollution n infection 0.00876484021587642000000

pollution n deterioration 0.00156711091942118000000

dependable a stable 0.65476885479600900000000

dependable a firm 0.00141198832394268000000

dependable a responsible 0.00917792410562758000000

dependable a sure 0.05506754463376550000000

dependable a strong 0.01764985404928380000000

dependable a true 0.14261082071821300000000

dependable a good 0.07554137533093470000000

dependable a safe 0.01341388907745570000000

dependable a trustworthy 0.00070599416197133900000

dependable a reliable 0.00564795329577082000000

dependable a steady 0.00423596497182810000000

dependable a secure 0.00141198832394268000000

dependable a honest 0.00211798248591405000000

dependable a certain 0.01623786572534110000000

deafen v stun 1.00000000000000000000000

transit n carriage 0.00003471468139660400000

transit n progress 0.01074976211652250000000

transit n transfer 0.02514036016293940000000

transit n change 0.37375403630607400000000

transit n transportation 0.19683436909137900000000

transit n conversion 0.00016608941173339400000

transit n transition 0.00056635438027846300000

transit n motion 0.00037583089816340500000

transit n shift 0.00047008516274302200000

transit n emigration 0.00000680767153674378000

transit n transmission 0.00467178017121303000000

transit n immigration 0.00211993247093524000000

transit n switch 0.00008985237740344060000

transit n assumption 0.00039931777571574100000

transit n passage 0.00000680767153674378000

transit n journey 0.00010627376595308300000

transit n passing 0.00006942936279320920000

transit n movement 0.00041299080475074400000

transit n shipment 0.00306541054069542000000

transit n flight 0.00890556812914028000000

transit n transport 0.37205422704709700000000

average n middle 0.02452803259658770000000

average n run 0.00827294294776219000000

average n median 0.13367917914283100000000

average n par 0.05545787020880250000000

average n medium 0.00104155668049037000000

average n proportion 0.00046358320162953000000

average n rule 0.02680879263559430000000

average n aggregate 0.02081848228026780000000

average n norm 0.08953649296842690000000

average n tally 0.07202579924259220000000

average n standard 0.07931475293556150000000

average n midpoint 0.00059418986351178100000

average n center 0.00789201561398815000000

average n mode 0.01213503050563980000000

average n mean 0.26994011088371500000000

average n percentage 0.19749116829260000000000

bevy n array 1.00000000000000000000000

bern n berne 1.00000000000000000000000

alter v renovate 0.00000188569702445539000

alter v substitute 0.00000524466857768370000

alter v neuter 0.00000062856567481845900

alter v revise 0.01166932028830270000000

alter v falsify 0.00565944878447407000000

alter v mend 0.00000325121426611694000

alter v adjust 0.04247473153485860000000

alter v qualify 0.00014828173462697800000

alter v reconstruct 0.00000674365061053072000

alter v twist 0.00000136520293920492000

alter v correct 0.00036292788214240400000

alter v transpose 0.00000136520293920492000

alter v mutilate 0.00000273040587840985000

alter v better 0.00000674680605773045000

alter v reverse 0.00047981793767719500000

alter v convert 0.00002769207490486090000

alter v amend 0.01143652924541770000000

alter v shift 0.00924608269535978000000

alter v rework 0.00001694671003240520000

alter v mix 0.00005404388153066870000

alter v exchange 0.00007943201499335270000

alter v reform 0.00034432078015723500000

alter v regulate 0.00007248916831565270000

alter v modify 0.18708945715287700000000

alter v change 0.23405499298958400000000

alter v vary 0.00000305912019145600000

alter v reduce 0.00299956493042174000000

alter v tweak 0.00501120492217494000000

alter v transform 0.00001508692479943740000

alter v castrate 0.00000674365061053072000

alter v moderate 0.00035373027129050400000

alter v diversify 0.00000401324473212087000

alter v develop 0.00016520596722730900000

alter v turn 0.00009094133175303920000

alter v adapt 0.00002213706611641570000

alter v evolve 0.00000199376861402338000

alter v fix 0.48807984251284600000000

artistry n way 0.75000000000000300000000

artistry n art 0.12499999999999800000000

artistry n skill 0.06249999999999920000000

artistry n gift 0.06249999999999920000000

ride v intimidate 0.00180760691241270000000

ride v harass 0.00000533866649434039000

ride v bear 0.01930094771032020000000

ride v handle 0.00946471859698118000000

ride v persecute 0.00000800799974151059000

ride v dominate 0.00000266933324717020000

ride v annoy 0.00551150582209473000000

ride v perform 0.01020710223656380000000

ride v rally 0.00049729165061464600000

ride v sit 0.05969186422525990000000

ride v hound 0.00000266933324717020000

ride v travel 0.10481298257624000000000

ride v float 0.00012773039153255200000

ride v proceed 0.00042257396046657600000

ride v curb 0.00014852651835594500000

ride v mount 0.42439662563428300000000

ride v move 0.13012994356207400000000

ride v drift 0.00049462231736747600000

ride v take 0.15547375964981200000000

ride v direct 0.00000266933324717020000

ride v provoke 0.00006460216995772450000

ride v plague 0.01286018025155440000000

ride v manage 0.03174438672184720000000

ride v tolerate 0.00012386567342110900000

ride v restrain 0.00001601599948302130000

ride v tour 0.00000533866649434039000

ride v post 0.00000800799974151059000

ride v hold 0.02605526397930710000000

ride v criticize 0.00000800799974151059000

ride v control 0.00053604086441762800000

ride v drive 0.00401260095607780000000

ride v suffer 0.00205653228759973000000

abeyance n reserve 1.00000000000000000000000

yoghurt n yogurt 1.00000000000000000000000

well-founded a tenable 1.00000000000000000000000

counsel n study 0.00207093248288778000000

counsel n opinion 0.00083383441145115500000

counsel n discussion 0.00045387584276317000000

counsel n lawyer 0.63906055451366200000000

counsel n plan 0.00914484251521215000000

counsel n direction 0.00028818203019914200000

counsel n attorney 0.29920661968784700000000

counsel n recommendation 0.00052705649807732700000

counsel n warning 0.00014275334176749700000

counsel n counsellor 0.01323701816846620000000

counsel n consideration 0.00006922838655342440000

counsel n dialogue 0.00408768010522174000000

counsel n analysis 0.00013435999457982800000

counsel n conference 0.00237559541170934000000

counsel n design 0.00004990722229231820000

counsel n consultation 0.00016220798024373100000

counsel n advice 0.02815535140706640000000

ultimately r eventually 0.71044962062444100000000

ultimately r finally 0.14050960451635300000000

ultimately r primarily 0.00060069814123297000000

ultimately r someday 0.00023865642760939600000

ultimately r basically 0.12899193953834500000000

ultimately r yet 0.01539386276058810000000

ultimately r essentially 0.00198594199857389000000

ultimately r fundamentally 0.00182967599285766000000

unimportant a poor 0.00821440746442659000000

unimportant a negligible 0.03107063595316450000000

unimportant a insignificant 0.01642881492885320000000

unimportant a light 0.13143051943082600000000

unimportant a irrelevant 0.01553531797658220000000

unimportant a slight 0.01553531797658220000000

unimportant a minor 0.37106461304823600000000

unimportant a small 0.41072037322133000000000

uneconomical a uneconomic 0.99864222674813300000000

uneconomical a unprofitable 0.00135777325186694000000

unbelievably r incredibly 1.00000000000000000000000

point n site 0.00165717793406767000000

point n level 0.20187454153025400000000

point n fact 0.00956774712054435000000

point n tenor 0.00000295176157772494000

point n motivation 0.00039256352623373000000

point n constituent 0.00085462818885408200000

point n quality 0.00067766094286438400000

point n drift 0.00000809179414097986000

point n limit 0.00594979353319493000000

point n matter 0.00409648829348392000000

point n dot 0.00005325376346776110000

point n sword 0.00003094202039773430000

point n moment 0.00637883898423320000000

point n direction 0.00271264590326707000000

point n end 0.03963381494955380000000

point n notch 0.00081755378515113400000

point n heart 0.00089010038913128000000

point n kernel 0.00000033730868045527900

point n idea 0.00395065703173756000000

point n apex 0.00014357786119128300000

point n appropriate 0.00001576918081128290000

point n locality 0.00000473524564955721000

point n essence 0.00011005829868950600000

point n isthmus 0.00001131955508916270000

point n nub 0.00000101440101710945000

point n snag 0.00055335186762290800000

point n mark 0.16083890466098400000000

point n core 0.00529535170086276000000

point n vein 0.00000207486062903725000

point n focus 0.00343693256917463000000

point n pinnacle 0.00001136956763572690000

point n peak 0.01378260154793790000000

point n part 0.00493635495852200000000

point n advantage 0.00106685727296227000000

point n tongue 0.00000267294730409985000

point n component 0.00417745908329403000000

point n purpose 0.00187708046190891000000

point n period 0.03697876182116460000000

point n phase 0.00122016121933320000000

point n pointer 0.00001054674952918020000

point n juncture 0.00015013298461759300000

point n gunpoint 0.00000799830685858755000

point n aim 0.00518942026130510000000

point n area 0.02029182224019550000000

point n cause 0.00231500976224631000000

point n intention 0.00104312998379936000000

point n meat 0.00002836334792763460000

point n degree 0.00111911535988241000000

point n feature 0.00107208000159960000000

point n bill 0.00962691131875813000000

point n nib 0.00000019790413904803800

point n instant 0.00000140170496100292000

point n score 0.00252105902132900000000

point n sense 0.00085447145347402900000

point n consideration 0.00125359114408287000000

point n objective 0.00148585172044109000000

point n tone 0.00081093442572006800000

point n extent 0.00143746931611260000000

point n pike 0.00000182924058083033000

point n second 0.00637233051879016000000

point n subject 0.00133159730797999000000

point n location 0.00355681111575492000000

point n force 0.00292600113595358000000

point n bluff 0.00000549509769696376000

point n projection 0.00257899468169467000000

point n element 0.00239132700789276000000

point n value 0.02100050784530790000000

point n argument 0.00066903635900903300000

point n trait 0.00000554520643913243000

point n bottom 0.00381185138843300000000

point n specific 0.00025230689298052600000

point n ness 0.00000218748634258391000

point n stop 0.00099864787204933000000

point n spirit 0.00071832243216072500000

point n gist 0.00001366835131429560000

point n position 0.01221632209771690000000

point n spur 0.00002117638354609210000

point n suggestion 0.00083811259211624600000

point n aspect 0.00046519539523005300000

point n crux 0.00002592203072263080000

point n case 0.01089822073034370000000

point n edge 0.00047572129293220600000

point n count 0.00123694060164893000000

point n implication 0.00014421986810756500000

point n import 0.01378018273169030000000

point n strip 0.00111748957726263000000

point n head 0.00413816454892117000000

point n intent 0.00057711823418969900000

point n condition 0.00529685120279556000000

point n place 0.01109911672716480000000

point n peninsula 0.00001060359538273360000

point n side 0.00638778482837619000000

point n pitch 0.00020862382162447400000

point n hour 0.01148075864031170000000

point n duration 0.00001475287713764790000

point n detail 0.00388282484511991000000

point n property 0.00705881908458696000000

point n thought 0.00015934327033637500000

point n substance 0.00073037184082682700000

point n root 0.00013036191311746500000

point n topic 0.00202109025980894000000

point n reach 0.00089648623175223200000

point n time 0.03987928227343090000000

point n motive 0.00041789071543320700000

point n ingredient 0.00001565023895493280000

point n stretch 0.00110461499003443000000

point n base 0.00681890628202238000000

point n object 0.00020775467202391400000

point n hint 0.00046549888145151500000

point n thing 0.01910726163663980000000

point n significance 0.00036375462370934500000

point n spot 0.01253457877620190000000

point n brink 0.00000505676130773641000

point n pea 0.00001808535908825330000

point n goal 0.08635131677431870000000

point n question 0.00911199878084170000000

point n reason 0.00844119597166173000000

point n tip 0.00001240516230708970000

point n factor 0.00646812328413025000000

point n meaning 0.00021016106149909700000

point n minute 0.01917731667983230000000

point n center 0.00142961785772970000000

point n issue 0.03906825646860300000000

point n verge 0.00002469040251561460000

point n spike 0.00005097636334605180000

point n tooth 0.00003669596759612840000

point n item 0.01222455664953960000000

point n use 0.00121720339231539000000

point n characteristic 0.00008045071993091470000

point n stage 0.02997195924078500000000

cut n graft 0.00000348114118292515000

cut n slice 0.00245627712871854000000

cut n chip 0.00016066948171709300000

cut n insult 0.00002032533317326700000

cut n reduction 0.52473047542984900000000

cut n deletion 0.00000781810645256314000

cut n course 0.01148419284724990000000

cut n excerpt 0.00091197573956805900000

cut n opening 0.00401884052266392000000

cut n type 0.00071207774448989000000

cut n form 0.00100119621311857000000

cut n kickback 0.00000228100355335553000

cut n notch 0.00148760897256850000000

cut n channel 0.00046293448134730900000

cut n mark 0.07621558715408020000000

cut n scratch 0.00016884715792124100000

cut n kind 0.00818307748356837000000

cut n intersection 0.00009578661123916680000

cut n track 0.00094738274799991300000

cut n omission 0.00000369299678557514000

cut n picture 0.00389249372680745000000

cut n quota 0.00186924120573065000000

cut n fragment 0.00000036541540260996200

cut n section 0.00005671970035384960000

cut n rebuff 0.00031226938645436900000

cut n snub 0.00000713159651349718000

cut n dig 0.00003576678578616960000

cut n trench 0.00007154957863827950000

cut n abatement 0.00031902265693053300000

cut n slowdown 0.00413914983714155000000

cut n portion 0.00018284246554474400000

cut n design 0.00006893920420474640000

cut n curtailment 0.00003118197252311210000

cut n hole 0.00065372550071869800000

cut n rollback 0.00165273032253541000000

cut n sort 0.00094675395795618400000

cut n segment 0.00173578571362093000000

cut n tunnel 0.00180527475865759000000

cut n piece 0.00026049723980711500000

cut n line 0.00652026751055442000000

cut n quantity 0.00266912019348413000000

cut n style 0.00004433446270931640000

cut n shape 0.00082693631930236200000

cut n sample 0.00009206394411955650000

cut n swing 0.00187532576478821000000

cut n strip 0.00034296471840236200000

cut n fashion 0.00003268235617087520000

cut n selection 0.00017174449975878000000

cut n joint 0.00000214824839332945000

cut n slash 0.00195168366533981000000

cut n decrease 0.01356748045616830000000

cut n belt 0.00001850623581410340000

cut n passage 0.00075512681503787300000

cut n fissure 0.00000243061636490873000

cut n manner 0.00000685193970690158000

cut n chop 0.00012198022908373800000

cut n print 0.00001673175877208190000

cut n impression 0.00238581055740411000000

cut n corridor 0.00162721184199602000000

cut n wound 0.00004462765223729670000

cut n stroke 0.00066673044015988900000

cut n plate 0.00000180057911339539000

cut n construction 0.00632069665576679000000

cut n avenue 0.00004772552287775300000

cut n cutting 0.01286499506589870000000

cut n fit 0.00098709844633113600000

cut n rift 0.00003467131199993110000

cut n severance 0.00020839032034555200000

cut n mode 0.00062556170517822300000

cut n separation 0.00011442698529788600000

cut n chunk 0.00142280035806947000000

cut n ilk 0.00001922841049595870000

cut n percentage 0.01700852472951570000000

cut n quarter 0.11276606246236800000000

cut n cutback 0.02880579222123930000000

cut n tear 0.00007530776803442120000

cut n quotation 0.00008464366609495400000

cut n share 0.13305917780377400000000

cut n penetration 0.00055137130315849700000

cut n thrust 0.00014299513609724200000

dull a muted 0.00101441865247718000000

dull a off 0.00003588926789404630000

dull a bleak 0.00035144323275447600000

dull a old 0.00059796042232583000000

dull a inactive 0.00897513549295967000000

dull a flat 0.00618606755926983000000

dull a cloudy 0.00018802828067266800000

dull a listless 0.02376164106364140000000

dull a quiet 0.36805341879006300000000

dull a shallow 0.00252446316961600000000

dull a stupid 0.00040065309564517300000

dull a unreceptive 0.00001808460001158830000

dull a featureless 0.01252423235477000000000

dull a slow 0.28030286205287900000000

dull a indifferent 0.00002908174823292080000

dull a murky 0.00018997042065111100000

dull a sleepy 0.01477823426597650000000

dull a soft 0.00368224323790024000000

dull a uneventful 0.00160299373898618000000

dull a regular 0.00267654500040387000000

dull a ordinary 0.00020529634956918100000

dull a lethargic 0.01453620279796380000000

dull a dead 0.00456559906136640000000

dull a boring 0.01151079677886130000000

dull a foolish 0.00010366167778754300000

dull a black 0.00016921432672934300000

dull a abused 0.00000026425910674694100

dull a feeble 0.00003274623133062150000

dull a pointless 0.00000071344209329172100

dull a muddy 0.00000427465353619504000

dull a bearish 0.00684368245475889000000

dull a sombre 0.00003616920002317650000

dull a uninspiring 0.00003044069897311990000

dull a opaque 0.00001608169656669590000

dull a senseless 0.00000639855301062169000

dull a accustomed 0.00001038586793906260000

dull a depressing 0.00016562619217973300000

dull a commonplace 0.00009827875940523840000

dull a lifeless 0.00354130732252224000000

dull a sluggish 0.07494529158978300000000

dull a lazy 0.00008125616175297310000

dull a usual 0.00003997564300428120000

dull a dry 0.00044041235727622000000

dull a apathetic 0.00007813350411738630000

dull a stale 0.00000519293396953130000

dull a ignorant 0.00001635718601537590000

dull a unexciting 0.00019971430014156600000

dull a archaic 0.00000213285100354056000

dull a low 0.07921347182777220000000

dull a square 0.00011401202789821700000

dull a dumb 0.00000248872208554151000

dull a common 0.00024482395040470100000

dull a hazy 0.00001808460001158830000

dull a lumpy 0.00001808460001158830000

dull a bland 0.00087513923605274600000

dull a simple 0.00078224313023236400000

dull a dreary 0.00007155299911522860000

dull a familiar 0.00004441426734686870000

dull a well-worn 0.00002908174823292080000

dull a routine 0.00138627466700153000000

dull a dark 0.00004797662251719730000

dull a tiring 0.00001808460001158830000

dull a depressed 0.00212988161447008000000

dull a numb 0.00000213285100354056000

dull a empty 0.00008515532976671010000

dull a broken 0.00001164452114204350000

dull a deadly 0.00000213285100354056000

dull a heavy 0.03715938754136000000000

dull a gray 0.00002823792684554860000

dull a torpid 0.00001380709967031800000

dull a grave 0.00005425380003476500000

dull a dirty 0.00002212843583434130000

dull a gloomy 0.00027845315531946600000

dull a prosaic 0.00001808460001158830000

dull a lackadaisical 0.00028556283623104800000

dull a stagnant 0.00296363407376441000000

dull a thick 0.00016763163960488200000

dull a sad 0.00629421458330756000000
[truncated: 7,594,060 more chars]
