# Supplementary material for: Benchmarking Ontologies: Bigger or Better?
Source: PLoS Comput Biol. 2011 Jan 13;7(1):e1001055. doi: 10.1371/journal.pcbi.1001055 (PMC3020923; doi:10.1371/journal.pcbi.1001055)
Supplement: Text S1 — Additional details on methods and data. (0.17 MB PDF) [file pcbi.1001055.s004.pdf]

Benchmarking Ontology Supplement

October 21, 2010

Contents

1 **Information retrieval metrics** 1

2 **Novel metrics for evaluating ontology fitness** 2

3 **The fittest ontology of given size** 4

4 **Comparing corpora** 5

5 **Data** 7

6 **Results** 9

1 Information retrieval metrics

In the field of information retrieval (IR), the goal is to identify documents from a large collection that are most relevant to a user's query. If the subset of relevant documents is known, we can evaluate the quality of an information retrieval method using the metrics of *precision*, *recall*, *accuracy*, *fallout* and the F-measure (harmonic mean of *precision* and *recall*). To define these metrics we need to determine the true positives (tp), false positives (fp), true negatives (tn), and false negatives (fn) achieved by the retrieval method. These are defined by the cross-tabulation between relevance and retrieval: *true positives* comprise documents that are both relevant to the query and retrieved by the method; *false positives* are documents retrieved but irrelevant; *false negatives* are relevant but not retrieved; and *true negatives* include all irrelevant documents not retrieved by the method.

In the case of synonym thesauri, all the synonym pairs happening in the processed thesauri can be grouped into four categories similarly: *true positives* which refer to synonym pairs that occur in both a given thesaurus and a given corpus, *false positives* which occur in the thesaurus but not the corpus, *false negatives* which occur in the corpus but not the thesaurus (but perhaps in *some other* thesaurus), and *true negatives* which occur in neither the thesaurus nor the corpus. As discussed in the main text, such a simple transfer

of definition to ontology has issues. However we computed the following IR metrics based on these definitions, mainly for a comparison with our proposed ontology-evaluation metrics.

Based on the above definitions, some common metrics used in IR are defined as follows:

$$\text{Precision} \stackrel{\text{def}}{=} \frac{N_{tp}}{N_{tp} + N_{fp}}, \tag{1}$$

$$\text{Recall} \stackrel{\text{def}}{=} \frac{N_{tp}}{N_{tp} + N_{fn}}, \tag{2}$$

$$\text{Accuracy} \stackrel{\text{def}}{=} \frac{N_{tp} + N_{tn}}{N_{tp} + N_{tn} + N_{fp} + N_{fn}}, \tag{3}$$

$$\text{Fallout} \stackrel{\text{def}}{=} \frac{N_{fp}}{N_{tn}}, \tag{4}$$

$$F \stackrel{\text{def}}{=} 2 \cdot \frac{\text{Precision} \cdot \text{Recall}}{\text{Precision} + \text{Recall}}, \tag{5}$$

$$F_\beta \stackrel{\text{def}}{=} (1 + \beta^2) \cdot \frac{\text{Precision} \cdot \text{Recall}}{(\beta^2 \cdot \text{Precision} + \text{Recall})}. \tag{6}$$

$F_\beta = F$ when $\beta = 1$. F_2 weights recall twice as much as precision and $F_{\frac{1}{2}}$ weights precision twice as much as recall. The results are listed in Table 2 in the *Results* section.

2 Novel metrics for evaluating ontology fitness

For a given reference corpus T, we define the *complete ontology* $\mathcal{O}(\mathcal{C}_T, \mathcal{R}_T)$ which incorporates all N concepts encountered in the corpus and all the relations between them. We also derive from the corpus T, a frequency f_i for each concept in $\mathcal{C}_T$ and an association probability p_{ij} for each relation in $\mathcal{R}_T$. f_i should be normalized in such a way that $\sum_{i \in C_T} f_i = \sum_{i=1}^{N} f_i = 1$ and by definition (See section one), p_{ij} is normalized so that $\sum_{j=1}^{M_i} p_{ij} = 1$ for a given concept i. In implementation, we (under)approximate the complete

ontology (thesaurus) with the union of thesauri, excluding concepts and relations not found in the corpus.

To evaluate an arbitrary ontology, $X = \{C_X, R_X\}$, regarding to corpus T, we can identify sets $C_X(tp)$, and $R_X(tp)$, such that $C_X(tp) = C_X \cap \mathcal{C}_T$, and $R_X(tp) = R_X \cap \mathcal{R}_T$.

This allows us to replace integer N_{tp} with real-valued weight $W_{C_X(tp)}$ such that

$$W_{C_X(tp)}(T) \stackrel{\text{def}}{=} \sum_{i \in C_X(tp)} f_i, \tag{7}$$

If we expand this measure to also account for relation importance, it becomes

$$W_{C_X(tp)\ R_X(tp)}(T) \stackrel{\text{def}}{=} \sum_{i \in C_X(tp)} \sum_{j \in C_X(tp)} \sum_{k(i) \in R_X(tp)} f_i p_{k|ij}, \tag{8}$$

where $p_{k|ij}$ is equal to the association probability between concepts i and j, p_{ij}, if a relation between them exists in X, and is zero otherwise.

Similarly we define $C_X(fn) = C_T - C_X(tp)$ and $R_X(fn) = R_T - R_X(tp)$ and get

$$W_{C_X(fn)\ R_X(fn)}(T) \stackrel{\text{def}}{=} \sum_{i \in C_X(fn)} \sum_{j \in C_X(fn)} \sum_{k \in R_X(fn)} f_i p_{k|ij}, \tag{9}$$

Now we are able to introduce our first ontology-evaluation measure –*breadth*– to capture the theoretical coverage of an ontology:

$$Breadth_X^2(T) \stackrel{\text{def}}{=} \frac{\sum_{i \in C_X(tp)} \sum_{j \in C_X(tp)} \sum_{k \in R_X(tp)} f_i p_{k|ij}}{\sum_{i' \in C_X(tp)} \sum_{j' \in C_X(tp)} \sum_{k' \in R_X(tp)} f_{i'} p_{k'|i'j'} + \sum_{i'' \in C_X(fn)} \sum_{j'' \in C_X(fn)} \sum_{k'' \in R_X(fn)} f_{i''} p_{k''|i''j''}} \tag{10}$$

Because every concept and its relations in a corpus either happen in the ontology (tp) or not (fn), equation (10) can be simplified as follows:

$$
\begin{aligned}
Breadth_X{}^2(T) &= \frac{\displaystyle\sum_{i\in C_X(tp)} \sum_{j\in C_X(tp)} \sum_{k\in R_X(tp)} f_i p_{k|ij}}{\displaystyle\sum_{i'\in C_T} \sum_{j'\in C_T} \sum_{k'\in R_T} f_{i'} p_{k'|i'j'}} \\
&= \frac{\displaystyle\sum_{i\in C_X(tp)} \sum_{j\in C_X(tp)} \sum_{k\in R_X(tp)} f_i p_{k|ij}}{\displaystyle\sum_{i'\in C_T} \sum_{j'\in C_T} f_{i'} \sum_{k'\in R_T} p_{k'|i'j'}} \\
&= \sum_{i\in C_X(tp)} \sum_{j\in C_X(tp)} \sum_{k(i)\in R_X(tp)} f_i p_{k|ij} \qquad (11) \\
&= W_{C_X(tp)\ R_X(tp)}(T). \qquad (12)
\end{aligned}
$$

This approach of weighing importance works as intended for N_{tp} and N_{fn}, but not for N_{fp} and N_{tn} because the corresponding f_i's all equal zero in the corpus.

We can further modify this measure of theoretical coverage to account also for parsimony, and thus develop a general measure of *Depth* of ontology X with respect to corpus T:

$$
\begin{aligned}
\text{Depth}_X^2(T) &\stackrel{\text{def}}{=} \frac{\text{Breadth}_X^2(T)}{\text{Number of relations in } X} \qquad (13) \\
&= \frac{\displaystyle\sum_{i\in C_X(tp)} \sum_{j\in C_X(tp)} \sum_{k\in R_X(tp)} f_i p_{k|ij}}{|R_X|}. \qquad (14)
\end{aligned}
$$

In the case of an ontology, *Depth* translates into the average probability mass (in a corpus) for each concept relation. Large ontologies would tend to have a better value of *Breadth*, but not necessarily a better *Depth*. This is because a large ontology may be padded with very rare concepts and relations lowering its fit to the corpus compared to a small ontology containing only the most frequent ones.

Finally, we can create a more general measure $Depth_\beta$ that allows flexibility in the specification of ontological coverage and parsimony, such that

$$
\text{Depth}_{X,\beta}(T) = \frac{[\text{Breadth}_X]^{(2-\beta)}}{|R_X|^\beta} \qquad (15)
$$

In implementation, we tried $\beta = 0.5, 0.75, 1.5$ for this equation. The results are presented in Table 2 of the *Results* section.

3 The fittest ontology of given size

We can then define the *fittest ontology of fixed size*, $\mathcal{O}_{c\,r}\left(T, C, R, \left\{f_i, \{p_{ij}\}_{j=1,\ldots,M_i'}\right\}_{i=1,\ldots,c}\right)$ with a predetermined c concepts and r relations ($r = \sum_{i\in c} M_i'$) such that $C \subset \mathcal{C}_{\mathcal{T}}$, $R \subset \mathcal{R}_{\mathcal{T}}$, and $Breadth_{\mathcal{O}_{c\,r}}(T)$ is maximized over all possible sets C and R of sizes c and r, correspondingly.

For an arbitrary ontology $O_{c\,r}$, we would like to benchmark it using the fittest ontology of the same size, $\mathcal{O}_{c\,r}$. Once we have estimated its $Breadth_{O_{c\,r}}$ and $Depth_{O_{c\,r}}$ for a given corpus T, we can compute the *loss measures* relative to its fittest counterpart:

$$\text{Breadth Loss}_{O_{c\,r}}(T) = \text{Breadth}_{\mathcal{O}_{c\,r}}(T) - \text{Breadth}_{O_{c\,r}}(T), \tag{16}$$

$$\text{Depth Loss}_{O_{c\,r}}(T) = \text{Depth}_{\mathcal{O}_{c\,r}}(T) - \text{Depth}_{O_{c\,r}}(T). \tag{17}$$

To ease computation, we can define simplified versions of these measures that constrain only the number of relations, r:

$$\text{Breadth Loss}_{O_{*\,r}}(T) = \text{Breadth}_{\mathcal{O}_{*\,r}}(T) - \text{Breadth}_{O_{*\,r}}(T), \tag{18}$$

$$\text{Depth Loss}_{O_{*\,r}}(T) = \text{Depth}_{\mathcal{O}_{*\,r}}(T) - \text{Depth}_{O_{*\,r}}(T), \tag{19}$$

where $*$ indicates that c is not constrained. These results are also summarized in Table 2.

The strength of the loss measure is its ability to compare a specific ontology to the *Depth*-optimized ontology of the same size, rather than one significantly larger or smaller. In theory, this could allow us to benchmark ontologies covering domains for which there may be no competing ontologies. The challenge with this in practice is that if there are no competing ontologies, then there is no superset of concepts and relations from which to draw into an optimal $\mathcal{O}_{c\,r}$ other than $O_{c\,r}$ itself. If we wanted to prune an ontology of its weakest parts, however, we could obtain the fittest sub-ontology $\mathcal{O}_{\gamma\,\phi}$, by specifying γ concepts and ϕ relations so that the *Depth* reaches its maximum for the given γ and ϕ.

4 Comparing corpora

In addition to comparing ontologies relative to the corpora they describe, we can compare different corpora with respect to one or more ontologies. Let T_1 and T_2 indicate two distinct

corpora, such as 19th Century English novels and 20th Century scholarly medical articles. We can define the *distance* between the two corpora with respect to headword h_i and its M_i synonyms by calculating the Minkowski distance with corpora-specific parameter estimates p_{ij} in the following way.

$$d_{T_1,T_2}(\mathrm{h}_i) \stackrel{\mathrm{def}}{=} \left[\sum_{j=1}^{M_i} |p_{ij}^{(T_1)} - p_{ij}^{(T_2)}|^r \right]^{\frac{1}{r}}. \tag{20}$$

Or

$$d_{T_1,T_2}(\mathrm{h}_i) \stackrel{\mathrm{def}}{=} \left[\sum_{j=1}^{M_i} |f_i^{(T_1)} p_{ij}^{(T_1)} - f_i^{(T_2)} p_{ij}^{(T_2)}|^r \right]^{\frac{1}{r}}. \tag{21}$$

In our practical implementations of this measures (we used both of the above equations in our practical experiments), we used $r = 1$ (the Manhattan distance), and $r = 2$ (the Eucleadean distance).

The three-way distance for three corpora, T_1, T_2, and T_3 is then just a sum of three pairwise distances.

$$d_{T_1,T_2,T_3}(\mathrm{h}_i) \stackrel{\mathrm{def}}{=} d_{T_1,T_2}(\mathrm{h}_i) + d_{T_1,T_3}(\mathrm{h}_i) + d_{T_2,T_3}(\mathrm{h}_i). \tag{22}$$

In our three-corpus example, the most interesting headwords to visualize are those with maximum $d_{T_1,T_2,T_3}(\mathrm{h}_i)$, which have the substitution probability estimates most unlike each other across the three corpora.

We can also define the overall distance between two corpora.

$$D_{T_1,T_2} \stackrel{\mathrm{def}}{=} \sum_{i=1}^{N} d_{T_1,T_2}(\mathrm{h}_i). \tag{23}$$

With this approach, we can compute a taxonomy or phylogeny of several corpora using a distance-matrix to construct the tree.

We can also calculate the entropy of synonyms in corpus T in bits. This captures the ambiguity or linguistic richness of a corpus with respect to a thesaurus.

$$H_T \stackrel{\text{def}}{=} -\sum_{i=1}^{N} f_i^{(T)} \sum_{j=1}^{M_i} p_{ij}^{(T)} \log_2 p_{ij}^{(T)}. \tag{24}$$

Finally, for symmetry, we can whimsically imagine the generation of a nonsense *fittest* corpus, which is completely consistent with a given ontology or thesaurus. That such a corpus would tend to be very redundant (or very small) highlights the limited representation most ontologies and thesauri provide of their domains, but also the collective importance of low-frequency relationships in modeling them.

5 Data

We used three very different corpora to illustrate our approaches.

1) *Medicine*: Clinical journal article abstracts from PubMed database.
Based on the clinical queries service offered by PubMed (`http://www.ncbi.nlm.nih.gov/corehtml/query/static/clinicaltable.html`), we generated a modified query:

```
   ((clinical[Title/Abstract] AND trial[Title/Abstract]) OR
clinical trials[MeSH Terms] OR clinical trial[Publication Type] OR
random*[Title/Abstract] OR random allocation[MeSH Terms] OR
therapeutic use[MeSH Subheading]) OR (sensitiv*[Title/Abstract]
OR sensitivity and specificity[MeSH Terms] OR
diagnos*[Title/Abstract] OR diagnosis[MeSH:noexp] OR
diagnostic * [MeSH:noexp] OR diagnosis,differential[MeSH:noexp] OR
diagnosis[Subheading:noexp])
```

By limiting ourselves only to English abstracts in the core clinical journals for the whole period covered by PubMed, up to Feb 25, 2009, we downloaded 786,180 clinical medicine-related abstracts.

2) *News*: Reuters News corpus

The Reuters corpus covered news stories between 08/20/1996 and 08/19/1997.

3) *Literature*: 19th century literature – written in English or translated to English.
We compiled a subjective list of the 50 best books of the 19th century based on the information from `http://www.goodreads.com/list/show/16.Best_Books_of_the_19th_Century`.

We then obtain the flat text files of these books from www.gutenberg.org (see Table 1).

Table 1: Contents of the *Literature* corpus.

Title	*Author*	*English translator*
Emma	Austen, Jane	
Mansfield Park	Austen, Jane	
Northanger Abbey	Austen, Jane	
Persuasion	Austen, Jane	
Pride and Prejudice	Austen, Jane	
Title Sense and Sensibility	Austen, Jane	
The Tenant of Wildfell Hall	Bront, Anne	
Jane Eyre	Bront, Charlotte	
Villette	Bront, Charlotte	
Wuthering Heights	Bront, Charlotte	
Alice's Adventures in Wonderland	Carroll, Lewis	
Through the Looking-Glass	Carroll, Lewis	
The Awakening and Selected Short Stories	Chopin, Kate	
The Woman in White	Collins, Wilkie	
Heart of Darkness	Conrad, Joseph	
A Christmas Carol	Dickens, Charles	
A Tale of Two Cities	Dickens, Charles	
Bleak House	Dickens, Charles	
David Copperfield	Dickens, Charles	
Great Expectations	Dickens, Charles	
Little Dorrit	Dickens, Charles	
Our Mutual Friend	Dickens, Charles	
Crime and Punishment	Dostoyevsky, Fyodor	Garnett, Constance
The Brothers Karamazov	Dostoyevsky, Fyodor	Garnett, Constance

Notes from the Underground	Dostoyevsky, Fyodor	unknown
A Study in Scarlet	Doyle, Arthur Conan, Sir	
The Count of Monte Cristo	Dumas pre, Alexandre	
Madame Bovary	Flaubert, Gustave	Aveling, Eleanor Marx
Far from the Madding Crowd	Hardy, Thomas	
Tess of the d'Urbervilles	Hardy, Thomas	
The Mayor of Casterbridge	Hardy, Thomas	
The Scarlet Letter	Hawthorne, Nathaniel	
Les Misrables	Hugo, Victor	Hapgood Isabel Florence
A Doll's House	Ibsen, Henrik	
Moby Dick, or, the whale	Melville, Herman	
Frankenstein	Shelley, Mary Wollstonecraft	
Treasure Island	Stevenson, Robert Louis	
Dracula	Stoker, Bram	
Vanity Fair	Thackeray, William Makepeace	
Anna Karenina	Tolstoy, Leo, graf	Garnett, Constance
War and Peace	Tolstoy, Leo, graf	Maude, Aylmer Maude, Louise Shanks
A Connecticut Yankee in King Arthur's Court	Twain, Mark	
Adventures of Huckleberry Finn	Twain, Mark	
The Adventures of Tom Sawyer	Twain, Mark	
The Prince and the Pauper	Twain, Mark	
The Tragedy of Pudd' nhead Wilson	Twain, Mark	
The Time Machine	Wells, H. G. (Herbert George)	
The War of the Worlds	Wells, H. G. (Herbert George)	
The Importance of Being Earnest	Wilde, Oscar	

6 Results

See three additional Tables with results that were not included into the main text.

Table 2: Statistics.

Measure[1]	Corpus	The synonym finder	New World Roget's A-Z thesaurus	WordNet	21st Century Synonym And Antonym Finder	The Oxford dictionary of synonyms and antonyms	A Dictionary of Synonyms and Antonyms	Scholastic Dictionary of Synonyms, Antonyms and Homonyms
Precision	Medicine	0.405	0.335	0.182	0.543	0.625	0.576	0.692
Precision	Novels	0.569	0.424	0.202	0.718	0.701	0.833	0.898
Precision	News	0.610	0.473	0.261	0.779	0.807	0.821	0.876
Recall	Medicine	0.690	0.248	0.126	0.179	0.149	0.074	0.031
Recall	Novels	0.726	0.235	0.104	0.177	0.125	0.080	0.030
Recall	News	0.697	0.235	0.120	0.172	0.129	0.071	0.026
Accuracy	Medicine	0.568	0.594	0.531	0.683	0.693	0.680	0.679
Accuracy	Novels	0.641	0.527	0.430	0.611	0.595	0.592	0.576
Accuracy	News	0.635	0.500	0.405	0.573	0.561	0.540	0.524
Fallout	Medicine	0.968	0.314	0.376	0.079	0.045	0.027	0.007
Fallout	Novels	0.741	0.328	0.467	0.057	0.043	0.013	0.003
Fallout	News	0.735	0.331	0.479	0.049	0.030	0.015	0.004
$F_{\beta=1}$	Medicine	0.510	0.285	0.149	0.270	0.240	0.131	0.059
$F_{\beta=1}$	Novels	0.638	0.303	0.137	0.285	0.212	0.147	0.058
$F_{\beta=1}$	News	0.651	0.314	0.165	0.282	0.222	0.131	0.051
$F_{\beta=2}$	Medicine	0.605	0.262	0.134	0.207	0.176	0.090	0.038
$F_{\beta=2}$	Novels	0.688	0.258	0.115	0.209	0.150	0.098	0.037
$F_{\beta=2}$	News	0.678	0.261	0.135	0.204	0.155	0.087	0.032
$F_{\beta=.5}$	Medicine	0.441	0.313	0.167	0.386	0.381	0.245	0.130
$F_{\beta=.5}$	Novels	0.595	0.365[3]	0.170	0.446	0.364[8]	0.290	0.132
$F_{\beta=.5}$	News	0.625	0.393[2]	0.212	0.457	0.393[1]	0.264	0.117
Breadth	Medicine	0.521	0.385	0.260	0.150	0.284	0.091	0.060

[1] Changes in ranking of a measure across three corpora are highlighted in red. Font size reflects the ranking of results, the best results shown with the largest font, the worst with the smallest.

Breadth	Novels	0.550	0.344	0.174	0.168	0.227	0.083	0.055
Breadth	News	0.529	0.369	0.251	0.158	0.337	0.098	0.056
Breadth Loss	Medicine	0.375	0.511	0.636	0.746	0.612	0.800	0.785
Breadth Loss	Novels	0.286	0.491	0.661	0.668	0.608	0.752	0.746
Breadth Loss	News	0.388	0.548	0.664	0.760	0.579	0.802	0.764
$Depth(\cdot 10^{-6})$	Medicine	0.686	1.168	0.849	1.023	2.682	1.584	3.012
$Depth(\cdot 10^{-6})$	Novels	0.725	1.045	0.570	1.145	2.147	1.450	2.792
$Depth(\cdot 10^{-6})$	News	0.698	1.120	0.819	1.073	3.181	1.706	2.818
$Depth_{17,\beta=.5}(\cdot 10^{-4})$	Medicine	4.314	4.163	2.399	1.520	4.651	1.144	1.033
$Depth_{17,\beta=.5}(\cdot 10^{-4})$	Novels	4.684	3.521	1.318	1.799	3.332	1.001	0.922
$Depth_{17,\beta=.5}(\cdot 10^{-4})$	News	4.421	3.910	2.271	1.632	6.008	1.279	0.935
$Depth_{17,\beta=.75}(\cdot 10^{-5})$	Medicine	1.721	2.206	1.427	1.247	3.532	1.346	1.764
$Depth_{17,\beta=.75}(\cdot 10^{-5})$	Novels	1.843	1.918	0.866	1.435	2.675	1.205	1.604
$Depth_{17,\beta=.75}(\cdot 10^{-5})$	News	1.756	2.093	1.364	1.323	4.372	1.477	1.623
$Depth_{17,\beta=1.5}(\cdot 10^{-8})$	Medicine	0.109	0.328	0.301	0.689	1.546	2.194	8.783
$Depth_{17,\beta=1.5}(\cdot 10^{-8})$	Novels	0.112	0.310	0.246	0.729	1.384	2.099	8.457
$Depth_{17,\beta=1.5}(\cdot 10^{-8})$	News	0.110	0.321	0.295	0.706	1.684	2.277	8.496
$Depth\ Loss(\cdot 10^{-5})$	Medicine	0.049	0.155	0.208	0.508	0.578	1.404	4.107
$Depth\ Loss(\cdot 10^{-5})$	Novels	0.038	0.149	0.216	0.455	0.574	1.312	3.813
$Depth\ Loss(\cdot 10^{-5})$	News	0.051	0.166	0.217	0.518	0.548	1.412	3.935

Table 3: Overlaps between thesauri (headwords).

Name X	*Name Y*	*Name Z*	X	Y	Z	$X \cap Y$	$Y \cap Z$	$X \cap Z$	$X \cap Y \cap Z$
finder	rogets	wordnet	20,249	29,925	115,201	15,945	17,594	16,501	13,700
finder	rogets	21 century	20,249	29,925	7,507	15,945	6,749	6,613	6,170
finder	rogets	oxford	20,249	29,925	8,487	15,945	7,498	7,681	7,103
finder	rogets	synonyms	20,249	29,925	3,771	15,945	3,540	3,626	3,457
finder	rogets	scholastic	20,249	29,925	2,147	15,945	2,044	2,085	2,018
finder	wordnet	21 century	20,249	115,201	7,507	16,501	6,494	6,613	5,853
finder	wordnet	oxford	20,249	115,201	8,487	16,501	7,527	7,681	6,951
finder	wordnet	synonyms	20,249	115,201	3,771	16,501	3,429	3,626	3,335
finder	wordnet	scholastic	20,249	115,201	2,147	16,501	1,966	2,085	1,929
finder	21 century	oxford	20,249	7,507	8,487	6,613	4,101	7,681	3,914

finder	21 century	synonyms	20,249	7,507	3,771	6,613	2,231	3,626	2,205
finder	21 century	scholastic	20,249	7,507	2,147	6,613	1,359	2,085	1,343
finder	oxford	synonyms	20,249	8,487	3,771	7,681	2,470	3,626	2,441
finder	oxford	scholastic	20,249	8,487	2,147	7,681	1,652	2,085	1,641
finder	synonyms	scholastic	20,249	3,771	2,147	3,626	1,259	2,085	1,249
rogets	wordnet	21 century	29,925	115,201	7,507	17,594	6,494	6,749	5,930
rogets	wordnet	oxford	29,925	115,201	8,487	17,594	7,527	7,498	6,792
rogets	wordnet	synonyms	29,925	115,201	3,771	17,594	3,429	3,540	3,261
rogets	wordnet	scholastic	29,925	115,201	2,147	17,594	1,966	2,044	1,892
rogets	21 century	oxford	29,925	7,507	8,487	6,749	4,101	7,498	3,846
rogets	21 century	synonyms	29,925	7,507	3,771	6,749	2,231	3,540	2,180
rogets	21 century	scholastic	29,925	7,507	2,147	6,749	1,359	2,044	1,334
rogets	oxford	synonyms	29,925	8,487	3,771	7,498	2,470	3,540	2,406
rogets	oxford	scholastic	29,925	8,487	2,147	7,498	1,652	2,044	1,624
rogets	synonyms	scholastic	29,925	3,771	2,147	3,540	1,259	2,044	1,244
wordnet	21 century	oxford	115,201	7,507	8,487	6,494	4,101	7,527	3,679
wordnet	21 century	synonyms	115,201	7,507	3,771	6,494	2,231	3,429	2,063
wordnet	21 century	scholastic	115,201	7,507	2,147	6,494	1,359	1,966	1,251
wordnet	oxford	synonyms	115,201	8,487	3,771	7,527	2,470	3,429	2,307
wordnet	oxford	scholastic	115,201	8,487	2,147	7,527	1,652	1,966	1,543
wordnet	synonyms	scholastic	115,201	3,771	2,147	3,429	1,259	1,966	1,174
21 century	oxford	synonyms	7,507	8,487	3,771	4,101	2,470	2,231	1,558
21 century	oxford	scholastic	7,507	8,487	2,147	4,101	1,652	1,359	1,080
21 century	synonyms	scholastic	7,507	3,771	2,147	2,231	1,259	1,359	885
oxford	synonyms	scholastic	8,487	3,771	2,147	2,470	1,259	1,652	1,053

Table 4: Overlaps between thesauri (synonym pairs).

Name X	*Name Y*	*Name Z*	X	Y	Z	$X \cap Y$	$Y \cap Z$	$X \cap Z$	$X \cap Y \cap Z$
finder	rogets	wordnet	758,611	329,669	306,472	97,204	20,804	39,094	14,591
finder	rogets	21 century	758,611	329,669	146,806	97,204	46,323	72,833	28,093
finder	rogets	oxford	758,611	329,669	105,902	97,204	30,914	56,054	23,885
finder	rogets	synonyms	758,611	329,669	57,366	97,204	21,821	32,390	15,900
finder	rogets	scholastic	758,611	329,669	19,759	97,204	7,650	13,031	6,422
finder	wordnet	21 century	758,611	306,472	146,806	39,094	13,511	72,833	9,942
finder	wordnet	oxford	758,611	306,472	105,902	39,094	13,714	56,054	10,292
finder	wordnet	synonyms	758,611	306,472	57,366	39,094	6,000	32,390	5,167
finder	wordnet	scholastic	758,611	306,472	19,759	39,094	2,959	13,031	2,617

finder	21 century	oxford	758,611	146,806	105,902	72,833	24,624	56,024	18,300
finder	21 century	synonyms	758,611	146,806	57,366	72,833	15,787	32,390	12,390
finder	21 century	scholastic	758,611	146,806	19,759	72,833	6,804	13,031	5,622
finder	oxford	synonyms	758,611	105,902	57,366	56,024	10,617	32,390	9,217
finder	oxford	scholastic	758,611	105,902	19,759	56,024	5,347	13,031	4,747
finder	synonyms	scholastic	758,611	57,366	19,759	32,390	7,521	13,031	6,091
rogets	wordnet	21 century	329,669	306,472	146,806	20,804	13,511	46,323	6,003
rogets	wordnet	oxford	329,669	306,472	105,902	20,804	13,714	30,914	6,699
rogets	wordnet	synonyms	329,669	306,472	57,366	20,804	6,000	21,821	3,499
rogets	wordnet	scholastic	329,669	306,472	19,759	20,804	2,959	7,650	1,749
rogets	21 century	oxford	329,669	146,806	105,902	46,323	24,624	30,914	11,178
rogets	21 century	synonyms	329,669	146,806	57,366	46,323	15,787	21,821	8,801
rogets	21 century	scholastic	329,669	146,806	19,759	46,323	6,804	7,650	3,577
rogets	oxford	synonyms	329,669	105,902	57,366	30,914	10,617	21,821	6,559
rogets	oxford	scholastic	329,669	105,902	19,759	30,914	5,347	7,650	3,297
rogets	synonyms	scholastic	329,669	57,366	19,759	21,821	7,521	7,650	4,292
wordnet	21 century	oxford	306,472	146,806	105,902	13,511	24,624	13,714	4,718
wordnet	21 century	synonyms	306,472	146,806	57,366	13,511	15,787	6,000	2,667
wordnet	21 century	scholastic	306,472	146,806	19,759	13,511	6,804	2,959	1,462
wordnet	oxford	synonyms	306,472	105,902	57,366	13,714	10,617	6,000	2,664
wordnet	oxford	scholastic	306,472	105,902	19,759	13,714	5,347	2,959	1,563
wordnet	synonyms	scholastic	306,472	57,366	19,759	6,000	7,521	2,959	1,543
21 century	oxford	synonyms	146,806	105,902	57,366	24,624	10,617	15,787	4,748
21 century	oxford	scholastic	146,806	105,902	19,759	24,624	5,347	6,804	2,570
21 century	synonyms	scholastic	146,806	57,366	19,759	15,787	7,521	6,804	3,432
oxford	synonyms	scholastic	105,902	57,366	19,759	10,617	7,521	5,347	2,963
